# Probably less than one-tenth of the genes produce only the wild type protein without at least one additional protein isoform in some human cancer cell lines

**DOI:** 10.18632/oncotarget.20015

**Published:** 2017-08-07

**Authors:** Rui Yan, Ju Zhang, Lucas Zellmer, Lichan Chen, Di Wu, Siqi Liu, Ningzhi Xu, Joshua D. Liao

**Affiliations:** ^1^ Nephrology Department, Guizhou Medical University Hospital, Guiyang, P.R. China; ^2^ CAS Key Laboratory of Genome Sciences and Information, Beijing Institute of Genomics, Chinese Academy of Sciences, Beijing, P.R. China; ^3^ Hormel Institute, University of Minnesota, Austin, Minnesota, USA; ^4^ Beijing Protein Innovation Co., Ltd, Beijing, P.R. China; ^5^ Laboratory of Cell and Molecular Biology & State Key Laboratory of Molecular Oncology, National Cancer Center/Cancer Hospital, Chinese Academy of Medical Sciences & Peking Union Medical College, Beijing, P.R. China; ^6^ Department of Pathology, Guizhou Medical University Hospital, Guiyang, P.R. China

**Keywords:** proteomics, mass spectrometry, protein isoform, western blotting, immunohistochemical staining

## Abstract

To estimate how many genes produce multiple protein isoforms, we electrophoresed proteins from MCF7 and MDA-MB231 (MB231) human breast cancer cells in SDS-PAGE and excised narrow stripes of the gel at the 48kD, 55kD and 72kD. Proteins in these stripes were identified using liquid chromatography and tandem mass spectrometry. A total of 765, 750 and 679 proteins from MB231 cells, as well as 470, 390 and 490 proteins from MCF7 cells, were identified from the 48kD, 55kD and 72kD stripes, respectively. We arbitrarily allowed a 10% technical variation from the proteins’ theoretical molecular mass (TMM) and considered those proteins with their TMMs within the 43-53 kD, 49-61 kD and 65-79 kD ranges as the wild type (WT) expected from the corresponding stripe, whereas those with a TMM above or below this range as a smaller- or larger-group, respectively. Only 263 (34.4%), 269 (35.9%) and 151 (22.2%) proteins from MB231 cells and 117 (24.9%), 135 (34.6%) and 130 (26.5%) proteins from MCF7 cells from the 48kD, 55kD and 72kD stripes, respectively, belonged to the WT, while the remaining majority belonged to the smaller- or larger-groups. Only about 3-16%, on average about 10% regardless of the stripe and cell line, of the proteins appeared in only one stripe and within the WT range, while the remaining preponderance appeared also in additional stripe(s) or had a larger or smaller TMM. We conclude that few (fewer than 10%) of the human genes produce only the WT protein without additional isoform(s).

## INTRODUCTION

A large number of genes in the human genome undergo alternative initiation or termination of transcription to generate different RNA transcripts. Over 95% of the human genes contain exons and introns, and 95% of these genes undergo alternative splicing to generate different mRNAs [1]. One mRNA may also be expressed to different protein isoforms via various mechanisms such as use of an alternative start codon or stop codon, as we recently reviewed [2]. Because of these and other mechanisms, in most cases one gene often produces multiple protein isoforms [3, 4], which provides the gene with multiple ways to diversify its functions and in turn allows the cell to be more flexible adapting to the environment. However, a good estimation on how many genes in the human genome produce multiple protein isoforms is still lacking, mainly due to the lack of convenient but reliable technology, although there have been frequent attempts [5].

Liquid chromatography together with tandem mass spectrometry, or LC-MS/MS, is a common approach for protein identification in a high throughput manner and has resulted in a huge amount of data [6–8], which should facilitate annotating protein-coding genes to a large extent [9] and have clinical value [10]. Routine LC-MS/MS is conducted in a bottom-up manner [11], in which intact proteins within the SDS-containing polyacrylamide gel (SDS-PAGE) are digested with a protease into short peptides, whereupon the short peptides are identified using LC-MS/MS in combination with a match of the MS data to a peptide reference. In an earlier study, we excised a narrowstripe of SDS-PAGE at 26 kD or 40 kD for LC-MS/MS analysis [12]. To our surprise, results from this sort of top-down approach of LC-MS/MS showed that only one-third to one-fourth of the proteins migrated in SDS-PAGE as anticipated from their theoretical molecular mass (TMM), while the vast rest of the proteins were those with a larger or a smaller TMM. Moreover, many proteins appeared in both of the 26kD and 40kD stripes, including some having a much larger or smaller TMM than 26kD or 40kD, indicating that these genes have one or more additional isoforms besides the wild type (WT) protein, i.e. the protein with its TMM expected in the 26 kD or 40kD stripe [12].

Western blotting (WB) is a common method for protein identification and semi-quantification, involving fractionation of proteins through SDS-PAGE, transfer of the proteins from the gel onto a membrane, and identification of the protein in question as a band on the membrane using a specific antibody [3]. As already explained in detail previously [12], proteins are known to be subject to many different types of chemical modification after synthesized in the ribosomes, including phosphorylation, de-phosphorylation, glycosylation, SUMOylation, ubiqulation, proteolysis, etc. More complexly, some of these modifications, such as phosphorylation, can occur at many sites of a single protein. Because of these complex post-translational modifications, together with the aforementioned relations at the transcriptional, post-transcriptional and translational levels, most genes will likely produces multiple bands in WB, in a large part because of multiple protein isoforms. However, ignoring this fact, most published studies reporting WB data present only one single band on the membrane, which implies to the readers that the gene produces only one single form of protein, with attribution of all observed functions of the gene to this one form of protein without discussing the possible contribution of other sibling proteins [4, 12].

In the present study, we continued to use our simplified top-down approach to analyze proteins isolated from SDS-PAGE at larger (48kD, 55kD and 72kD) molecular weights. The resulting data not only dovetail with our previous findings at lower ranges of SDS-PAGE but also further suggest that few genes in the human genome produce only the WT protein without at least one additional isoform.

## RESULTS

### The rates of peptide spectra matched to the reference were low

LC-MS/MS identified 43462-57078 spectra as clear inputs from different gel stripes of the two cell lines (Table 1). However, only 11.74-21.13% of these clear inputs could be matched to the reference and led to identification of proteins, whereas the remaining inputs were unmatchable. Adjustment of the cutoff parameters could modify the ratios of the matched to the unmatchable but could not change the fact that most inputs were unmatchable.

**Table 1:** The rates of the inputs matched to the reference

Stripe	MB231			MCF7		
	Search input	PSMs	Match rate	Search input	PSMs	Match rate
72kDa	51414	9416	18.31%	45504	7624	16.75%
55kDa	53899	11345	21.05%	48052	5640	11.74%
48kDa	57078	12063	21.13%	43462	7228	16.63%

PSMs: peptide spectrum matches.

### In SDS-PAGE, most proteins did not migrate as expected from their TMMs

LC-MS/MS identified 765, 750 and 679 proteins from MB231 cells, as well as 470, 390 and 490 proteins from MCF7 cells, from the 48kD, 55kD and 72kD stripes, respectively (Table 2; Supplementary Data 1). We arbitrarily set a 10% divergence at the left or the right side of the TMM as the allowed variation, which is a lax criterion allowing those proteins with a TMM within the 43-53 kD, 49-61 kD and 65-79 kD ranges to be the WT, or the expected, in the 48kD, 55kD and 72kD stripes, respectively. As we described before [12], those proteins with their TMMs above or below these ranges were categorized into a smaller-group or a larger-group, respectively, because their positions in the gel were smaller or larger, respectively, than their TMMs (Table 2).

**Table 2:** Fraction of LC-MS/MS identified proteins by their TMMs

Cell line	48kD stripe		55kD stripe		72kD stripe	
	TMM (kD)	Number of proteins	TMM (kD)	Number of proteins	TMM (kD)	Number of proteins
MB231	>53 (smaller)	178 (23.3%)^*a*^	>61 (smaller)	117 (15.6%)^*a*^	>79 (smaller)	89 (13.1%)^*a*^
	43-53 (WT)	263 (34.4%)	49-61 (WT)	269 (35.9%)	65-79 (WT)	151 (22.2%)
	<43 (larger)	324 (42.4%)^*a*^	<49 (larger)	364 (48.5%)^*a*^	<65 (larger)	439 (64.7%)^*a*^
	Total	765 (100%)	Total	750 (100%)	Total	679 (100%)
MCF7	>53 (smaller)	274 (58.3%)	>61 (smaller)	189 (48.5%)	>79 (smaller)	134 (27.3%)
	43-53 (WT)	117 (24.9%)	49-61 (WT)	135 (34.6%)	65-79 (WT)	130 (26.5%)
	<43 (larger)	79 (16.8%)	<49 (larger)	66 (16.9%)	<65 (larger)	226 (46.1%)
	Total	470 (100%)	Total	390 (100%)	Total	490 (100%)

*a*: significantly different from its counterpart from MCF7 cells (p<0.01; χ^2^ test).

There only were 263/765 (34.4%), 269/750 (35.9%) and 151/679 (22.2%) proteins identified from the 48kD, 55kD and 72kD stripes, respectively, from MB231 cells with their TMMs within the WT ranges, while the remaining 502 (65.5%), 481 (64.1%), and 528 (77.8%) proteins, respectively, belonged to either the larger-group or the smaller-group (Table 2). Similarly, there only were 117/470 (24.9%), 135/390 (34.6%) and 130/490 (26.5%) proteins identified from the 48kD, 55kD and 72kD stripes, respectively, from MCF7 cells with their TMMs within the WT ranges, while the remaining 353 (75.1%), 255 (65.4%) and 360 (73.5%) proteins, respectively, belonged to either the larger-group or the smaller-group (Table 2).

**Figure 1:**
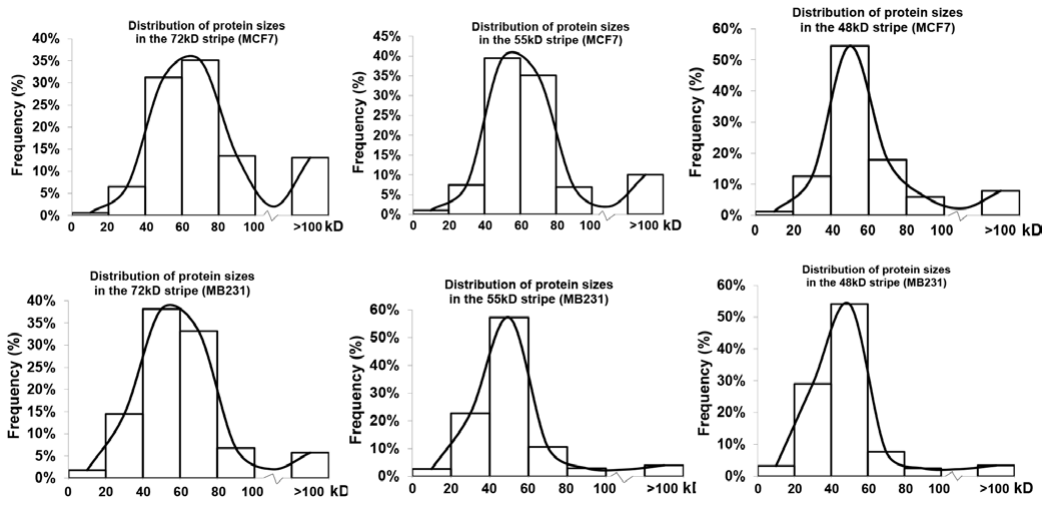
Distribution of proteins identified from each stripe from each cell line according to their TMMs Note that the three distribution curves from MCF7 cells have a higher right tail but a lower left tail compared with the curve from MB231 cells.

The larger-group and smaller-group showed significant differences between the two cell lines: MCF7 cells had 58.3%, 48.5% and 27.3% of the total proteins identified from the 48kD, 55kD and 72kD stripes, respectively, with their TMMs larger than the WT ranges, percentages which were significantly higher than their counterparts from MB231 cells that were 23.3%, 15.6% and 13.1%, respectively (Table 2). Reciprocally, MCF7 cells had 16.8%, 16.9% and 46.1% of the total proteins from the 48kD, 55kD and 72kD, respectively, with their TMMs below the WT ranges, significantly lower than their counterparts from MB231 cells that were 42.4%, 48.5% and 64.7%, respectively (Table 2). We plotted the number of the proteins from each stripe according to their TMMs, and the resulting distribution curve from MCF7 cells showed a higher right tail but a lower left tail, compared with the curve from MB231 cells (Figure 1), which dovetailed with the above described difference in the percentages of the larger- and smaller-groups between the two cell lines.

### A smaller coverage rate might indicate that a protein lacks part(s) of the sequence

**Figure 2:**
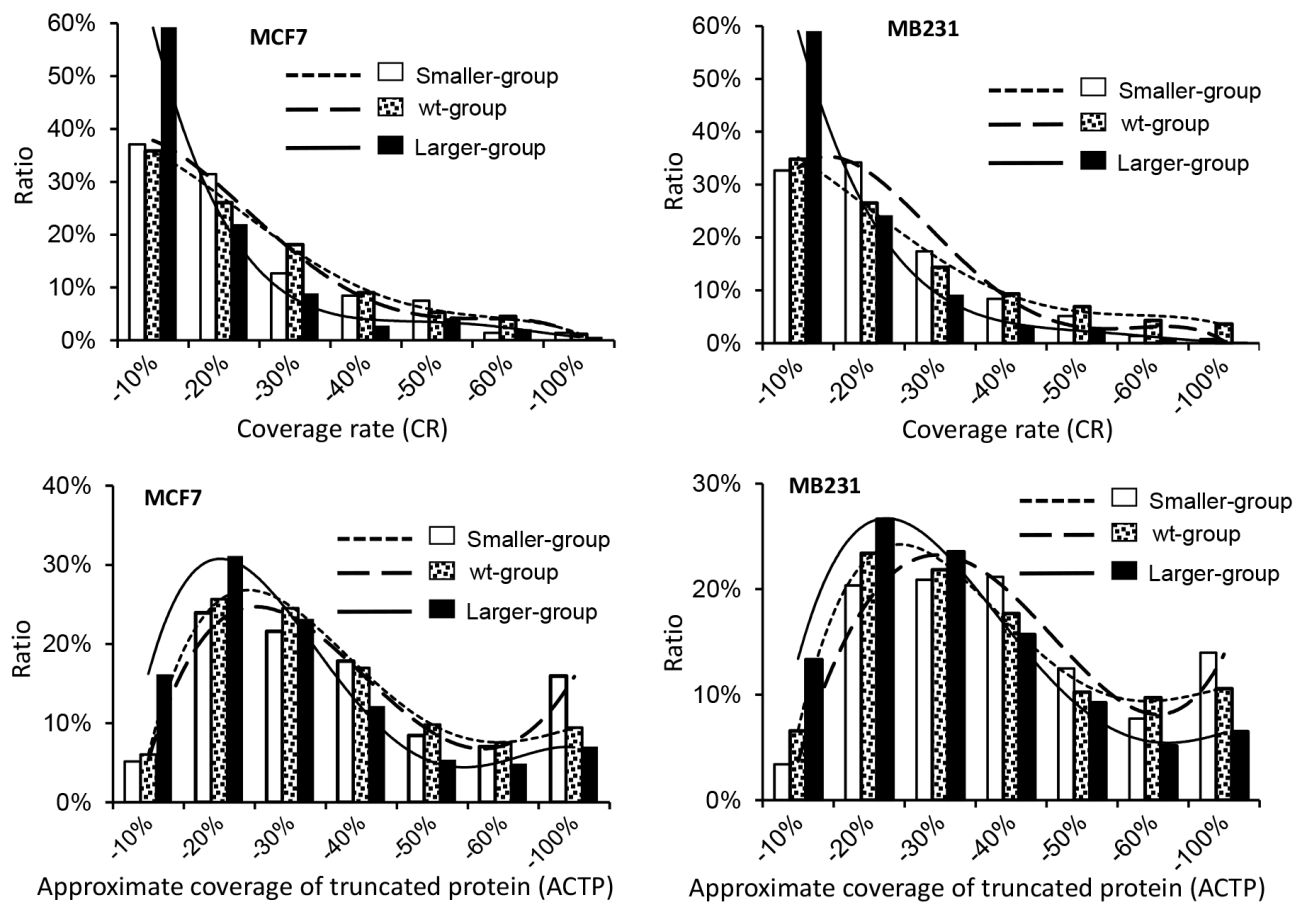
Coverage rate (CR) and approximate coverage of truncated protein (ACTP) of the proteins from different stripes from MB231 or MCF7 cells

A total of 1435 and 906 proteins from all three stripes from MB231 and MCF7 cells, respectively, contained two or more unique LC-MS/MS-identified peptides and thus had a definite identity. For each of these proteins we calculated its coverage rate (CR) of identified peptides (Table 3 and Figure 2), i.e. the total amino acids (AAs) of all identified peptides as the percentage of the total AAs of the WT protein [12]. The averages of the CRs of the 1435 and 906 proteins were 17.96% and 15.94%, respectively. The averages of the CRs of the WT proteins, i.e. those proteins with a TMM within the WT range, were 21.21% and 19.27% from MB231 and MCF7 cells, respectively, significantly higher than the 10.40% and 14.11% averages of the CRs of the proteins in the smaller-group, i.e. those with a TMM above the WT ranges, from MB231 and MCF7 cells, respectively. Actually, 40.43% and 38.11% of the WT proteins from MB231 and MCF7 cells, respectively, had a CR >20%, higher than the CRs of 12.22% and 18.74% of the proteins with a TMM above the WT range. In general, proteins with smaller TMMs had a higher CR than proteins with larger TMMs (Figure 1), as we reported previously [12].

**Table 3:** Average of coverage rate (ACR) and CR>20% of different protein groups

Cell line	MB231				MCF7			
Group	Total	Smaller	WT	Larger	Total	Smaller	WT	Larger
ACR	17.96%	10.40%	21.21%*	17.95%	15.94%	12.67%	19.27%*	18.20%
CR>20%	32.82%	12.22%	40.04%*	33.91%	27.48%	18.74%	38.11%*	31.46%

*: Significantly higher than the smaller-group (p<0.05, Wilcoxon rank sum test due to large variation).

For a given protein, the LC-MS/MS-identified peptides should be randomly distributed across the protein. Therefore, the “approximate coverage of truncated proteins” (ACTP), calculated as the coverage of the predicted truncated proteins with its first and last identified AAs as the N- and C-terminators, has been adopted to analyze the dispersion of the identified peptides across the protein and further estimate the distribution of those isoforms smaller than the WT form [12]. The results showed that compared with CR, ACTP of the smaller isoforms was much closer to that of the WT protein (Figure 2), countenancing the conclusion that there might be many smaller isoforms with an N- or C-terminal truncation. However, neither CR nor ACTP could distinguish those proteins larger than their TMMs from the WT proteins. Moreover, since ACTP could not mask the effect of the alternative splicing of the exons encoding the middle region of the protein, ACTP distribution of those proteins with smaller TMMs could not coincide with that of the WT proteins.

### One-tenth of the proteins meet two criteria, i.e. within the WT range and solely in one stripe

Of the 470, 390 and 490 proteins identified from the 48kD, 55kD and 72kD stripes, respectively, from MCF7 cells, only 183 (38.9%), 50 (12.8%) and 173 (35.3%) appeared solely in the stripe, coined herein as “unique proteins” (Table 4), while the remaining 287 (61.1%), 340 (87.2%) and 317 (64.7%) proteins, respectively, appeared in one or two additional stripes. Of these 183, 50 and 173 unique proteins, only 42, 11 and 54, respectively, had their TMMs within the WT ranges, which constituted 23.0%, 22.0% and 31.2%, respectively, of the unique proteins but only 8.9%, 2.8% and 11.0% of the total proteins identified from the corresponding stripe (Table 5). The remaining 141 (77.0% of the unique but 30.0% of the total), 39 (78.0% of the unique but 10.0% of the total) and 119 (68.8% of the unique but 24.3% of the total) proteins had their TMMs out of the WT ranges and thus might have their WT protein at another position of the gel, if the WT one was expressed.

**Table 4:** Number of proteins identified in one or more stripes

Stripe(s)	MCF7		MB231		
48kD only	183 (23.5%)	407 (52.2%)	224	17.7%	619 (49.0%)
55kD only	52 (6.7%)		134	10.6%	
72kD only	172 (22.1%)		261	20.7%	
48- & 55-kD	60 (7.7%)	181 (23.2%)	226	17.9%	357 (28.3%)
48- & 72-kD	35 (4.5%)		28	2.2%	
55- & 72-kD	86 (11.0%)		103	8.2%	
48- & 55- & 72-kD	192	24.6%	287		22.7%
Total Proteins	780	100.0%	1263		100.0%

**Table 5:** Number of proteins identified in only one single stripe

Stripe	MCF7			MB231		
	Total	Unique (%)	WT unique (% of total)	Total	Unique (%)	WT unique (% of total)
48kD	470	183 (38.9%)	42 (8.9%)	765	223 (29.2%)	85 (11.1%)
55kD	390	50 (12.8%)	11 (2.8%)	750	134 (17.9%)	76 (10.1%)
72kD	490	173 (35.3%)	54 (11.0%)	678	260 (38.3%)	108 (15.9%)
All stripes	1350	406 (30.1%)	107 (7.9%)	2193	617 (28.1%)	269 (12.3%)

Similarly, of the 765, 750 and 679 proteins identified from the 48kD, 55kD and 72kD stripes, respectively, from MB231 cells, only 223 (29.2%), 134 (17.9%), and 260 (38.3%) appeared solely in the stripe (Table 4), while the remaining 542 (70.8%), 616 (82.1%), and 419 (61.7%) proteins, respectively, were also detected in one or two additional stripes. Of these 223, 134 and 260 unique proteins, only 85, 76 and 108, respectively, had their TMMs within the WT ranges, which constituted 38.1%, 56.7% and 41.5%, respectively, of the unique proteins but only 11.1%, 10.1% and 15.9% of the total proteins identified in the corresponding stripe (Table 5). The remaining 138 (61.9% of the unique but 18.0% of the total), 58 (43.3% of the unique but 7.7% of the total) and 152 (58.5% of the unique but 22.4% of the total) proteins had their TMMs out of the WT ranges and thus might have their WT protein somewhere else in the gel, if the WT one was expressed.

On average, only 28.1% (from MB231) and 30.1% (from MCF7) of the proteins from all three stripes were detected solely in a single stripe, regardless whether they migrated as expected from their TMMs, with the remaining 71.9% (from MCF7) and 69.9% (from MB231) of the proteins appeared in at least one additional stripe (Table 5). If only those unique proteins that were within the WT range were counted, the percentage declined to 7.9% for MCF 7 cells and 12.3% for MB231 cells (Table 4), and averaging both cell lines, i.e. the 7.9% and 12.3%, got 10.1%, or about one-tenth. On the other hand, 24.6% (from MCF7) and 22.7% (from MB231) of the proteins identified by LC-MS/MS appeared in all three stripes (Table 4), and averaging both cell lines, i.e. the 24.6% and 22.7%, got 23.7%, or about one-fourth.

A significantly larger number of proteins appeared in two neighboring stripes than in two more-distant stripes, i.e. more proteins appeared in both 48kD and 55kD and in both 55kD and 72 kD than in both 48kD and 72kD (Table 4). This result suggests not only that the appearance of the same-gene-derived proteins in two different positions of SDS-PAGE is less-likely due to random degradation but also that different protein isoforms more often have a relatively smaller, than a larger, difference in their size, as we explained before [12].

### Examples of protein multiplicity included PLEC, AHNAK, histone 4H and cytochrome C

**Figure 3:**
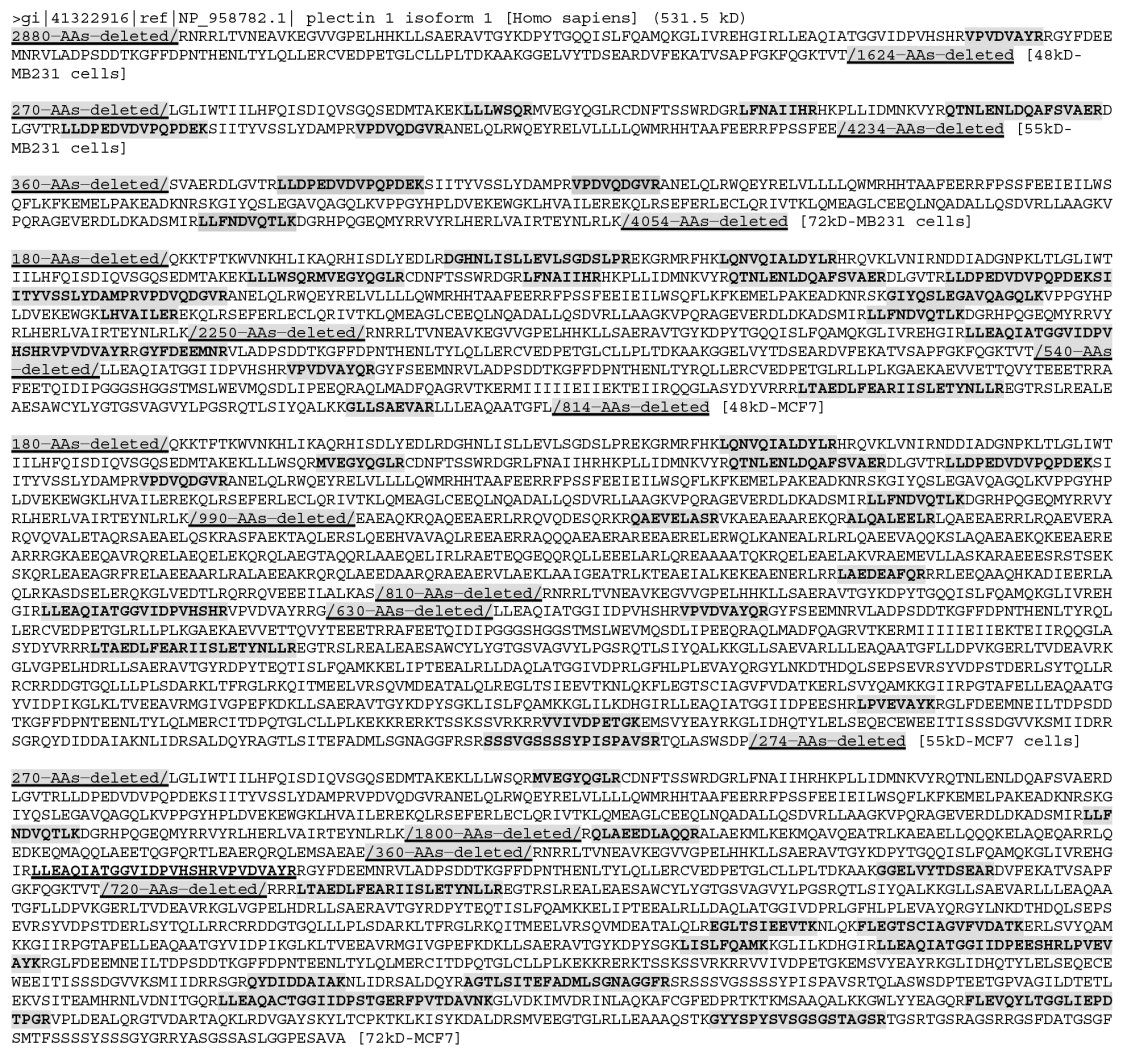
Sequences with the LC-MS/MS-detected (boldfaced and shaded) peptides that are unique to the Plectin-1 isoform 1 (PLEC), with the regions lacking any detected peptide not shown (i. e. deleted to shorten the length) A peptide (LLEAQIATGGVIDPVHSHRVPVDVAYR; boldfaced and underlined) that is not unique to PLEC (and might also appear in other proteins) was also detected in the 72kD stripe from MCF7 cells. In all three stripes from MB231 cells, these detected peptides are mainly localized to the middle region of PLEC. In contrast, in all three stripes from MCF7 cells, the detected peptides are localized to the N- and C-terminal regions, but not to the middle region, roughly speaking.

Plectin-isoform-1 gene (PLEC) has 22 mRNA variants listed in the NCBI database that encode 22 protein isoforms varying between 3429-4684 AAs, with the WT protein of 4684 AAs weighing 531.5 kD. We detected PLEC protein in all three stripes from both cell lines, with great differences between the two cell lines and among the three stripes from the same cell line, in the number and dispersion of the LC-MS/MS-detected peptides (Figure 3 and Supplementary Data 2): In MB231 cells, the detected peptides were localized roughly to the middle region of PLEC in the 48kD stripe but to the vicinity of the boundary between the N-terminal and middle regions in the 55kD and 72kD stripes. In contrast, there were many more LC-MS/MS-detected peptides that were widely distributed across PLEC protein from the three stripes from MCF7 cells. However, a caveat needs to be given that our method is not an authentic top-down approach and thus it cannot be ruled out that more than one isoform existed in the same stripe from MCF7 cells.

**Figure 4:**
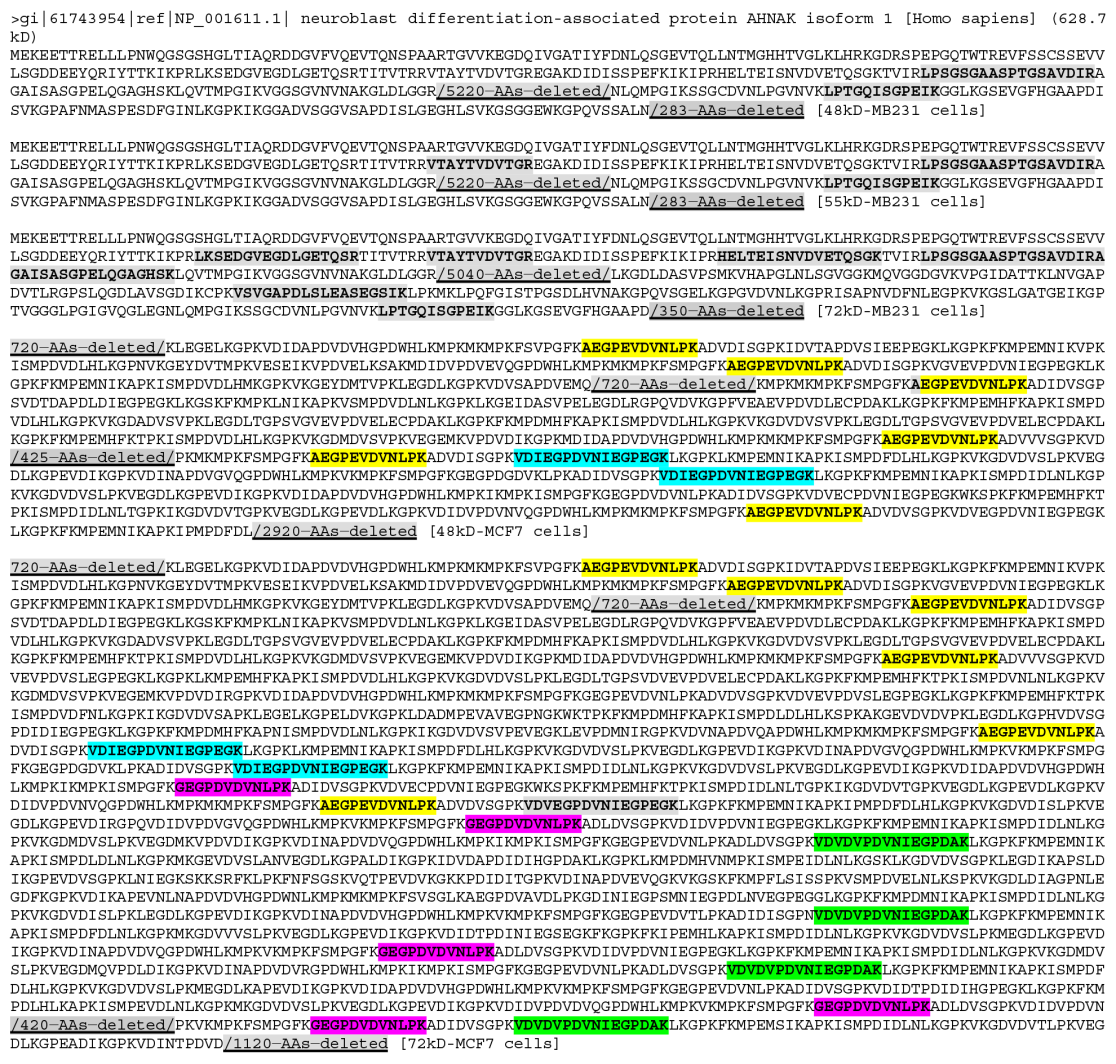
The LC-MS/MS-detected (boldfaced and shaded) peptides of AHNAK protein, with the regions lacking a detected peptide (shaded and underlined) not shown (i. e. deleted to shorten the length) In the three stripes from MB231 cells, the detected peptides were localized to the regions near the N- and C-termini, with a large (5040-5220 AAs) middle region without any detected peptide. In contrast, the detected peptides were localized to the N-terminal and middle regions, but not to the C-terminal region, in the 48kD and 72kD stripes from MCF7 cells. It was not detected in the 55kD stripe from MCF7 cells. Note that in the 48kD stripe from MCF7 cells there are actually only two peptide sequences detected, each (coded with a color) with repeats. Similarly, in the 72kD stripe there are actually only five different peptide sequences detected (coded with different colors), four of which have repeats. These repeats raise the possibility that the protein isoforms detected in these stripes may not be as large as shown in the figure, because some repeat-containing regions might not be there and because there may be more than one isoforms in the same gel stripe that were identified by LC-MS/MS collectively.

Another gene, “neuroblast differentiation-associated protein AHNAK isoform 1”, has nine mRNAs and also nine protein isoforms listed in the NCBI database. One isoform is as short as 149 AAs with a TMM of ∼16 kD, while the remaining eight isoforms vary between 4073-5890 AAs with their TMMs varying between 433-629 kD. Although these nine isoforms are either too small or too large to appear in any of our gel stripes, AHNAK was detected in almost all stripes from both cell lines expect the 55kD stripe from MCF7 cells (Figure 4 and Supplementary Data 2). In the three stripes from MB231 cells, the LC-MS/MS-detected peptides were localized roughly to the N- and C-terminal regions with a large middle region varying from 5040 to 5220 AAs without any detected peptide (Figure 4). In contrast, in the 48kD and 72kD stripes from MCF7 cells, the LC-MS/MS-detected peptides were localized roughly to the middle region, although there still were large regions without any detected peptide (Figure 4). Moreover, in the 48kD stripe from MCF7 cells, there were only two unique peptides detected, i.e. the AEGPEVDVNLPK and the VDIEGPDVNIEGPEGK sequences, but the AEGPEVDVNLPK had six repeats while the VDIEGPDVNIEGPEGK had two repeats detected. In the 72kD stripe from MCF7 cells, there were five unique peptides detected, one of which had one repeat and three of which had multiple repeats (Figure 4). These repeats raise the possibility that the actual AHNAK protein(s) in the stripe might not be as large as shown in Figure 4, since these repeated peptides might only come from one or some, but not all, regions of AHNAK. Moreover, there might be more than one isoform in each stripe, each of which might be relatively small.

We mapped LC-MS/MS identified peptides onto the 40 proteins with the largest TMMs from each stripe of each cell line (Supplementary Data 2), and found that many proteins had large region(s) without any LC-MS/MS-identified peptide, similar to PLEC and AHNAK proteins. This finding dovetails with our previous observations at lower (26-40 kD) ranges of SDS-PAGE [12]. Since it is unlikely that such a large region lacks a trypsin digestion site, we conclude that these regions may not actually be present in the proteins, due to various reasons such as alternative splicing of RNA or proteolysis of protein, which in turn might be a reason why many proteins were detected at a much lower position of SDS-PAGE than their TMMs. As exemplified by PLEC and AHANK, in most of these 40 proteins with the largest TMMs, LC-MS/MS-identified peptides for the same genes were not identical among different stripes from the same cell line or between the stripes at the same molecular weight from different cell lines (Supplementary Data 2). One possible explanation is that different protein isoforms of the same gene, such as the PLEC, exist in different stripes from the same cell line or exist in the same stripe from different cell lines.

**Figure 5:**
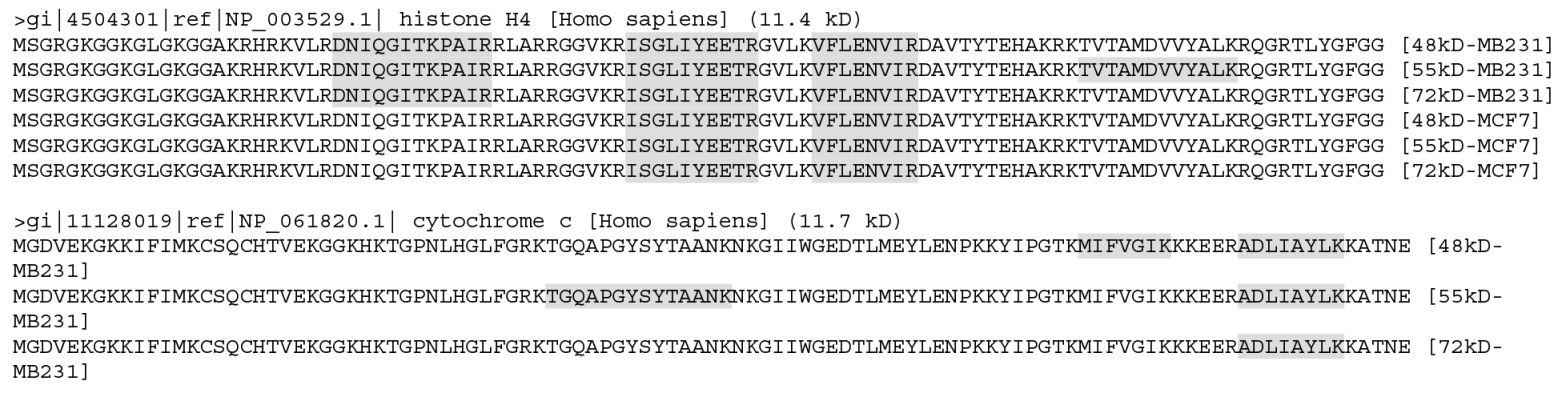
The LC-MS/MS-detected (boldfaces and shaded) peptides unique to the histone H4 (top panel) or cytochrome c (bottom panel) Note that the detected peptides of histone H4 were similar between the 48kD and 72kD stripes but differ between the 55kD stripe and these two stripes from MB231 cells. The detected peptides were the same among all the three stripes from MCF7 cells but differ from any of the stripes from MB231 cells. The detected sequences of cytochrome c differ among the three stripes from MB231 cells.

According to the NCBI database, the histone H4 (HIST1H4A) gene is only expressed to one mRNA that produces only one protein with a TMM of 11.4 kD, which should not appear in any of the three stripes. However, it was detected from all three stripes from both cell lines (Figure 5), suggesting that besides the 11.4 kD WT protein (if it was expressed), it may have at least three larger isoforms. Similarly, the NCBI database shows that the cytochrome c (CYCS) gene has only one mRNA that produces only one protein of 11.7 kD, but it was detected in all three stripes from MB231 cells, suggesting that it might have three larger isoforms, besides the WT protein at 11.7 kD needed by the cell. Cytochrome c was not detected in any of the three stripes from MCF7 cells, which most likely suggests that these larger isoforms may be cell-line specific, unlike the histone H4. We mapped LC-MS/MS identified peptides onto 20 proteins with the smallest TMMs from each stripe from each cell line, and found many cases similar to histone H4 and cytochrome c (Supplementary Data 2).

## DISCUSSION

### There are usually many unmatchable inputs in LC-MS/MS data

In all LC-MS/MS studies of proteins, there is always a large percentage of clear inputs that cannot be matched to the peptide reference. As discussed before [12], the reasons for this phenomenon have not yet been fully clarified but are likely to be multiple, including technical artifacts. One reason may be that all current algorithms for translation of mRNA to protein have many flaws and cannot annotate many protein-coding mRNAs [2, 12]. Indeed, protein products of a large number of already-annotated genes have not yet been identified [13], and all algorithms exclude those open reading frames smaller than 100 codons, although the existence of proteins much shorter than 100 AAs has been well documented [4–16]. Moreover, most, if not all, algorithms use only 20 amino acids shown on the genetic code list, despite that there are at least 22 proteinogenic amino acids [17]. This situation in turn is because we still know too little about translation process, although we have known much more about gene transcription and RNA processes, including RNA splicing [2, 18, 19]. It cannot be ruled out that some of the unmatchable inputs reflect some unknown protein isoforms produced via different mechanisms, such as unknown cis- or trans-splicing derived mRNAs, [2, 18, 19] unknown start or stop codon usage for protein translation, protein splicing, etc. In addition, translational errors [20], which occur more often in cancer cells, may also make some clear inputs unmatchable.

### Few genes are expressed to only the WT protein without a sibling isoform

An important finding of our study is that only about 3-16%, or on average ∼10%, of the total proteins identified had their TMMs within the WT range and appeared solely in one stripe (Table 5), while the vast rest of the proteins either have a TMM far away from the WT range or appear also in other stripe(s). These results in general dovetail with the our previous findings that only about one-fourth to one-third of the proteins from HEK293 cells at the 26kD and 40kD of a SDS-PAGE were at the WT range, regardless whether they appeared solely in the stripe [12]. Some of the proteins in the smaller-group, i.e. those appearing at a site of SDS-PAGE lower than their TMMs, may be nonspecifically degraded products, but most of them may not be, in part because the chance for the nonspecifically degraded products appear fortunately in all the three gel positions we analyzed is very low, especially for many proteins, as reasoned before [12]. Moreover, altered proteolysis may occur in the cell lines used, due to genetic alterations occurring such as during the decades of culture of the cell lines used, which may also contribute to the appearance of some proteins in the smaller-group. The reasons for the occurrence of the larger-group, i.e. appearing at a position of SDS-PAGE higher than their TMMs, are much less known, although some of them may derive from different types of post-translational modifications, as exemplified by glycosylation of mu opioid receptor [21]. Theoretically, all different types of post-translational modification can occur in a single protein, and one single type, typically phosphorylation, can occur at many sites of a single protein as well, which together can dramatically shift the position of a particular protein in SDS-PAGE. While this situation still awaits experimental verification, we surmise that it may not occur to a large percentage of proteins expressed in a single cell line. Moreover, adding all these post-translational modifications together still may not explain the observation that some proteins like histone H4 and cytochrome are gel-shifted so greatly, from about 11 kD to 72 kD.

Considering that we have only studied three narrow stripes, it is very likely that many of these 10% of genes may still have other protein products at other positions of the SDS-PAGE that were not analyzed herein. Therefore, the actual percentage of those genes that meet the two criteria, i.e. within the WT range and solely in one stripe, is very likely to be much smaller than 10%. Moreover, expression of many genes has cell- or tissue-specificity and varies among different developmental, physiological and pathological situations, whereas we have only studied two cell lines herein and another cell line (HEK293) previously that were cultured in a routine condition. Therefore, the actual percentage may be much lower than 10% genome-wide and regardless of cell- and tissue-specificity and of different developmental, physiological and pathological situations. In other words, probably few genes produce only the WT protein without a sibling, although a recent analysis of some LC-MS/MS databases suggests that most highly expressed genes produce only a single dominant protein isoform [22]. Our astonishing conclusion awaits further verification by an authentic top-down LC-MS/MS approach [23, 24], especially the newer version [11]. A caveat needs to be given that a 10% divergence to each side of TMM is actually larger than most WB may produce. Narrowing it, such as to 5%, will greatly shrink the WT-group and in turn enlarge the larger- and smaller-groups, making the conclusion even more astounding.

### Dispersion of identified peptides across to the WT protein is a useful criterion to distinguish different cells

Unlike most proteomic studies using a LC-MS/MS approach, we localize LC-MS/MS-identified peptides onto the corresponding WT protein and calculate the CR and ACTP. A higher CR indicates that the protein isoform detected is closer to the WT form in terms of sequence length, or has a higher chance to be the WT form. As shown by examples in Figures 4 and 5, although proteins derived from the same gene were identified at the same position of SDS-PAGE from different cell lines, the differences in CR as well as in dispersion of identified peptides suggest that this gene may produce different protein isoforms in different cell lines. Therefore, mapping identified peptides onto the WT protein sequence to determine the dispersion and CR can be a good approach to distinguish different cells, even cells from the same tissue origin such as mammary gland derived MB231 and MCF7. This is of significance because distinguishing cells of the same tissue origin usually is not as easy as differentiating cells from different tissue origins. This approach utilizes a huge number of proteins as biomarkers and thus may be more reliable than the routine method that only uses single or several biomarkers. In addition, the distribution curve of LC-MS/MS-identified proteins according to their TMMs, as shown in Figure 1, may also be used as a collective biomarker to distinguish cells from the same tissue origin, as evidenced by the difference between the MB231 and MCF7 cells.

### In most cases of WB, it creates a bias to cut away additional bands

The overarching conclusion that few genes produces only the WT protein without additional isoform(s) challenges our routine practice of WB. In most published results of WB, only a narrow band on the membrane was presented, which implies to the readers that the isoform of interest, usually the WT form, is the sole one expressed. However, usually there are additional band(s) on the membrane but they are regarded as “artifacts” without a convincing proof and thus are cut away to make the data explanation more straightforward, as pointed out recently [4]. Even worse, blamed by the customers for selling “not specific enough” antibodies, antibody producing companies try hard to select and market those antibodies that gave rise to only a single band on WB, while researchers also elect these “more specific” antibodies. This alliance between antibody suppliers and their customers will likely extinguish, via a “natural selection”, those antibodies that react to more isoforms and, meanwhile, mislead the biomedical fraternity to a distorted picture of the gene in question. In our opinion, many, but certainly not all, of those antibodies blamed to have poor specificity may actually reflect more faithfully the truth and provide us with a better global picture of the gene in question. Actually, some published studies with WB did detect and presented some bands larger than the TMM [25]. In our practice of WB, we routinely employ multiple antibodies and present all bands shown on the membrane [26–29].

### In most cases, correctly interpreting immunohistochemical data still remains impossible

Another alert set off by our findings pertains to immunohistochemistry and even immunocytochemistry, because on a glass slide the specificity of the signals yielded by the antibody used cannot be authenticated by the molecular weight of the targeted protein. Protein multiplicity is often associated with cell-type specificity, subcellular specificity (i.e. varying among different organelles or locations within the cell), and situation-specificity [4]. For instance, a smaller isoform of cyclin E derived from proteolysis of the WT protein is mainly localized to the cytoplasm of cancer cells, while the WT protein is mainly localized to the nucleus [30]. Moreover, in a routine WB, a protein is denatured but on a paraffin-embedded section it has a conformation close to the native status, owing to a quick fixation by formalin. This disparity disqualifies WB from endorsing the specificity of an antibody for immunohistochemical staining of paraffin-embedded sections. Because of these currently unsolved technical bottlenecks, although a positive signal resulting from a well-conducted staining is likely to indicate the presence of protein product(s) of the gene in question, in most cases it cannot tell which protein isoform(s) give rise to the signal. This situation greatly diminishes the power of immunohistochemistry in exploration into the biological function or mechanism of genes. The results and ensuing interpretations are convincing only in those studies on those well-characterized isoforms of well-characterized genes with a confirmed isoform-specific antibody, such as some p53 protein isoforms and some relevant antibodies [31]. Unfortunately, the number of such genes is very small, in sharp contrast to a plethora of published immunohistochemical data with antibodies not yet well-characterized for the isoform-specificity.

Few published studies involving immuno histochemical staining put enough attention on the protein multiplicity and confirm the isoform-specificity of the antibody used. In fact, many, if not most, researchers do not even know how many protein isoforms their target gene may produce and what similarity and disparity among these isoforms are, in terms of protein sequence. In most published studies on immunohistochemical data, the antibody used is assumed, although rarely declared, to recognize only a single protein isoform and thus all the positive staining is accredited to it. This situation greatly worries us as it has hardly been discussed in detail in the literature. Development of a good strategy to solve these technical bottlenecks is a prerequisite and an imperative for researchers to determine not only whether the gene of interest is expressed to a protein but also which protein isoform(s) are expressed.

## MATERIALS AND METHODS

### Protein sample preparation

MCF7 and MDA-MB231 (MB231) human breast cancer cell lines were cultured routinely in a Dulbecco’s modified eagle medium containing 10% fetal bovine serum. As described before [12], cells at about 80% confluence were washed with 1x phosphate buffered saline and then harvested via scraping in a lysis buffer containing 1x Proteinase Inhibitor Cocktail (Sigma-Aldrich, Inc, St. Louis, MS). After centrifugation to precipitate insoluble components, proteins were collected and diluted with a gel-loading buffer containing 2% SDS and 2% 2-mercaptoethanol as the final concentration. The protein samples were boiled for 5 min and then quickly cooled down on ice.

### SDS-PAGE and protein collection

**Figure 6:**
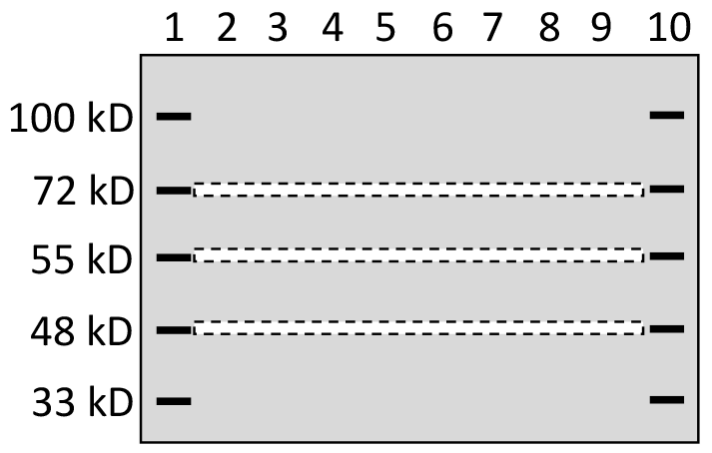
Depiction of gel stripe excision A 10% SDS-PAGE that was 2-cm longer in the vertical direction than regular mini-gels was loaded with a pre-stained protein marker in lanes 1 and 10 and with protein samples from one cell line in the lanes 2-9. After electrophoresis, narrow gel stripes of 2-mm in width (narrow dash-lined boxes) were excised out, using a surgical blade and with help of two rulers, for protein digestion and ensuing LC-MS/MS analysis.

To better separate the proteins, a 10% SDS-PAGE was made with 10x10.5 cm glass plates included in the Hoeffer SE260 vertical slab gel system (Hoeffer Inc; http://www.hoeferinc.com/), which produced a gel 2-cm longer in the vertical direction than the gel made using regular mini-gel cast systems of Hoeffer and other companies. The first and last wells of the gel were loaded with a pre-stained protein marker, while each remaining well was loaded with 50 μg of protein sample as illustrated in Figure 6. One gel was loaded with proteins from MCF7 cells while another was loaded with proteins from MB231 cells. The two gels were electrophoresed at the same time using the same power supplier. Guided by two rulers, narrow stripes, each about 2-mm in width, were excised out with a surgical blade from the gel at positions of 72kD, 55kD and 48kD indicated by pre-stained markers, as illustrated in Figure 6. These positions were selected for several reasons: First, our pre-stained protein markers showed these positions, which allowed us to precisely excise a narrow gel stripe at these positions. Second, this 48-72kD range resides in the middle of the 10% gel cast using most-commonly-used mini-gel cast systems. This middle range still leaves us with a large region below 48kD and another region above 72kD. Therefore, those 48-72kD WT proteins, which are more commonly studied in published papers, could be well differentiated from their isoforms at a much lower or higher molecular weight. Third, the 26-40 kD range has already been studied in our previous paper [12]. Fourth, proteins at higher positions (say higher than 100kD) of a 10% gel cannot be well separated. In summary, these positions were selected after many technical issues were taken into consideration.

### LC-MS/MS

The procedures for the sample preparation and the ensuing LC-MS/MS analysis were routine and were described in detail previously [12], and were conducted at Beijing Institute of Genomics, Chinese Academy of Sciences, Beijing, P.R. China. Briefly, the excised gel stripes were dehydrated with increasing concentrations of acetonitrile (ACN). The in-gel proteins were reduced and alkylated with 10 mM dithiothreitol and 55 mM iodoacetamide and then digested with trypsin at 37 °C for 16 hours [32]. The tryptic peptides were extracted with ACN containing 0.1% formic acid (FA), dried using vacuum, and dissolved in 0.1% FA. The peptides were delivered onto a nano RP column (5-μm Hypersil C18, 75 mm × 100 mm; Thermo Fisher Scientific, Waltham, MA, USA) and eluted with increasing concentrations (50-80%) of ACN for 60 min at 400 nL/min. Different fractions of the eluate were injected into a Q-Exactive mass spectrometry (Thermo Fisher Scientific, Waltham, MA, USA) set in a positive ion mode and a data-dependent manner with a full MS scan from 350 to 2,000 m/z. High collision energy dissociation was used as the MS/MS acquisition method. Raw MS/MS data were converted to an MGF format with Proteome Discoverer 1.2 (Thermo Fisher Scientific, Waltham, MA, USA). The exported MGF files were searched with Mascot v2.3.01 in local server against the human SwissProt_new xyzzy database (20255 sequences). All searches were carried out with a tryptic specificity allowing one missed cleavage. Carbamidomethylation was regarded as a fixed modification whereas oxidation (M) and Gln->pyro-Glu (N-term Q) as variable modifications. The mass tolerance for MS and MS/MS was 15 ppm and 20 mmu, respectively. Proteins with false discovery rates < 0.01 were further analyzed.

### Statistical analyses

The methods used for statistical comparisons were indicated in the relevant tables, with p<0.05 considered statistically significant.

## CONCLUSIONS

In conclusion, only 34.4%, 35.9% and 22.2% LC-MS/MS-identified proteins from MB231 cells and 24.9%, 34.6% and 26.5% proteins from MCF7 cells from the 48kD, 55kD and 72kD stripes, respectively, belong to the WT category, with the remaining preponderance having a TMM larger or smaller than the WT range. About 24.6% and 22.7% of the proteins identified by LC-MS/MS from MCF7 and MB231 cells, respectively, or on average 23.7%, appeared in all three stripes, indicating that about one-fourth of the proteins may have multiple isoforms. About 3-16%, on average about 10% regardless of the stripes and cell lines, of the proteins appear solely in one stripe and within the WT range. Considering that we have only studied three stripes and those “solely in one stripe” may also appear in another unstudied position of the SDS-PAGE, probably few genes in the human genome produce only the WT protein without at least one additional isoform. This astonishing conclusion questions some common practices in biomedical research, including WB and immunohistochemical staining.

## SUPPLEMENTARY MATERIALS TABLES







## Abbreviations

AA: amino acid
ACN: acetonitrile
ACTP: approximate coverage of truncated proteins
CR: coverage rate
FA: formic acid
PLEC: plectin-isoform-1 gene
SDS-PAGE: sodium dodecyl sulfate-containing polyacrylamide gel
kD: kilo-Dalton
LC-MS/MS: liquid chromatography together with tandem mass spectrometry
WT: wild type
WB: western blotting
TMM: theoretical molecular mass

## References

[1] Kornblihtt AR, Schor IE, Allo M, Dujardin G, Petrillo E, Munoz MJ (2013). Alternative splicing: a pivotal step between eukaryotic transcription and translation. Nat Rev Mol Cell Biol.

[2] Jia Y, Chen L, Ma Y, Zhang J, Xu N, Liao DJ (2015). To know how a gene works, we need to redefine it first but then, more importantly, to let the cell itself decide how to transcribe and process its RNAs. Int J Biol Sci.

[3] Gorr TA, Vogel J (2015). Western blotting revisited: critical perusal of underappreciated technical issues. Proteomics Clin Appl.

[4] Liu X, Wang Y, Yang W, Guan Z, Yu W, Liao DJ (2016). Protein multiplicity can lead to misconduct in western blotting and misinterpretation of immunohistochemical staining results, creating much conflicting data. Prog Histochem Cytochem.

[5] Ahmad Y, Boisvert FM, Lundberg E, Uhlen M, Lamond AI (2012). Systematic analysis of protein pools, isoforms, and modifications affecting turnover and subcellular localization. Mol Cell Proteomics.

[6] Ezkurdia I, Vazquez J, Valencia A, Tress M (2014). Analyzing the first drafts of the human proteome. J Proteome Res.

[7] Kim MS, Pinto SM, Getnet D, Nirujogi RS, Manda SS, Chaerkady R, Madugundu AK, Kelkar DS, Isserlin R, Jain S, Thomas JK, Muthusamy B, Leal-Rojas P (2014). A draft map of the human proteome. Nature.

[8] Wilhelm M, Schlegl J, Hahne H, Moghaddas GA, Lieberenz M, Savitski MM, Ziegler E, Butzmann L, Gessulat S, Marx H, Mathieson T, Lemeer S, Schnatbaum K (2014). Mass-spectrometry-based draft of the human proteome. Nature.

[9] Ezkurdia I, Juan D, Rodriguez JM, Frankish A, Diekhans M, Harrow J, Vazquez J, Valencia A, Tress ML (2014). Multiple evidence strands suggest that there may be as few as 19,000 human protein-coding genes. Hum Mol Genet.

[10] Ezkurdia I, Calvo E, Del PA, Vazquez J, Valencia A, Tress ML (2015). The potential clinical impact of the release of two drafts of the human proteome. Expert Rev Proteomics.

[11] Toby TK, Fornelli L, Kelleher NL (2016). Progress in top-down proteomics and the analysis of proteoforms. Annu Rev Anal Chem (Palo Alto Calif).

[12] Zhang J, Lou X, Shen H, Zellmer L, Sun Y, Liu S, Xu N, Liao DJ (2014). Isoforms of wild type proteins often appear as low molecular weight bands on SDS-PAGE. Biotechnol J.

[13] Reddy PJ, Ray S, Srivastava S (2015). The quest of the human proteome and the missing proteins: digging deeper. OMICS.

[14] Yagoub D, Tay AP, Chen Z, Hamey JJ, Cai C, Chia SZ, Hart-Smith G, Wilkins MR (2015). Proteogenomic discovery of a small, novel protein in yeast reveals a strategy for the detection of unannotated short open reading frames. J Proteome Res.

[15] Olexiouk V, Crappe J, Verbruggen S, Verhegen K, Martens L, Menschaert G (2016). sORFs.org: a repository of small ORFs identified by ribosome profiling. Nucleic Acids Res.

[16] Nelson BR, Makarewich CA, Anderson DM, Winders BR, Troupes CD, Wu F, Reese AL, McAnally JR, Chen X, Kavalali ET, Cannon SC, Houser SR, Bassel-Duby R, Olson EN (2016). A peptide encoded by a transcript annotated as long noncoding RNA enhances SERCA activity in muscle. Science.

[17] Hertweck C (2011). Biosynthesis and charging of pyrrolysine, the 22nd genetically encoded amino acid. Angew Chem Int Ed Engl.

[18] Peng Z, Yuan C, Zellmer L, Liu S, Xu N, Liao DJ (2015). Hypothesis: artifacts, including spurious chimeric RNAs with a short homologous sequence, caused by consecutive reverse transcriptions and endogenous random primers. J Cancer.

[19] Yuan C, Han Y, Zellmer L, Yang W, Guan Z, Yu W, Huang H, Liao DJ (2017). It is imperative to establish a pellucid definition of chimeric RNA and to clear up a lot of confusion in the relevant research. Int J Mol Sci.

[20] Mohler K, Aerni HR, Gassaway B, Ling J, Ibba M, Rinehart J (2017). MS-READ: quantitative measurement of amino acid incorporation. Biochim Biophys Acta.

[21] Huang P, Chen C, Liu-Chen LY (2015). Detection of mu opioid receptor (MOPR) and its glycosylation in rat and mouse brains by western blot with anti-muC, an affinity-purified polyclonal anti-MOPR antibody. Methods Mol Biol.

[22] Ezkurdia I, Rodriguez JM, Carrillo-de Santa PE, Vazquez J, Valencia A, Tress ML (2015). Most highly expressed protein-coding genes have a single dominant isoform. J Proteome Res.

[23] Tucher J, Koudelka T, Schlenk J, Tholey A (2016). From top-down to bottom-up: time-dependent monitoring of proteolytic protein degradation by LC-MS. J Chromatogr B Analyt Technol Biomed Life Sci.

[24] Sarsby J, Martin NJ, Lalor PF, Bunch J, Cooper HJ (2014). Top-down and bottom-up identification of proteins by liquid extraction surface analysis mass spectrometry of healthy and diseased human liver tissue. J Am Soc Mass Spectrom.

[25] You Z, Dong Y, Kong X, Zhang Y, Vessella RL, Melamed J (2007). Differential expression of IL-17RC isoforms in androgen-dependent and androgen-independent prostate cancers. Neoplasia.

[26] Sun Y, Cao S, Yang M, Wu S, Wang Z, Lin X, Song X, Liao DJ (2013). Basic anatomy and tumor biology of the RPS6KA6 gene that encodes the p90 ribosomal S6 kinase-4. Oncogene.

[27] Sun Y, Lou X, Yang M, Yuan C, Ma L, Xie BK, Wu JM, Yang W, Shen SX, Xu N, Liao DJ (2013). Cyclin-dependent kinase 4 may be expressed as multiple proteins and have functions that are independent of binding to CCND and RB and occur at the S and G 2/M phases of the cell cycle. Cell Cycle.

[28] Yang M, Sun Y, Ma L, Wang C, Wu JM, Bi A, Liao DJ (2011). Complex alternative splicing of the smarca2 gene suggests the importance of smarca2-B variants. J Cancer.

[29] Yang M, Wu J, Wu SH, Bi AD, Liao DJ (2012). Splicing of mouse p53 pre-mRNA does not always follow the “first come, first served” principle and may be influenced by cisplatin treatment and serum starvation. Mol Biol Rep.

[30] Karakas C, Biernacka A, Bui T, Sahin AA, Yi M, Akli S, Schafer J, Alexander A, Adjapong O, Hunt KK, Keyomarsi K (2016). Cytoplasmic cyclin E and phospho-cyclin-dependent kinase 2 are biomarkers of aggressive breast cancer. Am J Pathol.

[31] Marcel V, Khoury MP, Fernandes K, Diot A, Lane DP, Bourdon JC (2013). Detecting p53 isoforms at protein level. Methods Mol Biol.

[32] Meng B, Qian Z, Wei F, Wang W, Zhou C, Wang Z, Wang Q, Tong W, Wang Q, Ma Y, Xu N, Liu S (2009). Proteomic analysis on the temperature-dependent complexes in Thermoanaerobacter tengcongensis. Proteomics.

