# Supplementary material for: Probably less than one-tenth of the genes produce only the wild type protein without at least one additional protein isoform in some human cancer cell lines
Source: Oncotarget. 2017 Aug 7;8(47):82714–27. doi: 10.18632/oncotarget.20015 (PMC5669923; doi:10.18632/oncotarget.20015)
Supplement: Supplementary file 1 [file oncotarget-08-82714-s001.pdf]

Probably less than one-tenth of the genes produce only the wild type protein without at least one additional protein isoform in some human cancer cell lines

SUPPLEMENTARY MATERIALS

Supplementary Data 1: original MS-identified proteins.

See Supplementary File 1

Supplementary Data 2: The 20 smallest proteins identified in the 48-kD stripe (MB231).

See Supplementary File 2
