# Supplementary material for: Probably less than one-tenth of the genes produce only the wild type protein without at least one additional protein isoform in some human cancer cell lines
Source: Oncotarget. 2017 Aug 7;8(47):82714–27. doi: 10.18632/oncotarget.20015 (PMC5669923; doi:10.18632/oncotarget.20015)
Supplement: Supplementary file 3 [file oncotarget-08-82714-s003.docx]

**Supplementary data 2:**

**The 20 smallest proteins identified in the 48-kD stripe (MB231)**

1) >gi|4507131|ref|NP_003086.1| small nuclear ribonucleoprotein F [Homo sapiens] (9.7 kD)

MSLPLNPKPFLNGLTGKPVMVKLKWGMEYKGYLVSVDGYMNMQLANTEEYIDGALSGHLGEVLIRCNNVLYIRGVEEEEEDGEMRE

2) >gi|4507129|ref|NP_003085.1| small nuclear ribonucleoprotein E [Homo sapiens] (10.8 kD)

MAYRGQGQKVQKVMVQPINLIFRYLQNRSRIQVWLYEQVNMRIEGCIIGFDEYMNLVLDDAEEIHSKTKSRKQLGRIMLKGDNITLLQSVSN

3) >gi|4504523|ref|NP_002148.1| 10 kDa heat shock protein, mitochondrial [Homo sapiens] (10.9 kD)

MAGQAFRKFLPLFDRVLVERSAAETVTKGGIMLPEKSQGKVLQATVVAVGSGSKGKGGEIQPVSVKVGDKVLLPEYGGTKVVLDDKDYFLFRDGDILGKYVD

4) >gi|4504301|ref|NP_003529.1| histone H4 [Homo sapiens] (11.4 kD)

MSGRGKGGKGLGKGGAKRHRKVLRDNIQGITKPAIRRLARRGGVKRISGLIYEETRGVLKVFLENVIRDAVTYTEHAKRKTVTAMDVVYALKRQGRTLYGFGG

5) >gi|11128019|ref|NP_061820.1| cytochrome c [Homo sapiens] (11.7 kD)

MGDVEKGKKIFIMKCSQCHTVEKGGKHKTGPNLHGLFGRKTGQAPGYSYTAANKNKGIIWGEDTLMEYLENPKKYIPGTKMIFVGIKKKEERADLIAYLKKATNE

6) >gi|4503253|ref|NP_001335.1| dolichyl-diphosphooligosaccharide--protein glycosyltransferase subunit DAD1 [Homo sapiens] (12.5 kD)

MSASVVSVISRFLEEYLSSTPQRLKLLDAYLLYILLTGALQFGYCLLVGTFPFNSFLSGFISCVGSFILAVCLRIQINPQNKADFQGISPERAFADFLFASTILHLVVMNFVG

7) >gi|18105048|ref|NP_542160.1| histone H2B type 1-K [Homo sapiens] (13.9 kD)

MPEPAKSAPAPKKGSKKAVTKAQKKDGKKRKRSRKESYSVYVYKVLKQVHPDTGISSKAMGIMNSFVNDIFERIAGEASRLAHYNKRSTITSREIQTAVRLLLPGELAKHAVSEGTKAVTKYTSAK

8) >gi|4504981|ref|NP_002296.1| galectin-1 [Homo sapiens] (14.7 kD)

MACGLVASNLNLKPGECLRVRGEVAPDAKSFVLNLGKDSNNLCLHFNPRFNAHGDANTIVCNSKDGGAWGTEQREAVFPFQPGSVAEVCITFDQANLTVKLPDGYEFKFPNRLNLEAINYMAADGDFKIKCVAFD

9) >gi|4826898|ref|NP_005013.1| profilin-1 [Homo sapiens] (15 kD)

MAGWNAYIDNLMADGTCQDAAIVGYKDSPSVWAAVPGKTFVNITPAEVGVLVGKDRSSFYVNGLTLGGQKCSVIRDSLLQDGEFSMDLRTKSTGGAPTFNVTVTKTDKTLVLLMGKEGVHGGLINKKCYEMASHLRRSQY

10) >gi|7705636|ref|NP_057156.1| vesicle transport protein GOT1B [Homo sapiens] (15.4 kD)

MISLTDTQKIGMGLTGFGVFFLFFGMILFFDKALLAIGNVLFVAGLAFVIGLERTFRFFFQKHKMKATGFFLGGVFVVLIGWPLIGMIFEIYGFFLLFRGFFPVVVGFIRRVPVLGSLLNLPGIRSFVDKVGESNNMV

11) >gi|4506623|ref|NP_000979.1| 60S ribosomal protein L27 [Homo sapiens] (15.8 kD)

MGKFMKPGKVVLVLAGRYSGRKAVIVKNIDDGTSDRPYSHALVAGIDRYPRKVTAAMGKKKIAKRSKIKSFVKVYNYNHLMPTRYSVDIPLDKTVVNKDVFRDPALKRKARREAKVKFEERYKTGKNKWFFQKLRF

12) >gi|4506701|ref|NP_001016.1| 40S ribosomal protein S23 [Homo sapiens] (15.8 kD)

MGKCRGLRTARKLRSHRRDQKWHDKQYKKAHLGTALKANPFGGASHAKGIVLEKVGVEAKQPNSAIRKCVRVQLIKNGKKITAFVPNDGCLNFIEENDEVLVAGFGRKGHAVGDIPGVRFKVVKVANVSLLALYKGKKERPRS

13) >gi|21624607|ref|NP_066972.1| coactosin-like protein [Homo sapiens] (15.9 kD)

MATKIDKEACRAAYNLVRDDGSAVIWVTFKYDGSTIVPGEQGAEYQHFIQQCTDDVRLFAFVRFTTGDAMSKRSKFALITWIGENVSGLQRAKTGTDKTLVKEVVQNFAKEFVISDRKELEEDFIKSELKKAGGANYDAQTE

14) >gi|4506691|ref|NP_001011.1| 40S ribosomal protein S16 [Homo sapiens] (16.4 kD)

MPSKGPLQSVQVFGRKKTATAVAHCKRGNGLIKVNGRPLEMIEPRTLQYKLLEPVLLLGKERFAGVDIRVRVKGGGHVAQIYAIRQSISKALVAYYQKYVDEASKKEIKDILIQYDRTLLVADPRRCESKKFGGPGARARYQKSYR

15) >gi|4758714|ref|NP_004519.1| microsomal glutathione S-transferase 3 [Homo sapiens] (16.5 kD)

MAVLSKEYGFVLLTGAASFIMVAHLAINVSKARKKYKVEYPIMYSTDPENGHIFNCIQRAHQNTLEVYPPFLFFLAVGGVYHPRIASGLGLAWIVGRVLYAYGYYTGEPSKRSRGALGSIALLGLVGTTVCSAFQHLGWVKSGLGSGPKCCH

16) >gi|4505893|ref|NP_002659.1| proteolipid protein 2 [Homo sapiens] (16.7 kD)

MADSERLSAPGCWAACTNFSRTRKGILLFAEIILCLVILICFSASTPGYSSLSVIEMILAAIFFVVYMCDLHTKIPFINWPWSDFFRTLIAAILYLITSIVVLVERGNHSKIVAGVLGLIATCLFGYDAYVTFPVRQPRHTAAPTDPADGPV

17) >gi|17986258|ref|NP_066299.2| myosin light polypeptide 6 isoform 1 [Homo sapiens] (16.9 kD)

MCDFTEDQTAEFKEAFQLFDRTGDGKILYSQCGDVMRALGQNPTNAEVLKVLGNPKSDEMNVKVLDFEHFLPMLQTVAKNKDQGTYEDYVEGLRVFDKEGNGTVMGAEIRHVLVTLGEKMTEEEVEMLVAGHEDSNGCINYEAFVRHILSG

18) >gi|11968182|ref|NP_072045.1| 40S ribosomal protein S18 [Homo sapiens] (17.7 kD)

MSLVIPEKFQHILRVLNTNIDGRRKIAFAITAIKGVGRRYAHVVLRKADIDLTKRAGELTEDEVERVITIMQNPRQYKIPDWFLNRQKDVKDGKYSQVLANGLDNKLREDLERLKKIRAHRGLRHFWGLRVRGQHTKTTGRRGRTVGVSKKK

19) >gi|10863927|ref|NP_066953.1| peptidyl-prolyl cis-trans isomerase A [Homo sapiens] (18 kD)

MVNPTVFFDIAVDGEPLGRVSFELFADKVPKTAENFRALSTGEKGFGYKGSCFHRIIPGFMCQGGDFTRHNGTGGKSIYGEKFEDENFILKHTGPGILSMANAGPNTNGSQFFICTAKTEWLDGKHVVFGKVKEGMNIVEAMERFGSRNGKTSKKITIADCGQLE

20) >gi|5031635|ref|NP_005498.1| cofilin-1 [Homo sapiens] (18.5 kD)

MASGVAVSDGVIKVFNDMKVRKSSTPEEVKKRKKAVLFCLSEDKKNIILEEGKEILVGDVGQTVDDPYATFVKMLPDKDCRYALYDATYETKESKKEDLVFIFWAPESAPLKSKMIYASSKDAIKKKLTGIKHELQANCYEEVKDRCTLAEKLGGSAVISLEGKPL

**Figure legend**: These are 20 smallest proteins identified in the 48-kD stripe (MB231). The red underlined sequences are the LC-MS/MS identified peptide fragments that are unique to the protein, while the green underlined sequences are the LC-MS/MS identified peptide fragments that are not unique to the protein but can also appear in other proteins.

**The 40 largest proteins in the 48-kD stripe (MB231)**

1) >gi|61743954|ref|NP_001611.1| neuroblast differentiation-associated protein AHNAK isoform 1 [Homo sapiens] (628.7 kD)

MEKEETTRELLLPNWQGSGSHGLTIAQRDDGVFVQEVTQNSPAARTGVVKEGDQIVGATIYFDNLQSGEVTQLLNTMGHHTVGLKLHRKGDRSPEPGQTWTREVFSSCSSEVVLSGDDEEYQRIYTTKIKPRLKSEDGVEGDLGETQSRTITVTRRVTAYTVDVTGREGAKDIDISSPEFKIKIPRHELTEISNVDVETQSGKTVIRLPSGSGAASPTGSAVDIRAGAISASGPELQGAGHSKLQVTMPGIKVGGSGVNVNAKGLDLGGRGGVQVPAVDISSSLGGRAVEVQGPSLESGDHGKIKFPTMKVPKFGVSTGREGQTPKAGLRVSAPEVSVGHKGGKPGLTIQAPQLEVSVPSANIEGLEGKLKGPQITGPSLEGDLGLKGAKPQGHIGVDASAPQIGGSITGPSVEVQAPDIDVQGPGSKLNVPKMKVPKFSVSGAKGEETGIDVTLPTGEVTVPGVSGDVSLPEIATGGLEGKMKGTKVKTPEMIIQKPKISMQDVDLSLGSPKLKGDIKVSAPGVQGDVKGPQVALKGSRVDIETPNLEGTLTGPRLGSPSGKTGTCRISMSEVDLNVAAPKVKGGVDVTLPRVEGKVKVPEVDVRGPKVDVSAPDVEAHGPEWNLKMPKMKMPTFSTPGAKGEGPDVHMTLPKGDISISGPKVNVEAPDVNLEGLGGKLKGPDVKLPDMSVKTPKISMPDVDLHVKGTKVKGEYDVTVPKLEGELKGPKVDIDAPDVDVHGPDWHLKMPKMKMPKFSVPGFKAEGPEVDVNLPKADVDISGPKIDVTAPDVSIEEPEGKLKGPKFKMPEMNIKVPKISMPDVDLHLKGPNVKGEYDVTMPKVESEIKVPDVELKSAKMDIDVPDVEVQGPDWHLKMPKMKMPKFSMPGFKAEGPEVDVNLPKADVDISGPKVGVEVPDVNIEGPEGKLKGPKFKMPEMNIKAPKISMPDVDLHMKGPKVKGEYDMTVPKLEGDLKGPKVDVSAPDVEMQGPDWNLKMPKIKMPKFSMPSLKGEGPEFDVNLSKANVDISAPKVDTNAPDLSLEGPEGKLKGPKFKMPEMHFRAPKMSLPDVDLDLKGPKMKGNVDISAPKIEGEMQVPDVDIRGPKVDIKAPDVEGQGLDWSLKIPKMKMPKFSMPSLKGEGPEVDVNLPKADVVVSGPKVDIEAPDVSLEGPEGKLKGPKFKMPEMHFKTPKISMPDVDLHLKGPKVKGDVDVSVPKVEGEMKVPDVEIKGPKMDIDAPDVEVQGPDWHLKMPKMKMPKFSMPGFKGEGREVDVNLPKADIDVSGPKVDVEVPDVSLEGPEGKLKGPKFKMPEMHFKAPKISMPDVDLNLKGPKLKGDVDVSLPEVEGEMKVPDVDIKGPKVDISAPDVDVHGPDWHLKMPKVKMPKFSMPGFKGEGPEVDVKLPKADVDVSGPKMDAEVPDVNIEGPDAKLKGPKFKMPEMSIKPQKISIPDVGLHLKGPKMKGDYDVTVPKVEGEIKAPDVDIKGPKVDINAPDVEVHGPDWHLKMPKVKMPKFSMPGFKGEGPEVDMNLPKADLGVSGPKVDIDVPDVNLEAPEGKLKGPKFKMPSMNIQTHKISMPDVGLNLKAPKLKTDVDVSLPKVEGDLKGPEIDVKAPKMDVNVGDIDIEGPEGKLKGPKFKMPEMHFKAPKISMPDVDLHLKGPKVKGDMDVSVPKVEGEMKVPDVDIKGPKVDIDAPDVEVHDPDWHLKMPKMKMPKFSMPGFKAEGPEVDVNLPKADIDVSGPSVDTDAPDLDIEGPEGKLKGSKFKMPKLNIKAPKVSMPDVDLNLKGPKLKGEIDASVPELEGDLRGPQVDVKGPFVEAEVPDVDLECPDAKLKGPKFKMPEMHFKAPKISMPDVDLHLKGPKVKGDADVSVPKLEGDLTGPSVGVEVPDVELECPDAKLKGPKFKMPDMHFKAPKISMPDVDLHLKGPKVKGDVDVSVPKLEGDLTGPSVGVEVPDVELECPDAKLKGPKFKMPEMHFKTPKISMPDVDLHLKGPKVKGDMDVSVPKVEGEMKVPDVDIKGPKMDIDAPDVDVHGPDWHLKMPKMKMPKFSMPGFKAEGPEVDVNLPKADVVVSGPKVDVEVPDVSLEGPEGKLKGPKLKMPEMHFKAPKISMPDVDLHLKGPKVKGDVDVSLPKLEGDLTGPSVDVEVPDVELECPDAKLKGPKFKMPEMHFKTPKISMPDVNLNLKGPKVKGDMDVSVPKVEGEMKVPDVDIRGPKVDIDAPDVDVHGPDWHLKMPKMKMPKFSMPGFKGEGPEVDVNLPKADVDVSGPKVDVEVPDVSLEGPEGKLKGPKFKMPEMHFKTPKISMPDVDFNLKGPKIKGDVDVSAPKLEGELKGPELDVKGPKLDADMPEVAVEGPNGKWKTPKFKMPDMHFKAPKISMPDLDLHLKSPKAKGEVDVDVPKLEGDLKGPHVDVSGPDIDIEGPEGKLKGPKFKMPDMHFKAPNISMPDVDLNLKGPKIKGDVDVSVPEVEGKLEVPDMNIRGPKVDVNAPDVQAPDWHLKMPKMKMPKFSMPGFKAEGPEVDVNLPKADVDISGPKVDIEGPDVNIEGPEGKLKGPKLKMPEMNIKAPKISMPDFDLHLKGPKVKGDVDVSLPKVEGDLKGPEVDIKGPKVDINAPDVGVQGPDWHLKMPKVKMPKFSMPGFKGEGPDGDVKLPKADIDVSGPKVDIEGPDVNIEGPEGKLKGPKFKMPEMNIKAPKISMPDIDLNLKGPKVKGDVDVSLPKVEGDLKGPEVDIKGPKVDIDAPDVDVHGPDWHLKMPKIKMPKISMPGFKGEGPDVDVNLPKADIDVSGPKVDVECPDVNIEGPEGKWKSPKFKMPEMHFKTPKISMPDIDLNLTGPKIKGDVDVTGPKVEGDLKGPEVDLKGPKVDIDVPDVNVQGPDWHLKMPKMKMPKFSMPGFKAEGPEVDVNLPKADVDVSGPKVDVEGPDVNIEGPEGKLKGPKFKMPEMNIKAPKIPMPDFDLHLKGPKVKGDVDISLPKVEGDLKGPEVDIRGPQVDIDVPDVGVQGPDWHLKMPKVKMPKFSMPGFKGEGPDVDVNLPKADLDVSGPKVDIDVPDVNIEGPEGKLKGPKFKMPEMNIKAPKISMPDIDLNLKGPKVKGDMDVSLPKVEGDMKVPDVDIKGPKVDINAPDVDVQGPDWHLKMPKIKMPKISMPGFKGEGPEVDVNLPKADLDVSGPKVDVDVPDVNIEGPDAKLKGPKFKMPEMNIKAPKISMPDLDLNLKGPKMKGEVDVSLANVEGDLKGPALDIKGPKIDVDAPDIDIHGPDAKLKGPKLKMPDMHVNMPKISMPEIDLNLKGSKLKGDVDVSGPKLEGDIKAPSLDIKGPEVDVSGPKLNIEGKSKKSRFKLPKFNFSGSKVQTPEVDVKGKKPDIDITGPKVDINAPDVEVQGKVKGSKFKMPFLSISSPKVSMPDVELNLKSPKVKGDLDIAGPNLEGDFKGPKVDIKAPEVNLNAPDVDVHGPDWNLKMPKMKMPKFSVSGLKAEGPDVAVDLPKGDINIEGPSMNIEGPDLNVEGPEGGLKGPKFKMPDMNIKAPKISMPDIDLNLKGPKVKGDVDISLPKLEGDLKGPEVDIKGPKVDINAPDVDVHGPDWHLKMPKVKMPKFSMPGFKGEGPEVDVTLPKADIDISGPNVDVDVPDVNIEGPDAKLKGPKFKMPEMNIKAPKISMPDFDLNLKGPKMKGDVVVSLPKVEGDLKGPEVDIKGPKVDIDTPDINIEGSEGKFKGPKFKIPEMHLKAPKISMPDIDLNLKGPKVKGDVDVSLPKMEGDLKGPEVDIKGPKVDINAPDVDVQGPDWHLKMPKVKMPKFSMPGFKGEGPDVDVNLPKADLDVSGPKVDIDVPDVNIEGPEGKLKGPKFKMPEMNIKAPKISMPDIDLNLKGPKVKGDMDVSLPKVEGDMQVPDLDIKGPKVDINAPDVDVRGPDWHLKMPKIKMPKISMPGFKGEGPEVDVNLPKADLDVSGPKVDVDVPDVNIEGPDAKLKGPKFKMPEMNIKAPKISMPDFDLHLKGPKVKGDVDVSLPKMEGDLKAPEVDIKGPKVDIDAPDVDVHGPDWHLKMPKVKMPKFSMPGFKGEGPEVDVNLPKADIDVSGPKVDIDTPDIDIHGPEGKLKGPKFKMPDLHLKAPKISMPEVDLNLKGPKMKGDVDVSLPKVEGDLKGPEVDIKGPKVDIDVPDVDVQGPDWHLKMPKVKMPKFSMPGFKGEGPDVDVNLPKADLDVSGPKVDIDVPDVNIEGPDAKLKGPKFKMPEMNIKAPKISMPDFDLHLKGPKVKGDVDVSLPKVEGDLKGPEVDIKGPKVDIDAPDVDVHGPDWHLKMPKVKMPKFSMPGFKGEGPDVDVTLPKADIEISGPKVDIDAPDVSIEGPDAKLKGPKFKMPEMNIKAPKISMPDIDFNLKGPKVKGDVDVSLPKVEGDLKGPEIDIKGPSLDIDTPDVNIEGPEGKLKGPKFKMPEMNIKAPKISMPDFDLHLKGPKVKGDVDVSLPKVESDLKGPEVDIEGPEGKLKGPKFKMPDVHFKSPQISMSDIDLNLKGPKIKGDMDISVPKLEGDLKGPKVDVKGPKVGIDTPDIDIHGPEGKLKGPKFKMPDLHLKAPKISMPEVDLNLKGPKVKGDMDISLPKVEGDLKGPEVDIRDPKVDIDVPDVDVQGPDWHLKMPKVKMPKFSMPGFKGEGPDVDVNLPKADIDVSGPKVDVDVPDVNIEGPDAKLKGPKFKMPEMSIKAPKISMPDIDLNLKGPKVKGDVDVTLPKVEGDLKGPEADIKGPKVDINTPDVDVHGPDWHLKMPKVKMPKFSMPGFKGEGPDVDVSLPKADIDVSGPKVDVDIPDVNIEGPDAKLKGPKFKMPEINIKAPKISIPDVDLDLKGPKVKGDFDVSVPKVEGTLKGPEVDLKGPRLDFEGPDAKLSGPSLKMPSLEISAPKVTAPDVDLHLKAPKIGFSGPKLEGGEVDLKGPKVEAPSLDVHMDSPDINIEGPDVKIPKFKKPKFGFGAKSPKADIKSPSLDVTVPEAELNLETPEISVGGKGKKSKFKMPKIHMSGPKIKAKKQGFDLNVPGGEIDASLKAPDVDVNIAGPDAALKVDVKSPKTKKTMFGKMYFPDVEFDIKSPKFKAEAPLPSPKLEGELQAPDLELSLPAIHVEGLDIKAKAPKVKMPDVDISVPKIEGDLKGPKVQANLGAPDINIEGLDAKVKTPSFGISAPQVSIPDVNVNLKGPKIKGDVPSVGLEGPDVDLQGPEAKIKFPKFSMPKIGIPGVKMEGGGAEVHAQLPSLEGDLRGPDVKLEGPDVSLKGPGVDLPSVNLSMPKVSGPDLDLNLKGPSLKGDLDASVPSMKVHAPGLNLSGVGGKMQVGGDGVKVPGIDATTKLNVGAPDVTLRGPSLQGDLAVSGDIKCPKVSVGAPDLSLEASEGSIKLPKMKLPQFGISTPGSDLHVNAKGPQVSGELKGPGVDVNLKGPRISAPNVDFNLEGPKVKGSLGATGEIKGPTVGGGLPGIGVQGLEGNLQMPGIKSSGCDVNLPGVNVKLPTGQISGPEIKGGLKGSEVGFHGAAPDISVKGPAFNMASPESDFGINLKGPKIKGGADVSGGVSAPDISLGEGHLSVKGSGGEWKGPQVSSALNLDTSKFAGGLHFSGPKVEGGVKGGQIGLQAPGLSVSGPQGHLESGSGKVTFPKMKIPKFTFSGRELVGREMGVDVHFPKAEASIQAGAGDGEWEESEVKLKKSKIKMPKFNFSKPKGKGGVTGSPEASISGSKGDLKSSKASLGSLEGEAEAEASSPKGKFSLFKSKKPRHRSNSFSDEREFSGPSTPTGTLEFEGGEVSLEGGKVKGKHGKLKFGTFGGLGSKSKGHYEVTGSDDETGKLQGSGVSLASKKSRLSSSSSNDSGNKVGIQLPEVELSVSTKKE

2) >gi|41322916|ref|NP_958782.1| plectin 1 isoform 1 [Homo sapiens] (531.5 kD)

MVAGMLMPRDQLRAIYEVLFREGVMVAKKDRRPRSLHPHVPGVTNLQVMRAMASLRARGLVRETFAWCHFYWYLTNEGIAHLRQYLHLPPEIVPASLQRVRRPVAMVMPARRTPHVQAVQGPLGSPPKRGPLPTEEQRVYRRKELEEVSPETPVVPATTQRTLARPGPEPAPATDERDRVQKKTFTKWVNKHLIKAQRHISDLYEDLRDGHNLISLLEVLSGDSLPREKGRMRFHKLQNVQIALDYLRHRQVKLVNIRNDDIADGNPKLTLGLIWTIILHFQISDIQVSGQSEDMTAKEKLLLWSQRMVEGYQGLRCDNFTSSWRDGRLFNAIIHRHKPLLIDMNKVYRQTNLENLDQAFSVAERDLGVTRLLDPEDVDVPQPDEKSIITYVSSLYDAMPRVPDVQDGVRANELQLRWQEYRELVLLLLQWMRHHTAAFEERRFPSSFEEIEILWSQFLKFKEMELPAKEADKNRSKGIYQSLEGAVQAGQLKVPPGYHPLDVEKEWGKLHVAILEREKQLRSEFERLECLQRIVTKLQMEAGLCEEQLNQADALLQSDVRLLAAGKVPQRAGEVERDLDKADSMIRLLFNDVQTLKDGRHPQGEQMYRRVYRLHERLVAIRTEYNLRLKAGVAAPATQVAQVTLQSVQRRPELEDSTLRYLQDLLAWVEENQHRVDGAEWGVDLPSVEAQLGSHRGLHQSIEEFRAKIERARSDEGQLSPATRGAYRDCLGRLDLQYAKLLNSSKARLRSLESLHSFVAAATKELMWLNEKEEEEVGFDWSDRNTNMTAKKESYSALMRELELKEKKIKELQNAGDRLLREDHPARPTVESFQAALQTQWSWMLQLCCCIEAHLKENAAYFQFFSDVREAEGQLQKLQEALRRKYSCDRSATVTRLEDLLQDAQDEKEQLNEYKGHLSGLAKRAKAVVQLKPRHPAHPMRGRLPLLAVCDYKQVEVTVHKGDECQLVGPAQPSHWKVLSSSGSEAAVPSVCFLVPPPNQEAQEAVTRLEAQHQALVTLWHQLHVDMKSLLAWQSLRRDVQLIRSWSLATFRTLKPEEQRQALHSLELHYQAFLRDSQDAGGFGPEDRLMAEREYGSCSHHYQQLLQSLEQGAQEESRCQRCISELKDIRLQLEACETRTVHRLRLPLDKEPARECAQRIAEQQKAQAEVEGLGKGVARLSAEAEKVLALPEPSPAAPTLRSELELTLGKLEQVRSLSAIYLEKLKTISLVIRGTQGAEEVLRAHEEQLKEAQAVPATLPELEATKASLKKLRAQAEAQQPTFDALRDELRGAQEVGERLQQRHGERDVEVERWRERVAQLLERWQAVLAQTDVRQRELEQLGRQLRYYRESADPLGAWLQDARRRQEQIQAMPLADSQAVREQLRQEQALLEEIERHGEKVEECQRFAKQYINAIKDYELQLVTYKAQLEPVASPAKKPKVQSGSESVIQEYVDLRTHYSELTTLTSQYIKFISETLRRMEEEERLAEQQRAEERERLAEVEAALEKQRQLAEAHAQAKAQAEREAKELQQRMQEEVVRREEAAVDAQQQKRSIQEELQQLRQSSEAEIQAKARQAEAAERSRLRIEEEIRVVRLQLEATERQRGGAEGELQALRARAEEAEAQKRQAQEEAERLRRQVQDESQRKRQAEVELASRVKAEAEAAREKQRALQALEELRLQAEEAERRLRQAEVERARQVQVALETAQRSAEAELQSKRASFAEKTAQLERSLQEEHVAVAQLREEAERRAQQQAEAERAREEAERELERWQLKANEALRLRLQAEEVAQQKSLAQAEAEKQKEEAEREARRRGKAEEQAVRQRELAEQELEKQRQLAEGTAQQRLAAEQELIRLRAETEQGEQQRQLLEEELARLQREAAAATQKRQELEAELAKVRAEMEVLLASKARAEEESRSTSEKSKQRLEAEAGRFRELAEEAARLRALAEEAKRQRQLAEEDAARQRAEAERVLAEKLAAIGEATRLKTEAEIALKEKEAENERLRRLAEDEAFQRRRLEEQAAQHKADIEERLAQLRKASDSELERQKGLVEDTLRQRRQVEEEILALKASFEKAAAGKAELELELGRIRSNAEDTLRSKEQAELEAARQRQLAAEEERRRREAEERVQKSLAAEEEAARQRKAALEEVERLKAKVEEARRLRERAEQESARQLQLAQEAAQKRLQAEEKAHAFAVQQKEQELQQTLQQEQSVLDQLRGEAEAARRAAEEAEEARVQAEREAAQSRRQVEEAERLKQSAEEQAQARAQAQAAAEKLRKEAEQEAARRAQAEQAALRQKQAADAEMEKHKKFAEQTLRQKAQVEQELTTLRLQLEETDHQKNLLDEELQRLKAEATEAARQRSQVEEELFSVRVQMEELSKLKARIEAENRALILRDKDNTQRFLQEEAEKMKQVAEEAARLSVAAQEAARLRQLAEEDLAQQRALAEKMLKEKMQAVQEATRLKAEAELLQQQKELAQEQARRLQEDKEQMAQQLAEETQGFQRTLEAERQRQLEMSAEAERLKLRVAEMSRAQARAEEDAQRFRKQAEEIGEKLHRTELATQEKVTLVQTLEIQRQQSDHDAERLREAIAELEREKEKLQQEAKLLQLKSEEMQTVQQEQLLQETQALQQSFLSEKDSLLQRERFIEQEKAKLEQLFQDEVAKAQQLREEQQRQQQQMEQERQRLVASMEEARRRQHEAEEGVRRKQEELQQLEQQRRQQEELLAEENQRLREQLQLLEEQHRAALAHSEEVTASQVAATKTLPNGRDALDGPAAEAEPEHSFDGLRRKVSAQRLQEAGILSAEELQRLAQGHTTVDELARREDVRHYLQGRSSIAGLLLKATNEKLSVYAALQRQLLSPGTALILLEAQAASGFLLDPVRNRRLTVNEAVKEGVVGPELHHKLLSAERAVTGYKDPYTGQQISLFQAMQKGLIVREHGIRLLEAQIATGGVIDPVHSHRVPVDVAYRRGYFDEEMNRVLADPSDDTKGFFDPNTHENLTYLQLLERCVEDPETGLCLLPLTDKAAKGGELVYTDSEARDVFEKATVSAPFGKFQGKTVTIWEIINSEYFTAEQRRDLLRQFRTGRITVEKIIKIIITVVEEQEQKGRLCFEGLRSLVPAAELLESRVIDRELYQQLQRGERSVRDVAEVDTVRRALRGANVIAGVWLEEAGQKLSIYNALKKDLLPSDMAVALLEAQAGTGHIIDPATSARLTVDEAVRAGLVGPEFHEKLLSAEKAVTGYRDPYTGQSVSLFQALKKGLIPREQGLRLLDAQLSTGGIVDPSKSHRVPLDVACARGCLDEETSRALSAPRADAKAYSDPSTGEPATYGELQQRCRPDQLTGLSLLPLSEKAARARQEELYSELQARETFEKTPVEVPVGGFKGRTVTVWELISSEYFTAEQRQELLRQFRTGKVTVEKVIKILITIVEEVETLRQERLSFSGLRAPVPASELLASGVLSRAQFEQLKDGKTTVKDLSELGSVRTLLQGSGCLAGIYLEDTKEKVSIYEAMRRGLLRATTAALLLEAQAATGFLVDPVRNQRLYVHEAVKAGVVGPELHEQLLSAEKAVTGYRDPYSGSTISLFQAMQKGLVLRQHGIRLLEAQIATGGIIDPVHSHRVPVDVAYQRGYFSEEMNRVLADPSDDTKGFFDPNTHENLTYRQLLERCVEDPETGLRLLPLKGAEKAEVVETTQVYTEEETRRAFEETQIDIPGGGSHGGSTMSLWEVMQSDLIPEEQRAQLMADFQAGRVTKERMIIIIIEIIEKTEIIRQQGLASYDYVRRRLTAEDLFEARIISLETYNLLREGTRSLREALEAESAWCYLYGTGSVAGVYLPGSRQTLSIYQALKKGLLSAEVARLLLEAQAATGFLLDPVKGERLTVDEAVRKGLVGPELHDRLLSAERAVTGYRDPYTEQTISLFQAMKKELIPTEEALRLLDAQLATGGIVDPRLGFHLPLEVAYQRGYLNKDTHDQLSEPSEVRSYVDPSTDERLSYTQLLRRCRRDDGTGQLLLPLSDARKLTFRGLRKQITMEELVRSQVMDEATALQLREGLTSIEEVTKNLQKFLEGTSCIAGVFVDATKERLSVYQAMKKGIIRPGTAFELLEAQAATGYVIDPIKGLKLTVEEAVRMGIVGPEFKDKLLSAERAVTGYKDPYSGKLISLFQAMKKGLILKDHGIRLLEAQIATGGIIDPEESHRLPVEVAYKRGLFDEEMNEILTDPSDDTKGFFDPNTEENLTYLQLMERCITDPQTGLCLLPLKEKKRERKTSSKSSVRKRRVVIVDPETGKEMSVYEAYRKGLIDHQTYLELSEQECEWEEITISSSDGVVKSMIIDRRSGRQYDIDDAIAKNLIDRSALDQYRAGTLSITEFADMLSGNAGGFRSRSSSVGSSSSYPISPAVSRTQLASWSDPTEETGPVAGILDTETLEKVSITEAMHRNLVDNITGQRLLEAQACTGGIIDPSTGERFPVTDAVNKGLVDKIMVDRINLAQKAFCGFEDPRTKTKMSAAQALKKGWLYYEAGQRFLEVQYLTGGLIEPDTPGRVPLDEALQRGTVDARTAQKLRDVGAYSKYLTCPKTKLKISYKDALDRSMVEEGTGLRLLEAAAQSTKGYYSPYSVSGSGSTAGSRTGSRTGSRAGSRRGSFDATGSGFSMTFSSSSYSSSGYGRRYASGSSASLGGPESAVA

3) >gi|71061468|ref|NP_001804.2| centromere-associated protein E isoform 1 [Homo sapiens] (316.2 kD)

MAEEGAVAVCVRVRPLNSREESLGETAQVYWKTDNNVIYQVDGSKSFNFDRVFHGNETTKNVYEEIAAPIIDSAIQGYNGTIFAYGQTASGKTYTMMGSEDHLGVIPRAIHDIFQKIKKFPDREFLLRVSYMEIYNETITDLLCGTQKMKPLIIREDVNRNVYVADLTEEVVYTSEMALKWITKGEKSRHYGETKMNQRSSRSHTIFRMILESREKGEPSNCEGSVKVSHLNLVDLAGSERAAQTGAAGVRLKEGCNINRSLFILGQVIKKLSDGQVGGFINYRDSKLTRILQNSLGGNAKTRIICTITPVSFDETLTALQFASTAKYMKNTPYVNEVSTDEALLKRYRKEIMDLKKQLEEVSLETRAQAMEKDQLAQLLEEKDLLQKVQNEKIENLTRMLVTSSSLTLQQELKAKRKRRVTWCLGKINKMKNSNYADQFNIPTNITTKTHKLSINLLREIDESVCSESDVFSNTLDTLSEIEWNPATKLLNQENIESELNSLRADYDNLVLDYEQLRTEKEEMELKLKEKNDLDEFEALERKTKKDQEMQLIHEISNLKNLVKHAEVYNQDLENELSSKVELLREKEDQIKKLQEYIDSQKLENIKMDLSYSLESIEDPKQMKQTLFDAETVALDAKRESAFLRSENLELKEKMKELATTYKQMENDIQLYQSQLEAKKKMQVDLEKELQSAFNEITKLTSLIDGKVPKDLLCNLELEGKITDLQKELNKEVEENEALREEVILLSELKSLPSEVERLRKEIQDKSEELHIITSEKDKLFSEVVHKESRVQGLLEEIGKTKDDLATTQSNYKSTDQEFQNFKTLHMDFEQKYKMVLEENERMNQEIVNLSKEAQKFDSSLGALKTELSYKTQELQEKTREVQERLNEMEQLKEQLENRDSTLQTVEREKTLITEKLQQTLEEVKTLTQEKDDLKQLQESLQIERDQLKSDIHDTVNMNIDTQEQLRNALESLKQHQETINTLKSKISEEVSRNLHMEENTGETKDEFQQKMVGIDKKQDLEAKNTQTLTADVKDNEIIEQQRKIFSLIQEKNELQQMLESVIAEKEQLKTDLKENIEMTIENQEELRLLGDELKKQQEIVAQEKNHAIKKEGELSRTCDRLAEVEEKLKEKSQQLQEKQQQLLNVQEEMSEMQKKINEIENLKNELKNKELTLEHMETERLELAQKLNENYEEVKSITKERKVLKELQKSFETERDHLRGYIREIEATGLQTKEELKIAHIHLKEHQETIDELRRSVSEKTAQIINTQDLEKSHTKLQEEIPVLHEEQELLPNVKEVSETQETMNELELLTEQSTTKDSTTLARIEMERLRLNEKFQESQEEIKSLTKERDNLKTIKEALEVKHDQLKEHIRETLAKIQESQSKQEQSLNMKEKDNETTKIVSEMEQFKPKDSALLRIEIEMLGLSKRLQESHDEMKSVAKEKDDLQRLQEVLQSESDQLKENIKEIVAKHLETEEELKVAHCCLKEQEETINELRVNLSEKETEISTIQKQLEAINDKLQNKIQEIYEKEEQFNIKQISEVQEKVNELKQFKEHRKAKDSALQSIESKMLELTNRLQESQEEIQIMIKEKEEMKRVQEALQIERDQLKENTKEIVAKMKESQEKEYQFLKMTAVNETQEKMCEIEHLKEQFETQKLNLENIETENIRLTQILHENLEEMRSVTKERDDLRSVEETLKVERDQLKENLRETITRDLEKQEELKIVHMHLKEHQETIDKLRGIVSEKTNEISNMQKDLEHSNDALKAQDLKIQEELRIAHMHLKEQQETIDKLRGIVSEKTDKLSNMQKDLENSNAKLQEKIQELKANEHQLITLKKDVNETQKKVSEMEQLKKQIKDQSLTLSKLEIENLNLAQKLHENLEEMKSVMKERDNLRRVEETLKLERDQLKESLQETKARDLEIQQELKTARMLSKEHKETVDKLREKISEKTIQISDIQKDLDKSKDELQKKIQELQKKELQLLRVKEDVNMSHKKINEMEQLKKQFEAQNLSMQSVRMDNFQLTKKLHESLEEIRIVAKERDELRRIKESLKMERDQFIATLREMIARDRQNHQVKPEKRLLSDGQQHLTESLREKCSRIKELLKRYSEMDDHYECLNRLSLDLEKEIEFQKELSMRVKANLSLPYLQTKHIEKLFTANQRCSMEFHRIMKKLKYVLSYVTKIKEEQHESINKFEMDFIDEVEKQKELLIKIQHLQQDCDVPSRELRDLKLNQNMDLHIEEILKDFSESEFPSIKTEFQQVLSNRKEMTQFLEEWLNTRFDIEKLKNGIQKENDRICQVNNFFNNRIIAIMNESTEFEERSATISKEWEQDLKSLKEKNEKLFKNYQTLKTSLASGAQVNPTTQDNKNPHVTSRATQLTTEKIRELENSLHEAKESAMHKESKIIKMQKELEVTNDIIAKLQAKVHESNKCLEKTKETIQVLQDKVALGAKPYKEEIEDLKMKLVKIDLEKMKNAKEFEKEISATKATVEYQKEVIRLLRENLRRSQQAQDTSVISEHTDPQPSNKPLTCGGGSGIVQNTKALILKSEHIRLEKEISKLKQQNEQLIKQKNELLSNNQHLSNEVKTWKERTLKREAHKQVTCENSPKSPKVTGTASKKKQITPSQCKERNLQDPVPKESPKSCFFDSRSKSLPSPHPVRYFDNSSLGLCPEVQNAGAESVDSQPGPWHASSGKDVPECKTQ

4) >gi|105990514|ref|NP_001448.2| filamin-B isoform 2 [Homo sapiens] (278 kD)

MPVTEKDLAEDAPWKKIQQNTFTRWCNEHLKCVNKRIGNLQTDLSDGLRLIALLEVLSQKRMYRKYHQRPTFRQMQLENVSVALEFLDRESIKLVSIDSKAIVDGNLKLILGLVWTLILHYSISMPVWEDEGDDDAKKQTPKQRLLGWIQNKIPYLPITNFNQNWQDGKALGALVDSCAPGLCPDWESWDPQKPVDNAREAMQQADDWLGVPQVITPEEIIHPDVDEHSVMTYLSQFPKAKLKPGAPLKPKLNPKKARAYGRGIEPTGNMVKQPAKFTVDTISAGQGDVMVFVEDPEGNKEEAQVTPDSDKNKTYSVEYLPKVTGLHKVTVLFAGQHISKSPFEVSVDKAQGDASKVTAKGPGLEAVGNIANKPTYFDIYTAGAGVGDIGVEVEDPQGKNTVELLVEDKGNQVYRCVYKPMQPGPHVVKIFFAGDTIPKSPFVVQVGEACNPNACRASGRGLQPKGVRIRETTDFKVDTKAAGSGELGVTMKGPKGLEELVKQKDFLDGVYAFEYYPSTPGRYSIAITWGGHHIPKSPFEVQVGPEAGMQKVRAWGPGLHGGIVGRSADFVVESIGSEVGSLGFAIEGPSQAKIEYNDQNDGSCDVKYWPKEPGEYAVHIMCDDEDIKDSPYMAFIHPATGGYNPDLVRAYGPGLEKSGCIVNNLAEFTVDPKDAGKAPLKIFAQDGEGQRIDIQMKNRMDGTYACSYTPVKAIKHTIAVVWGGVNIPHSPYRVNIGQGSHPQKVKVFGPGVERSGLKANEPTHFTVDCTEAGEGDVSVGIKCDARVLSEDEEDVDFDIIHNANDTFTVKYVPPAAGRYTIKVLFASQEIPASPFRVKVDPSHDASKVKAEGPGLSKAGVENGKPTHFTVYTKGAGKAPLNVQFNSPLPGDAVKDLDIIDNYDYSHTVKYTPTQQGNMQVLVTYGGDPIPKSPFTVGVAAPLDLSKIKLNGLENRVEVGKDQEFTVDTRGAGGQGKLDVTILSPSRKVVPCLVTPVTGRENSTAKFIPREEGLYAVDVTYDGHPVPGSPYTVEASLPPDPSKVKAHGPGLEGGLVGKPAEFTIDTKGAGTGGLGLTVEGPCEAKIECSDNGDGTCSVSYLPTKPGEYFVNILFEEVHIPGSPFKADIEMPFDPSKVVASGPGLEHGKVGEAGLLSVDCSEAGPGALGLEAVSDSGTKAEVSIQNNKDGTYAVTYVPLTAGMYTLTMKYGGELVPHFPARVKVEPAVDTSRIKVFGPGIEGKDVFREATTDFTVDSRPLTQVGGDHIKAHIANPSGASTECFVTDNADGTYQVEYTPFEKGLHVVEVTYDDVPIPNSPFKVAVTEGCQPSRVQAQGPGLKEAFTNKPNVFTVVTRGAGIGGLGITVEGPSESKINCRDNKDGSCSAEYIPFAPGDYDVNITYGGAHIPGSPFRVPVKDVVDPSKVKIAGPGLGSGVRARVLQSFTVDSSKAGLAPLEVRVLGPRGLVEPVNVVDNGDGTHTVTYTPSQEGPYMVSVKYADEEIPRSPFKVKVLPTYDASKVTASGPGLSSYGVPASLPVDFAIDARDAGEGLLAVQITDQEGKPKRAIVHDNKDGTYAVTYIPDKTGRYMIGVTYGGDDIPLSPYRIRATQTGDASKCLATGPGIASTVKTGEEVGFVVDAKTAGKGKVTCTVLTPDGTEAEADVIENEDGTYDIFYTAAKPGTYVIYVRFGGVDIPNSPFTVMATDGEVTAVEEAPVNACPPGFRPWVTEEAYVPVSDMNGLGFKPFDLVIPFAVRKGEITGEVHMPSGKTATPEIVDNKDGTVTVRYAPTEVGLHEMHIKYMGSHIPESPLQFYVNYPNSGSVSAYGPGLVYGVANKTATFTIVTEDAGEGGLDLAIEGPSKAEISCIDNKDGTCTVTYLPTLPGDYSILVKYNDKHIPGSPFTAKITDDSRRCSQVKLGSAADFLLDISETDLSSLTASIKAPSGRDEPCLLKRLPNNHIGISFIPREVGEHLVSIKKNGNHVANSPVSIMVVQSEIGDARRAKVYGRGLSEGRTFEMSDFIVDTRDAGYGGISLAVEGPSKVDIQTEDLEDGTCKVSYFPTVPGVYIVSTKFADEHVPGSPFTVKISGEGRVKESITRTSRAPSVATVGSICDLNLKIPEINSSDMSAHVTSPSGRVTEAEIVPMGKNSHCVRFVPQEMGVHTVSVKYRGQHVTGSPFQFTVGPLGEGGAHKVRAGGPGLERGEAGVPAEFSIWTREAGAGGLSIAVEGPSKAEITFDDHKNGSCGVSYIAQEPGNYEVSIKFNDEHIPESPYLVPVIAPSDDARRLTVMSLQESGLKVNQPASFAIRLNGAKGKIDAKVHSPSGAVEECHVSELEPDKYAVRFIPHENGVHTIDVKFNGSHVVGSPFKVRVGEPGQAGNPALVSAYGTGLEGGTTGIQSEFFINTTRAGPGTLSVTIEGPSKVKMDCQETPEGYKVMYTPMAPGNYLISVKYGGPNHIVGSPFKAKVTGQRLVSPGSANETSSILVESVTRSSTETCYSAIPKASSDASKVTSKGAGLSKAFVGQKSSFLVDCSKAGSNMLLIGVHGPTTPCEEVSMKHVGNQQYNVTYVVKERGDYVLAVKWGEEHIPGSPFHVTVP

5) >gi|153945728|ref|NP_005900.2| microtubule-associated protein 1B [Homo sapiens] (270.5 kD)

MATVVVEATEPEPSGSIANPAASTSPSLSHRFLDSKFYLLVVVGEIVTEEHLRRAIGNIELGIRSWDTNLIECNLDQELKLFVSRHSARFSPEVPGQKILHHRSDVLETVVLINPSDEAVSTEVRLMITDAARHKLLVLTGQCFENTGELILQSGSFSFQNFIEIFTDQEIGELLSTTHPANKASLTLFCPEEGDWKNSNLDRHNLQDFINIKLNSASILPEMEGLSEFTEYLSESVEVPSPFDILEPPTSGGFLKLSKPCCYIFPGGRGDSALFAVNGFNMLINGGSERKSCFWKLIRHLDRVDSILLTHIGDDNLPGINSMLQRKIAELEEEQSQGSTTNSDWMKNLISPDLGVVFLNVPENLKNPEPNIKMKRSIEEACFTLQYLNKLSMKPEPLFRSVGNTIDPVILFQKMGVGKLEMYVLNPVKSSKEMQYFMQQWTGTNKDKAEFILPNGQEVDLPISYLTSVSSLIVWHPANPAEKIIRVLFPGNSTQYNILEGLEKLKHLDFLKQPLATQKDLTGQVPTPVVKQTKLKQRADSRESLKPAAKPLPSKSVRKESKEETPEVTKVNHVEKPPKVESKEKVMVKKDKPIKTETKPSVTEKEVPSKEEPSPVKAEVAEKQATDVKPKAAKEKTVKKETKVKPEDKKEEKEKPKKEVAKKEDKTPIKKEEKPKKEEVKKEVKKEIKKEEKKEPKKEVKKETPPKEVKKEVKKEEKKEVKKEEKEPKKEIKKLPKDAKKSSTPLSEAKKPAALKPKVPKKEESVKKDSVAAGKPKEKGKIKVIKKEGKAAEAVAAAVGTGATTAAVMAAAGIAAIGPAKELEAERSLMSSPEDLTKDFEELKAEEVDVTKDIKPQLELIEDEEKLKETEPVEAYVIQKEREVTKGPAESPDEGITTTEGEGECEQTPEELEPVEKQGVDDIEKFEDEGAGFEESSETGDYEEKAETEEAEEPEEDGEEHVCVSASKHSPTEDEESAKAEADAYIREKRESVASGDDRAEEDMDEAIEKGEAEQSEEEADEEDKAEDAREEEYEPEKMEAEDYVMAVVDKAAEAGGAEEQYGFLTTPTKQLGAQSPGREPASSIHDETLPGGSESEATASDEENREDQPEEFTATSGYTQSTIEISSEPTPMDEMSTPRDVMSDETNNEETESPSQEFVNITKYESSLYSQEYSKPADVTPLNGFSEGSKTDATDGKDYNASASTISPPSSMEEDKFSRSALRDAYCSEVKASTTLDIKDSISAVSSEKVSPSKSPSLSPSPPSPLEKTPLGERSVNFSLTPNEIKVSAEAEVAPVSPEVTQEVVEEHCASPEDKTLEVVSPSQSVTGSAGHTPYYQSPTDEKSSHLPTEVIEKPPAVPVSFEFSDAKDENERASVSPMDEPVPDSESPIEKVLSPLRSPPLIGSESAYESFLSADDKASGRGAESPFEEKSGKQGSPDQVSPVSEMTSTSLYQDKQEGKSTDFAPIKEDFGQEKKTDDVEAMSSQPALALDERKLGDVSPTQIDVSQFGSFKEDTKMSISEGTVSDKSATPVDEGVAEDTYSHMEGVASVSTASVATSSFPEPTTDDVSPSLHAEVGSPHSTEVDDSLSVSVVQTPTTFQETEMSPSKEECPRPMSISPPDFSPKTAKSRTPVQDHRSEQSSMSIEFGQESPEQSLAMDFSRQSPDHPTVGAGVLHITENGPTEVDYSPSDMQDSSLSHKIPPMEEPSYTQDNDLSELISVSQVEASPSTSSAHTPSQIASPLQEDTLSDVAPPRDMSLYASLTSEKVQSLEGEKLSPKSDISPLTPRESSPLYSPTFSDSTSAVKEKTATCHSSSSPPIDAASAEPYGFRASVLFDTMQHHLALNRDLSTPGLEKDSGGKTPGDFSYAYQKPEETTRSPDEEDYDYESYEKTTRTSDVGGYYYEKIERTTKSPSDSGYSYETIGKTTKTPEDGDYSYEIIEKTTRTPEEGGYSYDISEKTTSPPEVSGYSYEKTERSRRLLDDISNGYDDSEDGGHTLGDPSYSYETTEKITSFPESEGYSYETSTKTTRTPDTSTYCYETAEKITRTPQASTYSYETSDLCYTAEKKSPSEARQDVDLCLVSSCEYKHPKTELSPSFINPNPLEWFASEEPTEESEKPLTQSGGAPPPPGGKQQGRQCDETPPTSVSESAPSQTDSDVPPETEECPSITADANIDSEDESETIPTDKTVTYKHMDPPPAPVQDRSPSPRHPDVSMVDPEALAIEQNLGKALKKDLKEKTKTKKPGTKTKSSSPVKKSDGKSKPLAASPKPAGLKESSDKVSRVASPKKKESVEKAAKPTTTPEVKAARGEEKDKETKNAANASASKSAKTATAGPGTTKTTKSSAVPPGLPVYLDLCYIPNHSNSKNVDVEFFKRVRSSYYVVSGNDPAAEEPSRAVLDALLEGKAQWGSNMQVTLIPTHDSEVMREWYQETHEKQQDLNIMVLASSSTVVMQDESFPACKIEL

6) >gi|223029410|ref|NP_006280.3| talin-1 [Homo sapiens] (269.6 kD)

MVALSLKISIGNVVKTMQFEPSTMVYDACRIIRERIPEAPAGPPSDFGLFLSDDDPKKGIWLEAGKALDYYMLRNGDTMEYRKKQRPLKIRMLDGTVKTIMVDDSKTVTDMLMTICARIGITNHDEYSLVRELMEEKKEEITGTLRKDKTLLRDEKKMEKLKQKLHTDDELNWLDHGRTLREQGVEEHETLLLRRKFFYSDQNVDSRDPVQLNLLYVQARDDILNGSHPVSFDKACEFAGFQCQIQFGPHNEQKHKAGFLDLKDFLPKEYVKQKGERKIFQAHKNCGQMSEIEAKVRYVKLARSLKTYGVSFFLVKEKMKGKNKLVPRLLGITKECVMRVDEKTKEVIQEWNLTNIKRWAASPKSFTLDFGDYQDGYYSVQTTEGEQIAQLIAGYIDIILKKKKSKDHFGLEGDEESTMLEDSVSPKKSTVLQQQYNRVGKVEHGSVALPAIMRSGASGPENFQVGSMPPAQQQITSGQMHRGHMPPLTSAQQALTGTINSSMQAVQAAQATLDDFDTLPPLGQDAASKAWRKNKMDESKHEIHSQVDAITAGTASVVNLTAGDPAETDYTAVGCAVTTISSNLTEMSRGVKLLAALLEDEGGSGRPLLQAAKGLAGAVSELLRSAQPASAEPRQNLLQAAGNVGQASGELLQQIGESDTDPHFQDALMQLAKAVASAAAALVLKAKSVAQRTEDSGLQTQVIAAATQCALSTSQLVACTKVVAPTISSPVCQEQLVEAGRLVAKAVEGCVSASQAATEDGQLLRGVGAAATAVTQALNELLQHVKAHATGAGPAGRYDQATDTILTVTENIFSSMGDAGEMVRQARILAQATSDLVNAIKADAEGESDLENSRKLLSAAKILADATAKMVEAAKGAAAHPDSEEQQQRLREAAEGLRMATNAAAQNAIKKKLVQRLEHAAKQAAASATQTIAAAQHAASTPKASAGPQPLLVQSCKAVAEQIPLLVQGVRGSQAQPDSPSAQLALIAASQSFLQPGGKMVAAAKASVPTIQDQASAMQLSQCAKNLGTALAELRTAAQKAQEACGPLEMDSALSVVQNLEKDLQEVKAAARDGKLKPLPGETMEKCTQDLGNSTKAVSSAIAQLLGEVAQGNENYAGIAARDVAGGLRSLAQAARGVAALTSDPAVQAIVLDTASDVLDKASSLIEEAKKAAGHPGDPESQQRLAQVAKAVTQALNRCVSCLPGQRDVDNALRAVGDASKRLLSDSLPPSTGTFQEAQSRLNEAAAGLNQAATELVQASRGTPQDLARASGRFGQDFSTFLEAGVEMAGQAPSQEDRAQVVSNLKGISMSSSKLLLAAKALSTDPAAPNLKSQLAAAARAVTDSINQLITMCTQQAPGQKECDNALRELETVRELLENPVQPINDMSYFGCLDSVMENSKVLGEAMTGISQNAKNGNLPEFGDAISTASKALCGFTEAAAQAAYLVGVSDPNSQAGQQGLVEPTQFARANQAIQMACQSLGEPGCTQAQVLSAATIVAKHTSALCNSCRLASARTTNPTAKRQFVQSAKEVANSTANLVKTIKALDGAFTEENRAQCRAATAPLLEAVDNLSAFASNPEFSSIPAQISPEGRAAMEPIVISAKTMLESAGGLIQTARALAVNPRDPPSWSVLAGHSRTVSDSIKKLITSMRDKAPGQLECETAIAALNSCLRDLDQASLAAVSQQLAPREGISQEALHTQMLTAVQEISHLIEPLANAARAEASQLGHKVSQMAQYFEPLTLAAVGAASKTLSHPQQMALLDQTKTLAESALQLLYTAKEAGGNPKQAAHTQEALEEAVQMMTEAVEDLTTTLNEAASAAGVVGGMVDSITQAINQLDEGPMGEPEGSFVDYQTTMVRTAKAIAVTVQEMVTKSNTSPEELGPLANQLTSDYGRLASEAKPAAVAAENEEIGSHIKHRVQELGHGCAALVTKAGALQCSPSDAYTKKELIECARRVSEKVSHVLAALQAGNRGTQACITAASAVSGIIADLDTTIMFATAGTLNREGTETFADHREGILKTAKVLVEDTKVLVQNAAGSQEKLAQAAQSSVATITRLADVVKLGAASLGAEDPETQVVLINAVKDVAKALGDLISATKAAAGKVGDDPAVWQLKNSAKVMVTNVTSLLKTVKAVEDEATKGTRALEATTEHIRQELAVFCSPEPPAKTSTPEDFIRMTKGITMATAKAVAAGNSCRQEDVIATANLSRRAIADMLRACKEAAYHPEVAPDVRLRALHYGRECANGYLELLDHVLLTLQKPSPELKQQLTGHSKRVAGSVTELIQAAEAMKGTEWVDPEDPTVIAENELLGAAAAIEAAAKKLEQLKPRAKPKEADESLNFEEQILEAAKSIAAATSALVKAASAAQRELVAQGKVGAIPANALDDGQWSQGLISAARMVAAATNNLCEAANAAVQGHASQEKLISSAKQVAASTAQLLVACKVKADQDSEAMKRLQAAGNAVKRASDNLVKAAQKAAAFEEQENETVVVKEKMVGGIAQIIAAQEEMLRKERELEEARKKLAQIRQQQYKFLPSELRDEH

7) >gi|302699237|ref|NP_886553.3| eukaryotic translation initiation factor 4 gamma 1 isoform 1 [Homo sapiens] (175.4 kD)

MNKAPQSTGPPPAPSPGLPQPAFPPGQTAPVVFSTPQATQMNTPSQPRQHFYPSRAQPPSSAASRVQSAAPARPGPAAHVYPAGSQVMMIPSQISYPASQGAYYIPGQGRSTYVVPTQQYPVQPGAPGFYPGASPTEFGTYAGAYYPAQGVQQFPTGVAPAPVLMNQPPQIAPKRERKTIRIRDPNQGGKDITEEIMSGARTASTPTPPQTGGGLEPQANGETPQVAVIVRPDDRSQGAIIADRPGLPGPEHSPSESQPSSPSPTPSPSPVLEPGSEPNLAVLSIPGDTMTTIQMSVEESTPISRETGEPYRLSPEPTPLAEPILEVEVTLSKPVPESEFSSSPLQAPTPLASHTVEIHEPNGMVPSEDLEPEVESSPELAPPPACPSESPVPIAPTAQPEELLNGAPSPPAVDLSPVSEPEEQAKEVTASMAPPTIPSATPATAPSATSPAQEEEMEEEEEEEEGEAGEAGEAESEKGGEELLPPESTPIPANLSQNLEAAAATQVAVSVPKRRRKIKELNKKEAVGDLLDAFKEANPAVPEVENQPPAGSNPGPESEGSGVPPRPEEADETWDSKEDKIHNAENIQPGEQKYEYKSDQWKPLNLEEKKRYDREFLLGFQFIFASMQKPEGLPHISDVVLDKANKTPLRPLDPTRLQGINCGPDFTPSFANLGRTTLSTRGPPRGGPGGELPRGPQAGLGPRRSQQGPRKEPRKIIATVLMTEDIKLNKAEKAWKPSSKRTAADKDRGEEDADGSKTQDLFRRVRSILNKLTPQMFQQLMKQVTQLAIDTEERLKGVIDLIFEKAISEPNFSVAYANMCRCLMALKVPTTEKPTVTVNFRKLLLNRCQKEFEKDKDDDEVFEKKQKEMDEAATAEERGRLKEELEEARDIARRRSLGNIKFIGELFKLKMLTEAIMHDCVVKLLKNHDEESLECLCRLLTTIGKDLDFEKAKPRMDQYFNQMEKIIKEKKTSSRIRFMLQDVLDLRGSNWVPRRGDQGPKTIDQIHKEAEMEEHREHIKVQQLMAKGSDKRRGGPPGPPISRGLPLVDDGGWNTVPISKGSRPIDTSRLTKITKPGSIDSNNQLFAPGGRLSWGKGSSGGSGAKPSDAASEAARPATSTLNRFSALQQAVPTESTDNRRVVQRSSLSRERGEKAGDRGDRLERSERGGDRGDRLDRARTPATKRSFSKEVEERSRERPSQPEGLRKAASLTEDRDRGRDAVKREAALPPVSPLKAALSEEELEKKSKAIIEEYLHLNDMKEAVQCVQELASPSLLFIFVRHGVESTLERSAIAREHMGQLLHQLLCAGHLSTAQYYQGLYEILELAEDMEIDIPHVWLYLAELVTPILQEGGVPMGELFREITKPLRPLGKAASLLLEILGLLCKSMGPKKVGTLWREAGLSWKEFLPEGQDIGAFVAEQKVEYTLGEESEAPGQRALPSEELNRQLEKLLKEGSSNQRVFDWIEANLSEQQIVSNTLVRALMTAVCYSAIIFETPLRVDVAVLKARAKLLQKYLCDEQKELQALYALQALVVTLEQPPNLLRMFFDALYDEDVVKEDAFYSWESSKDPAEQQGKGVALKSVTAFFKWLREAEEESDHN

8) >gi|55741719|ref|NP_055621.1| iporin [Homo sapiens] (161.1 kD)

MDSPPKLTGETLIVHHIPLVHCQVPDRQCCGGAGGGGGSTRPNPFCPPELGITQPDQDLGQADSLLFSSLHSAPGGTARSIDSTKSRSRDGRGPGAPKRHNPFLLQEGVGEPGLGDLYDDSIGDSATQQSFHLHGTGQPNFHLSSFQLPPSGPRVGRPWGTTRSRAGVVEGQEQEPVMTLDTQQCGTSHCCRPELEAETMELDECGGPGGSGSGGGASDTSGFSFDQEWKLSSDESPRNPGCSGSGDQHCRCSSTSSQSEAADQSMGYVSDSSCNSSDGVLVTFSTLYNKMHGTPRANLNSAPQSCSDSSFCSHSDPGAFYLDLQPSPFESKMSYESHHPESGGREGGYGCPHASSPELDANCNSYRPHCEPCPAVADLTACFQSQARLVVATQNYYKLVTCDLSSQSSPSPAGSSITSCSEEHTKISPPPGPGPDPGPSQPSEYYLFQKPEVQPEEQEAVSSSTQAAAAVGPTVLEGQVYTNTSPPNLSTGRQRSRSYDRSLQRSPPVRLGSLERMLSCPVRLSEGPAAMAGPGSPPRRVTSFAELAKGRKKTGGSGSPPLRVSVGDSSQEFSPIQEAQQDRGAPLDEGTCCSHSLPPMPLGPGMDLLGPDPSPPWSTQVCQGPHSSEMPPAGLRATGQGPLAQLMDPGPALPGSPANSHTQRDARARADGGGTESRPVLRYSKEQRPTTLPIQPFVFQHHFPKQLAKARALHSLSQLYSLSGCSRTQQPAPLAAPAAQVSVPAPSGEPQASTPRATGRGARKAGSEPETSRPSPLGSYSPIRSVGPFGPSTDSSASTSCSPPPEQPTATESLPPWSHSCPSAVRPATSQQPQKEDQKILTLTEYRLHGTGSLPPLGSWRSGLSRAESLARGGGEGSMATRPSNANHLSPQALKWREYRRKNPLGPPGLSGSLDRRSQEARLARRNPIFEFPGSLSAASHLNCRLNGQAVKPLPLTCPDFQDPFSLTEKPPAEFCLSPDGSSEAISIDLLQKKGLVKAVNIAVDLIVAHFGTSRDPGVKAKLGNSSVSPNVGHLVLKYLCPAVRAVLEDGLKAFVLDVIIGQRKNMPWSVVEASTQLGPSTKVLHGLYNKVSQFPELTSHTMRFNAFILGLLNIRSLEFWFNHLYNHEDIIQTHYQPWGFLSAAHTVCPGLFEELLLLLQPLALLPFSLDLLFQHRLLQSGQQQRQHKELLRVSQDLLLSAHSTLQLARARGQEGPGDVDRAAQGERVKGVGASEGGEEEEEEEETEEVAEAAGGSGRARWARGGQAGWWYQLMQSSQVYIDGSIEGSRFPRGSSNSSSEKKKGAGGGGPPQAPPPREGVVEGAEACPASEEALGRERGWPFWMGSPPDSVLAELRRSREREGPAASPAENEEGASEPSPGGIKWGHLFGSRKAQREARPTNRLPSDWLSLDKSMFQLVAQTVGSRREPEPKESLQEPHSPALPSSPPCEVQALCHHLATGPGQLSFHKGDILRVLGRAGGDWLRCSRGPDSGLVPLAYVTLTPTPSPTPGSSQN

9) >gi|31621305|ref|NP_573566.2| leucine-rich PPR motif-containing protein, mitochondrial precursor [Homo sapiens] (157.8 kD)

MAALLRSARWLLRAGAAPRLPLSLRLLPGGPGRLHAASYLPAARAGPVAGGLLSPARLYAIAAKEKDIQEESTFSSRKISNQFDWALMRLDLSVRRTGRIPKKLLQKVFNDTCRSGGLGGSHALLLLRSCGSLLPELKLEERTEFAHRIWDTLQKLGAVYDVSHYNALLKVYLQNEYKFSPTDFLAKMEEANIQPNRVTYQRLIASYCNVGDIEGASKILGFMKTKDLPVTEAVFSALVTGHARAGDMENAENILTVMRDAGIEPGPDTYLALLNAYAEKGDIDHVKQTLEKVEKSELHLMDRDLLQIIFSFSKAGYPQYVSEILEKVTCERRYIPDAMNLILLLVTEKLEDVALQILLACPVSKEDGPSVFGSFFLQHCVTMNTPVEKLTDYCKKLKEVQMHSFPLQFTLHCALLANKTDLAKALMKAVKEEGFPIRPHYFWPLLVGRRKEKNVQGIIEILKGMQELGVHPDQETYTDYVIPCFDSVNSARAILQENGCLSDSDMFSQAGLRSEAANGNLDFVLSFLKSNTLPISLQSIRSSLLLGFRRSMNINLWSEITELLYKDGRYCQEPRGPTEAVGYFLYNLIDSMSDSEVQAKEEHLRQYFHQLEKMNVKIPENIYRGIRNLLESYHVPELIKDAHLLVESKNLDFQKTVQLTSSELESTLETLKAENQPIRDVLKQLILVLCSEENMQKALELKAKYESDMVTGGYAALINLCCRHDKVEDALNLKEEFDRLDSSAVLDTGKYVGLVRVLAKHGKLQDAINILKEMKEKDVLIKDTTALSFFHMLNGAALRGEIETVKQLHEAIVTLGLAEPSTNISFPLVTVHLEKGDLSTALEVAIDCYEKYKVLPRIHDVLCKLVEKGETDLIQKAMDFVSQEQGEMVMLYDLFFAFLQTGNYKEAKKIIETPGIRARSARLQWFCDRCVANNQVETLEKLVELTQKLFECDRDQMYYNLLKLYKINGDWQRADAVWNKIQEENVIPREKTLRLLAEILREGNQEVPFDVPELWYEDEKHSLNSSSASTTEPDFQKDILIACRLNQKKGAYDIFLNAKEQNIVFNAETYSNLIKLLMSEDYFTQAMEVKAFAETHIKGFTLNDAANSRLIITQVRRDYLKEAVTTLKTVLDQQQTPSRLAVTRVIQALAMKGDVENIEVVQKMLNGLEDSIGLSKMVFINNIALAQIKNNNIDAAIENIENMLTSENKVIEPQYFGLAYLFRKVIEEQLEPAVEKISIMAERLANQFAIYKPVTDFFLQLVDAGKVDDARALLQRCGAIAEQTPILLLFLLRNSRKQGKASTVKSVLELIPELNEKEEAYNSLMKSYVSEKDVTSAKALYEHLTAKNTKLDDLFLKRYASLLKYAGEPVPFIEPPESFEFYAQQLRKLRENSS

10) >gi|7662238|ref|NP_055792.1| apoptotic chromatin condensation inducer in the nucleus isoform 1 [Homo sapiens] (151.8 kD)

MWRRKHPRTSGGTRGVLSGNRGVEYGSGRGHLGTFEGRWRKLPKMPEAVGTDPSTSRKMAELEEVTLDGKPLQALRVTDLKAALEQRGLAKSGQKSALVKRLKGALMLENLQKHSTPHAAFQPNSQIGEEMSQNSFIKQYLEKQQELLRQRLEREAREAAELEEASAESEDEMIHPEGVASLLPPDFQSSLERPELELSRHSPRKSSSISEEKGDSDDEKPRKGERRSSRVRQARAAKLSEGSQPAEEEEDQETPSRNLRVRADRNLKTEEEEEEEEEEEEDDEEEEGDDEGQKSREAPILKEFKEEGEEIPRVKPEEMMDERPKTRSQEQEVLERGGRFTRSQEEARKSHLARQQQEKEMKTTSPLEEEEREIKSSQGLKEKSKSPSPPRLTEDRKKASLVALPEQTASEEETPPPLLTKEASSPPPHPQLHSEEEIEPMEGPAPPVLIQLSPPNTDADTRELLVSQHTVQLVGGLSPLSSPSDTKAESPAEKVPEESVLPLVQKSTLADYSAQKDLEPESDRSAQPLPLKIEELALAKGITEECLKQPSLEQKEGRRASHTLLPSHRLKQSADSSSSRSSSSSSSSSRSRSRSPDSSGSRSHSPLRSKQRDVAQARTHANPRGRPKMGSRSTSESRSRSRSRSRSASSNSRKSLSPGVSRDSSTSYTETKDPSSGQEVATPPVPQLQVCEPKERTSTSSSSVQARRLSQPESAEKHVTQRLQPERGSPKKCEAEEAEPPAATQPQTSETQTSHLPESERIHHTVEEKEEVTMDTSENRPENDVPEPPMPIADQVSNDDRPEGSVEDEEKKESSLPKSFKRKISVVSATKGVPAGNSDTEGGQPGRKRRWGASTATTQKKPSISITTESLKSLIPDIKPLAGQEAVVDLHADDSRISEDETERNGDDGTHDKGLKICRTVTQVVPAEGQENGQREEEEEEKEPEAEPPVPPQVSVEVALPPPAEHEVKKVTLGDTLTRRSISQQKSGVSITIDDPVRTAQVPSPPRGKISNIVHISNLVRPFTLGQLKELLGRTGTLVEEAFWIDKIKSHCFVTYSTVEEAVATRTALHGVKWPQSNPKFLCADYAEQDELDYHRGLLVDRPSETKTEEQGIPRPLHPPPPPPVQPPQHPRAEQREQERAVREQWAEREREMERRERTRSEREWDRDKVREGPRSRSRSRDRRRKERAKSKEKKSEKKEKAQEEPPAKLLDDLFRKTKAAPCIYWLPLTDSQIVQKEAERAERAKEREKRRKEQEEEEQKEREKEAERERNRQLEREKRREHSRERDRERERERERDRGDRDRDRERDRERGRERDRRDTKRHSRSRSRSTPVRDRGGRR

11) >gi|24431935|ref|NP_065393.1| reticulon-4 isoform A [Homo sapiens] (129.9 kD)

MEDLDQSPLVSSSDSPPRPQPAFKYQFVREPEDEEEEEEEEEEDEDEDLEELEVLERKPAAGLSAAPVPTAPAAGAPLMDFGNDFVPPAPRGPLPAAPPVAPERQPSWDPSPVSSTVPAPSPLSAAAVSPSKLPEDDEPPARPPPPPPASVSPQAEPVWTPPAPAPAAPPSTPAAPKRRGSSGSVDETLFALPAASEPVIRSSAENMDLKEQPGNTISAGQEDFPSVLLETAASLPSLSPLSAASFKEHEYLGNLSTVLPTEGTLQENVSEASKEVSEKAKTLLIDRDLTEFSELEYSEMGSSFSVSPKAESAVIVANPREEIIVKNKDEEEKLVSNNILHNQQELPTALTKLVKEDEVVSSEKAKDSFNEKRVAVEAPMREEYADFKPFERVWEVKDSKEDSDMLAAGGKIESNLESKVDKKCFADSLEQTNHEKDSESSNDDTSFPSTPEGIKDRSGAYITCAPFNPAATESIATNIFPLLGDPTSENKTDEKKIEEKKAQIVTEKNTSTKTSNPFLVAAQDSETDYVTTDNLTKVTEEVVANMPEGLTPDLVQEACESELNEVTGTKIAYETKMDLVQTSEVMQESLYPAAQLCPSFEESEATPSPVLPDIVMEAPLNSAVPSAGASVIQPSSSPLEASSVNYESIKHEPENPPPYEEAMSVSLKKVSGIKEEIKEPENINAALQETEAPYISIACDLIKETKLSAEPAPDFSDYSEMAKVEQPVPDHSELVEDSSPDSEPVDLFSDDSIPDVPQKQDETVMLVKESLTETSFESMIEYENKEKLSALPPEGGKPYLESFKLSLDNTKDTLLPDEVSTLSKKEKIPLQMEELSTAVYSNDDLFISKEAQIRETETFSDSSPIEIIDEFPTLISSKTDSFSKLAREYTDLEVSHKSEIANAPDGAGSLPCTELPHDLSLKNIQPKVEEKISFSDDFSKNGSATSKVLLLPPDVSALATQAEIESIVKPKVLVKEAEKKLPSDTEKEDRSPSAIFSAELSKTSVVDLLYWRDIKKTGVVFGASLFLLLSLTVFSIVSVTAYIALALLSVTISFRIYKGVIQAIQKSDEGHPFRAYLESEVAISEELVQKYSNSALGHVNCTIKELRRLFLVDDLVDSLKFAVLMWVFTYVGALFNGLTLLILALISLFSVPVIYERHQAQIDHYLGLANKNVKDAMAKIQAKIPGLKRKAE

12) >gi|7669550|ref|NP_054706.1| vinculin isoform meta-VCL [Homo sapiens] (123.7 kD)

MPVFHTRTIESILEPVAQQISHLVIMHEEGEVDGKAIPDLTAPVAAVQAAVSNLVRVGKETVQTTEDQILKRDMPPAFIKVENACTKLVQAAQMLQSDPYSVPARDYLIDGSRGILSGTSDLLLTFDEAEVRKIIRVCKGILEYLTVAEVVETMEDLVTYTKNLGPGMTKMAKMIDERQQELTHQEHRVMLVNSMNTVKELLPVLISAMKIFVTTKNSKNQGIEEALKNRNFTVEKMSAEINEIIRVLQLTSWDEDAWASKDTEAMKRALASIDSKLNQAKGWLRDPSASPGDAGEQAIRQILDEAGKVGELCAGKERREILGTCKMLGQMTDQVADLRARGQGSSPVAMQKAQQVSQGLDVLTAKVENAARKLEAMTNSKQSIAKKIDAAQNWLADPNGGPEGEEQIRGALAEARKIAELCDDPKERDDILRSLGEISALTSKLADLRRQGKGDSPEARALAKQVATALQNLQTKTNRAVANSRPAKAAVHLEGKIEQAQRWIDNPTVDDRGVGQAAIRGLVAEGHRLANVMMGPYRQDLLAKCDRVDQLTAQLADLAARGEGESPQARALASQLQDSLKDLKARMQEAMTQEVSDVFSDTTTPIKLLAVAATAPPDAPNREEVFDERAANFENHSGKLGATAEKAAAVGTANKSTVEGIQASVKTARELTPQVVSAARILLRNPGNQAAYEHFETMKNQWIDNVEKMTGLVDEAIDTKSLLDASEEAIKKDLDKCKVAMANIQPQMLVAGATSIARRANRILLVAKREVENSEDPKFREAVKAASDELSKTISPMVMDAKAVAGNISDPGLQKSFLDSGYRILGAVAKVREAFQPQEPDFPPPPPDLEQLRLTDELAPPKPPLPEGEVPPPRPPPPEEKDEEFPEQKAGEVINQPMMMAARQLHDEARKWSSKPGIPAAEVGIGVVAEADAADAAGFPVPPDMEDDYEPELLLMPSNQPVNQPILAAAQSLHREATKWSSKGNDIIAAAKRMALLMAEMSRLVRGGSGTKRALIQCAKDIAKASDEVTRLAKEVAKQCTDKRIRTNLLQVCERIPTISTQLKILSTVKATMLGRTNISDEESEQATEMLVHNAQNLMQSVKETVREAEAASIKIRTDAGFTLRWVRKTPWYQ

13) >gi|134133226|ref|NP_001077007.1| POTE ankyrin domain family member E [Homo sapiens] (121.3 kD)

MVVEVDSMPAASSVKKPFGLRSKMGKWCCRCFPCYRESGKSNVGTSGDHDDSAMKTLRSKMGKWCHHCFPCCRGSGKSNVGASGDHDDSAMKTLRNKMGKWCCHCFPCCRGSGKSKVGAWGDYDDSAFMEPRYHVRGEDLDKLHRAAWWGKVPRKDLIVMLRDTDVNKKDKQKRTALHLASANGNSEVVKLLLDRRCQLNVLDNKKRTALIKAVQCQEDECALMLLEHGTDPNIPDEYGNTTLHYAIYNEDKLMAKALLLYGADIESKNKHGLTPLLLGVHEQKQQVVKFLIKKKANLNALDRYGRTALILAVCCGSASIVSLLLEQNIDVSSQDLSGQTAREYAVSSHHHVICQLLSDYKEKQMLKISSENSNPEQELKLTSEEESQRFKGSENSQPEKMSQELEINKDGDREVEEEMKKHESNNVGLLENLTNGVTAGNGDNGLIPQRKSRTPENQQFPDNESEEYHRICELLSDYKEKQMPKYSSENSNPEQDLKLTSEEESQRLKGSENGQPEKRSQEPEINKDGDRELENFMAIEEMKKHGSTHVGFPENLTNGATAGNGDDGLIPPRKSRTPESQQFPDTENEEYHSDEQNDTQKQFCEEQNTGILHDEILIHEEKQIEVVEKMNSELSLSCKKEKDVLHENSTLREEIAMLRLELDTMKHQSQLREKKYLEDIESVKKKNDNLLKALQLNELTMDDDTAVLVIDNGSGMCKAGFAGDDAPRAVFPSIVGRPRQQGMMGGMHQKESYVGKEAQSKRGILTLKYPMEHGIITNWDDMEKIWHHTFYNELRVAPEEHPILLTEAPLNPKANREKMTQIMFETFNTPAMYVAIQAVPSLYTSGRTTGIVMDSGDGVTHTVPIYEGNALPHATLRLDLAGRELPDYLMKILTERGYRFTTMAEREIVRDIKEKLCYVALDFEQEMATAASSSSLEKSYELPDGQVITIGNERFRCPEALFQPCFLGMESCGIHETTFNSIMKSDVDIRKDLYTNTVLSGGTTMYPGMAHRMQKEIAALAPSMMKIRIIAPPKRKYSVWVGGSILASLSTFQQMWISKQEYDESGPSIVHRKCF

14) >gi|6006011|ref|NP_005492.1| integrin alpha-3 isoform b precursor [Homo sapiens] (118.7 kD)

MGPGPSRAPRAPRLMLCALALMVAAGGCVVSAFNLDTRFLVVKEAGNPGSLFGYSVALHRQTERQQRYLLLAGAPRELAVPDGYTNRTGAVYLCPLTAHKDDCERMNITVKNDPGHHIIEDMWLGVTVASQGPAGRVLVCAHRYTQVLWSGSEDQRRMVGKCYVRGNDLELDSSDDWQTYHNEMCNSNTDYLETGMCQLGTSGGFTQNTVYFGAPGAYNWKGNSYMIQRKEWDLSEYSYKDPEDQGNLYIGYTMQVGSFILHPKNITIVTGAPRHRHMGAVFLLSQEAGGDLRRRQVLEGSQVGAYFGSAIALADLNNDGWQDLLVGAPYYFERKEEVGGAIYVFMNQAGTSFPAHPSLLLHGPSGSAFGLSVASIGDINQDGFQDIAVGAPFEGLGKVYIYHSSSKGLLRQPQQVIHGEKLGLPGLATFGYSLSGQMDVDENFYPDLLVGSLSDHIVLLRARPVINIVHKTLVPRPAVLDPALCTATSCVQVELCFAYNQSAGNPNYRRNITLAYTLEADRDRRPPRLRFAGSESAVFHGFFSMPEMRCQKLELLLMDNLRDKLRPIIISMNYSLPLRMPDRPRLGLRSLDAYPILNQAQALENHTEVQFQKECGPDNKCESNLQMRAAFVSEQQQKLSRLQYSRDVRKLLLSINVTNTRTSERSGEDAHEALLTLVVPPALLLSSVRPPGACQANETIFCELGNPFKRNQRMELLIAFEVIGVTLHTRDLQVQLQLSTSSHQDNLWPMILTLLVDYTLQTSLSMVNHRLQSFFGGTVMGESGMKTVEDVGSPLKYEFQVGPMGEGLVGLGTLVLGLEWPYEVSNGKWLLYPTEITVHGNGSWPCRPPGDLINPLNLTLSDPGDRPSSPQRRRRQLDPGGGQGPPPVTLAAAKKAKSETVLTCATGRAHCVWLECPIPDAPVVTNVTVKARVWNSTFIEDYRDFDRVRVNGWATLFLRTSIPTINMENKTTWFSVDIDSELVEELPAEIELWLVLVAVGAGLLLLGLIILLLWKCDFFKRTRYYQIMPKYHAVRIREEERYPPPGSTLPTKKHWVTSWQTRDQYY

15) >gi|188497758|ref|NP_055662.3| ubiquitin-associated protein 2-like isoform a [Homo sapiens] (114.5 kD)

MMTSVGTNRARGNWEQPQNQNQTQHKQRPQATAEQIRLAQMISDHNDADFEEKVKQLIDITGKNQDECVIALHDCNGDVNRAINVLLEGNPDTHSWEMVGKKKGVSGQKDGGQTESNEEGKENRDRDRDYSRRRGGPPRRGRGASRGREFRGQENGLDGTKSGGPSGRGTERGRRGRGRGRGGSGRRGGRFSAQGMGTFNPADYAEPANTDDNYGNSSGNTWNNTGHFEPDDGTSAWRTATEEWGTEDWNEDLSETKIFTASNVSSVPLPAENVTITAGQRIDLAVLLGKTPSTMENDSSNLDPSQAPSLAQPLVFSNSKQTAISQPASGNTFSHHSMVSMLGKGFGDVGEAKGGSTTGSQFLEQFKTAQALAQLAAQHSQSGSTTTSSWDMGSTTQSPSLVQYDLKNPSDSAVHSPFTKRQAFTPSSTMMEVFLQEKSPAVATSTAAPPPPSSPLPSKSTSAPQMSPGSSDNQSSSPQPAQQKLKQQKKKASLTSKIPALAVEMPGSADISGLNLQFGALQFGSEPVLSDYESTPTTSASSSQAPSSLYTSTASESSSTISSNQSQESGYQSGPIQSTTYTSQNNAQGPLYEQRSTQTRRYPSSISSSPQKDLTQAKNGFSSVQATQLQTTQSVEGATGSAVKSDSPSTSSIPPLNETVSAASLLTTTNQHSSSLGGLSHSEEIPNTTTTQHSSTLSTQQNTLSSSTSSGRTSTSTLLHTSVESEANLHSSSSTFSTTSSTVSAPPPVVSVSSSLNSGSSLGLSLGSNSTVTASTRSSVATTSGKAPPNLPPGVPPLLPNPYIMAPGLLHAYPPQVYGYDDLQMLQTRFPLDYYSIPFPTPTTPLTGRDGSLASNPYSGDLTKFGRGDASSPAPATTLAQPQQNQTQTHHTTQQTFLNPALPPGYSYTSLPYYTGVPGLPSTFQYGPAVFPVAPTSSKQHGVNVSVNASATPFQQPSGYGSHGYNTGVSVTSSNTGVPDISGSVYSKTQQSFEKQGFHSGTPAASFNLPSALGSGGPINPATAAAYPPAPFMHILTPHQQPHSQILHHHLQQDGQTGSGQRSQTSSIPQKPQTNKSAYNSYSWGAN

16) >gi|388240768|ref|NP_001252518.1| reticulon-3 isoform e [Homo sapiens] (112.5 kD)

MAEPSAATQSHSISSSSFGAEPSAPGGGGSPGACPALGTKSCSSSCADSFVSSSSSQPVSLFSTSQEGLSSLCSDEPSSEIMTSSFLSSSEIHNTGLTILHGEKSHVLGSQPILAKEGKDHLDLLDMKKMEKPQGTSNNVSDSSVSLAAGVHCDRPSIPASFPEHPAFLSKKIGQVEEQIDKETKNPNGVSSREAKTALDADDRFTLLTAQKPPTEYSKVEGIYTYSLSPSKVSGDDVIEKDSPESPFEVIIDKAAFDKEFKDSYKESTDDFGSWSVHTDKESSEDISETNDKLFPLRNKEAGRYPMSALLSRQFSHTNAALEEVSRCVNDMHNFTNEILTWDLVPQVKQQTDKSSDCITKTTGLDMSEYNSEIPVVNLKTSTHQKTPVCSIDGSTPITKSTGDWAEASLQQENAITGKPVPDSLNSTKEFSIKGVQGNMQKQDDTLAELPGSPPEKCDSLGSGVATVKVVLPDDHLKDEMDWQSSALGEITEADSSGESDDTVIEDITADTSFENNKIQAEKPVSIPSAVVKTGEREIKEIPSCEREEKTSKNFEELVSDSELHQDQPDILGRSPASEAACSKVPDTNVSLEDVSEVAPEKPITTENPKLPSTVSPNVFNETEFSLNVTTSAYLESLHGKNVKHIDDSSPEDLIAAFTETRDKGIVDSERNAFKAISEKMTDFKTTPPVEVLHENESGGSEIKDIGSKYSEQSKETNGSEPLGVFPTQGTPVASLDLEQEQLTIKALKELGERQVEKSTSAQRDAELPSEEVLKQTFTFAPESWPQRSYDILERNVKNGSDLGISQKPITIRETTRVDAVSSLSKTELVKKHVLARLLTDFSVHDLIFWRDVKKTGFVFGTTLIMLLSLAAFSVISVVSYLILALLSVTISFRIYKSVIQAVQKSEEGHPFKAYLDVDITLSSEAFHNYMNAAMVHINRALKLIIRLFLVEDLVDSLKLAVFMWLMTYVGAVFNGITLLILAELLIFSVPIVYEKYKTQIDHYVGIARDQTKSIVEKIQAKLPGIAKKKAE

17) >gi|22024390|ref|NP_647479.2| potassium voltage-gated channel subfamily H member 5 isoform 1 [Homo sapiens] (111.8 kD)

MPGGKRGLVAPQNTFLENIVRRSSESSFLLGNAQIVDWPVVYSNDGFCKLSGYHRADVMQKSSTCSFMYGELTDKKTIEKVRQTFDNYESNCFEVLLYKKNRTPVWFYMQIAPIRNEHEKVVLFLCTFKDITLFKQPIEDDSTKGWTKFARLTRALTNSRSVLQQLTPMNKTEVVHKHSRLAEVLQLGSDILPQYKQEAPKTPPHIILHYCAFKTTWDWVILILTFYTAIMVPYNVSFKTKQNNIAWLVLDSVVDVIFLVDIVLNFHTTFVGPGGEVISDPKLIRMNYLKTWFVIDLLSCLPYDIINAFENVDEGISSLFSSLKVVRLLRLGRVARKLDHYLEYGAAVLVLLVCVFGLVAHWLACIWYSIGDYEVIDEVTNTIQIDSWLYQLALSIGTPYRYNTSAGIWEGGPSKDSLYVSSLYFTMTSLTTIGFGNIAPTTDVEKMFSVAMMMVGSLLYATIFGNVTTIFQQMYANTNRYHEMLNNVRDFLKLYQVPKGLSERVMDYIVSTWSMSKGIDTEKVLSICPKDMRADICVHLNRKVFNEHPAFRLASDGCLRALAVEFQTIHCAPGDLIYHAGESVDALCFVVSGSLEVIQDDEVVAILGKGDVFGDIFWKETTLAHACANVRALTYCDLHIIKREALLKVLDFYTAFANSFSRNLTLTCNLRKRIIFRKISDVKKEEEERLRQKNEVTLSIPVDHPVRKLFQKFKQQKELRNQGSTQGDPERNQLQVESRSLQNGASITGTSVVTVSQITPIQTSLAYVKTSESLKQNNRDAMELKPNGGADQKCLKVNSPIRMKNGNGKGWLRLKNNMGAHEEKKEDWNNVTKAESMGLLSEDPKSSDSENSVTKNPLRKTDSCDSGITKSDLRLDKAGEARSPLEHSPIQADAKHPFYPIPEQALQTTLQEVKHELKEDIQLLSCRMTALEKQVAEILKILSEKSVPQASSPKSQMPLQVPPQIPCQDIFSVSRPESPESDKDEIHF

18) >gi|29029559|ref|NP_001307.2| exportin-2 isoform 1 [Homo sapiens] (110.3 kD)

MELSDANLQTLTEYLKKTLDPDPAIRRPAEKFLESVEGNQNYPLLLLTLLEKSQDNVIKVCASVTFKNYIKRNWRIVEDEPNKICEADRVAIKANIVHLMLSSPEQIQKQLSDAISIIGREDFPQKWPDLLTEMVNRFQSGDFHVINGVLRTAHSLFKRYRHEFKSNELWTEIKLVLDAFALPLTNLFKATIELCSTHANDASALRILFSSLILISKLFYSLNFQDLPEFFEDNMETWMNNFHTLLTLDNKLLQTDDEEEAGLLELLKSQICDNAALYAQKYDEEFQRYLPRFVTAIWNLLVTTGQEVKYDLLVSNAIQFLASVCERPHYKNLFEDQNTLTSICEKVIVPNMEFRAADEEAFEDNSEEYIRRDLEGSDIDTRRRAACDLVRGLCKFFEGPVTGIFSGYVNSMLQEYAKNPSVNWKHKDAAIYLVTSLASKAQTQKHGITQANELVNLTEFFVNHILPDLKSANVNEFPVLKADGIKYIMIFRNQVPKEHLLVSIPLLINHLQAESIVVHTYAAHALERLFTMRGPNNATLFTAAEIAPFVEILLTNLFKALTLPGSSENEYIMKAIMRSFSLLQEAIIPYIPTLITQLTQKLLAVSKNPSKPHFNHYMFEAICLSIRITCKANPAAVVNFEEALFLVFTEILQNDVQEFIPYVFQVMSLLLETHKNDIPSSYMALFPHLLQPVLWERTGNIPALVRLLQAFLERGSNTIASAAADKIPGLLGVFQKLIASKANDHQGFYLLNSIIEHMPPESVDQYRKQIFILLFQRLQNSKTTKFIKSFLVFINLYCIKYGALALQEIFDGIQPKMFGMVLEKIIIPEIQKVSGNVEKKICAVGITKLLTECPPMMDTEYTKLWTPLLQSLIGLFELPEDDTIPDEEHFIDIEDTPGYQTAFSQLAFAGKKEHDPVGQMVNNPKIHLAQSLHKLSTACPGRVPSMVSTSLNAEALQYLQGYLQAASVTLL

19) >gi|32967311|ref|NP_004422.2| ephrin type-A receptor 2 precursor [Homo sapiens] (108.2 kD)

MELQAARACFALLWGCALAAAAAAQGKEVVLLDFAAAGGELGWLTHPYGKGWDLMQNIMNDMPIYMYSVCNVMSGDQDNWLRTNWVYRGEAERIFIELKFTVRDCNSFPGGASSCKETFNLYYAESDLDYGTNFQKRLFTKIDTIAPDEITVSSDFEARHVKLNVEERSVGPLTRKGFYLAFQDIGACVALLSVRVYYKKCPELLQGLAHFPETIAGSDAPSLATVAGTCVDHAVVPPGGEEPRMHCAVDGEWLVPIGQCLCQAGYEKVEDACQACSPGFFKFEASESPCLECPEHTLPSPEGATSCECEEGFFRAPQDPASMPCTRPPSAPHYLTAVGMGAKVELRWTPPQDSGGREDIVYSVTCEQCWPESGECGPCEASVRYSEPPHGLTRTSVTVSDLEPHMNYTFTVEARNGVSGLVTSRSFRTASVSINQTEPPKVRLEGRSTTSLSVSWSIPPPQQSRVWKYEVTYRKKGDSNSYNVRRTEGFSVTLDDLAPDTTYLVQVQALTQEGQGAGSKVHEFQTLSPEGSGNLAVIGGVAVGVVLLLVLAGVGFFIHRRRKNQRARQSPEDVYFSKSEQLKPLKTYVDPHTYEDPNQAVLKFTTEIHPSCVTRQKVIGAGEFGEVYKGMLKTSSGKKEVPVAIKTLKAGYTEKQRVDFLGEAGIMGQFSHHNIIRLEGVISKYKPMMIITEYMENGALDKFLREKDGEFSVLQLVGMLRGIAAGMKYLANMNYVHRDLAARNILVNSNLVCKVSDFGLSRVLEDDPEATYTTSGGKIPIRWTAPEAISYRKFTSASDVWSFGIVMWEVMTYGERPYWELSNHEVMKAINDGFRLPTPMDCPSAIYQLMMQCWQQERARRPKFADIVSILDKLIRAPDSLKTLADFDPRVSIRLPSTSGSEGVPFRTVSEWLESIKMQQYTEHFMAAGYTAIEKVVQMTNDDIKRIGVRLPGHQKRIAYSLLGLKDQVNTVGIPI

20) >gi|530418825|ref|XP_005260998.1| PREDICTED: trifunctional purine biosynthetic protein adenosine-3 isoform X1 [Homo sapiens] (107.7 kD)

MAARVLIIGSGGREHTLAWKLAQSHHVKQVLVAPGNAGTACSEKISNTAISISDHTALAQFCKEKKIEFVVVGPEAPLAAGIVGNLRSAGVQCFGPTAEAAQLESSKRFAKEFMDRHGIPTAQWKAFTKPEEACSFILSADFPALVVKASGLAAGKGVIVAKSKEEACKAVQEIMQEKAFGAAGETIVIEELLDGEEVSCLCFTDGKTVAPMPPAQDHKRLLEGDGGPNTGGMGAYCPAPQVSNDLLLKIKDTVLQRTVDGMQQEGTPYTGILYAGIMLTKNGPKVLEFNCRFGDPECQVILPLLKSDLYEVIQSTLDGLLCTSLPVWLENHTALTVVMASKGYPGDYTKGVEITGFPEAQALGLEVFHAGTALKNGKVVTHGGRVLAVTAIRENLISALEEAKKGLAAIKFEGAIYRKDVGFRAIAFLQQPRSLTYKESGVDIAAGNMLVKKIQPLAKATSRSGCKVDLGGFAGLFDLKAAGFKDPLLASGTDGVGTKLKIAQLCNKHDTIGQDLVAMCVNDILAQGAEPLFFLDYFSCGKLDLSVTEAVVAGIAKACGKAGCALLGGETAEMPDMYPPGEYDLAGFAVGAMERDQKLPHLERITEGDVVVGIASSGLHSNGFSLVRKIVAKSSLQYSSPAPDGCGDQTLGDLLLTPTRIYSHSLLPVLRSGHVKAFAHITGGGLLENIPRVLPEKLGVDLDAQTWRIPRVFSWLQQEGHLSEEEMARTFNCGVGAVLVVSKEQTEQILRDIQQHKEEAWVIGSVVARAEGSPRVKVKNLIESMQINGSVLKNGSLTNHFSFEKKKARVAVLISGTGSNLQALIDSTREPNSSAQIDIVISNKAAVAGLDKAERAGIPTRVINHKLYKNRVEFDSAIDLVLEEFSIDIVCLAGFMRILSGPFVQKWNGKMLNIHPSLLPSFKGSNAHEQALETGVTVTGCTVHFVAEDVDAGQIILQEAVPVKRGDTVATLSERVKLAEHKIFPAALQLVASGTVQLGENGKICWVKEE

21) >gi|259155302|ref|NP_001158884.1| nuclear factor NF-kappa-B p105 subunit isoform 2 [Homo sapiens] (105.3 kD)

MAEDDPYLGRPEQMFHLDPSLTHTIFNPEVFQPQMALPTDGPYLQILEQPKQRGFRFRYVCEGPSHGGLPGASSEKNKKSYPQVKICNYVGPAKVIVQLVTNGKNIHLHAHSLVGKHCEDGICTVTAGPKDMVVGFANLGILHVTKKKVFETLEARMTEACIRGYNPGLLVHPDLAYLQAEGGGDRQLGDREKELIRQAALQQTKEMDLSVVRLMFTAFLPDSTGSFTRRLEPVVSDAIYDSKAPNASNLKIVRMDRTAGCVTGGEEIYLLCDKVQKDDIQIRFYEEEENGGVWEGFGDFSPTDVHRQFAIVFKTPKYKDINITKPASVFVQLRRKSDLETSEPKPFLYYPEIKDKEEVQRKRQKLMPNFSDSFGGGSGAGAGGGGMFGSGGGGGGTGSTGPGYSFPHYGFPTYGGITFHPGTTKSNAGMKHGTMDTESKKDPEGCDKSDDKNTVNLFGKVIETTEQDQEPSEATVGNGEVTLTYATGTKEESAGVQDNLFLEKAMQLAKRHANALFDYAVTGDVKMLLAVQRHLTAVQDENGDSVLHLAIIHLHSQLVRDLLEVTSGLISDDIINMRNDLYQTPLHLAVITKQEDVVEDLLRAGADLSLLDRLGNSVLHLAAKEGHDKVLSILLKHKKAALLLDHPNGDGLNAIHLAMMSNSLPCLLLLVAAGADVNAQEQKSGRTALHLAVEHDNISLAGCLLLEGDAHVDSTTYDGTTPLHIAAGRGSTRLAALLKAAGADPLVENFEPLYDLDDSWENAGEDEGVVPGTTPLDMATSWQVFDILNGKPYEPEFTSDDLLAQGDMKQLAEDVKLQLYKLLEIPDPDKNWATLAQKLGLGILNNAFRLSPAPSKTLMDNYEVSGGTVRELVEALRQMGYTEAIEVIQAASSPVKTTSQAHSLPLSPASTRQQIDELRDSDSVCDSGVETSFRKLSFTESLTSGASLLTLNKMPHDYGQEGPLEGKI

22) >gi|578834952|ref|XP_006723469.1| PREDICTED: alpha-actinin-4 isoform X3 [Homo sapiens] (104.8 kD)

MVDYHAANQSYQYGPSSAGNGAGGGGSMGDYMAQEDDWDRDLLLDPAWEKQQRKTFTAWCNSHLRKAGTQIENIDEDFRDGLKLMLLLEVISGERLPKPERGKMRVHKINNVNKALDFIASKGVKLVSIGAEEIVDGNAKMTLGMIWTIILRFAIQDISVEETSAKEGLLLWCQRKTAPYKNVNVQNFHISWKDGLAFNALIHRHRPELIEYDKLRKDDPVTNLNNAFEVAEKYLDIPKMLDAEDIVGTLRPDEKAIMTYVSCFYHAFSGAQKAETAANRICKVLAVNQENEHLMEDYEKLASDLLEWIRRTIPWLEDRVPQKTIQEMQQKLEDFRDYRRVHKPPKVQEKCQLEINFNTLQTKLRLSNRPAFMPSEGKMVSDINNGWQHLEQAEKGYEEWLLNEIRRLERLDHLAEKFRQKASIHEAWTDGKEAMLKHRDYETATLSDIKALIRKHEAFESDLAAHQDRVEQIAAIAQELNELDYYDSHNVNTRCQKICDQWDALGSLTHSRREALEKTEKQLEAIDQLHLEYAKRAAPFNNWMESAMEDLQDMFIVHTIEEIEGLISAHDQFKSTLPDADREREAILAIHKEAQRIAESNHIKLSGSNPYTTVTPQIINSKWEKVQQLVPKRDHALLEEQSKQQSNEHLRRQFASQANVVGPWIQTKMEEIGRISIEMNGTLEDQLSHLKQYERSIVDYKPNLDLLEQQHQLIQEALIFDNKHTNYTMEHIRVGWEQLLTTIARTINEVENQILTRDAKGISQEQMQEFRASFNHFDKDHGGALGPEEFKACLISLGYDVENDRQGEAEFNRIMSLVDPNHSGLVTFQAFIDFMSRETTDTDTADQVIASFKVLAGDKNFITAEELRRELPPDQAEYCIARMAPYQGPDAVPGALDYKSFSTALYGESDL

23) >gi|21264343|ref|NP_002958.2| scaffold attachment factor B1 isoform 3 [Homo sapiens] (102.6 kD)

MAETLSGLGDSGAAGAAALSSASSETGTRRLSDLRVIDLRAELRKRNVDSSGNKSVLMERLKKAIEDEGGNPDEIEITSEGNKKTSKRSSKGRKPEEEGVEDNGLEENSGDGQEDVETSLENLQDIDIMDISVLDEAEIDNGSVADCVEDDDADNLQESLSDSRELVEGEMKELPEQLQEHAIEDKETINNLDTSSSDFTILQEIEEPSLEPENEKILDILGETCKSEPVKEESSELEQPFAQDTSSVGPDRKLAEEEDLFDSAHPEEGDLDLASESTAHAQSSKADSLLAVVKREPAEQPGDGERTDCEPVGLEPAVEQSSAASELAEASSEELAEAPTEAPSPEARDSKEDGRKFDFDACNEVPPAPKESSTSEGADQKMSSPEDDSDTKRLSKEEKGRSSCGRNFWVSGLSSTTRATDLKNLFSKYGKVVGAKVVTNARSPGARCYGFVTMSTAEEATKCINHLHKTELHGKMISVEKAKNEPVGKKTSDKRDSDGKKEKSSNSDRSTNLKRDDKCDRKDDAKKGDDGSGEKSKDQDDQKPGPSERSRATKSGSRGTERTVVMDKSKGVPVISVKTSGSKERASKSQDRKSASREKRSVVSFDKVKEPRKSRDSESHSRVRERSEREQRMQAQWEREERERLEIARERLAFQRQRLERERMERERLERERMHVEHERRREQERIHREREELRRQQELRYEQERRPAVRRPYDLDRRDDAYWPEAKRAALDERYHSDFNRQDRFHDFDHRDRGRYPDHSVDRREGSRSMMGEREGQHYPERHGGPERHGRDSRDGWGGYGSDKRMSEGRGLPPPPRRDWGDHGRREDDRSWQGTADGGMMDRDHKRWQGGERSMSGHSGPGHMMNRGGMSGRGSFAPGGASRGHPIPHGGMQGGFGGQSRGSRPSDARFTRRY

24) >gi|289577080|ref|NP_001409.3| eukaryotic translation initiation factor 4 gamma 2 isoform 1 [Homo sapiens] (102.3 kD)

MESAIAEGGASRFSASSGGGGSRGAPQHYPKTAGNSEFLGKTPGQNAQKWIPARSTRRDDNSAANNSANEKERHDAIFRKVRGILNKLTPEKFDKLCLELLNVGVESKLILKGVILLIVDKALEEPKYSSLYAQLCLRLAEDAPNFDGPAAEGQPGQKQSTTFRRLLISKLQDEFENRTRNVDVYDKRENPLLPEEEEQRAIAKIKMLGNIKFIGELGKLDLIHESILHKCIKTLLEKKKRVQLKDMGEDLECLCQIMRTVGPRLDHERAKSLMDQYFARMCSLMLSKELPARIRFLLQDTVELREHHWVPRKAFLDNGPKTINQIRQDAVKDLGVFIPAPMAQGMRSDFFLEGPFMPPRMKMDRDPLGGLADMFGQMPGSGIGTGPGVIQDRFSPTMGRHRSNQLFNGHGGHIMPPTQSQFGEMGGKFMKSQGLSQLYHNQSQGLLSQLQGQSKDMPPRFSKKGQLNADEISLRPAQSFLMNKNQVPKLQPQITMIPPSAQPPRTQTPPLGQTPQLGLKTNPPLIQEKPAKTSKKPPPSKEELLKLTETVVTEYLNSGNANEAVNGVREMRAPKHFLPEMLSKVIILSLDRSDEDKEKASSLISLLKQEGIATSDNFMQAFLNVLDQCPKLEVDIPLVKSYLAQFAARAIISELVSISELAQPLESGTHFPLFLLCLQQLAKLQDREWLTELFQQSKVNMQKMLPEIDQNKDRMLEILEGKGLSFLFPLLKLEKELLKQIKLDPSPQTIYKWIKDNISPKLHVDKGFVNILMTSFLQYISSEVNPPSDETDSSSAPSKEQLEQEKQLLLSFKPVMQKFLHDHVDLQVSALYALQVHCYNSNFPKGMLLRFFVHFYDMEIIEEEAFLAWKEDITQEFPGKGKALFQVNQWLTWLETAEEEESEEEAD

25) >gi|27477136|ref|NP_064504.2| zinc finger CCCH-type antiviral protein 1 isoform 1 [Homo sapiens] (101.4 kD)

MADPEVCCFITKILCAHGGRMALDALLQEIALSEPQLCEVLQVAGPDRFVVLETGGEAGITRSVVATTRARVCRRKYCQRPCDNLHLCKLNLLGRCNYSQSERNLCKYSHEVLSEENFKVLKNHELSGLNKEELAVLLLQSDPFFMPEICKSYKGEGRQQICNQQPPCSRLHICDHFTRGNCRFPNCLRSHNLMDRKVLAIMREHGLNPDVVQNIQDICNSKHMQKNPPGPRAPSSHRRNMAYRARSKSRDRFFQGSQEFLASASASAERSCTPSPDQISHRASLEDAPVDDLTRKFTYLGSQDRARPPSGSSKATDLGGTSQAGTSQRFLENGSQEDLLHGNPGSTYLASNSTSAPNWKSLTSWTNDQGARRKTVFSPTLPAARSSLGSLQTPEAVTTRKGTGLLSSDYRIINGKSGTQDIQPGPLFNNNADGVATDITSTRSLNYKSTSSGHREISSPRIQDAGPASRDVQATGRIADDADPRVALVNDSLSDVTSTTSSRVDDHDSEEICLDHLCKGCPLNGSCSKVHFHLPYRWQMLIGKTWTDFEHMETIEKGYCNPGIHLCSVGSYTINFRVMSCDSFPIRRLSTPSSVTKPANSVFTTKWIWYWKNESGTWIQYGEEKDKRKNSNVDSSYLESLYQSCPRGVVPFQAGSRNYELSFQGMIQTNIASKTQKDVIRRPTFVPQWYVQQMKRGPDHQPAKTSSVSLTATFRPQEDFCFLSSKKYKLSEIHHLHPEYVRVSEHFKASMKNFKIEKIKKIENSELLDKFTWKKSQMKEEGKLLFYATSRAYVESICSNNFDSFLHETHENKYGKGIYFAKDAIYSHKNCPYDAKNVVMFVAQVLVGKFTEGNITYTSPPPQFDSCVDTRSNPSVFVIFQKDQVYPQYVIEYTEDKACVIS

26) >gi|21361863|ref|NP_149095.2| stonin-2 isoform 1 [Homo sapiens] (101.1 kD)

MTTLDHVIATHQSEWVSFNEEPPFPAHSQGGTEEHLPGLSSSPDQSESSSGENHVVDGGSQDHSHSEQDDSSEKMGLISEAASPPGSPEQPPPDLASAISNWVQFEDDTPWASTSPPHQETAETALPLTMPCWTCPSFDSLGRCPLTSESSWTTHSEDTSSPSFGCSYTDLQLINAEEQTSGQASGADSTDNSSSLQEDEEVEMEAISWQASSPAMNGHPAPPVTSARFPSWVTFDDNEVSCPLPPVTSPLKPNTPPSASVIPDVPYNSMGSFKKRDRPKSTLMNFSKVQKLDISSLNRTPSVTEASPWRATNPFLNETLQDVQPSPINPFSAFFEEQERRSQNSSISSTTGKSQRDSLIVIYQDAISFDDSSKTQSHSDAVEKLKQLQIDDPDHFGSATLPDDDPVAWIELDAHPPGSARSQPRDGWPMMLRIPEKKNIMSSRHWGPIFVKLTDTGYLQLYYEQGLEKPFREFKLEICHEISEPRLQNYDENGRIHSLRIDRVTYKEKKKYQPKPAVAHTAEREQVIKLGTTNYDDFLSFIHAVQDRLMDLPVLSMDLSTVGLNYLEEEITVDVRDEFSGIVSKGDNQILQHHVLTRIHILSFLSGLAECRLGLNDILVKGNEIVLRQDIMPTTTTKWIKLHECRFHGCVDEDVFHNSRVILFNPLDACRFELMRFRTVFAEKTLPFTLRTATSVNGAEVEVQSWLRMSTGFSANRDPLTQVPCENVMIRYPVPSEWVKNFRRESVLGEKSLKAKVNRGASFGSTSVSGSEPVMRVTLGTAKYEHAFNSIVWRINRLPDKNSASGHPHCFFCHLELGSDREVPSRFANHVNVEFSMPTTSASKASVRSISVEDKTDVRKWVNYSAHYSYQVALGSIWLMLPTPFVHPTTLPLLFLLAMLTMFAW

27) >gi|7706607|ref|NP_055134.2| inner nuclear membrane protein Man1 isoform 1 [Homo sapiens] (99.9 kD)

MAAAAASAPQQLSDEELFSQLRRYGLSPGPVTESTRPVYLKKLKKLREEEQQQHRSGGRGNKTRNSNNNNTAAATVAAAGPAAAAAAGMGVRPVSGDLSYLRTPGGLCRISASGPESLLGGPGGASAAPAAGSKVLLGFSSDESDVEASPRDQAGGGGRKDRASLQYRGLKAPPAPLAASEVTNSNSAERRKPHSWWGARRPAGPELQTPPGKDGAVEDEEGEGEDGEERDPETEEPLWASRTVNGSRLVPYSCRENYSDSEEEDDDDVASSRQVLKDDSLSRHRPRRTHSKPLPPLTAKSAGGRLETSVQGGGGLAMNDRAAAAGSLDRSRNLEEAAAAEQGGGCDQVDSSPVPRYRVNAKKLTPLLPPPLTDMDSTLDSSTGSLLKTNNHIGGGAFSVDSPRIYSNSLPPSAAVAASSSLRINHANHTGSNHTYLKNTYNKPKLSEPEEELLQQFKREEVSPTGSFSAHYLSMFLLTAACLFFLILGLTYLGMRGTGVSEDGELSIENPFGETFGKIQESEKTLMMNTLYKLHDRLAQLAGDHECGSSSQRTLSVQEAAAYLKDLGPEYEGIFNTSLQWILENGKDVGIRCVGFGPEEELTNITDVQFLQSTRPLMSFWCRFRRAFVTVTHRLLLLCLGVVMVCVVLRYMKYRWTKEEEETRQMYDMVVKIIDVLRSHNEACQENKDLQPYMPIPHVRDSLIQPHDRKKMKKVWDRAVDFLAANESRVRTETRRIGGADFLVWRWIQPSASCDKILVIPSKVWQGQAFHLDRRNSPPNSLTPCLKIRNMFDPVMEIGDQWHLAIQEAILEKCSDNDGIVHIAVDKNSREGCVYVKCLSPEYAGKAFKALHGSWFDGKLVTVKYLRLDRYHHRFPQALTSNTPLKPSNKHMNSMSHLRLRTGLTNSQGSS

28) >gi|21536466|ref|NP_068713.2| tyrosine-protein kinase receptor UFO isoform 1 precursor [Homo sapiens] (98.3 kD)

MAWRCPRMGRVPLAWCLALCGWACMAPRGTQAEESPFVGNPGNITGARGLTGTLRCQLQVQGEPPEVHWLRDGQILELADSTQTQVPLGEDEQDDWIVVSQLRITSLQLSDTGQYQCLVFLGHQTFVSQPGYVGLEGLPYFLEEPEDRTVAANTPFNLSCQAQGPPEPVDLLWLQDAVPLATAPGHGPQRSLHVPGLNKTSSFSCEAHNAKGVTTSRTATITVLPQQPRNLHLVSRQPTELEVAWTPGLSGIYPLTHCTLQAVLSDDGMGIQAGEPDPPEEPLTSQASVPPHQLRLGSLHPHTPYHIRVACTSSQGPSSWTHWLPVETPEGVPLGPPENISATRNGSQAFVHWQEPRAPLQGTLLGYRLAYQGQDTPEVLMDIGLRQEVTLELQGDGSVSNLTVCVAAYTAAGDGPWSLPVPLEAWRPGQAQPVHQLVKEPSTPAFSWPWWYVLLGAVVAAACVLILALFLVHRRKKETRYGEVFEPTVERGELVVRYRVRKSYSRRTTEATLNSLGISEELKEKLRDVMVDRHKVALGKTLGEGEFGAVMEGQLNQDDSILKVAVKTMKIAICTRSELEDFLSEAVCMKEFDHPNVMRLIGVCFQGSERESFPAPVVILPFMKHGDLHSFLLYSRLGDQPVYLPTQMLVKFMADIASGMEYLSTKRFIHRDLAARNCMLNENMSVCVADFGLSKKIYNGDYYRQGRIAKMPVKWIAIESLADRVYTSKSDVWSFGVTMWEIATRGQTPYPGVENSEIYDYLRQGNRLKQPADCLDGLYALMSRCWELNPQDRPSFTELREDLENTLKALPPAQEPDEILYVNMDEGGGYPEPPGAAGGADPPTQPDPKDSCSCLTAAEVHPAGRYVLCPSTTPSPAQPADRGSPAAPGQEDGA

29) >gi|33667117|ref|NP_891554.1| MICAL-like protein 2 [Homo sapiens] (97.4 kD)

MAAIRALQQWCRQQCEGYRDVNICNMTTSFRDGLAFCAILHRHRPDLINFSALKKENIYENNKLAFRVAEEHLGIPALLDAEDMVALKVPDRLSILTYVSQYYNYFHGRSPIGGMAGVKRASEDSEEEPSGKKAPVQAAKLPSPAPARKPPLSPAQTNPVVQRRNEGAGGPPPKTDQALAGSLVSSTCGVCGKHVHLVQRHLADGRLYHRSCFRCKQCSCTLHSGAYKATGEPGTFVCTSHLPAAASASPKLTGLVPRQPGAMGVDSRTSCSPQKAQEANKARPSAWEPAAGNSPARASVPAAPNPAATSATSVHVRSPARPSESRLAPTPTEGKVRPRVTNSSPMGWSSAAPCTAAAASHPAVPPSAPDPRPATPQGGGAPRVAAPQTTLSSSSTSAATVDPPAWTPSASRTQQARNKFFQTSAVPPGTSLSGRGPTPSLVLSKDSSKEQARNFLKQALSALEEAGAPAPGRPSPATAAVPSSQPKTEAPQASPLAKPLQSSSPRVLGLPSRMEPPAPLSTSSTSQASALPPAGRRNLAESSGVGRVGAGSRPKPEAPMAKGKSTTLTQDMSTSLQEGQEDGPAGWRANLKPVDRRSPAERTLKPKEPRALAEPRAGEAPRKVSGSFAGSVHITLTPVRPDRTPRPASPGPSLPARSPSPPRRRRLAVPASLDVCDNWLRPEPPGQEARVQSWKEEEKKPHLQGKPGRPLSPANVPALPGETVTSPVRLHPDYLSPEEIQRQLQDIERRLDALELRGVELEKRLRAAEGDDAEDSLMVDWFWLIHEKQLLLRQESELMYKSKAQRLEEQQLDIEGELRRLMAKPEALKSLQERRREQELLEQYVSTVNDRSDIVDSLDEDRLREQEEDQMLRDMIEKLGLQRKKSKFRLSKIWSPKSKSSPSQ

30) >gi|19923142|ref|NP_002256.2| importin subunit beta-1 isoform 1 [Homo sapiens] (97.1 kD)

MELITILEKTVSPDRLELEAAQKFLERAAVENLPTFLVELSRVLANPGNSQVARVAAGLQIKNSLTSKDPDIKAQYQQRWLAIDANARREVKNYVLQTLGTETYRPSSASQCVAGIACAEIPVNQWPELIPQLVANVTNPNSTEHMKESTLEAIGYICQDIDPEQLQDKSNEILTAIIQGMRKEEPSNNVKLAATNALLNSLEFTKANFDKESERHFIMQVVCEATQCPDTRVRVAALQNLVKIMSLYYQYMETYMGPALFAITIEAMKSDIDEVALQGIEFWSNVCDEEMDLAIEASEAAEQGRPPEHTSKFYAKGALQYLVPILTQTLTKQDENDDDDDWNPCKAAGVCLMLLATCCEDDIVPHVLPFIKEHIKNPDWRYRDAAVMAFGCILEGPEPSQLKPLVIQAMPTLIELMKDPSVVVRDTAAWTVGRICELLPEAAINDVYLAPLLQCLIEGLSAEPRVASNVCWAFSSLAEAAYEAADVADDQEEPATYCLSSSFELIVQKLLETTDRPDGHQNNLRSSAYESLMEIVKNSAKDCYPAVQKTTLVIMERLQQVLQMESHIQSTSDRIQFNDLQSLLCATLQNVLRKVQHQDALQISDVVMASLLRMFQSTAGSGGVQEDALMAVSTLVEVLGGEFLKYMEAFKPFLGIGLKNYAEYQVCLAAVGLVGDLCRALQSNIIPFCDEVMQLLLENLGNENVHRSVKPQILSVFGDIALAIGGEFKKYLEVVLNTLQQASQAQVDKSDYDMVDYLNELRESCLEAYTGIVQGLKGDQENVHPDVMLVQPRVEFILSFIDHIAGDEDHTDGVVACAAGLIGDLCTAFGKDVLKLVEARPMIHELLTEGRRSKTNKAKTLATWATKELRKLKNQA

31) >gi|4503483|ref|NP_001952.1| elongation factor 2 [Homo sapiens] (95.3 kD)

MVNFTVDQIRAIMDKKANIRNMSVIAHVDHGKSTLTDSLVCKAGIIASARAGETRFTDTRKDEQERCITIKSTAISLFYELSENDLNFIKQSKDGAGFLINLIDSPGHVDFSSEVTAALRVTDGALVVVDCVSGVCVQTETVLRQAIAERIKPVLMMNKMDRALLELQLEPEELYQTFQRIVENVNVIISTYGEGESGPMGNIMIDPVLGTVGFGSGLHGWAFTLKQFAEMYVAKFAAKGEGQLGPAERAKKVEDMMKKLWGDRYFDPANGKFSKSATSPEGKKLPRTFCQLILDPIFKVFDAIMNFKKEETAKLIEKLDIKLDSEDKDKEGKPLLKAVMRRWLPAGDALLQMITIHLPSPVTAQKYRCELLYEGPPDDEAAMGIKSCDPKGPLMMYISKMVPTSDKGRFYAFGRVFSGLVSTGLKVRIMGPNYTPGKKEDLYLKPIQRTILMMGRYVEPIEDVPCGNIVGLVGVDQFLVKTGTITTFEHAHNMRVMKFSVSPVVRVAVEAKNPADLPKLVEGLKRLAKSDPMVQCIIEESGEHIIAGAGELHLEICLKDLEEDHACIPIKKSDPVVSYRETVSEESNVLCLSKSPNKHNRLYMKARPFPDGLAEDIDKGEVSARQELKQRARYLAEKYEWDVAEARKIWCFGPDGTGPNILTDITKGVQYLNEIKDSVVAGFQWATKEGALCEENMRGVRFDVHDVTLHADAIHRGGGQIIPTARRCLYASVLTAQPRLMEPIYLVEIQCPEQVVGGIYGVLNRKRGHVFEESQVAGTPMFVVKAYLPVNESFGFTADLRSNTGGQAFPQCVFDHWQILPGDPFDNSSRPSQVVAETRKRKGLKEGIPALDNFLDKL

32) >gi|4507677|ref|NP_003290.1| endoplasmin precursor [Homo sapiens] (92.4 kD)

MRALWVLGLCCVLLTFGSVRADDEVDVDGTVEEDLGKSREGSRTDDEVVQREEEAIQLDGLNASQIRELREKSEKFAFQAEVNRMMKLIINSLYKNKEIFLRELISNASDALDKIRLISLTDENALSGNEELTVKIKCDKEKNLLHVTDTGVGMTREELVKNLGTIAKSGTSEFLNKMTEAQEDGQSTSELIGQFGVGFYSAFLVADKVIVTSKHNNDTQHIWESDSNEFSVIADPRGNTLGRGTTITLVLKEEASDYLELDTIKNLVKKYSQFINFPIYVWSSKTETVEEPMEEEEAAKEEKEESDDEAAVEEEEEEKKPKTKKVEKTVWDWELMNDIKPIWQRPSKEVEEDEYKAFYKSFSKESDDPMAYIHFTAEGEVTFKSILFVPTSAPRGLFDEYGSKKSDYIKLYVRRVFITDDFHDMMPKYLNFVKGVVDSDDLPLNVSRETLQQHKLLKVIRKKLVRKTLDMIKKIADDKYNDTFWKEFGTNIKLGVIEDHSNRTRLAKLLRFQSSHHPTDITSLDQYVERMKEKQDKIYFMAGSSRKEAESSPFVERLLKKGYEVIYLTEPVDEYCIQALPEFDGKRFQNVAKEGVKFDESEKTKESREAVEKEFEPLLNWMKDKALKDKIEKAVVSQRLTESPCALVASQYGWSGNMERIMKAQAYQTGKDISTNYYASQKKTFEINPRHPLIRDMLRRIKEDEDDKTVLDLAVVLFETATLRSGYLLPDTKAYGDRIERMLRLSLNIDPDAKVEEEPEEEPEETAEDT

TEDTEQDEDEEMDVGTDEEEETAKESTAEKDEL

33) >gi|17978491|ref|NP_510966.1| CD97 antigen isoform 1 preproprotein [Homo sapiens] (91.8 kD)

MGGRVFLAFCVWLTLPGAETQDSRGCARWCPQNSSCVNATACRCNPGFSSFSEIITTPTETCDDINECATPSKVSCGKFSDCWNTEGSYDCVCSPGYEPVSGAKTFKNESENTCQDVDECQQNPRLCKSYGTCVNTLGSYTCQCLPGFKFIPEDPKVCTDVNECTSGQNPCHSSTHCLNNVGSYQCRCRPGWQPIPGSPNGPNNTVCEDVDECSSGQHQCDSSTVCFNTVGSYSCRCRPGWKPRHGIPNNQKDTVCEDMTFSTWTPPPGVHSQTLSRFFDKVQDLGRDSKTSSAEVTIQNVIKLVDELMEAPGDVEALAPPVRHLIATQLLSNLEDIMRILAKSLPKGPFTYISPSNTELTLMIQERGDKNVTMGQSSARMKLNWAVAAGAEDPGPAVAGILSIQNMTTLLANASLNLHSKKQAELEEIYESSIRGVQLRRLSAVNSIFLSHNNTKELNSPILFAFSHLESSDGEAGRDPPAKDVMPGPRQELLCAFWKSDSDRGGHWATEGCQVLGSKNGSTTCQCSHLSSFAILMAHYDVEDWKLTLITRVGLALSLFCLLLCILTFLLVRPIQGSRTTIHLHLCICLFVGSTIFLAGIENEGGQVGLRCRLVAGLLHYCFLAAFCWMSLEGLELYFLVVRVFQGQGLSTRWLCLIGYGVPLLIVGVSAAIYSKGYGRPRYCWLDFEQGFLWSFLGPVTFIILCNAVIFVTTVWKLTQKFSEINPDMKKLKKARALTITAIAQLFLLGCTWVFGLFIFDDRSLVLTYVFTILNCLQGAFLYLLHCLLNKKVREEYRKWACLVAGGSKYSEFTSTTSGTGHNQTRALRASESGI

34) >gi|5032087|ref|NP_005868.1| splicing factor 3A subunit 1 isoform 1 [Homo sapiens] (88.8 kD)

MPAGPVQAVPPPPPVPTEPKQPTEEEASSKEDSAPSKPVVGIIYPPPEVRNIVDKTASFVARNGPEFEARIRQNEINNPKFNFLNPNDPYHAYYRHKVSEFKEGKAQEPSAAIPKVMQQQQQTTQQQLPQKVQAQVIQETIVPKEPPPEFEFIADPPSISAFDLDVVKLTAQFVARNGRQFLTQLMQKEQRNYQFDFLRPQHSLFNYFTKLVEQYTKILIPPKGLFSKLKKEAENPREVLDQVCYRVEWAKFQERERKKEEEEKEKERVAYAQIDWHDFVVVETVDFQPNEQGNFPPPTTPEELGARILIQERYEKFGESEEVEMEVESDEEDDKQEKAEEPPSQLDQDTQVQDMDEGSDDEEEGQKVPPPPETPMPPPLPPTPDQVIVRKDYDPKASKPLPPAPAPDEYLVSPITGEKIPASKMQEHMRIGLLDPRWLEQRDRSIREKQSDDEVYAPGLDIESSLKQLAERRTDIFGVEETAIGKKIGEEEIQKPEEKVTWDGHSGSMARTQQAAQANITLQEQIEAIHKAKGLVPEDDTKEKIGPSKPNEIPQQPPPPSSATNIPSSAPPITSVPRPPTMPPPVRTTVVSAVPVMPRPPMASVVRLPPGSVIAPMPPIIHAPRINVVPMPPSAPPIMAPRPPPMIVPTAFVPAPPVAPVPAPAPMPPVHPPPPMEDEPTSKKLKTEDSLMPEEEFLRRNKGPVSIKVQVPNMQDKTEWKLNGQVLVFTLPLTDQVSVIKVKIHEATGMPAGKQKLQYEGIFIKDSNSLAYYNMANGAVIHLALKERGGRKK

35) >gi|19743813|ref|NP_002202.2| integrin beta-1 isoform 1A precursor [Homo sapiens] (88.4 kD)

MNLQPIFWIGLISSVCCVFAQTDENRCLKANAKSCGECIQAGPNCGWCTNSTFLQEGMPTSARCDDLEALKKKGCPPDDIENPRGSKDIKKNKNVTNRSKGTAEKLKPEDITQIQPQQLVLRLRSGEPQTFTLKFKRAEDYPIDLYYLMDLSYSMKDDLENVKSLGTDLMNEMRRITSDFRIGFGSFVEKTVMPYISTTPAKLRNPCTSEQNCTSPFSYKNVLSLTNKGEVFNELVGKQRISGNLDSPEGGFDAIMQVAVCGSLIGWRNVTRLLVFSTDAGFHFAGDGKLGGIVLPNDGQCHLENNMYTMSHYYDYPSIAHLVQKLSENNIQTIFAVTEEFQPVYKELKNLIPKSAVGTLSANSSNVIQLIIDAYNSLSSEVILENGKLSEGVTISYKSYCKNGVNGTGENGRKCSNISIGDEVQFEISITSNKCPKKDSDSFKIRPLGFTEEVEVILQYICECECQSEGIPESPKCHEGNGTFECGACRCNEGRVGRHCECSTDEVNSEDMDAYCRKENSSEICSNNGECVCGQCVCRKRDNTNEIYSGKFCECDNFNCDRSNGLICGGNGVCKCRVCECNPNYTGSACDCSLDTSTCEASNGQICNGRGICECGVCKCTDPKFQGQTCEMCQTCLGVCAEHKECVQCRAFNKGEKKDTCTQECSYFNITKVESRDKLPQPVQPDPVSHCKEKDVDDCWFYFTYSVNGNNEVMVHVVENPECPTGPDIIPIVAGVVAGIVLIGLALLLIWKLLMIIHDRREFAKFEKEKMNAKWDTGENPIYKSAVTTVVNPKYEGK

36) >gi|72534670|ref|NP_001026859.1| phospholipase A-2-activating protein [Homo sapiens] (87.1 kD)

MTSGATRYRLSCSLRGHELDVRGLVCCAYPPGAFVSVSRDRTTRLWAPDSPNRSFTEMHCMSGHSNFVSCVCIIPSSDIYPHGLIATGGNDHNICIFSLDSPMPLYILKGHKNTVCSLSSGKFGTLLSGSWDTTAKVWLNDKCMMTLQGHTAAVWAVKILPEQGLMLTGSADKTVKLWKAGRCERTFSGHEDCVRGLAILSETEFLSCANDASIRRWQITGECLEVYYGHTNYIYSISVFPNCRDFVTTAEDRSLRIWKHGECAQTIRLPAQSIWCCCVLDNGDIVVGASDGIIRVFTESEDRTASAEEIKAFEKELSHATIDSKTGDLGDINAEQLPGREHLNEPGTREGQTRLIRDGEKVEAYQWSVSEGRWIKIGDVVGSSGANQQTSGKVLYEGKEFDYVFSIDVNEGGPSYKLPYNTSDDPWLTAYNFLQKNDLNPMFLDQVAKFIIDNTKGQMLGLGNPSFSDPFTGGGRYVPGSSGSSNTLPTADPFTGAGRYVPGSASMGTTMAGVDPFTGNSAYRSAASKTMNIYFPKKEAVTFDQANPTQILGKLKELNGTAPEEKKLTEDDLILLEKILSLICNSSSEKPTVQQLQILWKAINCPEDIVFPALDILRLSIKHPSVNENFCNEKEGAQFSSHLINLLNPKGKPANQLLALRTFCNCFVGQAGQKLMMSQRESLMSHAIELKSGSNKNIHIALATLALNYSVCFHKDHNIEGKAQCLSLISTILEVVQDLEATFRLLVALGTLISDDSNAVQLAKSLGVDSQIKKYSSVSEPAKVSECCRFILNLL

37) >gi|14589866|ref|NP_004309.2| aspartyl/asparaginyl beta-hydroxylase isoform a [Homo sapiens] (85.8 kD)

MAQRKNAKSSGNSSSSGSGSGSTSAGSSSPGARRETKHGGHKNGRKGGLSGTSFFTWFMVIALLGVWTSVAVVWFDLVDYEEVLGKLGIYDADGDGDFDVDDAKVLLGLKERSTSEPAVPPEEAEPHTEPEEQVPVEAEPQNIEDEAKEQIQSLLHEMVHAEHVEGEDLQQEDGPTGEPQQEDDEFLMATDVDDRFETLEPEVSHEETEHSYHVEETVSQDCNQDMEEMMSEQENPDSSEPVVEDERLHHDTDDVTYQVYEEQAVYEPLENEGIEITEVTAPPEDNPVEDSQVIVEEVSIFPVEEQQEVPPETNRKTDDPEQKAKVKKKKPKLLNKFDKTIKAELDAAEKLRKRGKIEEAVNAFKELVRKYPQSPRARYGKAQCEDDLAEKRRSNEVLRGAIETYQEVASLPDVPADLLKLSLKRRSDRQQFLGHMRGSLLTLQRLVQLFPNDTSLKNDLGVGYLLIGDNDNAKKVYEEVLSVTPNDGFAKVHYGFILKAQNKIAESIPYLKEGIESGDPGTDDGRFYFHLGDAMQRVGNKEAYKWYELGHKRGHFASVWQRSLYNVNGLKAQPWWTPKETGYTELVKSLERNWKLIRDEGLAVMDKAKGLFLPEDENLREKGDWSQFTLWQQGRRNENACKGAPKTCTLLEKFPETTGCRRGQIKYSIMHPGTHVWPHTGPTNCRLRMHLGLVIPKEGCKIRCANETKTWEEGKVLIFDDSFEHEVWQDASSFRLIFIVDVWHPELTPQQRRSLPAI

38) >gi|7705373|ref|NP_057441.1| LIM domain and actin-binding protein 1 isoform 2 [Homo sapiens] (85.2 kD)

MESSPFNRRQWTSLSLRVTAKELSLVNKNKSSAIVEIFSKYQKAAEETNMEKKRSNTENLSQHFRKGTLTVLKKKWENPGLGAESHTDSLRNSSTEIRHRADHPPAEVTSHAASGAKADQEEQIHPRSRLRSPPEALVQGRYPHIKDGEDLKDHSTESKKMENCLGESRHEVEKSEISENTDASGKIEKYNVPLNRLKMMFEKGEPTQTKILRAQSRSASGRKISENSYSLDDLEIGPGQLSSSTFDSEKNESRRNLELPRLSETSIKDRMAKYQAAVSKQSSSTNYTNELKASGGEIKIHKMEQKENVPPGPEVCITHQEGEKISANENSLAVRSTPAEDDSRDSQVKSEVQQPVHPKPLSPDSRASSLSESSPPKAMKKFQAPARETCVECQKTVYPMERLLANQQVFHISCFRCSYCNNKLSLGTYASLHGRIYCKPHFNQLFKSKGNYDEGFGHRPHKDLWASKNENEEILERPAQLANARETPHSPGVEDAPIAKVGVLAASMEAKASSQQEKEDKPAETKKLRIAWPPPTELGSSGSALEEGIKMSKPKWPPEDEISKPEVPEDVDLDLKKLRRSSSLKERSRPFTVAASFQSTSVKSPKTVSPPIRKGWSMSEQSEESVGGRVAERKQVENAKASKKNGNVGKTTWQNKESKGETGKRSKEGHSLEMENENLVENGADSDEDDNSFLKQQSPQEPKSLNWSSFVDNTFAEEFTTQNQKSQDVELWEGEVVKELSVEEQIKRNRYYDEDEDEE

39) >gi|27262628|ref|NP_002473.2| nuclear autoantigenic sperm protein isoform 2 [Homo sapiens] (85.2 kD)

MAMESTATAAVAAELVSADKIEDVPAPSTSADKVESLDVDSEAKKLLGLGQKHLVMGDIPAAVNAFQEAASLLGKKYGETANECGEAFFFYGKSLLELARMENGVLGNALEGVHVEEEEGEKTEDESLVENNDNIDEEAREELREQVYDAMGEKEEAKKTEDKSLAKPETDKEQDSEMEKGGREDMDISKSAEEPQEKVDLTLDWLTETSEEAKGGAAPEGPNEAEVTSGKPEQEVPDAEEEKSVSGTDVQEECREKGGQEKQGEVIVSIEEKPKEVSEEQPVVTLEKQGTAVEVEAESLDPTVKPVDVGGDEPEEKVVTSENEAGKAVLEQLVGQEVPPAEESPEVTTEAAEASAVEAGSEVSEKPGQEAPVLPKDGAVNGPSVVGDQTPIEPQTSIERLTETKDGSGLEEKVRAKLVPSQEETKLSVEESEAAGDGVDTKVAQGATEKSPEDKVQIAANEETQEREEQMKEGEETEGSEEDDKENDKTEEMPNDSVLENKSLQENEEEEIGNLELAWDMLDLAKIIFKRQETKEAQLYAAQAHLKLGEVSVESENYVQAVEEFQSCLNLQEQYLEAHDRLLAETHYQLGLAYGYNSQYDEAVAQFSKSIEVIENRMAVLNEQVKEAEGSSAEYKKEIEELKELLPEIREKIEDAKESQRSGNVAELALKATLVESSTSGFTPGGGGSSVSMIASRKPTDGASSSNCVTDISHLVRKKRKPEEESPRKDDAKKAKQEPEVNGGSGDAVPSGNEVSENMEEEAENQAESR

AAVEGTVEAGATVESTAC

40) >gi|189458819|ref|NP_001121620.1| transferrin receptor protein 1 [Homo sapiens] (84.8 kD)

MMDQARSAFSNLFGGEPLSYTRFSLARQVDGDNSHVEMKLAVDEEENADNNTKANVTKPKRCSGSICYGTIAVIVFFLIGFMIGYLGYCKGVEPKTECERLAGTESPVREEPGEDFPAARRLYWDDLKRKLSEKLDSTDFTGTIKLLNENSYVPREAGSQKDENLALYVENQFREFKLSKVWRDQHFVKIQVKDSAQNSVIIVDKNGRLVYLVENPGGYVAYSKAATVTGKLVHANFGTKKDFEDLYTPVNGSIVIVRAGKITFAEKVANAESLNAIGVLIYMDQTKFPIVNAELSFFGHAHLGTGDPYTPGFPSFNHTQFPPSRSSGLPNIPVQTISRAAAEKLFGNMEGDCPSDWKTDSTCRMVTSESKNVKLTVSNVLKEIKILNIFGVIKGFVEPDHYVVVGAQRDAWGPGAAKSGVGTALLLKLAQMFSDMVLKDGFQPSRSIIFASWSAGDFGSVGATEWLEGYLSSLHLKAFTYINLDKAVLGTSNFKVSASPLLYTLIEKTMQNVKHPVTGQFLYQDSNWASKVEKLTLDNAAFPFLAYSGIPAVSFCFCEDTDYPYLGTTMDTYKELIERIPELNKVARAAAEVAGQFVIKLTHDVELNLDYERYNSQLLSFVRDLNQYRADIKEMGLSLQWLYSARGDFFRATSRLTTDFGNAEKTDRFVMKKLNDRVMRVEYHFLSPYVSPKESPFRHVFWGSGSHTLPALLENLKLRKQNNGAFNETLFRNQLALATWTIQGAANALSGDVWDIDNEF

**Figure legend**: These are 40 largest proteins identified from the 48-kD stripe (MB231). The red underlined sequences are the LC-MS/MS identified peptide fragments that are unique to the protein, while the green underlined sequences are the LC-MS/MS identified peptide fragments that are not unique to the protein but can also appear in other proteins.

**The 20 smallest proteins identified in the 55-kD stripe (MB231)**

1) >gi|4507131|ref|NP_003086.1| small nuclear ribonucleoprotein F [Homo sapiens] (9.7 kD)

MSLPLNPKPFLNGLTGKPVMVKLKWGMEYKGYLVSVDGYMNMQLANTEEYIDGALSGHLGEVLIRCNNVLYIRGVEEEEEDGEMRE

2) >gi|4504523|ref|NP_002148.1| 10 kDa heat shock protein, mitochondrial [Homo sapiens] (10.9 kD)

MAGQAFRKFLPLFDRVLVERSAAETVTKGGIMLPEKSQGKVLQATVVAVGSGSKGKGGEIQPVSVKVGDKVLLPEYGGTKVVLDDKDYFLFRDGDILGKYVD

3) >gi|4504301|ref|NP_003529.1| histone H4 [Homo sapiens] (11.4 kD)

MSGRGKGGKGLGKGGAKRHRKVLRDNIQGITKPAIRRLARRGGVKRISGLIYEETRGVLKVFLENVIRDAVTYTEHAKRKTVTAMDVVYALKRQGRTLYGFGG

4) >gi|11128019|ref|NP_061820.1| cytochrome c [Homo sapiens] (11.7 kD)

MGDVEKGKKIFIMKCSQCHTVEKGGKHKTGPNLHGLFGRKTGQAPGYSYTAANKNKGIIWGEDTLMEYLENPKKYIPGTKMIFVGIKKKEERADLIAYLKKATNE

5) >gi|4759160|ref|NP_004166.1| small nuclear ribonucleoprotein Sm D3 [Homo sapiens] (13.9 kD)

MSIGVPIKVLHEAEGHIVTCETNTGEVYRGKLIEAEDNMNCQMSNITVTYRDGRVAQLEQVYIRGSKIRFLILPDMLKNAPMLKSMKNKNQGSGAGRGKAAILKAQVAARGRGRGMGRGNIFQKRR

6) >gi|18105048|ref|NP_542160.1| histone H2B type 1-K [Homo sapiens] (13.9 kD)

MPEPAKSAPAPKKGSKKAVTKAQKKDGKKRKRSRKESYSVYVYKVLKQVHPDTGISSKAMGIMNSFVNDIFERIAGEASRLAHYNKRSTITSREIQTAVRLLLPGELAKHAVSEGTKAVTKYTSAK

7) >gi|18105045|ref|NP_542163.1| histone H2A type 1-H [Homo sapiens] (13.9 kD)

MSGRGKQGGKARAKAKTRSSRAGLQFPVGRVHRLLRKGNYAERVGAGAPVYLAAVLEYLTAEILELAGNAARDNKKTRIIPRHLQLAIRNDEELNKLLGKVTIAQGGVLPNIQAVLLPKKTESHHKAK

8) >gi|4504981|ref|NP_002296.1| galectin-1 [Homo sapiens] (14.7 kD)

MACGLVASNLNLKPGECLRVRGEVAPDAKSFVLNLGKDSNNLCLHFNPRFNAHGDANTIVCNSKDGGAWGTEQREAVFPFQPGSVAEVCITFDQANLTVKLPDGYEFKFPNRLNLEAINYMAADGDFKIKCVAFD

9) >gi|71772415|ref|NP_001025180.1| 40S ribosomal protein S15a [Homo sapiens] (14.8 kD)

MVRMNVLADALKSINNAEKRGKRQVLIRPCSKVIVRFLTVMMKHGYIGEFEIIDDHRAGKIVVNLTGRLNKCGVISPRFDVQLKDLEKWQNNLLPSRQFGFIVLTTSAGIMDHEEARRKHTGGKILGFFF

10) >gi|4826898|ref|NP_005013.1| profilin-1 [Homo sapiens] (15 kD)

MAGWNAYIDNLMADGTCQDAAIVGYKDSPSVWAAVPGKTFVNITPAEVGVLVGKDRSSFYVNGLTLGGQKCSVIRDSLLQDGEFSMDLRTKSTGGAPTFNVTVTKTDKTLVLLMGKEGVHGGLINKKCYEMASHLRRSQY

11) >gi|4506703|ref|NP_001017.1| 40S ribosomal protein S24 isoform c [Homo sapiens] (15.4 kD)

MNDTVTIRTRKFMTNRLLQRKQMVIDVLHPGKATVPKTEIREKLAKMYKTTPDVIFVFGFRTHFGGGKTTGFGMIYDSLDYAKKNEPKHRLARHGLYEKKKTSRKQRKERKNRMKKVRGTAKANVGAGKKPKE

12) >gi|4506701|ref|NP_001016.1| 40S ribosomal protein S23 [Homo sapiens] (15.8 kD)

MGKCRGLRTARKLRSHRRDQKWHDKQYKKAHLGTALKANPFGGASHAKGIVLEKVGVEAKQPNSAIRKCVRVQLIKNGKKITAFVPNDGCLNFIEENDEVLVAGFGRKGHAVGDIPGVRFKVVKVANVSLLALYKGKKERPRS

13) >gi|4506691|ref|NP_001011.1| 40S ribosomal protein S16 [Homo sapiens] (16.4 kD)

MPSKGPLQSVQVFGRKKTATAVAHCKRGNGLIKVNGRPLEMIEPRTLQYKLLEPVLLLGKERFAGVDIRVRVKGGGHVAQIYAIRQSISKALVAYYQKYVDEASKKEIKDILIQYDRTLLVADPRRCESKKFGGPGARARYQKSYR

14) >gi|4826774|ref|NP_005092.1| ubiquitin-like protein ISG15 precursor [Homo sapiens] (17.9 kD)

MGWDLTVKMLAGNEFQVSLSSSMSVSELKAQITQKIGVHAFQQRLAVHPSGVALQDRVPLASQGLGPGSTVLLVVDKCDEPLSILVRNNKGRSSTYEVRLTQTVAHLKQQVSGLEGVQDDLFWLTFEGKPLEDQLPLGEYGLKPLSTVFMNLRLRGGGTEPGGRS

15) >gi|10863927|ref|NP_066953.1| peptidyl-prolyl cis-trans isomerase A [Homo sapiens] (18 kD)

MVNPTVFFDIAVDGEPLGRVSFELFADKVPKTAENFRALSTGEKGFGYKGSCFHRIIPGFMCQGGDFTRHNGTGGKSIYGEKFEDENFILKHTGPGILSMANAGPNTNGSQFFICTAKTEWLDGKHVVFGKVKEGMNIVEAMERFGSRNGKTSKKITIADCGQLE

16) >gi|5031635|ref|NP_005498.1| cofilin-1 [Homo sapiens] (18.5 kD)

MASGVAVSDGVIKVFNDMKVRKSSTPEEVKKRKKAVLFCLSEDKKNIILEEGKEILVGDVGQTVDDPYATFVKMLPDKDCRYALYDATYETKESKKEDLVFIFWAPESAPLKSKMIYASSKDAIKKKLTGIKHELQANCYEEVKDRCTLAEKLGGSAVISLEGKPL

17) >gi|4506679|ref|NP_001005.1| 40S ribosomal protein S10 [Homo sapiens] (18.9 kD)

MLMPKKNRIAIYELLFKEGVMVAKKDVHMPKHPELADKNVPNLHVMKAMQSLKSRGYVKEQFAWRHFYWYLTNEGIQYLRDYLHLPPEIVPATLRRSRPETGRPRPKGLEGERPARLTRGEADRDTYRRSAVPPGADKKAEAGAGSATEFQFRGGFGRGRGQPPQ

18) >gi|5031595|ref|NP_005709.1| actin-related protein 2/3 complex subunit 4 isoform a [Homo sapiens] (19.7 kD)

MTATLRPYLSAVRATLQAALCLENFSSQVVERHNKPEVEVRSSKELLLQPVTISRNEKEKVLIEGSINSVRVSIAVKQADEIEKILCHKFMRFMMMRAENFFILRRKPVEGYDISFLITNFHTEQMYKHKLVDFVIHFMEEIDKEISEMKLSVNARARIVAEEFLKNF

19) >gi|15431290|ref|NP_000966.2| 60S ribosomal protein L11 isoform 1 [Homo sapiens] (20.2 kD)

MAQDQGEKENPMRELRIRKLCLNICVGESGDRLTRAAKVLEQLTGQTPVFSKARYTVRSFGIRRNEKIAVHCTVRGAKAEEILEKGLKVREYELRKNNFSDTGNFGFGIQEHIDLGIKYDPSIGIYGLDFYVVLGRPGFSIADKKRRTGCIGAKHRISKEEAMRWFQQKYDGIILPGK

20) >gi|4502205|ref|NP_001651.1| ADP-ribosylation factor 4 [Homo sapiens] (20.5 kD)

MGLTISSLFSRLFGKKQMRILMVGLDAAGKTTILYKLKLGEIVTTIPTIGFNVETVEYKNICFTVWDVGGQDRIRPLWKHYFQNTQGLIFVVDSNDRERIQEVADELQKMLLVDELRDAVLLLFANKQDLPNAMAISEMTDKLGLQSLRNRTWYVQATCATQGTGLYEGLDWLSNELSKR

**Figure legend**: These are 20 smallest proteins identified in the 55-kD stripe (MB231). The red underlined sequences are the LC-MS/MS identified peptide fragments that are unique to the protein, while the green underlined sequences are the LC-MS/MS identified peptide fragments that are not unique to the protein but can also appear in other proteins.

**The 40 largest proteins in the 55-kD stripe (MB231)**

1) >gi|61743954|ref|NP_001611.1| neuroblast differentiation-associated protein AHNAK isoform 1 [Homo sapiens] (628.7 kD)

MEKEETTRELLLPNWQGSGSHGLTIAQRDDGVFVQEVTQNSPAARTGVVKEGDQIVGATIYFDNLQSGEVTQLLNTMGHHTVGLKLHRKGDRSPEPGQTWTREVFSSCSSEVVLSGDDEEYQRIYTTKIKPRLKSEDGVEGDLGETQSRTITVTRRVTAYTVDVTGREGAKDIDISSPEFKIKIPRHELTEISNVDVETQSGKTVIRLPSGSGAASPTGSAVDIRAGAISASGPELQGAGHSKLQVTMPGIKVGGSGVNVNAKGLDLGGRGGVQVPAVDISSSLGGRAVEVQGPSLESGDHGKIKFPTMKVPKFGVSTGREGQTPKAGLRVSAPEVSVGHKGGKPGLTIQAPQLEVSVPSANIEGLEGKLKGPQITGPSLEGDLGLKGAKPQGHIGVDASAPQIGGSITGPSVEVQAPDIDVQGPGSKLNVPKMKVPKFSVSGAKGEETGIDVTLPTGEVTVPGVSGDVSLPEIATGGLEGKMKGTKVKTPEMIIQKPKISMQDVDLSLGSPKLKGDIKVSAPGVQGDVKGPQVALKGSRVDIETPNLEGTLTGPRLGSPSGKTGTCRISMSEVDLNVAAPKVKGGVDVTLPRVEGKVKVPEVDVRGPKVDVSAPDVEAHGPEWNLKMPKMKMPTFSTPGAKGEGPDVHMTLPKGDISISGPKVNVEAPDVNLEGLGGKLKGPDVKLPDMSVKTPKISMPDVDLHVKGTKVKGEYDVTVPKLEGELKGPKVDIDAPDVDVHGPDWHLKMPKMKMPKFSVPGFKAEGPEVDVNLPKADVDISGPKIDVTAPDVSIEEPEGKLKGPKFKMPEMNIKVPKISMPDVDLHLKGPNVKGEYDVTMPKVESEIKVPDVELKSAKMDIDVPDVEVQGPDWHLKMPKMKMPKFSMPGFKAEGPEVDVNLPKADVDISGPKVGVEVPDVNIEGPEGKLKGPKFKMPEMNIKAPKISMPDVDLHMKGPKVKGEYDMTVPKLEGDLKGPKVDVSAPDVEMQGPDWNLKMPKIKMPKFSMPSLKGEGPEFDVNLSKANVDISAPKVDTNAPDLSLEGPEGKLKGPKFKMPEMHFRAPKMSLPDVDLDLKGPKMKGNVDISAPKIEGEMQVPDVDIRGPKVDIKAPDVEGQGLDWSLKIPKMKMPKFSMPSLKGEGPEVDVNLPKADVVVSGPKVDIEAPDVSLEGPEGKLKGPKFKMPEMHFKTPKISMPDVDLHLKGPKVKGDVDVSVPKVEGEMKVPDVEIKGPKMDIDAPDVEVQGPDWHLKMPKMKMPKFSMPGFKGEGREVDVNLPKADIDVSGPKVDVEVPDVSLEGPEGKLKGPKFKMPEMHFKAPKISMPDVDLNLKGPKLKGDVDVSLPEVEGEMKVPDVDIKGPKVDISAPDVDVHGPDWHLKMPKVKMPKFSMPGFKGEGPEVDVKLPKADVDVSGPKMDAEVPDVNIEGPDAKLKGPKFKMPEMSIKPQKISIPDVGLHLKGPKMKGDYDVTVPKVEGEIKAPDVDIKGPKVDINAPDVEVHGPDWHLKMPKVKMPKFSMPGFKGEGPEVDMNLPKADLGVSGPKVDIDVPDVNLEAPEGKLKGPKFKMPSMNIQTHKISMPDVGLNLKAPKLKTDVDVSLPKVEGDLKGPEIDVKAPKMDVNVGDIDIEGPEGKLKGPKFKMPEMHFKAPKISMPDVDLHLKGPKVKGDMDVSVPKVEGEMKVPDVDIKGPKVDIDAPDVEVHDPDWHLKMPKMKMPKFSMPGFKAEGPEVDVNLPKADIDVSGPSVDTDAPDLDIEGPEGKLKGSKFKMPKLNIKAPKVSMPDVDLNLKGPKLKGEIDASVPELEGDLRGPQVDVKGPFVEAEVPDVDLECPDAKLKGPKFKMPEMHFKAPKISMPDVDLHLKGPKVKGDADVSVPKLEGDLTGPSVGVEVPDVELECPDAKLKGPKFKMPDMHFKAPKISMPDVDLHLKGPKVKGDVDVSVPKLEGDLTGPSVGVEVPDVELECPDAKLKGPKFKMPEMHFKTPKISMPDVDLHLKGPKVKGDMDVSVPKVEGEMKVPDVDIKGPKMDIDAPDVDVHGPDWHLKMPKMKMPKFSMPGFKAEGPEVDVNLPKADVVVSGPKVDVEVPDVSLEGPEGKLKGPKLKMPEMHFKAPKISMPDVDLHLKGPKVKGDVDVSLPKLEGDLTGPSVDVEVPDVELECPDAKLKGPKFKMPEMHFKTPKISMPDVNLNLKGPKVKGDMDVSVPKVEGEMKVPDVDIRGPKVDIDAPDVDVHGPDWHLKMPKMKMPKFSMPGFKGEGPEVDVNLPKADVDVSGPKVDVEVPDVSLEGPEGKLKGPKFKMPEMHFKTPKISMPDVDFNLKGPKIKGDVDVSAPKLEGELKGPELDVKGPKLDADMPEVAVEGPNGKWKTPKFKMPDMHFKAPKISMPDLDLHLKSPKAKGEVDVDVPKLEGDLKGPHVDVSGPDIDIEGPEGKLKGPKFKMPDMHFKAPNISMPDVDLNLKGPKIKGDVDVSVPEVEGKLEVPDMNIRGPKVDVNAPDVQAPDWHLKMPKMKMPKFSMPGFKAEGPEVDVNLPKADVDISGPKVDIEGPDVNIEGPEGKLKGPKLKMPEMNIKAPKISMPDFDLHLKGPKVKGDVDVSLPKVEGDLKGPEVDIKGPKVDINAPDVGVQGPDWHLKMPKVKMPKFSMPGFKGEGPDGDVKLPKADIDVSGPKVDIEGPDVNIEGPEGKLKGPKFKMPEMNIKAPKISMPDIDLNLKGPKVKGDVDVSLPKVEGDLKGPEVDIKGPKVDIDAPDVDVHGPDWHLKMPKIKMPKISMPGFKGEGPDVDVNLPKADIDVSGPKVDVECPDVNIEGPEGKWKSPKFKMPEMHFKTPKISMPDIDLNLTGPKIKGDVDVTGPKVEGDLKGPEVDLKGPKVDIDVPDVNVQGPDWHLKMPKMKMPKFSMPGFKAEGPEVDVNLPKADVDVSGPKVDVEGPDVNIEGPEGKLKGPKFKMPEMNIKAPKIPMPDFDLHLKGPKVKGDVDISLPKVEGDLKGPEVDIRGPQVDIDVPDVGVQGPDWHLKMPKVKMPKFSMPGFKGEGPDVDVNLPKADLDVSGPKVDIDVPDVNIEGPEGKLKGPKFKMPEMNIKAPKISMPDIDLNLKGPKVKGDMDVSLPKVEGDMKVPDVDIKGPKVDINAPDVDVQGPDWHLKMPKIKMPKISMPGFKGEGPEVDVNLPKADLDVSGPKVDVDVPDVNIEGPDAKLKGPKFKMPEMNIKAPKISMPDLDLNLKGPKMKGEVDVSLANVEGDLKGPALDIKGPKIDVDAPDIDIHGPDAKLKGPKLKMPDMHVNMPKISMPEIDLNLKGSKLKGDVDVSGPKLEGDIKAPSLDIKGPEVDVSGPKLNIEGKSKKSRFKLPKFNFSGSKVQTPEVDVKGKKPDIDITGPKVDINAPDVEVQGKVKGSKFKMPFLSISSPKVSMPDVELNLKSPKVKGDLDIAGPNLEGDFKGPKVDIKAPEVNLNAPDVDVHGPDWNLKMPKMKMPKFSVSGLKAEGPDVAVDLPKGDINIEGPSMNIEGPDLNVEGPEGGLKGPKFKMPDMNIKAPKISMPDIDLNLKGPKVKGDVDISLPKLEGDLKGPEVDIKGPKVDINAPDVDVHGPDWHLKMPKVKMPKFSMPGFKGEGPEVDVTLPKADIDISGPNVDVDVPDVNIEGPDAKLKGPKFKMPEMNIKAPKISMPDFDLNLKGPKMKGDVVVSLPKVEGDLKGPEVDIKGPKVDIDTPDINIEGSEGKFKGPKFKIPEMHLKAPKISMPDIDLNLKGPKVKGDVDVSLPKMEGDLKGPEVDIKGPKVDINAPDVDVQGPDWHLKMPKVKMPKFSMPGFKGEGPDVDVNLPKADLDVSGPKVDIDVPDVNIEGPEGKLKGPKFKMPEMNIKAPKISMPDIDLNLKGPKVKGDMDVSLPKVEGDMQVPDLDIKGPKVDINAPDVDVRGPDWHLKMPKIKMPKISMPGFKGEGPEVDVNLPKADLDVSGPKVDVDVPDVNIEGPDAKLKGPKFKMPEMNIKAPKISMPDFDLHLKGPKVKGDVDVSLPKMEGDLKAPEVDIKGPKVDIDAPDVDVHGPDWHLKMPKVKMPKFSMPGFKGEGPEVDVNLPKADIDVSGPKVDIDTPDIDIHGPEGKLKGPKFKMPDLHLKAPKISMPEVDLNLKGPKMKGDVDVSLPKVEGDLKGPEVDIKGPKVDIDVPDVDVQGPDWHLKMPKVKMPKFSMPGFKGEGPDVDVNLPKADLDVSGPKVDIDVPDVNIEGPDAKLKGPKFKMPEMNIKAPKISMPDFDLHLKGPKVKGDVDVSLPKVEGDLKGPEVDIKGPKVDIDAPDVDVHGPDWHLKMPKVKMPKFSMPGFKGEGPDVDVTLPKADIEISGPKVDIDAPDVSIEGPDAKLKGPKFKMPEMNIKAPKISMPDIDFNLKGPKVKGDVDVSLPKVEGDLKGPEIDIKGPSLDIDTPDVNIEGPEGKLKGPKFKMPEMNIKAPKISMPDFDLHLKGPKVKGDVDVSLPKVESDLKGPEVDIEGPEGKLKGPKFKMPDVHFKSPQISMSDIDLNLKGPKIKGDMDISVPKLEGDLKGPKVDVKGPKVGIDTPDIDIHGPEGKLKGPKFKMPDLHLKAPKISMPEVDLNLKGPKVKGDMDISLPKVEGDLKGPEVDIRDPKVDIDVPDVDVQGPDWHLKMPKVKMPKFSMPGFKGEGPDVDVNLPKADIDVSGPKVDVDVPDVNIEGPDAKLKGPKFKMPEMSIKAPKISMPDIDLNLKGPKVKGDVDVTLPKVEGDLKGPEADIKGPKVDINTPDVDVHGPDWHLKMPKVKMPKFSMPGFKGEGPDVDVSLPKADIDVSGPKVDVDIPDVNIEGPDAKLKGPKFKMPEINIKAPKISIPDVDLDLKGPKVKGDFDVSVPKVEGTLKGPEVDLKGPRLDFEGPDAKLSGPSLKMPSLEISAPKVTAPDVDLHLKAPKIGFSGPKLEGGEVDLKGPKVEAPSLDVHMDSPDINIEGPDVKIPKFKKPKFGFGAKSPKADIKSPSLDVTVPEAELNLETPEISVGGKGKKSKFKMPKIHMSGPKIKAKKQGFDLNVPGGEIDASLKAPDVDVNIAGPDAALKVDVKSPKTKKTMFGKMYFPDVEFDIKSPKFKAEAPLPSPKLEGELQAPDLELSLPAIHVEGLDIKAKAPKVKMPDVDISVPKIEGDLKGPKVQANLGAPDINIEGLDAKVKTPSFGISAPQVSIPDVNVNLKGPKIKGDVPSVGLEGPDVDLQGPEAKIKFPKFSMPKIGIPGVKMEG

GGAEVHAQLPSLEGDLRGPDVKLEGPDVSLKGPGVDLPSVNLSMPKVSGPDLDLNLKGPSLKGDLDASVPSMKVHAPGLNLSGVGGKMQVGGDGVKVPGIDATTKLNVGAPDVTLRGPSLQGDLAVSGDIKCPKVSVGAPDLSLEASEGSIKLPKMKLPQFGISTPGSDLHVNAKGPQVSGELKGPGVDVNLKGPRISAPNVDFNLEGPKVKGSLGATGEIKGPTVGGGLPGIGVQGLEGNLQMPGIKSSGCDVNLPGVNVKLPTGQISGPEIKGGLKGSEVGFHGAAPDISVKGPAFNMASPESDFGINLKGPKIKGGADVSGGVSAPDISLGEGHLSVKGSGGEWKGPQVSSALNLDTSKFAGGLHFSGPKVEGGVKGGQIGLQAPGLSVSGPQGHLESGSGKVTFPKMKIPKFTFSGRELVGREMGVDVHFPKAEASIQAGAGDGEWEESEVKLKKSKIKMPKFNFSKPKGKGGVTGSPEASISGSKGDLKSSKASLGSLEGEAEAEASSPKGKFSLFKSKKPRHRSNSFSDEREFSGPSTPTGTLEFEGGEVSLEGGKVKGKHGKLKFGTFGGLGSKSKGHYEVTGSDDETGKLQGSGVSLASKKSRLSSSSSNDSGNKVGIQLPEVELSVSTKKE

2) >gi|41322916|ref|NP_958782.1| plectin isoform 1 [Homo sapiens] (531.5 kD)

MVAGMLMPRDQLRAIYEVLFREGVMVAKKDRRPRSLHPHVPGVTNLQVMRAMASLRARGLVRETFAWCHFYWYLTNEGIAHLRQYLHLPPEIVPASLQRVRRPVAMVMPARRTPHVQAVQGPLGSPPKRGPLPTEEQRVYRRKELEEVSPETPVVPATTQRTLARPGPEPAPATDERDRVQKKTFTKWVNKHLIKAQRHISDLYEDLRDGHNLISLLEVLSGDSLPREKGRMRFHKLQNVQIALDYLRHRQVKLVNIRNDDIADGNPKLTLGLIWTIILHFQISDIQVSGQSEDMTAKEKLLLWSQRMVEGYQGLRCDNFTSSWRDGRLFNAIIHRHKPLLIDMNKVYRQTNLENLDQAFSVAERDLGVTRLLDPEDVDVPQPDEKSIITYVSSLYDAMPRVPDVQDGVRANELQLRWQEYRELVLLLLQWMRHHTAAFEERRFPSSFEEIEILWSQFLKFKEMELPAKEADKNRSKGIYQSLEGAVQAGQLKVPPGYHPLDVEKEWGKLHVAILEREKQLRSEFERLECLQRIVTKLQMEAGLCEEQLNQADALLQSDVRLLAAGKVPQRAGEVERDLDKADSMIRLLFNDVQTLKDGRHPQGEQMYRRVYRLHERLVAIRTEYNLRLKAGVAAPATQVAQVTLQSVQRRPELEDSTLRYLQDLLAWVEENQHRVDGAEWGVDLPSVEAQLGSHRGLHQSIEEFRAKIERARSDEGQLSPATRGAYRDCLGRLDLQYAKLLNSSKARLRSLESLHSFVAAATKELMWLNEKEEEEVGFDWSDRNTNMTAKKESYSALMRELELKEKKIKELQNAGDRLLREDHPARPTVESFQAALQTQWSWMLQLCCCIEAHLKENAAYFQFFSDVREAEGQLQKLQEALRRKYSCDRSATVTRLEDLLQDAQDEKEQLNEYKGHLSGLAKRAKAVVQLKPRHPAHPMRGRLPLLAVCDYKQVEVTVHKGDECQLVGPAQPSHWKVLSSSGSEAAVPSVCFLVPPPNQEAQEAVTRLEAQHQALVTLWHQLHVDMKSLLAWQSLRRDVQLIRSWSLATFRTLKPEEQRQALHSLELHYQAFLRDSQDAGGFGPEDRLMAEREYGSCSHHYQQLLQSLEQGAQEESRCQRCISELKDIRLQLEACETRTVHRLRLPLDKEPARECAQRIAEQQKAQAEVEGLGKGVARLSAEAEKVLALPEPSPAAPTLRSELELTLGKLEQVRSLSAIYLEKLKTISLVIRGTQGAEEVLRAHEEQLKEAQAVPATLPELEATKASLKKLRAQAEAQQPTFDALRDELRGAQEVGERLQQRHGERDVEVERWRERVAQLLERWQAVLAQTDVRQRELEQLGRQLRYYRESADPLGAWLQDARRRQEQIQAMPLADSQAVREQLRQEQALLEEIERHGEKVEECQRFAKQYINAIKDYELQLVTYKAQLEPVASPAKKPKVQSGSESVIQEYVDLRTHYSELTTLTSQYIKFISETLRRMEEEERLAEQQRAEERERLAEVEAALEKQRQLAEAHAQAKAQAEREAKELQQRMQEEVVRREEAAVDAQQQKRSIQEELQQLRQSSEAEIQAKARQAEAAERSRLRIEEEIRVVRLQLEATERQRGGAEGELQALRARAEEAEAQKRQAQEEAERLRRQVQDESQRKRQAEVELASRVKAEAEAAREKQRALQALEELRLQAEEAERRLRQAEVERARQVQVALETAQRSAEAELQSKRASFAEKTAQLERSLQEEHVAVAQLREEAERRAQQQAEAERAREEAERELERWQLKANEALRLRLQAEEVAQQKSLAQAEAEKQKEEAEREARRRGKAEEQAVRQRELAEQELEKQRQLAEGTAQQRLAAEQELIRLRAETEQGEQQRQLLEEELARLQREAAAATQKRQELEAELAKVRAEMEVLLASKARAEEESRSTSEKSKQRLEAEAGRFRELAEEAARLRALAEEAKRQRQLAEEDAARQRAEAERVLAEKLAAIGEATRLKTEAEIALKEKEAENERLRRLAEDEAFQRRRLEEQAAQHKADIEERLAQLRKASDSELERQKGLVEDTLRQRRQVEEEILALKASFEKAAAGKAELELELGRIRSNAEDTLRSKEQAELEAARQRQLAAEEERRRREAEERVQKSLAAEEEAARQRKAALEEVERLKAKVEEARRLRERAEQESARQLQLAQEAAQKRLQAEEKAHAFAVQQKEQELQQTLQQEQSVLDQLRGEAEAARRAAEEAEEARVQAEREAAQSRRQVEEAERLKQSAEEQAQARAQAQAAAEKLRKEAEQEAARRAQAEQAALRQKQAADAEMEKHKKFAEQTLRQKAQVEQELTTLRLQLEETDHQKNLLDEELQRLKAEATEAARQRSQVEEELFSVRVQMEELSKLKARIEAENRALILRDKDNTQRFLQEEAEKMKQVAEEAARLSVAAQEAARLRQLAEEDLAQQRALAEKMLKEKMQAVQEATRLKAEAELLQQQKELAQEQARRLQEDKEQMAQQLAEETQGFQRTLEAERQRQLEMSAEAERLKLRVAEMSRAQARAEEDAQRFRKQAEEIGEKLHRTELATQEKVTLVQTLEIQRQQSDHDAERLREAIAELEREKEKLQQEAKLLQLKSEEMQTVQQEQLLQETQALQQSFLSEKDSLLQRERFIEQEKAKLEQLFQDEVAKAQQLREEQQRQQQQMEQERQRLVASMEEARRRQHEAEEGVRRKQEELQQLEQQRRQQEELLAEENQRLREQLQLLEEQHRAALAHSEEVTASQVAATKTLPNGRDALDGPAAEAEPEHSFDGLRRKVSAQRLQEAGILSAEELQRLAQGHTTVDELARREDVRHYLQGRSSIAGLLLKATNEKLSVYAALQRQLLSPGTALILLEAQAASGFLLDPVRNRRLTVNEAVKEGVVGPELHHKLLSAERAVTGYKDPYTGQQISLFQAMQKGLIVREHGIRLLEAQIATGGVIDPVHSHRVPVDVAYRRGYFDEEMNRVLADPSDDTKGFFDPNTHENLTYLQLLERCVEDPETGLCLLPLTDKAAKGGELVYTDSEARDVFEKATVSAPFGKFQGKTVTIWEIINSEYFTAEQRRDLLRQFRTGRITVEKIIKIIITVVEEQEQKGRLCFEGLRSLVPAAELLESRVIDRELYQQLQRGERSVRDVAEVDTVRRALRGANVIAGVWLEEAGQKLSIYNALKKDLLPSDMAVALLEAQAGTGHIIDPATSARLTVDEAVRAGLVGPEFHEKLLSAEKAVTGYRDPYTGQSVSLFQALKKGLIPREQGLRLLDAQLSTGGIVDPSKSHRVPLDVACARGCLDEETSRALSAPRADAKAYSDPSTGEPATYGELQQRCRPDQLTGLSLLPLSEKAARARQEELYSELQARETFEKTPVEVPVGGFKGRTVTVWELISSEYFTAEQRQELLRQFRTGKVTVEKVIKILITIVEEVETLRQERLSFSGLRAPVPASELLASGVLSRAQFEQLKDGKTTVKDLSELGSVRTLLQGSGCLAGIYLEDTKEKVSIYEAMRRGLLRATTAALLLEAQAATGFLVDPVRNQRLYVHEAVKAGVVGPELHEQLLSAEKAVTGYRDPYSGSTISLFQAMQKGLVLRQHGIRLLEAQIATGGIIDPVHSHRVPVDVAYQRGYFSEEMNRVLADPSDDTKGFFDPNTHENLTYRQLLERCVEDPETGLRLLPLKGAEKAEVVETTQVYTEEETRRAFEETQIDIPGGGSHGGSTMSLWEVMQSDLIPEEQRAQLMADFQAGRVTKERMIIIIIEIIEKTEIIRQQGLASYDYVRRRLTAEDLFEARIISLETYNLLREGTRSLREALEAESAWCYLYGTGSVAGVYLPGSRQTLSIYQALKKGLLSAEVARLLLEAQAATGFLLDPVKGERLTVDEAVRKGLVGPELHDRLLSAERAVTGYRDPYTEQTISLFQAMKKELIPTEEALRLLDAQLATGGIVDPRLGFHLPLEVAYQRGYLNKDTHDQLSEPSEVRSYVDPSTDERLSYTQLLRRCRRDDGTGQLLLPLSDARKLTFRGLRKQITMEELVRSQVMDEATALQLREGLTSIEEVTKNLQKFLEGTSCIAGVFVDATKERLSVYQAMKKGIIRPGTAFELLEAQAATGYVIDPIKGLKLTVEEAVRMGIVGPEFKDKLLSAERAVTGYKDPYSGKLISLFQAMKKGLILKDHGIRLLEAQIATGGIIDPEESHRLPVEVAYKRGLFDEEMNEILTDPSDDTKGFFDPNTEENLTYLQLMERCITDPQTGLCLLPLKEKKRERKTSSKSSVRKRRVVIVDPETGKEMSVYEAYRKGLIDHQTYLELSEQECEWEEITISSSDGVVKSMIIDRRSGRQYDIDDAIAKNLIDRSALDQYRAGTLSITEFADMLSGNAGGFRSRSSSVGSSSSYPISPAVSRTQLASWSDPTEETGPVAGILDTETLEKVSITEAMHRNLVDNITGQRLLEAQACTGGIIDPSTGERFPVTDAVNKGLVDKIMVDRINLAQKAFCGFEDPRTKTKMSAAQALKKGWLYYEAGQRFLEVQYLTGGLIEPDTPGRVPLDEALQRGTVDARTAQKLRDVGAYSKYLTCPKTKLKISYKDALDRSMVEEGTGLRLLEAAAQSTKGYYSPYSVSGSGSTAGSRTGSRTGSRAGSRRGSFDATGSGFSMTFSSSSYSSSGYGRRYASGSSASLGGPESAVA

3) >gi|118572613|ref|NP_057417.3| serine/arginine repetitive matrix protein 2 [Homo sapiens] (299.4 kD)

MYNGIGLPTPRGSGTNGYVQRNLSLVRGRRGERPDYKGEEELRRLEAALVKRPNPDILDHERKRRVELRCLELEEMMEEQGYEEQQIQEKVATFRLMLLEKDVNPGGKEETPGQRPAVTETHQLAELNEKKNERLRAAFGISDSYVDGSSFDPQRRAREAKQPAPEPPKPYSLVRESSSSRSPTPKQKKKKKKKDRGRRSESSSPRRERKKSSKKKKHRSESESKKRKHRSPTPKSKRKSKDKKRKRSRSTTPAPKSRRAHRSTSADSASSSDTSRSRSRSAAAKTHTTALAGRSPSPASGRRGEGDAPFSEPGTTSTQRPSSPETATKQPSSPYEDKDKDKKEKSATRPSPSPERSSTGPEPPAPTPLLAERHGGSPQPLATTPLSQEPVNPPSEASPTRDRSPPKSPEKLPQSSSSESSPPSPQPTKVSRHASSSPESPKPAPAPGSHREISSSPTSKNRSHGRAKRDKSHSHTPSRRMGRSRSPATAKRGRSRSRTPTKRGHSRSRSPQWRRSRSAQRWGRSRSPQRRGRSRSPQRPGWSRSRNTQRRGRSRSARRGRSHSRSPATRGRSRSRTPARRGRSRSRTPARRRSRSRTPTRRRSRSRTPARRGRSRSRTPARRRSRTRSPVRRRSRSRSPARRSGRSRSRTPARRGRSRSRTPARRGRSRSRTPARRSGRSRSRTPARRGRSRSRTPRRGRSRSRSLVRRGRSHSRTPQRRGRSGSSSERKNKSRTSQRRSRSNSSPEMKKSRISSRRSRSLSSPRSKAKSRLSLRRSLSGSSPCPKQKSQTPPRRSRSGSSQPKAKSRTPPRRSRSSSSPPPKQKSKTPSRQSHSSSSPHPKVKSGTPPRQGSITSPQANEQSVTPQRRSCFESSPDPELKSRTPSRHSCSGSSPPRVKSSTPPRQSPSRSSSPQPKVKAIISPRQRSHSGSSSPSPSRVTSRTTPRRSRSVSPCSNVESRLLPRYSHSGSSSPDTKVKPETPPRQSHSGSISPYPKVKAQTPPGPSLSGSKSPCPQEKSKDSLVQSCPGSLSLCAGVKSSTPPGESYFGVSSLQLKGQSQTSPDHRSDTSSPEVRQSHSESPSLQSKSQTSPKGGRSRSSSPVTELASRSPIRQDRGEFSASPMLKSGMSPEQSRFQSDSSSYPTVDSNSLLGQSRLETAESKEKMALPPQEDATASPPRQKDKFSPFPVQDRPESSLVFKDTLRTPPRERSGAGSSPETKEQNSALPTSSQDEELMEVVEKSEEPAGQILSHLSSELKEMSTSNFESSPEVEERPAVSLTLDQSQSQASLEAVEVPSMASSWGGPHFSPEHKELSNSPLRENSFGSPLEFRNSGPLGTEMNTGFSSEVKEDLNGPFLNQLETDPSLDMKEQSTRSSGHSSSELSPDAVEKAGMSSNQSISSPVLDAVPRTPSRERSSSASSPEMKDGLPRTPSRRSRSGSSPGLRDGSGTPSRHSLSGSSPGMKDIPRTPSRGRSECDSSPEPKALPQTPRPRSRSPSSPELNNKCLTPQRERSGSESSVDQKTVARTPLGQRSRSGSSQELDVKPSASPQERSESDSSPDSKAKTRTPLRQRSRSGSSPEVDSKSRLSPRRSRSGSSPEVKDKPRAAPRAQSGSDSSPEPKAPAPRALPRRSRSGSSSKGRGPSPEGSSSTESSPEHPPKSRTARRGSRSSPEPKTKSRTPPRRRSSRSSPELTRKARLSRRSRSASSSPETRSRTPPRHRRSPSVSSPEPAEKSRSSRRRRSASSPRTKTTSRRGRSPSPKPRGLQRSRSRSRREKTRTTRRRDRSGSSQSTSRRRQRSRSRSRVTRRRRGGSGYHSRSPARQESSRTSSRRRRGRSRTPPTSRKRSRSRTSPAPWKRSRSRASPATHRRSRSRTPLISRRRSRSRTSPVSRRRSRSRTSVTRRRSRSRASPVSRRRSRSRTPPVTRRRSRSRTPTTRRRSRSRTPPVTRRRSRSRTPPVTRRRSRSRTSPITRRRSRSRTSPVTRRRSRSRTSPVTRRRSRSRTSPVTRRRSRSRTPPAIRRRSRSRTPLLPRKRSRSRSPLAIRRRSRSRTPRTARGKRSLTRSPPAIRRRSASGSSSDRSRSATPPATRNHSGSRTPPVALNSSRMSCFSRPSMSPTPLDRCRSPGMLEPLGSSRTPMSVLQQAGGSMMDGPGPRIPDHQRTSVPENHAQSRIALALTAISLGTARPPPSMSAAGLAARMSQVPAPVPLMSLRTAPAANLASRIPAASAAAMNLASARTPAIPTAVNLADSRTPAAAAAMNLASPRTAVAPSAVNLADPRTPTAPAVNLAGARTPAALAALSLTGSGTPPTAANYPSSSRTPQAPASANLVGPRSAHATAPVNIAGSRTAAALAPASLTSARMAPALSGANLTSPRVPLSAYERVSGRTSPPLLDRARSRTPPSAPSQSRMTSERAPSPSSRMGQAPSQSLLPPAQDQPRSPVPSAFSDQSRCLIAQTTPVAGSQSLSSGAVATTTSSAGDHNGMLSVPAPGVPHSDVGEPPASTGAQQPSALAALQPAKERRSSSSSSSSSSSSSSSSSSSSSSSSSGSSSSDSEGSSLPVQPEVALKRVPSPTPAPKEAVREGRPPEPTPAKRKRRSSSSSSSSSSSSSSSSSSSSSSSSSSSSSSSSSSSSSSSSSSPSPAKPGPQALPKPASPKKPPPGERRSRSPRKPIDSLRDSRSLSYSPVERRRPSPQPSPRDQQSSSSERGSRRGQRGDSRSPSHKRRRETPSPRPMRHRSSRSP

4) >gi|153945728|ref|NP_005900.2| microtubule-associated protein 1B [Homo sapiens] (270.5 kD)

MATVVVEATEPEPSGSIANPAASTSPSLSHRFLDSKFYLLVVVGEIVTEEHLRRAIGNIELGIRSWDTNLIECNLDQELKLFVSRHSARFSPEVPGQKILHHRSDVLETVVLINPSDEAVSTEVRLMITDAARHKLLVLTGQCFENTGELILQSGSFSFQNFIEIFTDQEIGELLSTTHPANKASLTLFCPEEGDWKNSNLDRHNLQDFINIKLNSASILPEMEGLSEFTEYLSESVEVPSPFDILEPPTSGGFLKLSKPCCYIFPGGRGDSALFAVNGFNMLINGGSERKSCFWKLIRHLDRVDSILLTHIGDDNLPGINSMLQRKIAELEEEQSQGSTTNSDWMKNLISPDLGVVFLNVPENLKNPEPNIKMKRSIEEACFTLQYLNKLSMKPEPLFRSVGNTIDPVILFQKMGVGKLEMYVLNPVKSSKEMQYFMQQWTGTNKDKAEFILPNGQEVDLPISYLTSVSSLIVWHPANPAEKIIRVLFPGNSTQYNILEGLEKLKHLDFLKQPLATQKDLTGQVPTPVVKQTKLKQRADSRESLKPAAKPLPSKSVRKESKEETPEVTKVNHVEKPPKVESKEKVMVKKDKPIKTETKPSVTEKEVPSKEEPSPVKAEVAEKQATDVKPKAAKEKTVKKETKVKPEDKKEEKEKPKKEVAKKEDKTPIKKEEKPKKEEVKKEVKKEIKKEEKKEPKKEVKKETPPKEVKKEVKKEEKKEVKKEEKEPKKEIKKLPKDAKKSSTPLSEAKKPAALKPKVPKKEESVKKDSVAAGKPKEKGKIKVIKKEGKAAEAVAAAVGTGATTAAVMAAAGIAAIGPAKELEAERSLMSSPEDLTKDFEELKAEEVDVTKDIKPQLELIEDEEKLKETEPVEAYVIQKEREVTKGPAESPDEGITTTEGEGECEQTPEELEPVEKQGVDDIEKFEDEGAGFEESSETGDYEEKAETEEAEEPEEDGEEHVCVSASKHSPTEDEESAKAEADAYIREKRESVASGDDRAEEDMDEAIEKGEAEQSEEEADEEDKAEDAREEEYEPEKMEAEDYVMAVVDKAAEAGGAEEQYGFLTTPTKQLGAQSPGREPASSIHDETLPGGSESEATASDEENREDQPEEFTATSGYTQSTIEISSEPTPMDEMSTPRDVMSDETNNEETESPSQEFVNITKYESSLYSQEYSKPADVTPLNGFSEGSKTDATDGKDYNASASTISPPSSMEEDKFSRSALRDAYCSEVKASTTLDIKDSISAVSSEKVSPSKSPSLSPSPPSPLEKTPLGERSVNFSLTPNEIKVSAEAEVAPVSPEVTQEVVEEHCASPEDKTLEVVSPSQSVTGSAGHTPYYQSPTDEKSSHLPTEVIEKPPAVPVSFEFSDAKDENERASVSPMDEPVPDSESPIEKVLSPLRSPPLIGSESAYESFLSADDKASGRGAESPFEEKSGKQGSPDQVSPVSEMTSTSLYQDKQEGKSTDFAPIKEDFGQEKKTDDVEAMSSQPALALDERKLGDVSPTQIDVSQFGSFKEDTKMSISEGTVSDKSATPVDEGVAEDTYSHMEGVASVSTASVATSSFPEPTTDDVSPSLHAEVGSPHSTEVDDSLSVSVVQTPTTFQETEMSPSKEECPRPMSISPPDFSPKTAKSRTPVQDHRSEQSSMSIEFGQESPEQSLAMDFSRQSPDHPTVGAGVLHITENGPTEVDYSPSDMQDSSLSHKIPPMEEPSYTQDNDLSELISVSQVEASPSTSSAHTPSQIASPLQEDTLSDVAPPRDMSLYASLTSEKVQSLEGEKLSPKSDISPLTPRESSPLYSPTFSDSTSAVKEKTATCHSSSSPPIDAASAEPYGFRASVLFDTMQHHLALNRDLSTPGLEKDSGGKTPGDFSYAYQKPEETTRSPDEEDYDYESYEKTTRTSDVGGYYYEKIERTTKSPSDSGYSYETIGKTTKTPEDGDYSYEIIEKTTRTPEEGGYSYDISEKTTSPPEVSGYSYEKTERSRRLLDDISNGYDDSEDGGHTLGDPSYSYETTEKITSFPESEGYSYETSTKTTRTPDTSTYCYETAEKITRTPQASTYSYETSDLCYTAEKKSPSEARQDVDLCLVSSCEYKHPKTELSPSFINPNPLEWFASEEPTEESEKPLTQSGGAPPPPGGKQQGRQCDETPPTSVSESAPSQTDSDVPPETEECPSITADANIDSEDESETIPTDKTVTYKHMDPPPAPVQDRSPSPRHPDVSMVDPEALAIEQNLGKALKKDLKEKTKTKKPGTKTKSSSPVKKSDGKSKPLAASPKPAGLKESSDKVSRVASPKKKESVEKAAKPTTTPEVKAARGEEKDKETKNAANASASKSAKTATAGPGTTKTTKSSAVPPGLPVYLDLCYIPNHSNSKNVDVEFFKRVRSSYYVVSGNDPAAEEPSRAVLDALLEGKAQWGSNMQVTLIPTHDSEVMREWYQETHEKQQDLNIMVLASSSTVVMQDESFPACKIEL

5) >gi|223029410|ref|NP_006280.3| talin-1 [Homo sapiens] (269.6 kD)

MVALSLKISIGNVVKTMQFEPSTMVYDACRIIRERIPEAPAGPPSDFGLFLSDDDPKKGIWLEAGKALDYYMLRNGDTMEYRKKQRPLKIRMLDGTVKTIMVDDSKTVTDMLMTICARIGITNHDEYSLVRELMEEKKEEITGTLRKDKTLLRDEKKMEKLKQKLHTDDELNWLDHGRTLREQGVEEHETLLLRRKFFYSDQNVDSRDPVQLNLLYVQARDDILNGSHPVSFDKACEFAGFQCQIQFGPHNEQKHKAGFLDLKDFLPKEYVKQKGERKIFQAHKNCGQMSEIEAKVRYVKLARSLKTYGVSFFLVKEKMKGKNKLVPRLLGITKECVMRVDEKTKEVIQEWNLTNIKRWAASPKSFTLDFGDYQDGYYSVQTTEGEQIAQLIAGYIDIILKKKKSKDHFGLEGDEESTMLEDSVSPKKSTVLQQQYNRVGKVEHGSVALPAIMRSGASGPENFQVGSMPPAQQQITSGQMHRGHMPPLTSAQQALTGTINSSMQAVQAAQATLDDFDTLPPLGQDAASKAWRKNKMDESKHEIHSQVDAITAGTASVVNLTAGDPAETDYTAVGCAVTTISSNLTEMSRGVKLLAALLEDEGGSGRPLLQAAKGLAGAVSELLRSAQPASAEPRQNLLQAAGNVGQASGELLQQIGESDTDPHFQDALMQLAKAVASAAAALVLKAKSVAQRTEDSGLQTQVIAAATQCALSTSQLVACTKVVAPTISSPVCQEQLVEAGRLVAKAVEGCVSASQAATEDGQLLRGVGAAATAVTQALNELLQHVKAHATGAGPAGRYDQATDTILTVTENIFSSMGDAGEMVRQARILAQATSDLVNAIKADAEGESDLENSRKLLSAAKILADATAKMVEAAKGAAAHPDSEEQQQRLREAAEGLRMATNAAAQNAIKKKLVQRLEHAAKQAAASATQTIAAAQHAASTPKASAGPQPLLVQSCKAVAEQIPLLVQGVRGSQAQPDSPSAQLALIAASQSFLQPGGKMVAAAKASVPTIQDQASAMQLSQCAKNLGTALAELRTAAQKAQEACGPLEMDSALSVVQNLEKDLQEVKAAARDGKLKPLPGETMEKCTQDLGNSTKAVSSAIAQLLGEVAQGNENYAGIAARDVAGGLRSLAQAARGVAALTSDPAVQAIVLDTASDVLDKASSLIEEAKKAAGHPGDPESQQRLAQVAKAVTQALNRCVSCLPGQRDVDNALRAVGDASKRLLSDSLPPSTGTFQEAQSRLNEAAAGLNQAATELVQASRGTPQDLARASGRFGQDFSTFLEAGVEMAGQAPSQEDRAQVVSNLKGISMSSSKLLLAAKALSTDPAAPNLKSQLAAAARAVTDSINQLITMCTQQAPGQKECDNALRELETVRELLENPVQPINDMSYFGCLDSVMENSKVLGEAMTGISQNAKNGNLPEFGDAISTASKALCGFTEAAAQAAYLVGVSDPNSQAGQQGLVEPTQFARANQAIQMACQSLGEPGCTQAQVLSAATIVAKHTSALCNSCRLASARTTNPTAKRQFVQSAKEVANSTANLVKTIKALDGAFTEENRAQCRAATAPLLEAVDNLSAFASNPEFSSIPAQISPEGRAAMEPIVISAKTMLESAGGLIQTARALAVNPRDPPSWSVLAGHSRTVSDSIKKLITSMRDKAPGQLECETAIAALNSCLRDLDQASLAAVSQQLAPREGISQEALHTQMLTAVQEISHLIEPLANAARAEASQLGHKVSQMAQYFEPLTLAAVGAASKTLSHPQQMALLDQTKTLAESALQLLYTAKEAGGNPKQAAHTQEALEEAVQMMTEAVEDLTTTLNEAASAAGVVGGMVDSITQAINQLDEGPMGEPEGSFVDYQTTMVRTAKAIAVTVQEMVTKSNTSPEELGPLANQLTSDYGRLASEAKPAAVAAENEEIGSHIKHRVQELGHGCAALVTKAGALQCSPSDAYTKKELIECARRVSEKVSHVLAALQAGNRGTQACITAASAVSGIIADLDTTIMFATAGTLNREGTETFADHREGILKTAKVLVEDTKVLVQNAAGSQEKLAQAAQSSVATITRLADVVKLGAASLGAEDPETQVVLINAVKDVAKALGDLISATKAAAGKVGDDPAVWQLKNSAKVMVTNVTSLLKTVKAVEDEATKGTRALEATTEHIRQELAVFCSPEPPAKTSTPEDFIRMTKGITMATAKAVAAGNSCRQEDVIATANLSRRAIADMLRACKEAAYHPEVAPDVRLRALHYGRECANGYLELLDHVLLTLQKPSPELKQQLTGHSKRVAGSVTELIQAAEAMKGTEWVDPEDPTVIAENELLGAAAAIEAAAKKLEQLKPRAKPKEADESLNFEEQILEAAKSIAAATSALVKAASAAQRELVAQGKVGAIPANALDDGQWSQGLISAARMVAAATNNLCEAANAAVQGHASQEKLISSAKQVAASTAQLLVACKVKADQDSEAMKRLQAAGNAVKRASDNLVKAAQKAAAFEEQENETVVVKEKMVGGIAQIIAAQEEMLRKERELEEARKKLAQIRQQQYKFLPSELRDEH

6) >gi|302699237|ref|NP_886553.3| eukaryotic translation initiation factor 4 gamma 1 isoform 1 [Homo sapiens] (175.4 kD)

MNKAPQSTGPPPAPSPGLPQPAFPPGQTAPVVFSTPQATQMNTPSQPRQHFYPSRAQPPSSAASRVQSAAPARPGPAAHVYPAGSQVMMIPSQISYPASQGAYYIPGQGRSTYVVPTQQYPVQPGAPGFYPGASPTEFGTYAGAYYPAQGVQQFPTGVAPAPVLMNQPPQIAPKRERKTIRIRDPNQGGKDITEEIMSGARTASTPTPPQTGGGLEPQANGETPQVAVIVRPDDRSQGAIIADRPGLPGPEHSPSESQPSSPSPTPSPSPVLEPGSEPNLAVLSIPGDTMTTIQMSVEESTPISRETGEPYRLSPEPTPLAEPILEVEVTLSKPVPESEFSSSPLQAPTPLASHTVEIHEPNGMVPSEDLEPEVESSPELAPPPACPSESPVPIAPTAQPEELLNGAPSPPAVDLSPVSEPEEQAKEVTASMAPPTIPSATPATAPSATSPAQEEEMEEEEEEEEGEAGEAGEAESEKGGEELLPPESTPIPANLSQNLEAAAATQVAVSVPKRRRKIKELNKKEAVGDLLDAFKEANPAVPEVENQPPAGSNPGPESEGSGVPPRPEEADETWDSKEDKIHNAENIQPGEQKYEYKSDQWKPLNLEEKKRYDREFLLGFQFIFASMQKPEGLPHISDVVLDKANKTPLRPLDPTRLQGINCGPDFTPSFANLGRTTLSTRGPPRGGPGGELPRGPQAGLGPRRSQQGPRKEPRKIIATVLMTEDIKLNKAEKAWKPSSKRTAADKDRGEEDADGSKTQDLFRRVRSILNKLTPQMFQQLMKQVTQLAIDTEERLKGVIDLIFEKAISEPNFSVAYANMCRCLMALKVPTTEKPTVTVNFRKLLLNRCQKEFEKDKDDDEVFEKKQKEMDEAATAEERGRLKEELEEARDIARRRSLGNIKFIGELFKLKMLTEAIMHDCVVKLLKNHDEESLECLCRLLTTIGKDLDFEKAKPRMDQYFNQMEKIIKEKKTSSRIRFMLQDVLDLRGSNWVPRRGDQGPKTIDQIHKEAEMEEHREHIKVQQLMAKGSDKRRGGPPGPPISRGLPLVDDGGWNTVPISKGSRPIDTSRLTKITKPGSIDSNNQLFAPGGRLSWGKGSSGGSGAKPSDAASEAARPATSTLNRFSALQQAVPTESTDNRRVVQRSSLSRERGEKAGDRGDRLERSERGGDRGDRLDRARTPATKRSFSKEVEERSRERPSQPEGLRKAASLTEDRDRGRDAVKREAALPPVSPLKAALSEEELEKKSKAIIEEYLHLNDMKEAVQCVQELASPSLLFIFVRHGVESTLERSAIAREHMGQLLHQLLCAGHLSTAQYYQGLYEILELAEDMEIDIPHVWLYLAELVTPILQEGGVPMGELFREITKPLRPLGKAASLLLEILGLLCKSMGPKKVGTLWREAGLSWKEFLPEGQDIGAFVAEQKVEYTLGEESEAPGQRALPSEELNRQLEKLLKEGSSNQRVFDWIEANLSEQQIVSNTLVRALMTAVCYSAIIFETPLRVDVAVLKARAKLLQKYLCDEQKELQALYALQALVVTLEQPPNLLRMFFDALYDEDVVKEDAFYSWESSKDPAEQQGKGVALKSVTAFFKWLREAEEESDHN

7) >gi|111118970|ref|NP_542411.2| collagen alpha-2(XI) chain isoform 1 preproprotein [Homo sapiens] (171.7 kD)

MERCSRCHRLLLLLPLVLGLSAAPGWAGAPPVDVLRALRFPSLPDGVRRAKGICPADVAYRVARPAQLSAPTRQLFPGGFPKDFSLLTVVRTRPGLQAPLLTLYSAQGVRQLGLELGRPVRFLYEDQTGRPQPPSQPVFRGLSLADGKWHRVAVAVKGQSVTLIVDCKKRVTRPLPRSARPVLDTHGVIIFGARILDEEVFEGDVQELAIVPGVQAAYESCEQKELECEGGQRERPQNQQPHRAQRSPQQQPSRLHRPQNQEPQSQPTESLYYDYEPPYYDVMTTGTTPDYQDPTPGEEEEILESSLLPPLEEEQTDLQVPPTADRFQAEEYGEGGTDPPEGPYDYTYGYGDDYREETELGPALSAETAHSGAAAHGPRGLKGEKGEPAVLEPGMLVEGPPGPEGPAGLIGPPGIQGNPGPVGDPGERGPPGRAGLPGSDGAPGPPGTSLMLPFRFGSGGGDKGPVVAAQEAQAQAILQQARLALRGPPGPMGYTGRPGPLGQPGSPGLKGESGDLGPQGPRGPQGLTGPPGKAGRRGRAGADGARGMPGDPGVKGDRGFDGLPGLPGEKGHRGDTGAQGLPGPPGEDGERGDDGEIGPRGLPGESGPRGLLGPKGPPGIPGPPGVRGMDGPQGPKGSLGPQGEPGPPGQQGTPGTQGLPGPQGAIGPHGEKGPQGKPGLPGMPGSDGPPGHPGKEGPPGTKGNQGPSGPQGPLGYPGPRGVKGVDGIRGLKGHKGEKGEDGFPGFKGDIGVKGDRGEVGVPGSRGEDGPEGPKGRTGPTGDPGPPGLMGEKGKLGVPGLPGYPGRQGPKGSLGFPGFPGASGEKGARGLSGKSGPRGERGPTGPRGQRGPRGATGKSGAKGTSGGDGPHGPPGERGLPGPQGPNGFPGPKGPPGPPGKDGLPGHPGQRGEVGFQGKTGPPGPPGVVGPQGAAGETGPMGERGHPGPPGPPGEQGLPGTAGKEGTKGDPGPPGAPGKDGPAGLRGFPGERGLPGTAGGPGLKGNEGPSGPPGPAGSPGERGAAGSGGPIGPPGRPGPQGPPGAAGEKGVPGEKGPIGPTGRDGVQGPVGLPGPAGPPGVAGEDGDKGEVGDPGQKGTKGNKGEHGPPGPPGPIGPVGQPGAAGADGEPGARGPQGHFGAKGDEGTRGFNGPPGPIGLQGLPGPSGEKGETGDVGPMGPPGPPGPRGPAGPNGADGPQGPPGGVGNLGPPGEKGEPGESGSPGIQGEPGVKGPRGERGEKGESGQPGEPGPPGPKGPTGDDGPKGNPGPVGFPGDPGPPGEGGPRGQDGAKGDRGEDGEPGQPGSPGPTGENGPPGPLGKRGPAGSPGSEGRQGGKGAKGDPGAIGAPGKTGPVGPAGPAGKPGPDGLRGLPGSVGQQGRPGATGQAGPPGPVGPPGLPGLRGDAGAKGEKGHPGLIGLIGPPGEQGEKGDRGLPGPQGSPGQKGEMGIPGASGPIGPGGPPGLPGPAGPKGAKGATGPGGPKGEKGVQGPPGHPGPPGEVIQPLPIQMPKKTRRSVDGSRLMQEDEAIPTGGAPGSPGGLEEIFGSLDSLREEIEQMRRPTGTQDSPARTCQDLKLCHPELPDGEYWVDPNQGCARDAFRVFCNFTAGGETCVTPRDDVTQFSYVDSEGSPVGVVQLTFLRLLSVSAHQDVSYPCSGAARDGPLRLRGANEDELSPETSPYVKEFRDGCQTQQGRTVLEVRTPVLEQLPVLDASFSDLGAPPRRGGVLLGPVCFMG

8) >gi|262118216|ref|NP_115627.6| coiled-coil domain-containing protein 88B precursor [Homo sapiens] (164.7 kD)

MEGGKGPRLRDFLSGSLATWALGLAGLVGEAEDSEGEEEEEEEEPPLWLEKRFLRLSDGALLLRVLGIIAPSSRGGPRMLRGLDGPAAWRVWNLNHLWGRLRDFYQEELQLLILSPPPDLQTLGFDPLSEEAVEQLEGVLRLLLGASVQCEHRELFIRHIQGLSLEVQSELAAAIQEVTQPGAGVVLALSGPDPGELAPAELEMLSRSLMGTLSKLARERDLGAQRLAELLLEREPLCLRPEAPSRAPAEGPSHHLALQLANAKAQLRRLRQELEEKAELLLDSQAEVQGLEAEIRRLRQEAQALSGQAKRAELYREEAEALRERAGRLPRLQEELRRCRERLQAAEAYKSQLEEERVLSGVLEASKALLEEQLEAARERCARLHETQRENLLLRTRLGEAHAELDSLRHQVDQLAEENVELELELQRSLEPPPGSPGEAPLAGAAPSLQDEVREAEAGRLRTLERENRELRGLLQVLQGQPGGQHPLLEAPREDPVLPVLEEAPQTPVAFDHSPQGLVQKARDGGPQALDLAPPALDSVLEASAECPQAPDSDPQEAESPLQAAAMDPQASDWSPQESGSPVETQESPEKAGRRSSLQSPASVAPPQGPGTKIQAPQLLGGETEGREAPQGELVPEAWGLRQEGPEHKPGPSEPSSVQLEEQEGPNQGLDLATGQAEAREHDQRLEGTVRDPAWQKPQQKSEGALEVQVWEGPIPGESLASGVAEQEALREEVAQLRRKAEALGDELEAQARKLEAQNTEAARLSKELAQARRAEAEAHREAEAQAWEQARLREAVEAAGQELESASQEREALVEALAAAGRERRQWEREGSRLRAQSEAAEERMQVLESEGRQHLEEAERERREKEALQAELEKAVVRGKELGDRLEHLQRELEQAALERQEFLREKESQHQRYQGLEQRLEAELQAAATSKEEALMELKTRALQLEEELFQLRQGPAGLGPKKRAEPQLVETQNVRLIEVERSNAMLVAEKAALQGQLQHLEGQLGSLQGRAQELLLQSQRAQEHSSRLQAEKSVLEIQGQELHRKLEVLEEEVRAARQSQEETRGQQQALLRDHKALAQLQRRQEAELEGLLVRHRDLKANMRALELAHRELQGRHEQLQAQRASVEAQEVALLAERERLMQDGHRQRGLEEELRRLQSEHDRAQMLLAELSRERGELQGERGELRGRLARLELERAQLEMQSQQLRESNQQLDLSACRLTTQCELLTQLRSAQEEENRQLLAEVQALSRENRELLERSLESRDHLHREQREYLDQLNALRREKQKLVEKIMDQYRVLEPVPLPRTKKGSWLADKVKRLMRPRREGGPPGGLRLGADGAGSTESLGGPPETELPEGREADGTGSPSPAPMRRAQSSLCLRDETLAGGQRRKLSSRFPVGRSSESFSPGDTPRQRFRQRHPGPLGAPVSHSKGPGVGWENSAETLQEHETDANREGPEVQEPEKRPLTPSLSQ

9) >gi|55741719|ref|NP_055621.1| iporin [Homo sapiens] (161.1 kD)

MDSPPKLTGETLIVHHIPLVHCQVPDRQCCGGAGGGGGSTRPNPFCPPELGITQPDQDLGQADSLLFSSLHSAPGGTARSIDSTKSRSRDGRGPGAPKRHNPFLLQEGVGEPGLGDLYDDSIGDSATQQSFHLHGTGQPNFHLSSFQLPPSGPRVGRPWGTTRSRAGVVEGQEQEPVMTLDTQQCGTSHCCRPELEAETMELDECGGPGGSGSGGGASDTSGFSFDQEWKLSSDESPRNPGCSGSGDQHCRCSSTSSQSEAADQSMGYVSDSSCNSSDGVLVTFSTLYNKMHGTPRANLNSAPQSCSDSSFCSHSDPGAFYLDLQPSPFESKMSYESHHPESGGREGGYGCPHASSPELDANCNSYRPHCEPCPAVADLTACFQSQARLVVATQNYYKLVTCDLSSQSSPSPAGSSITSCSEEHTKISPPPGPGPDPGPSQPSEYYLFQKPEVQPEEQEAVSSSTQAAAAVGPTVLEGQVYTNTSPPNLSTGRQRSRSYDRSLQRSPPVRLGSLERMLSCPVRLSEGPAAMAGPGSPPRRVTSFAELAKGRKKTGGSGSPPLRVSVGDSSQEFSPIQEAQQDRGAPLDEGTCCSHSLPPMPLGPGMDLLGPDPSPPWSTQVCQGPHSSEMPPAGLRATGQGPLAQLMDPGPALPGSPANSHTQRDARARADGGGTESRPVLRYSKEQRPTTLPIQPFVFQHHFPKQLAKARALHSLSQLYSLSGCSRTQQPAPLAAPAAQVSVPAPSGEPQASTPRATGRGARKAGSEPETSRPSPLGSYSPIRSVGPFGPSTDSSASTSCSPPPEQPTATESLPPWSHSCPSAVRPATSQQPQKEDQKILTLTEYRLHGTGSLPPLGSWRSGLSRAESLARGGGEGSMATRPSNANHLSPQALKWREYRRKNPLGPPGLSGSLDRRSQEARLARRNPIFEFPGSLSAASHLNCRLNGQAVKPLPLTCPDFQDPFSLTEKPPAEFCLSPDGSSEAISIDLLQKKGLVKAVNIAVDLIVAHFGTSRDPGVKAKLGNSSVSPNVGHLVLKYLCPAVRAVLEDGLKAFVLDVIIGQRKNMPWSVVEASTQLGPSTKVLHGLYNKVSQFPELTSHTMRFNAFILGLLNIRSLEFWFNHLYNHEDIIQTHYQPWGFLSAAHTVCPGLFEELLLLLQPLALLPFSLDLLFQHRLLQSGQQQRQHKELLRVSQDLLLSAHSTLQLARARGQEGPGDVDRAAQGERVKGVGASEGGEEEEEEEETEEVAEAAGGSGRARWARGGQAGWWYQLMQSSQVYIDGSIEGSRFPRGSSNSSSEKKKGAGGGGPPQAPPPREGVVEGAEACPASEEALGRERGWPFWMGSPPDSVLAELRRSREREGPAASPAENEEGASEPSPGGIKWGHLFGSRKAQREARPTNRLPSDWLSLDKSMFQLVAQTVGSRREPEPKESLQEPHSPALPSSPPCEVQALCHHLATGPGQLSFHKGDILRVLGRAGGDWLRCSRGPDSGLVPLAYVTLTPTPSPTPGSSQN

10) >gi|358679311|ref|NP_001240626.1| protein LAP2 isoform 1 [Homo sapiens] (158.2 kD)

MTTKRSLFVRLVPCRCLRGEEETVTTLDYSHCSLEQVPKEIFTFEKTLEELYLDANQIEELPKQLFNCQSLHKLSLPDNDLTTLPASIANLINLRELDVSKNGIQEFPENIKNCKVLTIVEASVNPISKLPDGFSQLLNLTQLYLNDAFLEFLPANFGRLTKLQILELRENQLKMLPKTMNRLTQLERLDLGSNEFTEVPEVLEQLSGLKEFWMDANRLTFIPGFIGSLKQLTYLDVSKNNIEMVEEGISTCENLQDLLLSSNSLQQLPETIGSLKNITTLKIDENQLMYLPDSIGGLISVEELDCSFNEVEALPSSIGQLTNLRTFAADHNYLQQLPPEIGSWKNITVLFLHSNKLETLPEEMGDMQKLKVINLSDNRLKNLPFSFTKLQQLTAMWLSDNQSKPLIPLQKETDSETQKMVLTNYMFPQQPRTEDVMFISDNESFNPSLWEEQRKQRAQVAFECDEDKDEREAPPREGNLKRYPTPYPDELKNMVKTVQTIVHRLKDEETNEDSGRDLKPHEDQQDINKDVGVKTSESTTTVKSKVDEREKYMIGNSVQKISEPEAEISPGSLPVTANMKASENLKHIVNHDDVFEESEELSSDEEMKMAEMRPPLIETSINQPKVVALSNNKKDDTKETDSLSDEVTHNSNQNNSNCSSPSRMSDSVSLNTDSSQDTSLCSPVKQTHIDINSKIRQEDENFNSLLQNGDILNSSTEEKFKAHDKKDFNLPEYDLNVEERLVLIEKSVDSTATADDTHKLDHINMNLNKLITNDTFQPEIMERSKTQDIVLGTSFLSINSKEETEHLENGNKYPNLESVNKVNGHSEETSQSPNRTEPHDSDCSVDLGISKSTEDLSPQKSGPVGSVVKSHSITNMEIGGLKIYDILSDNGPQQPSTTVKITSAVDGKNIVRSKSATLLYDQPLQVFTGSSSSSDLISGTKAIFKFDSNHNPEEPNIIRGPTSGPQSAPQIYGPPQYNIQYSSSAAVKDTLWHSKQNPQIDHASFPPQLLPRSESTENQSYAKHSANMNFSNHNNVRANTAYHLHQRLGPARHGEMWAISPNDRLIPAVTRSTIQRQSSVSSTASVNLGDPGSTRRAQIPEGDYLSYREFHSAGRTPPMMPGSQRPLSARTYSIDGPNASRPQSARPSINEIPERTMSVSDFNYSRTSPSKRPNARVGSEHSLLDPPGKSKVPRDWREQVLRHIEAKKLEKKHPQTSSSGDPCQDGIFISGQQNYSSATLSHKDVPPDSLMKMPLSNGQMGQPLRPQANYSQIHHPPQASVARHPSREQLIDYLMLKVAHQPPYTQPHCSPRQGHELAKQEIRVRVEKDPELGFSISGGVGGRGNPFRPDDDGIFVTRVQPEGPASKLLQPGDKIIQANGYSFINIEHGQAVSLLKTFQNTVELIIVREVSS

11) >gi|31621305|ref|NP_573566.2| leucine-rich PPR motif-containing protein, mitochondrial precursor [Homo sapiens] (157.8 kD)

MAALLRSARWLLRAGAAPRLPLSLRLLPGGPGRLHAASYLPAARAGPVAGGLLSPARLYAIAAKEKDIQEESTFSSRKISNQFDWALMRLDLSVRRTGRIPKKLLQKVFNDTCRSGGLGGSHALLLLRSCGSLLPELKLEERTEFAHRIWDTLQKLGAVYDVSHYNALLKVYLQNEYKFSPTDFLAKMEEANIQPNRVTYQRLIASYCNVGDIEGASKILGFMKTKDLPVTEAVFSALVTGHARAGDMENAENILTVMRDAGIEPGPDTYLALLNAYAEKGDIDHVKQTLEKVEKSELHLMDRDLLQIIFSFSKAGYPQYVSEILEKVTCERRYIPDAMNLILLLVTEKLEDVALQILLACPVSKEDGPSVFGSFFLQHCVTMNTPVEKLTDYCKKLKEVQMHSFPLQFTLHCALLANKTDLAKALMKAVKEEGFPIRPHYFWPLLVGRRKEKNVQGIIEILKGMQELGVHPDQETYTDYVIPCFDSVNSARAILQENGCLSDSDMFSQAGLRSEAANGNLDFVLSFLKSNTLPISLQSIRSSLLLGFRRSMNINLWSEITELLYKDGRYCQEPRGPTEAVGYFLYNLIDSMSDSEVQAKEEHLRQYFHQLEKMNVKIPENIYRGIRNLLESYHVPELIKDAHLLVESKNLDFQKTVQLTSSELESTLETLKAENQPIRDVLKQLILVLCSEENMQKALELKAKYESDMVTGGYAALINLCCRHDKVEDALNLKEEFDRLDSSAVLDTGKYVGLVRVLAKHGKLQDAINILKEMKEKDVLIKDTTALSFFHMLNGAALRGEIETVKQLHEAIVTLGLAEPSTNISFPLVTVHLEKGDLSTALEVAIDCYEKYKVLPRIHDVLCKLVEKGETDLIQKAMDFVSQEQGEMVMLYDLFFAFLQTGNYKEAKKIIETPGIRARSARLQWFCDRCVANNQVETLEKLVELTQKLFECDRDQMYYNLLKLYKINGDWQRADAVWNKIQEENVIPREKTLRLLAEILREGNQEVPFDVPELWYEDEKHSLNSSSASTTEPDFQKDILIACRLNQKKGAYDIFLNAKEQNIVFNAETYSNLIKLLMSEDYFTQAMEVKAFAETHIKGFTLNDAANSRLIITQVRRDYLKEAVTTLKTVLDQQQTPSRLAVTRVIQALAMKGDVENIEVVQKMLNGLEDSIGLSKMVFINNIALAQIKNNNIDAAIENIENMLTSENKVIEPQYFGLAYLFRKVIEEQLEPAVEKISIMAERLANQFAIYKPVTDFFLQLVDAGKVDDARALLQRCGAIAEQTPILLLFLLRNSRKQGKASTVKSVLELIPELNEKEEAYNSLMKSYVSEKDVTSAKALYEHLTAKNTKLDDLFLKRYASLLKYAGEPVPFIEPPESFEFYAQQLRKLRENSS

12) >gi|66392157|ref|NP_001013860.1| leucine-rich repeat-containing protein 16C [Homo sapiens] (154.6 kD)

MAQTPDGISCELRGEITRFLWPKEVELLLKTWLPGEGAVQNHVLALLRWRAYLLHTTCLPLRVDCTFSYLEVQAMALQETPPQVTFELESLRELVLEFPGVAALEQLAQHVAAAIKKVFPRSTLGKLFRRPTPASMLARLERSSPSESTDPCSPCGGFLETYEALCDYNGFPFREEIQWDVDTIYHRQGCRHFSLGDFSHLGSRDLALSVAALSYNLWFRCLSCVDMKLSLEVSEQILHMMSQSSHLEELVLETCSLRGDFVRRLAQALAGHSSSGLRELSLAGNLLDDRGMTALSRHLERCPGALRRLSLAQTGLTPRGMRALGRALATNAAFDSTLTHLDLSGNPGALGASEDSGGLYSFLSRPNVLSFLNLAGTDTALDTVRGCSVGGWMTGRADWRAGRGGLGPPAGVANSLPPQLFAAVSRGCCTSLTHLDASRNVFSRTKSRAAPAALQLFLSRARTLRHLGLAGCKLPPDALRALLDGLALNTHLRDLHLDLSACELRSAGAQVIQDLVCDAGAVSSLDLADNGFGSDMVTLVLAIGRSRSLRHVALGRNFNVRCKETLDDVLHRIVQLMQDDDCPLQSLSVAESRLKLGASVLLRALATNPNLTALDISGNAMGDAGAKLLAKALRVNSRLRSVVWDRNHTSALGLLDVAQALEQNHSLKAMPLPLNDVAQAQRSRPELTARAVHQIQACLLRNNRADPASSDHTTRLQPLGLVSDPSEQEVNELCQSVQEHVELLGCGAGPQGEAAVRQAEDAIQNANFSLSILPILYEAGSSPSHHWQLGQKLEGLLRQVGEVCRQDIQDFTQATLDTARSLCPQMLQGSSWREQLEGVLAGSRGLPELLPEQLLQDAFTRLRDMRLSITGTLAESIVAQALAGLSAARDQLVESLAQQATVTMPPALPAPDGGEPSLLEPGELEGLFFPEEKEEEKEKDDSPPQKWPELSHGLHLVPFIHSAAEEAEPEPELAAPGEDAEPQAGPSARGSPSPAAPGPPAGPLPRMDLPLAGQPLRHPTRARPRPRRQHHHRPPPGGPQVPPALPQEGNGLSARVDEGVEEFFSKRLIQQDRLWAPEEDPATEGGATPVPRTLRKKLGTLFAFKKPRSTRGPRTDLETSPGAAPRTRKTTFGDLLRPPTRPSRGEELGGAEGDTSSPDPAGRSRPRYTRDSKAYSMILLPAEEEATLGARPDKRRPLERGETELAPSFEQRVQVMLQRIGVSRGSGGAEGKRKQSKDGEIKKAGSDGDIMDSSTEAPPISIKSRTHSVSADPSCRPGPGSQGPESATWKTLGQQLNAELRSRGWGQQDGPGPPSPGQSPSPCRTSPSPDSLGLPEDPCLGPRNEDGQLRPRPLSAGRRAVSVHEDQLQAPAERPLRLQRSPVLKRRPKLEAPPSPSLGSGLGTEPLPPQPTEPSSPERSPPSPATDQRGGGPNP

13) >gi|124028529|ref|NP_004810.2| symplekin [Homo sapiens] (141.1 kD)

MASGSGDSVTRRSVASQFFTQEEGPGIDGMTTSERVVDLLNQAALITNDSKITVLKQVQELIINKDPTLLDNFLDEIIAFQADKSIEVRKFVIGFIEEACKRDIELLLKLIANLNMLLRDENVNVVKKAILTMTQLYKVALQWMVKSRVISELQEACWDMVSAMAGDIILLLDSDNDGIRTHAIKFVEGLIVTLSPRMADSEIPRRQEHDISLDRIPRDHPYIQYNVLWEEGKAALEQLLKFMVHPAISSINLTTALGSLANIARQRPMFMSEVIQAYETLHANLPPTLAKSQVSSVRKNLKLHLLSVLKHPASLEFQAQITTLLVDLGTPQAEIARNMPSSKDTRKRPRDDSDSTLKKMKLEPNLGEDDEDKDLEPGPSGTSKASAQISGQSDTDITAEFLQPLLTPDNVANLVLISMVYLPEAMPASFQAIYTPVESAGTEAQIKHLARLMATQMTAAGLGPGVEQTKQCKEEPKEEKVVKTESVLIKRRLSAQGQAISVVGSLSSMSPLEEEAPQAKRRPEPIIPVTQPRLAGAGGRKKIFRLSDVLKPLTDAQVEAMKLGAVKRILRAEKAVACSGAAQVRIKILASLVTQFNSGLKAEVLSFILEDVRARLDLAFAWLYQEYNAYLAAGASGSLDKYEDCLIRLLSGLQEKPDQKDGIFTKVVLEAPLITESALEVVRKYCEDESRTYLGMSTLRDLIFKRPSRQFQYLHVLLDLSSHEKDKVRSQALLFIKRMYEKEQLREYVEKFALNYLQLLVHPNPPSVLFGADKDTEVAAPWTEETVKQCLYLYLALLPQNHKLIHELAAVYTEAIADIKRTVLRVIEQPIRGMGMNSPELLLLVENCPKGAETLVTRCLHSLTDKVPPSPELVKRVRDLYHKRLPDVRFLIPVLNGLEKKEVIQALPKLIKLNPIVVKEVFNRLLGTQHGEGNSALSPLNPGELLIALHNIDSVKCDMKSIIKATNLCFAERNVYTSEVLAVVMQQLMEQSPLPMLLMRTVIQSLTMYPRLGGFVMNILSRLIMKQVWKYPKVWEGFIKCCQRTKPQSFQVILQLPPQQLGAVFDKCPELREPLLAHVRSFTPHQQAHIPNSIMTILEASGKQEPEAKEAPAGPLEEDDLEPLTLAPAPAPRPPQDLIGLRLAQEKALKRQLEEEQKLKPGGVGAPSSSSPSPSPSARPGPPPSEEAMDFREEGPECETPGIFISMDDDSGLTEAALLDSSLEGPLPKETAAGGLTLKEERSPQTLAPVGEDAMKTPSPAAEDAREPEAKGNS

14) >gi|149999380|ref|NP_071934.3| inverted formin-2 isoform 1 [Homo sapiens] (135.5 kD)

MSVKEGAQRKWAALKEKLGPQDSDPTEANLESADPELCIRLLQMPSVVNYSGLRKRLEGSDGGWMVQFLEQSGLDLLLEALARLSGRGVARISDALLQLTCVSCVRAVMNSRQGIEYILSNQGYVRQLSQALDTSNVMVKKQVFELLAALCIYSPEGHVLTLDALDHYKTVCSQQYRFSIVMNELSGSDNVPYVVTLLSVINAVILGPEDLRARTQLRNEFIGLQLLDVLARLRDLEDADLLIQLEAFEEAKAEDEEELLRVSGGVDMSSHQEVFASLFHKVSCSPVSAQLLSVLQGLLHLEPTLRSSQLLWEALESLVNRAVLLASDAQECTLEEVVERLLSVKGRPRPSPLVKAHKSVQANLDQSQRGSSPQNTTTPKPSVEGQQPAAAAACEPVDHAQSESILKVSQPRALEQQASTPPPPPPPPLLPGSSAEPPPPPPPPPLPSVGAKALPTAPPPPPLPGLGAMAPPAPPLPPPLPGSCEFLPPPPPPLPGLGCPPPPPPLLPGMGWGPPPPPPPLLPCTCSPPVAGGMEEVIVAQVDHGLGSAWVPSHRRVNPPTLRMKKLNWQKLPSNVAREHNSMWASLSSPDAEAVEPDFSSIERLFSFPAAKPKEPTMVAPRARKEPKEITFLDAKKSLNLNIFLKQFKCSNEEVAAMIRAGDTTKFDVEVLKQLLKLLPEKHEIENLRAFTEERAKLASADHFYLLLLAIPCYQLRIECMLLCEGAAAVLDMVRPKAQLVLAACESLLTSRQLPIFCQLILRIGNFLNYGSHTGDADGFKISTLLKLTETKSQQNRVTLLHHVLEEAEKSHPDLLQLPRDLEQPSQAAGINLEIIRSEASSNLKKLLETERKVSASVAEVQEQYTERLQASISAFRALDELFEAIEQKQRELADYLCEDAQQLSLEDTFSTMKAFRDLFLRALKENKDRKEQAAKAERRKQQLAEEEARRPRGEDGKPVRKGPGKQEEVCVIDALLADIRKGFQLRKTARGRGDTDGGSKAASMDPPRATEPVATSNPAGDPVGSTRCPASEPGLDATTASESRGWDLVDAVTPGPQPTLEQLEEGGPRPLERRSSWYVDASDVLTTEDPQCPQPLEGAWPVTLGDAQALKPLKFSSNQPPAAGSSRQDAKDPTSLLGVLQAEADSTSEGLEDAVHSRGARPPAAGPGGDEDEDEEDTAPESALDTSLDKSFSEDAVTDSSGSGTLPRARGRASKGTGKRRKKRPSRSQEEVPPDSDDNKTKKLCVIQ

15) >gi|24431935|ref|NP_065393.1| reticulon-4 isoform A [Homo sapiens] (129.9 kD)

MEDLDQSPLVSSSDSPPRPQPAFKYQFVREPEDEEEEEEEEEEDEDEDLEELEVLERKPAAGLSAAPVPTAPAAGAPLMDFGNDFVPPAPRGPLPAAPPVAPERQPSWDPSPVSSTVPAPSPLSAAAVSPSKLPEDDEPPARPPPPPPASVSPQAEPVWTPPAPAPAAPPSTPAAPKRRGSSGSVDETLFALPAASEPVIRSSAENMDLKEQPGNTISAGQEDFPSVLLETAASLPSLSPLSAASFKEHEYLGNLSTVLPTEGTLQENVSEASKEVSEKAKTLLIDRDLTEFSELEYSEMGSSFSVSPKAESAVIVANPREEIIVKNKDEEEKLVSNNILHNQQELPTALTKLVKEDEVVSSEKAKDSFNEKRVAVEAPMREEYADFKPFERVWEVKDSKEDSDMLAAGGKIESNLESKVDKKCFADSLEQTNHEKDSESSNDDTSFPSTPEGIKDRSGAYITCAPFNPAATESIATNIFPLLGDPTSENKTDEKKIEEKKAQIVTEKNTSTKTSNPFLVAAQDSETDYVTTDNLTKVTEEVVANMPEGLTPDLVQEACESELNEVTGTKIAYETKMDLVQTSEVMQESLYPAAQLCPSFEESEATPSPVLPDIVMEAPLNSAVPSAGASVIQPSSSPLEASSVNYESIKHEPENPPPYEEAMSVSLKKVSGIKEEIKEPENINAALQETEAPYISIACDLIKETKLSAEPAPDFSDYSEMAKVEQPVPDHSELVEDSSPDSEPVDLFSDDSIPDVPQKQDETVMLVKESLTETSFESMIEYENKEKLSALPPEGGKPYLESFKLSLDNTKDTLLPDEVSTLSKKEKIPLQMEELSTAVYSNDDLFISKEAQIRETETFSDSSPIEIIDEFPTLISSKTDSFSKLAREYTDLEVSHKSEIANAPDGAGSLPCTELPHDLSLKNIQPKVEEKISFSDDFSKNGSATSKVLLLPPDVSALATQAEIESIVKPKVLVKEAEKKLPSDTEKEDRSPSAIFSAELSKTSVVDLLYWRDIKKTGVVFGASLFLLLSLTVFSIVSVTAYIALALLSVTISFRIYKGVIQAIQKSDEGHPFRAYLESEVAISEELVQKYSNSALGHVNCTIKELRRLFLVDDLVDSLKFAVLMWVFTYVGALFNGLTLLILALISLFSVPVIYERHQAQIDHYLGLANKNVKDAMAKIQAKIPGLKRKAE

16) >gi|62868215|ref|NP_000219.2| laminin subunit beta-3 precursor [Homo sapiens] (129.5 kD)

MRPFFLLCFALPGLLHAQQACSRGACYPPVGDLLVGRTRFLRASSTCGLTKPETYCTQYGEWQMKCCKCDSRQPHNYYSHRVENVASSSGPMRWWQSQNDVNPVSLQLDLDRRFQLQEVMMEFQGPMPAGMLIERSSDFGKTWRVYQYLAADCTSTFPRVRQGRPQSWQDVRCQSLPQRPNARLNGGKVQLNLMDLVSGIPATQSQKIQEVGEITNLRVNFTRLAPVPQRGYHPPSAYYAVSQLRLQGSCFCHGHADRCAPKPGASAGPSTAVQVHDVCVCQHNTAGPNCERCAPFYNNRPWRPAEGQDAHECQRCDCNGHSETCHFDPAVFAASQGAYGGVCDNCRDHTEGKNCERCQLHYFRNRRPGASIQETCISCECDPDGAVPGAPCDPVTGQCVCKEHVQGERCDLCKPGFTGLTYANPQGCHRCDCNILGSRRDMPCDEESGRCLCLPNVVGPKCDQCAPYHWKLASGQGCEPCACDPHNSLSPQCNQFTGQCPCREGFGGLMCSAAAIRQCPDRTYGDVATGCRACDCDFRGTEGPGCDKASGRCLCRPGLTGPRCDQCQRGYCNRYPVCVACHPCFQTYDADLREQALRFGRLRNATASLWSGPGLEDRGLASRILDAKSKIEQIRAVLSSPAVTEQEVAQVASAILSLRRTLQGLQLDLPLEEETLSLPRDLESLDRSFNGLLTMYQRKREQFEKISSADPSGAFRMLSTAYEQSAQAAQQVSDSSRLLDQLRDSRREAERLVRQAGGGGGTGSPKLVALRLEMSSLPDLTPTFNKLCGNSRQMACTPISCPGELCPQDNGTACGSRCRGVLPRAGGAFLMAGQVAEQLRGFNAQLQRTRQMIRAAEESASQIQSSAQRLETQVSASRSQMEEDVRRTRLLIQQVRDFLTDPDTDAATIQEVSEAVLALWLPTDSATVLQKMNEIQAIAARLPNVDLVLSQTKQDIARARRLQAEAEEARSRAHAVEGQVEDVVGNLRQGTVALQEAQDTMQGTSRSLRLIQDRVAEVQQVLRPAEKLVTSMTKQLGDFWTRMEELRHQARQQGAEAVQAQQLAEGASEQALSAQEGFERIKQKYAELKDRLGQSSMLGEQGARIQSVKTEAEELFGETMEMMDRMKDMELELLRGSQAIMLRSADLTGLEKRVEQIRDHINGRVLYYATCK

17) >gi|578831368|ref|XP_006722131.1| PREDICTED: formin-like protein 1 isoform X7 [Homo sapiens] (121.8 kD)

MGNAAGSAEQPAGPAAPPPKQPAPPKQPMPAAGELEERFNRALNCMNLPPDKVQLLSQYDNEKKWELICDQERFQVKNPPAAYIQKLKSYVDTGGVSRKVAADWMSNLGFKRRVQESTQVLRELETSLRTNHIGWVQEFLNEENRGLDVLLEYLAFAQCSVTYDMESTDNGASNSEKNKPLEQSVEDLSKGPPSSVPKSRHLTIKLTPAHSRKALRNSRIVSQKDDVHVCIMCLRAIMNYQSGFSLVMNHPACVNEIALSLNNKNPRTKALVLELLAAVCLVRGGHDIILAAFDNFKEVCGEQHRFEKLMEYFRNEDSNIDFMVACMQFINIVVHSVENMNFRVFLQYEFTHLGLDLYLERLRLTESDKLQVQIQAYLDNIFDVGALLEDTETKNAVLEHMEELQEQVALLTERLRDAENESMAKIAELEKQLSQARKELETLRERFSESTAMGPSRRPPEPEKAPPAAPTRPSALELKVEELEEKGLIRILRGPGDAVSIEILPVAVATPSGGDAPTPGVPTGSPSPDLAPAAEPAPGAAPPPPPPLPGLPSPQEAPPSAPPQAPPLPGSPEPPPAPPLPGDLPPPPPPPPPPPGTDGPVPPPPPPPPPPPGGPPDALGRRDSELGPGVKAKKPIQTKFRMPLLNWVALKPSQITGTVFTELNDEKVLQELDMSDFEEQFKTKSQGPSLDLSALKSKAAQKAPSKATLIEANRAKNLAITLRKGNLGAERICQAIEAYDLQALGLDFLELLMRFLPTEYERSLITRFEREQRPMEELSEEDRFMLCFSRIPRLPERMTTLTFLGNFPDTAQLLMPQLNAIIAASMSIKSSDKLRQILEIVLAFGNYMNSSKRGAAYGFRLQSLDALLEMKSTDRKQTLLHYLVKVIAEKYPQLTGFHSDLHFLDKAGSVSLDSVLADVRSLQRGLELTQREFVRQDDCMVLKEFLRANSPTMDKLLADSKTAQEAFESVVEYFGENPKTTSPGLFFSLFSRFIKAYKKAEQEVEQWKKEAAAQEAGADTPGKGEPPAPKSPPKARRPQMDLISELKRRQQKEPLIYESDRDGAIEDIITVIKTVPFTARTGKRTSRLLCEASLGEEMPL

18) >gi|134133226|ref|NP_001077007.1| POTE ankyrin domain family member E [Homo sapiens] (121.3 kD)

MVVEVDSMPAASSVKKPFGLRSKMGKWCCRCFPCYRESGKSNVGTSGDHDDSAMKTLRSKMGKWCHHCFPCCRGSGKSNVGASGDHDDSAMKTLRNKMGKWCCHCFPCCRGSGKSKVGAWGDYDDSAFMEPRYHVRGEDLDKLHRAAWWGKVPRKDLIVMLRDTDVNKKDKQKRTALHLASANGNSEVVKLLLDRRCQLNVLDNKKRTALIKAVQCQEDECALMLLEHGTDPNIPDEYGNTTLHYAIYNEDKLMAKALLLYGADIESKNKHGLTPLLLGVHEQKQQVVKFLIKKKANLNALDRYGRTALILAVCCGSASIVSLLLEQNIDVSSQDLSGQTAREYAVSSHHHVICQLLSDYKEKQMLKISSENSNPEQELKLTSEEESQRFKGSENSQPEKMSQELEINKDGDREVEEEMKKHESNNVGLLENLTNGVTAGNGDNGLIPQRKSRTPENQQFPDNESEEYHRICELLSDYKEKQMPKYSSENSNPEQDLKLTSEEESQRLKGSENGQPEKRSQEPEINKDGDRELENFMAIEEMKKHGSTHVGFPENLTNGATAGNGDDGLIPPRKSRTPESQQFPDTENEEYHSDEQNDTQKQFCEEQNTGILHDEILIHEEKQIEVVEKMNSELSLSCKKEKDVLHENSTLREEIAMLRLELDTMKHQSQLREKKYLEDIESVKKKNDNLLKALQLNELTMDDDTAVLVIDNGSGMCKAGFAGDDAPRAVFPSIVGRPRQQGMMGGMHQKESYVGKEAQSKRGILTLKYPMEHGIITNWDDMEKIWHHTFYNELRVAPEEHPILLTEAPLNPKANREKMTQIMFETFNTPAMYVAIQAVPSLYTSGRTTGIVMDSGDGVTHTVPIYEGNALPHATLRLDLAGRELPDYLMKILTERGYRFTTMAEREIVRDIKEKLCYVALDFEQEMATAASSSSLEKSYELPDGQVITIGNERFRCPEALFQPCFLGMESCGIHETTFNSIMKSDVDIRKDLYTNTVLSGGTTMYPGMAHRMQKEIAALAPSMMKIRIIAPPKRKYSVWVGGSILASLSTFQQMWISKQEYDESGPSIVHRKCF

19) >gi|149158692|ref|NP_004630.3| large proline-rich protein BAG6 isoform a [Homo sapiens] (119.3 kD)

MEPNDSTSTAVEEPDSLEVLVKTLDSQTRTFIVGAQMNVKEFKEHIAASVSIPSEKQRLIYQGRVLQDDKKLQEYNVGGKVIHLVERAPPQTHLPSGASSGTGSASATHGGGSPPGTRGPGASVHDRNANSYVMVGTFNLPSDGSAVDVHINMEQAPIQSEPRVRLVMAQHMIRDIQTLLSRMETLPYLQCRGGPQPQHSQPPPQPPAVTPEPVALSSQTSEPVESEAPPREPMEAEEVEERAPAQNPELTPGPAPAGPTPAPETNAPNHPSPAEYVEVLQELQRLESRLQPFLQRYYEVLGAAATTDYNNNHEGREEDQRLINLVGESLRLLGNTFVALSDLRCNLACTPPRHLHVVRPMSHYTTPMVLQQAAIPIQINVGTTVTMTGNGTRPPPTPNAEAPPPGPGQASSVAPSSTNVESSAEGAPPPGPAPPPATSHPRVIRISHQSVEPVVMMHMNIQDSGTQPGGVPSAPTGPLGPPGHGQTLGQQVPGFPTAPTRVVIARPTPPQARPSHPGGPPVSGTLQGAGLGTNASLAQMVSGLVGQLLMQPVLVAQGTPGMAPPPAPATASASAGTTNTATTAGPAPGGPAQPPPTPQPSMADLQFSQLLGNLLGPAGPGAGGSGVASPTITVAMPGVPAFLQGMTDFLQATQTAPPPPPPPPPPPPAPEQQTMPPPGSPSGGAGSPGGLGLESLSPEFFTSVVQGVLSSLLGSLGARAGSSESIAAFIQRLSGSSNIFEPGADGALGFFGALLSLLCQNFSMVDVVMLLHGHFQPLQRLQPQLRSFFHQHYLGGQEPTPSNIRMATHTLITGLEEYVRESFSLVQVQPGVDIIRTNLEFLQEQFNSIAAHVLHCTDSGFGARLLELCNQGLFECLALNLHCLGGQQMELAAVINGRIRRMSRGVNPSLVSWLTTMMGLRLQVVLEHMPVGPDAILRYVRRVGDPPQPLPEEPMEVQGAERASPEPQRENASPAPGTTAEEAMSRGPPPAPEGGSRDEQDGASAETEPWAAAVPPEWVPIIQQDIQSQRKVKPQPPLSDAYLSGMPAKRRKTMQGEGPQLLLSEAVSRAAKAAGARPLTSPESLSRDLEAPEVQESYRQQLRSDIQKRLQEDPNYSPQRFPNAQRAFADDP

20) >gi|23510340|ref|NP_695012.1| ubiquitin-like modifier-activating enzyme 1 [Homo sapiens] (117.8 kD)

MSSSPLSKKRRVSGPDPKPGSNCSPAQSVLSEVPSVPTNGMAKNGSEADIDEGLYSRQLYVLGHEAMKRLQTSSVLVSGLRGLGVEIAKNIILGGVKAVTLHDQGTAQWADLSSQFYLREEDIGKNRAEVSQPRLAELNSYVPVTAYTGPLVEDFLSGFQVVVLTNTPLEDQLRVGEFCHNRGIKLVVADTRGLFGQLFCDFGEEMILTDSNGEQPLSAMVSMVTKDNPGVVTCLDEARHGFESGDFVSFSEVQGMVELNGNQPMEIKVLGPYTFSICDTSNFSDYIRGGIVSQVKVPKKISFKSLVASLAEPDFVVTDFAKFSRPAQLHIGFQALHQFCAQHGRPPRPRNEEDAAELVALAQAVNARALPAVQQNNLDEDLIRKLAYVAAGDLAPINAFIGGLAAQEVMKACSGKFMPIMQWLYFDALECLPEDKEVLTEDKCLQRQNRYDGQVAVFGSDLQEKLGKQKYFLVGAGAIGCELLKNFAMIGLGCGEGGEIIVTDMDTIEKSNLNRQFLFRPWDVTKLKSDTAAAAVRQMNPHIRVTSHQNRVGPDTERIYDDDFFQNLDGVANALDNVDARMYMDRRCVYYRKPLLESGTLGTKGNVQVVIPFLTESYSSSQDPPEKSIPICTLKNFPNAIEHTLQWARDEFEGLFKQPAENVNQYLTDPKFVERTLRLAGTQPLEVLEAVQRSLVLQRPQTWADCVTWACHHWHTQYSNNIRQLLHNFPPDQLTSSGAPFWSGPKRCPHPLTFDVNNPLHLDYVMAAANLFAQTYGLTGSQDRAAVATFLQSVQVPEFTPKSGVKIHVSDQELQSANASVDDSRLEELKATLPSPDKLPGFKMYPIDFEKDDDSNFHMDFIVAASNLRAENYDIPSADRHKSKLIAGKIIPAIATTTAAVVGLVCLELYKVVQGHRQLDSYKNGFLNLALPFFGFSEPLAAPRHQYYNQEWTLWDRFEVQGLQPNGEEMTLKQFLDYFKTEHKLEITMLSQGVSMLYSFFMPAAKLKERLDQPMTEIVSRVSKRKLGRHVRALVLELCCNDESGEDVEVPYVRYTIR

21) >gi|188497758|ref|NP_055662.3| ubiquitin-associated protein 2-like isoform a [Homo sapiens] (114.5 kD)

MMTSVGTNRARGNWEQPQNQNQTQHKQRPQATAEQIRLAQMISDHNDADFEEKVKQLIDITGKNQDECVIALHDCNGDVNRAINVLLEGNPDTHSWEMVGKKKGVSGQKDGGQTESNEEGKENRDRDRDYSRRRGGPPRRGRGASRGREFRGQENGLDGTKSGGPSGRGTERGRRGRGRGRGGSGRRGGRFSAQGMGTFNPADYAEPANTDDNYGNSSGNTWNNTGHFEPDDGTSAWRTATEEWGTEDWNEDLSETKIFTASNVSSVPLPAENVTITAGQRIDLAVLLGKTPSTMENDSSNLDPSQAPSLAQPLVFSNSKQTAISQPASGNTFSHHSMVSMLGKGFGDVGEAKGGSTTGSQFLEQFKTAQALAQLAAQHSQSGSTTTSSWDMGSTTQSPSLVQYDLKNPSDSAVHSPFTKRQAFTPSSTMMEVFLQEKSPAVATSTAAPPPPSSPLPSKSTSAPQMSPGSSDNQSSSPQPAQQKLKQQKKKASLTSKIPALAVEMPGSADISGLNLQFGALQFGSEPVLSDYESTPTTSASSSQAPSSLYTSTASESSSTISSNQSQESGYQSGPIQSTTYTSQNNAQGPLYEQRSTQTRRYPSSISSSPQKDLTQAKNGFSSVQATQLQTTQSVEGATGSAVKSDSPSTSSIPPLNETVSAASLLTTTNQHSSSLGGLSHSEEIPNTTTTQHSSTLSTQQNTLSSSTSSGRTSTSTLLHTSVESEANLHSSSSTFSTTSSTVSAPPPVVSVSSSLNSGSSLGLSLGSNSTVTASTRSSVATTSGKAPPNLPPGVPPLLPNPYIMAPGLLHAYPPQVYGYDDLQMLQTRFPLDYYSIPFPTPTTPLTGRDGSLASNPYSGDLTKFGRGDASSPAPATTLAQPQQNQTQTHHTTQQTFLNPALPPGYSYTSLPYYTGVPGLPSTFQYGPAVFPVAPTSSKQHGVNVSVNASATPFQQPSGYGSHGYNTGVSVTSSNTGVPDISGSVYSKTQQSFEKQGFHSGTPAASFNLPSALGSGGPINPATAAAYPPAPFMHILTPHQQPHSQILHHHLQQDGQTGSGQRSQTSSIPQKPQTNKSAYNSYSWGAN

22) >gi|388240768|ref|NP_001252518.1| reticulon-3 isoform e [Homo sapiens] (112.5 kD)

MAEPSAATQSHSISSSSFGAEPSAPGGGGSPGACPALGTKSCSSSCADSFVSSSSSQPVSLFSTSQEGLSSLCSDEPSSEIMTSSFLSSSEIHNTGLTILHGEKSHVLGSQPILAKEGKDHLDLLDMKKMEKPQGTSNNVSDSSVSLAAGVHCDRPSIPASFPEHPAFLSKKIGQVEEQIDKETKNPNGVSSREAKTALDADDRFTLLTAQKPPTEYSKVEGIYTYSLSPSKVSGDDVIEKDSPESPFEVIIDKAAFDKEFKDSYKESTDDFGSWSVHTDKESSEDISETNDKLFPLRNKEAGRYPMSALLSRQFSHTNAALEEVSRCVNDMHNFTNEILTWDLVPQVKQQTDKSSDCITKTTGLDMSEYNSEIPVVNLKTSTHQKTPVCSIDGSTPITKSTGDWAEASLQQENAITGKPVPDSLNSTKEFSIKGVQGNMQKQDDTLAELPGSPPEKCDSLGSGVATVKVVLPDDHLKDEMDWQSSALGEITEADSSGESDDTVIEDITADTSFENNKIQAEKPVSIPSAVVKTGEREIKEIPSCEREEKTSKNFEELVSDSELHQDQPDILGRSPASEAACSKVPDTNVSLEDVSEVAPEKPITTENPKLPSTVSPNVFNETEFSLNVTTSAYLESLHGKNVKHIDDSSPEDLIAAFTETRDKGIVDSERNAFKAISEKMTDFKTTPPVEVLHENESGGSEIKDIGSKYSEQSKETNGSEPLGVFPTQGTPVASLDLEQEQLTIKALKELGERQVEKSTSAQRDAELPSEEVLKQTFTFAPESWPQRSYDILERNVKNGSDLGISQKPITIRETTRVDAVSSLSKTELVKKHVLARLLTDFSVHDLIFWRDVKKTGFVFGTTLIMLLSLAAFSVISVVSYLILALLSVTISFRIYKSVIQAVQKSEEGHPFKAYLDVDITLSSEAFHNYMNAAMVHINRALKLIIRLFLVEDLVDSLKLAVFMWLMTYVGAVFNGITLLILAELLIFSVPIVYEKYKTQIDHYVGIARDQTKSIVEKIQAKLPGIAKKKAE

23) >gi|22748667|ref|NP_689509.1| sodium/potassium-transporting ATPase subunit alpha-3 isoform 1 [Homo sapiens] (111.7 kD)

MGDKKDDKDSPKKNKGKERRDLDDLKKEVAMTEHKMSVEEVCRKYNTDCVQGLTHSKAQEILARDGPNALTPPPTTPEWVKFCRQLFGGFSILLWIGAILCFLAYGIQAGTEDDPSGDNLYLGIVLAAVVIITGCFSYYQEAKSSKIMESFKNMVPQQALVIREGEKMQVNAEEVVVGDLVEIKGGDRVPADLRIISAHGCKVDNSSLTGESEPQTRSPDCTHDNPLETRNITFFSTNCVEGTARGVVVATGDRTVMGRIATLASGLEVGKTPIAIEIEHFIQLITGVAVFLGVSFFILSLILGYTWLEAVIFLIGIIVANVPEGLLATVTVCLTLTAKRMARKNCLVKNLEAVETLGSTSTICSDKTGTLTQNRMTVAHMWFDNQIHEADTTEDQSGTSFDKSSHTWVALSHIAGLCNRAVFKGGQDNIPVLKRDVAGDASESALLKCIELSSGSVKLMRERNKKVAEIPFNSTNKYQLSIHETEDPNDNRYLLVMKGAPERILDRCSTILLQGKEQPLDEEMKEAFQNAYLELGGLGERVLGFCHYYLPEEQFPKGFAFDCDDVNFTTDNLCFVGLMSMIDPPRAAVPDAVGKCRSAGIKVIMVTGDHPITAKAIAKGVGIISEGNETVEDIAARLNIPVSQVNPRDAKACVIHGTDLKDFTSEQIDEILQNHTEIVFARTSPQQKLIIVEGCQRQGAIVAVTGDGVNDSPALKKADIGVAMGIAGSDVSKQAADMILLDDNFASIVTGVEEGRLIFDNLKKSIAYTLTSNIPEITPFLLFIMANIPLPLGTITILCIDLGTDMVPAISLAYEAAESDIMKRQPRNPRTDKLVNERLISMAYGQIGMIQALGGFFSYFVILAENGFLPGNLVGIRLNWDDRTVNDLEDSYGQQWTYEQRKVVEFTCHTAFFVSIVVVQWADLIICKTRRNSVFQQGMKNKILIFGLFEETALAAFLSYCPGMDVALRMYPLKPSWWFCAFPYSFLIFVYDEIRKLILRRNPGGWVEKETYY

24) >gi|32967311|ref|NP_004422.2| ephrin type-A receptor 2 precursor [Homo sapiens] (108.2 kD)

MELQAARACFALLWGCALAAAAAAQGKEVVLLDFAAAGGELGWLTHPYGKGWDLMQNIMNDMPIYMYSVCNVMSGDQDNWLRTNWVYRGEAERIFIELKFTVRDCNSFPGGASSCKETFNLYYAESDLDYGTNFQKRLFTKIDTIAPDEITVSSDFEARHVKLNVEERSVGPLTRKGFYLAFQDIGACVALLSVRVYYKKCPELLQGLAHFPETIAGSDAPSLATVAGTCVDHAVVPPGGEEPRMHCAVDGEWLVPIGQCLCQAGYEKVEDACQACSPGFFKFEASESPCLECPEHTLPSPEGATSCECEEGFFRAPQDPASMPCTRPPSAPHYLTAVGMGAKVELRWTPPQDSGGREDIVYSVTCEQCWPESGECGPCEASVRYSEPPHGLTRTSVTVSDLEPHMNYTFTVEARNGVSGLVTSRSFRTASVSINQTEPPKVRLEGRSTTSLSVSWSIPPPQQSRVWKYEVTYRKKGDSNSYNVRRTEGFSVTLDDLAPDTTYLVQVQALTQEGQGAGSKVHEFQTLSPEGSGNLAVIGGVAVGVVLLLVLAGVGFFIHRRRKNQRARQSPEDVYFSKSEQLKPLKTYVDPHTYEDPNQAVLKFTTEIHPSCVTRQKVIGAGEFGEVYKGMLKTSSGKKEVPVAIKTLKAGYTEKQRVDFLGEAGIMGQFSHHNIIRLEGVISKYKPMMIITEYMENGALDKFLREKDGEFSVLQLVGMLRGIAAGMKYLANMNYVHRDLAARNILVNSNLVCKVSDFGLSRVLEDDPEATYTTSGGKIPIRWTAPEAISYRKFTSASDVWSFGIVMWEVMTYGERPYWELSNHEVMKAINDGFRLPTPMDCPSAIYQLMMQCWQQERARRPKFADIVSILDKLIRAPDSLKTLADFDPRVSIRLPSTSGSEGVPFRTVSEWLESIKMQQYTEHFMAAGYTAIEKVVQMTNDDIKRIGVRLPGHQKRIAYSLLGLKDQVNTVGIPI

25) >gi|530418825|ref|XP_005260998.1| PREDICTED: trifunctional purine biosynthetic protein adenosine-3 isoform X1 [Homo sapiens] (107.7 kD)

MAARVLIIGSGGREHTLAWKLAQSHHVKQVLVAPGNAGTACSEKISNTAISISDHTALAQFCKEKKIEFVVVGPEAPLAAGIVGNLRSAGVQCFGPTAEAAQLESSKRFAKEFMDRHGIPTAQWKAFTKPEEACSFILSADFPALVVKASGLAAGKGVIVAKSKEEACKAVQEIMQEKAFGAAGETIVIEELLDGEEVSCLCFTDGKTVAPMPPAQDHKRLLEGDGGPNTGGMGAYCPAPQVSNDLLLKIKDTVLQRTVDGMQQEGTPYTGILYAGIMLTKNGPKVLEFNCRFGDPECQVILPLLKSDLYEVIQSTLDGLLCTSLPVWLENHTALTVVMASKGYPGDYTKGVEITGFPEAQALGLEVFHAGTALKNGKVVTHGGRVLAVTAIRENLISALEEAKKGLAAIKFEGAIYRKDVGFRAIAFLQQPRSLTYKESGVDIAAGNMLVKKIQPLAKATSRSGCKVDLGGFAGLFDLKAAGFKDPLLASGTDGVGTKLKIAQLCNKHDTIGQDLVAMCVNDILAQGAEPLFFLDYFSCGKLDLSVTEAVVAGIAKACGKAGCALLGGETAEMPDMYPPGEYDLAGFAVGAMERDQKLPHLERITEGDVVVGIASSGLHSNGFSLVRKIVAKSSLQYSSPAPDGCGDQTLGDLLLTPTRIYSHSLLPVLRSGHVKAFAHITGGGLLENIPRVLPEKLGVDLDAQTWRIPRVFSWLQQEGHLSEEEMARTFNCGVGAVLVVSKEQTEQILRDIQQHKEEAWVIGSVVARAEGSPRVKVKNLIESMQINGSVLKNGSLTNHFSFEKKKARVAVLISGTGSNLQALIDSTREPNSSAQIDIVISNKAAVAGLDKAERAGIPTRVINHKLYKNRVEFDSAIDLVLEEFSIDIVCLAGFMRILSGPFVQKWNGKMLNIHPSLLPSFKGSNAHEQALETGVTVTGCTVHFVAEDVDAGQIILQEAVPVKRGDTVATLSERVKLAEHKIFPAALQLVASGTVQLGENGKICWVKEE

26) >gi|221316630|ref|NP_001137533.1| coatomer subunit beta [Homo sapiens] (107.1 kD)

MTAAENVCYTLINVPMDSEPPSEISLKNDLEKGDVKSKTEALKKVIIMILNGEKLPGLLMTIIRFVLPLQDHTIKKLLLVFWEIVPKTTPDGRLLHEMILVCDAYRKDLQHPNEFIRGSTLRFLCKLKEAELLEPLMPAIRACLEHRHSYVRRNAVLAIYTIYRNFEHLIPDAPELIHDFLVNEKDASCKRNAFMMLIHADQDRALDYLSTCIDQVQTFGDILQLVIVELIYKVCHANPSERARFIRCIYNLLQSSSPAVKYEAAGTLVTLSSAPTAIKAAAQCYIDLIIKESDNNVKLIVLDRLIELKEHPAHERVLQDLVMDILRVLSTPDLEVRKKTLQLALDLVSSRNVEELVIVLKKEVIKTNNVSEHEDTDKYRQLLVRTLHSCSVRFPDMAANVIPVLMEFLSDNNEAAAADVLEFVREAIQRFDNLRMLIVEKMLEVFHAIKSVKIYRGALWILGEYCSTKEDIQSVMTEIRRSLGEIPIVESEIKKEAGELKPEEEITVGPVQKLVTEMGTYATQSALSSSRPTKKEEDRPPLRGFLLDGDFFVAASLATTLTKIALRYVALVQEKKKQNSFVAEAMLLMATILHLGKSSLPKKPITDDDVDRISLCLKVLSECSPLMNDIFNKECRQSLSHMLSAKLEEEKLSQKKESEKRNVTVQPDDPISFMQLTAKNEMNCKEDQFQLSLLAAMGNTQRKEAADPLASKLNKVTQLTGFSDPVYAEAYVHVNQYDIVLDVLVVNQTSDTLQNCTLELATLGDLKLVEKPSPLTLAPHDFANIKANVKVASTENGIIFGNIVYDVSGAASDRNCVVLSDIHIDIMDYIQPATCTDAEFRQMWAEFEWENKVTVNTNMVDLNDYLQHILKSTNMKCLTPEKALSGYCGFMAANLYARSIFGEDALANVSIEKPIHQGPDAAVTGHIRIRAKSQGMALSLGDKINLSQKKTSI

27) >gi|578834952|ref|XP_006723469.1| PREDICTED: alpha-actinin-4 isoform X3 [Homo sapiens] (104.8 kD)

MVDYHAANQSYQYGPSSAGNGAGGGGSMGDYMAQEDDWDRDLLLDPAWEKQQRKTFTAWCNSHLRKAGTQIENIDEDFRDGLKLMLLLEVISGERLPKPERGKMRVHKINNVNKALDFIASKGVKLVSIGAEEIVDGNAKMTLGMIWTIILRFAIQDISVEETSAKEGLLLWCQRKTAPYKNVNVQNFHISWKDGLAFNALIHRHRPELIEYDKLRKDDPVTNLNNAFEVAEKYLDIPKMLDAEDIVGTLRPDEKAIMTYVSCFYHAFSGAQKAETAANRICKVLAVNQENEHLMEDYEKLASDLLEWIRRTIPWLEDRVPQKTIQEMQQKLEDFRDYRRVHKPPKVQEKCQLEINFNTLQTKLRLSNRPAFMPSEGKMVSDINNGWQHLEQAEKGYEEWLLNEIRRLERLDHLAEKFRQKASIHEAWTDGKEAMLKHRDYETATLSDIKALIRKHEAFESDLAAHQDRVEQIAAIAQELNELDYYDSHNVNTRCQKICDQWDALGSLTHSRREALEKTEKQLEAIDQLHLEYAKRAAPFNNWMESAMEDLQDMFIVHTIEEIEGLISAHDQFKSTLPDADREREAILAIHKEAQRIAESNHIKLSGSNPYTTVTPQIINSKWEKVQQLVPKRDHALLEEQSKQQSNEHLRRQFASQANVVGPWIQTKMEEIGRISIEMNGTLEDQLSHLKQYERSIVDYKPNLDLLEQQHQLIQEALIFDNKHTNYTMEHIRVGWEQLLTTIARTINEVENQILTRDAKGISQEQMQEFRASFNHFDKDHGGALGPEEFKACLISLGYDVENDRQGEAEFNRIMSLVDPNHSGLVTFQAFIDFMSRETTDTDTADQVIASFKVLAGDKNFITAEELRRELPPDQAEYCIARMAPYQGPDAVPGALDYKSFSTALYGESDL

28) >gi|21264343|ref|NP_002958.2| scaffold attachment factor B1 isoform 3 [Homo sapiens] (102.6 kD)

MAETLSGLGDSGAAGAAALSSASSETGTRRLSDLRVIDLRAELRKRNVDSSGNKSVLMERLKKAIEDEGGNPDEIEITSEGNKKTSKRSSKGRKPEEEGVEDNGLEENSGDGQEDVETSLENLQDIDIMDISVLDEAEIDNGSVADCVEDDDADNLQESLSDSRELVEGEMKELPEQLQEHAIEDKETINNLDTSSSDFTILQEIEEPSLEPENEKILDILGETCKSEPVKEESSELEQPFAQDTSSVGPDRKLAEEEDLFDSAHPEEGDLDLASESTAHAQSSKADSLLAVVKREPAEQPGDGERTDCEPVGLEPAVEQSSAASELAEASSEELAEAPTEAPSPEARDSKEDGRKFDFDACNEVPPAPKESSTSEGADQKMSSPEDDSDTKRLSKEEKGRSSCGRNFWVSGLSSTTRATDLKNLFSKYGKVVGAKVVTNARSPGARCYGFVTMSTAEEATKCINHLHKTELHGKMISVEKAKNEPVGKKTSDKRDSDGKKEKSSNSDRSTNLKRDDKCDRKDDAKKGDDGSGEKSKDQDDQKPGPSERSRATKSGSRGTERTVVMDKSKGVPVISVKTSGSKERASKSQDRKSASREKRSVVSFDKVKEPRKSRDSESHSRVRERSEREQRMQAQWEREERERLEIARERLAFQRQRLERERMERERLERERMHVEHERRREQERIHREREELRRQQELRYEQERRPAVRRPYDLDRRDDAYWPEAKRAALDERYHSDFNRQDRFHDFDHRDRGRYPDHSVDRREGSRSMMGEREGQHYPERHGGPERHGRDSRDGWGGYGSDKRMSEGRGLPPPPRRDWGDHGRREDDRSWQGTADGGMMDRDHKRWQGGERSMSGHSGPGHMMNRGGMSGRGSFAPGGASRGHPIPHGGMQGGFGGQSRGSRPSDARFTRRY

29) >gi|530380833|ref|XP_005265863.1| PREDICTED: G protein-regulated inducer of neurite outgrowth 1 isoform X1 [Homo sapiens] (102.3 kD)

MDTAEDPAWLQLLQKDSSPPGPRPTAFFCPQDGSLGAGSSAMRDYCPSQQKASPAPPRHTPDQSPGMESRHRSPSGAGEGASCSDGPRGSLACPSPTCFSPQESPSKETLEAHGASISGTPEATTSGKPEPVSSVKTEPKSSDDRNPMFLEKMDFKSSKQADSTSIGKEDPGSSRKADPMFTGKAEPEILGKGDPVAPGRMDPMTVRKEDLGSLGKVDPLCSSKTYTVSPRKEDPGSLRKVDPVSSDKVDPVFPRKEEPRYSGKEHPVSSEKVAPTSAEKVDLVLSGKRDPGPSGKADPMPLESMDSASTGKTEPGLLGKLIPGSSGKNGPVSSGTGAPGSLGRLDPTCLGMADPASVGNVETVPATKEDSRFLGKMDPASSGEGRPVSGHTDTTASAKTDLTSLKNVDPMSSGKVDPVSLGKMDPMCSGKPELLSPGQAERVSVGKAGTVSPGKEDPVSSRREDPISAGSRKTSSEKVNPESSGKTNPVSSGPGDPRSLGTAGPPSAVKAEPATGGKGDPLSSEKAGLVASGKAAPTASGKAEPLAVGKEDPVSKGKADAGPSGQGDSVSIGKVVSTPGKTVPVPSGKVDPVSLGKAEAIPEGKVGSLPLEKGSPVTTTKADPRASGKAQPQSGGKAETKLPGQEGAAAPGEAGAVCLKKETPQASEKVDPGSCRKAEPLASGKGEPVSLGKADSAPSRKTESPSLGKVVPLSLEKTKPSSSSRQLDRKALGSARSPEGARGSEGRVEPKAEPVSSTEASSLGQKDLEAAGAERSPCPEAAAPPPGPRTRDNFTKAPSWEASAPPPPREDAGTQAGAQACVSVAVSPMSPQDGAGGSAFSFQAAPRAPSPPSRRDAGLQVSLGAAETRSVATGPMTPQAAAPPAFPEVRVRPGSALAAAVAPPEPAEPVRDVSWDEKGMTWEVYGAAMEVEVLGMAIQKHLERQIEEHGRQGAPAPPPAARAGPGRSGSVRTAPPDGAAKRPPGLFRALLQSVRRPRCCSRAGPTAE

30) >gi|289577080|ref|NP_001409.3| eukaryotic translation initiation factor 4 gamma 2 isoform 1 [Homo sapiens] (102.3 kD)

MESAIAEGGASRFSASSGGGGSRGAPQHYPKTAGNSEFLGKTPGQNAQKWIPARSTRRDDNSAANNSANEKERHDAIFRKVRGILNKLTPEKFDKLCLELLNVGVESKLILKGVILLIVDKALEEPKYSSLYAQLCLRLAEDAPNFDGPAAEGQPGQKQSTTFRRLLISKLQDEFENRTRNVDVYDKRENPLLPEEEEQRAIAKIKMLGNIKFIGELGKLDLIHESILHKCIKTLLEKKKRVQLKDMGEDLECLCQIMRTVGPRLDHERAKSLMDQYFARMCSLMLSKELPARIRFLLQDTVELREHHWVPRKAFLDNGPKTINQIRQDAVKDLGVFIPAPMAQGMRSDFFLEGPFMPPRMKMDRDPLGGLADMFGQMPGSGIGTGPGVIQDRFSPTMGRHRSNQLFNGHGGHIMPPTQSQFGEMGGKFMKSQGLSQLYHNQSQGLLSQLQGQSKDMPPRFSKKGQLNADEISLRPAQSFLMNKNQVPKLQPQITMIPPSAQPPRTQTPPLGQTPQLGLKTNPPLIQEKPAKTSKKPPPSKEELLKLTETVVTEYLNSGNANEAVNGVREMRAPKHFLPEMLSKVIILSLDRSDEDKEKASSLISLLKQEGIATSDNFMQAFLNVLDQCPKLEVDIPLVKSYLAQFAARAIISELVSISELAQPLESGTHFPLFLLCLQQLAKLQDREWLTELFQQSKVNMQKMLPEIDQNKDRMLEILEGKGLSFLFPLLKLEKELLKQIKLDPSPQTIYKWIKDNISPKLHVDKGFVNILMTSFLQYISSEVNPPSDETDSSSAPSKEQLEQEKQLLLSFKPVMQKFLHDHVDLQVSALYALQVHCYNSNFPKGMLLRFFVHFYDMEIIEEEAFLAWKEDITQEFPGKGKALFQVNQWLTWLETAEEEESEEEAD

31) >gi|67089149|ref|NP_150241.2| protein PML isoform 1 [Homo sapiens] (97.5 kD)

MEPAPARSPRPQQDPARPQEPTMPPPETPSEGRQPSPSPSPTERAPASEEEFQFLRCQQCQAEAKCPKLLPCLHTLCSGCLEASGMQCPICQAPWPLGADTPALDNVFFESLQRRLSVYRQIVDAQAVCTRCKESADFWCFECEQLLCAKCFEAHQWFLKHEARPLAELRNQSVREFLDGTRKTNNIFCSNPNHRTPTLTSIYCRGCSKPLCCSCALLDSSHSELKCDISAEIQQRQEELDAMTQALQEQDSAFGAVHAQMHAAVGQLGRARAETEELIRERVRQVVAHVRAQERELLEAVDARYQRDYEEMASRLGRLDAVLQRIRTGSALVQRMKCYASDQEVLDMHGFLRQALCRLRQEEPQSLQAAVRTDGFDEFKVRLQDLSSCITQGKDAAVSKKASPEAASTPRDPIDVDLPEEAERVKAQVQALGLAEAQPMAVVQSVPGAHPVPVYAFSIKGPSYGEDVSNTTTAQKRKCSQTQCPRKVIKMESEEGKEARLARSSPEQPRPSTSKAVSPPHLDGPPSPRSPVIGSEVFLPNSNHVASGAGEAEERVVVISSSEDSDAENSSSRELDDSSSESSDLQLEGPSTLRVLDENLADPQAEDRPLVFFDLKIDNETQKISQLAAVNRESKFRVVIQPEAFFSIYSKAVSLEVGLQHFLSFLSSMRRPILACYKLWGPGLPNFFRALEDINRLWEFQEAISGFLAALPLIRERVPGASSFKLKNLAQTYLARNMSERSAMAAVLAMRDLCRLLEVSPGPQLAQHVYPFSSLQCFASLQPLVQAAVLPRAEARLLALHNVSFMELLSAHRRDRQGGLKKYSRYLSLQTTTLPPAQPAFNLQALGTYFEGLLEGPALARAEGVSTPLAGRGLAERASQQS

32) >gi|21914927|ref|NP_060533.2| lymphoid-specific helicase isoform 1 [Homo sapiens] (97 kD)

MPAERPAGSGGSEAPAMVEQLDTAVITPAMLEEEEQLEAAGLERERKMLEKARMSWDRESTEIRYRRLQHLLEKSNIYSKFLLTKMEQQQLEEQKKKEKLERKKESLKVKKGKNSIDASEEKPVMRKKRGREDESYNISEVMSKEEILSVAKKNKKENEDENSSSTNLCVEDLQKNKDSNSIIKDRLSETVRQNTKFFFDPVRKCNGQPVPFQQPKHFTGGVMRWYQVEGMEWLRMLWENGINGILADEMGLGKTVQCIATIALMIQRGVPGPFLVCGPLSTLPNWMAEFKRFTPDIPTMLYHGTQEERQKLVRNIYKRKGTLQIHPVVITSFEIAMRDRNALQHCYWKYLIVDEGHRIKNMKCRLIRELKRFNADNKLLLTGTPLQNNLSELWSLLNFLLPDVFDDLKSFESWFDITSLSETAEDIIAKEREQNVLHMLHQILTPFLLRRLKSDVALEVPPKREVVVYAPLSKKQEIFYTAIVNRTIANMFGSSEKETIELSPTGRPKRRTRKSINYSKIDDFPNELEKLISQIQPEVDRERAVVEVNIPVESEVNLKLQNIMMLLRKCCNHPYLIEYPIDPVTQEFKIDEELVTNSGKFLILDRMLPELKKRGHKVLLFSQMTSMLDILMDYCHLRDFNFSRLDGSMSYSEREKNMHSFNTDPEVFIFLVSTRAGGLGINLTAADTVIIYDSDWNPQSDLQAQDRCHRIGQTKPVVVYRLVTANTIDQKIVERAAAKRKLEKLIIHKNHFKGGQSGLNLSKNFLDPKELMELLKSRDYEREIKGSREKVISDKDLELLLDRSDLIDQMNASGPIKEKMGIFKILENSEDSSPECLF

33) >gi|4503483|ref|NP_001952.1| elongation factor 2 [Homo sapiens] (95.3 kD)

MVNFTVDQIRAIMDKKANIRNMSVIAHVDHGKSTLTDSLVCKAGIIASARAGETRFTDTRKDEQERCITIKSTAISLFYELSENDLNFIKQSKDGAGFLINLIDSPGHVDFSSEVTAALRVTDGALVVVDCVSGVCVQTETVLRQAIAERIKPVLMMNKMDRALLELQLEPEELYQTFQRIVENVNVIISTYGEGESGPMGNIMIDPVLGTVGFGSGLHGWAFTLKQFAEMYVAKFAAKGEGQLGPAERAKKVEDMMKKLWGDRYFDPANGKFSKSATSPEGKKLPRTFCQLILDPIFKVFDAIMNFKKEETAKLIEKLDIKLDSEDKDKEGKPLLKAVMRRWLPAGDALLQMITIHLPSPVTAQKYRCELLYEGPPDDEAAMGIKSCDPKGPLMMYISKMVPTSDKGRFYAFGRVFSGLVSTGLKVRIMGPNYTPGKKEDLYLKPIQRTILMMGRYVEPIEDVPCGNIVGLVGVDQFLVKTGTITTFEHAHNMRVMKFSVSPVVRVAVEAKNPADLPKLVEGLKRLAKSDPMVQCIIEESGEHIIAGAGELHLEICLKDLEEDHACIPIKKSDPVVSYRETVSEESNVLCLSKSPNKHNRLYMKARPFPDGLAEDIDKGEVSARQELKQRARYLAEKYEWDVAEARKIWCFGPDGTGPNILTDITKGVQYLNEIKDSVVAGFQWATKEGALCEENMRGVRFDVHDVTLHADAIHRGGGQIIPTARRCLYASVLTAQPRLMEPIYLVEIQCPEQVVGGIYGVLNRKRGHVFEESQVAGTPMFVVKAYLPVNESFGFTADLRSNTGGQAFPQCVFDHWQILPGDPFDNSSRPSQVVAETRKRKGLKEGIPALDNFLDKL

34) >gi|4507677|ref|NP_003290.1| endoplasmin precursor [Homo sapiens] (92.4 kD)

MRALWVLGLCCVLLTFGSVRADDEVDVDGTVEEDLGKSREGSRTDDEVVQREEEAIQLDGLNASQIRELREKSEKFAFQAEVNRMMKLIINSLYKNKEIFLRELISNASDALDKIRLISLTDENALSGNEELTVKIKCDKEKNLLHVTDTGVGMTREELVKNLGTIAKSGTSEFLNKMTEAQEDGQSTSELIGQFGVGFYSAFLVADKVIVTSKHNNDTQHIWESDSNEFSVIADPRGNTLGRGTTITLVLKEEASDYLELDTIKNLVKKYSQFINFPIYVWSSKTETVEEPMEEEEAAKEEKEESDDEAAVEEEEEEKKPKTKKVEKTVWDWELMNDIKPIWQRPSKEVEEDEYKAFYKSFSKESDDPMAYIHFTAEGEVTFKSILFVPTSAPRGLFDEYGSKKSDYIKLYVRRVFITDDFHDMMPKYLNFVKGVVDSDDLPLNVSRETLQQHKLLKVIRKKLVRKTLDMIKKIADDKYNDTFWKEFGTNIKLGVIEDHSNRTRLAKLLRFQSSHHPTDITSLDQYVERMKEKQDKIYFMAGSSRKEAESSPFVERLLKKGYEVIYLTEPVDEYCIQALPEFDGKRFQNVAKEGVKFDESEKTKESREAVEKEFEPLLNWMKDKALKDKIEKAVVSQRLTESPCALVASQYGWSGNMERIMKAQAYQTGKDISTNYYASQKKTFEINPRHPLIRDMLRRIKEDEDDKTVLDLAVVLFETATLRSGYLLPDTKAYGDRIERMLRLSLNIDPDAKVEEEPEEEPEETAEDTTEDTEQDEDEEMDVGTDEEEETAKESTAEKDEL

35) >gi|11067747|ref|NP_001244.1| cell division cycle 5-like protein [Homo sapiens] (92.2 kD)

MPRIMIKGGVWRNTEDEILKAAVMKYGKNQWSRIASLLHRKSAKQCKARWYEWLDPSIKKTEWSREEEEKLLHLAKLMPTQWRTIAPIIGRTAAQCLEHYEFLLDKAAQRDNEEETTDDPRKLKPGEIDPNPETKPARPDPIDMDEDELEMLSEARARLANTQGKKAKRKAREKQLEEARRLAALQKRRELRAAGIEIQKKRKRKRGVDYNAEIPFEKKPALGFYDTSEENYQALDADFRKLRQQDLDGELRSEKEGRDRKKDKQHLKRKKESDLPSAILQTSGVSEFTKKRSKLVLPAPQISDAELQEVVKVGQASEIARQTAEESGITNSASSTLLSEYNVTNNSVALRTPRTPASQDRILQEAQNLMALTNVDTPLKGGLNTPLHESDFSGVTPQRQVVQTPNTVLSTPFRTPSNGAEGLTPRSGTTPKPVINSTPGRTPLRDKLNINPEDGMADYSDPSYVKQMERESREHLRLGLLGLPAPKNDFEIVLPENAEKELEEREIDDTYIEDAADVDARKQAIRDAERVKEMKRMHKAVQKDLPRPSEVNETILRPLNVEPPLTDLQKSEELIKKEMITMLHYDLLHHPYEPSGNKKGKTVGFGTNNSEHITYLEHNPYEKFSKEELKKAQDVLVQEMEVVKQGMSHGELSSEAYNQVWEECYSQVLYLPGQSRYTRANLASKKDRIESLEKRLEINRGHMTTEAKRAAKMEKKMKILLGGYQSRAMGLMKQLNDLWDQIEQAHLELRTFEELKKHEDSAIPRRLECLKEDVQRQQEREKELQHRYADLLLEKETLKSKF

36) >gi|17978491|ref|NP_510966.1| CD97 antigen isoform 1 preproprotein [Homo sapiens] (91.8 kD)

MGGRVFLAFCVWLTLPGAETQDSRGCARWCPQNSSCVNATACRCNPGFSSFSEIITTPTETCDDINECATPSKVSCGKFSDCWNTEGSYDCVCSPGYEPVSGAKTFKNESENTCQDVDECQQNPRLCKSYGTCVNTLGSYTCQCLPGFKFIPEDPKVCTDVNECTSGQNPCHSSTHCLNNVGSYQCRCRPGWQPIPGSPNGPNNTVCEDVDECSSGQHQCDSSTVCFNTVGSYSCRCRPGWKPRHGIPNNQKDTVCEDMTFSTWTPPPGVHSQTLSRFFDKVQDLGRDSKTSSAEVTIQNVIKLVDELMEAPGDVEALAPPVRHLIATQLLSNLEDIMRILAKSLPKGPFTYISPSNTELTLMIQERGDKNVTMGQSSARMKLNWAVAAGAEDPGPAVAGILSIQNMTTLLANASLNLHSKKQAELEEIYESSIRGVQLRRLSAVNSIFLSHNNTKELNSPILFAFSHLESSDGEAGRDPPAKDVMPGPRQELLCAFWKSDSDRGGHWATEGCQVLGSKNGSTTCQCSHLSSFAILMAHYDVEDWKLTLITRVGLALSLFCLLLCILTFLLVRPIQGSRTTIHLHLCICLFVGSTIFLAGIENEGGQVGLRCRLVAGLLHYCFLAAFCWMSLEGLELYFLVVRVFQGQGLSTRWLCLIGYGVPLLIVGVSAAIYSKGYGRPRYCWLDFEQGFLWSFLGPVTFIILCNAVIFVTTVWKLTQKFSEINPDMKKLKKARALTITAIAQLFLLGCTWVFGLFIFDDRSLVLTYVFTILNCLQGAFLYLLHCLLNKKVREEYRKWACLVAGGSKYSEFTSTTSGTGHNQTRALRASESGI

37) >gi|74136883|ref|NP_114032.2| heterogeneous nuclear ribonucleoprotein U isoform a [Homo sapiens] (90.5 kD)

MSSSPVNVKKLKVSELKEELKKRRLSDKGLKAELMERLQAALDDEEAGGRPAMEPGNGSLDLGGDSAGRSGAGLEQEAAAGGDEEEEEEEEEEEGISALDGDQMELGEENGAAGAADSGPMEEEEAASEDENGDDQGFQEGEDELGDEEEGAGDENGHGEQQPQPPATQQQQPQQQRGAAKEAAGKSSGPTSLFAVTVAPPGARQGQQQAGGKKKAEGGGGGGRPGAPAAGDGKTEQKGGDKKRGVKRPREDHGRGYFEYIEENKYSRAKSPQPPVEEEDEHFDDTVVCLDTYNCDLHFKISRDRLSASSLTMESFAFLWAGGRASYGVSKGKVCFEMKVTEKIPVRHLYTKDIDIHEVRIGWSLTTSGMLLGEEEFSYGYSLKGIKTCNCETEDYGEKFDENDVITCFANFESDEVELSYAKNGQDLGVAFKISKEVLAGRPLFPHVLCHNCAVEFNFGQKEKPYFPIPEEYTFIQNVPLEDRVRGPKGPEEKKDCEVVMMIGLPGAGKTTWVTKHAAENPGKYNILGTNTIMDKMMVAGFKKQMADTGKLNTLLQRAPQCLGKFIEIAARKKRNFILDQTNVSAAAQRRKMCLFAGFQRKAVVVCPKDEDYKQRTQKKAEVEGKDLPEHAVLKMKGNFTLPEVAECFDEITYVELQKEEAQKLLEQYKEESKKALPPEKKQNTGSKKSNKNKSGKNQFNRGGGHRGRGGFNMRGGNFRGGAPGNRGGYNRRGNMPQRGGGGGGSGGIGYPYPRAPVFPGRGSYSNRGNYNRGGMPNRGNYNQNFRGRGNNRGYKNQSQGYNQWQQGQFWGQKPWSQHYHQGYY

38) >gi|5032087|ref|NP_005868.1| splicing factor 3A subunit 1 isoform 1 [Homo sapiens] (88.8 kD)

MPAGPVQAVPPPPPVPTEPKQPTEEEASSKEDSAPSKPVVGIIYPPPEVRNIVDKTASFVARNGPEFEARIRQNEINNPKFNFLNPNDPYHAYYRHKVSEFKEGKAQEPSAAIPKVMQQQQQTTQQQLPQKVQAQVIQETIVPKEPPPEFEFIADPPSISAFDLDVVKLTAQFVARNGRQFLTQLMQKEQRNYQFDFLRPQHSLFNYFTKLVEQYTKILIPPKGLFSKLKKEAENPREVLDQVCYRVEWAKFQERERKKEEEEKEKERVAYAQIDWHDFVVVETVDFQPNEQGNFPPPTTPEELGARILIQERYEKFGESEEVEMEVESDEEDDKQEKAEEPPSQLDQDTQVQDMDEGSDDEEEGQKVPPPPETPMPPPLPPTPDQVIVRKDYDPKASKPLPPAPAPDEYLVSPITGEKIPASKMQEHMRIGLLDPRWLEQRDRSIREKQSDDEVYAPGLDIESSLKQLAERRTDIFGVEETAIGKKIGEEEIQKPEEKVTWDGHSGSMARTQQAAQANITLQEQIEAIHKAKGLVPEDDTKEKIGPSKPNEIPQQPPPPSSATNIPSSAPPITSVPRPPTMPPPVRTTVVSAVPVMPRPPMASVVRLPPGSVIAPMPPIIHAPRINVVPMPPSAPPIMAPRPPPMIVPTAFVPAPPVAPVPAPAPMPPVHPPPPMEDEPTSKKLKTEDSLMPEEEFLRRNKGPVSIKVQVPNMQDKTEWKLNGQVLVFTLPLTDQVSVIKVKIHEATGMPAGKQKLQYEGIFIKDSNSLAYYNMANGAVIHLALKERGGRKK

39) >gi|5032179|ref|NP_005753.1| transcription intermediary factor 1-beta [Homo sapiens] (88.5 kD)

MAASAAAASAAAASAASGSPGPGEGSAGGEKRSTAPSAAASASASAAASSPAGGGAEALELLEHCGVCRERLRPEREPRLLPCLHSACSACLGPAAPAAANSSGDGGAAGDGTVVDCPVCKQQCFSKDIVENYFMRDSGSKAATDAQDANQCCTSCEDNAPATSYCVECSEPLCETCVEAHQRVKYTKDHTVRSTGPAKSRDGERTVYCNVHKHEPLVLFCESCDTLTCRDCQLNAHKDHQYQFLEDAVRNQRKLLASLVKRLGDKHATLQKSTKEVRSSIRQVSDVQKRVQVDVKMAILQIMKELNKRGRVLVNDAQKVTEGQQERLERQHWTMTKIQKHQEHILRFASWALESDNNTALLLSKKLIYFQLHRALKMIVDPVEPHGEMKFQWDLNAWTKSAEAFGKIVAERPGTNSTGPAPMAPPRAPGPLSKQGSGSSQPMEVQEGYGFGSGDDPYSSAEPHVSGVKRSRSGEGEVSGLMRKVPRVSLERLDLDLTADSQPPVFKVFPGSTTEDYNLIVIERGAAAAATGQPGTAPAGTPGAPPLAGMAIVKEEETEAAIGAPPTATEGPETKPVLMALAEGPGAEGPRLASPSGSTSSGLEVVAPEGTSAPGGGPGTLDDSATICRVCQKPGDLVMCNQCEFCFHLDCHLPALQDVPGEEWSCSLCHVLPDLKEEDGSLSLDGADSTGVVAKLSPANQRKCERVLLALFCHEPCRPLHQLATDSTFSLDQPGGTLDLTLIRARLQEKLSPPYSSPQEFAQDVGRMFKQFNKLTEDKADVQSIIGLQRFFETRMNEAFGDTKFSAVLVEPPPMSLPGAGLSSQELSGGPGDGP

40) >gi|19743813|ref|NP_002202.2| integrin beta-1 isoform 1A precursor [Homo sapiens] (88.4 kD)

MNLQPIFWIGLISSVCCVFAQTDENRCLKANAKSCGECIQAGPNCGWCTNSTFLQEGMPTSARCDDLEALKKKGCPPDDIENPRGSKDIKKNKNVTNRSKGTAEKLKPEDITQIQPQQLVLRLRSGEPQTFTLKFKRAEDYPIDLYYLMDLSYSMKDDLENVKSLGTDLMNEMRRITSDFRIGFGSFVEKTVMPYISTTPAKLRNPCTSEQNCTSPFSYKNVLSLTNKGEVFNELVGKQRISGNLDSPEGGFDAIMQVAVCGSLIGWRNVTRLLVFSTDAGFHFAGDGKLGGIVLPNDGQCHLENNMYTMSHYYDYPSIAHLVQKLSENNIQTIFAVTEEFQPVYKELKNLIPKSAVGTLSANSSNVIQLIIDAYNSLSSEVILENGKLSEGVTISYKSYCKNGVNGTGENGRKCSNISIGDEVQFEISITSNKCPKKDSDSFKIRPLGFTEEVEVILQYICECECQSEGIPESPKCHEGNGTFECGACRCNEGRVGRHCECSTDEVNSEDMDAYCRKENSSEICSNNGECVCGQCVCRKRDNTNEIYSGKFCECDNFNCDRSNGLICGGNGVCKCRVCECNPNYTGSACDCSLDTSTCEASNGQICNGRGICECGVCKCTDPKFQGQTCEMCQTCLGVCAEHKECVQCRAFNKGEKKDTCTQECSYFNITKVESRDKLPQPVQPDPVSHCKEKDVDDCWFYFTYSVNGNNEVMVHVVENPECPTGPDIIPIVAGVVAGIVLIGLALLLIWKLLMIIHDRREFAKFEKEKMNAKWDTGENPIYKSAVTTVVNPKYEGK

**Figure legend**: These are 40 largest proteins identified from the 55-kD stripe (MB231). The red underlined sequences are the LC-MS/MS identified peptide fragments that are unique to the protein, while the green underlined sequences are the LC-MS/MS identified peptide fragments that are not unique to the protein but can also appear in other proteins.

**The 20 smallest proteins identified in the 72-kD stripe (MB231)**

1) >gi|4504301|ref|NP_003529.1| histone H4 [Homo sapiens] (11.4 kD)

MSGRGKGGKGLGKGGAKRHRKVLRDNIQGITKPAIRRLARRGGVKRISGLIYEETRGVLKVFLENVIRDAVTYTEHAKRKTVTAMDVVYALKRQGRTLYGFGG

2) >gi|11128019|ref|NP_061820.1| cytochrome c [Homo sapiens] (11.7 kD)

MGDVEKGKKIFIMKCSQCHTVEKGGKHKTGPNLHGLFGRKTGQAPGYSYTAANKNKGIIWGEDTLMEYLENPKKYIPGTKMIFVGIKKKEERADLIAYLKKATNE

3) >gi|4504981|ref|NP_002296.1| galectin-1 [Homo sapiens] (14.7 kD)

MACGLVASNLNLKPGECLRVRGEVAPDAKSFVLNLGKDSNNLCLHFNPRFNAHGDANTIVCNSKDGGAWGTEQREAVFPFQPGSVAEVCITFDQANLTVKLPDGYEFKFPNRLNLEAINYMAADGDFKIKCVAFD

4) >gi|71772415|ref|NP_001025180.1| 40S ribosomal protein S15a [Homo sapiens] (14.8 kD)

MVRMNVLADALKSINNAEKRGKRQVLIRPCSKVIVRFLTVMMKHGYIGEFEIIDDHRAGKIVVNLTGRLNKCGVISPRFDVQLKDLEKWQNNLLPSRQFGFIVLTTSAGIMDHEEARRKHTGGKILGFFF

5) >gi|4826898|ref|NP_005013.1| profilin-1 [Homo sapiens] (15 kD)

MAGWNAYIDNLMADGTCQDAAIVGYKDSPSVWAAVPGKTFVNITPAEVGVLVGKDRSSFYVNGLTLGGQKCSVIRDSLLQDGEFSMDLRTKSTGGAPTFNVTVTKTDKTLVLLMGKEGVHGGLINKKCYEMASHLRRSQY

6) >gi|7705636|ref|NP_057156.1| vesicle transport protein GOT1B [Homo sapiens] (15.4 kD)

MISLTDTQKIGMGLTGFGVFFLFFGMILFFDKALLAIGNVLFVAGLAFVIGLERTFRFFFQKHKMKATGFFLGGVFVVLIGWPLIGMIFEIYGFFLLFRGFFPVVVGFIRRVPVLGSLLNLPGIRSFVDKVGESNNMV

7) >gi|4506701|ref|NP_001016.1| 40S ribosomal protein S23 [Homo sapiens] (15.8 kD)

MGKCRGLRTARKLRSHRRDQKWHDKQYKKAHLGTALKANPFGGASHAKGIVLEKVGVEAKQPNSAIRKCVRVQLIKNGKKITAFVPNDGCLNFIEENDEVLVAGFGRKGHAVGDIPGVRFKVVKVANVSLLALYKGKKERPRS

8) >gi|4502297|ref|NP_001678.1| ATP synthase subunit delta, mitochondrial precursor [Homo sapiens] (17.5 kD)

MLPAALLRRPGLGRLVRHARAYAEAAAAPAAASGPNQMSFTFASPTQVFFNGANVRQVDVPTLTGAFGILAAHVPTLQVLRPGLVVVHAEDGTTSKYFVSSGSIAVNADSSVQLLAEEAVTLDMLDLGAAKANLEKAQAELVGTADEATRAEIQIRIEANEALVKALE

9) >gi|4826774|ref|NP_005092.1| ubiquitin-like protein ISG15 precursor [Homo sapiens] (17.9 kD)

MGWDLTVKMLAGNEFQVSLSSSMSVSELKAQITQKIGVHAFQQRLAVHPSGVALQDRVPLASQGLGPGSTVLLVVDKCDEPLSILVRNNKGRSSTYEVRLTQTVAHLKQQVSGLEGVQDDLFWLTFEGKPLEDQLPLGEYGLKPLSTVFMNLRLRGGGTEPGGRS

10) >gi|5031635|ref|NP_005498.1| cofilin-1 [Homo sapiens] (18.5 kD)

MASGVAVSDGVIKVFNDMKVRKSSTPEEVKKRKKAVLFCLSEDKKNIILEEGKEILVGDVGQTVDDPYATFVKMLPDKDCRYALYDATYETKESKKEDLVFIFWAPESAPLKSKMIYASSKDAIKKKLTGIKHELQANCYEEVKDRCTLAEKLGGSAVISLEGKPL

11) >gi|4506679|ref|NP_001005.1| 40S ribosomal protein S10 [Homo sapiens] (18.9 kD)

MLMPKKNRIAIYELLFKEGVMVAKKDVHMPKHPELADKNVPNLHVMKAMQSLKSRGYVKEQFAWRHFYWYLTNEGIQYLRDYLHLPPEIVPATLRRSRPETGRPRPKGLEGERPARLTRGEADRDTYRRSAVPPGADKKAEAGAGSATEFQFRGGFGRGRGQPPQ

12) >gi|4502205|ref|NP_001651.1| ADP-ribosylation factor 4 [Homo sapiens] (20.5 kD)

MGLTISSLFSRLFGKKQMRILMVGLDAAGKTTILYKLKLGEIVTTIPTIGFNVETVEYKNICFTVWDVGGQDRIRPLWKHYFQNTQGLIFVVDSNDRERIQEVADELQKMLLVDELRDAVLLLFANKQDLPNAMAISEMTDKLGLQSLRNRTWYVQATCATQGTGLYEGLDWLSNELSKR

13) >gi|7657609|ref|NP_055115.1| signal peptidase complex catalytic subunit SEC11A isoform 2 [Homo sapiens] (20.6 kD)

MLSLDFLDDVRRMNKRQLYYQVLNFGMIVSSALMIWKGLMVITGSESPIVVVLSGSMEPAFHRGDLLFLTNRVEDPIRVGEIVVFRIEGREIPIVHRVLKIHEKQNGHIKFLTKGDNNAVDDRGLYKQGQHWLEKKDVVGRARGFVPYIGIVTILMNDYPKFKYAVLFLLGLFVLVHRE

14) >gi|7661678|ref|NP_056461.1| ras-related protein Rap-1b isoform 1 precursor [Homo sapiens] (20.8 kD)

MREYKLVVLGSGGVGKSALTVQFVQGIFVEKYDPTIEDSYRKQVEVDAQQCMLEILDTAGTEQFTAMRDLYMKNGQGFALVYSITAQSTFNDLQDLREQILRVKDTDDVPMILVGNKCDLEDERVVGKEQGQNLARQWNNCAFLESSAKSKINVNEIFYDLVRQINRKTPVPGKARKKSSCQLL

15) >gi|4885375|ref|NP_005310.1| histone H1.2 [Homo sapiens] (21.4 kD)

MSETAPAAPAAAPPAEKAPVKKKAAKKAGGTPRKASGPPVSELITKAVAASKERSGVSLAALKKALAAAGYDVEKNNSRIKLGLKSLVSKGTLVQTKGTGASGSFKLNKKAASGEAKPKVKKAGGTKPKKPVGAAKKPKKAAGGATPKKSAKKTPKKAKKPAAATVTKKVAKSPKKAKVAKPKKAAKSAAKAVKPKAAKPKVVKPKKAAPKKK

16) >gi|4506381|ref|NP_002863.1| ras-related C3 botulinum toxin substrate 2 [Homo sapiens] (21.4 kD)

MQAIKCVVVGDGAVGKTCLLISYTTNAFPGEYIPTVFDNYSANVMVDSKPVNLGLWDTAGQEDYDRLRPLSYPQTDVFLICFSLVSPASYENVRAKWFPEVRHHCPSTPIILVGTKLDLRDDKDTIEKLKEKKLAPITYPQGLALAKEIDSVKYLECSALTQRGLKTVFDEAIRAVLCPQPTRQQKRACSLL

17) >gi|320461711|ref|NP_001189360.1| peroxiredoxin-1 [Homo sapiens] (22.1 kD)

MSSGNAKIGHPAPNFKATAVMPDGQFKDISLSDYKGKYVVFFFYPLDFTFVCPTEIIAFSDRAEEFKKLNCQVIGASVDSHFCHLAWVNTPKKQGGLGPMNIPLVSDPKRTIAQDYGVLKADEGISFRGLFIIDDKGILRQITVNDLPVGRSVDETLRLVQAFQFTDKHGEVCPAGWKPGSDTIKPDVQKSKEYFSKQK

18) >gi|256222019|ref|NP_057215.3| ras-related protein Rab-10 [Homo sapiens] (22.5 kD)

MAKKTYDLLFKLLLIGDSGVGKTCVLFRFSDDAFNTTFISTIGIDFKIKTVELQGKKIKLQIWDTAGQERFHTITTSYYRGAMGIMLVYDITNGKSFENISKWLRNIDEHANEDVERMLLGNKCDMDDKRVVPKGKGEQIAREHGIRFFETSAKANINIEKAFLTLAEDILRKTPVKEPNSENVDISSGGGVTGWKSKCC

19) >gi|530433542|ref|XP_005278344.1| PREDICTED: 40S ribosomal protein S9 isoform X16 [Homo sapiens] (22.6 kD)

MPVARSWVCRKTYVTPRRPFEKSRLDQELKLIGEYGLRNKREVWRVKFTLAKIRKAARELLTLDEKDPRRLFEGNALLRRLVRIGVLDEGKMKLDYILGLKIEDFLERRLQTQVFKLGLAKSIHHARVLIRQRHIRVRKQVVNIPSFIVRLDSQKHIDFSLRSPYGGGRPGRVKRKNAKKGQGGAGAGDDEEED

20) >gi|409971425|ref|NP_001258535.1| brain acid soluble protein 1 [Homo sapiens] (22.7 kD)

MGGKLSKKKKGYNVNDEKAKEKDKKAEGAATEEEGTPKESEPQAAAEPAEAKEGKEKPDQDAEGKAEEKEGEKDAAAAKEEAPKAEPEKTEGAAEAKAEPPKAPEQEQAAPGPAAGGEAPKAAEAAAAPAESAAPAAGEEPSKEEGEPKKTEAPAAPAAQETKSDGAPASDSKPGSSEAAPSSKETPAATEAPSSTPKAQGPAASAEEPKPVEAPAANSDQTVTVKE

**Figure legend**: These are 20 smallest proteins identified in the 72-kD stripe (MB231). The red underlined sequences are the LC-MS/MS identified peptide fragments that are unique to the protein, while the green underlined sequences are the LC-MS/MS identified peptide fragments that are not unique to the protein but can also appear in other proteins.

**The 40 largest proteins in the 72-kD stripe (MB231)**

1) >gi|61743954|ref|NP_001611.1| neuroblast differentiation-associated protein AHNAK isoform 1 [Homo sapiens] (628.7 kD)

MEKEETTRELLLPNWQGSGSHGLTIAQRDDGVFVQEVTQNSPAARTGVVKEGDQIVGATIYFDNLQSGEVTQLLNTMGHHTVGLKLHRKGDRSPEPGQTWTREVFSSCSSEVVLSGDDEEYQRIYTTKIKPRLKSEDGVEGDLGETQSRTITVTRRVTAYTVDVTGREGAKDIDISSPEFKIKIPRHELTEISNVDVETQSGKTVIRLPSGSGAASPTGSAVDIRAGAISASGPELQGAGHSKLQVTMPGIKVGGSGVNVNAKGLDLGGRGGVQVPAVDISSSLGGRAVEVQGPSLESGDHGKIKFPTMKVPKFGVSTGREGQTPKAGLRVSAPEVSVGHKGGKPGLTIQAPQLEVSVPSANIEGLEGKLKGPQITGPSLEGDLGLKGAKPQGHIGVDASAPQIGGSITGPSVEVQAPDIDVQGPGSKLNVPKMKVPKFSVSGAKGEETGIDVTLPTGEVTVPGVSGDVSLPEIATGGLEGKMKGTKVKTPEMIIQKPKISMQDVDLSLGSPKLKGDIKVSAPGVQGDVKGPQVALKGSRVDIETPNLEGTLTGPRLGSPSGKTGTCRISMSEVDLNVAAPKVKGGVDVTLPRVEGKVKVPEVDVRGPKVDVSAPDVEAHGPEWNLKMPKMKMPTFSTPGAKGEGPDVHMTLPKGDISISGPKVNVEAPDVNLEGLGGKLKGPDVKLPDMSVKTPKISMPDVDLHVKGTKVKGEYDVTVPKLEGELKGPKVDIDAPDVDVHGPDWHLKMPKMKMPKFSVPGFKAEGPEVDVNLPKADVDISGPKIDVTAPDVSIEEPEGKLKGPKFKMPEMNIKVPKISMPDVDLHLKGPNVKGEYDVTMPKVESEIKVPDVELKSAKMDIDVPDVEVQGPDWHLKMPKMKMPKFSMPGFKAEGPEVDVNLPKADVDISGPKVGVEVPDVNIEGPEGKLKGPKFKMPEMNIKAPKISMPDVDLHMKGPKVKGEYDMTVPKLEGDLKGPKVDVSAPDVEMQGPDWNLKMPKIKMPKFSMPSLKGEGPEFDVNLSKANVDISAPKVDTNAPDLSLEGPEGKLKGPKFKMPEMHFRAPKMSLPDVDLDLKGPKMKGNVDISAPKIEGEMQVPDVDIRGPKVDIKAPDVEGQGLDWSLKIPKMKMPKFSMPSLKGEGPEVDVNLPKADVVVSGPKVDIEAPDVSLEGPEGKLKGPKFKMPEMHFKTPKISMPDVDLHLKGPKVKGDVDVSVPKVEGEMKVPDVEIKGPKMDIDAPDVEVQGPDWHLKMPKMKMPKFSMPGFKGEGREVDVNLPKADIDVSGPKVDVEVPDVSLEGPEGKLKGPKFKMPEMHFKAPKISMPDVDLNLKGPKLKGDVDVSLPEVEGEMKVPDVDIKGPKVDISAPDVDVHGPDWHLKMPKVKMPKFSMPGFKGEGPEVDVKLPKADVDVSGPKMDAEVPDVNIEGPDAKLKGPKFKMPEMSIKPQKISIPDVGLHLKGPKMKGDYDVTVPKVEGEIKAPDVDIKGPKVDINAPDVEVHGPDWHLKMPKVKMPKFSMPGFKGEGPEVDMNLPKADLGVSGPKVDIDVPDVNLEAPEGKLKGPKFKMPSMNIQTHKISMPDVGLNLKAPKLKTDVDVSLPKVEGDLKGPEIDVKAPKMDVNVGDIDIEGPEGKLKGPKFKMPEMHFKAPKISMPDVDLHLKGPKVKGDMDVSVPKVEGEMKVPDVDIKGPKVDIDAPDVEVHDPDWHLKMPKMKMPKFSMPGFKAEGPEVDVNLPKADIDVSGPSVDTDAPDLDIEGPEGKLKGSKFKMPKLNIKAPKVSMPDVDLNLKGPKLKGEIDASVPELEGDLRGPQVDVKGPFVEAEVPDVDLECPDAKLKGPKFKMPEMHFKAPKISMPDVDLHLKGPKVKGDADVSVPKLEGDLTGPSVGVEVPDVELECPDAKLKGPKFKMPDMHFKAPKISMPDVDLHLKGPKVKGDVDVSVPKLEGDLTGPSVGVEVPDVELECPDAKLKGPKFKMPEMHFKTPKISMPDVDLHLKGPKVKGDMDVSVPKVEGEMKVPDVDIKGPKMDIDAPDVDVHGPDWHLKMPKMKMPKFSMPGFKAEGPEVDVNLPKADVVVSGPKVDVEVPDVSLEGPEGKLKGPKLKMPEMHFKAPKISMPDVDLHLKGPKVKGDVDVSLPKLEGDLTGPSVDVEVPDVELECPDAKLKGPKFKMPEMHFKTPKISMPDVNLNLKGPKVKGDMDVSVPKVEGEMKVPDVDIRGPKVDIDAPDVDVHGPDWHLKMPKMKMPKFSMPGFKGEGPEVDVNLPKADVDVSGPKVDVEVPDVSLEGPEGKLKGPKFKMPEMHFKTPKISMPDVDFNLKGPKIKGDVDVSAPKLEGELKGPELDVKGPKLDADMPEVAVEGPNGKWKTPKFKMPDMHFKAPKISMPDLDLHLKSPKAKGEVDVDVPKLEGDLKGPHVDVSGPDIDIEGPEGKLKGPKFKMPDMHFKAPNISMPDVDLNLKGPKIKGDVDVSVPEVEGKLEVPDMNIRGPKVDVNAPDVQAPDWHLKMPKMKMPKFSMPGFKAEGPEVDVNLPKADVDISGPKVDIEGPDVNIEGPEGKLKGPKLKMPEMNIKAPKISMPDFDLHLKGPKVKGDVDVSLPKVEGDLKGPEVDIKGPKVDINAPDVGVQGPDWHLKMPKVKMPKFSMPGFKGEGPDGDVKLPKADIDVSGPKVDIEGPDVNIEGPEGKLKGPKFKMPEMNIKAPKISMPDIDLNLKGPKVKGDVDVSLPKVEGDLKGPEVDIKGPKVDIDAPDVDVHGPDWHLKMPKIKMPKISMPGFKGEGPDVDVNLPKADIDVSGPKVDVECPDVNIEGPEGKWKSPKFKMPEMHFKTPKISMPDIDLNLTGPKIKGDVDVTGPKVEGDLKGPEVDLKGPKVDIDVPDVNVQGPDWHLKMPKMKMPKFSMPGFKAEGPEVDVNLPKADVDVSGPKVDVEGPDVNIEGPEGKLKGPKFKMPEMNIKAPKIPMPDFDLHLKGPKVKGDVDISLPKVEGDLKGPEVDIRGPQVDIDVPDVGVQGPDWHLKMPKVKMPKFSMPGFKGEGPDVDVNLPKADLDVSGPKVDIDVPDVNIEGPEGKLKGPKFKMPEMNIKAPKISMPDIDLNLKGPKVKGDMDVSLPKVEGDMKVPDVDIKGPKVDINAPDVDVQGPDWHLKMPKIKMPKISMPGFKGEGPEVDVNLPKADLDVSGPKVDVDVPDVNIEGPDAKLKGPKFKMPEMNIKAPKISMPDLDLNLKGPKMKGEVDVSLANVEGDLKGPALDIKGPKIDVDAPDIDIHGPDAKLKGPKLKMPDMHVNMPKISMPEIDLNLKGSKLKGDVDVSGPKLEGDIKAPSLDIKGPEVDVSGPKLNIEGKSKKSRFKLPKFNFSGSKVQTPEVDVKGKKPDIDITGPKVDINAPDVEVQGKVKGSKFKMPFLSISSPKVSMPDVELNLKSPKVKGDLDIAGPNLEGDFKGPKVDIKAPEVNLNAPDVDVHGPDWNLKMPKMKMPKFSVSGLKAEGPDVAVDLPKGDINIEGPSMNIEGPDLNVEGPEGGLKGPKFKMPDMNIKAPKISMPDIDLNLKGPKVKGDVDISLPKLEGDLKGPEVDIKGPKVDINAPDVDVHGPDWHLKMPKVKMPKFSMPGFKGEGPEVDVTLPKADIDISGPNVDVDVPDVNIEGPDAKLKGPKFKMPEMNIKAPKISMPDFDLNLKGPKMKGDVVVSLPKVEGDLKGPEVDIKGPKVDIDTPDINIEGSEGKFKGPKFKIPEMHLKAPKISMPDIDLNLKGPKVKGDVDVSLPKMEGDLKGPEVDIKGPKVDINAPDVDVQGPDWHLKMPKVKMPKFSMPGFKGEGPDVDVNLPKADLDVSGPKVDIDVPDVNIEGPEGKLKGPKFKMPEMNIKAPKISMPDIDLNLKGPKVKGDMDVSLPKVEGDMQVPDLDIKGPKVDINAPDVDVRGPDWHLKMPKIKMPKISMPGFKGEGPEVDVNLPKADLDVSGPKVDVDVPDVNIEGPDAKLKGPKFKMPEMNIKAPKISMPDFDLHLKGPKVKGDVDVSLPKMEGDLKAPEVDIKGPKVDIDAPDVDVHGPDWHLKMPKVKMPKFSMPGFKGEGPEVDVNLPKADIDVSGPKVDIDTPDIDIHGPEGKLKGPKFKMPDLHLKAPKISMPEVDLNLKGPKMKGDVDVSLPKVEGDLKGPEVDIKGPKVDIDVPDVDVQGPDWHLKMPKVKMPKFSMPGFKGEGPDVDVNLPKADLDVSGPKVDIDVPDVNIEGPDAKLKGPKFKMPEMNIKAPKISMPDFDLHLKGPKVKGDVDVSLPKVEGDLKGPEVDIKGPKVDIDAPDVDVHGPDWHLKMPKVKMPKFSMPGFKGEGPDVDVTLPKADIEISGPKVDIDAPDVSIEGPDAKLKGPKFKMPEMNIKAPKISMPDIDFNLKGPKVKGDVDVSLPKVEGDLKGPEIDIKGPSLDIDTPDVNIEGPEGKLKGPKFKMPEMNIKAPKISMPDFDLHLKGPKVKGDVDVSLPKVESDLKGPEVDIEGPEGKLKGPKFKMPDVHFKSPQISMSDIDLNLKGPKIKGDMDISVPKLEGDLKGPKVDVKGPKVGIDTPDIDIHGPEGKLKGPKFKMPDLHLKAPKISMPEVDLNLKGPKVKGDMDISLPKVEGDLKGPEVDIRDPKVDIDVPDVDVQGPDWHLKMPKVKMPKFSMPGFKGEGPDVDVNLPKADIDVSGPKVDVDVPDVNIEGPDAKLKGPKFKMPEMSIKAPKISMPDIDLNLKGPKVKGDVDVTLPKVEGDLKGPEADIKGPKVDINTPDVDVHGPDWHLKMPKVKMPKFSMPGFKGEGPDVDVSLPKADIDVSGPKVDVDIPDVNIEGPDAKLKGPKFKMPEINIKAPKISIPDVDLDLKGPKVKGDFDVSVPKVEGTLKGPEVDLKGPRLDFEGPDAKLSGPSLKMPSLEISAPKVTAPDVDLHLKAPKIGFSGPKLEGGEVDLKGPKVEAPSLDVHMDSPDINIEGPDVKIPKFKKPKFGFGAKSPKADIKSPSLDVTVPEAELNLETPEISVGGKGKKSKFKMPKIHMSGPKIKAKKQGFDLNVPGGEIDASLKAPDVDVNIAGPDAALKVDVKSPKTKKTMFGKMYFPDVEFDIKSPKFKAEAPLPSPKLEGELQAPDLELSLPAIHVEGLDIKAKAPKVKMPDVDISVPKIEGDLKGPKVQANLGAPDINIEGLDAKVKTPSFGISAPQVSIPDVNVNLKGPKIKGDVPSVGLEGPDVDLQGPEAKIKFPKFSMPKIGIPGVKMEGGGAEVHAQLPSLEGDLRGPDVKLEGPDVSLKGPGVDLPSVNLSMPKVSGPDLDLNLKGPSLKGDLDASVPSMKVHAPGLNLSGVGGKMQVGGDGVKVPGIDATTKLNVGAPDVTLRGPSLQGDLAVSGDIKCPKVSVGAPDLSLEASEGSIKLPKMKLPQFGISTPGSDLHVNAKGPQVSGELKGPGVDVNLKGPRISAPNVDFNLEGPKVKGSLGATGEIKGPTVGGGLPGIGVQGLEGNLQMPGIKSSGCDVNLPGVNVKLPTGQISGPEIKGGLKGSEVGFHGAAPDISVKGPAFNMASPESDFGINLKGPKIKGGADVSGGVSAPDISLGEGHLSVKGSGGEWKGPQVSSALNLDTSKFAGGLHFSGPKVEGGVKGGQIGLQAPGLSVSGPQGHLESGSGKVTFPKMKIPKFTFSGRELVGREMGVDVHFPKAEASIQAGAGDGEWEESEVKLKKSKIKMPKFNFSKPKGKGGVTGSPEASISGSKGDLKSSKASLGSLEGEAEAEASSPKGKFSLFKSKKPRHRSNSFSDEREFSGPSTPTGTLEFEGGEVSLEGGKVKGKHGKLKFGTFGGLGSKSKGHYEVTGSDDETGKLQGSGVSLASKKSRLSSSSSNDSGNKVGIQLPEVELSVSTKKE

2) >gi|41322916|ref|NP_958782.1| plectin isoform 1 [Homo sapiens] (531.5 kD)

MVAGMLMPRDQLRAIYEVLFREGVMVAKKDRRPRSLHPHVPGVTNLQVMRAMASLRARGLVRETFAWCHFYWYLTNEGIAHLRQYLHLPPEIVPASLQRVRRPVAMVMPARRTPHVQAVQGPLGSPPKRGPLPTEEQRVYRRKELEEVSPETPVVPATTQRTLARPGPEPAPATDERDRVQKKTFTKWVNKHLIKAQRHISDLYEDLRDGHNLISLLEVLSGDSLPREKGRMRFHKLQNVQIALDYLRHRQVKLVNIRNDDIADGNPKLTLGLIWTIILHFQISDIQVSGQSEDMTAKEKLLLWSQRMVEGYQGLRCDNFTSSWRDGRLFNAIIHRHKPLLIDMNKVYRQTNLENLDQAFSVAERDLGVTRLLDPEDVDVPQPDEKSIITYVSSLYDAMPRVPDVQDGVRANELQLRWQEYRELVLLLLQWMRHHTAAFEERRFPSSFEEIEILWSQFLKFKEMELPAKEADKNRSKGIYQSLEGAVQAGQLKVPPGYHPLDVEKEWGKLHVAILEREKQLRSEFERLECLQRIVTKLQMEAGLCEEQLNQADALLQSDVRLLAAGKVPQRAGEVERDLDKADSMIRLLFNDVQTLKDGRHPQGEQMYRRVYRLHERLVAIRTEYNLRLKAGVAAPATQVAQVTLQSVQRRPELEDSTLRYLQDLLAWVEENQHRVDGAEWGVDLPSVEAQLGSHRGLHQSIEEFRAKIERARSDEGQLSPATRGAYRDCLGRLDLQYAKLLNSSKARLRSLESLHSFVAAATKELMWLNEKEEEEVGFDWSDRNTNMTAKKESYSALMRELELKEKKIKELQNAGDRLLREDHPARPTVESFQAALQTQWSWMLQLCCCIEAHLKENAAYFQFFSDVREAEGQLQKLQEALRRKYSCDRSATVTRLEDLLQDAQDEKEQLNEYKGHLSGLAKRAKAVVQLKPRHPAHPMRGRLPLLAVCDYKQVEVTVHKGDECQLVGPAQPSHWKVLSSSGSEAAVPSVCFLVPPPNQEAQEAVTRLEAQHQALVTLWHQLHVDMKSLLAWQSLRRDVQLIRSWSLATFRTLKPEEQRQALHSLELHYQAFLRDSQDAGGFGPEDRLMAEREYGSCSHHYQQLLQSLEQGAQEESRCQRCISELKDIRLQLEACETRTVHRLRLPLDKEPARECAQRIAEQQKAQAEVEGLGKGVARLSAEAEKVLALPEPSPAAPTLRSELELTLGKLEQVRSLSAIYLEKLKTISLVIRGTQGAEEVLRAHEEQLKEAQAVPATLPELEATKASLKKLRAQAEAQQPTFDALRDELRGAQEVGERLQQRHGERDVEVERWRERVAQLLERWQAVLAQTDVRQRELEQLGRQLRYYRESADPLGAWLQDARRRQEQIQAMPLADSQAVREQLRQEQALLEEIERHGEKVEECQRFAKQYINAIKDYELQLVTYKAQLEPVASPAKKPKVQSGSESVIQEYVDLRTHYSELTTLTSQYIKFISETLRRMEEEERLAEQQRAEERERLAEVEAALEKQRQLAEAHAQAKAQAEREAKELQQRMQEEVVRREEAAVDAQQQKRSIQEELQQLRQSSEAEIQAKARQAEAAERSRLRIEEEIRVVRLQLEATERQRGGAEGELQALRARAEEAEAQKRQAQEEAERLRRQVQDESQRKRQAEVELASRVKAEAEAAREKQRALQALEELRLQAEEAERRLRQAEVERARQVQVALETAQRSAEAELQSKRASFAEKTAQLERSLQEEHVAVAQLREEAERRAQQQAEAERAREEAERELERWQLKANEALRLRLQAEEVAQQKSLAQAEAEKQKEEAEREARRRGKAEEQAVRQRELAEQELEKQRQLAEGTAQQRLAAEQELIRLRAETEQGEQQRQLLEEELARLQREAAAATQKRQELEAELAKVRAEMEVLLASKARAEEESRSTSEKSKQRLEAEAGRFRELAEEAARLRALAEEAKRQRQLAEEDAARQRAEAERVLAEKLAAIGEATRLKTEAEIALKEKEAENERLRRLAEDEAFQRRRLEEQAAQHKADIEERLAQLRKASDSELERQKGLVEDTLRQRRQVEEEILALKASFEKAAAGKAELELELGRIRSNAEDTLRSKEQAELEAARQRQLAAEEERRRREAEERVQKSLAAEEEAARQRKAALEEVERLKAKVEEARRLRERAEQESARQLQLAQEAAQKRLQAEEKAHAFAVQQKEQELQQTLQQEQSVLDQLRGEAEAARRAAEEAEEARVQAEREAAQSRRQVEEAERLKQSAEEQAQARAQAQAAAEKLRKEAEQEAARRAQAEQAALRQKQAADAEMEKHKKFAEQTLRQKAQVEQELTTLRLQLEETDHQKNLLDEELQRLKAEATEAARQRSQVEEELFSVRVQMEELSKLKARIEAENRALILRDKDNTQRFLQEEAEKMKQVAEEAARLSVAAQEAARLRQLAEEDLAQQRALAEKMLKEKMQAVQEATRLKAEAELLQQQKELAQEQARRLQEDKEQMAQQLAEETQGFQRTLEAERQRQLEMSAEAERLKLRVAEMSRAQARAEEDAQRFRKQAEEIGEKLHRTELATQEKVTLVQTLEIQRQQSDHDAERLREAIAELEREKEKLQQEAKLLQLKSEEMQTVQQEQLLQETQALQQSFLSEKDSLLQRERFIEQEKAKLEQLFQDEVAKAQQLREEQQRQQQQMEQERQRLVASMEEARRRQHEAEEGVRRKQEELQQLEQQRRQQEELLAEENQRLREQLQLLEEQHRAALAHSEEVTASQVAATKTLPNGRDALDGPAAEAEPEHSFDGLRRKVSAQRLQEAGILSAEELQRLAQGHTTVDELARREDVRHYLQGRSSIAGLLLKATNEKLSVYAALQRQLLSPGTALILLEAQAASGFLLDPVRNRRLTVNEAVKEGVVGPELHHKLLSAERAVTGYKDPYTGQQISLFQAMQKGLIVREHGIRLLEAQIATGGVIDPVHSHRVPVDVAYRRGYFDEEMNRVLADPSDDTKGFFDPNTHENLTYLQLLERCVEDPETGLCLLPLTDKAAKGGELVYTDSEARDVFEKATVSAPFGKFQGKTVTIWEIINSEYFTAEQRRDLLRQFRTGRITVEKIIKIIITVVEEQEQKGRLCFEGLRSLVPAAELLESRVIDRELYQQLQRGERSVRDVAEVDTVRRALRGANVIAGVWLEEAGQKLSIYNALKKDLLPSDMAVALLEAQAGTGHIIDPATSARLTVDEAVRAGLVGPEFHEKLLSAEKAVTGYRDPYTGQSVSLFQALKKGLIPREQGLRLLDAQLSTGGIVDPSKSHRVPLDVACARGCLDEETSRALSAPRADAKAYSDPSTGEPATYGELQQRCRPDQLTGLSLLPLSEKAARARQEELYSELQARETFEKTPVEVPVGGFKGRTVTVWELISSEYFTAEQRQELLRQFRTGKVTVEKVIKILITIVEEVETLRQERLSFSGLRAPVPASELLASGVLSRAQFEQLKDGKTTVKDLSELGSVRTLLQGSGCLAGIYLEDTKEKVSIYEAMRRGLLRATTAALLLEAQAATGFLVDPVRNQRLYVHEAVKAGVVGPELHEQLLSAEKAVTGYRDPYSGSTISLFQAMQKGLVLRQHGIRLLEAQIATGGIIDPVHSHRVPVDVAYQRGYFSEEMNRVLADPSDDTKGFFDPNTHENLTYRQLLERCVEDPETGLRLLPLKGAEKAEVVETTQVYTEEETRRAFEETQIDIPGGGSHGGSTMSLWEVMQSDLIPEEQRAQLMADFQAGRVTKERMIIIIIEIIEKTEIIRQQGLASYDYVRRRLTAEDLFEARIISLETYNLLREGTRSLREALEAESAWCYLYGTGSVAGVYLPGSRQTLSIYQALKKGLLSAEVARLLLEAQAATGFLLDPVKGERLTVDEAVRKGLVGPELHDRLLSAERAVTGYRDPYTEQTISLFQAMKKELIPTEEALRLLDAQLATGGIVDPRLGFHLPLEVAYQRGYLNKDTHDQLSEPSEVRSYVDPSTDERLSYTQLLRRCRRDDGTGQLLLPLSDARKLTFRGLRKQITMEELVRSQVMDEATALQLREGLTSIEEVTKNLQKFLEGTSCIAGVFVDATKERLSVYQAMKKGIIRPGTAFELLEAQAATGYVIDPIKGLKLTVEEAVRMGIVGPEFKDKLLSAERAVTGYKDPYSGKLISLFQAMKKGLILKDHGIRLLEAQIATGGIIDPEESHRLPVEVAYKRGLFDEEMNEILTDPSDDTKGFFDPNTEENLTYLQLMERCITDPQTGLCLLPLKEKKRERKTSSKSSVRKRRVVIVDPETGKEMSVYEAYRKGLIDHQTYLELSEQECEWEEITISSSDGVVKSMIIDRRSGRQYDIDDAIAKNLIDRSALDQYRAGTLSITEFADMLSGNAGGFRSRSSSVGSSSSYPISPAVSRTQLASWSDPTEETGPVAGILDTETLEKVSITEAMHRNLVDNITGQRLLEAQACTGGIIDPSTGERFPVTDAVNKGLVDKIMVDRINLAQKAFCGFEDPRTKTKMSAAQALKKGWLYYEAGQRFLEVQYLTGGLIEPDTPGRVPLDEALQRGTVDARTAQKLRDVGAYSKYLTCPKTKLKISYKDALDRSMVEEGTGLRLLEAAAQSTKGYYSPYSVSGSGSTAGSRTGSRTGSRAGSRRGSFDATGSGFSMTFSSSSYSSSGYGRRYASGSSASLGGPESAVA

3) >gi|118572613|ref|NP_057417.3| serine/arginine repetitive matrix protein 2 [Homo sapiens] (299.4 kD)

MYNGIGLPTPRGSGTNGYVQRNLSLVRGRRGERPDYKGEEELRRLEAALVKRPNPDILDHERKRRVELRCLELEEMMEEQGYEEQQIQEKVATFRLMLLEKDVNPGGKEETPGQRPAVTETHQLAELNEKKNERLRAAFGISDSYVDGSSFDPQRRAREAKQPAPEPPKPYSLVRESSSSRSPTPKQKKKKKKKDRGRRSESSSPRRERKKSSKKKKHRSESESKKRKHRSPTPKSKRKSKDKKRKRSRSTTPAPKSRRAHRSTSADSASSSDTSRSRSRSAAAKTHTTALAGRSPSPASGRRGEGDAPFSEPGTTSTQRPSSPETATKQPSSPYEDKDKDKKEKSATRPSPSPERSSTGPEPPAPTPLLAERHGGSPQPLATTPLSQEPVNPPSEASPTRDRSPPKSPEKLPQSSSSESSPPSPQPTKVSRHASSSPESPKPAPAPGSHREISSSPTSKNRSHGRAKRDKSHSHTPSRRMGRSRSPATAKRGRSRSRTPTKRGHSRSRSPQWRRSRSAQRWGRSRSPQRRGRSRSPQRPGWSRSRNTQRRGRSRSARRGRSHSRSPATRGRSRSRTPARRGRSRSRTPARRRSRSRTPTRRRSRSRTPARRGRSRSRTPARRRSRTRSPVRRRSRSRSPARRSGRSRSRTPARRGRSRSRTPARRGRSRSRTPARRSGRSRSRTPARRGRSRSRTPRRGRSRSRSLVRRGRSHSRTPQRRGRSGSSSERKNKSRTSQRRSRSNSSPEMKKSRISSRRSRSLSSPRSKAKSRLSLRRSLSGSSPCPKQKSQTPPRRSRSGSSQPKAKSRTPPRRSRSSSSPPPKQKSKTPSRQSHSSSSPHPKVKSGTPPRQGSITSPQANEQSVTPQRRSCFESSPDPELKSRTPSRHSCSGSSPPRVKSSTPPRQSPSRSSSPQPKVKAIISPRQRSHSGSSSPSPSRVTSRTTPRRSRSVSPCSNVESRLLPRYSHSGSSSPDTKVKPETPPRQSHSGSISPYPKVKAQTPPGPSLSGSKSPCPQEKSKDSLVQSCPGSLSLCAGVKSSTPPGESYFGVSSLQLKGQSQTSPDHRSDTSSPEVRQSHSESPSLQSKSQTSPKGGRSRSSSPVTELASRSPIRQDRGEFSASPMLKSGMSPEQSRFQSDSSSYPTVDSNSLLGQSRLETAESKEKMALPPQEDATASPPRQKDKFSPFPVQDRPESSLVFKDTLRTPPRERSGAGSSPETKEQNSALPTSSQDEELMEVVEKSEEPAGQILSHLSSELKEMSTSNFESSPEVEERPAVSLTLDQSQSQASLEAVEVPSMASSWGGPHFSPEHKELSNSPLRENSFGSPLEFRNSGPLGTEMNTGFSSEVKEDLNGPFLNQLETDPSLDMKEQSTRSSGHSSSELSPDAVEKAGMSSNQSISSPVLDAVPRTPSRERSSSASSPEMKDGLPRTPSRRSRSGSSPGLRDGSGTPSRHSLSGSSPGMKDIPRTPSRGRSECDSSPEPKALPQTPRPRSRSPSSPELNNKCLTPQRERSGSESSVDQKTVARTPLGQRSRSGSSQELDVKPSASPQERSESDSSPDSKAKTRTPLRQRSRSGSSPEVDSKSRLSPRRSRSGSSPEVKDKPRAAPRAQSGSDSSPEPKAPAPRALPRRSRSGSSSKGRGPSPEGSSSTESSPEHPPKSRTARRGSRSSPEPKTKSRTPPRRRSSRSSPELTRKARLSRRSRSASSSPETRSRTPPRHRRSPSVSSPEPAEKSRSSRRRRSASSPRTKTTSRRGRSPSPKPRGLQRSRSRSRREKTRTTRRRDRSGSSQSTSRRRQRSRSRSRVTRRRRGGSGYHSRSPARQESSRTSSRRRRGRSRTPPTSRKRSRSRTSPAPWKRSRSRASPATHRRSRSRTPLISRRRSRSRTSPVSRRRSRSRTSVTRRRSRSRASPVSRRRSRSRTPPVTRRRSRSRTPTTRRRSRSRTPPVTRRRSRSRTPPVTRRRSRSRTSPITRRRSRSRTSPVTRRRSRSRTSPVTRRRSRSRTSPVTRRRSRSRTPPAIRRRSRSRTPLLPRKRSRSRSPLAIRRRSRSRTPRTARGKRSLTRSPPAIRRRSASGSSSDRSRSATPPATRNHSGSRTPPVALNSSRMSCFSRPSMSPTPLDRCRSPGMLEPLGSSRTPMSVLQQAGGSMMDGPGPRIPDHQRTSVPENHAQSRIALALTAISLGTARPPPSMSAAGLAARMSQVPAPVPLMSLRTAPAANLASRIPAASAAAMNLASARTPAIPTAVNLADSRTPAAAAAMNLASPRTAVAPSAVNLADPRTPTAPAVNLAGARTPAALAALSLTGSGTPPTAANYPSSSRTPQAPASANLVGPRSAHATAPVNIAGSRTAAALAPASLTSARMAPALSGANLTSPRVPLSAYERVSGRTSPPLLDRARSRTPPSAPSQSRMTSERAPSPSSRMGQAPSQSLLPPAQDQPRSPVPSAFSDQSRCLIAQTTPVAGSQSLSSGAVATTTSSAGDHNGMLSVPAPGVPHSDVGEPPASTGAQQPSALAALQPAKERRSSSSSSSSSSSSSSSSSSSSSSSSSGSSSSDSEGSSLPVQPEVALKRVPSPTPAPKEAVREGRPPEPTPAKRKRRSSSSSSSSSSSSSSSSSSSSSSSSSSSSSSSSSSSSSSSSSSPSPAKPGPQALPKPASPKKPPPGERRSRSPRKPIDSLRDSRSLSYSPVERRRPSPQPSPRDQQSSSSERGSRRGQRGDSRSPSHKRRRETPSPRPMRHRSSRSP

4) >gi|154759259|ref|NP_003118.2| spectrin alpha chain, non-erythrocytic 1 isoform 2 [Homo sapiens] (284.4 kD)

MDPSGVKVLETAEDIQERRQQVLDRYHRFKELSTLRRQKLEDSYRFQFFQRDAEELEKWIQEKLQIASDENYKDPTNLQGKLQKHQAFEAEVQANSGAIVKLDETGNLMISEGHFASETIRTRLMELHRQWELLLEKMREKGIKLLQAQKLVQYLRECEDVMDWINDKEAIVTSEELGQDLEHVEVLQKKFEEFQTDMAAHEERVNEVNQFAAKLIQEQHPEEELIKTKQDEVNAAWQRLKGLALQRQGKLFGAAEVQRFNRDVDETISWIKEKEQLMASDDFGRDLASVQALLRKHEGLERDLAALEDKVKALCAEADRLQQSHPLSATQIQVKREELITNWEQIRTLAAERHARLNDSYRLQRFLADFRDLTSWVTEMKALINADELASDVAGAEALLDRHQEHKGEIDAHEDSFKSADESGQALLAAGHYASDEVREKLTVLSEERAALLELWELRRQQYEQCMDLQLFYRDTEQVDNWMSKQEAFLLNEDLGDSLDSVEALLKKHEDFEKSLSAQEEKITALDEFATKLIQNNHYAMEDVATRRDALLSRRNALHERAMRRRAQLADSFHLQQFFRDSDELKSWVNEKMKTATDEAYKDPSNLQGKVQKHQAFEAELSANQSRIDALEKAGQKLIDVNHYAKDEVAARMNEVISLWKKLLEATELKGIKLREANQQQQFNRNVEDIELWLYEVEGHLASDDYGKDLTNVQNLQKKHALLEADVAAHQDRIDGITIQARQFQDAGHFDAENIKKKQEALVARYEALKEPMVARKQKLADSLRLQQLFRDVEDEETWIREKEPIAASTNRGKDLIGVQNLLKKHQALQAEIAGHEPRIKAVTQKGNAMVEEGHFAAEDVKAKLHELNQKWEALKAKASQRRQDLEDSLQAQQYFADANEAESWMREKEPIVGSTDYGKDEDSAEALLKKHEALMSDLSAYGSSIQALREQAQSCRQQVAPTDDETGKELVLALYDYQEKSPREVTMKKGDILTLLNSTNKDWWKVEVNDRQGFVPAAYVKKLDPAQSASRENLLEEQGSIALRQEQIDNQTRITKEAGSVSLRMKQVEELYHSLLELGEKRKGMLEKSCKKFMLFREANELQQWINEKEAALTSEEVGADLEQVEVLQKKFDDFQKDLKANESRLKDINKVAEDLESEGLMAEEVQAVQQQEVYGMMPRDETDSKTASPWKSARLMVHTVATFNSIKELNERWRSLQQLAEERSQLLGSAHEVQRFHRDADETKEWIEEKNQALNTDNYGHDLASVQALQRKHEGFERDLAALGDKVNSLGETAERLIQSHPESAEDLQEKCTELNQAWSSLGKRADQRKAKLGDSHDLQRFLSDFRDLMSWINGIRGLVSSDELAKDVTGAEALLERHQEHRTEIDARAGTFQAFEQFGQQLLAHGHYASPEIKQKLDILDQERADLEKAWVQRRMMLDQCLELQLFHRDCEQAENWMAAREAFLNTEDKGDSLDSVEALIKKHEDFDKAINVQEEKIAALQAFADQLIAAGHYAKGDISSRRNEVLDRWRRLKAQMIEKRSKLGESQTLQQFSRDVDEIEAWISEKLQTASDESYKDPTNIQSKHQKHQAFEAELHANADRIRGVIDMGNSLIERGACAGSEDAVKARLAALADQWQFLVQKSAEKSQKLKEANKQQNFNTGIKDFDFWLSEVEALLASEDYGKDLASVNNLLKKHQLLEADISAHEDRLKDLNSQADSLMTSSAFDTSQVKDKRDTINGRFQKIKSMAASRRAKLNESHRLHQFFRDMDDEESWIKEKKLLVGSEDYGRDLTGVQNLRKKHKRLEAELAAHEPAIQGVLDTGKKLSDDNTIGKEEIQQRLAQFVEHWKELKQLAAARGQRLEESLEYQQFVANVEEEEAWINEKMTLVASEDYGDTLAAIQGLLKKHEAFETDFTVHKDRVNDVCTNGQDLIKKNNHHEENISSKMKGLNGKVSDLEKAAAQRKAKLDENSAFLQFNWKADVVESWIGEKENSLKTDDYGRDLSSVQTLLTKQETFDAGLQAFQQEGIANITALKDQLLAAKHVQSKAIEARHASLMKRWSQLLANSAARKKKLLEAQSHFRKVEDLFLTFAKKASAFNSWFENAEEDLTDPVRCNSLEEIKALREAHDAFRSSLSSAQADFNQLAELDRQIKSFRVASNPYTWFTMEALEETWRNLQKIIKERELELQKEQRRQEENDKLRQEFAQHANAFHQWIQETRTYLLDGSCMVEESGTLESQLEATKRKHQEIRAMRSQLKKIEDLGAAMEEALILDNKYTEHSTVGLAQQWDQLDQLGMRMQHNLEQQIQARNTTGVTEEALKEFSMMFKHFDKDKSGRLNHQEFKSCLRSLGYDLPMVEEGEPDPEFEAILDTVDPNRDGHVSLQEYMAFMISRETENVKSSEEIESAFRALSSEGKPYVTKEELYQNLTREQADYCVSHMKPYVDGKGRELPTAFDYVEFTRSLFVN

5) >gi|153945728|ref|NP_005900.2| microtubule-associated protein 1B [Homo sapiens] (270.5 kD)

MATVVVEATEPEPSGSIANPAASTSPSLSHRFLDSKFYLLVVVGEIVTEEHLRRAIGNIELGIRSWDTNLIECNLDQELKLFVSRHSARFSPEVPGQKILHHRSDVLETVVLINPSDEAVSTEVRLMITDAARHKLLVLTGQCFENTGELILQSGSFSFQNFIEIFTDQEIGELLSTTHPANKASLTLFCPEEGDWKNSNLDRHNLQDFINIKLNSASILPEMEGLSEFTEYLSESVEVPSPFDILEPPTSGGFLKLSKPCCYIFPGGRGDSALFAVNGFNMLINGGSERKSCFWKLIRHLDRVDSILLTHIGDDNLPGINSMLQRKIAELEEEQSQGSTTNSDWMKNLISPDLGVVFLNVPENLKNPEPNIKMKRSIEEACFTLQYLNKLSMKPEPLFRSVGNTIDPVILFQKMGVGKLEMYVLNPVKSSKEMQYFMQQWTGTNKDKAEFILPNGQEVDLPISYLTSVSSLIVWHPANPAEKIIRVLFPGNSTQYNILEGLEKLKHLDFLKQPLATQKDLTGQVPTPVVKQTKLKQRADSRESLKPAAKPLPSKSVRKESKEETPEVTKVNHVEKPPKVESKEKVMVKKDKPIKTETKPSVTEKEVPSKEEPSPVKAEVAEKQATDVKPKAAKEKTVKKETKVKPEDKKEEKEKPKKEVAKKEDKTPIKKEEKPKKEEVKKEVKKEIKKEEKKEPKKEVKKETPPKEVKKEVKKEEKKEVKKEEKEPKKEIKKLPKDAKKSSTPLSEAKKPAALKPKVPKKEESVKKDSVAAGKPKEKGKIKVIKKEGKAAEAVAAAVGTGATTAAVMAAAGIAAIGPAKELEAERSLMSSPEDLTKDFEELKAEEVDVTKDIKPQLELIEDEEKLKETEPVEAYVIQKEREVTKGPAESPDEGITTTEGEGECEQTPEELEPVEKQGVDDIEKFEDEGAGFEESSETGDYEEKAETEEAEEPEEDGEEHVCVSASKHSPTEDEESAKAEADAYIREKRESVASGDDRAEEDMDEAIEKGEAEQSEEEADEEDKAEDAREEEYEPEKMEAEDYVMAVVDKAAEAGGAEEQYGFLTTPTKQLGAQSPGREPASSIHDETLPGGSESEATASDEENREDQPEEFTATSGYTQSTIEISSEPTPMDEMSTPRDVMSDETNNEETESPSQEFVNITKYESSLYSQEYSKPADVTPLNGFSEGSKTDATDGKDYNASASTISPPSSMEEDKFSRSALRDAYCSEVKASTTLDIKDSISAVSSEKVSPSKSPSLSPSPPSPLEKTPLGERSVNFSLTPNEIKVSAEAEVAPVSPEVTQEVVEEHCASPEDKTLEVVSPSQSVTGSAGHTPYYQSPTDEKSSHLPTEVIEKPPAVPVSFEFSDAKDENERASVSPMDEPVPDSESPIEKVLSPLRSPPLIGSESAYESFLSADDKASGRGAESPFEEKSGKQGSPDQVSPVSEMTSTSLYQDKQEGKSTDFAPIKEDFGQEKKTDDVEAMSSQPALALDERKLGDVSPTQIDVSQFGSFKEDTKMSISEGTVSDKSATPVDEGVAEDTYSHMEGVASVSTASVATSSFPEPTTDDVSPSLHAEVGSPHSTEVDDSLSVSVVQTPTTFQETEMSPSKEECPRPMSISPPDFSPKTAKSRTPVQDHRSEQSSMSIEFGQESPEQSLAMDFSRQSPDHPTVGAGVLHITENGPTEVDYSPSDMQDSSLSHKIPPMEEPSYTQDNDLSELISVSQVEASPSTSSAHTPSQIASPLQEDTLSDVAPPRDMSLYASLTSEKVQSLEGEKLSPKSDISPLTPRESSPLYSPTFSDSTSAVKEKTATCHSSSSPPIDAASAEPYGFRASVLFDTMQHHLALNRDLSTPGLEKDSGGKTPGDFSYAYQKPEETTRSPDEEDYDYESYEKTTRTSDVGGYYYEKIERTTKSPSDSGYSYETIGKTTKTPEDGDYSYEIIEKTTRTPEEGGYSYDISEKTTSPPEVSGYSYEKTERSRRLLDDISNGYDDSEDGGHTLGDPSYSYETTEKITSFPESEGYSYETSTKTTRTPDTSTYCYETAEKITRTPQASTYSYETSDLCYTAEKKSPSEARQDVDLCLVSSCEYKHPKTELSPSFINPNPLEWFASEEPTEESEKPLTQSGGAPPPPGGKQQGRQCDETPPTSVSESAPSQTDSDVPPETEECPSITADANIDSEDESETIPTDKTVTYKHMDPPPAPVQDRSPSPRHPDVSMVDPEALAIEQNLGKALKKDLKEKTKTKKPGTKTKSSSPVKKSDGKSKPLAASPKPAGLKESSDKVSRVASPKKKESVEKAAKPTTTPEVKAARGEEKDKETKNAANASASKSAKTATAGPGTTKTTKSSAVPPGLPVYLDLCYIPNHSNSKNVDVEFFKRVRSSYYVVSGNDPAAEEPSRAVLDALLEGKAQWGSNMQVTLIPTHDSEVMREWYQETHEKQQDLNIMVLASSSTVVMQDESFPACKIEL

6) >gi|7305053|ref|NP_038479.1| myoferlin isoform a [Homo sapiens] (234.6 kD)

MLRVIVESASNIPKTKFGKPDPIVSVIFKDEKKKTKKVDNELNPVWNEILEFDLRGIPLDFSSSLGIIVKDFETIGQNKLIGTATVALKDLTGDQSRSLPYKLISLLNEKGQDTGATIDLVIGYDPPSAPHPNDLSGPSVPGMGGDGEEDEGDEDRLDNAVRGPGPKGPVGTVSEAQLARRLTKVKNSRRMLSNKPQDFQIRVRVIEGRQLSGNNIRPVVKVHVCGQTHRTRIKRGNNPFFDELFFYNVNMTPSELMDEIISIRVYNSHSLRADCLMGEFKIDVGFVYDEPGHAVMRKWLLLNDPEDTSSGSKGYMKVSMFVLGTGDEPPPERRDRDNDSDDVESNLLLPAGIALRWVTFLLKIYRAEDIPQMDDAFSQTVKEIFGGNADKKNLVDPFVEVSFAGKKVCTNIIEKNANPEWNQVVNLQIKFPSVCEKIKLTIYDWDRLTKNDVVGTTYLHLSKIAASGGEVEDFSSSGTGAASYTVNTGETEVGFVPTFGPCYLNLYGSPREYTGFPDPYDELNTGKGEGVAYRGRILVELATFLEKTPPDKKLEPISNDDLLVVEKYQRRRKYSLSAVFHSATMLQDVGEAIQFEVSIGNYGNKFDTTCKPLASTTQYSRAVFDGNYYYYLPWAHTKPVVTLTSYWEDISHRLDAVNTLLAMAERLQTNIEALKSGIQGKIPANQLAELWLKLIDEVIEDTRYTLPLTEGKANVTVLDTQIRKLRSRSLSQIHEAAVRMRSEATDVKSTLAEIEDWLDKLMQLTEEPQNSMPDIIIWMIRGEKRLAYARIPAHQVLYSTSGENASGKYCGKTQTIFLKYPQEKNNGPKVPVELRVNIWLGLSAVEKKFNSFAEGTFTVFAEMYENQALMFGKWGTSGLVGRHKFSDVTGKIKLKREFFLPPKGWEWEGEWIVDPERSLLTEADAGHTEFTDEVYQNESRYPGGDWKPAEDTYTDANGDKAASPSELTCPPGWEWEDDAWSYDINRAVDEKGWEYGITIPPDHKPKSWVAAEKMYHTHRRRRLVRKRKKDLTQTASSTARAMEELQDQEGWEYASLIGWKFHWKQRSSDTFRRRRWRRKMAPSETHGAAAIFKLEGALGADTTEDGDEKSLEKQKHSATTVFGANTPIVSCNFDRVYIYHLRCYVYQARNLLALDKDSFSDPYAHICFLHRSKTTEIIHSTLNPTWDQTIIFDEVEIYGEPQTVLQNPPKVIMELFDNDQVGKDEFLGRSIFSPVVKLNSEMDITPKLLWHPVMNGDKACGDVLVTAELILRGKDGSNLPILPPQRAPNLYMVPQGIRPVVQLTAIEILAWGLRNMKNFQMASITSPSLVVECGGERVESVVIKNLKKTPNFPSSVLFMKVFLPKEELYMPPLVIKVIDHRQFGRKPVVGQCTIERLDRFRCDPYAGKEDIVPQLKASLLSAPPCRDIVIEMEDTKPLLASKLTEKEEEIVDWWSKFYASSGEHEKCGQYIQKGYSKLKIYNCELENVAEFEGLTDFSDTFKLYRGKSDENEDPSVVGEFKGSFRIYPLPDDPSVPAPPRQFRELPDSVPQECTVRIYIVRGLELQPQDNNGLCDPYIKITLGKKVIEDRDHYIPNTLNPVFGRMYELSCYLPQEKDLKISVYDYDTFTRDEKVGETIIDLENRFLSRFGSHCGIPEEYCVSGVNTWRDQLRPTQLLQNVARFKGFPQPILSEDGSRIRYGGRDYSLDEFEANKILHQHLGAPEERLALHILRTQGLVPEHVETRTLHSTFQPNISQGKLQMWVDVFPKSLGPPGPPFNITPRKAKKYYLRVIIWNTKDVILDEKSITGEEMSDIYVKGWIPGNEENKQKTDVHYRSLDGEGNFNWRFVFPFDYLPAEQLCIVAKKEHFWSIDQTEFRIPPRLIIQIWDNDKFSLDDYLGFLELDLRHTIIPAKSPEKCRLDMIPDLKAMNPLKAKTASLFEQKSMKGWWPCYAEKDGARVMAGKVEMTLEILNEKEADERPAGKGRDEPNMNPKLDLPNRPETSFLWFTNPCKTMKFIVWRRFKWVIIGLLFLLILLLFVAVLLYSLPNYLSMKIVKPNV

7) >gi|57164942|ref|NP_001008938.1| cytoskeleton-associated protein 5 isoform a [Homo sapiens] (225.4 kD)

MGDDSEWLKLPVDQKCEHKLWKARLSGYEEALKIFQKIKDEKSPEWSKFLGLIKKFVTDSNAVVQLKGLEAALVYVENAHVAGKTTGEVVSGVVSKVFNQPKAKAKELGIEICLMYIEIEKGEAVQEELLKGLDNKNPKIIVACIETLRKALSEFGSKIILLKPIIKVLPKLFESREKAVRDEAKLIAVEIYRWIRDALRPPLQNINSVQLKELEEEWVKLPTSAPRPTRFLRSQQELEAKLEQQQSAGGDAEGGGDDGDEVPQIDAYELLEAVEILSKLPKDFYDKIEAKKWQERKEALESVEVLIKNPKLEAGDYADLVKALKKVVGKDTNVMLVALAAKCLTGLAVGLRKKFGQYAGHVVPTILEKFKEKKPQVVQALQEAIDAIFLTTTLQNISEDVLAVMDNKNPTIKQQTSLFIARSFRHCTASTLPKSLLKPFCAALLKHINDSAPEVRDAAFEALGTALKVVGEKAVNPFLADVDKLKLDKIKECSEKVELIHGKKAGLAADKKEFKPLPGRTAASGAAGDKDTKDISAPKPGPLKKAPAAKAGGPPKKGKPAAPGGAGNTGTKNKKGLETKEIVEPELSIEVCEEKASAVLPPTCIQLLDSSNWKERLACMEEFQKAVELMDRTEMPCQALVRMLAKKPGWKETNFQVMQMKLHIVALIAQKGNFSKTSAQVVLDGLVDKIGDVKCGNNAKEAMTAIAEACMLPWTAEQVVSMAFSQKNPKNQSETLNWLSNAIKEFGFSGLNVKAFISNVKTALAATNPAVRTAAITLLGVMYLYVGPSLRMFFEDEKPALLSQIDAEFEKMQGQSPPAPTRGISKHSTSGTDEGEDGDEPDDGSNDVVDLLPRTEISDKITSELVSKIGDKNWKIRKEGLDEVAGIINDAKFIQPNIGELPTALKGRLNDSNKILVQQTLNILQQLAVAMGPNIKQHVKNLGIPIITVLGDSKNNVRAAALATVNAWAEQTGMKEWLEGEDLSEELKKENPFLRQELLGWLAEKLPTLRSTPTDLILCVPHLYSCLEDRNGDVRKKAQDALPFFMMHLGYEKMAKATGKLKPTSKDQVLAMLEKAKVNMPAKPAPPTKATSKPMGGSAPAKFQPASAPAEDCISSSTEPKPDPKKAKAPGLSSKAKSAQGKKMPSKTSLKEDEDKSGPIFIVVPNGKEQRMKDEKGLKVLKWNFTTPRDEYIEQLKTQMSSCVAKWLQDEMFHSDFQHHNKALAVMVDHLESEKEGVIGCLDLILKWLTLRFFDTNTSVLMKALEYLKLLFTLLSEEEYHLTENEASSFIPYLVVKVGEPKDVIRKDVRAILNRMCLVYPASKMFPFIMEGTKSKNSKQRAECLEELGCLVESYGMNVCQPTPGKALKEIAVHIGDRDNAVRNAALNTIVTVYNVHGDQVFKLIGNLSEKDMSMLEERIKRSAKRPSAAPIKQVEEKPQRAQNISSNANMLRKGPAEDMSSKLNQARSMSGHPEAAQMVRREFQLDLDEIENDNGTVRCEMPELVQHKLDDIFEPVLIPEPKIRAVSPHFDDMHSNTASTINFIISQVASGDINTSIQALTQIDEVLRQEDKAEAMSGHIDQFLIATFMQLRLIYNTHMADEKLEKDEIIKLYSCIIGNMISLFQIESLAREASTGVLKDLMHGLITLMLDSRIEDLEEGQQVIRSVNLLVVKVLEKSDQTNILSALLVLLQDSLLATASSPKFSELVMKCLWRMVRLLPDTINSINLDRILLDIHIFMKVFPKEKLKQCKSEFPIRTLKTLLHTLCKLKGPKILDHLTMIDNKNESELEAHLCRMMKHSMDQTGSKSDKETEKGASRIDEKSSKAKVNDFLAEIFKKIGSKENTKEGLAELYEYKKKYSDADIEPFLKNSSQFFQSYVERGLRVIEMEREGKGRISTSTGISPQMEVTCVPTPTSTVSSIGNTNGEEVGPSVYLERLKILRQRCGLDNTKQDDRPPLTSLLSKPAVPTVASSTDMLHSKLSQLRESREQHQHSDLDSNQTHSSGTVTSSSSTANIDDLKKRLERIKSSRK

8) >gi|242246985|ref|NP_009029.3| clathrin heavy chain 2 isoform 1 [Homo sapiens] (186.9 kD)

MAQILPVRFQEHFQLQNLGINPANIGFSTLTMESDKFICIREKVGEQAQVTIIDMSDPMAPIRRPISAESAIMNPASKVIALKAGKTLQIFNIEMKSKMKAHTMAEEVIFWKWVSVNTVALVTETAVYHWSMEGDSQPMKMFDRHTSLVGCQVIHYRTDEYQKWLLLVGISAQQNRVVGAMQLYSVDRKVSQPIEGHAAAFAEFKMEGNAKPATLFCFAVRNPTGGKLHIIEVGQPAAGNQPFVKKAVDVFFPPEAQNDFPVAMQIGAKHGVIYLITKYGYLHLYDLESGVCICMNRISADTIFVTAPHKPTSGIIGVNKKGQVLSVCVEEDNIVNYATNVLQNPDLGLRLAVRSNLAGAEKLFVRKFNTLFAQGSYAEAAKVAASAPKGILRTRETVQKFQSIPAQSGQASPLLQYFGILLDQGQLNKLESLELCHLVLQQGRKQLLEKWLKEDKLECSEELGDLVKTTDPMLALSVYLRANVPSKVIQCFAETGQFQKIVLYAKKVGYTPDWIFLLRGVMKISPEQGLQFSRMLVQDEEPLANISQIVDIFMENSLIQQCTSFLLDALKNNRPAEGLLQTWLLEMNLVHAPQVADAILGNKMFTHYDRAHIAQLCEKAGLLQQALEHYTDLYDIKRAVVHTHLLNPEWLVNFFGSLSVEDSVECLHAMLSANIRQNLQLCVQVASKYHEQLGTQALVELFESFKSYKGLFYFLGSIVNFSQDPDVHLKYIQAACKTGQIKEVERICRESSCYNPERVKNFLKEAKLTDQLPLIIVCDRFGFVHDLVLYLYRNNLQRYIEIYVQKVNPSRTPAVIGGLLDVDCSEEVIKHLIMAVRGQFSTDELVAEVEKRNRLKLLLPWLESQIQEGCEEPATHNALAKIYIDSNNSPECFLRENAYYDSSVVGRYCEKRDPHLACVAYERGQCDLELIKVCNENSLFKSEARYLVCRKDPELWAHVLEETNPSRRQLIDQVVQTALSETRDPEEISVTVKAFMTADLPNELIELLEKIVLDNSVFSEHRNLQNLLILTAIKADRTRVMEYISRLDNYDALDIASIAVSSALYEEAFTVFHKFDMNASAIQVLIEHIGNLDRAYEFAERCNEPAVWSQLAQAQLQKDLVKEAINSYIRGDDPSSYLEVVQSASRSNNWEDLVKFLQMARKKGRESYIETELIFALAKTSRVSELEDFINGPNNAHIQQVGDRCYEEGMYEAAKLLYSNVSNFARLASTLVHLGEYQAAVDNSRKASSTRTWKEVCFACMDGQEFRFAQLCGLHIVIHADELEELMCYYQDRGYFEELILLLEAALGLERAHMGMFTELAILYSKFKPQKMLEHLELFWSRVNIPKVLRAAEQAHLWAELVFLYDKYEEYDNAVLTMMSHPTEAWKEGQFKDIITKVANVELCYRALQFYLDYKPLLINDLLLVLSPRLDHTWTVSFFSKAGQLPLVKPYLRSVQSHNNKSVNEALNHLLTEEEDYQGLRASIDAYDNFDNISLAQQLEKHQLMEFRCIAAYLYKGNNWWAQSVELCKKDHLYKDAMQHAAESRDAELAQKLLQWFLEEGKRECFAACLFTCYDLLRPDMVLELAWRHNLVDLAMPYFIQVMREYLSKVDKLDALESLRKQEEHVTEPAPLVFDFDGHE

9) >gi|302699237|ref|NP_886553.3| eukaryotic translation initiation factor 4 gamma 1 isoform 1 [Homo sapiens] (175.4 kD)

MNKAPQSTGPPPAPSPGLPQPAFPPGQTAPVVFSTPQATQMNTPSQPRQHFYPSRAQPPSSAASRVQSAAPARPGPAAHVYPAGSQVMMIPSQISYPASQGAYYIPGQGRSTYVVPTQQYPVQPGAPGFYPGASPTEFGTYAGAYYPAQGVQQFPTGVAPAPVLMNQPPQIAPKRERKTIRIRDPNQGGKDITEEIMSGARTASTPTPPQTGGGLEPQANGETPQVAVIVRPDDRSQGAIIADRPGLPGPEHSPSESQPSSPSPTPSPSPVLEPGSEPNLAVLSIPGDTMTTIQMSVEESTPISRETGEPYRLSPEPTPLAEPILEVEVTLSKPVPESEFSSSPLQAPTPLASHTVEIHEPNGMVPSEDLEPEVESSPELAPPPACPSESPVPIAPTAQPEELLNGAPSPPAVDLSPVSEPEEQAKEVTASMAPPTIPSATPATAPSATSPAQEEEMEEEEEEEEGEAGEAGEAESEKGGEELLPPESTPIPANLSQNLEAAAATQVAVSVPKRRRKIKELNKKEAVGDLLDAFKEANPAVPEVENQPPAGSNPGPESEGSGVPPRPEEADETWDSKEDKIHNAENIQPGEQKYEYKSDQWKPLNLEEKKRYDREFLLGFQFIFASMQKPEGLPHISDVVLDKANKTPLRPLDPTRLQGINCGPDFTPSFANLGRTTLSTRGPPRGGPGGELPRGPQAGLGPRRSQQGPRKEPRKIIATVLMTEDIKLNKAEKAWKPSSKRTAADKDRGEEDADGSKTQDLFRRVRSILNKLTPQMFQQLMKQVTQLAIDTEERLKGVIDLIFEKAISEPNFSVAYANMCRCLMALKVPTTEKPTVTVNFRKLLLNRCQKEFEKDKDDDEVFEKKQKEMDEAATAEERGRLKEELEEARDIARRRSLGNIKFIGELFKLKMLTEAIMHDCVVKLLKNHDEESLECLCRLLTTIGKDLDFEKAKPRMDQYFNQMEKIIKEKKTSSRIRFMLQDVLDLRGSNWVPRRGDQGPKTIDQIHKEAEMEEHREHIKVQQLMAKGSDKRRGGPPGPPISRGLPLVDDGGWNTVPISKGSRPIDTSRLTKITKPGSIDSNNQLFAPGGRLSWGKGSSGGSGAKPSDAASEAARPATSTLNRFSALQQAVPTESTDNRRVVQRSSLSRERGEKAGDRGDRLERSERGGDRGDRLDRARTPATKRSFSKEVEERSRERPSQPEGLRKAASLTEDRDRGRDAVKREAALPPVSPLKAALSEEELEKKSKAIIEEYLHLNDMKEAVQCVQELASPSLLFIFVRHGVESTLERSAIAREHMGQLLHQLLCAGHLSTAQYYQGLYEILELAEDMEIDIPHVWLYLAELVTPILQEGGVPMGELFREITKPLRPLGKAASLLLEILGLLCKSMGPKKVGTLWREAGLSWKEFLPEGQDIGAFVAEQKVEYTLGEESEAPGQRALPSEELNRQLEKLLKEGSSNQRVFDWIEANLSEQQIVSNTLVRALMTAVCYSAIIFETPLRVDVAVLKARAKLLQKYLCDEQKELQALYALQALVVTLEQPPNLLRMFFDALYDEDVVKEDAFYSWESSKDPAEQQGKGVALKSVTAFFKWLREAEEESDHN

10) >gi|4503509|ref|NP_003741.1| eukaryotic translation initiation factor 3 subunit A [Homo sapiens] (166.5 kD)

MPAYFQRPENALKRANEFLEVGKKQPALDVLYDVMKSKKHRTWQKIHEPIMLKYLELCVDLRKSHLAKEGLYQYKNICQQVNIKSLEDVVRAYLKMAEEKTEAAKEESQQMVLDIEDLDNIQTPESVLLSAVSGEDTQDRTDRLLLTPWVKFLWESYRQCLDLLRNNSRVERLYHDIAQQAFKFCLQYTRKAEFRKLCDNLRMHLSQIQRHHNQSTAINLNNPESQSMHLETRLVQLDSAISMELWQEAFKAVEDIHGLFSLSKKPPKPQLMANYYNKVSTVFWKSGNALFHASTLHRLYHLSREMRKNLTQDEMQRMSTRVLLATLSIPITPERTDIARLLDMDGIIVEKQRRLATLLGLQAPPTRIGLINDMVRFNVLQYVVPEVKDLYNWLEVEFNPLKLCERVTKVLNWVREQPEKEPELQQYVPQLQNNTILRLLQQVSQIYQSIEFSRLTSLVPFVDAFQLERAIVDAARHCDLQVRIDHTSRTLSFGSDLNYATREDAPIGPHLQSMPSEQIRNQLTAMSSVLAKALEVIKPAHILQEKEEQHQLAVTAYLKNSRKEHQRILARRQTIEERKERLESLNIQREKEELEQREAELQKVRKAEEERLRQEAKEREKERILQEHEQIKKKTVRERLEQIKKTELGAKAFKDIDIEDLEELDPDFIMAKQVEQLEKEKKELQERLKNQEKKIDYFERAKRLEEIPLIKSAYEEQRIKDMDLWEQQEEERITTMQLEREKALEHKNRMSRMLEDRDLFVMRLKAARQSVYEEKLKQFEERLAEERHNRLEERKRQRKEERRITYYREKEEEEQRRAEEQMLKEREERERAERAKREEELREYQERVKKLEEVERKKRQRELEIEERERRREEERRLGDSSLSRKDSRWGDRDSEGTWRKGPEADSEWRRGPPEKEWRRGEGRDEDRSHRRDEERPRRLGDDEDREPSLRPDDDRVPRRGMDDDRGPRRGPEEDRFSRRGADDDRPSWRNTDDDRPPRRIADEDRGNWRHADDDRPPRRGLDEDRGSWRTADEDRGPRRGMDDDRGPRRGGADDERSSWRNADDDRGPRRGLDDDRGPRRGMDDDRGPRRGMDDDRGPRRGMDDDRGPRRGLDDDRGPWRNADDDRIPRRGAEDDRGPWRNMDDDRLSRRADDDRFPRRGDDSRPGPWRPLVKPGGWREKEKAREESWGPPRESRPSEEREWDREKERDRDNQDREENDKDPERERDRERDVDREDRFRRPRDEGGWRRGPAEESSSWRDSSRRDDRDRDDRRRERDDRRDLRERRDLRDDRDRRGPPLRSEREEVSSWRRADDRKDDRVEERDPPRRVPPPALSRDRERDRDREREGEKEKASWRAEKDRESLRRTKNETDEDGWTTVRR

11) >gi|55741719|ref|NP_055621.1| iporin [Homo sapiens] (161.1 kD)

MDSPPKLTGETLIVHHIPLVHCQVPDRQCCGGAGGGGGSTRPNPFCPPELGITQPDQDLGQADSLLFSSLHSAPGGTARSIDSTKSRSRDGRGPGAPKRHNPFLLQEGVGEPGLGDLYDDSIGDSATQQSFHLHGTGQPNFHLSSFQLPPSGPRVGRPWGTTRSRAGVVEGQEQEPVMTLDTQQCGTSHCCRPELEAETMELDECGGPGGSGSGGGASDTSGFSFDQEWKLSSDESPRNPGCSGSGDQHCRCSSTSSQSEAADQSMGYVSDSSCNSSDGVLVTFSTLYNKMHGTPRANLNSAPQSCSDSSFCSHSDPGAFYLDLQPSPFESKMSYESHHPESGGREGGYGCPHASSPELDANCNSYRPHCEPCPAVADLTACFQSQARLVVATQNYYKLVTCDLSSQSSPSPAGSSITSCSEEHTKISPPPGPGPDPGPSQPSEYYLFQKPEVQPEEQEAVSSSTQAAAAVGPTVLEGQVYTNTSPPNLSTGRQRSRSYDRSLQRSPPVRLGSLERMLSCPVRLSEGPAAMAGPGSPPRRVTSFAELAKGRKKTGGSGSPPLRVSVGDSSQEFSPIQEAQQDRGAPLDEGTCCSHSLPPMPLGPGMDLLGPDPSPPWSTQVCQGPHSSEMPPAGLRATGQGPLAQLMDPGPALPGSPANSHTQRDARARADGGGTESRPVLRYSKEQRPTTLPIQPFVFQHHFPKQLAKARALHSLSQLYSLSGCSRTQQPAPLAAPAAQVSVPAPSGEPQASTPRATGRGARKAGSEPETSRPSPLGSYSPIRSVGPFGPSTDSSASTSCSPPPEQPTATESLPPWSHSCPSAVRPATSQQPQKEDQKILTLTEYRLHGTGSLPPLGSWRSGLSRAESLARGGGEGSMATRPSNANHLSPQALKWREYRRKNPLGPPGLSGSLDRRSQEARLARRNPIFEFPGSLSAASHLNCRLNGQAVKPLPLTCPDFQDPFSLTEKPPAEFCLSPDGSSEAISIDLLQKKGLVKAVNIAVDLIVAHFGTSRDPGVKAKLGNSSVSPNVGHLVLKYLCPAVRAVLEDGLKAFVLDVIIGQRKNMPWSVVEASTQLGPSTKVLHGLYNKVSQFPELTSHTMRFNAFILGLLNIRSLEFWFNHLYNHEDIIQTHYQPWGFLSAAHTVCPGLFEELLLLLQPLALLPFSLDLLFQHRLLQSGQQQRQHKELLRVSQDLLLSAHSTLQLARARGQEGPGDVDRAAQGERVKGVGASEGGEEEEEEEETEEVAEAAGGSGRARWARGGQAGWWYQLMQSSQVYIDGSIEGSRFPRGSSNSSSEKKKGAGGGGPPQAPPPREGVVEGAEACPASEEALGRERGWPFWMGSPPDSVLAELRRSREREGPAASPAENEEGASEPSPGGIKWGHLFGSRKAQREARPTNRLPSDWLSLDKSMFQLVAQTVGSRREPEPKESLQEPHSPALPSSPPCEVQALCHHLATGPGQLSFHKGDILRVLGRAGGDWLRCSRGPDSGLVPLAYVTLTPTPSPTPGSSQN

12) >gi|358679311|ref|NP_001240626.1| protein LAP2 isoform 1 [Homo sapiens] (158.2 kD)

MTTKRSLFVRLVPCRCLRGEEETVTTLDYSHCSLEQVPKEIFTFEKTLEELYLDANQIEELPKQLFNCQSLHKLSLPDNDLTTLPASIANLINLRELDVSKNGIQEFPENIKNCKVLTIVEASVNPISKLPDGFSQLLNLTQLYLNDAFLEFLPANFGRLTKLQILELRENQLKMLPKTMNRLTQLERLDLGSNEFTEVPEVLEQLSGLKEFWMDANRLTFIPGFIGSLKQLTYLDVSKNNIEMVEEGISTCENLQDLLLSSNSLQQLPETIGSLKNITTLKIDENQLMYLPDSIGGLISVEELDCSFNEVEALPSSIGQLTNLRTFAADHNYLQQLPPEIGSWKNITVLFLHSNKLETLPEEMGDMQKLKVINLSDNRLKNLPFSFTKLQQLTAMWLSDNQSKPLIPLQKETDSETQKMVLTNYMFPQQPRTEDVMFISDNESFNPSLWEEQRKQRAQVAFECDEDKDEREAPPREGNLKRYPTPYPDELKNMVKTVQTIVHRLKDEETNEDSGRDLKPHEDQQDINKDVGVKTSESTTTVKSKVDEREKYMIGNSVQKISEPEAEISPGSLPVTANMKASENLKHIVNHDDVFEESEELSSDEEMKMAEMRPPLIETSINQPKVVALSNNKKDDTKETDSLSDEVTHNSNQNNSNCSSPSRMSDSVSLNTDSSQDTSLCSPVKQTHIDINSKIRQEDENFNSLLQNGDILNSSTEEKFKAHDKKDFNLPEYDLNVEERLVLIEKSVDSTATADDTHKLDHINMNLNKLITNDTFQPEIMERSKTQDIVLGTSFLSINSKEETEHLENGNKYPNLESVNKVNGHSEETSQSPNRTEPHDSDCSVDLGISKSTEDLSPQKSGPVGSVVKSHSITNMEIGGLKIYDILSDNGPQQPSTTVKITSAVDGKNIVRSKSATLLYDQPLQVFTGSSSSSDLISGTKAIFKFDSNHNPEEPNIIRGPTSGPQSAPQIYGPPQYNIQYSSSAAVKDTLWHSKQNPQIDHASFPPQLLPRSESTENQSYAKHSANMNFSNHNNVRANTAYHLHQRLGPARHGEMWAISPNDRLIPAVTRSTIQRQSSVSSTASVNLGDPGSTRRAQIPEGDYLSYREFHSAGRTPPMMPGSQRPLSARTYSIDGPNASRPQSARPSINEIPERTMSVSDFNYSRTSPSKRPNARVGSEHSLLDPPGKSKVPRDWREQVLRHIEAKKLEKKHPQTSSSGDPCQDGIFISGQQNYSSATLSHKDVPPDSLMKMPLSNGQMGQPLRPQANYSQIHHPPQASVARHPSREQLIDYLMLKVAHQPPYTQPHCSPRQGHELAKQEIRVRVEKDPELGFSISGGVGGRGNPFRPDDDGIFVTRVQPEGPASKLLQPGDKIIQANGYSFINIEHGQAVSLLKTFQNTVELIIVREVSS

13) >gi|31621305|ref|NP_573566.2| leucine-rich PPR motif-containing protein, mitochondrial precursor [Homo sapiens] (157.8 kD)

MAALLRSARWLLRAGAAPRLPLSLRLLPGGPGRLHAASYLPAARAGPVAGGLLSPARLYAIAAKEKDIQEESTFSSRKISNQFDWALMRLDLSVRRTGRIPKKLLQKVFNDTCRSGGLGGSHALLLLRSCGSLLPELKLEERTEFAHRIWDTLQKLGAVYDVSHYNALLKVYLQNEYKFSPTDFLAKMEEANIQPNRVTYQRLIASYCNVGDIEGASKILGFMKTKDLPVTEAVFSALVTGHARAGDMENAENILTVMRDAGIEPGPDTYLALLNAYAEKGDIDHVKQTLEKVEKSELHLMDRDLLQIIFSFSKAGYPQYVSEILEKVTCERRYIPDAMNLILLLVTEKLEDVALQILLACPVSKEDGPSVFGSFFLQHCVTMNTPVEKLTDYCKKLKEVQMHSFPLQFTLHCALLANKTDLAKALMKAVKEEGFPIRPHYFWPLLVGRRKEKNVQGIIEILKGMQELGVHPDQETYTDYVIPCFDSVNSARAILQENGCLSDSDMFSQAGLRSEAANGNLDFVLSFLKSNTLPISLQSIRSSLLLGFRRSMNINLWSEITELLYKDGRYCQEPRGPTEAVGYFLYNLIDSMSDSEVQAKEEHLRQYFHQLEKMNVKIPENIYRGIRNLLESYHVPELIKDAHLLVESKNLDFQKTVQLTSSELESTLETLKAENQPIRDVLKQLILVLCSEENMQKALELKAKYESDMVTGGYAALINLCCRHDKVEDALNLKEEFDRLDSSAVLDTGKYVGLVRVLAKHGKLQDAINILKEMKEKDVLIKDTTALSFFHMLNGAALRGEIETVKQLHEAIVTLGLAEPSTNISFPLVTVHLEKGDLSTALEVAIDCYEKYKVLPRIHDVLCKLVEKGETDLIQKAMDFVSQEQGEMVMLYDLFFAFLQTGNYKEAKKIIETPGIRARSARLQWFCDRCVANNQVETLEKLVELTQKLFECDRDQMYYNLLKLYKINGDWQRADAVWNKIQEENVIPREKTLRLLAEILREGNQEVPFDVPELWYEDEKHSLNSSSASTTEPDFQKDILIACRLNQKKGAYDIFLNAKEQNIVFNAETYSNLIKLLMSEDYFTQAMEVKAFAETHIKGFTLNDAANSRLIITQVRRDYLKEAVTTLKTVLDQQQTPSRLAVTRVIQALAMKGDVENIEVVQKMLNGLEDSIGLSKMVFINNIALAQIKNNNIDAAIENIENMLTSENKVIEPQYFGLAYLFRKVIEEQLEPAVEKISIMAERLANQFAIYKPVTDFFLQLVDAGKVDDARALLQRCGAIAEQTPILLLFLLRNSRKQGKASTVKSVLELIPELNEKEEAYNSLMKSYVSEKDVTSAKALYEHLTAKNTKLDDLFLKRYASLLKYAGEPVPFIEPPESFEFYAQQLRKLRENSS

14) >gi|24430146|ref|NP_005115.2| nuclear pore complex protein Nup153 isoform 2 [Homo sapiens] (153.8 kD)

MASGAGGVGGGGGGKIRTRRCHQGPIKPYQQGRQQHQGILSRVTESVKNIVPGWLQRYFNKNEDVCSCSTDTSEVPRWPENKEDHLVYADEESSNITDGRITPEPAVSNTEEPSTTSTASNYPDVLTRPSLHRSHLNFSMLESPALHCQPSTSSAFPIGSSGFSLVKEIKDSTSQHDDDNISTTSGFSSRASDKDITVSKNTSLPPLWSPEAERSHSLSQHTATSSKKPAFNLSAFGTLSPSLGNSSILKTSQLGDSPFYPGKTTYGGAAAAVRQSKLRNTPYQAPVRRQMKAKQLSAQSYGVTSSTARRILQSLEKMSSPLADAKRIPSIVSSPLNSPLDRSGIDITDFQAKREKVDSQYPPVQRLMTPKPVSIATNRSVYFKPSLTPSGEFRKTNQRIDNKCSTGYEKNMTPGQNREQRESGFSYPNFSLPAANGLSSGVGGGGGKMRRERTRFVASKPLEEEEMEVPVLPKISLPITSSSLPTFNFSSPEITTSSPSPINSSQALTNKVQMTSPSSTGSPMFKFSSPIVKSTEANVLPPSSIGFTFSVPVAKTAELSGSSSTLEPIISSSAHHVTTVNSTNCKKTPPEDCEGPFRPAEILKEGSVLDILKSPGFASPKIDSVAAQPTATSPVVYTRPAISSFSSSGIGFGESLKAGSSWQCDTCLLQNKVTDNKCIACQAAKLSPRDTAKQTGIETPNKSGKTTLSASGTGFGDKFKPVIGTWDCDTCLVQNKPEAIKCVACETPKPGTCVKRALTLTVVSESAETMTASSSSCTVTTGTLGFGDKFKRPIGSWECSVCCVSNNAEDNKCVSCMSEKPGSSVPASSSSTVPVSLPSGGSLGLEKFKKPEGSWDCELCLVQNKADSTKCLACESAKPGTKSGFKGFDTSSSSSNSAASSSFKFGVSSSSSGPSQTLTSTGNFKFGDQGGFKIGVSSDSGSINPMSEGFKFSKPIGDFKFGVSSESKPEEVKKDSKNDNFKFGLSSGLSNPVSLTPFQFGVSNLGQEEKKEELPKSSSAGFSFGTGVINSTPAPANTIVTSENKSSFNLGTIETKSASVAPFTCKTSEAKKEEMPATKGGFSFGNVEPASLPSASVFVLGRTEEKQQEPVTSTSLVFGKKADNEEPKCQPVFSFGNSEQTKDENSSKSTFSFSMTKPSEKESEQPAKATFAFGAQTSTTADQGAAKPVFSFLNNSSSSSSTPATSAGGGIFGSSTSSSNPPVATFVFGQSSNPVSSSAFGNTAESSTSQSLLFSQDSKLATTSSTGTAVTPFVFGPGASSNNTTTSGFGFGATTTSSSAGSSFVFGTGPSAPSASPAFGANQTPTFGQSQGASQPNPPGFGSISSSTALFPTGSQPAPPTFGTVSSSSQPPVFGQQPSQSAFGSGTTPNSSSAFQFGSSTTNFNFTNNSPSGVFTFGANSSTPAASAQPSGSGGFPFNQSPAAFTVGSNGKNVFSSSGTSFSGRKIKTAVRRRK

15) >gi|336020358|ref|NP_001229488.1| mitogen-activated protein kinase kinase kinase kinase 4 isoform 4 [Homo sapiens] (142 kD)

MANDSPAKSLVDIDLSSLRDPAGIFELVEVVGNGTYGQVYKGRHVKTGQLAAIKVMDVTEDEEEEIKLEINMLKKYSHHRNIATYYGAFIKKSPPGHDDQLWLVMEFCGAGSITDLVKNTKGNTLKEDWIAYISREILRGLAHLHIHHVIHRDIKGQNVLLTENAEVKLVDFGVSAQLDRTVGRRNTFIGTPYWMAPEVIACDENPDATYDYRSDLWSCGITAIEMAEGAPPLCDMHPMRALFLIPRNPPPRLKSKKWSKKFFSFIEGCLVKNYMQRPSTEQLLKHPFIRDQPNERQVRIQLKDHIDRTRKKRGEKDETEYEYSGSEEEEEEVPEQEGEPSSIVNVPGESTLRRDFLRLQQENKERSEALRRQQLLQEQQLREQEEYKRQLLAERQKRIEQQKEQRRRLEEQQRREREARRQQEREQRRREQEEKRRLEELERRRKEEEERRRAEEEKRRVEREQEYIRRQLEEEQRHLEVLQQQLLQEQAMLLECRWREMEEHRQAERLQRQLQQEQAYLLSLQHDHRRPHPQHSQQPPPPQQERSKPSFHAPEPKAHYEPADRAREVEDRFRKTNHSSPEAQSKQTGRVLEPPVPSRSESFSNGNSESVHPALQRPAEPQVPVRTTSRSPVLSRRDSPLQGSGQQNSQAGQRNSTSIEPRLLWERVEKLVPRPGSGSSSGSSNSGSQPGSHPGSQSGSGERFRVRSSSKSEGSPSQRLENAVKKPEDKKEVFRPLKPADLTALAKELRAVEDVRPPHKVTDYSSSSEESGTTDEEDDDVEQEGADESTSGPEDTRAASSLNLSNGETESVKTMIVHDDVESEPAMTPSKEGTLIVRQTQSASSTLQKHKSSSSFTPFIDPRLLQISPSSGTTVTSVVGFSCDGMRPEAIRQDPTRKGSVVNVNPTNTRPQSDTPEIRKYKKRFNSEILCAALWGVNLLVGTESGLMLLDRSGQGKVYPLINRRRFQQMDVLEGLNVLVTISGKKDKLRVYYLSWLRNKILHNDPEVEKKQGWTTVGDLEGCVHYKVVKYERIKFLVIALKSSVEVYAWAPKPYHKFMAFKSFGELVHKPLLVDLTVEEGQRLKVIYGSCAGFHAVDVDSGSVYDIYLPTHIQCSIKPHAIIILPNTDGMELLVCYEDEGVYVNTYGRITKDVVLQWGEMPTSVAYIRSNQTMGWGEKAIEIRSVETGHLDGVFMHKRAQRLKFLCERNDKVFFASVRSGGSSQVYFMTLGRTSLLSW

16) >gi|149999380|ref|NP_071934.3| inverted formin-2 isoform 1 [Homo sapiens] (135.5 kD)

MSVKEGAQRKWAALKEKLGPQDSDPTEANLESADPELCIRLLQMPSVVNYSGLRKRLEGSDGGWMVQFLEQSGLDLLLEALARLSGRGVARISDALLQLTCVSCVRAVMNSRQGIEYILSNQGYVRQLSQALDTSNVMVKKQVFELLAALCIYSPEGHVLTLDALDHYKTVCSQQYRFSIVMNELSGSDNVPYVVTLLSVINAVILGPEDLRARTQLRNEFIGLQLLDVLARLRDLEDADLLIQLEAFEEAKAEDEEELLRVSGGVDMSSHQEVFASLFHKVSCSPVSAQLLSVLQGLLHLEPTLRSSQLLWEALESLVNRAVLLASDAQECTLEEVVERLLSVKGRPRPSPLVKAHKSVQANLDQSQRGSSPQNTTTPKPSVEGQQPAAAAACEPVDHAQSESILKVSQPRALEQQASTPPPPPPPPLLPGSSAEPPPPPPPPPLPSVGAKALPTAPPPPPLPGLGAMAPPAPPLPPPLPGSCEFLPPPPPPLPGLGCPPPPPPLLPGMGWGPPPPPPPLLPCTCSPPVAGGMEEVIVAQVDHGLGSAWVPSHRRVNPPTLRMKKLNWQKLPSNVAREHNSMWASLSSPDAEAVEPDFSSIERLFSFPAAKPKEPTMVAPRARKEPKEITFLDAKKSLNLNIFLKQFKCSNEEVAAMIRAGDTTKFDVEVLKQLLKLLPEKHEIENLRAFTEERAKLASADHFYLLLLAIPCYQLRIECMLLCEGAAAVLDMVRPKAQLVLAACESLLTSRQLPIFCQLILRIGNFLNYGSHTGDADGFKISTLLKLTETKSQQNRVTLLHHVLEEAEKSHPDLLQLPRDLEQPSQAAGINLEIIRSEASSNLKKLLETERKVSASVAEVQEQYTERLQASISAFRALDELFEAIEQKQRELADYLCEDAQQLSLEDTFSTMKAFRDLFLRALKENKDRKEQAAKAERRKQQLAEEEARRPRGEDGKPVRKGPGKQEEVCVIDALLADIRKGFQLRKTARGRGDTDGGSKAASMDPPRATEPVATSNPAGDPVGSTRCPASEPGLDATTASESRGWDLVDAVTPGPQPTLEQLEEGGPRPLERRSSWYVDASDVLTTEDPQCPQPLEGAWPVTLGDAQALKPLKFSSNQPPAAGSSRQDAKDPTSLLGVLQAEADSTSEGLEDAVHSRGARPPAAGPGGDEDEDEEDTAPESALDTSLDKSFSEDAVTDSSGSGTLPRARGRASKGTGKRRKKRPSRSQEEVPPDSDDNKTKKLCVIQ

17) >gi|24431935|ref|NP_065393.1| reticulon-4 isoform A [Homo sapiens] (129.9 kD)

MEDLDQSPLVSSSDSPPRPQPAFKYQFVREPEDEEEEEEEEEEDEDEDLEELEVLERKPAAGLSAAPVPTAPAAGAPLMDFGNDFVPPAPRGPLPAAPPVAPERQPSWDPSPVSSTVPAPSPLSAAAVSPSKLPEDDEPPARPPPPPPASVSPQAEPVWTPPAPAPAAPPSTPAAPKRRGSSGSVDETLFALPAASEPVIRSSAENMDLKEQPGNTISAGQEDFPSVLLETAASLPSLSPLSAASFKEHEYLGNLSTVLPTEGTLQENVSEASKEVSEKAKTLLIDRDLTEFSELEYSEMGSSFSVSPKAESAVIVANPREEIIVKNKDEEEKLVSNNILHNQQELPTALTKLVKEDEVVSSEKAKDSFNEKRVAVEAPMREEYADFKPFERVWEVKDSKEDSDMLAAGGKIESNLESKVDKKCFADSLEQTNHEKDSESSNDDTSFPSTPEGIKDRSGAYITCAPFNPAATESIATNIFPLLGDPTSENKTDEKKIEEKKAQIVTEKNTSTKTSNPFLVAAQDSETDYVTTDNLTKVTEEVVANMPEGLTPDLVQEACESELNEVTGTKIAYETKMDLVQTSEVMQESLYPAAQLCPSFEESEATPSPVLPDIVMEAPLNSAVPSAGASVIQPSSSPLEASSVNYESIKHEPENPPPYEEAMSVSLKKVSGIKEEIKEPENINAALQETEAPYISIACDLIKETKLSAEPAPDFSDYSEMAKVEQPVPDHSELVEDSSPDSEPVDLFSDDSIPDVPQKQDETVMLVKESLTETSFESMIEYENKEKLSALPPEGGKPYLESFKLSLDNTKDTLLPDEVSTLSKKEKIPLQMEELSTAVYSNDDLFISKEAQIRETETFSDSSPIEIIDEFPTLISSKTDSFSKLAREYTDLEVSHKSEIANAPDGAGSLPCTELPHDLSLKNIQPKVEEKISFSDDFSKNGSATSKVLLLPPDVSALATQAEIESIVKPKVLVKEAEKKLPSDTEKEDRSPSAIFSAELSKTSVVDLLYWRDIKKTGVVFGASLFLLLSLTVFSIVSVTAYIALALLSVTISFRIYKGVIQAIQKSDEGHPFRAYLESEVAISEELVQKYSNSALGHVNCTIKELRRLFLVDDLVDSLKFAVLMWVFTYVGALFNGLTLLILALISLFSVPVIYERHQAQIDHYLGLANKNVKDAMAKIQAKIPGLKRKAE

18) >gi|4507943|ref|NP_003391.1| exportin-1 [Homo sapiens] (123.3 kD)

MPAIMTMLADHAARQLLDFSQKLDINLLDNVVNCLYHGEGAQQRMAQEVLTHLKEHPDAWTRVDTILEFSQNMNTKYYGLQILENVIKTRWKILPRNQCEGIKKYVVGLIIKTSSDPTCVEKEKVYIGKLNMILVQILKQEWPKHWPTFISDIVGASRTSESLCQNNMVILKLLSEEVFDFSSGQITQVKSKHLKDSMCNEFSQIFQLCQFVMENSQNAPLVHATLETLLRFLNWIPLGYIFETKLISTLIYKFLNVPMFRNVSLKCLTEIAGVSVSQYEEQFVTLFTLTMMQLKQMLPLNTNIRLAYSNGKDDEQNFIQNLSLFLCTFLKEHDQLIEKRLNLRETLMEALHYMLLVSEVEETEIFKICLEYWNHLAAELYRESPFSTSASPLLSGSQHFDVPPRRQLYLPMLFKVRLLMVSRMAKPEEVLVVENDQGEVVREFMKDTDSINLYKNMRETLVYLTHLDYVDTERIMTEKLHNQVNGTEWSWKNLNTLCWAIGSISGAMHEEDEKRFLVTVIKDLLGLCEQKRGKDNKAIIASNIMYIVGQYPRFLRAHWKFLKTVVNKLFEFMHETHDGVQDMACDTFIKIAQKCRRHFVQVQVGEVMPFIDEILNNINTIICDLQPQQVHTFYEAVGYMIGAQTDQTVQEHLIEKYMLLPNQVWDSIIQQATKNVDILKDPETVKQLGSILKTNVRACKAVGHPFVIQLGRIYLDMLNVYKCLSENISAAIQANGEMVTKQPLIRSMRTVKRETLKLISGWVSRSNDPQMVAENFVPPLLDAVLIDYQRNVPAAREPEVLSTMAIIVNKLGGHITAEIPQIFDAVFECTLNMINKDFEEYPEHRTNFFLLLQAVNSHCFPAFLAIPPTQFKLVLDSIIWAFKHTMRNVADTGLQILFTLLQNVAQEEAAAQSFYQTYFCDILQHIFSVVTDTSHTAGLTMHASILAYMFNLVEEGKISTSLNPGNPVNNQIFLQEYVANLLKSAFPHLQDAQVKLFVTGLFSLNQDIPAFKEHLRDFLVQIKEFAGEDTSDLFLEEREIALRQADEEKHKRQMSVPGIFNPHEIPEEMCD

19) >gi|47519639|ref|NP_002366.2| microtubule-associated protein 4 isoform 1 [Homo sapiens] (120.9 kD)

MADLSLADALTEPSPDIEGEIKRDFIATLEAEAFDDVVGETVGKTDYIPLLDVDEKTGNSESKKKPCSETSQIEDTPSSKPTLLANGGHGVEGSDTTGSPTEFLEEKMAYQEYPNSQNWPEDTNFCFQPEQVVDPIQTDPFKMYHDDDLADLVFPSSATADTSIFAGQNDPLKDSYGMSPCNTAVVPQGWSVEALNSPHSESFVSPEAVAEPPQPTAVPLELAKEIEMASEERPPAQALEIMMGLKTTDMAPSKETEMALAKDMALATKTEVALAKDMESPTKLDVTLAKDMQPSMESDMALVKDMELPTEKEVALVKDVRWPTETDVSSAKNVVLPTETEVAPAKDVTLLKETERASPIKMDLAPSKDMGPPKENKKETERASPIKMDLAPSKDMGPPKENKIVPAKDLVLLSEIEVAQANDIISSTEISSAEKVALSSETEVALARDMTLPPETNVILTKDKALPLEAEVAPVKDMAQLPETEIAPAKDVAPSTVKEVGLLKDMSPLSETEMALGKDVTPPPETEVVLIKNVCLPPEMEVALTEDQVPALKTEAPLAKDGVLTLANNVTPAKDVPPLSETEATPVPIKDMEIAQTQKGISEDSHLESLQDVGQSAAPTFMISPETVTGTGKKCSLPAEEDSVLEKLGERKPCNSQPSELSSETSGIARPEEGRPVVSGTGNDITTPPNKELPPSPEKKTKPLATTQPAKTSTSKAKTQPTSLPKQPAPTTIGGLNKKPMSLASGLVPAAPPKRPAVASARPSILPSKDVKPKPIADAKAPEKRASPSKPASAPASRSGSKSTQTVAKTTTAAAVASTGPSSRSPSTLLPKKPTAIKTEGKPAEVKKMTAKSVPADLSRPKSTSTSSMKKTTTLSGTAPAAGVVPSRVKATPMPSRPSTTPFIDKKPTSAKPSSTTPRLSRLATNTSAPDLKNVRSKVGSTENIKHQPGGGRAKVEKKTEAAATTRKPESNAVTKTAGPIASAQKQPAGKVQIVSKKVSYSHIQSKCGSKDNIKHVPGGGNVQIQNKKVDISKVSSKCGSKANIKHKPGGGDVKIESQKLNFKEKAQAKVGSLDNVGHLPAGGAVKTEGGGSEAPLCPGPPAGEEPAISEAAPEAGAPTSASGLNGHPTLSGGGDQREAQTLDSQIQETSI

20) >gi|149158692|ref|NP_004630.3| large proline-rich protein BAG6 isoform a [Homo sapiens] (119.3 kD)

MEPNDSTSTAVEEPDSLEVLVKTLDSQTRTFIVGAQMNVKEFKEHIAASVSIPSEKQRLIYQGRVLQDDKKLQEYNVGGKVIHLVERAPPQTHLPSGASSGTGSASATHGGGSPPGTRGPGASVHDRNANSYVMVGTFNLPSDGSAVDVHINMEQAPIQSEPRVRLVMAQHMIRDIQTLLSRMETLPYLQCRGGPQPQHSQPPPQPPAVTPEPVALSSQTSEPVESEAPPREPMEAEEVEERAPAQNPELTPGPAPAGPTPAPETNAPNHPSPAEYVEVLQELQRLESRLQPFLQRYYEVLGAAATTDYNNNHEGREEDQRLINLVGESLRLLGNTFVALSDLRCNLACTPPRHLHVVRPMSHYTTPMVLQQAAIPIQINVGTTVTMTGNGTRPPPTPNAEAPPPGPGQASSVAPSSTNVESSAEGAPPPGPAPPPATSHPRVIRISHQSVEPVVMMHMNIQDSGTQPGGVPSAPTGPLGPPGHGQTLGQQVPGFPTAPTRVVIARPTPPQARPSHPGGPPVSGTLQGAGLGTNASLAQMVSGLVGQLLMQPVLVAQGTPGMAPPPAPATASASAGTTNTATTAGPAPGGPAQPPPTPQPSMADLQFSQLLGNLLGPAGPGAGGSGVASPTITVAMPGVPAFLQGMTDFLQATQTAPPPPPPPPPPPPAPEQQTMPPPGSPSGGAGSPGGLGLESLSPEFFTSVVQGVLSSLLGSLGARAGSSESIAAFIQRLSGSSNIFEPGADGALGFFGALLSLLCQNFSMVDVVMLLHGHFQPLQRLQPQLRSFFHQHYLGGQEPTPSNIRMATHTLITGLEEYVRESFSLVQVQPGVDIIRTNLEFLQEQFNSIAAHVLHCTDSGFGARLLELCNQGLFECLALNLHCLGGQQMELAAVINGRIRRMSRGVNPSLVSWLTTMMGLRLQVVLEHMPVGPDAILRYVRRVGDPPQPLPEEPMEVQGAERASPEPQRENASPAPGTTAEEAMSRGPPPAPEGGSRDEQDGASAETEPWAAAVPPEWVPIIQQDIQSQRKVKPQPPLSDAYLSGMPAKRRKTMQGEGPQLLLSEAVSRAAKAAGARPLTSPESLSRDLEAPEVQESYRQQLRSDIQKRLQEDPNYSPQRFPNAQRAFADDP

21) >gi|24476013|ref|NP_722560.1| focal adhesion kinase 1 isoform a [Homo sapiens] (119.2 kD)

MAAAYLDPNLNHTPNSSTKTHLGTGMERSPGAMERVLKVFHYFESNSEPTTWASIIRHGDATDVRGIIQKIVDSHKVKHVACYGFRLSHLRSEEVHWLHVDMGVSSVREKYELAHPPEEWKYELRIRYLPKGFLNQFTEDKPTLNFFYQQVKSDYMLEIADQVDQEIALKLGCLEIRRSYWEMRGNALEKKSNYEVLEKDVGLKRFFPKSLLDSVKAKTLRKLIQQTFRQFANLNREESILKFFEILSPVYRFDKECFKCALGSSWIISVELAIGPEEGISYLTDKGCNPTHLADFTQVQTIQYSNSEDKDRKGMLQLKIAGAPEPLTVTAPSLTIAENMADLIDGYCRLVNGTSQSFIIRPQKEGERALPSIPKLANSEKQGMRTHAVSVSETDDYAEIIDEEDTYTMPSTRDYEIQRERIELGRCIGEGQFGDVHQGIYMSPENPALAVAIKTCKNCTSDSVREKFLQEALTMRQFDHPHIVKLIGVITENPVWIIMELCTLGELRSFLQVRKYSLDLASLILYAYQLSTALAYLESKRFVHRDIAARNVLVSSNDCVKLGDFGLSRYMEDSTYYKASKGKLPIKWMAPESINFRRFTSASDVWMFGVCMWEILMHGVKPFQGVKNNDVIGRIENGERLPMPPNCPPTLYSLMTKCWAYDPSRRPRFTELKAQLSTILEEEKAQQEERMRMESRRQATVSWDSGGSDEAPPKPSRPGYPSPRSSEGFYPSPQHMVQTNHYQVSGYPGSHGITAMAGSIYPGQASLLDQTDSWNHRPQEIAMWQPNVEDSTVLDLRGIGQVLPTHLMEERLIRQQQEMEEDQRWLEKEERFLKPDVRLSRGSIDREDGSLQGPIGNQHIYQPVGKPDPAAPPKKPPRPGAPGHLGSLASLSSPADSYNEGVKLQPQEISPPPTANLDRSNDKVYENVTGLVKAVIEMSSKIQPAPPEEYVPMVKEVGLALRTLLATVDETIPLLPASTHREIEMAQKLLNSDLGELINKMKLAQQYVMTSLQQEYKKQMLTAAHALAVDAKNLLDVIDQARLKMLGQTRPH

22) >gi|23510340|ref|NP_695012.1| ubiquitin-like modifier-activating enzyme 1 [Homo sapiens] (177.8 kD)

MSSSPLSKKRRVSGPDPKPGSNCSPAQSVLSEVPSVPTNGMAKNGSEADIDEGLYSRQLYVLGHEAMKRLQTSSVLVSGLRGLGVEIAKNIILGGVKAVTLHDQGTAQWADLSSQFYLREEDIGKNRAEVSQPRLAELNSYVPVTAYTGPLVEDFLSGFQVVVLTNTPLEDQLRVGEFCHNRGIKLVVADTRGLFGQLFCDFGEEMILTDSNGEQPLSAMVSMVTKDNPGVVTCLDEARHGFESGDFVSFSEVQGMVELNGNQPMEIKVLGPYTFSICDTSNFSDYIRGGIVSQVKVPKKISFKSLVASLAEPDFVVTDFAKFSRPAQLHIGFQALHQFCAQHGRPPRPRNEEDAAELVALAQAVNARALPAVQQNNLDEDLIRKLAYVAAGDLAPINAFIGGLAAQEVMKACSGKFMPIMQWLYFDALECLPEDKEVLTEDKCLQRQNRYDGQVAVFGSDLQEKLGKQKYFLVGAGAIGCELLKNFAMIGLGCGEGGEIIVTDMDTIEKSNLNRQFLFRPWDVTKLKSDTAAAAVRQMNPHIRVTSHQNRVGPDTERIYDDDFFQNLDGVANALDNVDARMYMDRRCVYYRKPLLESGTLGTKGNVQVVIPFLTESYSSSQDPPEKSIPICTLKNFPNAIEHTLQWARDEFEGLFKQPAENVNQYLTDPKFVERTLRLAGTQPLEVLEAVQRSLVLQRPQTWADCVTWACHHWHTQYSNNIRQLLHNFPPDQLTSSGAPFWSGPKRCPHPLTFDVNNPLHLDYVMAAANLFAQTYGLTGSQDRAAVATFLQSVQVPEFTPKSGVKIHVSDQELQSANASVDDSRLEELKATLPSPDKLPGFKMYPIDFEKDDDSNFHMDFIVAASNLRAENYDIPSADRHKSKLIAGKIIPAIATTTAAVVGLVCLELYKVVQGHRQLDSYKNGFLNLALPFFGFSEPLAAPRHQYYNQEWTLWDRFEVQGLQPNGEEMTLKQFLDYFKTEHKLEITMLSQGVSMLYSFFMPAAKLKERLDQPMTEIVSRVSKRKLGRHVRALVLELCCNDESGEDVEVPYVRYTIR

23) >gi|34101286|ref|NP_057191.2| zinc finger RNA-binding protein [Homo sapiens] (116.9 kD)

MIPICPVVSFTYVPSRLGEDAKMATGNYFGFTHSGAAAAAAAAQYSQQPASGVAYSHPTTVASYTVHQAPVAAHTVTAAYAPAAATVAVARPAPVAVAAAATAAAYGGYPTAHTATDYGYTQRQQEAPPPPPPATTQNYQDSYSYVRSTAPAVAYDSKQYYQQPTATAAAVAAAAQPQPSVAETYYQTAPKAGYSQGATQYTQAQQTRQVTAIKPATPSPATTTFSIYPVSSTVQPVAAAATVVPSYTQSATYSTTAVTYSGTSYSGYEAAVYSAASSYYQQQQQQQKQAAAAAAAAAATAAWTGTTFTKKAPFQNKQLKPKQPPKPPQIHYCDVCKISCAGPQTYKEHLEGQKHKKKEAALKASQNTSSSNSSTRGTQNQLRCELCDVSCTGADAYAAHIRGAKHQKVVKLHTKLGKPIPSTEPNVVSQATSSTAVSASKPTASPSSIAANNCTVNTSSVATSSMKGLTTTGNSSLNSTSNTKVSAVPTNMAAKKTSTPKINFVGGNKLQSTGNKAEDIKGTECVKSTPVTSAVQIPEVKQDTVSEPVTPASLAALQSDVQPVGHDYVEEVRNDEGKVIRFHCKLCECSFNDPNAKEMHLKGRRHRLQYKKKVNPDLQVEVKPSIRARKIQEEKMRKQMQKEEYWRRREEEERWRMEMRRYEEDMYWRRMEEEQHHWDDRRRMPDGGYPHGPPGPLGLLGVRPGMPPQPQGPAPLRRPDSSDDRYVMTKHATIYPTEEELQAVQKIVSITERALKLVSDSLSEHEKNKNKEGDDKKEGGKDRALKGVLRVGVLAKGLLLRGDRNVNLVLLCSEKPSKTLLSRIAENLPKQLAVISPEKYDIKCAVSEAAIILNSCVEPKMQVTITLTSPIIREENMREGDVTSGMVKDPPDVLDRQKCLDALAALRHAKWFQARANGLQSCVIIIRILRDLCQRVPTWSDFPSWAMELLVEKAISSASSPQSPGDALRRVFECISSGIILKGSPGLLDPCEKDPFDTLATMTDQQREDITSSAQFALRLLAFRQIHKVLGMDPLPQMSQRFNIHNNRKRRRDSDGVDGFEAEGKKDKKDYDNF

24) >gi|188497758|ref|NP_055662.3| ubiquitin-associated protein 2-like isoform a [Homo sapiens] (114.5 kD)

MMTSVGTNRARGNWEQPQNQNQTQHKQRPQATAEQIRLAQMISDHNDADFEEKVKQLIDITGKNQDECVIALHDCNGDVNRAINVLLEGNPDTHSWEMVGKKKGVSGQKDGGQTESNEEGKENRDRDRDYSRRRGGPPRRGRGASRGREFRGQENGLDGTKSGGPSGRGTERGRRGRGRGRGGSGRRGGRFSAQGMGTFNPADYAEPANTDDNYGNSSGNTWNNTGHFEPDDGTSAWRTATEEWGTEDWNEDLSETKIFTASNVSSVPLPAENVTITAGQRIDLAVLLGKTPSTMENDSSNLDPSQAPSLAQPLVFSNSKQTAISQPASGNTFSHHSMVSMLGKGFGDVGEAKGGSTTGSQFLEQFKTAQALAQLAAQHSQSGSTTTSSWDMGSTTQSPSLVQYDLKNPSDSAVHSPFTKRQAFTPSSTMMEVFLQEKSPAVATSTAAPPPPSSPLPSKSTSAPQMSPGSSDNQSSSPQPAQQKLKQQKKKASLTSKIPALAVEMPGSADISGLNLQFGALQFGSEPVLSDYESTPTTSASSSQAPSSLYTSTASESSSTISSNQSQESGYQSGPIQSTTYTSQNNAQGPLYEQRSTQTRRYPSSISSSPQKDLTQAKNGFSSVQATQLQTTQSVEGATGSAVKSDSPSTSSIPPLNETVSAASLLTTTNQHSSSLGGLSHSEEIPNTTTTQHSSTLSTQQNTLSSSTSSGRTSTSTLLHTSVESEANLHSSSSTFSTTSSTVSAPPPVVSVSSSLNSGSSLGLSLGSNSTVTASTRSSVATTSGKAPPNLPPGVPPLLPNPYIMAPGLLHAYPPQVYGYDDLQMLQTRFPLDYYSIPFPTPTTPLTGRDGSLASNPYSGDLTKFGRGDASSPAPATTLAQPQQNQTQTHHTTQQTFLNPALPPGYSYTSLPYYTGVPGLPSTFQYGPAVFPVAPTSSKQHGVNVSVNASATPFQQPSGYGSHGYNTGVSVTSSNTGVPDISGSVYSKTQQSFEKQGFHSGTPAASFNLPSALGSGGPINPATAAAYPPAPFMHILTPHQQPHSQILHHHLQQDGQTGSGQRSQTSSIPQKPQTNKSAYNSYSWGAN

25) >gi|388240768|ref|NP_001252518.1| reticulon-3 isoform e [Homo sapiens] (112.5 kD)

MAEPSAATQSHSISSSSFGAEPSAPGGGGSPGACPALGTKSCSSSCADSFVSSSSSQPVSLFSTSQEGLSSLCSDEPSSEIMTSSFLSSSEIHNTGLTILHGEKSHVLGSQPILAKEGKDHLDLLDMKKMEKPQGTSNNVSDSSVSLAAGVHCDRPSIPASFPEHPAFLSKKIGQVEEQIDKETKNPNGVSSREAKTALDADDRFTLLTAQKPPTEYSKVEGIYTYSLSPSKVSGDDVIEKDSPESPFEVIIDKAAFDKEFKDSYKESTDDFGSWSVHTDKESSEDISETNDKLFPLRNKEAGRYPMSALLSRQFSHTNAALEEVSRCVNDMHNFTNEILTWDLVPQVKQQTDKSSDCITKTTGLDMSEYNSEIPVVNLKTSTHQKTPVCSIDGSTPITKSTGDWAEASLQQENAITGKPVPDSLNSTKEFSIKGVQGNMQKQDDTLAELPGSPPEKCDSLGSGVATVKVVLPDDHLKDEMDWQSSALGEITEADSSGESDDTVIEDITADTSFENNKIQAEKPVSIPSAVVKTGEREIKEIPSCEREEKTSKNFEELVSDSELHQDQPDILGRSPASEAACSKVPDTNVSLEDVSEVAPEKPITTENPKLPSTVSPNVFNETEFSLNVTTSAYLESLHGKNVKHIDDSSPEDLIAAFTETRDKGIVDSERNAFKAISEKMTDFKTTPPVEVLHENESGGSEIKDIGSKYSEQSKETNGSEPLGVFPTQGTPVASLDLEQEQLTIKALKELGERQVEKSTSAQRDAELPSEEVLKQTFTFAPESWPQRSYDILERNVKNGSDLGISQKPITIRETTRVDAVSSLSKTELVKKHVLARLLTDFSVHDLIFWRDVKKTGFVFGTTLIMLLSLAAFSVISVVSYLILALLSVTISFRIYKSVIQAVQKSEEGHPFKAYLDVDITLSSEAFHNYMNAAMVHINRALKLIIRLFLVEDLVDSLKLAVFMWLMTYVGAVFNGITLLILAELLIFSVPIVYEKYKTQIDHYVGIARDQTKSIVEKIQAKLPGIAKKKAE

26) >gi|22748667|ref|NP_689509.1| sodium/potassium-transporting ATPase subunit alpha-3 isoform 1 [Homo sapiens] (111.7 kD)

MGDKKDDKDSPKKNKGKERRDLDDLKKEVAMTEHKMSVEEVCRKYNTDCVQGLTHSKAQEILARDGPNALTPPPTTPEWVKFCRQLFGGFSILLWIGAILCFLAYGIQAGTEDDPSGDNLYLGIVLAAVVIITGCFSYYQEAKSSKIMESFKNMVPQQALVIREGEKMQVNAEEVVVGDLVEIKGGDRVPADLRIISAHGCKVDNSSLTGESEPQTRSPDCTHDNPLETRNITFFSTNCVEGTARGVVVATGDRTVMGRIATLASGLEVGKTPIAIEIEHFIQLITGVAVFLGVSFFILSLILGYTWLEAVIFLIGIIVANVPEGLLATVTVCLTLTAKRMARKNCLVKNLEAVETLGSTSTICSDKTGTLTQNRMTVAHMWFDNQIHEADTTEDQSGTSFDKSSHTWVALSHIAGLCNRAVFKGGQDNIPVLKRDVAGDASESALLKCIELSSGSVKLMRERNKKVAEIPFNSTNKYQLSIHETEDPNDNRYLLVMKGAPERILDRCSTILLQGKEQPLDEEMKEAFQNAYLELGGLGERVLGFCHYYLPEEQFPKGFAFDCDDVNFTTDNLCFVGLMSMIDPPRAAVPDAVGKCRSAGIKVIMVTGDHPITAKAIAKGVGIISEGNETVEDIAARLNIPVSQVNPRDAKACVIHGTDLKDFTSEQIDEILQNHTEIVFARTSPQQKLIIVEGCQRQGAIVAVTGDGVNDSPALKKADIGVAMGIAGSDVSKQAADMILLDDNFASIVTGVEEGRLIFDNLKKSIAYTLTSNIPEITPFLLFIMANIPLPLGTITILCIDLGTDMVPAISLAYEAAESDIMKRQPRNPRTDKLVNERLISMAYGQIGMIQALGGFFSYFVILAENGFLPGNLVGIRLNWDDRTVNDLEDSYGQQWTYEQRKVVEFTCHTAFFVSIVVVQWADLIICKTRRNSVFQQGMKNKILIFGLFEETALAAFLSYCPGMDVALRMYPLKPSWWFCAFPYSFLIFVYDEIRKLILRRNPGGWVEKETYY

27) >gi|195976805|ref|NP_001124463.1| hypoxia up-regulated protein 1 precursor [Homo sapiens] (111.3 kD)

MADKVRRQRPRRRVCWALVAVLLADLLALSDTLAVMSVDLGSESMKVAIVKPGVPMEIVLNKESRRKTPVIVTLKENERFFGDSAASMAIKNPKATLRYFQHLLGKQADNPHVALYQARFPEHELTFDPQRQTVHFQISSQLQFSPEEVLGMVLNYSRSLAEDFAEQPIKDAVITVPVFFNQAERRAVLQAARMAGLKVLQLINDNTATALSYGVFRRKDINTTAQNIMFYDMGSGSTVCTIVTYQMVKTKEAGMQPQLQIRGVGFDRTLGGLEMELRLRERLAGLFNEQRKGQRAKDVRENPRAMAKLLREANRLKTVLSANADHMAQIEGLMDDVDFKAKVTRVEFEELCADLFERVPGPVQQALQSAEMSLDEIEQVILVGGATRVPRVQEVLLKAVGKEELGKNINADEAAAMGAVYQAAALSKAFKVKPFVVRDAVVYPILVEFTREVEEEPGIHSLKHNKRVLFSRMGPYPQRKVITFNRYSHDFNFHINYGDLGFLGPEDLRVFGSQNLTTVKLKGVGDSFKKYPDYESKGIKAHFNLDESGVLSLDRVESVFETLVEDSAEEESTLTKLGNTISSLFGGGTTPDAKENGTDTVQEEEESPAEGSKDEPGEQVELKEEAEAPVEDGSQPPPPEPKGDATPEGEKATEKENGDKSEAQKPSEKAEAGPEGVAPAPEGEKKQKPARKRRMVEEIGVELVVLDLPDLPEDKLAQSVQKLQDLTLRDLEKQEREKAANSLEAFIFETQDKLYQPEYQEVSTEEQREEISGKLSAASTWLEDEGVGATTVMLKEKLAELRKLCQGLFFRVEERKKWPERLSALDNLLNHSSMFLKGARLIPEMDQIFTEVEMTTLEKVINETWAWKNATLAEQAKLPATEKPVLLSKDIEAKMMALDREVQYLLNKAKFTKPRPRPKDKNGTRAEPPLNASASDQGEKVIPPAGQTEDAEPISEPEKVETGSEPGDTEPLELGGPGAEPEQKEQSTGQKRPLKNDEL

28) >gi|29029559|ref|NP_001307.2| exportin-2 isoform 1 [Homo sapiens] (110.3 kD)

MELSDANLQTLTEYLKKTLDPDPAIRRPAEKFLESVEGNQNYPLLLLTLLEKSQDNVIKVCASVTFKNYIKRNWRIVEDEPNKICEADRVAIKANIVHLMLSSPEQIQKQLSDAISIIGREDFPQKWPDLLTEMVNRFQSGDFHVINGVLRTAHSLFKRYRHEFKSNELWTEIKLVLDAFALPLTNLFKATIELCSTHANDASALRILFSSLILISKLFYSLNFQDLPEFFEDNMETWMNNFHTLLTLDNKLLQTDDEEEAGLLELLKSQICDNAALYAQKYDEEFQRYLPRFVTAIWNLLVTTGQEVKYDLLVSNAIQFLASVCERPHYKNLFEDQNTLTSICEKVIVPNMEFRAADEEAFEDNSEEYIRRDLEGSDIDTRRRAACDLVRGLCKFFEGPVTGIFSGYVNSMLQEYAKNPSVNWKHKDAAIYLVTSLASKAQTQKHGITQANELVNLTEFFVNHILPDLKSANVNEFPVLKADGIKYIMIFRNQVPKEHLLVSIPLLINHLQAESIVVHTYAAHALERLFTMRGPNNATLFTAAEIAPFVEILLTNLFKALTLPGSSENEYIMKAIMRSFSLLQEAIIPYIPTLITQLTQKLLAVSKNPSKPHFNHYMFEAICLSIRITCKANPAAVVNFEEALFLVFTEILQNDVQEFIPYVFQVMSLLLETHKNDIPSSYMALFPHLLQPVLWERTGNIPALVRLLQAFLERGSNTIASAAADKIPGLLGVFQKLIASKANDHQGFYLLNSIIEHMPPESVDQYRKQIFILLFQRLQNSKTTKFIKSFLVFINLYCIKYGALALQEIFDGIQPKMFGMVLEKIIIPEIQKVSGNVEKKICAVGITKLLTECPPMMDTEYTKLWTPLLQSLIGLFELPEDDTIPDEEHFIDIEDTPGYQTAFSQLAFAGKKEHDPVGQMVNNPKIHLAQSLHKLSTACPGRVPSMVSTSLNAEALQYLQGYLQAASVTLL

29) >gi|530418825|ref|XP_005260998.1| PREDICTED: trifunctional purine biosynthetic protein adenosine-3 isoform X1 [Homo sapiens] (107.7 kD)

MAARVLIIGSGGREHTLAWKLAQSHHVKQVLVAPGNAGTACSEKISNTAISISDHTALAQFCKEKKIEFVVVGPEAPLAAGIVGNLRSAGVQCFGPTAEAAQLESSKRFAKEFMDRHGIPTAQWKAFTKPEEACSFILSADFPALVVKASGLAAGKGVIVAKSKEEACKAVQEIMQEKAFGAAGETIVIEELLDGEEVSCLCFTDGKTVAPMPPAQDHKRLLEGDGGPNTGGMGAYCPAPQVSNDLLLKIKDTVLQRTVDGMQQEGTPYTGILYAGIMLTKNGPKVLEFNCRFGDPECQVILPLLKSDLYEVIQSTLDGLLCTSLPVWLENHTALTVVMASKGYPGDYTKGVEITGFPEAQALGLEVFHAGTALKNGKVVTHGGRVLAVTAIRENLISALEEAKKGLAAIKFEGAIYRKDVGFRAIAFLQQPRSLTYKESGVDIAAGNMLVKKIQPLAKATSRSGCKVDLGGFAGLFDLKAAGFKDPLLASGTDGVGTKLKIAQLCNKHDTIGQDLVAMCVNDILAQGAEPLFFLDYFSCGKLDLSVTEAVVAGIAKACGKAGCALLGGETAEMPDMYPPGEYDLAGFAVGAMERDQKLPHLERITEGDVVVGIASSGLHSNGFSLVRKIVAKSSLQYSSPAPDGCGDQTLGDLLLTPTRIYSHSLLPVLRSGHVKAFAHITGGGLLENIPRVLPEKLGVDLDAQTWRIPRVFSWLQQEGHLSEEEMARTFNCGVGAVLVVSKEQTEQILRDIQQHKEEAWVIGSVVARAEGSPRVKVKNLIESMQINGSVLKNGSLTNHFSFEKKKARVAVLISGTGSNLQALIDSTREPNSSAQIDIVISNKAAVAGLDKAERAGIPTRVINHKLYKNRVEFDSAIDLVLEEFSIDIVCLAGFMRILSGPFVQKWNGKMLNIHPSLLPSFKGSNAHEQALETGVTVTGCTVHFVAEDVDAGQIILQEAVPVKRGDTVATLSERVKLAEHKIFPAALQLVASGTVQLGENGKICWVKEE

30) >gi|221316630|ref|NP_001137533.1| coatomer subunit beta [Homo sapiens] (107.1 kD)

MTAAENVCYTLINVPMDSEPPSEISLKNDLEKGDVKSKTEALKKVIIMILNGEKLPGLLMTIIRFVLPLQDHTIKKLLLVFWEIVPKTTPDGRLLHEMILVCDAYRKDLQHPNEFIRGSTLRFLCKLKEAELLEPLMPAIRACLEHRHSYVRRNAVLAIYTIYRNFEHLIPDAPELIHDFLVNEKDASCKRNAFMMLIHADQDRALDYLSTCIDQVQTFGDILQLVIVELIYKVCHANPSERARFIRCIYNLLQSSSPAVKYEAAGTLVTLSSAPTAIKAAAQCYIDLIIKESDNNVKLIVLDRLIELKEHPAHERVLQDLVMDILRVLSTPDLEVRKKTLQLALDLVSSRNVEELVIVLKKEVIKTNNVSEHEDTDKYRQLLVRTLHSCSVRFPDMAANVIPVLMEFLSDNNEAAAADVLEFVREAIQRFDNLRMLIVEKMLEVFHAIKSVKIYRGALWILGEYCSTKEDIQSVMTEIRRSLGEIPIVESEIKKEAGELKPEEEITVGPVQKLVTEMGTYATQSALSSSRPTKKEEDRPPLRGFLLDGDFFVAASLATTLTKIALRYVALVQEKKKQNSFVAEAMLLMATILHLGKSSLPKKPITDDDVDRISLCLKVLSECSPLMNDIFNKECRQSLSHMLSAKLEEEKLSQKKESEKRNVTVQPDDPISFMQLTAKNEMNCKEDQFQLSLLAAMGNTQRKEAADPLASKLNKVTQLTGFSDPVYAEAYVHVNQYDIVLDVLVVNQTSDTLQNCTLELATLGDLKLVEKPSPLTLAPHDFANIKANVKVASTENGIIFGNIVYDVSGAASDRNCVVLSDIHIDIMDYIQPATCTDAEFRQMWAEFEWENKVTVNTNMVDLNDYLQHILKSTNMKCLTPEKALSGYCGFMAANLYARSIFGEDALANVSIEKPIHQGPDAAVTGHIRIRAKSQGMALSLGDKINLSQKKTSI

31) >gi|38202257|ref|NP_938148.1| neutral alpha-glucosidase AB isoform 2 precursor [Homo sapiens] (106.8 kD)

MAAVAAVAARRRRSWASLVLAFLGVCLGITLAVDRSNFKTCEESSFCKRQRSIRPGLSPYRALLDSLQLGPDSLTVHLIHEVTKVLLVLELQGLQKNMTRFRIDELEPRRPRYRVPDVLVADPPIARLSVSGRDENSVELTMAEGPYKIILTARPFRLDLLEDRSLLLSVNARGLLEFEHQRAPRVSQGSKDPAEGDGAQPEETPRDGDKPEETQGKAEKDEPGAWEETFKTHSDSKPYGPMSVGLDFSLPGMEHVYGIPEHADNLRLKVTEGGEPYRLYNLDVFQYELYNPMALYGSVPVLLAHNPHRDLGIFWLNAAETWVDISSNTAGKTLFGKMMDYLQGSGETPQTDVRWMSETGIIDVFLLLGPSISDVFRQYASLTGTQALPPLFSLGYHQSRWNYRDEADVLEVDQGFDDHNLPCDVIWLDIEHADGKRYFTWDPSRFPQPRTMLERLASKRRKLVAIVDPHIKVDSGYRVHEELRNLGLYVKTRDGSDYEGWCWPGSAGYPDFTNPTMRAWWANMFSYDNYEGSAPNLFVWNDMNEPSVFNGPEVTMLKDAQHYGGWEHRDVHNIYGLYVHMATADGLRQRSGGMERPFVLARAFFAGSQRFGAVWTGDNTAEWDHLKISIPMCLSLGLVGLSFCGADVGGFFKNPEPELLVRWYQMGAYQPFFRAHAHLDTGRREPWLLPSQHNDIIRDALGQRYSLLPFWYTLLYQAHREGIPVMRPLWVQYPQDVTTFNIDDQYLLGDALLVHPVSDSGAHGVQVYLPGQGEVWYDIQSYQKHHGPQTLYLPVTLSSIPVFQRGGTIVPRWMRVRRSSECMKDDPITLFVALSPQGTAQGELFLDDGHTFNYQTRQEFLLRRFSFSGNTLVSSSADPEGHFETPIWIERVVIIGAGKPAAVVLQTKGSPESRLSFQHDPETSVLVLRKPGINVASDWSIHLR

32) >gi|578834952|ref|XP_006723469.1| PREDICTED: alpha-actinin-4 isoform X3 [Homo sapiens] (104.8 kD)

MVDYHAANQSYQYGPSSAGNGAGGGGSMGDYMAQEDDWDRDLLLDPAWEKQQRKTFTAWCNSHLRKAGTQIENIDEDFRDGLKLMLLLEVISGERLPKPERGKMRVHKINNVNKALDFIASKGVKLVSIGAEEIVDGNAKMTLGMIWTIILRFAIQDISVEETSAKEGLLLWCQRKTAPYKNVNVQNFHISWKDGLAFNALIHRHRPELIEYDKLRKDDPVTNLNNAFEVAEKYLDIPKMLDAEDIVGTLRPDEKAIMTYVSCFYHAFSGAQKAETAANRICKVLAVNQENEHLMEDYEKLASDLLEWIRRTIPWLEDRVPQKTIQEMQQKLEDFRDYRRVHKPPKVQEKCQLEINFNTLQTKLRLSNRPAFMPSEGKMVSDINNGWQHLEQAEKGYEEWLLNEIRRLERLDHLAEKFRQKASIHEAWTDGKEAMLKHRDYETATLSDIKALIRKHEAFESDLAAHQDRVEQIAAIAQELNELDYYDSHNVNTRCQKICDQWDALGSLTHSRREALEKTEKQLEAIDQLHLEYAKRAAPFNNWMESAMEDLQDMFIVHTIEEIEGLISAHDQFKSTLPDADREREAILAIHKEAQRIAESNHIKLSGSNPYTTVTPQIINSKWEKVQQLVPKRDHALLEEQSKQQSNEHLRRQFASQANVVGPWIQTKMEEIGRISIEMNGTLEDQLSHLKQYERSIVDYKPNLDLLEQQHQLIQEALIFDNKHTNYTMEHIRVGWEQLLTTIARTINEVENQILTRDAKGISQEQMQEFRASFNHFDKDHGGALGPEEFKACLISLGYDVENDRQGEAEFNRIMSLVDPNHSGLVTFQAFIDFMSRETTDTDTADQVIASFKVLAGDKNFITAEELRRELPPDQAEYCIARMAPYQGPDAVPGALDYKSFSTALYGESDL

33) >gi|4557469|ref|NP_001273.1| AP-2 complex subunit beta isoform b [Homo sapiens] (104.5 kD)

MTDSKYFTTNKKGEIFELKAELNNEKKEKRKEAVKKVIAAMTVGKDVSSLFPDVVNCMQTDNLELKKLVYLYLMNYAKSQPDMAIMAVNSFVKDCEDPNPLIRALAVRTMGCIRVDKITEYLCEPLRKCLKDEDPYVRKTAAVCVAKLHDINAQMVEDQGFLDSLRDLIADSNPMVVANAVAALSEISESHPNSNLLDLNPQNINKLLTALNECTEWGQIFILDCLSNYNPKDDREAQSICERVTPRLSHANSAVVLSAVKVLMKFLELLPKDSDYYNMLLKKLAPPLVTLLSGEPEVQYVALRNINLIVQKRPEILKQEIKVFFVKYNDPIYVKLEKLDIMIRLASQANIAQVLAELKEYATEVDVDFVRKAVRAIGRCAIKVEQSAERCVSTLLDLIQTKVNYVVQEAIVVIRDIFRKYPNKYESIIATLCENLDSLDEPDARAAMIWIVGEYAERIDNADELLESFLEGFHDESTQVQLTLLTAIVKLFLKKPSETQELVQQVLSLATQDSDNPDLRDRGYIYWRLLSTDPVTAKEVVLSEKPLISEETDLIEPTLLDELICHIGSLASVYHKPPNAFVEGSHGIHRKHLPIHHGSTDAGDSPVGTTTATNLEQPQVIPSQGDLLGDLLNLDLGPPVNVPQVSSMQMGAVDLLGGGLDSLVGQSFIPSSVPATFAPSPTPAVVSSGLNDLFELSTGIGMAPGGYVAPKAVWLPAVKAKGLEISGTFTHRQGHIYMEMNFTNKALQHMTDFAIQFNKNSFGVIPSTPLAIHTPLMPNQSIDVSLPLNTLGPVMKMEPLNNLQVAVKNNIDVFYFSCLIPLNVLFVEDGKMERQVFLATWKDIPNENELQFQIKECHLNADTVSSKLQNNNVYTIAKRNVEGQDMLYQSLKLTNGIWILAELRIQPGNPNYTLSLKCRAPEVSQYIYQVYDSILKN

34) >gi|27477041|ref|NP_036437.1| AP-2 complex subunit alpha-2 isoform 2 [Homo sapiens] (103.9 kD)

MPAVSKGDGMRGLAVFISDIRNCKSKEAEIKRINKELANIRSKFKGDKALDGYSKKKYVCKLLFIFLLGHDIDFGHMEAVNLLSSNRYTEKQIGYLFISVLVNSNSELIRLINNAIKNDLASRNPTFMGLALHCIASVGSREMAEAFAGEIPKVLVAGDTMDSVKQSAALCLLRLYRTSPDLVPMGDWTSRVVHLLNDQHLGVVTAATSLITTLAQKNPEEFKTSVSLAVSRLSRIVTSASTDLQDYTYYFVPAPWLSVKLLRLLQCYPPPDPAVRGRLTECLETILNKAQEPPKSKKVQHSNAKNAVLFEAISLIIHHDSEPNLLVRACNQLGQFLQHRETNLRYLALESMCTLASSEFSHEAVKTHIETVINALKTERDVSVRQRAVDLLYAMCDRSNAPQIVAEMLSYLETADYSIREEIVLKVAILAEKYAVDYTWYVDTILNLIRIAGDYVSEEVWYRVIQIVINRDDVQGYAAKTVFEALQAPACHENLVKVGGYILGEFGNLIAGDPRSSPLIQFHLLHSKFHLCSVPTRALLLSTYIKFVNLFPEVKPTIQDVLRSDSQLRNADVELQQRAVEYLRLSTVASTDILATVLEEMPPFPERESSILAKLKKKKGPSTVTDLEDTKRDRSVDVNGGPEPAPASTSAVSTPSPSADLLGLGAAPPAPAGPPPSSGGSGLLVDVFSDSASVVAPLAPGSEDNFARFVCKNNGVLFENQLLQIGLKSEFRQNLGRMFIFYGNKTSTQFLNFTPTLICSDDLQPNLNLQTKPVDPTVEGGAQVQQVVNIECVSDFTEAPVLNIQFRYGGTFQNVSVQLPITLNKFFQPTEMASQDFFQRWKQLSNPQQEVQNIFKAKHPMDTEVTKAKIIGFGSALLEEVDPNPANFVGAGIIHTKTTQIGCLLRLEPNLQAQMYRLTLRTSKEAVSQRLCELLSAQF

35) >gi|4501891|ref|NP_001093.1| alpha-actinin-1 isoform b [Homo sapiens] (103 kD)

MDHYDSQQTNDYMQPEEDWDRDLLLDPAWEKQQRKTFTAWCNSHLRKAGTQIENIEEDFRDGLKLMLLLEVISGERLAKPERGKMRVHKISNVNKALDFIASKGVKLVSIGAEEIVDGNVKMTLGMIWTIILRFAIQDISVEETSAKEGLLLWCQRKTAPYKNVNIQNFHISWKDGLGFCALIHRHRPELIDYGKLRKDDPLTNLNTAFDVAEKYLDIPKMLDAEDIVGTARPDEKAIMTYVSSFYHAFSGAQKAETAANRICKVLAVNQENEQLMEDYEKLASDLLEWIRRTIPWLENRVPENTMHAMQQKLEDFRDYRRLHKPPKVQEKCQLEINFNTLQTKLRLSNRPAFMPSEGRMVSDINNAWGCLEQVEKGYEEWLLNEIRRLERLDHLAEKFRQKASIHEAWTDGKEAMLRQKDYETATLSEIKALLKKHEAFESDLAAHQDRVEQIAAIAQELNELDYYDSPSVNARCQKICDQWDNLGALTQKRREALERTEKLLETIDQLYLEYAKRAAPFNNWMEGAMEDLQDTFIVHTIEEIQGLTTAHEQFKATLPDADKERLAILGIHNEVSKIVQTYHVNMAGTNPYTTITPQEINGKWDHVRQLVPRRDQALTEEHARQQHNERLRKQFGAQANVIGPWIQTKMEEIGRISIEMHGTLEDQLSHLRQYEKSIVNYKPKIDQLEGDHQLIQEALIFDNKHTNYTMEHIRVGWEQLLTTIARTINEVENQILTRDAKGISQEQMNEFRASFNHFDRDHSGTLGPEEFKACLISLGYDIGNDPQGEAEFARIMSIVDPNRLGVVTFQAFIDFMSRETADTDTADQVMASFKILAGDKNYITMDELRRELPPDQAEYCIARMAPYTGPDSVPGALDYMSFSTALYGESDL

36) >gi|188497754|ref|NP_000179.2| hexokinase-1 isoform HKI [Homo sapiens] (102.4 kD)

MIAAQLLAYYFTELKDDQVKKIDKYLYAMRLSDETLIDIMTRFRKEMKNGLSRDFNPTATVKMLPTFVRSIPDGSEKGDFIALDLGGSSFRILRVQVNHEKNQNVHMESEVYDTPENIVHGSGSQLFDHVAECLGDFMEKRKIKDKKLPVGFTFSFPCQQSKIDEAILITWTKRFKASGVEGADVVKLLNKAIKKRGDYDANIVAVVNDTVGTMMTCGYDDQHCEVGLIIGTGTNACYMEELRHIDLVEGDEGRMCINTEWGAFGDDGSLEDIRTEFDREIDRGSLNPGKQLFEKMVSGMYLGELVRLILVKMAKEGLLFEGRITPELLTRGKFNTSDVSAIEKNKEGLHNAKEILTRLGVEPSDDDCVSVQHVCTIVSFRSANLVAATLGAILNRLRDNKGTPRLRTTVGVDGSLYKTHPQYSRRFHKTLRRLVPDSDVRFLLSESGSGKGAAMVTAVAYRLAEQHRQIEETLAHFHLTKDMLLEVKKRMRAEMELGLRKQTHNNAVVKMLPSFVRRTPDGTENGDFLALDLGGTNFRVLLVKIRSGKKRTVEMHNKIYAIPIEIMQGTGEELFDHIVSCISDFLDYMGIKGPRMPLGFTFSFPCQQTSLDAGILITWTKGFKATDCVGHDVVTLLRDAIKRREEFDLDVVAVVNDTVGTMMTCAYEEPTCEVGLIVGTGSNACYMEEMKNVEMVEGDQGQMCINMEWGAFGDNGCLDDIRTHYDRLVDEYSLNAGKQRYEKMISGMYLGEIVRNILIDFTKKGFLFRGQISETLKTRGIFETKFLSQIESDRLALLQVRAILQQLGLNSTCDDSILVKTVCGVVSRRAAQLCGAGMAAVVDKIRENRGLDRLNVTVGVDGTLYKLHPHFSRIMHQTVKELSPKCNVSFLLSEDGSGKGAALITAVGVRLRTEASS

37) >gi|19923236|ref|NP_003104.2| nuclear autoantigen Sp-100 isoform 2 [Homo sapiens] (100.4 kD)

MAGGGGDLSTRRLNECISPVANEMNHLPAHSHDLQRMFTEDQGVDDRLLYDIVFKHFKRNKVEISNAIKKTFPFLEGLRDRDLITNKMFEDSQDSCRNLVPVQRVVYNVLSELEKTFNLPVLEALFSDVNMQEYPDLIHIYKGFENVIHDKLPLQESEEEEREERSGLQLSLEQGTGENSFRSLTWPPSGSPSHAGTTPPENGLSEHPCETEQINAKRKDTTSDKDDSLGSQQTNEQCAQKAEPTESCEQIAVQVNNGDAGREMPCPLPCDEESPEAELHNHGIQINSCSVRLVDIKKEKPFSNSKVECQAQARTHHNQASDIIVISSEDSEGSTDVDEPLEVFISAPRSEPVINNDNPLESNDEKEGQEATCSRPQIVPEPMDFRKLSTFRESFKKRVIGQDHDFSESSEEEAPAEASSGALRSKHGEKAPMTSRSTSTWRIPSRKRRFSSSDFSDLSNGEELQETCSSSLRRGSGSQPQEPENKKCSCVMCFPKGVPRSQEARTESSQASDMMDTMDVENNSTLEKHSGKRRKKRRHRSKVNGLQRGRKKDRPRKHLTLNNKVQKKRWQQRGRKANTRPLKRRRKRGPRIPKDENINFKQSELPVTCGEVKGTLYKERFKQGTSKKCIQSEDKKWFTPREFEIEGDRGASKNWKLSIRCGGYTLKVLMENKFLPEPPSTRKKRILESHNNTLVDPCEEHKKKNPDASVKFSEFLKKCSETWKTIFAKEKGKFEDMAKADKAHYEREMKTYIPPKGEKKKKFKDPNAPKRPPLAFFLFCSEYRPKIKGEHPGLSIDDVVKKLAGMWNNTAAADKQFYEKKAAKLKEKYKKDIAAYRAKGKPNSAKKRVVKAEKSKKKKEEEEDEEDEQEEENEEDDDK

38) >gi|51094101|ref|NP_076977.3| ATP-dependent RNA helicase DDX54 isoform 2 [Homo sapiens] (98.5 kD)

MAADKGPAAGPRSRAAMAQWRKKKGLRKRRGAASQARGSDSEDGEFEIQAEDDARARKLGPGRPLPTFPTSECTSDVEPDTREMVRAQNKKKKKSGGFQSMGLSYPVFKGIMKKGYKVPTPIQRKTIPVILDGKDVVAMARTGSGKTACFLLPMFERLKTHSAQTGARALILSPTRELALQTLKFTKELGKFTGLKTALILGGDRMEDQFAALHENPDIIIATPGRLVHVAVEMSLKLQSVEYVVFDEADRLFEMGFAEQLQEIIARLPGGHQTVLFSATLPKLLVEFARAGLTEPVLIRLDVDTKLNEQLKTSFFLVREDTKAAVLLHLLHNVVRPQDQTVVFVATKHHAEYLTELLTTQRVSCAHIYSALDPTARKINLAKFTLGKCSTLIVTDLAARGLDIPLLDNVINYSFPAKGKLFLHRVGRVARAGRSGTAYSLVAPDEIPYLLDLHLFLGRSLTLARPLKEPSGVAGVDGMLGRVPQSVVDEEDSGLQSTLEASLELRGLARVADNAQQQYVRSRPAPSPESIKRAKEMDLVGLGLHPLFSSRFEEEELQRLRLVDSIKNYRSRATIFEINASSRDLCSQVMRAKRQKDRKAIARFQQGQQGRQEQQEGPVGPAPSRPALQEKQPEKEEEEEAGESVEDIFSEVVGRKRQRSGPNRGAKRRREEARQRDQEFYIPYRPKDFDSERGLSISGEGGAFEQQAAGAVLDLMGDEAQNLTRGRQQLKWDRKKKRFVGQSGQEDKKKIKTESGRYISSSYKRDLYQKWKQKQKIDDRDSDEEGASDRRGPERRGGKRDRGQGASRPHAPGTPAGRVRPELKTKQQILKQRRRAQKLHFLQRGGLKQLSARNRRRVQELQQGAFGRGARSKKGKMRKRM

39) >gi|21536466|ref|NP_068713.2| tyrosine-protein kinase receptor UFO isoform 1 precursor [Homo sapiens] (98.3 kD)

MAWRCPRMGRVPLAWCLALCGWACMAPRGTQAEESPFVGNPGNITGARGLTGTLRCQLQVQGEPPEVHWLRDGQILELADSTQTQVPLGEDEQDDWIVVSQLRITSLQLSDTGQYQCLVFLGHQTFVSQPGYVGLEGLPYFLEEPEDRTVAANTPFNLSCQAQGPPEPVDLLWLQDAVPLATAPGHGPQRSLHVPGLNKTSSFSCEAHNAKGVTTSRTATITVLPQQPRNLHLVSRQPTELEVAWTPGLSGIYPLTHCTLQAVLSDDGMGIQAGEPDPPEEPLTSQASVPPHQLRLGSLHPHTPYHIRVACTSSQGPSSWTHWLPVETPEGVPLGPPENISATRNGSQAFVHWQEPRAPLQGTLLGYRLAYQGQDTPEVLMDIGLRQEVTLELQGDGSVSNLTVCVAAYTAAGDGPWSLPVPLEAWRPGQAQPVHQLVKEPSTPAFSWPWWYVLLGAVVAAACVLILALFLVHRRKKETRYGEVFEPTVERGELVVRYRVRKSYSRRTTEATLNSLGISEELKEKLRDVMVDRHKVALGKTLGEGEFGAVMEGQLNQDDSILKVAVKTMKIAICTRSELEDFLSEAVCMKEFDHPNVMRLIGVCFQGSERESFPAPVVILPFMKHGDLHSFLLYSRLGDQPVYLPTQMLVKFMADIASGMEYLSTKRFIHRDLAARNCMLNENMSVCVADFGLSKKIYNGDYYRQGRIAKMPVKWIAIESLADRVYTSKSDVWSFGVTMWEIATRGQTPYPGVENSEIYDYLRQGNRLKQPADCLDGLYALMSRCWELNPQDRPSFTELREDLENTLKALPPAQEPDEILYVNMDEGGGYPEPPGAAGGADPPTQPDPKDSCSCLTAAEVHPAGRYVLCPSTTPSPAQPADRGSPAAPGQEDGA

40) >gi|109134349|ref|NP_036265.3| coatomer subunit gamma-2 isoform 1 [Homo sapiens] (97.6 kD)

MIKKFDKKDEESGSGSNPFQHLEKSAVLQEARIFNETPINPRRCLHILTKILYLLNQGEHFGTTEATEAFFAMTRLFQSNDQTLRRMCYLTIKEMATISEDVIIVTSSLTKDMTGKEDVYRGPAIRALCRITDGTMLQAIERYMKQAIVDKVSSVSSSALVSSLHMMKISYDVVKRWINEAQEAASSDNIMVQYHALGVLYHLRKNDRLAVSKMLNKFTKSGLKSQFAYCMLIRIASRLLKETEDGHESPLFDFIESCLRNKHEMVIYEAASAIIHLPNCTARELAPAVSVLQLFCSSPKPALRYAAVRTLNKVAMKHPSAVTACNLDLENLITDSNRSIATLAITTLLKTGSESSVDRLMKQISSFVSEISDEFKVVVVQAISALCQKYPRKHSVMMTFLSNMLRDDGGFEYKRAIVDCIISIVEENPESKEAGLAHLCEFIEDCEHTVLATKILHLLGKEGPRTPVPSKYIRFIFNRVVLENEAVRAAAVSALAKFGAQNESLLPSILVLLQRCMMDTDDEVRDRATFYLNVLQQRQMALNATYIFNGLTVSVPGMEKALHQYTLEPSEKPFDMKSIPLAMAPVFEQKAEITLVATKPEKLAPSRQDIFQEQLAAIPEFLNIGPLFKSSEPVQLTEAETEYFVRCIKHMFTNHIVFQFDCTNTLNDQLLEKVTVQMEPSDSYEVLSCIPAPSLPYNQPGICYTLVRLPDDDPTAVAGSFSCTMKFTVRDCDPNTGVPDEDGYDDEYVLEDLEVTVSDHIQKVLKPNFAAAWEEVGDTFEKEETFALSSTKTLEEAVNNIITFLGMQPCERSDKVPENKNSHSLYLAGIFRGGYDLLVRSRLALADGVTMQVTVRSKERTPVDVILASVG

**Figure legend**: These are 40 largest proteins identified from the 72-kD stripe (MB231). The red underlined sequences are the LC-MS/MS identified peptide fragments that are unique to the protein, while the green underlined sequences are the LC-MS/MS identified peptide fragments that are not unique to the protein but can also appear in other proteins.

**The 20 smallest proteins identified in the 48-kD stripe (MCF7)**

1) >gi|4504301|ref|NP_003529.1| histone H4 [Homo sapiens] (11.4 kD)

MSGRGKGGKGLGKGGAKRHRKVLRDNIQGITKPAIRRLARRGGVKRISGLIYEETRGVLKVFLENVIRDAVTYTEHAKRKTVTAMDVVYALKRQGRTLYGFGG

2) >gi|18105048|ref|NP_542160.1| histone H2B type 1-K [Homo sapiens] (13.9 kD)

MPEPAKSAPAPKKGSKKAVTKAQKKDGKKRKRSRKESYSVYVYKVLKQVHPDTGISSKAMGIMNSFVNDIFERIAGEASRLAHYNKRSTITSREIQTAVRLLLPGELAKHAVSEGTKAVTKYTSAK

3) >gi|150170694|ref|NP_997329.2| membrane protein FAM174B precursor [Homo sapiens] (16.8 kD)

MRAVPLPAPLLPLLLLALLAAPAARASRAESVSAPWPEPERESRPPPGPGPGNTTRFGSGAAGGSGSSSSNSSGDALVTRISILLRDLPTLKAAVIVAFAFTTLLIACLLLRVFRSGKRLKKTRKYDIITTPAERVEMAPLNEEDDEDEDSTVFDIKYR

4) >gi|5803141|ref|NP_006858.1| RNA-binding protein with multiple splicing isoform A [Homo sapiens] (21.8 kD)

MNNGGKAEKENTPSEANLQEEEVRTLFVSGLPLDIKPRELYLLFRPFKGYEGSLIKLTSKQPVGFVSFDSRSEAEAAKNALNGIRFDPEIPQTLRLEFAKANTKMAKNKLVGTPNPSTPLPNTVPQFIAREPYELTVPALYPSSPEVWAPYPLYPAELAPALPPPAFTYPASLHAQMRWLPPSEATSQGWKSRQFC

5) >gi|320461711|ref|NP_001189360.1| peroxiredoxin-1 [Homo sapiens] (22.1 kD)

MSSGNAKIGHPAPNFKATAVMPDGQFKDISLSDYKGKYVVFFFYPLDFTFVCPTEIIAFSDRAEEFKKLNCQVIGASVDSHFCHLAWVNTPKKQGGLGPMNIPLVSDPKRTIAQDYGVLKADEGISFRGLFIIDDKGILRQITVNDLPVGRSVDETLRLVQAFQFTDKHGEVCPAGWKPGSDTIKPDVQKSKEYFSKQK

6) >gi|256222019|ref|NP_057215.3| ras-related protein Rab-10 [Homo sapiens] (22.5 kD)

MAKKTYDLLFKLLLIGDSGVGKTCVLFRFSDDAFNTTFISTIGIDFKIKTVELQGKKIKLQIWDTAGQERFHTITTSYYRGAMGIMLVYDITNGKSFENISKWLRNIDEHANEDVERMLLGNKCDMDDKRVVPKGKGEQIAREHGIRFFETSAKANINIEKAFLTLAEDILRKTPVKEPNSENVDISSGGGVTGWKSKCC

7) >gi|409971425|ref|NP_001258535.1| brain acid soluble protein 1 [Homo sapiens] (22.7 kD)

MGGKLSKKKKGYNVNDEKAKEKDKKAEGAATEEEGTPKESEPQAAAEPAEAKEGKEKPDQDAEGKAEEKEGEKDAAAAKEEAPKAEPEKTEGAAEAKAEPPKAPEQEQAAPGPAAGGEAPKAAEAAAAPAESAAPAAGEEPSKEEGEPKKTEAPAAPAAQETKSDGAPASDSKPGSSEAAPSSKETPAATEAPSSTPKAQGPAASAEEPKPVEAPAANSDQTVTVKE

8) >gi|358356396|ref|NP_001240308.1| 60S ribosomal protein L15 isoform 1 [Homo sapiens] (24.1 kD)

MGAYKYIQELWRKKQSDVMRFLLRVRCWQYRQLSALHRAPRPTRPDKARRLGYKAKQGYVIYRIRVRRGGRKRPVPKGATYGKPVHHGVNQLKFARSLQSVAEERAGRHCGALRVLNSYWVGEDSTYKFFEVILIDPFHKAIRRNPDTQWITKPVHKHREMRGLTSAGRKSRGLGKGHKFHHTIGGSRRAAWRRRNTLQLHRYR

9) >gi|383872447|ref|NP_001244318.1| CD63 antigen isoform A [Homo sapiens] (25.6 kD)

MAVEGGMKCVKFLLYVLLLAFCACAVGLIAVGVGAQLVLSQTIIQGATPGSLLPVVIIAVGVFLFLVAFVGCCGACKENYCLMITFAIFLSLIMLVEVAAAIAGYVFRDKVMSEFNNNFRQQMENYPKNNHTASILDRMQADFKCCGAANYTDWEKIPSMSKNRVPDSCCINVTVGCGINFNEKAIHKEGCVEKIGGWLRKNVLVVAAAALGIAFVEVLGIVFACCLVKSIRSGYEVM

10) >gi|378548190|ref|NP_001243731.1| 40S ribosomal protein S3 isoform 1 [Homo sapiens] (26.7 kD)

MAVQISKKRKFVADGIFKAELNEFLTRELAEDGYSGVEVRVTPTRTEIIILATRTQNVLGEKGRRIRELTAVVQKRFGFPEGSVELYAEKVATRGLCAIAQAESLRYKLLGGLAVRRACYGVLRFIMESGAKGCEVVVSGKLRGQRAKSMKFVDGLMIHSGDPVNYYVDTAVRHVLLRQGVLGIKVKIMLPWDPTGKIGPKKPLPDHVSIVEPKDEILPTTPISEQKGGKPEPPAMPQPVPTA

11) >gi|4507953|ref|NP_003397.1| 14-3-3 protein zeta/delta [Homo sapiens] (27.7 kD)

MDKNELVQKAKLAEQAERYDDMAACMKSVTEQGAELSNEERNLLSVAYKNVVGARRSSWRVVSSIEQKTEGAEKKQQMAREYREKIETELRDICNDVLSLLEKFLIPNASQAESKVFYLKMKGDYYRYLAEVAAGDDKKGIVDQSQQAYQEAFEISKKEMQPTHPIRLGLALNFSVFYYEILNSPEKACSLAKTAFDEAIAELDTLSEESYKDSTLIMQLLRDNLTLWTSDTQGDEAEAGEGGEN

12) >gi|4502677|ref|NP_001770.1| lymphocyte function-associated antigen 3 isoform 1 [Homo sapiens] (28.1 kD)

MVAGSDAGRALGVLSVVCLLHCFGFISCFSQQIYGVVYGNVTFHVPSNVPLKEVLWKKQKDKVAELENSEFRAFSSFKNRVYLDTVSGSLTIYNLTSSDEDEYEMESPNITDTMKFFLYVLESLPSPTLTCALTNGSIEVQCMIPEHYNSHRGLIMYSWDCPMEQCKRNSTSIYFKMENDLPQKIQCTLSNPLFNTTSSIILTTCIPSSGHSRHRYALIPIPLAVITTCIVLYMNGILKCDRKPDRTNSN

13) >gi|5803185|ref|NP_006745.1| synaptophysin-like protein 1 isoform a [Homo sapiens] (28.5 kD)

MAPNIYLVRQRISRLGQRMSGFQINLNPLKEPLGFIKVLEWIASIFAFATCGGFKGQTEIQVNCPPAVTENKTVTATFGYPFRLNEASFQPPPGVNICDVNWKDYVLIGDYSSSAQFYVTFAVFVFLYCIAALLLYVGYTSLYLDSRKLPMIDFVVTLVATFLWLVSTSAWAKALTDIKIATGHNIIDELPPCKKKAVLCYFGSVTSMGSLNVSVIFGFLNMILWGGNAWFVYKETSLHSPSNTSAPHSQGGIPPPTGI

14) >gi|5803225|ref|NP_006752.1| 14-3-3 protein epsilon [Homo sapiens] (29.2 kD)

MDDREDLVYQAKLAEQAERYDEMVESMKKVAGMDVELTVEERNLLSVAYKNVIGARRASWRIISSIEQKEENKGGEDKLKMIREYRQMVETELKLICCDILDVLDKHLIPAANTGESKVFYYKMKGDYHRYLAEFATGNDRKEAAENSLVAYKAASDIAMTELPPTHPIRLGLALNFSVFYYEILNSPDRACRLAKAAFDDAIAELDTLSEESYKDSTLIMQLLRDNLTLWTSDMQGDGEEQNKEALQDVEDENQ

15) >gi|4507879|ref|NP_003365.1| voltage-dependent anion-selective channel protein 1 [Homo sapiens] (30.8 kD)

MAVPPTYADLGKSARDVFTKGYGFGLIKLDLKTKSENGLEFTSSGSANTETTKVTGSLETKYRWTEYGLTFTEKWNTDNTLGTEITVEDQLARGLKLTFDSSFSPNTGKKNAKIKTGYKREHINLGCDMDFDIAGPSIRGALVLGYEGWLAGYQMNFETAKSRVTQSNFAVGYKTDEFQLHTNVNDGTEFGGSIYQKVNKKLETAVNLAWTAGNSNTRFGIAAKYQIDPDACFSAKVNNSSLIGLGYTQTLKPGIKLTLSALLDGKNVNAGGHKLGLGLEFQA

16) >gi|25453472|ref|NP_001951.2| elongation factor 1-delta isoform 2 [Homo sapiens] (31.1 kD)

MATNFLAHEKIWFDKFKYDDAERRFYEQMNGPVAGASRQENGASVILRDIARARENIQKSLAGSSGPGASSGTSGDHGELVVRIASLEVENQSLRGVVQELQQAISKLEARLNVLEKSSPGHRATAPQTQHVSPMRQVEPPAKKPATPAEDDEDDDIDLFGSDNEEEDKEAAQLREERLRQYAEKKAKKPALVAKSSILLDVKPWDDETDMAQLEACVRSIQLDGLVWGASKLVPVGYGIRKLQIQCVVEDDKVGTDLLEEEITKFEEHVQSVDIAAFNKI

17) >gi|296317339|ref|NP_001171752.1| voltage-dependent anion-selective channel protein 2 isoform 2 [Homo sapiens] (31.5 kD)

MATHGQTCARPMCIPPSYADLGKAARDIFNKGFGFGLVKLDVKTKSCSGVEFSTSGSSNTDTGKVTGTLETKYKWCEYGLTFTEKWNTDNTLGTEIAIEDQICQGLKLTFDTTFSPNTGKKSGKIKSSYKRECINLGCDVDFDFAGPAIHGSAVFGYEGWLAGYQMTFDSAKSKLTRNNFAVGYRTGDFQLHTNVNDGTEFGGSIYQKVCEDLDTSVNLAWTSGTNCTRFGIAAKYQLDPTASISAKVNNSSLIGVGYTQTLRPGVKLTLSALVDGKSINAGGHKVGLALELEA

18) >gi|61966711|ref|NP_001013653.1| heterogeneous nuclear ribonucleoprotein C-like 1 [Homo sapiens] (32.1 kD)

MASNVTNKMDPHSMNSRVFIGNLNTLVVKKSDVEAIFSKYGKIAGCSVHKGFAFVQYDKEKNARAAVAGEDGRMIASQVVDINLAAEPKVNRGNAGVKRSAAEMYGSSFDLDYGFQRDYYDGMYSFPARVPPPPPIALAVVPSKRQRLSGNTSRRGKSGFNSKSGKRGSSKSGKLKGDDLQAIKQELTQIKQKVDSLLENLEKIEKEQSKQEVEVKNAKSEEEQSSSSMKKDETHVKMESEGGAEDSAEEGDPLDDDVNEDQGDDQLELIKDDEKEAEEGEDDRDSTNGQDDS

19) >gi|10835063|ref|NP_002511.1| nucleophosmin isoform 1 [Homo sapiens] (32.6 kD)

MEDSMDMDMSPLRPQNYLFGCELKADKDYHFKVDNDENEHQLSLRTVSLGAGAKDELHIVEAEAMNYEGSPIKVTLATLKMSVQPTVSLGGFEITPPVVLRLKCGSGPVHISGQHLVAVEEDAESEDEEEEDVKLLSISGKRSAPGGGSKVPQKKVKLAADEDDDDDDEEDDDEDDDDDDFDDEEAEEKAPVKKSIRDTPAKNAQKSNQNGKDSKPSSTPRSKGQESFKKQEKTPKTPKGPSSVEDIKAKMQASIEKGGSLPKVEAKFINYVKNCFRMTDQEAIQDLWQWRKSL

20) >gi|9558733|ref|NP_037425.1| transformer-2 protein homolog alpha isoform 1 [Homo sapiens] (32.7 kD)

MSDVEENNFEGRESRSQSKSPTGTPARVKSESRSGSRSPSRVSKHSESHSRSRSKSRSRSRRHSHRRYTRSRSHSHSHRRRSRSRSYTPEYRRRRSRSHSPMSNRRRHTGSRANPDPNTCLGVFGLSLYTTERDLREVFSRYGPLSGVNVVYDQRTGRSRGFAFVYFERIDDSKEAMERANGMELDGRRIRVDYSITKRAHTPTPGIYMGRPTHSGGGGGGGGGGGGGGGGRRRDSYYDRGYDRGYDRYEDYDYRYRRRSPSPYYSRYRSRSRSRSYSPRRY

**Figure legend**: These are 20 smallest proteins identified in the 48-kD stripe (MCF7). The red underlined sequences are the LC-MS/MS identified peptide fragments that are unique to the protein, while the green underlined sequences are the LC-MS/MS identified peptide fragments that are not unique to the protein but can also appear in other proteins.

**The 40 largest proteins in the 48-kD stripe (MCF7)**

1) 1) >gi|61743954|ref|NP_001611.1| neuroblast differentiation-associated protein AHNAK isoform 1 [Homo sapiens] (628.7 kD)

MEKEETTRELLLPNWQGSGSHGLTIAQRDDGVFVQEVTQNSPAARTGVVKEGDQIVGATIYFDNLQSGEVTQLLNTMGHHTVGLKLHRKGDRSPEPGQTWTREVFSSCSSEVVLSGDDEEYQRIYTTKIKPRLKSEDGVEGDLGETQSRTITVTRRVTAYTVDVTGREGAKDIDISSPEFKIKIPRHELTEISNVDVETQSGKTVIRLPSGSGAASPTGSAVDIRAGAISASGPELQGAGHSKLQVTMPGIKVGGSGVNVNAKGLDLGGRGGVQVPAVDISSSLGGRAVEVQGPSLESGDHGKIKFPTMKVPKFGVSTGREGQTPKAGLRVSAPEVSVGHKGGKPGLTIQAPQLEVSVPSANIEGLEGKLKGPQITGPSLEGDLGLKGAKPQGHIGVDASAPQIGGSITGPSVEVQAPDIDVQGPGSKLNVPKMKVPKFSVSGAKGEETGIDVTLPTGEVTVPGVSGDVSLPEIATGGLEGKMKGTKVKTPEMIIQKPKISMQDVDLSLGSPKLKGDIKVSAPGVQGDVKGPQVALKGSRVDIETPNLEGTLTGPRLGSPSGKTGTCRISMSEVDLNVAAPKVKGGVDVTLPRVEGKVKVPEVDVRGPKVDVSAPDVEAHGPEWNLKMPKMKMPTFSTPGAKGEGPDVHMTLPKGDISISGPKVNVEAPDVNLEGLGGKLKGPDVKLPDMSVKTPKISMPDVDLHVKGTKVKGEYDVTVPKLEGELKGPKVDIDAPDVDVHGPDWHLKMPKMKMPKFSVPGFKAEGPEVDVNLPKADVDISGPKIDVTAPDVSIEEPEGKLKGPKFKMPEMNIKVPKISMPDVDLHLKGPNVKGEYDVTMPKVESEIKVPDVELKSAKMDIDVPDVEVQGPDWHLKMPKMKMPKFSMPGFKAEGPEVDVNLPKADVDISGPKVGVEVPDVNIEGPEGKLKGPKFKMPEMNIKAPKISMPDVDLHMKGPKVKGEYDMTVPKLEGDLKGPKVDVSAPDVEMQGPDWNLKMPKIKMPKFSMPSLKGEGPEFDVNLSKANVDISAPKVDTNAPDLSLEGPEGKLKGPKFKMPEMHFRAPKMSLPDVDLDLKGPKMKGNVDISAPKIEGEMQVPDVDIRGPKVDIKAPDVEGQGLDWSLKIPKMKMPKFSMPSLKGEGPEVDVNLPKADVVVSGPKVDIEAPDVSLEGPEGKLKGPKFKMPEMHFKTPKISMPDVDLHLKGPKVKGDVDVSVPKVEGEMKVPDVEIKGPKMDIDAPDVEVQGPDWHLKMPKMKMPKFSMPGFKGEGREVDVNLPKADIDVSGPKVDVEVPDVSLEGPEGKLKGPKFKMPEMHFKAPKISMPDVDLNLKGPKLKGDVDVSLPEVEGEMKVPDVDIKGPKVDISAPDVDVHGPDWHLKMPKVKMPKFSMPGFKGEGPEVDVKLPKADVDVSGPKMDAEVPDVNIEGPDAKLKGPKFKMPEMSIKPQKISIPDVGLHLKGPKMKGDYDVTVPKVEGEIKAPDVDIKGPKVDINAPDVEVHGPDWHLKMPKVKMPKFSMPGFKGEGPEVDMNLPKADLGVSGPKVDIDVPDVNLEAPEGKLKGPKFKMPSMNIQTHKISMPDVGLNLKAPKLKTDVDVSLPKVEGDLKGPEIDVKAPKMDVNVGDIDIEGPEGKLKGPKFKMPEMHFKAPKISMPDVDLHLKGPKVKGDMDVSVPKVEGEMKVPDVDIKGPKVDIDAPDVEVHDPDWHLKMPKMKMPKFSMPGFKAEGPEVDVNLPKADIDVSGPSVDTDAPDLDIEGPEGKLKGSKFKMPKLNIKAPKVSMPDVDLNLKGPKLKGEIDASVPELEGDLRGPQVDVKGPFVEAEVPDVDLECPDAKLKGPKFKMPEMHFKAPKISMPDVDLHLKGPKVKGDADVSVPKLEGDLTGPSVGVEVPDVELECPDAKLKGPKFKMPDMHFKAPKISMPDVDLHLKGPKVKGDVDVSVPKLEGDLTGPSVGVEVPDVELECPDAKLKGPKFKMPEMHFKTPKISMPDVDLHLKGPKVKGDMDVSVPKVEGEMKVPDVDIKGPKMDIDAPDVDVHGPDWHLKMPKMKMPKFSMPGFKAEGPEVDVNLPKADVVVSGPKVDVEVPDVSLEGPEGKLKGPKLKMPEMHFKAPKISMPDVDLHLKGPKVKGDVDVSLPKLEGDLTGPSVDVEVPDVELECPDAKLKGPKFKMPEMHFKTPKISMPDVNLNLKGPKVKGDMDVSVPKVEGEMKVPDVDIRGPKVDIDAPDVDVHGPDWHLKMPKMKMPKFSMPGFKGEGPEVDVNLPKADVDVSGPKVDVEVPDVSLEGPEGKLKGPKFKMPEMHFKTPKISMPDVDFNLKGPKIKGDVDVSAPKLEGELKGPELDVKGPKLDADMPEVAVEGPNGKWKTPKFKMPDMHFKAPKISMPDLDLHLKSPKAKGEVDVDVPKLEGDLKGPHVDVSGPDIDIEGPEGKLKGPKFKMPDMHFKAPNISMPDVDLNLKGPKIKGDVDVSVPEVEGKLEVPDMNIRGPKVDVNAPDVQAPDWHLKMPKMKMPKFSMPGFKAEGPEVDVNLPKADVDISGPKVDIEGPDVNIEGPEGKLKGPKLKMPEMNIKAPKISMPDFDLHLKGPKVKGDVDVSLPKVEGDLKGPEVDIKGPKVDINAPDVGVQGPDWHLKMPKVKMPKFSMPGFKGEGPDGDVKLPKADIDVSGPKVDIEGPDVNIEGPEGKLKGPKFKMPEMNIKAPKISMPDIDLNLKGPKVKGDVDVSLPKVEGDLKGPEVDIKGPKVDIDAPDVDVHGPDWHLKMPKIKMPKISMPGFKGEGPDVDVNLPKADIDVSGPKVDVECPDVNIEGPEGKWKSPKFKMPEMHFKTPKISMPDIDLNLTGPKIKGDVDVTGPKVEGDLKGPEVDLKGPKVDIDVPDVNVQGPDWHLKMPKMKMPKFSMPGFKAEGPEVDVNLPKADVDVSGPKVDVEGPDVNIEGPEGKLKGPKFKMPEMNIKAPKIPMPDFDLHLKGPKVKGDVDISLPKVEGDLKGPEVDIRGPQVDIDVPDVGVQGPDWHLKMPKVKMPKFSMPGFKGEGPDVDVNLPKADLDVSGPKVDIDVPDVNIEGPEGKLKGPKFKMPEMNIKAPKISMPDIDLNLKGPKVKGDMDVSLPKVEGDMKVPDVDIKGPKVDINAPDVDVQGPDWHLKMPKIKMPKISMPGFKGEGPEVDVNLPKADLDVSGPKVDVDVPDVNIEGPDAKLKGPKFKMPEMNIKAPKISMPDLDLNLKGPKMKGEVDVSLANVEGDLKGPALDIKGPKIDVDAPDIDIHGPDAKLKGPKLKMPDMHVNMPKISMPEIDLNLKGSKLKGDVDVSGPKLEGDIKAPSLDIKGPEVDVSGPKLNIEGKSKKSRFKLPKFNFSGSKVQTPEVDVKGKKPDIDITGPKVDINAPDVEVQGKVKGSKFKMPFLSISSPKVSMPDVELNLKSPKVKGDLDIAGPNLEGDFKGPKVDIKAPEVNLNAPDVDVHGPDWNLKMPKMKMPKFSVSGLKAEGPDVAVDLPKGDINIEGPSMNIEGPDLNVEGPEGGLKGPKFKMPDMNIKAPKISMPDIDLNLKGPKVKGDVDISLPKLEGDLKGPEVDIKGPKVDINAPDVDVHGPDWHLKMPKVKMPKFSMPGFKGEGPEVDVTLPKADIDISGPNVDVDVPDVNIEGPDAKLKGPKFKMPEMNIKAPKISMPDFDLNLKGPKMKGDVVVSLPKVEGDLKGPEVDIKGPKVDIDTPDINIEGSEGKFKGPKFKIPEMHLKAPKISMPDIDLNLKGPKVKGDVDVSLPKMEGDLKGPEVDIKGPKVDINAPDVDVQGPDWHLKMPKVKMPKFSMPGFKGEGPDVDVNLPKADLDVSGPKVDIDVPDVNIEGPEGKLKGPKFKMPEMNIKAPKISMPDIDLNLKGPKVKGDMDVSLPKVEGDMQVPDLDIKGPKVDINAPDVDVRGPDWHLKMPKIKMPKISMPGFKGEGPEVDVNLPKADLDVSGPKVDVDVPDVNIEGPDAKLKGPKFKMPEMNIKAPKISMPDFDLHLKGPKVKGDVDVSLPKMEGDLKAPEVDIKGPKVDIDAPDVDVHGPDWHLKMPKVKMPKFSMPGFKGEGPEVDVNLPKADIDVSGPKVDIDTPDIDIHGPEGKLKGPKFKMPDLHLKAPKISMPEVDLNLKGPKMKGDVDVSLPKVEGDLKGPEVDIKGPKVDIDVPDVDVQGPDWHLKMPKVKMPKFSMPGFKGEGPDVDVNLPKADLDVSGPKVDIDVPDVNIEGPDAKLKGPKFKMPEMNIKAPKISMPDFDLHLKGPKVKGDVDVSLPKVEGDLKGPEVDIKGPKVDIDAPDVDVHGPDWHLKMPKVKMPKFSMPGFKGEGPDVDVTLPKADIEISGPKVDIDAPDVSIEGPDAKLKGPKFKMPEMNIKAPKISMPDIDFNLKGPKVKGDVDVSLPKVEGDLKGPEIDIKGPSLDIDTPDVNIEGPEGKLKGPKFKMPEMNIKAPKISMPDFDLHLKGPKVKGDVDVSLPKVESDLKGPEVDIEGPEGKLKGPKFKMPDVHFKSPQISMSDIDLNLKGPKIKGDMDISVPKLEGDLKGPKVDVKGPKVGIDTPDIDIHGPEGKLKGPKFKMPDLHLKAPKISMPEVDLNLKGPKVKGDMDISLPKVEGDLKGPEVDIRDPKVDIDVPDVDVQGPDWHLKMPKVKMPKFSMPGFKGEGPDVDVNLPKADIDVSGPKVDVDVPDVNIEGPDAKLKGPKFKMPEMSIKAPKISMPDIDLNLKGPKVKGDVDVTLPKVEGDLKGPEADIKGPKVDINTPDVDVHGPDWHLKMPKVKMPKFSMPGFKGEGPDVDVSLPKADIDVSGPKVDVDIPDVNIEGPDAKLKGPKFKMPEINIKAPKISIPDVDLDLKGPKVKGDFDVSVPKVEGTLKGPEVDLKGPRLDFEGPDAKLSGPSLKMPSLEISAPKVTAPDVDLHLKAPKIGFSGPKLEGGEVDLKGPKVEAPSLDVHMDSPDINIEGPDVKIPKFKKPKFGFGAKSPKADIKSPSLDVTVPEAELNLETPEISVGGKGKKSKFKMPKIHMSGPKIKAKKQGFDLNVPGGEIDASLKAPDVDVNIAGPDAALKVDVKSPKTKKTMFGKMYFPDVEFDIKSPKFKAEAPLPSPKLEGELQAPDLELSLPAIHVEGLDIKAKAPKVKMPDVDISVPKIEGDLKGPKVQANLGAPDINIEGLDAKVKTPSFGISAPQVSIPDVNVNLKGPKIKGDVPSVGLEGPDVDLQGPEAKIKFPKFSMPKIGIPGVKMEGGGAEVHAQLPSLEGDLRGPDVKLEGPDVSLKGPGVDLPSVNLSMPKVSGPDLDLNLKGPSLKGDLDASVPSMKVHAPGLNLSGVGGKMQVGGDGVKVPGIDATTKLNVGAPDVTLRGPSLQGDLAVSGDIKCPKVSVGAPDLSLEASEGSIKLPKMKLPQFGISTPGSDLHVNAKGPQVSGELKGPGVDVNLKGPRISAPNVDFNLEGPKVKGSLGATGEIKGPTVGGGLPGIGVQGLEGNLQMPGIKSSGCDVNLPGVNVKLPTGQISGPEIKGGLKGSEVGFHGAAPDISVKGPAFNMASPESDFGINLKGPKIKGGADVSGGVSAPDISLGEGHLSVKGSGGEWKGPQVSSALNLDTSKFAGGLHFSGPKVEGGVKGGQIGLQAPGLSVSGPQGHLESGSGKVTFPKMKIPKFTFSGRELVGREMGVDVHFPKAEASIQAGAGDGEWEESEVKLKKSKIKMPKFNFSKPKGKGGVTGSPEASISGSKGDLKSSKASLGSLEGEAEAEASSPKGKFSLFKSKKPRHRSNSFSDEREFSGPSTPTGTLEFEGGEVSLEGGKVKGKHGKLKFGTFGGLGSKSKGHYEVTGSDDETGKLQGSGVSLASKKSRLSSSSSNDSGNKVGIQLPEVELSVSTKKE

2) >gi|41322916|ref|NP_958782.1| plectin isoform 1 [Homo sapiens] (531.5 kD)

MVAGMLMPRDQLRAIYEVLFREGVMVAKKDRRPRSLHPHVPGVTNLQVMRAMASLRARGLVRETFAWCHFYWYLTNEGIAHLRQYLHLPPEIVPASLQRVRRPVAMVMPARRTPHVQAVQGPLGSPPKRGPLPTEEQRVYRRKELEEVSPETPVVPATTQRTLARPGPEPAPATDERDRVQKKTFTKWVNKHLIKAQRHISDLYEDLRDGHNLISLLEVLSGDSLPREKGRMRFHKLQNVQIALDYLRHRQVKLVNIRNDDIADGNPKLTLGLIWTIILHFQISDIQVSGQSEDMTAKEKLLLWSQRMVEGYQGLRCDNFTSSWRDGRLFNAIIHRHKPLLIDMNKVYRQTNLENLDQAFSVAERDLGVTRLLDPEDVDVPQPDEKSIITYVSSLYDAMPRVPDVQDGVRANELQLRWQEYRELVLLLLQWMRHHTAAFEERRFPSSFEEIEILWSQFLKFKEMELPAKEADKNRSKGIYQSLEGAVQAGQLKVPPGYHPLDVEKEWGKLHVAILEREKQLRSEFERLECLQRIVTKLQMEAGLCEEQLNQADALLQSDVRLLAAGKVPQRAGEVERDLDKADSMIRLLFNDVQTLKDGRHPQGEQMYRRVYRLHERLVAIRTEYNLRLKAGVAAPATQVAQVTLQSVQRRPELEDSTLRYLQDLLAWVEENQHRVDGAEWGVDLPSVEAQLGSHRGLHQSIEEFRAKIERARSDEGQLSPATRGAYRDCLGRLDLQYAKLLNSSKARLRSLESLHSFVAAATKELMWLNEKEEEEVGFDWSDRNTNMTAKKESYSALMRELELKEKKIKELQNAGDRLLREDHPARPTVESFQAALQTQWSWMLQLCCCIEAHLKENAAYFQFFSDVREAEGQLQKLQEALRRKYSCDRSATVTRLEDLLQDAQDEKEQLNEYKGHLSGLAKRAKAVVQLKPRHPAHPMRGRLPLLAVCDYKQVEVTVHKGDECQLVGPAQPSHWKVLSSSGSEAAVPSVCFLVPPPNQEAQEAVTRLEAQHQALVTLWHQLHVDMKSLLAWQSLRRDVQLIRSWSLATFRTLKPEEQRQALHSLELHYQAFLRDSQDAGGFGPEDRLMAEREYGSCSHHYQQLLQSLEQGAQEESRCQRCISELKDIRLQLEACETRTVHRLRLPLDKEPARECAQRIAEQQKAQAEVEGLGKGVARLSAEAEKVLALPEPSPAAPTLRSELELTLGKLEQVRSLSAIYLEKLKTISLVIRGTQGAEEVLRAHEEQLKEAQAVPATLPELEATKASLKKLRAQAEAQQPTFDALRDELRGAQEVGERLQQRHGERDVEVERWRERVAQLLERWQAVLAQTDVRQRELEQLGRQLRYYRESADPLGAWLQDARRRQEQIQAMPLADSQAVREQLRQEQALLEEIERHGEKVEECQRFAKQYINAIKDYELQLVTYKAQLEPVASPAKKPKVQSGSESVIQEYVDLRTHYSELTTLTSQYIKFISETLRRMEEEERLAEQQRAEERERLAEVEAALEKQRQLAEAHAQAKAQAEREAKELQQRMQEEVVRREEAAVDAQQQKRSIQEELQQLRQSSEAEIQAKARQAEAAERSRLRIEEEIRVVRLQLEATERQRGGAEGELQALRARAEEAEAQKRQAQEEAERLRRQVQDESQRKRQAEVELASRVKAEAEAAREKQRALQALEELRLQAEEAERRLRQAEVERARQVQVALETAQRSAEAELQSKRASFAEKTAQLERSLQEEHVAVAQLREEAERRAQQQAEAERAREEAERELERWQLKANEALRLRLQAEEVAQQKSLAQAEAEKQKEEAEREARRRGKAEEQAVRQRELAEQELEKQRQLAEGTAQQRLAAEQELIRLRAETEQGEQQRQLLEEELARLQREAAAATQKRQELEAELAKVRAEMEVLLASKARAEEESRSTSEKSKQRLEAEAGRFRELAEEAARLRALAEEAKRQRQLAEEDAARQRAEAERVLAEKLAAIGEATRLKTEAEIALKEKEAENERLRRLAEDEAFQRRRLEEQAAQHKADIEERLAQLRKASDSELERQKGLVEDTLRQRRQVEEEILALKASFEKAAAGKAELELELGRIRSNAEDTLRSKEQAELEAARQRQLAAEEERRRREAEERVQKSLAAEEEAARQRKAALEEVERLKAKVEEARRLRERAEQESARQLQLAQEAAQKRLQAEEKAHAFAVQQKEQELQQTLQQEQSVLDQLRGEAEAARRAAEEAEEARVQAEREAAQSRRQVEEAERLKQSAEEQAQARAQAQAAAEKLRKEAEQEAARRAQAEQAALRQKQAADAEMEKHKKFAEQTLRQKAQVEQELTTLRLQLEETDHQKNLLDEELQRLKAEATEAARQRSQVEEELFSVRVQMEELSKLKARIEAENRALILRDKDNTQRFLQEEAEKMKQVAEEAARLSVAAQEAARLRQLAEEDLAQQRALAEKMLKEKMQAVQEATRLKAEAELLQQQKELAQEQARRLQEDKEQMAQQLAEETQGFQRTLEAERQRQLEMSAEAERLKLRVAEMSRAQARAEEDAQRFRKQAEEIGEKLHRTELATQEKVTLVQTLEIQRQQSDHDAERLREAIAELEREKEKLQQEAKLLQLKSEEMQTVQQEQLLQETQALQQSFLSEKDSLLQRERFIEQEKAKLEQLFQDEVAKAQQLREEQQRQQQQMEQERQRLVASMEEARRRQHEAEEGVRRKQEELQQLEQQRRQQEELLAEENQRLREQLQLLEEQHRAALAHSEEVTASQVAATKTLPNGRDALDGPAAEAEPEHSFDGLRRKVSAQRLQEAGILSAEELQRLAQGHTTVDELARREDVRHYLQGRSSIAGLLLKATNEKLSVYAALQRQLLSPGTALILLEAQAASGFLLDPVRNRRLTVNEAVKEGVVGPELHHKLLSAERAVTGYKDPYTGQQISLFQAMQKGLIVREHGIRLLEAQIATGGVIDPVHSHRVPVDVAYRRGYFDEEMNRVLADPSDDTKGFFDPNTHENLTYLQLLERCVEDPETGLCLLPLTDKAAKGGELVYTDSEARDVFEKATVSAPFGKFQGKTVTIWEIINSEYFTAEQRRDLLRQFRTGRITVEKIIKIIITVVEEQEQKGRLCFEGLRSLVPAAELLESRVIDRELYQQLQRGERSVRDVAEVDTVRRALRGANVIAGVWLEEAGQKLSIYNALKKDLLPSDMAVALLEAQAGTGHIIDPATSARLTVDEAVRAGLVGPEFHEKLLSAEKAVTGYRDPYTGQSVSLFQALKKGLIPREQGLRLLDAQLSTGGIVDPSKSHRVPLDVACARGCLDEETSRALSAPRADAKAYSDPSTGEPATYGELQQRCRPDQLTGLSLLPLSEKAARARQEELYSELQARETFEKTPVEVPVGGFKGRTVTVWELISSEYFTAEQRQELLRQFRTGKVTVEKVIKILITIVEEVETLRQERLSFSGLRAPVPASELLASGVLSRAQFEQLKDGKTTVKDLSELGSVRTLLQGSGCLAGIYLEDTKEKVSIYEAMRRGLLRATTAALLLEAQAATGFLVDPVRNQRLYVHEAVKAGVVGPELHEQLLSAEKAVTGYRDPYSGSTISLFQAMQKGLVLRQHGIRLLEAQIATGGIIDPVHSHRVPVDVAYQRGYFSEEMNRVLADPSDDTKGFFDPNTHENLTYRQLLERCVEDPETGLRLLPLKGAEKAEVVETTQVYTEEETRRAFEETQIDIPGGGSHGGSTMSLWEVMQSDLIPEEQRAQLMADFQAGRVTKERMIIIIIEIIEKTEIIRQQGLASYDYVRRRLTAEDLFEARIISLETYNLLREGTRSLREALEAESAWCYLYGTGSVAGVYLPGSRQTLSIYQALKKGLLSAEVARLLLEAQAATGFLLDPVKGERLTVDEAVRKGLVGPELHDRLLSAERAVTGYRDPYTEQTISLFQAMKKELIPTEEALRLLDAQLATGGIVDPRLGFHLPLEVAYQRGYLNKDTHDQLSEPSEVRSYVDPSTDERLSYTQLLRRCRRDDGTGQLLLPLSDARKLTFRGLRKQITMEELVRSQVMDEATALQLREGLTSIEEVTKNLQKFLEGTSCIAGVFVDATKERLSVYQAMKKGIIRPGTAFELLEAQAATGYVIDPIKGLKLTVEEAVRMGIVGPEFKDKLLSAERAVTGYKDPYSGKLISLFQAMKKGLILKDHGIRLLEAQIATGGIIDPEESHRLPVEVAYKRGLFDEEMNEILTDPSDDTKGFFDPNTEENLTYLQLMERCITDPQTGLCLLPLKEKKRERKTSSKSSVRKRRVVIVDPETGKEMSVYEAYRKGLIDHQTYLELSEQECEWEEITISSSDGVVKSMIIDRRSGRQYDIDDAIAKNLIDRSALDQYRAGTLSITEFADMLSGNAGGFRSRSSSVGSSSSYPISPAVSRTQLASWSDPTEETGPVAGILDTETLEKVSITEAMHRNLVDNITGQRLLEAQACTGGIIDPSTGERFPVTDAVNKGLVDKIMVDRINLAQKAFCGFEDPRTKTKMSAAQALKKGWLYYEAGQRFLEVQYLTGGLIEPDTPGRVPLDEALQRGTVDARTAQKLRDVGAYSKYLTCPKTKLKISYKDALDRSMVEEGTGLRLLEAAAQSTKGYYSPYSVSGSGSTAGSRTGSRTGSRAGSRRGSFDATGSGFSMTFSSSSYSSSGYGRRYASGSSASLGGPESAVA

3) >gi|32967601|ref|NP_066267.2| ankyrin-3 isoform 1 [Homo sapiens] (480.1 kD)

MAHAASQLKKNRDLEINAEEEPEKKRKHRKRSRDRKKKSDANASYLRAARAGHLEKALDYIKNGVDINICNQNGLNALHLASKEGHVEVVSELLQREANVDAATKKGNTALHIASLAGQAEVVKVLVTNGANVNAQSQNGFTPLYMAAQENHLEVVKFLLDNGASQSLATEDGFTPLAVALQQGHDQVVSLLLENDTKGKVRLPALHIAARKDDTKAAALLLQNDNNADVESKSGFTPLHIAAHYGNINVATLLLNRAAAVDFTARNDITPLHVASKRGNANMVKLLLDRGAKIDAKTRDGLTPLHCGARSGHEQVVEMLLDRAAPILSKTKNGLSPLHMATQGDHLNCVQLLLQHNVPVDDVTNDYLTALHVAAHCGHYKVAKVLLDKKANPNAKALNGFTPLHIACKKNRIKVMELLLKHGASIQAVTESGLTPIHVAAFMGHVNIVSQLMHHGASPNTTNVRGETALHMAARSGQAEVVRYLVQDGAQVEAKAKDDQTPLHISARLGKADIVQQLLQQGASPNAATTSGYTPLHLSAREGHEDVAAFLLDHGASLSITTKKGFTPLHVAAKYGKLEVANLLLQKSASPDAAGKSGLTPLHVAAHYDNQKVALLLLDQGASPHAAAKNGYTPLHIAAKKNQMDIATTLLEYGADANAVTRQGIASVHLAAQEGHVDMVSLLLGRNANVNLSNKSGLTPLHLAAQEDRVNVAEVLVNQGAHVDAQTKMGYTPLHVGCHYGNIKIVNFLLQHSAKVNAKTKNGYTPLHQAAQQGHTHIINVLLQNNASPNELTVNGNTALGIARRLGYISVVDTLKIVTEETMTTTTVTEKHKMNVPETMNEVLDMSDDEVRKANAPEMLSDGEYISDVEEGEDAMTGDTDKYLGPQDLKELGDDSLPAEGYMGFSLGARSASLRSFSSDRSYTLNRSSYARDSMMIEELLVPSKEQHLTFTREFDSDSLRHYSWAADTLDNVNLVSSPIHSGFLVSFMVDARGGSMRGSRHHGMRIIIPPRKCTAPTRITCRLVKRHKLANPPPMVEGEGLASRLVEMGPAGAQFLGPVIVEIPHFGSMRGKERELIVLRSENGETWKEHQFDSKNEDLTELLNGMDEELDSPEELGKKRICRIITKDFPQYFAVVSRIKQESNQIGPEGGILSSTTVPLVQASFPEGALTKRIRVGLQAQPVPDEIVKKILGNKATFSPIVTVEPRRRKFHKPITMTIPVPPPSGEGVSNGYKGDTTPNLRLLCSITGGTSPAQWEDITGTTPLTFIKDCVSFTTNVSARFWLADCHQVLETVGLATQLYRELICVPYMAKFVVFAKMNDPVESSLRCFCMTDDKVDKTLEQQENFEEVARSKDIEVLEGKPIYVDCYGNLAPLTKGGQQLVFNFYSFKENRLPFSIKIRDTSQEPCGRLSFLKEPKTTKGLPQTAVCNLNITLPAHKKETESDQDDEIEKTDRRQSFASLALRKRYSYLTEPGMIERSTGATRSLPTTYSYKPFFSTRPYQSWTTAPITVPGPAKSGFTSLSSSSSNTPSASPLKSIWSVSTPSPIKSTLGASTTSSVKSISDVASPIRSFRTMSSPIKTVVSQSPYNIQVSSGTLARAPAVTEATPLKGLASNSTFSSRTSPVTTAGSLLERSSITMTPPASPKSNINMYSSSLPFKSIITSAAPLISSPLKSVVSPVKSAVDVISSAKITMASSLSSPVKQMPGHAEVALVNGSISPLKYPSSSTLINGCKATATLQEKISSATNSVSSVVSAATDTVEKVFSTTTAMPFSPLRSYVSAAPSAFQSLRTPSASALYTSLGSSISATTSSVTSSIITVPVYSVVNVLPEPALKKLPDSNSFTKSAAALLSPIKTLTTETHPQPHFSRTSSPVKSSLFLAPSALKLSTPSSLSSSQEILKDVAEMKEDLMRMTAILQTDVPEEKPFQPELPKEGRIDDEEPFKIVEKVKEDLVKVSEILKKDVCVDNKGSPKSPKSDKGHSPEDDWIEFSSEEIREARQQAAASQSPSLPERVQVKAKAASEKDYNLTKVIDYLTNDIGSSSLTNLKYKFEDAKKDGEERQKRVLKPAIALQEHKLKMPPASMRTSTSEKELCKMADSFFGTDTILESPDDFSQHDQDKSPLSDSGFETRSEKTPSAPQSAESTGPKPLFHEVPIPPVITETRTEVVHVIRSYDPSAGDVPQTQPEEPVSPKPSPTFMELEPKPTTSSIKEKVKAFQMKASSEEDDHNRVLSKGMRVKEETHITTTTRMVYHSPPGGEGASERIEETMSVHDIMKAFQSGRDPSKELAGLFEHKSAVSPDVHKSAAETSAQHAEKDNQMKPKLERIIEVHIEKGNQAEPTEVIIRETKKHPEKEMYVYQKDLSRGDINLKDFLPEKHDAFPCSEEQGQQEEEELTAEESLPSYLESSRVNTPVSQEEDSRPSSAQLISDDSYKTLKLLSQHSIEYHDDELSELRGESYRFAEKMLLSEKLDVSHSDTEESVTDHAGPPSSELQGSDKRSREKIATAPKKEILSKIYKDVSENGVGKVSKDEHFDKVTVLHYSGNVSSPKHAMWMRFTEDRLDRGREKLIYEDRVDRTVKEAEEKLTEVSQFFRDKTEKLNDELQSPEKKARPKNGKEYSSQSPTSSSPEKVLLTELLASNDEWVKARQHGPDGQGFPKAEEKAPSLPSSPEKMVLSQQTEDSKSTVEAKGSISQSKAPDGPQSGFQLKQSKLSSIRLKFEQGTHAKSKDMSQEDRKSDGQSRIPVKKIQESKLPVYQVFAREKQQKAIDLPDESVSVQKDFMVLKTKDEHAQSNEIVVNDSGSDNVKKQRTEMSSKAMPDSFSEQQAKDLACHITSDLATRGPWDKKVFRTWESSGATNNKSQKEKLSHVLVHDVRENHIGHPESKSVDQKNEFMSVTERERKLLTNGSLSEIKEMTVKSPSKKVLYREYVVKEGDHPGGLLDQPSRRSESSAVSHIPVRVADERRMLSSNIPDGFCEQSAFPKHELSQKLSQSSMSKETVETQHFNSIEDEKVTYSEISKVSKHQSYVGLCPPLEETETSPTKSPDSLEFSPGKESPSSDVFDHSPIDGLEKLAPLAQTEGGKEIKTLPVYVSFVQVGKQYEKEIQQGGVKKIISQECKTVQETRGTFYTTRQQKQPPSPQGSPEDDTLEQVSFLDSSGKSPLTPETPSSEEVSYEFTSKTPDSLIAYIPGKPSPIPEVSEESEEEEQAKSTSLKQTTVEETAVEREMPNDVSKDSNQRPKNNRVAYIEFPPPPPLDADQIESDKKHHYLPEKEVDMIEVNLQDEHDKYQLAEPVIRVQPPSPVPPGADVSDSSDDESIYQPVPVKKYTFKLKEVDDEQKEKPKASAEKASNQKELESNGSGKDNEFGLGLDSPQNEIAQNGNNDQSITECSIATTAEFSHDTDATEIDSLDGYDLQDEDDGLTESDSKLPIQAMEIKKDIWNTEGILKPADRSFSQSKLEVIEEEGKVGPDEDKPPSKSSSSEKTPDKTDQKSGAQFFTLEGRHPDRSVFPDTYFSYKVDEEFATPFKTVATKGLDFDPWSNNRGDDEVFDSKSREDETKPFGLAVEDRSPATTPDTTPARTPTDESTPTSEPNPFPFHEGKMFEMTRSGAIDMSKRDFVEERLQFFQIGEHTSEGKSGDQGEGDKSMVTATPQPQSGDTTVETNLERNVETPTVEPNPSIPTSGECQEGTSSSGSLEKSAAATNTSKVDPKLRTPIKMGISASTMTMKKEGPGEITDKIEAVMTSCQGLENETITMISNTANSQMGVRPHEKHDFQKDNFNNNNNLDSSTIQTDNIMSNIVLTEHSAPTCTTEKDNPVKVSSGKKTGVLQGHCVRDKQKVLGEQQKTKELIGIRQKSKLPIKATSPKDTFPPNHMSNTKASKMKQVSQSEKTKALTTSSCVDVKSRIPVKNTHRDNIIAVRKACATQKQGQPEKGKAKQLPSKLPVKVRSTCVTTTTTTATTTTTTTTTTTTSCTVKVRKSQLKEVCKHSIEYFKGISGETLKLVDRLSEEEKKMQSELSDEEESTSRNTSLSETSRGGQPSVTTKSARDKKTEAAPLKSKSEKAGSEKRSSRRTGPQSPCERTDIRMAIVADHLGLSWTELARELNFSVDEINQIRVENPNSLISQSFMLLKKWVTRDGKNATTDALTSVLTKINRIDIVTLLEGPIFDYGNISGTRSFADENNVFHDPVDGWQNETSSGNLESCAQARRVTGGLLDRLDDSPDQCRDSITSYLKGEAGKFEANGSHTEITPEAKTKSYFPESQNDVGKQSTKETLKPKIHGSGHVEEPASPLAAYQKSLEETSKLIIEETKPCVPVSMKKMSRTSPADGKPRLSLHEEEGSSGSEQKQGEGFKVKTKKEIRHVEKKSHS

4) >gi|13654237|ref|NP_008835.5| DNA-dependent protein kinase catalytic subunit isoform 1 [Homo sapiens] (468.8 kD)

MAGSGAGVRCSLLRLQETLSAADRCGAALAGHQLIRGLGQECVLSSSPAVLALQTSLVFSRDFGLLVFVRKSLNSIEFRECREEILKFLCIFLEKMGQKIAPYSVEIKNTCTSVYTKDRAAKCKIPALDLLIKLLQTFRSSRLMDEFKIGELFSKFYGELALKKKIPDTVLEKVYELLGLLGEVHPSEMINNAENLFRAFLGELKTQMTSAVREPKLPVLAGCLKGLSSLLCNFTKSMEEDPQTSREIFNFVLKAIRPQIDLKRYAVPSAGLRLFALHASQFSTCLLDNYVSLFEVLLKWCAHTNVELKKAALSALESFLKQVSNMVAKNAEMHKNKLQYFMEQFYGIIRNVDSNNKELSIAIRGYGLFAGPCKVINAKDVDFMYVELIQRCKQMFLTQTDTGDDRVYQMPSFLQSVASVLLYLDTVPEVYTPVLEHLVVMQIDSFPQYSPKMQLVCCRAIVKVFLALAAKGPVLRNCISTVVHQGLIRICSKPVVLPKGPESESEDHRASGEVRTGKWKVPTYKDYVDLFRHLLSSDQMMDSILADEAFFSVNSSSESLNHLLYDEFVKSVLKIVEKLDLTLEIQTVGEQENGDEAPGVWMIPTSDPAANLHPAKPKDFSAFINLVEFCREILPEKQAEFFEPWVYSFSYELILQSTRLPLISGFYKLLSITVRNAKKIKYFEGVSPKSLKHSPEDPEKYSCFALFVKFGKEVAVKMKQYKDELLASCLTFLLSLPHNIIELDVRAYVPALQMAFKLGLSYTPLAEVGLNALEEWSIYIDRHVMQPYYKDILPCLDGYLKTSALSDETKNNWEVSALSRAAQKGFNKVVLKHLKKTKNLSSNEAISLEEIRIRVVQMLGSLGGQINKNLLTVTSSDEMMKSYVAWDREKRLSFAVPFREMKPVIFLDVFLPRVTELALTASDRQTKVAACELLHSMVMFMLGKATQMPEGGQGAPPMYQLYKRTFPVLLRLACDVDQVTRQLYEPLVMQLIHWFTNNKKFESQDTVALLEAILDGIVDPVDSTLRDFCGRCIREFLKWSIKQITPQQQEKSPVNTKSLFKRLYSLALHPNAFKRLGASLAFNNIYREFREEESLVEQFVFEALVIYMESLALAHADEKSLGTIQQCCDAIDHLCRIIEKKHVSLNKAKKRRLPRGFPPSASLCLLDLVKWLLAHCGRPQTECRHKSIELFYKFVPLLPGNRSPNLWLKDVLKEEGVSFLINTFEGGGCGQPSGILAQPTLLYLRGPFSLQATLCWLDLLLAALECYNTFIGERTVGALQVLGTEAQSSLLKAVAFFLESIAMHDIIAAEKCFGTGAAGNRTSPQEGERYNYSKCTVVVRIMEFTTTLLNTSPEGWKLLKKDLCNTHLMRVLVQTLCEPASIGFNIGDVQVMAHLPDVCVNLMKALKMSPYKDILETHLREKITAQSIEELCAVNLYGPDAQVDRSRLAAVVSACKQLHRAGLLHNILPSQSTDLHHSVGTELLSLVYKGIAPGDERQCLPSLDLSCKQLASGLLELAFAFGGLCERLVSLLLNPAVLSTASLGSSQGSVIHFSHGEYFYSLFSETINTELLKNLDLAVLELMQSSVDNTKMVSAVLNGMLDQSFRERANQKHQGLKLATTILQHWKKCDSWWAKDSPLETKMAVLALLAKILQIDSSVSFNTSHGSFPEVFTTYISLLADTKLDLHLKGQAVTLLPFFTSLTGGSLEELRRVLEQLIVAHFPMQSREFPPGTPRFNNYVDCMKKFLDALELSQSPMLLELMTEVLCREQQHVMEELFQSSFRRIARRGSCVTQVGLLESVYEMFRKDDPRLSFTRQSFVDRSLLTLLWHCSLDALREFFSTIVVDAIDVLKSRFTKLNESTFDTQITKKMGYYKILDVMYSRLPKDDVHAKESKINQVFHGSCITEGNELTKTLIKLCYDAFTENMAGENQLLERRRLYHCAAYNCAISVICCVFNELKFYQGFLFSEKPEKNLLIFENLIDLKRRYNFPVEVEVPMERKKKYIEIRKEAREAANGDSDGPSYMSSLSYLADSTLSEEMSQFDFSTGVQSYSYSSQDPRPATGRFRRREQRDPTVHDDVLELEMDELNRHECMAPLTALVKHMHRSLGPPQGEEDSVPRDLPSWMKFLHGKLGNPIVPLNIRLFLAKLVINTEEVFRPYAKHWLSPLLQLAASENNGGEGIHYMVVEIVATILSWTGLATPTGVPKDEVLANRLLNFLMKHVFHPKRAVFRHNLEIIKTLVECWKDCLSIPYRLIFEKFSGKDPNSKDNSVGIQLLGIVMANDLPPYDPQCGIQSSEYFQALVNNMSFVRYKEVYAAAAEVLGLILRYVMERKNILEESLCELVAKQLKQHQNTMEDKFIVCLNKVTKSFPPLADRFMNAVFFLLPKFHGVLKTLCLEVVLCRVEGMTELYFQLKSKDFVQVMRHRDDERQKVCLDIIYKMMPKLKPVELRELLNPVVEFVSHPSTTCREQMYNILMWIHDNYRDPESETDNDSQEIFKLAKDVLIQGLIDENPGLQLIIRNFWSHETRLPSNTLDRLLALNSLYSPKIEVHFLSLATNFLLEMTSMSPDYPNPMFEHPLSECEFQEYTIDSDWRFRSTVLTPMFVETQASQGTLQTRTQEGSLSARWPVAGQIRATQQQHDFTLTQTADGRSSFDWLTGSSTDPLVDHTSPSSDSLLFAHKRSERLQRAPLKSVGPDFGKKRLGLPGDEVDNKVKGAAGRTDLLRLRRRFMRDQEKLSLMYARKGVAEQKREKEIKSELKMKQDAQVVLYRSYRHGDLPDIQIKHSSLITPLQAVAQRDPIIAKQLFSSLFSGILKEMDKFKTLSEKNNITQKLLQDFNRFLNTTFSFFPPFVSCIQDISCQHAALLSLDPAAVSAGCLASLQQPVGIRLLEEALLRLLPAELPAKRVRGKARLPPDVLRWVELAKLYRSIGEYDVLRGIFTSEIGTKQITQSALLAEARSDYSEAAKQYDEALNKQDWVDGEPTEAEKDFWELASLDCYNHLAEWKSLEYCSTASIDSENPPDLNKIWSEPFYQETYLPYMIRSKLKLLLQGEADQSLLTFIDKAMHGELQKAILELHYSQELSLLYLLQDDVDRAKYYIQNGIQSFMQNYSSIDVLLHQSRLTKLQSVQALTEIQEFISFISKQGNLSSQVPLKRLLNTWTNRYPDAKMDPMNIWDDIITNRCFFLSKIEEKLTPLPEDNSMNVDQDGDPSDRMEVQEQEEDISSLIRSCKFSMKMKMIDSARKQNNFSLAMKLLKELHKESKTRDDWLVSWVQSYCRLSHCRSRSQGCSEQVLTVLKTVSLLDENNVSSYLSKNILAFRDQNILLGTTYRIIANALSSEPACLAEIEEDKARRILELSGSSSEDSEKVIAGLYQRAFQHLSEAVQAAEEEAQPPSWSCGPAAGVIDAYMTLADFCDQQLRKEEENASVIDSAELQAYPALVVEKMLKALKLNSNEARLKFPRLLQIIERYPEETLSLMTKEISSVPCWQFISWISHMVALLDKDQAVAVQHSVEEITDNYPQAIVYPFIISSESYSFKDTSTGHKNKEFVARIKSKLDQGGVIQDFINALDQLSNPELLFKDWSNDVRAELAKTPVNKKNIEKMYERMYAALGDPKAPGLGAFRRKFIQTFGKEFDKHFGKGGSKLLRMKLSDFNDITNMLLLKMNKDSKPPGNLKECSPWMSDFKVEFLRNELEIPGQYDGRGKPLPEYHVRIAGFDERVTVMASLRRPKRIIIRGHDEREHPFLVKGGEDLRQDQRVEQLFQVMNGILAQDSACSQRALQLRTYSVVPMTSRLGLIEWLENTVTLKDLLLNTMSQEEKAAYLSDPRAPPCEYKDWLTKMSGKHDVGAYMLMYKGANRTETVTSFRKRESKVPADLLKRAFVRMSTSPEAFLALRSHFASSHALICISHWILGIGDRHLNNFMVAMETGGVIGIDFGHAFGSATQFLPVPELMPFRLTRQFINLMLPMKETGLMYSIMVHALRAFRSDPGLLTNTMDVFVKEPSFDWKNFEQKMLKKGGSWIQEINVAEKNWYPRQKICYAKRKLAGANPAVITCDELLLGHEKAPAFRDYVAVARGSKDHNIRAQEPESGLSEETQVKCLMDQATDPNILGRTWEGWEPWM

5) >gi|58530840|ref|NP_004406.2| desmoplakin isoform I [Homo sapiens] (331.6 kD)

MSCNGGSHPRINTLGRMIRAESGPDLRYEVTSGGGGTSRMYYSRRGVITDQNSDGYCQTGTMSRHQNQNTIQELLQNCSDCLMRAELIVQPELKYGDGIQLTRSRELDECFAQANDQMEILDSLIREMRQMGQPCDAYQKRLLQLQEQMRALYKAISVPRVRRASSKGGGGYTCQSGSGWDEFTKHVTSECLGWMRQQRAEMDMVAWGVDLASVEQHINSHRGIHNSIGDYRWQLDKIKADLREKSAIYQLEEEYENLLKASFERMDHLRQLQNIIQATSREIMWINDCEEEELLYDWSDKNTNIAQKQEAFSIRMSQLEVKEKELNKLKQESDQLVLNQHPASDKIEAYMDTLQTQWSWILQITKCIDVHLKENAAYFQFFEEAQSTEAYLKGLQDSIRKKYPCDKNMPLQHLLEQIKELEKEREKILEYKRQVQNLVNKSKKIVQLKPRNPDYRSNKPIILRALCDYKQDQKIVHKGDECILKDNNERSKWYVTGPGGVDMLVPSVGLIIPPPNPLAVDLSCKIEQYYEAILALWNQLYINMKSLVSWHYCMIDIEKIRAMTIAKLKTMRQEDYMKTIADLELHYQEFIRNSQGSEMFGDDDKRKIQSQFTDAQKHYQTLVIQLPGYPQHQTVTTTEITHHGTCQDVNHNKVIETNRENDKQETWMLMELQKIRRQIEHCEGRMTLKNLPLADQGSSHHITVKINELKSVQNDSQAIAEVLNQLKDMLANFRGSEKYCYLQNEVFGLFQKLENINGVTDGYLNSLCTVRALLQAILQTEDMLKVYEARLTEEETVCLDLDKVEAYRCGLKKIKNDLNLKKSLLATMKTELQKAQQIHSQTSQQYPLYDLDLGKFGEKVTQLTDRWQRIDKQIDFRLWDLEKQIKQLRNYRDNYQAFCKWLYDAKRRQDSLESMKFGDSNTVMRFLNEQKNLHSEISGKRDKSEEVQKIAELCANSIKDYELQLASYTSGLETLLNIPIKRTMIQSPSGVILQEAADVHARYIELLTRSGDYYRFLSEMLKSLEDLKLKNTKIEVLEEELRLARDANSENCNKNKFLDQNLQKYQAECSQFKAKLASLEELKRQAELDGKSAKQNLDKCYGQIKELNEKITRLTYEIEDEKRRRKSVEDRFDQQKNDYDQLQKARQCEKENLGWQKLESEKAIKEKEYEIERLRVLLQEEGTRKREYENELAKVRNHYNEEMSNLRNKYETEINITKTTIKEISMQKEDDSKNLRNQLDRLSRENRDLKDEIVRLNDSILQATEQRRRAEENALQQKACGSEIMQKKQHLEIELKQVMQQRSEDNARHKQSLEEAAKTIQDKNKEIERLKAEFQEEAKRRWEYENELSKVRNNYDEEIISLKNQFETEINITKTTIHQLTMQKEEDTSGYRAQIDNLTRENRSLSEEIKRLKNTLTQTTENLRRVEEDIQQQKATGSEVSQRKQQLEVELRQVTQMRTEESVRYKQSLDDAAKTIQDKNKEIERLKQLIDKETNDRKCLEDENARLQRVQYDLQKANSSATETINKLKVQEQELTRLRIDYERVSQERTVKDQDITRFQNSLKELQLQKQKVEEELNRLKRTASEDSCKRKKLEEELEGMRRSLKEQAIKITNLTQQLEQASIVKKRSEDDLRQQRDVLDGHLREKQRTQEELRRLSSEVEALRRQLLQEQESVKQAHLRNEHFQKAIEDKSRSLNESKIEIERLQSLTENLTKEHLMLEEELRNLRLEYDDLRRGRSEADSDKNATILELRSQLQISNNRTLELQGLINDLQRERENLRQEIEKFQKQALEASNRIQESKNQCTQVVQERESLLVKIKVLEQDKARLQRLEDELNRAKSTLEAETRVKQRLECEKQQIQNDLNQWKTQYSRKEEAIRKIESEREKSEREKNSLRSEIERLQAEIKRIEERCRRKLEDSTRETQSQLETERSRYQREIDKLRQRPYGSHRETQTECEWTVDTSKLVFDGLRKKVTAMQLYECQLIDKTTLDKLLKGKKSVEEVASEIQPFLRGAGSIAGASASPKEKYSLVEAKRKKLISPESTVMLLEAQAATGGIIDPHRNEKLTVDSAIARDLIDFDDRQQIYAAEKAITGFDDPFSGKTVSVSEAIKKNLIDRETGMRLLEAQIASGGVVDPVNSVFLPKDVALARGLIDRDLYRSLNDPRDSQKNFVDPVTKKKVSYVQLKERCRIEPHTGLLLLSVQKRSMSFQGIRQPVTVTELVDSGILRPSTVNELESGQISYDEVGERIKDFLQGSSCIAGIYNETTKQKLGIYEAMKIGLVRPGTALELLEAQAATGFIVDPVSNLRLPVEEAYKRGLVGIEFKEKLLSAERAVTGYNDPETGNIISLFQAMNKELIEKGHGIRLLEAQIATGGIIDPKESHRLPVDIAYKRGYFNEELSEILSDPSDDTKGFFDPNTEENLTYLQLKERCIKDEETGLCLLPLKEKKKQVQTSQKNTLRKRRVVIVDPETNKEMSVQEAYKKGLIDYETFKELCEQECEWEEITITGSDGSTRVVLVDRKTGSQYDIQDAIDKGLVDRKFFDQYRSGSLSLTQFADMISLKNGVGTSSSMGSGVSDDVFSSSRHESVSKISTISSVRNLTIRSSSFSDTLEESSPIAAIFDTENLEKISITEGIERGIVDSITGQRLLEAQACTGGIIHPTTGQKLSLQDAVSQGVIDQDMATRLKPAQKAFIGFEGVKGKKKMSAAEAVKEKWLPYEAGQRFLEFQYLTGGLVDPEVHGRISTEEAIRKGFIDGRAAQRLQDTSSYAKILTCPKTKLKISYKDAINRSMVEDITGLRLLEAASVSSKGLPSPYNMSSAPGSRSGSRSGSRSGSRSGSRSGSRRGSFDATGNSSYSYSYSFSSSSIGH

6) >gi|118572613|ref|NP_057417.3| serine/arginine repetitive matrix protein 2 [Homo sapiens] (299.4 kD)

MYNGIGLPTPRGSGTNGYVQRNLSLVRGRRGERPDYKGEEELRRLEAALVKRPNPDILDHERKRRVELRCLELEEMMEEQGYEEQQIQEKVATFRLMLLEKDVNPGGKEETPGQRPAVTETHQLAELNEKKNERLRAAFGISDSYVDGSSFDPQRRAREAKQPAPEPPKPYSLVRESSSSRSPTPKQKKKKKKKDRGRRSESSSPRRERKKSSKKKKHRSESESKKRKHRSPTPKSKRKSKDKKRKRSRSTTPAPKSRRAHRSTSADSASSSDTSRSRSRSAAAKTHTTALAGRSPSPASGRRGEGDAPFSEPGTTSTQRPSSPETATKQPSSPYEDKDKDKKEKSATRPSPSPERSSTGPEPPAPTPLLAERHGGSPQPLATTPLSQEPVNPPSEASPTRDRSPPKSPEKLPQSSSSESSPPSPQPTKVSRHASSSPESPKPAPAPGSHREISSSPTSKNRSHGRAKRDKSHSHTPSRRMGRSRSPATAKRGRSRSRTPTKRGHSRSRSPQWRRSRSAQRWGRSRSPQRRGRSRSPQRPGWSRSRNTQRRGRSRSARRGRSHSRSPATRGRSRSRTPARRGRSRSRTPARRRSRSRTPTRRRSRSRTPARRGRSRSRTPARRRSRTRSPVRRRSRSRSPARRSGRSRSRTPARRGRSRSRTPARRGRSRSRTPARRSGRSRSRTPARRGRSRSRTPRRGRSRSRSLVRRGRSHSRTPQRRGRSGSSSERKNKSRTSQRRSRSNSSPEMKKSRISSRRSRSLSSPRSKAKSRLSLRRSLSGSSPCPKQKSQTPPRRSRSGSSQPKAKSRTPPRRSRSSSSPPPKQKSKTPSRQSHSSSSPHPKVKSGTPPRQGSITSPQANEQSVTPQRRSCFESSPDPELKSRTPSRHSCSGSSPPRVKSSTPPRQSPSRSSSPQPKVKAIISPRQRSHSGSSSPSPSRVTSRTTPRRSRSVSPCSNVESRLLPRYSHSGSSSPDTKVKPETPPRQSHSGSISPYPKVKAQTPPGPSLSGSKSPCPQEKSKDSLVQSCPGSLSLCAGVKSSTPPGESYFGVSSLQLKGQSQTSPDHRSDTSSPEVRQSHSESPSLQSKSQTSPKGGRSRSSSPVTELASRSPIRQDRGEFSASPMLKSGMSPEQSRFQSDSSSYPTVDSNSLLGQSRLETAESKEKMALPPQEDATASPPRQKDKFSPFPVQDRPESSLVFKDTLRTPPRERSGAGSSPETKEQNSALPTSSQDEELMEVVEKSEEPAGQILSHLSSELKEMSTSNFESSPEVEERPAVSLTLDQSQSQASLEAVEVPSMASSWGGPHFSPEHKELSNSPLRENSFGSPLEFRNSGPLGTEMNTGFSSEVKEDLNGPFLNQLETDPSLDMKEQSTRSSGHSSSELSPDAVEKAGMSSNQSISSPVLDAVPRTPSRERSSSASSPEMKDGLPRTPSRRSRSGSSPGLRDGSGTPSRHSLSGSSPGMKDIPRTPSRGRSECDSSPEPKALPQTPRPRSRSPSSPELNNKCLTPQRERSGSESSVDQKTVARTPLGQRSRSGSSQELDVKPSASPQERSESDSSPDSKAKTRTPLRQRSRSGSSPEVDSKSRLSPRRSRSGSSPEVKDKPRAAPRAQSGSDSSPEPKAPAPRALPRRSRSGSSSKGRGPSPEGSSSTESSPEHPPKSRTARRGSRSSPEPKTKSRTPPRRRSSRSSPELTRKARLSRRSRSASSSPETRSRTPPRHRRSPSVSSPEPAEKSRSSRRRRSASSPRTKTTSRRGRSPSPKPRGLQRSRSRSRREKTRTTRRRDRSGSSQSTSRRRQRSRSRSRVTRRRRGGSGYHSRSPARQESSRTSSRRRRGRSRTPPTSRKRSRSRTSPAPWKRSRSRASPATHRRSRSRTPLISRRRSRSRTSPVSRRRSRSRTSVTRRRSRSRASPVSRRRSRSRTPPVTRRRSRSRTPTTRRRSRSRTPPVTRRRSRSRTPPVTRRRSRSRTSPITRRRSRSRTSPVTRRRSRSRTSPVTRRRSRSRTSPVTRRRSRSRTPPAIRRRSRSRTPLLPRKRSRSRSPLAIRRRSRSRTPRTARGKRSLTRSPPAIRRRSASGSSSDRSRSATPPATRNHSGSRTPPVALNSSRMSCFSRPSMSPTPLDRCRSPGMLEPLGSSRTPMSVLQQAGGSMMDGPGPRIPDHQRTSVPENHAQSRIALALTAISLGTARPPPSMSAAGLAARMSQVPAPVPLMSLRTAPAANLASRIPAASAAAMNLASARTPAIPTAVNLADSRTPAAAAAMNLASPRTAVAPSAVNLADPRTPTAPAVNLAGARTPAALAALSLTGSGTPPTAANYPSSSRTPQAPASANLVGPRSAHATAPVNIAGSRTAAALAPASLTSARMAPALSGANLTSPRVPLSAYERVSGRTSPPLLDRARSRTPPSAPSQSRMTSERAPSPSSRMGQAPSQSLLPPAQDQPRSPVPSAFSDQSRCLIAQTTPVAGSQSLSSGAVATTTSSAGDHNGMLSVPAPGVPHSDVGEPPASTGAQQPSALAALQPAKERRSSSSSSSSSSSSSSSSSSSSSSSSSGSSSSDSEGSSLPVQPEVALKRVPSPTPAPKEAVREGRPPEPTPAKRKRRSSSSSSSSSSSSSSSSSSSSSSSSSSSSSSSSSSSSSSSSSSPSPAKPGPQALPKPASPKKPPPGERRSRSPRKPIDSLRDSRSLSYSPVERRRPSPQPSPRDQQSSSSERGSRRGQRGDSRSPSHKRRRETPSPRPMRHRSSRSP

7) >gi|54607053|ref|NP_006827.1| translational activator GCN1 [Homo sapiens] (292.6 kD)

MAADTQVSETLKRFAGKVTTASVKERREILSELGKCVAGKDLPEGAVKGLCKLFCLTLHRYRDAASRRALQAAIQQLAEAQPEATAKNLLHSLQSSGIGSKAGVPSKSSGSAALLALTWTCLLVRIVFPSRAKRQGDIWNKLVEVQCLLLLEVLGGSHKHAVDGAVKKLTKLWKENPGLVEQYLSAILSLEPNQNYAGMLGLLVQFCTSHKEMDVVSQHKSALLDFYMKNILMSKVKPPKYLLDSCAPLLRYLSHSEFKDLILPTIQKSLLRSPENVIETISSLLASVTLDLSQYAMDIVKGLAGHLKSNSPRLMDEAVLALRNLARQCSDSSAMESLTKHLFAILGGSEGKLTVVAQKMSVLSGIGSVSHHVVSGPSSQVLNGIVAELFIPFLQQEVHEGTLVHAVSVLALWCNRFTMEVPKKLTEWFKKAFSLKTSTSAVRHAYLQCMLASYRGDTLLQALDLLPLLIQTVEKAASQSTQVPTITEGVAAALLLLKLSVADSQAEAKLSSFWQLIVDEKKQVFTSEKFLVMASEDALCTVLHLTERLFLDHPHRLTGNKVQQYHRALVAVLLSRTWHVRRQAQQTVRKLLSSLGGFKLAHGLLEELKTVLSSHKVLPLEALVTDAGEVTEAGKAYVPPRVLQEALCVISGVPGLKGDVTDTEQLAQEMLIISHHPSLVAVQSGLWPALLARMKIDPEAFITRHLDQIIPRMTTQSPLNQSSMNAMGSLSVLSPDRVLPQLISTITASVQNPALRLVTREEFAIMQTPAGELYDKSIIQSAQQDSIKKANMKRENKAYSFKEQIIELELKEEIKKKKGIKEEVQLTSKQKEMLQAQLDREAQVRRRLQELDGELEAALGLLDIILAKNPSGLTQYIPVLVDSFLPLLKSPLAAPRIKNPFLSLAACVMPSRLKALGTLVSHVTLRLLKPECVLDKSWCQEELSVAVKRAVMLLHTHTITSRVGKGEPGAAPLSAPAFSLVFPFLKMVLTEMPHHSEEEEEWMAQILQILTVQAQLRASPNTPPGRVDENGPELLPRVAMLRLLTWVIGTGSPRLQVLASDTLTTLCASSSGDDGCAFAEQEEVDVLLCALQSPCASVRETVLRGLMELHMVLPAPDTDEKNGLNLLRRLWVVKFDKEEEIRKLAERLWSMMGLDLQPDLCSLLIDDVIYHEAAVRQAGAEALSQAVARYQRQAAEVMGRLMEIYQEKLYRPPPVLDALGRVISESPPDQWEARCGLALALNKLSQYLDSSQVKPLFQFFVPDALNDRHPDVRKCMLDAALATLNTHGKENVNSLLPVFEEFLKNAPNDASYDAVRQSVVVLMGSLAKHLDKSDPKVKPIVAKLIAALSTPSQQVQESVASCLPPLVPAIKEDAGGMIQRLMQQLLESDKYAERKGAAYGLAGLVKGLGILSLKQQEMMAALTDAIQDKKNFRRREGALFAFEMLCTMLGKLFEPYVVHVLPHLLLCFGDGNQYVREAADDCAKAVMSNLSAHGVKLVLPSLLAALEEESWRTKAGSVELLGAMAYCAPKQLSSCLPNIVPKLTEVLTDSHVKVQKAGQQALRQIGSVIRNPEILAIAPVLLDALTDPSRKTQKCLQTLLDTKFVHFIDAPSLALIMPIVQRAFQDRSTDTRKMAAQIIGNMYSLTDQKDLAPYLPSVTPGLKASLLDPVPEVRTVSAKALGAMVKGMGESCFEDLLPWLMETLTYEQSSVDRSGAAQGLAEVMAGLGVEKLEKLMPEIVATASKVDIAPHVRDGYIMMFNYLPITFGDKFTPYVGPIIPCILKALADENEFVRDTALRAGQRVISMYAETAIALLLPQLEQGLFDDLWRIRFSSVQLLGDLLFHISGVTGKMTTETASEDDNFGTAQSNKAIITALGVERRNRVLAGLYMGRSDTQLVVRQASLHVWKIVVSNTPRTLREILPTLFGLLLGFLASTCADKRTIAARTLGDLVRKLGEKILPEIIPILEEGLRSQKSDERQGVCIGLSEIMKSTSRDAVLYFSESLVPTARKALCDPLEEVREAAAKTFEQLHSTIGHQALEDILPFLLKQLDDEEVSEFALDGLKQVMAIKSRVVLPYLVPKLTTPPVNTRVLAFLSSVAGDALTRHLGVILPAVMLALKEKLGTPDEQLEMANCQAVILSVEDDTGHRIIIEDLLEATRSPEVGMRQAAAIILNIYCSRSKADYTSHLRSLVSGLIRLFNDSSPVVLEESWDALNAITKKLDAGNQLALIEELHKEIRLIGNESKGEHVPGFCLPKKGVTSILPVLREGVLTGSPEQKEEAAKALGLVIRLTSADALRPSVVSITGPLIRILGDRFSWNVKAALLETLSLLLAKVGIALKPFLPQLQTTFTKALQDSNRGVRLKAADALGKLISIHIKVDPLFTELLNGIRAMEDPGVRDTMLQALRFVIQGAGAKVDAVIRKNIVSLLLSMLGHDEDNTRISSAGCLGELCAFLTEEELSAVLQQCLLADVSGIDWMVRHGRSLALSVAVNVAPGRLCAGRYSSDVQEMILSSATADRIPIAVSGVRGMGFLMRHHIETGGGQLPAKLSSLFVKCLQNPSSDIRLVAEKMIWWANKDPLPPLDPQAIKPILKALLDNTKDKNTVVRAYSDQAIVNLLKMRQGEEVFQSLSKILDVASLEVLNEVNRRSLKKLASQADSTEQVDDTILT

8) >gi|114155142|ref|NP_003283.2| nucleoprotein TPR [Homo sapiens] (267.1 kD)

MAAVLQQVLERTELNKLPKSVQNKLEKFLADQQSEIDGLKGRHEKFKVESEQQYFEIEKRLSHSQERLVNETRECQSLRLELEKLNNQLKALTEKNKELEIAQDRNIAIQSQFTRTKEELEAEKRDLIRTNERLSQELEYLTEDVKRLNEKLKESNTTKGELQLKLDELQASDVSVKYREKRLEQEKELLHSQNTWLNTELKTKTDELLALGREKGNEILELKCNLENKKEEVSRLEEQMNGLKTSNEHLQKHVEDLLTKLKEAKEQQASMEEKFHNELNAHIKLSNLYKSAADDSEAKSNELTRAVEELHKLLKEAGEANKAIQDHLLEVEQSKDQMEKEMLEKIGRLEKELENANDLLSATKRKGAILSEEELAAMSPTAAAVAKIVKPGMKLTELYNAYVETQDQLLLEKLENKRINKYLDEIVKEVEAKAPILKRQREEYERAQKAVASLSVKLEQAMKEIQRLQEDTDKANKQSSVLERDNRRMEIQVKDLSQQIRVLLMELEEARGNHVIRDEEVSSADISSSSEVISQHLVSYRNIEELQQQNQRLLVALRELGETREREEQETTSSKITELQLKLESALTELEQLRKSRQHQMQLVDSIVRQRDMYRILLSQTTGVAIPLHASSLDDVSLASTPKRPSTSQTVSTPAPVPVIESTEAIEAKAALKQLQEIFENYKKEKAENEKIQNEQLEKLQEQVTDLRSQNTKISTQLDFASKRYEMLQDNVEGYRREITSLHERNQKLTATTQKQEQIINTMTQDLRGANEKLAVAEVRAENLKKEKEMLKLSEVRLSQQRESLLAEQRGQNLLLTNLQTIQGILERSETETKQRLSSQIEKLEHEISHLKKKLENEVEQRHTLTRNLDVQLLDTKRQLDTETNLHLNTKELLKNAQKEIATLKQHLSNMEVQVASQSSQRTGKGQPSNKEDVDDLVSQLRQTEEQVNDLKERLKTSTSNVEQYQAMVTSLEESLNKEKQVTEEVRKNIEVRLKESAEFQTQLEKKLMEVEKEKQELQDDKRRAIESMEQQLSELKKTLSSVQNEVQEALQRASTALSNEQQARRDCQEQAKIAVEAQNKYERELMLHAADVEALQAAKEQVSKMASVRQHLEETTQKAESQLLECKASWEERERMLKDEVSKCVCRCEDLEKQNRLLHDQIEKLSDKVVASVKEGVQGPLNVSLSEEGKSQEQILEILRFIRREKEIAETRFEVAQVESLRYRQRVELLERELQELQDSLNAEREKVQVTAKTMAQHEELMKKTETMNVVMETNKMLREEKERLEQDLQQMQAKVRKLELDILPLQEANAELSEKSGMLQAEKKLLEEDVKRWKARNQHLVSQQKDPDTEEYRKLLSEKEVHTKRIQQLTEEIGRLKAEIARSNASLTNNQNLIQSLKEDLNKVRTEKETIQKDLDAKIIDIQEKVKTITQVKKIGRRYKTQYEELKAQQDKVMETSAQSSGDHQEQHVSVQEMQELKETLNQAETKSKSLESQVENLQKTLSEKETEARNLQEQTVQLQSELSRLRQDLQDRTTQEEQLRQQITEKEEKTRKAIVAAKSKIAHLAGVKDQLTKENEELKQRNGALDQQKDELDVRITALKSQYEGRISRLERELREHQERHLEQRDEPQEPSNKVPEQQRQITLKTTPASGERGIASTSDPPTANIKPTPVVSTPSKVTAAAMAGNKSTPRASIRPMVTPATVTNPTTTPTATVMPTTQVESQEAMQSEGPVEHVPVFGSTSGSVRSTSPNVQPSISQPILTVQQQTQATAFVQPTQQSHPQIEPANQELSSNIVEVVQSSPVERPSTSTAVFGTVSATPSSSLPKRTREEEEDSTIEASDQVSDDTVEMPLPKKLKSVTPVGTEEEVMAEESTDGEVETQVYNQDSQDSIGEGVTQGDYTPMEDSEETSQSLQIDLGPLQSDQQTTTSSQDGQGKGDDVIVIDSDDEEEDDDENDGEHEDYEEDEEDDDDDEDDTGMGDEGEDSNEGTGSADGNDGYEADDAEGGDGTDPGTETEESMGGGEGNHRAADSQNSGEGNTGAAESSFSQEVSREQQPSSASERQAPRAPQSPRRPPHPLPPRLTIHAPPQELGPPVQRIQMTRRQSVGRGLQLTPGIGGMQQHFFDDEDRTVPSTPTLVVPHRTDGFAEAIHSPQVAGVPRFRFGPPEDMPQTSSSHSDLGQLASQGGLGMYETPLFLAHEEESGGRSVPTTPLQVAAPVTVFTESTTSDASEHASQSVPMVTTSTGTLSTTNETATGDDGDEVFVEAESEGISSEAGLEIDSQQEEEPVQASDESDLPSTSQDPPSSSSVDTSSSQPKPFRRVRLQTTLRQGVRGRQFNRQRGVSHAMGGRGGINRGNIN

9) >gi|62122917|ref|NP_001014364.1| filaggrin-2 [Homo sapiens] (247.9 kD)

MTDLLRSVVTVIDVFYKYTKQDGECGTLSKGELKELLEKELHPVLKNPDDPDTVDVIMHMLDRDHDRRLDFTEFLLMIFKLTMACNKVLSKEYCKASGSKKHRRGHRHQEEESETEEDEEDTPGHKSGYRHSSWSEGEEHGYSSGHSRGTVKCRHGSNSRRLGRQGNLSSSGNQEGSQKRYHRSSCGHSWSGGKDRHGSSSVELRERINKSHISPSRESGEEYESGSGSNSWERKGHGGLSCGLETSGHESNSTQSRIREQKLGSSCSGSGDSGRRSHACGYSNSSGCGRPQNASSSCQSHRFGGQGNQFSYIQSGCQSGIKGGQGHGCVSGGQPSGCGQPESNPCSQSYSQRGYGARENGQPQNCGGQWRTGSSQSSCCGQYGSGGSQSCSNGQHEYGSCGRFSNSSSSNEFSKCDQYGSGSSQSTSFEQHGTGLSQSSGFEQHVCGSGQTCGQHESTSSQSLGYDQHGSSSGKTSGFGQHGSGSGQSSGFGQCGSGSGQSSGFGQHGSVSGQSSGFGQHGSVSGQSSGFGQHESRSRQSSYGQHGSGSSQSSGYGQYGSRETSGFGQHGLGSGQSTGFGQYGSGSGQSSGFGQHGSGSGQSSGFGQHESRSGQSSYGQHSSGSSQSSGYGQHGSRQTSGFGQHGSGSSQSTGFGQYGSGSGQSSGFGQHVSGSGQSSGFGQHESRSGHSSYGQHGFGSSQSSGYGQHGSSSGQTSGFGQHELSSGQSSSFGQHGSGSGQSSGFGQHGSGSGQSSGFGQHESRSGQSSYGQHSSGSSQSSGYGQHGSRQTSGFGQHGSGSSQSTGFGQYGSGSGQSAGFGQHGSGSGQSSGFGQHESRSHQSSYGQHGSGSSQSSGYGQHGSSSGQTSGFGQHRSSSGQYSGFGQHGSGSGQSSGFGQHGTGSGQYSGFGQHESRSHQSSYGQHGSGSSQSSGYGQHGSSSGQTFGFGQHRSGSGQSSGFGQHGSGSGQSSGFGQHESGSGKSSGFGQHESRSSQSNYGQHGSGSSQSSGYGQHGSSSGQTTGFGQHRSSSGQYSGFGQHGSGSDQSSGFGQHGTGSGQSSGFGQYESRSRQSSYGQHGSGSSQSSGYGQHGSNSGQTSGFGQHRPGSGQSSGFGQYGSGSGQSSGFGQHGSGTGKSSGFAQHEYRSGQSSYGQHGTGSSQSSGCGQHESGSGPTTSFGQHVSGSDNFSSSGQHISDSGQSTGFGQYGSGSGQSTGLGQGESQQVESGSTVHGRQETTHGQTINTTRHSQSGQGQSTQTGSRVTRRRRSSQSENSDSEVHSKVSHRHSEHIHTQAGSHYPKSGSTVRRRQGTTHGQRGDTTRHGHSGHGQSTQTGSRTSGRQRFSHSDATDSEVHSGVSHRPHSQEQTHSQAGSQHGESESTVHERHETTYGQTGEATGHGHSGHGQSTQRGSRTTGRRGSGHSESSDSEVHSGGSHRPQSQEQTHGQAGSQHGESGSTVHGRHGTTHGQTGDTTRHAHYHHGKSTQRGSSTTGRRGSGHSESSDSEVHSGGSHTHSGHTHGQSGSQHGESESIIHDRHRITHGQTGDTTRHSYSGHEQTTQTGSRTTGRQRTSHSESTDSEVHSGGSHRPHSREHTYGQAGSQHEEPEFTVHERHGTTHGQIGDTTGHSHSGHGQSTQRGSRTTGRQRSSHSESSDSEVHSGVSHTHTGHTHGQAGSQHGQSESIVPERHGTTHGQTGDTTRHAHYHHGLTTQTGSRTTGRRGSGHSEYSDSEGYSGVSHTHSGHTHGQARSQHGESESIVHERHGTIHGQTGDTTRHAHSGHGQSTQTGSRTTGRRSSGHSEYSDSEGHSGFSQRPHSRGHTHGQAGSQHGESESIVDERHGTTHGQTGDTSGHSQSGHGQSTQSGSSTTGRRRSGHSESSDSEVHSGGSHTHSGHTHSQARSQHGESESTVHKRHQTTHGQTGDTTEHGHPSHGQTIQTGSRTTGRRGSGHSEYSDSEGPSGVSHTHSGHTHGQAGSHYPESGSSVHERHGTTHGQTADTTRHGHSGHGQSTQRGSRTTGRRASGHSEYSDSEGHSGVSHTHSGHAHGQAGSQHGESGSSVHERHGTTHGQTGDTTRHAHSGHGQSTQRGSRTAGRRGSGHSESSDSEVHSGVSHTHSGHTYGQARSQHGESGSAIHGRQGTIHGQTGDTTRHGQSGHGQSTQTGSRTTGRQRSSHSESSDSEVHSEASPTHSGHTHSQAGSRHGQSGSSGHGRQGTTHGQTGDTTRHAHYGYGQSTQRGSRTTGRRGSGHSESSDSEVHSWGSHTHSGHIQGQAGSQQRQPGSTVHGRLETTHGQTGDTTRHGHSGYGQSTQTGSRSSRASHFQSHSSERQRHGSSQVWKHGSYGPAEYDYGHTGYGPSGGSRKSISNSHLSWSTDSTANKQLSRH

10) >gi|367460087|ref|NP_005955.3| myosin-10 isoform 2 [Homo sapiens] (228.9 kD)

MAQRTGLEDPERYLFVDRAVIYNPATQADWTAKKLVWIPSERHGFEAASIKEERGDEVMVELAENGKKAMVNKDDIQKMNPPKFSKVEDMAELTCLNEASVLHNLKDRYYSGLIYTYSGLFCVVINPYKNLPIYSENIIEMYRGKKRHEMPPHIYAISESAYRCMLQDREDQSILCTGESGAGKTENTKKVIQYLAHVASSHKGRKDHNIPGELERQLLQANPILESFGNAKTVKNDNSSRFGKFIRINFDVTGYIVGANIETYLLEKSRAVRQAKDERTFHIFYQLLSGAGEHLKSDLLLEGFNNYRFLSNGYIPIPGQQDKDNFQETMEAMHIMGFSHEEILSMLKVVSSVLQFGNISFKKERNTDQASMPENTVAQKLCHLLGMNVMEFTRAILTPRIKVGRDYVQKAQTKEQADFAVEALAKATYERLFRWLVHRINKALDRTKRQGASFIGILDIAGFEIFELNSFEQLCINYTNEKLQQLFNHTMFILEQEEYQREGIEWNFIDFGLDLQPCIDLIERPANPPGVLALLDEECWFPKATDKTFVEKLVQEQGSHSKFQKPRQLKDKADFCIIHYAGKVDYKADEWLMKNMDPLNDNVATLLHQSSDRFVAELWKDVDRIVGLDQVTGMTETAFGSAYKTKKGMFRTVGQLYKESLTKLMATLRNTNPNFVRCIIPNHEKRAGKLDPHLVLDQLRCNGVLEGIRICRQGFPNRIVFQEFRQRYEILTPNAIPKGFMDGKQACERMIRALELDPNLYRIGQSKIFFRAGVLAHLEEERDLKITDIIIFFQAVCRGYLARKAFAKKQQQLSALKVLQRNCAAYLKLRHWQWWRVFTKVKPLLQVTRQEEELQAKDEELLKVKEKQTKVEGELEEMERKHQQLLEEKNILAEQLQAETELFAEAEEMRARLAAKKQELEEILHDLESRVEEEEERNQILQNEKKKMQAHIQDLEEQLDEEEGARQKLQLEKVTAEAKIKKMEEEILLLEDQNSKFIKEKKLMEDRIAECSSQLAEEEEKAKNLAKIRNKQEVMISDLEERLKKEEKTRQELEKAKRKLDGETTDLQDQIAELQAQIDELKLQLAKKEEELQGALARGDDETLHKNNALKVVRELQAQIAELQEDFESEKASRNKAEKQKRDLSEELEALKTELEDTLDTTAAQQELRTKREQEVAELKKALEEETKNHEAQIQDMRQRHATALEELSEQLEQAKRFKANLEKNKQGLETDNKELACEVKVLQQVKAESEHKRKKLDAQVQELHAKVSEGDRLRVELAEKASKLQNELDNVSTLLEEAEKKGIKFAKDAASLESQLQDTQELLQEETRQKLNLSSRIRQLEEEKNSLQEQQEEEEEARKNLEKQVLALQSQLADTKKKVDDDLGTIESLEEAKKKLLKDAEALSQRLEEKALAYDKLEKTKNRLQQELDDLTVDLDHQRQVASNLEKKQKKFDQLLAEEKSISARYAEERDRAEAEAREKETKALSLARALEEALEAKEEFERQNKQLRADMEDLMSSKDDVGKNVHELEKSKRALEQQVEEMRTQLEELEDELQATEDAKLRLEVNMQAMKAQFERDLQTRDEQNEEKKRLLIKQVRELEAELEDERKQRALAVASKKKMEIDLKDLEAQIEAANKARDEVIKQLRKLQAQMKDYQRELEEARASRDEIFAQSKESEKKLKSLEAEILQLQEELASSERARRHAEQERDELADEITNSASGKSALLDEKRRLEARIAQLEEELEEEQSNMELLNDRFRKTTLQVDTLNAELAAERSAAQKSDNARQQLERQNKELKAKLQELEGAVKSKFKATISALEAKIGQLEEQLEQEAKERAAANKLVRRTEKKLKEIFMQVEDERRHADQYKEQMEKANARMKQLKRQLEEAEEEATRANASRRKLQRELDDATEANEGLSREVSTLKNRLRRGGPISFSSSRSGRRQLHLEGASLELSDDDTESKTSDVNETQPPQSE

11) >gi|57164942|ref|NP_001008938.1| cytoskeleton-associated protein 5 isoform a [Homo sapiens] (225.4 kD)

MGDDSEWLKLPVDQKCEHKLWKARLSGYEEALKIFQKIKDEKSPEWSKFLGLIKKFVTDSNAVVQLKGLEAALVYVENAHVAGKTTGEVVSGVVSKVFNQPKAKAKELGIEICLMYIEIEKGEAVQEELLKGLDNKNPKIIVACIETLRKALSEFGSKIILLKPIIKVLPKLFESREKAVRDEAKLIAVEIYRWIRDALRPPLQNINSVQLKELEEEWVKLPTSAPRPTRFLRSQQELEAKLEQQQSAGGDAEGGGDDGDEVPQIDAYELLEAVEILSKLPKDFYDKIEAKKWQERKEALESVEVLIKNPKLEAGDYADLVKALKKVVGKDTNVMLVALAAKCLTGLAVGLRKKFGQYAGHVVPTILEKFKEKKPQVVQALQEAIDAIFLTTTLQNISEDVLAVMDNKNPTIKQQTSLFIARSFRHCTASTLPKSLLKPFCAALLKHINDSAPEVRDAAFEALGTALKVVGEKAVNPFLADVDKLKLDKIKECSEKVELIHGKKAGLAADKKEFKPLPGRTAASGAAGDKDTKDISAPKPGPLKKAPAAKAGGPPKKGKPAAPGGAGNTGTKNKKGLETKEIVEPELSIEVCEEKASAVLPPTCIQLLDSSNWKERLACMEEFQKAVELMDRTEMPCQALVRMLAKKPGWKETNFQVMQMKLHIVALIAQKGNFSKTSAQVVLDGLVDKIGDVKCGNNAKEAMTAIAEACMLPWTAEQVVSMAFSQKNPKNQSETLNWLSNAIKEFGFSGLNVKAFISNVKTALAATNPAVRTAAITLLGVMYLYVGPSLRMFFEDEKPALLSQIDAEFEKMQGQSPPAPTRGISKHSTSGTDEGEDGDEPDDGSNDVVDLLPRTEISDKITSELVSKIGDKNWKIRKEGLDEVAGIINDAKFIQPNIGELPTALKGRLNDSNKILVQQTLNILQQLAVAMGPNIKQHVKNLGIPIITVLGDSKNNVRAAALATVNAWAEQTGMKEWLEGEDLSEELKKENPFLRQELLGWLAEKLPTLRSTPTDLILCVPHLYSCLEDRNGDVRKKAQDALPFFMMHLGYEKMAKATGKLKPTSKDQVLAMLEKAKVNMPAKPAPPTKATSKPMGGSAPAKFQPASAPAEDCISSSTEPKPDPKKAKAPGLSSKAKSAQGKKMPSKTSLKEDEDKSGPIFIVVPNGKEQRMKDEKGLKVLKWNFTTPRDEYIEQLKTQMSSCVAKWLQDEMFHSDFQHHNKALAVMVDHLESEKEGVIGCLDLILKWLTLRFFDTNTSVLMKALEYLKLLFTLLSEEEYHLTENEASSFIPYLVVKVGEPKDVIRKDVRAILNRMCLVYPASKMFPFIMEGTKSKNSKQRAECLEELGCLVESYGMNVCQPTPGKALKEIAVHIGDRDNAVRNAALNTIVTVYNVHGDQVFKLIGNLSEKDMSMLEERIKRSAKRPSAAPIKQVEEKPQRAQNISSNANMLRKGPAEDMSSKLNQARSMSGHPEAAQMVRREFQLDLDEIENDNGTVRCEMPELVQHKLDDIFEPVLIPEPKIRAVSPHFDDMHSNTASTINFIISQVASGDINTSIQALTQIDEVLRQEDKAEAMSGHIDQFLIATFMQLRLIYNTHMADEKLEKDEIIKLYSCIIGNMISLFQIESLAREASTGVLKDLMHGLITLMLDSRIEDLEEGQQVIRSVNLLVVKVLEKSDQTNILSALLVLLQDSLLATASSPKFSELVMKCLWRMVRLLPDTINSINLDRILLDIHIFMKVFPKEKLKQCKSEFPIRTLKTLLHTLCKLKGPKILDHLTMIDNKNESELEAHLCRMMKHSMDQTGSKSDKETEKGASRIDEKSSKAKVNDFLAEIFKKIGSKENTKEGLAELYEYKKKYSDADIEPFLKNSSQFFQSYVERGLRVIEMEREGKGRISTSTGISPQMEVTCVPTPTSTVSSIGNTNGEEVGPSVYLERLKILRQRCGLDNTKQDDRPPLTSLLSKPAVPTVASSTDMLHSKLSQLRESREQHQHSDLDSNQTHSSGTVTSSSSTANIDDLKKRLERIKSSRK

12) >gi|302699237|ref|NP_886553.3| eukaryotic translation initiation factor 4 gamma 1 isoform 1 [Homo sapiens] (175.4 kD)

MNKAPQSTGPPPAPSPGLPQPAFPPGQTAPVVFSTPQATQMNTPSQPRQHFYPSRAQPPSSAASRVQSAAPARPGPAAHVYPAGSQVMMIPSQISYPASQGAYYIPGQGRSTYVVPTQQYPVQPGAPGFYPGASPTEFGTYAGAYYPAQGVQQFPTGVAPAPVLMNQPPQIAPKRERKTIRIRDPNQGGKDITEEIMSGARTASTPTPPQTGGGLEPQANGETPQVAVIVRPDDRSQGAIIADRPGLPGPEHSPSESQPSSPSPTPSPSPVLEPGSEPNLAVLSIPGDTMTTIQMSVEESTPISRETGEPYRLSPEPTPLAEPILEVEVTLSKPVPESEFSSSPLQAPTPLASHTVEIHEPNGMVPSEDLEPEVESSPELAPPPACPSESPVPIAPTAQPEELLNGAPSPPAVDLSPVSEPEEQAKEVTASMAPPTIPSATPATAPSATSPAQEEEMEEEEEEEEGEAGEAGEAESEKGGEELLPPESTPIPANLSQNLEAAAATQVAVSVPKRRRKIKELNKKEAVGDLLDAFKEANPAVPEVENQPPAGSNPGPESEGSGVPPRPEEADETWDSKEDKIHNAENIQPGEQKYEYKSDQWKPLNLEEKKRYDREFLLGFQFIFASMQKPEGLPHISDVVLDKANKTPLRPLDPTRLQGINCGPDFTPSFANLGRTTLSTRGPPRGGPGGELPRGPQAGLGPRRSQQGPRKEPRKIIATVLMTEDIKLNKAEKAWKPSSKRTAADKDRGEEDADGSKTQDLFRRVRSILNKLTPQMFQQLMKQVTQLAIDTEERLKGVIDLIFEKAISEPNFSVAYANMCRCLMALKVPTTEKPTVTVNFRKLLLNRCQKEFEKDKDDDEVFEKKQKEMDEAATAEERGRLKEELEEARDIARRRSLGNIKFIGELFKLKMLTEAIMHDCVVKLLKNHDEESLECLCRLLTTIGKDLDFEKAKPRMDQYFNQMEKIIKEKKTSSRIRFMLQDVLDLRGSNWVPRRGDQGPKTIDQIHKEAEMEEHREHIKVQQLMAKGSDKRRGGPPGPPISRGLPLVDDGGWNTVPISKGSRPIDTSRLTKITKPGSIDSNNQLFAPGGRLSWGKGSSGGSGAKPSDAASEAARPATSTLNRFSALQQAVPTESTDNRRVVQRSSLSRERGEKAGDRGDRLERSERGGDRGDRLDRARTPATKRSFSKEVEERSRERPSQPEGLRKAASLTEDRDRGRDAVKREAALPPVSPLKAALSEEELEKKSKAIIEEYLHLNDMKEAVQCVQELASPSLLFIFVRHGVESTLERSAIAREHMGQLLHQLLCAGHLSTAQYYQGLYEILELAEDMEIDIPHVWLYLAELVTPILQEGGVPMGELFREITKPLRPLGKAASLLLEILGLLCKSMGPKKVGTLWREAGLSWKEFLPEGQDIGAFVAEQKVEYTLGEESEAPGQRALPSEELNRQLEKLLKEGSSNQRVFDWIEANLSEQQIVSNTLVRALMTAVCYSAIIFETPLRVDVAVLKARAKLLQKYLCDEQKELQALYALQALVVTLEQPPNLLRMFFDALYDEDVVKEDAFYSWESSKDPAEQQGKGVALKSVTAFFKWLREAEEESDHN

13) >gi|355390315|ref|NP_056171.3| protein scribble homolog isoform b [Homo sapiens] (174.8 kD)

MLKCIPLWRCNRHVESVDKRHCSLQAVPEEIYRYSRSLEELLLDANQLRELPKPFFRLLNLRKLGLSDNEIQRLPPEVANFMQLVELDVSRNDIPEIPESIKFCKALEIADFSGNPLSRLPDGFTQLRSLAHLALNDVSLQALPGDVGNLANLVTLELRENLLKSLPASLSFLVKLEQLDLGGNDLEVLPDTLGALPNLRELWLDRNQLSALPPELGNLRRLVCLDVSENRLEELPAELGGLVLLTDLLLSQNLLRRLPDGIGQLKQLSILKVDQNRLCEVTEAIGDCENLSELILTENLLMALPRSLGKLTKLTNLNVDRNHLEALPPEIGGCVALSVLSLRDNRLAVLPPELAHTTELHVLDVAGNRLQSLPFALTHLNLKALWLAENQAQPMLRFQTEDDARTGEKVLTCYLLPQQPPPSLEDAGQQGSLSETWSDAPPSRVSVIQFLEAPIGDEDAEEAAAEKRGLQRRATPHPSELKVMKRSIEGRRSEACPCQPDSGSPLPAEEEKRLSAESGLSEDSRPSASTVSEAEPEGPSAEAQGGSQQEATTAGGEEDAEEDYQEPTVHFAEDALLPGDDREIEEGQPEAPWTLPGGRQRLIRKDTPHYKKHFKISKLPQPEAVVALLQGMQPDGEGPVAPGGWHNGPHAPWAPRAQKEEEEEEEGSPQEEEEEEEEENRAEEEEASTEEEDKEGAVVSAPSVKGVSFDQANNLLIEPARIEEEELTLTILRQTGGLGISIAGGKGSTPYKGDDEGIFISRVSEEGPAARAGVRVGDKLLEVNGVALQGAEHHEAVEALRGAGTAVQMRVWRERMVEPENAVTITPLRPEDDYSPRERRGGGLRLPLLPPESPGPLRQRHVACLARSERGLGFSIAGGKGSTPYRAGDAGIFVSRIAEGGAAHRAGTLQVGDRVLSINGVDVTEARHDHAVSLLTAASPTIALLLEREAGGPLPPSPLPHSSPPTAAVATTSITTATPGVPGLPSLAPSLLAAALEGPYPVEEIRLPRAGGPLGLSIVGGSDHSSHPFGVQEPGVFISKVLPRGLAARSGLRVGDRILAVNGQDVRDATHQEAVSALLRPCLELSLLVRRDPAPPGLRELCIQKAPGERLGISIRGGARGHAGNPRDPTDEGIFISKVSPTGAAGRDGRLRVGLRLLEVNQQSLLGLTHGEAVQLLRSVGDTLTVLVCDGFEASTDAALEVSPGVIANPFAAGIGHRNSLESISSIDRELSPEGPGKEKELPGQTLHWGPEATEAAGRGLQPLKLDYRALAAVPSAGSVQRVPSGAAGGKMAESPCSPSGQQPPSPPSPDELPANVKQAYRAFAAVPTSHPPEDAPAQPPTPGPAASPEQLSFRERQKYFELEVRVPQAEGPPKRVSLVGADDLRKMQEEEARKLQQKRAQMLREAAEAGAEARLALDGETLGEEEQEDEQPPWASPSPTSRQSPASPPPLGGGAPVRTAKAERRHQERLRVQSPEPPAPERALSPAELRALEAEKRALWRAARMKSLEQDALRAQMVLSRSQEGRGTRGPLERLAEAPSPAPTPSPTPVEDLGPQTSTSPGRLSPDFAEELRSLEPSPSPGPQEEDGEVALVLLGRPSPGAVGPEDVALCSSRRPVRPGRRGLGPVPS

14) >gi|156766068|ref|NP_059995.2| serine/threonine-protein kinase MRCK gamma [Homo sapiens] (172.4 kD)

MERRLRALEQLARGEAGGCPGLDGLLDLLLALHHELSSGPLRRERSVAQFLSWASPFVSKVKELRLQRDDFEILKVIGRGAFGEVTVVRQRDTGQIFAMKMLHKWEMLKRAETACFREERDVLVKGDSRWVTTLHYAFQDEEYLYLVMDYYAGGDLLTLLSRFEDRLPPELAQFYLAEMVLAIHSLHQLGYVHRDVKPDNVLLDVNGHIRLADFGSCLRLNTNGMVDSSVAVGTPDYISPEILQAMEEGKGHYGPQCDWWSLGVCAYELLFGETPFYAESLVETYGKIMNHEDHLQFPPDVPDVPASAQDLIRQLLCRQEERLGRGGLDDFRNHPFFEGVDWERLASSTAPYIPELRGPMDTSNFDVDDDTLNHPGTLPPPSHGAFSGHHLPFVGFTYTSGSHSPESSSEAWAALERKLQCLEQEKVELSRKHQEALHAPTDHRELEQLRKEVQTLRDRLPEMLRDKASLSQTDGPPAGSPGQDSDLRQELDRLHRELAEGRAGLQAQEQELCRAQGQQEELLQRLQEAQEREAATASQTRALSSQLEEARAAQRELEAQVSSLSRQVTQLQGQWEQRLEESSQAKTIHTASETNGMGPPEGGPQEAQLRKEVAALREQLEQAHSHRPSGKEEALCQLQEENRRLSREQERLEAELAQEQESKQRLEGERRETESNWEAQLADILSWVNDEKVSRGYLQALATKMAEELESLRNVGTQTLPARPLDHQWKARRLQKMEASARLELQSALEAEIRAKQGLQERLTQVQEAQLQAERRLQEAEKQSQALQQELAMLREELRARGPVDTKPSNSLIPFLSFRSSEKDSAKDPGISGEATRHGGEPDLRPEGRRSLRMGAVFPRAPTANTASTEGLPAKPGSHTLRPRSFPSPTKCLRCTSLMLGLGRQGLGCDACGYFCHTTCAPQAPPCPVPPDLLRTALGVHPETGTGTAYEGFLSVPRPSGVRRGWQRVFAALSDSRLLLFDAPDLRLSPPSGALLQVLDLRDPQFSATPVLASDVIHAQSRDLPRIFRVTTSQLAVPPTTCTVLLLAESEGERERWLQVLGELQRLLLDARPRPRPVYTLKEAYDNGLPLLPHTLCAAILDQDRLALGTEEGLFVIHLRSNDIFQVGECRRVQQLTLSPSAGLLVVLCGRGPSVRLFALAELENIEVAGAKIPESRGCQVLAAGSILQARTPVLCVAVKRQVLCYQLGPGPGPWQRRIRELQAPATVQSLGLLGDRLCVGAAGGFALYPLLNEAAPLALGAGLVPEELPPSRGGLGEALGAVELSLSEFLLLFTTAGIYVDGAGRKSRGHELLWPAAPMGWGYAAPYLTVFSENSIDVFDVRRAEWVQTVPLKKVRPLNPEGSLFLYGTEKVRLTYLRNQLAEKDEFDIPDLTDNSRRQLFRTKSKRRFFFRVSEEQQKQQRREMLKDPFVRSKLISPPTNFNHLVHVGPANGRPGARDKSPAPEEKGRVARGSGPQRPHSFSEALRRPASMGSEGLGGDADPMKRKPWTSLSSESVSCPQGSLSPATSLMQVSERPRSLPLSPELESSP

15) >gi|358679311|ref|NP_001240626.1| protein LAP2 isoform 1 [Homo sapiens] (158.2 kD)

MTTKRSLFVRLVPCRCLRGEEETVTTLDYSHCSLEQVPKEIFTFEKTLEELYLDANQIEELPKQLFNCQSLHKLSLPDNDLTTLPASIANLINLRELDVSKNGIQEFPENIKNCKVLTIVEASVNPISKLPDGFSQLLNLTQLYLNDAFLEFLPANFGRLTKLQILELRENQLKMLPKTMNRLTQLERLDLGSNEFTEVPEVLEQLSGLKEFWMDANRLTFIPGFIGSLKQLTYLDVSKNNIEMVEEGISTCENLQDLLLSSNSLQQLPETIGSLKNITTLKIDENQLMYLPDSIGGLISVEELDCSFNEVEALPSSIGQLTNLRTFAADHNYLQQLPPEIGSWKNITVLFLHSNKLETLPEEMGDMQKLKVINLSDNRLKNLPFSFTKLQQLTAMWLSDNQSKPLIPLQKETDSETQKMVLTNYMFPQQPRTEDVMFISDNESFNPSLWEEQRKQRAQVAFECDEDKDEREAPPREGNLKRYPTPYPDELKNMVKTVQTIVHRLKDEETNEDSGRDLKPHEDQQDINKDVGVKTSESTTTVKSKVDEREKYMIGNSVQKISEPEAEISPGSLPVTANMKASENLKHIVNHDDVFEESEELSSDEEMKMAEMRPPLIETSINQPKVVALSNNKKDDTKETDSLSDEVTHNSNQNNSNCSSPSRMSDSVSLNTDSSQDTSLCSPVKQTHIDINSKIRQEDENFNSLLQNGDILNSSTEEKFKAHDKKDFNLPEYDLNVEERLVLIEKSVDSTATADDTHKLDHINMNLNKLITNDTFQPEIMERSKTQDIVLGTSFLSINSKEETEHLENGNKYPNLESVNKVNGHSEETSQSPNRTEPHDSDCSVDLGISKSTEDLSPQKSGPVGSVVKSHSITNMEIGGLKIYDILSDNGPQQPSTTVKITSAVDGKNIVRSKSATLLYDQPLQVFTGSSSSSDLISGTKAIFKFDSNHNPEEPNIIRGPTSGPQSAPQIYGPPQYNIQYSSSAAVKDTLWHSKQNPQIDHASFPPQLLPRSESTENQSYAKHSANMNFSNHNNVRANTAYHLHQRLGPARHGEMWAISPNDRLIPAVTRSTIQRQSSVSSTASVNLGDPGSTRRAQIPEGDYLSYREFHSAGRTPPMMPGSQRPLSARTYSIDGPNASRPQSARPSINEIPERTMSVSDFNYSRTSPSKRPNARVGSEHSLLDPPGKSKVPRDWREQVLRHIEAKKLEKKHPQTSSSGDPCQDGIFISGQQNYSSATLSHKDVPPDSLMKMPLSNGQMGQPLRPQANYSQIHHPPQASVARHPSREQLIDYLMLKVAHQPPYTQPHCSPRQGHELAKQEIRVRVEKDPELGFSISGGVGGRGNPFRPDDDGIFVTRVQPEGPASKLLQPGDKIIQANGYSFINIEHGQAVSLLKTFQNTVELIIVREVSS

16) >gi|31621305|ref|NP_573566.2| leucine-rich PPR motif-containing protein, mitochondrial precursor [Homo sapiens] (157.8 kD)

MAALLRSARWLLRAGAAPRLPLSLRLLPGGPGRLHAASYLPAARAGPVAGGLLSPARLYAIAAKEKDIQEESTFSSRKISNQFDWALMRLDLSVRRTGRIPKKLLQKVFNDTCRSGGLGGSHALLLLRSCGSLLPELKLEERTEFAHRIWDTLQKLGAVYDVSHYNALLKVYLQNEYKFSPTDFLAKMEEANIQPNRVTYQRLIASYCNVGDIEGASKILGFMKTKDLPVTEAVFSALVTGHARAGDMENAENILTVMRDAGIEPGPDTYLALLNAYAEKGDIDHVKQTLEKVEKSELHLMDRDLLQIIFSFSKAGYPQYVSEILEKVTCERRYIPDAMNLILLLVTEKLEDVALQILLACPVSKEDGPSVFGSFFLQHCVTMNTPVEKLTDYCKKLKEVQMHSFPLQFTLHCALLANKTDLAKALMKAVKEEGFPIRPHYFWPLLVGRRKEKNVQGIIEILKGMQELGVHPDQETYTDYVIPCFDSVNSARAILQENGCLSDSDMFSQAGLRSEAANGNLDFVLSFLKSNTLPISLQSIRSSLLLGFRRSMNINLWSEITELLYKDGRYCQEPRGPTEAVGYFLYNLIDSMSDSEVQAKEEHLRQYFHQLEKMNVKIPENIYRGIRNLLESYHVPELIKDAHLLVESKNLDFQKTVQLTSSELESTLETLKAENQPIRDVLKQLILVLCSEENMQKALELKAKYESDMVTGGYAALINLCCRHDKVEDALNLKEEFDRLDSSAVLDTGKYVGLVRVLAKHGKLQDAINILKEMKEKDVLIKDTTALSFFHMLNGAALRGEIETVKQLHEAIVTLGLAEPSTNISFPLVTVHLEKGDLSTALEVAIDCYEKYKVLPRIHDVLCKLVEKGETDLIQKAMDFVSQEQGEMVMLYDLFFAFLQTGNYKEAKKIIETPGIRARSARLQWFCDRCVANNQVETLEKLVELTQKLFECDRDQMYYNLLKLYKINGDWQRADAVWNKIQEENVIPREKTLRLLAEILREGNQEVPFDVPELWYEDEKHSLNSSSASTTEPDFQKDILIACRLNQKKGAYDIFLNAKEQNIVFNAETYSNLIKLLMSEDYFTQAMEVKAFAETHIKGFTLNDAANSRLIITQVRRDYLKEAVTTLKTVLDQQQTPSRLAVTRVIQALAMKGDVENIEVVQKMLNGLEDSIGLSKMVFINNIALAQIKNNNIDAAIENIENMLTSENKVIEPQYFGLAYLFRKVIEEQLEPAVEKISIMAERLANQFAIYKPVTDFFLQLVDAGKVDDARALLQRCGAIAEQTPILLLFLLRNSRKQGKASTVKSVLELIPELNEKEEAYNSLMKSYVSEKDVTSAKALYEHLTAKNTKLDDLFLKRYASLLKYAGEPVPFIEPPESFEFYAQQLRKLRENSS

17) >gi|148536853|ref|NP_004362.2| coatomer subunit alpha isoform 2 [Homo sapiens] (138.3 kD)

MLTKFETKSARVKGLSFHPKRPWILTSLHNGVIQLWDYRMCTLIDKFDEHDGPVRGIDFHKQQPLFVSGGDDYKIKVWNYKLRRCLFTLLGHLDYIRTTFFHHEYPWILSASDDQTIRVWNWQSRTCVCVLTGHNHYVMCAQFHPTEDLVVSASLDQTVRVWDISGLRKKNLSPGAVESDVRGITGVDLFGTTDAVVKHVLEGHDRGVNWAAFHPTMPLIVSGADDRQVKIWRMNESKAWEVDTCRGHYNNVSCAVFHPRQELILSNSEDKSIRVWDMSKRTGVQTFRRDHDRFWVLAAHPNLNLFAAGHDGGMIVFKLERERPAYAVHGNMLHYVKDRFLRQLDFNSSKDVAVMQLRSGSKFPVFNMSYNPAENAVLLCTRASNLENSTYDLYTIPKDADSQNPDAPEGKRSSGLTAVWVARNRFAVLDRMHSLLIKNLKNEITKKVQVPNCDEIFYAGTGNLLLRDADSITLFDVQQKRTLASVKISKVKYVIWSADMSHVALLAKHAIVICNRKLDALCNIHENIRVKSGAWDESGVFIYTTSNHIKYAVTTGDHGIIRTLDLPIYVTRVKGNNVYCLDRECRPRVLTIDPTEFKFKLALINRKYDEVLHMVRNAKLVGQSIIAYLQKKGYPEVALHFVKDEKTRFSLALECGNIEIALEAAKALDDKNCWEKLGEVALLQGNHQIVEMCYQRTKNFDKLSFLYLITGNLEKLRKMMKIAEIRKDMSGHYQNALYLGDVSERVRILKNCGQKSLAYLTAATHGLDEEAESLKETFDPEKETIPDIDPNAKLLQPPAPIMPLDTNWPLLTVSKGFFEGTIASKGKGGALAADIDIDTVGTEGWGEDAELQLDEDGFVEATEGLGDDALGKGQEEGGGWDVEEDLELPPELDISPGAAGGAEDGFFVPPTKGTSPTQIWCNNSQLPVDHILAGSFETAMRLLHDQVGVIQFGPYKQLFLQTYARGRTTYQALPCLPSMYGYPNRNWKDAGLKNGVPAVGLKLNDLIQRLQLCYQLTTVGKFEEAVEKFRSILLSVPLLVVDNKQEIAEAQQLITICREYIVGLSVETERKKLPKETLEQQKRICEMAAYFTHSNLQPVHMILVLRTALNLFFKLKNFKTAATFARRLLELGPKPEVAQQTRKILSACEKNPTDAYQLNYDMHNPFDICAASYRPIYRGKPVEKCPLSGACYSPEFKGQICRVTTVTEIGKDVIGLRISPLQFR

18) >gi|21361794|ref|NP_060918.2| cullin-associated NEDD8-dissociated protein 1 [Homo sapiens] (136.3 kD)

MASASYHISNLLEKMTSSDKDFRFMATNDLMTELQKDSIKLDDDSERKVVKMILKLLEDKNGEVQNLAVKCLGPLVSKVKEYQVETIVDTLCTNMLSDKEQLRDISSIGLKTVIGELPPASSGSALAANVCKKITGRLTSAIAKQEDVSVQLEALDIMADMLSRQGGLLVNFHPSILTCLLPQLTSPRLAVRKRTIIALGHLVMSCGNIVFVDLIEHLLSELSKNDSMSTTRTYIQCIAAISRQAGHRIGEYLEKIIPLVVKFCNVDDDELREYCIQAFESFVRRCPKEVYPHVSTIINICLKYLTYDPNYNYDDEDEDENAMDADGGDDDDQGSDDEYSDDDDMSWKVRRAAAKCLDAVVSTRHEMLPEFYKTVSPALISRFKEREENVKADVFHAYLSLLKQTRPVQSWLCDPDAMEQGETPLTMLQSQVPNIVKALHKQMKEKSVKTRQCCFNMLTELVNVLPGALTQHIPVLVPGIIFSLNDKSSSSNLKIDALSCLYVILCNHSPQVFHPHVQALVPPVVACVGDPFYKITSEALLVTQQLVKVIRPLDQPSSFDATPYIKDLFTCTIKRLKAADIDQEVKERAISCMGQIICNLGDNLGSDLPNTLQIFLERLKNEITRLTTVKALTLIAGSPLKIDLRPVLGEGVPILASFLRKNQRALKLGTLSALDILIKNYSDSLTAAMIDAVLDELPPLISESDMHVSQMAISFLTTLAKVYPSSLSKISGSILNELIGLVRSPLLQGGALSAMLDFFQALVVTGTNNLGYMDLLRMLTGPVYSQSTALTHKQSYYSIAKCVAALTRACPKEGPAVVGQFIQDVKNSRSTDSIRLLALLSLGEVGHHIDLSGQLELKSVILEAFSSPSEEVKSAASYALGSISVGNLPEYLPFVLQEITSQPKRQYLLLHSLKEIISSASVVGLKPYVENIWALLLKHCECAEEGTRNVVAECLGKLTLIDPETLLPRLKGYLISGSSYARSSVVTAVKFTISDHPQPIDPLLKNCIGDFLKTLEDPDLNVRRVALVTFNSAAHNKPSLIRDLLDTVLPHLYNETKVRKELIREVEMGPFKHTVDDGLDIRKAAFECMYTLLDSCLDRLDIFEFLNHVEDGLKDHYDIKMLTFLMLVRLSTLCPSAVLQRLDRLVEPLRATCTTKVKANSVKQEFEKQDELKRSAMRAVAALLTIPEAEKSPLMSEFQSQISSNPELAAIFESIQKDSSSTNLESMDTS

19) >gi|41349439|ref|NP_055748.2| protein transport protein Sec31A isoform 1 [Homo sapiens] (132.9 kD)

MKLKEVDRTAMQAWSPAQNHPIYLATGTSAQQLDATFSTNASLEIFELDLSDPSLDMKSCATFSSSHRYHKLIWGPYKMDSKGDVSGVLIAGGENGNIILYDPSKIIAGDKEVVIAQNDKHTGPVRALDVNIFQTNLVASGANESEIYIWDLNNFATPMTPGAKTQPPEDISCIAWNRQVQHILASASPSGRATVWDLRKNEPIIKVSDHSNRMHCSGLAWHPDVATQMVLASEDDRLPVIQMWDLRFASSPLRVLENHARGILAIAWSMADPELLLSCGKDAKILCSNPNTGEVLYELPTNTQWCFDIQWCPRNPAVLSAASFDGRISVYSIMGGSTDGLRQKQVDKLSSSFGNLDPFGTGQPLPPLQIPQQTAQHSIVLPLKKPPKWIRRPVGASFSFGGKLVTFENVRMPSHQGAEQQQQQHHVFISQVVTEKEFLSRSDQLQQAVQSQGFINYCQKKIDASQTEFEKNVWSFLKVNFEDDSRGKYLELLGYRKEDLGKKIALALNKVDGANVALKDSDQVAQSDGEESPAAEEQLLGEHIKEEKEESEFLPSSGGTFNISVSGDIDGLITQALLTGNFESAVDLCLHDNRMADAIILAIAGGQELLARTQKKYFAKSQSKITRLITAVVMKNWKEIVESCDLKNWREALAAVLTYAKPDEFSALCDLLGTRLENEGDSLLQTQACLCYICAGNVEKLVACWTKAQDGSHPLSLQDLIEKVVILRKAVQLTQAMDTSTVGVLLAAKMSQYANLLAAQGSIAAALAFLPDNTNQPNIMQLRDRLCRAQGEPVAGHESPKIPYEKQQLPKGRPGPVAGHHQMPRVQTQQYYPHGENPPPPGFIMHGNVNPNAAGQLPTSPGHMHTQVPPYPQPQPYQPAQPYPFGTGGSAMYRPQQPVAPPTSNAYPNTPYISSASSYTGQSQLYAAQHQASSPTSSPATSFPPPPSSGASFQHGGPGAPPSSSAYALPPGTTGTLPAASELPASQRTGPQNGWNDPPALNRVPKKKKMPENFMPPVPITSPIMNPLGDPQSQMLQQQPSAPVPLSSQSSFPQPHLPGGQPFHGVQQPLGQTGMPPSFSKPNIEGAPGAPIGNTFQHVQSLPTKKITKKPIPDEHLILKTTFEDLIQRCLSSATDPQTKRKLDDASKRLEFLYDKLREQTLSPTITSGLHNIARSIETRNYSEGLTMHTHIVSTSNFSETSAFMPVLKVVLTQANKLGV

20) >gi|109659845|ref|NP_878913.2| filamin A-interacting protein 1-like isoform 1 [Homo sapiens] (130.3 kD)

MRSRGSDTEGSAQKKFPRHTKGHSFQGPKNMKHRQQDKDSPSESDVILPCPKAEKPHSGNGHQAEDLSRDDLLFLLSILEGELQARDEVIGILKAEKMDLALLEAQYGFVTPKKVLEALQRDAFQAKSTPWQEDIYEKPMNELDKVVEKHKESYRRILGQLLVAEKSRRQTILELEEEKRKHKEYMEKSDEFICLLEQECERLKKLIDQEIKSQEEKEQEKEKRVTTLKEELTKLKSFALMVVDEQQRLTAQLTLQRQKIQELTTNAKETHTKLALAEARVQEEEQKATRLEKELQTQTTKFHQDQDTIMAKLTNEDSQNRQLQQKLAALSRQIDELEETNRSLRKAEEELQDIKEKISKGEYGNAGIMAEVEELRKRVLDMEGKDEELIKMEEQCRDLNKRLERETLQSKDFKLEVEKLSKRIMALEKLEDAFNKSKQECYSLKCNLEKERMTTKQLSQELESLKVRIKELEAIESRLEKTEFTLKEDLTKLKTLTVMFVDERKTMSEKLKKTEDKLQAASSQLQVEQNKVTTVTEKLIEETKRALKSKTDVEEKMYSVTKERDDLKNKLKAEEEKGNDLLSRVNMLKNRLQSLEAIEKDFLKNKLNQDSGKSTTALHQENNKIKELSQEVERLKLKLKDMKAIEDDLMKTEDEYETLERRYANERDKAQFLSKELEHVKMELAKYKLAEKTETSHEQWLFKRLQEEEAKSGHLSREVDALKEKIHEYMATEDLICHLQGDHSVLQKKLNQQENRNRDLGREIENLTKELERYRHFSKSLRPSLNGRRISDPQVFSKEVQTEAVDNEPPDYKSLIPLERAVINGQLYEESENQDEDPNDEGSVLSFKCSQSTPCPVNRKLWIPWMKSKEGHLQNGKMQTKPNANFVQPGDLVLSHTPGQPLHIKVTPDHVQNTATLEITSPTTESPHSYTSTAVIPNCGTPKQRITILQNASITPVKSKTSTEDLMNLEQGMSPITMATFARAQTPESCGSLTPERTMSPIQVLAVTGSASSPEQGRSPEPTEISAKHAIFRVSPDRQSSWQFQRSNSNSSSVITTEDNKIHIHLGSPYMQAVASPVRPASPSAPLQDNRTQGLINGALNKTTNKVTSSITITPTATPLPRQSQITVEPLLLPH

21) >gi|42475558|ref|NP_775179.1| protein SMG7 isoform 1 [Homo sapiens] (127.2 kD)

MSLQSAQYLRQAEVLKADMTDSKLGPAEVWTSRQALQDLYQKMLVTDLEYALDKKVEQDLWNHAFKNQITTLQGQAKNRANPNRSEVQANLSLFLEAASGFYTQLLQELCTVFNVDLPCRVKSSQLGIISNKQTHTSAIVKPQSSSCSYICQHCLVHLGDIARYRNQTSQAESYYRHAAQLVPSNGQPYNQLAILASSKGDHLTTIFYYCRSIAVKFPFPAASTNLQKALSKALESRDEVKTKWGVSDFIKAFIKFHGHVYLSKSLEKLSPLREKLEEQFKRLLFQKAFNSQQLVHVTVINLFQLHHLRDFSNETEQHTYSQDEQLCWTQLLALFMSFLGILCKCPLQNESQEESYNAYPLPAVKVSMDWLRLRPRVFQEAVVDERQYIWPWLISLLNSFHPHEEDLSSISATPLPEEFELQGFLALRPSFRNLDFSKGHQGITGDKEGQQRRIRQQRLISIGKWIADNQPRLIQCENEVGKLLFITEIPELILEDPSEAKENLILQETSVIESLAADGSPGLKSVLSTSRNLSNNCDTGEKPVVTFKENIKTREVNRDQGRSFPPKEVRRDYSKGITVTKNDGKKDNNKRKTETKKCTLEKLQETGKQNVAVQVKSQTELRKTPVSEARKTPVTQTPTQASNSQFIPIHHPGAFPPLPSRPGFPPPTYVIPPPVAFSMGSGYTFPAGVSVPGTFLQPTAHSPAGNQVQAGKQSHIPYSQQRPSGPGPMNQGPQQSQPPSQQPLTSLPAQPTAQSTSQLQVQALTQQQQSPTKAVPALGKSPPHHSGFQQYQQADASKQLWNPPQVQGPLGKIMPVKQPYYLQTQDPIKLFEPSLQPPVMQQQPLEKKMKPFPMEPYNHNPSEVKVPEFYWDSSYSMADNRSVMAQQANIDRRGKRSPGVFRPEQDPVPRMPFEKSLLEKPSELMSHSSSFLSLTGFSLNQERYPNNSMFNEVYGKNLTSSSKAELSPSMAPQETSLYSLFEGTPWSPSLPASSDHSTPASQSPHSSNPSSLPSSPPTHNHNSVPFSNFGPIGTPDNRDRRTADRWKTDKPAMGGFGIDYLSATSSSESSWHQASTPSGTWTGHGPSMEDSSAVLMESLKSIWSSSMMHPGPSALEQLLMQQKQKQQRGQGTMNPPH

22) >gi|21327715|ref|NP_006697.2| transcription elongation regulator 1 isoform 1 [Homo sapiens] (123.8 kD)

MAERGGDGGESERFNPGELRMAQQQALRFRGPAPPPNAVMRGPPPLMRPPPPFGMMRGPPPPPRPPFGRPPFDPNMPPMPPPGGIPPPMGPPHLQRPPFMPPPMSSMPPPPGMMFPPGMPPVTAPGTPALPPTEEIWVENKTPDGKVYYYNARTRESAWTKPDGVKVIQQSELTPMLAAQAQVQAQAQAQAQAQAQAQAQAQAQAQAQAQAQAQAQAQAQAQAQAQAQAQAQAQAQAQAQAQVQAQVQAQVQAQAVGASTPTTSSPAPAVSTSTSSSTPSSTTSTTTTATSVAQTVSTPTTQDQTPSSAVSVATPTVSVSTPAPTATPVQTVPQPHPQTLPPAVPHSVPQPTTAIPAFPPVMVPPFRVPLPGMPIPLPGVAMMQIVSCPYVKTVATTKTGVLPGMAPPIVPMIHPQVAIAASPATLAGATAVSEWTEYKTADGKTYYYNNRTLESTWEKPQELKEKEKLEEKIKEPIKEPSEEPLPMETEEEDPKEEPIKEIKEEPKEEEMTEEEKAAQKAKPVATAPIPGTPWCVVWTGDERVFFYNPTTRLSMWDRPDDLIGRADVDKIIQEPPHKKGMEELKKLRHPTPTMLSIQKWQFSMSAIKEEQELMEEINEDEPVKAKKRKRDDNKDIDSEKEAAMEAEIKAARERAIVPLEARMKQFKDMLLERGVSAFSTWEKELHKIVFDPRYLLLNPKERKQVFDQYVKTRAEEERREKKNKIMQAKEDFKKMMEEAKFNPRATFSEFAAKHAKDSRFKAIEKMKDREALFNEFVAAARKKEKEDSKTRGEKIKSDFFELLSNHHLDSQSRWSKVKDKVESDPRYKAVDSSSMREDLFKQYIEKIAKNLDSEKEKELERQARIEASLREREREVQKARSEQTKEIDREREQHKREEAIQNFKALLSDMVRSSDVSWSDTRRTLRKDHRWESGSLLEREEKEKLFNEHIEALTKKKREHFRQLLDETSAITLTSTWKEVKKIIKEDPRCIKFSSSDRKKQREFEEYIRDKYITAKADFRTLLKETKFITYRSKKLIQESDQHLKDVEKILQNDKRYLVLDCVPEERRKLIVAYVDDLDRRGPPPPPTASEPTRRSTK

23) >gi|14149680|ref|NP_056107.1| extended synaptotagmin-1 isoform 2 [Homo sapiens] (122.8 kD)

MERSPGEGPSPSPMDQPSAPSDPTDQPPAAHAKPDPGSGGQPAGPGAAGEALAVLTSFGRRLLVLIPVYLAGAVGLSVGFVLFGLALYLGWRRVRDEKERSLRAARQLLDDEEQLTAKTLYMSHRELPAWVSFPDVEKAEWLNKIVAQVWPFLGQYMEKLLAETVAPAVRGSNPHLQTFTFTRVELGEKPLRIIGVKVHPGQRKEQILLDLNISYVGDVQIDVEVKKYFCKAGVKGMQLHGVLRVILEPLIGDLPFVGAVSMFFIRRPTLDINWTGMTNLLDIPGLSSLSDTMIMDSIAAFLVLPNRLLVPLVPDLQDVAQLRSPLPRGIIRIHLLAARGLSSKDKYVKGLIEGKSDPYALVRLGTQTFCSRVIDEELNPQWGETYEVMVHEVPGQEIEVEVFDKDPDKDDFLGRMKLDVGKVLQASVLDDWFPLQGGQGQVHLRLEWLSLLSDAEKLEQVLQWNWGVSSRPDPPSAAILVVYLDRAQDLPLKKGNKEPNPMVQLSIQDVTQESKAVYSTNCPVWEEAFRFFLQDPQSQELDVQVKDDSRALTLGALTLPLARLLTAPELILDQWFQLSSSGPNSRLYMKLVMRILYLDSSEICFPTVPGCPGAWDVDSENPQRGSSVDAPPRPCHTTPDSQFGTEHVLRIHVLEAQDLIAKDRFLGGLVKGKSDPYVKLKLAGRSFRSHVVREDLNPRWNEVFEVIVTSVPGQELEVEVFDKDLDKDDFLGRCKVRLTTVLNSGFLDEWLTLEDVPSGRLHLRLERLTPRPTAAELEEVLQVNSLIQTQKSAELAAALLSIYMERAEDLPLRKGTKHLSPYATLTVGDSSHKTKTISQTSAPVWDESASFLIRKPHTESLELQVRGEGTGVLGSLSLPLSELLVADQLCLDRWFTLSSGQGQVLLRAQLGILVSQHSGVEAHSHSYSHSSSSLSEEPELSGGPPHITSSAPELRQRLTHVDSPLEAPAGPLGQVKLTLWYYSEERKLVSIVHGCRSLRQNGRDPPDPYVSLLLLPDKNRGTKRRTSQKKRTLSPEFNERFEWELPLDEAQRRKLDVSVKSNSSFMSRERELLGKVQLDLAETDLSQGVARWYDLMDNKDKGSS

24) >gi|530412286|ref|XP_005257452.1| PREDICTED: ATP-citrate synthase isoform X3 [Homo sapiens] (120.8 kD)

MSAKAISEQTGKELLYKFICTTSAIQNRFKYARVTPDTDWARLLQDHPWLLSQNLVVKPDQLIKRRGKLGLVGVNLTLDGVKSWLKPRLGQEATVGKATGFLKNFLIEPFVPHSQAEEFYVCIYATREGDYVLFHHEGGVDVGDVDAKAQKLLVGVDEKLNPEDIKKHLLVHAPEDKKEILASFISGLFNFYEDLYFTYLEINPLVVTKDGVYVLDLAAKVDATADYICKVKWGDIEFPPPFGREAYPEEAYIADLDAKSGASLKLTLLNPKGRIWTMVAGGGASVVYSDTICDLGGVNELANYGEYSGAPSEQQTYDYAKTILSLMTREKHPDGKILIIGGSIANFTNVAATFKGIVRAIRDYQGPLKEHEVTIFVRRGGPNYQEGLRVMGEVGKTTGIPIHVFGTETHMTAIVGMALGHRPIPNQPPTAAHTANFLLNASGSTSTPAPSRTASFSESRADEVAPAKKAKPAMPQDSVPSPRSLQGKSTTLFSRHTKAIVWGMQTRAVQGMLDFDYVCSRDEPSVAAMVYPFTGDHKQKFYWGHKEILIPVFKNMADAMRKHPEVDVLINFASLRSAYDSTMETMNYAQIRTIAIIAEGIPEALTRKLIKKADQKGVTIIGPATVGGIKPGCFKIGNTGGMLDNILASKLYRPGSVAYVSRSGGMSNELNNIISRTTDGVYEGVAIGGDRYPGSTFMDHVLRYQDTPGVKMIVVLGEIGGTEEYKICRGIKEGRLTKPIVCWCIGTCATMFSSEVQFGHAGACANQASETAVAKNQALKEAGVFVPRSFDELGEIIQSVYEDLVANGVIVPAQEVPPPTVPMDYSWARELGLIRKPASFMTSICDERGQELIYAGMPITEVFKEEMGIGGVLGLLWFQKRLPKYSCQFIEMCLMVTADHGPAVSGAHNTIICARAGKDLVSSLTSGLLTIGDRFGGALDAAAKMFSKAFDSGIIPMEFVNKMKKEGKLIMGIGHRVKSINNPDMRVQILKDYVRQHFPATPLLDYALEVEKITTSKKPNLILNVDGLIGVAFVDMLRNCGSFTREEADEYIDIGALNGIFVLGRSMGFIGHYLDQKRLKQGLYRHPWDDISYVLPEHMSM

25) >gi|62460637|ref|NP_078934.3| importin-4 [Homo sapiens] (118.6 kD)

MESAGLEQLLRELLLPDTERIRRATEQLQIVLRAPAALPALCDLLASAADPQIRQFAAVLTRRRLNTRWRRLAAEQRESLKSLILTALQRETEHCVSLSLAQLSATIFRKEGLEAWPQLLQLLQHSTHSPHSPEREMGLLLLSVVVTSRPEAFQPHHRELLRLLNETLGEVGSPGLLFYSLRTLTTMAPYLSTEDVPLARMLVPKLIMAMQTLIPIDEAKACEALEALDELLESEVPVITPYLSEVLTFCLEVARNVALGNAIRIRILCCLTFLVKVKSKALLKNRLLPPLLHTLFPIVAAEPPPGQLDPEDQDSEEEELEIELMGETPKHFAVQVVDMLALHLPPEKLCPQLMPMLEEALRSESPYQRKAGLLVLAVLSDGAGDHIRQRLLPPLLQIVCKGLEDPSQVVRNAALFALGQFSENLQPHISSYSREVMPLLLAYLKSVPLGHTHHLAKACYALENFVENLGPKVQPYLPELMECMLQLLRNPSSPRAKELAVSALGAIATAAQASLLPYFPAIMEHLREFLLTGREDLQPVQIQSLETLGVLARAVGEPMRPLAEECCQLGLGLCDQVDDPDLRRCTYSLFAALSGLMGEGLAPHLEQITTLMLLSLRSTEGIVPQYDGSSSFLLFDDESDGEEEEELMDEDVEEEDDSEISGYSVENAFFDEKEDTCAAVGEISVNTSVAFLPYMESVFEEVFKLLECPHLNVRKAAHEALGQFCCALHKACQSCPSEPNTAALQAALARVVPSYMQAVNRERERQVVMAVLEALTGVLRSCGTLTLKPPGRLAELCGVLKAVLQRKTACQDTDEEEEEEDDDQAEYDAMLLEHAGEAIPALAAAAGGDSFAPFFAGFLPLLVCKTKQGCTVAEKSFAVGTLAETIQGLGAASAQFVSRLLPVLLSTAQEADPEVRSNAIFGMGVLAEHGGHPAQEHFPKLLGLLFPLLARERHDRVRDNICGALARLLMASPTRKPEPQVLAALLHALPLKEDLEEWVTIGRLFSFLYQSSPDQVIDVAPELLRICSLILADNKIPPDTKAALLLLLTFLAKQHTDSFQAALGSLPVDKAQELQAVLGLS

26) >gi|41327773|ref|NP_055644.2| probable ATP-dependent RNA helicase DDX46 [Homo sapiens] (117.3 kD)

MGRESRHYRKRSASRGRSGSRSRSRSPSDKRSKRGDDRRSRSRDRDRRRERSRSRDKRRSRSRDRKRLRRSRSRERDRSRERRRSRSRDRRRSRSRSRGRRSRSSSPGNKSKKTENRSRSKEKTDGGESSKEKKKDKDDKEDEKEKDAGNFDQNKLEEEMRKRKERVEKWREEQRKKAMENIGELKKEIEEMKQGKKWSLEDDDDDEDDPAEAEKEGNEMEGEELDPLDAYMEEVKEEVKKFNMRSVKGGGGNEKKSGPTVTKVVTVVTTKKAVVDSDKKKGELMENDQDAMEYSSEEEEVDLQTALTGYQTKQRKLLEPVDHGKIEYEPFRKNFYVEVPELAKMSQEEVNVFRLEMEGITVKGKGCPKPIKSWVQCGISMKILNSLKKHGYEKPTPIQTQAIPAIMSGRDLIGIAKTGSGKTIAFLLPMFRHIMDQRSLEEGEGPIAVIMTPTRELALQITKECKKFSKTLGLRVVCVYGGTGISEQIAELKRGAEIIVCTPGRMIDMLAANSGRVTNLRRVTYVVLDEADRMFDMGFEPQVMRIVDNVRPDRQTVMFSATFPRAMEALARRILSKPIEVQVGGRSVVCSDVEQQVIVIEEEKKFLKLLELLGHYQESGSVIIFVDKQEHADGLLKDLMRASYPCMSLHGGIDQYDRDSIINDFKNGTCKLLVATSVAARGLDVKHLILVVNYSCPNHYEDYVHRAGRTGRAGNKGYAYTFITEDQARYAGDIIKALELSGTAVPPDLEKLWSDFKDQQKAEGKIIKKSSGFSGKGFKFDETEQALANERKKLQKAALGLQDSDDEDAAVDIDEQIESMFNSKKRVKDMAAPGTSSVPAPTAGNAEKLEIAKRLALRINAQKNLGIESQDVMQQATNAILRGGTILAPTVSAKTIAEQLAEKINAKLNYVPLEKQEEERQDGGQNESFKRYEEELEINDFPQTARWKVTSKEALQRISEYSEAAITIRGTYFPPGKEPKEGERKIYLAIESANELAVQKAKAEITRLIKEELIRLQNSYQPTNKGRYKVL

27) >gi|34101286|ref|NP_057191.2| zinc finger RNA-binding protein [Homo sapiens] (116.9 kD)

MIPICPVVSFTYVPSRLGEDAKMATGNYFGFTHSGAAAAAAAAQYSQQPASGVAYSHPTTVASYTVHQAPVAAHTVTAAYAPAAATVAVARPAPVAVAAAATAAAYGGYPTAHTATDYGYTQRQQEAPPPPPPATTQNYQDSYSYVRSTAPAVAYDSKQYYQQPTATAAAVAAAAQPQPSVAETYYQTAPKAGYSQGATQYTQAQQTRQVTAIKPATPSPATTTFSIYPVSSTVQPVAAAATVVPSYTQSATYSTTAVTYSGTSYSGYEAAVYSAASSYYQQQQQQQKQAAAAAAAAAATAAWTGTTFTKKAPFQNKQLKPKQPPKPPQIHYCDVCKISCAGPQTYKEHLEGQKHKKKEAALKASQNTSSSNSSTRGTQNQLRCELCDVSCTGADAYAAHIRGAKHQKVVKLHTKLGKPIPSTEPNVVSQATSSTAVSASKPTASPSSIAANNCTVNTSSVATSSMKGLTTTGNSSLNSTSNTKVSAVPTNMAAKKTSTPKINFVGGNKLQSTGNKAEDIKGTECVKSTPVTSAVQIPEVKQDTVSEPVTPASLAALQSDVQPVGHDYVEEVRNDEGKVIRFHCKLCECSFNDPNAKEMHLKGRRHRLQYKKKVNPDLQVEVKPSIRARKIQEEKMRKQMQKEEYWRRREEEERWRMEMRRYEEDMYWRRMEEEQHHWDDRRRMPDGGYPHGPPGPLGLLGVRPGMPPQPQGPAPLRRPDSSDDRYVMTKHATIYPTEEELQAVQKIVSITERALKLVSDSLSEHEKNKNKEGDDKKEGGKDRALKGVLRVGVLAKGLLLRGDRNVNLVLLCSEKPSKTLLSRIAENLPKQLAVISPEKYDIKCAVSEAAIILNSCVEPKMQVTITLTSPIIREENMREGDVTSGMVKDPPDVLDRQKCLDALAALRHAKWFQARANGLQSCVIIIRILRDLCQRVPTWSDFPSWAMELLVEKAISSASSPQSPGDALRRVFECISSGIILKGSPGLLDPCEKDPFDTLATMTDQQREDITSSAQFALRLLAFRQIHKVLGMDPLPQMSQRFNIHNNRKRRRDSDGVDGFEAEGKKDKKDYDNF

28) >gi|29029559|ref|NP_001307.2| exportin-2 isoform 1 [Homo sapiens] (110.3 kD)

MELSDANLQTLTEYLKKTLDPDPAIRRPAEKFLESVEGNQNYPLLLLTLLEKSQDNVIKVCASVTFKNYIKRNWRIVEDEPNKICEADRVAIKANIVHLMLSSPEQIQKQLSDAISIIGREDFPQKWPDLLTEMVNRFQSGDFHVINGVLRTAHSLFKRYRHEFKSNELWTEIKLVLDAFALPLTNLFKATIELCSTHANDASALRILFSSLILISKLFYSLNFQDLPEFFEDNMETWMNNFHTLLTLDNKLLQTDDEEEAGLLELLKSQICDNAALYAQKYDEEFQRYLPRFVTAIWNLLVTTGQEVKYDLLVSNAIQFLASVCERPHYKNLFEDQNTLTSICEKVIVPNMEFRAADEEAFEDNSEEYIRRDLEGSDIDTRRRAACDLVRGLCKFFEGPVTGIFSGYVNSMLQEYAKNPSVNWKHKDAAIYLVTSLASKAQTQKHGITQANELVNLTEFFVNHILPDLKSANVNEFPVLKADGIKYIMIFRNQVPKEHLLVSIPLLINHLQAESIVVHTYAAHALERLFTMRGPNNATLFTAAEIAPFVEILLTNLFKALTLPGSSENEYIMKAIMRSFSLLQEAIIPYIPTLITQLTQKLLAVSKNPSKPHFNHYMFEAICLSIRITCKANPAAVVNFEEALFLVFTEILQNDVQEFIPYVFQVMSLLLETHKNDIPSSYMALFPHLLQPVLWERTGNIPALVRLLQAFLERGSNTIASAAADKIPGLLGVFQKLIASKANDHQGFYLLNSIIEHMPPESVDQYRKQIFILLFQRLQNSKTTKFIKSFLVFINLYCIKYGALALQEIFDGIQPKMFGMVLEKIIIPEIQKVSGNVEKKICAVGITKLLTECPPMMDTEYTKLWTPLLQSLIGLFELPEDDTIPDEEHFIDIEDTPGYQTAFSQLAFAGKKEHDPVGQMVNNPKIHLAQSLHKLSTACPGRVPSMVSTSLNAEALQYLQGYLQAASVTLL

29) >gi|146231940|ref|NP_001078927.1| catenin delta-1 isoform 1ABC [Homo sapiens] (108.1 kD)

MDDSEVESTASILASVKEQEAQFEKLTRALEEERRHVSAQLERVRVSPQDANPLMANGTLTRRHQNGRFVGDADLERQKFSDLKLNGPQDHSHLLYSTIPRMQEPGQIVETYTEEDPEGAMSVVSVETSDDGTTRRTETTVKKVVKTVTTRTVQPVAMGPDGLPVDASSVSNNYIQTLGRDFRKNGNGGPGPYVGQAGTATLPRNFHYPPDGYSRHYEDGYPGGSDNYGSLSRVTRIEERYRPSMEGYRAPSRQDVYGPQPQVRVGGSSVDLHRFHPEPYGLEDDQRSMGYDDLDYGMMSDYGTARRTGTPSDPRRRLRSYEDMIGEEVPSDQYYWAPLAQHERGSLASLDSLRKGGPPPPNWRQPELPEVIAMLGFRLDAVKSNAAAYLQHLCYRNDKVKTDVRKLKGIPVLVGLLDHPKKEVHLGACGALKNISFGRDQDNKIAIKNCDGVPALVRLLRKARDMDLTEVITGTLWNLSSHDSIKMEIVDHALHALTDEVIIPHSGWEREPNEDCKPRHIEWESVLTNTAGCLRNVSSERSEARRKLRECDGLVDALIFIVQAEIGQKDSDSKLVENCVCLLRNLSYQVHREIPQAERYQEAAPNVANNTGPHAASCFGAKKGKDEWFSRGKKPIEDPANDTVDFPKRTSPARGYELLFQPEVVRIYISLLKESKTPAILEASAGAIQNLCAGRWTYGRYIRSALRQEKALSAIADLLTNEHERVVKAASGALRNLAVDARNKELIGKHAIPNLVKNLPGGQQNSSWNFSEDTVISILNTINEVIAENLEAAKKLRETQGIEKLVLINKSGNRSEKEVRAAALVLQTIWGYKELRKPLEKEGWKKSDFQVNLNNASRSQSSHSYDDSTLPLIDRNQKSDKKPDREEIQMSNMGSNTKSLDNNYSTPNERGDHNRTLDRSGDLGDMEPLKGTTPLMQDEGQESLEEELDVLVLDDEGGQVSYPSMQKI

30) >gi|530418825|ref|XP_005260998.1| PREDICTED: trifunctional purine biosynthetic protein adenosine-3 isoform X1 [Homo sapiens] (107.7 kD)

MAARVLIIGSGGREHTLAWKLAQSHHVKQVLVAPGNAGTACSEKISNTAISISDHTALAQFCKEKKIEFVVVGPEAPLAAGIVGNLRSAGVQCFGPTAEAAQLESSKRFAKEFMDRHGIPTAQWKAFTKPEEACSFILSADFPALVVKASGLAAGKGVIVAKSKEEACKAVQEIMQEKAFGAAGETIVIEELLDGEEVSCLCFTDGKTVAPMPPAQDHKRLLEGDGGPNTGGMGAYCPAPQVSNDLLLKIKDTVLQRTVDGMQQEGTPYTGILYAGIMLTKNGPKVLEFNCRFGDPECQVILPLLKSDLYEVIQSTLDGLLCTSLPVWLENHTALTVVMASKGYPGDYTKGVEITGFPEAQALGLEVFHAGTALKNGKVVTHGGRVLAVTAIRENLISALEEAKKGLAAIKFEGAIYRKDVGFRAIAFLQQPRSLTYKESGVDIAAGNMLVKKIQPLAKATSRSGCKVDLGGFAGLFDLKAAGFKDPLLASGTDGVGTKLKIAQLCNKHDTIGQDLVAMCVNDILAQGAEPLFFLDYFSCGKLDLSVTEAVVAGIAKACGKAGCALLGGETAEMPDMYPPGEYDLAGFAVGAMERDQKLPHLERITEGDVVVGIASSGLHSNGFSLVRKIVAKSSLQYSSPAPDGCGDQTLGDLLLTPTRIYSHSLLPVLRSGHVKAFAHITGGGLLENIPRVLPEKLGVDLDAQTWRIPRVFSWLQQEGHLSEEEMARTFNCGVGAVLVVSKEQTEQILRDIQQHKEEAWVIGSVVARAEGSPRVKVKNLIESMQINGSVLKNGSLTNHFSFEKKKARVAVLISGTGSNLQALIDSTREPNSSAQIDIVISNKAAVAGLDKAERAGIPTRVINHKLYKNRVEFDSAIDLVLEEFSIDIVCLAGFMRILSGPFVQKWNGKMLNIHPSLLPSFKGSNAHEQALETGVTVTGCTVHFVAEDVDAGQIILQEAVPVKRGDTVATLSERVKLAEHKIFPAALQLVASGTVQLGENGKICWVKEE

31) >gi|221316630|ref|NP_001137533.1| coatomer subunit beta [Homo sapiens] (107.1 kD)

MTAAENVCYTLINVPMDSEPPSEISLKNDLEKGDVKSKTEALKKVIIMILNGEKLPGLLMTIIRFVLPLQDHTIKKLLLVFWEIVPKTTPDGRLLHEMILVCDAYRKDLQHPNEFIRGSTLRFLCKLKEAELLEPLMPAIRACLEHRHSYVRRNAVLAIYTIYRNFEHLIPDAPELIHDFLVNEKDASCKRNAFMMLIHADQDRALDYLSTCIDQVQTFGDILQLVIVELIYKVCHANPSERARFIRCIYNLLQSSSPAVKYEAAGTLVTLSSAPTAIKAAAQCYIDLIIKESDNNVKLIVLDRLIELKEHPAHERVLQDLVMDILRVLSTPDLEVRKKTLQLALDLVSSRNVEELVIVLKKEVIKTNNVSEHEDTDKYRQLLVRTLHSCSVRFPDMAANVIPVLMEFLSDNNEAAAADVLEFVREAIQRFDNLRMLIVEKMLEVFHAIKSVKIYRGALWILGEYCSTKEDIQSVMTEIRRSLGEIPIVESEIKKEAGELKPEEEITVGPVQKLVTEMGTYATQSALSSSRPTKKEEDRPPLRGFLLDGDFFVAASLATTLTKIALRYVALVQEKKKQNSFVAEAMLLMATILHLGKSSLPKKPITDDDVDRISLCLKVLSECSPLMNDIFNKECRQSLSHMLSAKLEEEKLSQKKESEKRNVTVQPDDPISFMQLTAKNEMNCKEDQFQLSLLAAMGNTQRKEAADPLASKLNKVTQLTGFSDPVYAEAYVHVNQYDIVLDVLVVNQTSDTLQNCTLELATLGDLKLVEKPSPLTLAPHDFANIKANVKVASTENGIIFGNIVYDVSGAASDRNCVVLSDIHIDIMDYIQPATCTDAEFRQMWAEFEWENKVTVNTNMVDLNDYLQHILKSTNMKCLTPEKALSGYCGFMAANLYARSIFGEDALANVSIEKPIHQGPDAAVTGHIRIRAKSQGMALSLGDKINLSQKKTSI

32) >gi|4501891|ref|NP_001093.1| alpha-actinin-1 isoform b [Homo sapiens] (103 kD)

MDHYDSQQTNDYMQPEEDWDRDLLLDPAWEKQQRKTFTAWCNSHLRKAGTQIENIEEDFRDGLKLMLLLEVISGERLAKPERGKMRVHKISNVNKALDFIASKGVKLVSIGAEEIVDGNVKMTLGMIWTIILRFAIQDISVEETSAKEGLLLWCQRKTAPYKNVNIQNFHISWKDGLGFCALIHRHRPELIDYGKLRKDDPLTNLNTAFDVAEKYLDIPKMLDAEDIVGTARPDEKAIMTYVSSFYHAFSGAQKAETAANRICKVLAVNQENEQLMEDYEKLASDLLEWIRRTIPWLENRVPENTMHAMQQKLEDFRDYRRLHKPPKVQEKCQLEINFNTLQTKLRLSNRPAFMPSEGRMVSDINNAWGCLEQVEKGYEEWLLNEIRRLERLDHLAEKFRQKASIHEAWTDGKEAMLRQKDYETATLSEIKALLKKHEAFESDLAAHQDRVEQIAAIAQELNELDYYDSPSVNARCQKICDQWDNLGALTQKRREALERTEKLLETIDQLYLEYAKRAAPFNNWMEGAMEDLQDTFIVHTIEEIQGLTTAHEQFKATLPDADKERLAILGIHNEVSKIVQTYHVNMAGTNPYTTITPQEINGKWDHVRQLVPRRDQALTEEHARQQHNERLRKQFGAQANVIGPWIQTKMEEIGRISIEMHGTLEDQLSHLRQYEKSIVNYKPKIDQLEGDHQLIQEALIFDNKHTNYTMEHIRVGWEQLLTTIARTINEVENQILTRDAKGISQEQMNEFRASFNHFDRDHSGTLGPEEFKACLISLGYDIGNDPQGEAEFARIMSIVDPNRLGVVTFQAFIDFMSRETADTDTADQVMASFKILAGDKNYITMDELRRELPPDQAEYCIARMAPYTGPDSVPGALDYMSFSTALYGESDL

33) >gi|21264343|ref|NP_002958.2| scaffold attachment factor B1 isoform 3 [Homo sapiens] (102.6 kD)

MAETLSGLGDSGAAGAAALSSASSETGTRRLSDLRVIDLRAELRKRNVDSSGNKSVLMERLKKAIEDEGGNPDEIEITSEGNKKTSKRSSKGRKPEEEGVEDNGLEENSGDGQEDVETSLENLQDIDIMDISVLDEAEIDNGSVADCVEDDDADNLQESLSDSRELVEGEMKELPEQLQEHAIEDKETINNLDTSSSDFTILQEIEEPSLEPENEKILDILGETCKSEPVKEESSELEQPFAQDTSSVGPDRKLAEEEDLFDSAHPEEGDLDLASESTAHAQSSKADSLLAVVKREPAEQPGDGERTDCEPVGLEPAVEQSSAASELAEASSEELAEAPTEAPSPEARDSKEDGRKFDFDACNEVPPAPKESSTSEGADQKMSSPEDDSDTKRLSKEEKGRSSCGRNFWVSGLSSTTRATDLKNLFSKYGKVVGAKVVTNARSPGARCYGFVTMSTAEEATKCINHLHKTELHGKMISVEKAKNEPVGKKTSDKRDSDGKKEKSSNSDRSTNLKRDDKCDRKDDAKKGDDGSGEKSKDQDDQKPGPSERSRATKSGSRGTERTVVMDKSKGVPVISVKTSGSKERASKSQDRKSASREKRSVVSFDKVKEPRKSRDSESHSRVRERSEREQRMQAQWEREERERLEIARERLAFQRQRLERERMERERLERERMHVEHERRREQERIHREREELRRQQELRYEQERRPAVRRPYDLDRRDDAYWPEAKRAALDERYHSDFNRQDRFHDFDHRDRGRYPDHSVDRREGSRSMMGEREGQHYPERHGGPERHGRDSRDGWGGYGSDKRMSEGRGLPPPPRRDWGDHGRREDDRSWQGTADGGMMDRDHKRWQGGERSMSGHSGPGHMMNRGGMSGRGSFAPGGASRGHPIPHGGMQGGFGGQSRGSRPSDARFTRRY

34) >gi|133925811|ref|NP_002261.3| transportin-1 isoform 1 [Homo sapiens] (102.3 kD)

MVWDRQTKMEYEWKPDEQGLQQILQLLKESQSPDTTIQRTVQQKLEQLNQYPDFNNYLIFVLTKLKSEDEPTRSLSGLILKNNVKAHFQNFPNGVTDFIKSECLNNIGDSSPLIRATVGILITTIASKGELQNWPDLLPKLCSLLDSEDYNTCEGAFGALQKICEDSAEILDSDVLDRPLNIMIPKFLQFFKHSSPKIRSHAVACVNQFIISRTQALMLHIDSFIENLFALAGDEEPEVRKNVCRALVMLLEVRMDRLLPHMHNIVEYMLQRTQDQDENVALEACEFWLTLAEQPICKDVLVRHLPKLIPVLVNGMKYSDIDIILLKGDVEEDETIPDSEQDIRPRFHRSRTVAQQHDEDGIEEEDDDDDEIDDDDTISDWNLRKCSAAALDVLANVYRDELLPHILPLLKELLFHHEWVVKESGILVLGAIAEGCMQGMIPYLPELIPHLIQCLSDKKALVRSITCWTLSRYAHWVVSQPPDTYLKPLMTELLKRILDSNKRVQEAACSAFATLEEEACTELVPYLAYILDTLVFAFSKYQHKNLLILYDAIGTLADSVGHHLNKPEYIQMLMPPLIQKWNMLKDEDKDLFPLLECLSSVATALQSGFLPYCEPVYQRCVNLVQKTLAQAMLNNAQPDQYEAPDKDFMIVALDLLSGLAEGLGGNIEQLVARSNILTLMYQCMQDKMPEVRQSSFALLGDLTKACFQHVKPCIADFMPILGTNLNPEFISVCNNATWAIGEISIQMGIEMQPYIPMVLHQLVEIINRPNTPKTLLENTAITIGRLGYVCPQEVAPMLQQFIRPWCTSLRNIRDNEEKDSAFRGICTMISVNPSGVIQDFIFFCDAVASWINPKDDLRDMFCKILHGFKNQVGDENWRRFSDQFPLPLKERLAAFYGV

35) >gi|289577080|ref|NP_001409.3| eukaryotic translation initiation factor 4 gamma 2 isoform 1 [Homo sapiens] (102.3 kD)

MESAIAEGGASRFSASSGGGGSRGAPQHYPKTAGNSEFLGKTPGQNAQKWIPARSTRRDDNSAANNSANEKERHDAIFRKVRGILNKLTPEKFDKLCLELLNVGVESKLILKGVILLIVDKALEEPKYSSLYAQLCLRLAEDAPNFDGPAAEGQPGQKQSTTFRRLLISKLQDEFENRTRNVDVYDKRENPLLPEEEEQRAIAKIKMLGNIKFIGELGKLDLIHESILHKCIKTLLEKKKRVQLKDMGEDLECLCQIMRTVGPRLDHERAKSLMDQYFARMCSLMLSKELPARIRFLLQDTVELREHHWVPRKAFLDNGPKTINQIRQDAVKDLGVFIPAPMAQGMRSDFFLEGPFMPPRMKMDRDPLGGLADMFGQMPGSGIGTGPGVIQDRFSPTMGRHRSNQLFNGHGGHIMPPTQSQFGEMGGKFMKSQGLSQLYHNQSQGLLSQLQGQSKDMPPRFSKKGQLNADEISLRPAQSFLMNKNQVPKLQPQITMIPPSAQPPRTQTPPLGQTPQLGLKTNPPLIQEKPAKTSKKPPPSKEELLKLTETVVTEYLNSGNANEAVNGVREMRAPKHFLPEMLSKVIILSLDRSDEDKEKASSLISLLKQEGIATSDNFMQAFLNVLDQCPKLEVDIPLVKSYLAQFAARAIISELVSISELAQPLESGTHFPLFLLCLQQLAKLQDREWLTELFQQSKVNMQKMLPEIDQNKDRMLEILEGKGLSFLFPLLKLEKELLKQIKLDPSPQTIYKWIKDNISPKLHVDKGFVNILMTSFLQYISSEVNPPSDETDSSSAPSKEQLEQEKQLLLSFKPVMQKFLHDHVDLQVSALYALQVHCYNSNFPKGMLLRFFVHFYDMEIIEEEAFLAWKEDITQEFPGKGKALFQVNQWLTWLETAEEEESEEEAD

36) >gi|13435361|ref|NP_077739.1| desmocollin-1 isoform Dsc1a preproprotein [Homo sapiens] (99.9 kD)

MALASAAPGSIFCKQLLFSLLVLTLLCDACQKVYLRVPSHLQAETLVGKVNLEECLKSASLIRSSDPAFRILEDGSIYTTHDLILSSERKSFSIFLSDGQRREQQEIKVVLSARENKSPKKRHTKDTALKRSKRRWAPIPASLMENSLGPFPQHVQQIQSDAAQNYTIFYSISGPGVDKEPFNLFYIEKDTGDIFCTRSIDREKYEQFALYGYATTADGYAPEYPLPLIIKIEDDNDNAPYFEHRVTIFTVPENCRSGTSVGKVTATDLDEPDTLHTRLKYKILQQIPDHPKHFSIHPDTGVITTTTPFLDREKCDTYQLIMEVRDMGGQPFGLFNTGTITISLEDENDNPPSFTETSYVTEVEENRIDVEILRMKVQDQDLPNTPHSKAVYKILQGNENGNFIISTDPNTNEGVLCVVKPLNYEVNRQVILQVGVINEAQFSKAASSQTPTMCTTTVTVKIIDSDEGPECHPPVKVIQSQDGFPAGQELLGYKALDPEISSGEGLRYQKLGDEDNWFEINQHTGDLRTLKVLDRESKFVKNNQYNISVVAVDAVGRSCTGTLVVHLDDYNDHAPQIDKEVTICQNNEDFAVLKPVDPDGPENGPPFQFFLDNSASKNWNIEEKDGKTAILRQRQNLDYNYYSVPIQIKDRHGLVATHMLTVRVCDCSTPSECRMKDKSTRDVRPNVILGRWAILAMVLGSVLLLCILFTCFCVTAKRTVKKCFPEDIAQQNLIVSNTEGPGEEVTEANIRLPMQTSNICDTSMSVGTVGGQGIKTQQSFEMVKGGYTLDSNKGGGHQTLESVKGVGQGDTGRYAYTDWQSFTQPRLGEKVYLCGQDEEHKHCEDYVCSYNYEGKGSLAGSVGCCSDRQEEEGLEFLDHLEPKFRTLAKTCIKK

37) >gi|4503593|ref|NP_001972.1| epidermal growth factor receptor substrate 15 isoform A [Homo sapiens] (98.6 kD)

MAAAAQLSLTQLSSGNPVYEKYYRQVDTGNTGRVLASDAAAFLKKSGLPDLILGKIWDLADTDGKGILNKQEFFVALRLVACAQNGLEVSLSSLNLAVPPPRFHDTSSPLLISGTSAAELPWAVKPEDKAKYDAIFDSLSPVNGFLSGDKVKPVLLNSKLPVDILGRVWELSDIDHDGMLDRDEFAVAMFLVYCALEKEPVPMSLPPALVPPSKRKTWVVSPAEKAKYDEIFLKTDKDMDGFVSGLEVREIFLKTGLPSTLLAHIWSLCDTKDCGKLSKDQFALAFHLISQKLIKGIDPPHVLTPEMIPPSDRASLQKNIIGSSPVADFSAIKELDTLNNEIVDLQREKNNVEQDLKEKEDTIKQRTSEVQDLQDEVQRENTNLQKLQAQKQQVQELLDELDEQKAQLEEQLKEVRKKCAEEAQLISSLKAELTSQESQISTYEEELAKAREELSRLQQETAELEESVESGKAQLEPLQQHLQDSQQEISSMQMKLMEMKDLENHNSQLNWCSSPHSILVNGATDYCSLSTSSSETANLNEHVEGQSNLESEPIHQESPARSSPELLPSGVTDENEVTTAVTEKVCSELDNNRHSKEEDPFNVDSSSLTGPVADTNLDFFQSDPFVGSDPFKDDPFGKIDPFGGDPFKGSDPFASDCFFRQSTDPFATSSTDPFSAANNSSITSVETLKHNDPFAPGGTVVAASDSATDPFASVFGNESFGGGFADFSTLSKVNNEDPFRSATSSSVSNVVITKNVFEETSVKSEDEPPALPPKIGTPTRPCPLPPGKRSINKLDSPDPFKLNDPFQPFPGNDSPKEKDPEIFCDPFTSATTTTNKEADPSNFANFSAYPSEEDMIEWAKRESEREEEQRLARLNQQEQEDLELAIALSKSEISEA

38) >gi|51094101|ref|NP_076977.3| ATP-dependent RNA helicase DDX54 isoform 2 [Homo sapiens] (98.5 kD)

MAADKGPAAGPRSRAAMAQWRKKKGLRKRRGAASQARGSDSEDGEFEIQAEDDARARKLGPGRPLPTFPTSECTSDVEPDTREMVRAQNKKKKKSGGFQSMGLSYPVFKGIMKKGYKVPTPIQRKTIPVILDGKDVVAMARTGSGKTACFLLPMFERLKTHSAQTGARALILSPTRELALQTLKFTKELGKFTGLKTALILGGDRMEDQFAALHENPDIIIATPGRLVHVAVEMSLKLQSVEYVVFDEADRLFEMGFAEQLQEIIARLPGGHQTVLFSATLPKLLVEFARAGLTEPVLIRLDVDTKLNEQLKTSFFLVREDTKAAVLLHLLHNVVRPQDQTVVFVATKHHAEYLTELLTTQRVSCAHIYSALDPTARKINLAKFTLGKCSTLIVTDLAARGLDIPLLDNVINYSFPAKGKLFLHRVGRVARAGRSGTAYSLVAPDEIPYLLDLHLFLGRSLTLARPLKEPSGVAGVDGMLGRVPQSVVDEEDSGLQSTLEASLELRGLARVADNAQQQYVRSRPAPSPESIKRAKEMDLVGLGLHPLFSSRFEEEELQRLRLVDSIKNYRSRATIFEINASSRDLCSQVMRAKRQKDRKAIARFQQGQQGRQEQQEGPVGPAPSRPALQEKQPEKEEEEEAGESVEDIFSEVVGRKRQRSGPNRGAKRRREEARQRDQEFYIPYRPKDFDSERGLSISGEGGAFEQQAAGAVLDLMGDEAQNLTRGRQQLKWDRKKKRFVGQSGQEDKKKIKTESGRYISSSYKRDLYQKWKQKQKIDDRDSDEEGASDRRGPERRGGKRDRGQGASRPHAPGTPAGRVRPELKTKQQILKQRRRAQKLHFLQRGGLKQLSARNRRRVQELQQGAFGRGARSKKGKMRKRM

39) >gi|56549121|ref|NP_001005360.1| dynamin-2 isoform 1 [Homo sapiens] (98 kD)

MGNRGMEELIPLVNKLQDAFSSIGQSCHLDLPQIAVVGGQSAGKSSVLENFVGRDFLPRGSGIVTRRPLILQLIFSKTEHAEFLHCKSKKFTDFDEVRQEIEAETDRVTGTNKGISPVPINLRVYSPHVLNLTLIDLPGITKVPVGDQPPDIEYQIKDMILQFISRESSLILAVTPANMDLANSDALKLAKEVDPQGLRTIGVITKLDLMDEGTDARDVLENKLLPLRRGYIGVVNRSQKDIEGKKDIRAALAAERKFFLSHPAYRHMADRMGTPHLQKTLNQQLTNHIRESLPALRSKLQSQLLSLEKEVEEYKNFRPDDPTRKTKALLQMVQQFGVDFEKRIEGSGDQVDTLELSGGARINRIFHERFPFELVKMEFDEKDLRREISYAIKNIHGVRTGLFTPDLAFEAIVKKQVVKLKEPCLKCVDLVIQELINTVRQCTSKLSSYPRLREETERIVTTYIREREGRTKDQILLLIDIEQSYINTNHEDFIGFANAQQRSTQLNKKRAIPNQGEILVIRRGWLTINNISLMKGGSKEYWFVLTAESLSWYKDEEEKEKKYMLPLDNLKIRDVEKGFMSNKHVFAIFNTEQRNVYKDLRQIELACDSQEDVDSWKASFLRAGVYPEKDQAENEDGAQENTFSMDPQLERQVETIRNLVDSYVAIINKSIRDLMPKTIMHLMINNTKAFIHHELLAYLYSSADQSSLMEESADQAQRRDDMLRMYHALKEALNIIGDISTSTVSTPVPPPVDDTWLQSASSHSPTPQRRPVSSIHPPGRPPAVRGPTPGPPLIPVPVGAAASFSAPPIPSRPGPQSVFANSDLFPAPPQIPSRPVRIPPGIPPGVPSRRPPAAPSRPTIIRPAEPSLLD

40) >gi|109134349|ref|NP_036265.3| coatomer subunit gamma-2 isoform 1 [Homo sapiens] (97.6 kD)

MIKKFDKKDEESGSGSNPFQHLEKSAVLQEARIFNETPINPRRCLHILTKILYLLNQGEHFGTTEATEAFFAMTRLFQSNDQTLRRMCYLTIKEMATISEDVIIVTSSLTKDMTGKEDVYRGPAIRALCRITDGTMLQAIERYMKQAIVDKVSSVSSSALVSSLHMMKISYDVVKRWINEAQEAASSDNIMVQYHALGVLYHLRKNDRLAVSKMLNKFTKSGLKSQFAYCMLIRIASRLLKETEDGHESPLFDFIESCLRNKHEMVIYEAASAIIHLPNCTARELAPAVSVLQLFCSSPKPALRYAAVRTLNKVAMKHPSAVTACNLDLENLITDSNRSIATLAITTLLKTGSESSVDRLMKQISSFVSEISDEFKVVVVQAISALCQKYPRKHSVMMTFLSNMLRDDGGFEYKRAIVDCIISIVEENPESKEAGLAHLCEFIEDCEHTVLATKILHLLGKEGPRTPVPSKYIRFIFNRVVLENEAVRAAAVSALAKFGAQNESLLPSILVLLQRCMMDTDDEVRDRATFYLNVLQQRQMALNATYIFNGLTVSVPGMEKALHQYTLEPSEKPFDMKSIPLAMAPVFEQKAEITLVATKPEKLAPSRQDIFQEQLAAIPEFLNIGPLFKSSEPVQLTEAETEYFVRCIKHMFTNHIVFQFDCTNTLNDQLLEKVTVQMEPSDSYEVLSCIPAPSLPYNQPGICYTLVRLPDDDPTAVAGSFSCTMKFTVRDCDPNTGVPDEDGYDDEYVLEDLEVTVSDHIQKVLKPNFAAAWEEVGDTFEKEETFALSSTKTLEEAVNNIITFLGMQPCERSDKVPENKNSHSLYLAGIFRGGYDLLVRSRLALADGVTMQVTVRSKERTPVDVILASVG

**Figure legend**: These are 40 largest proteins identified from the 48-kD stripe (MCF7). The red underlined sequences are the LC-MS/MS identified peptide fragments that are unique to the protein, while the green underlined sequences are the LC-MS/MS identified peptide fragments that are not unique to the protein but can also appear in other proteins.

**The 20 smallest proteins identified in the 55-kD stripe (MCF7)**

1) >gi|16751921|ref|NP_444513.1| dermcidin preproprotein [Homo sapiens] (11.3 kD)

MRFMTLLFLTALAGALVCAYDPEAASAPGSGNPCHEASAAQKENAGEDPGLARQAPKPRKQRSSLLEKGLDGAKKAVGGLGKLGKDAVEDLESVGKGAVHDVKDVLDSVL

2) >gi|4504301|ref|NP_003529.1| histone H4 [Homo sapiens] (11.4 kD)

MSGRGKGGKGLGKGGAKRHRKVLRDNIQGITKPAIRRLARRGGVKRISGLIYEETRGVLKVFLENVIRDAVTYTEHAKRKTVTAMDVVYALKRQGRTLYGFGG

3) >gi|38150007|ref|NP_937859.1| small nuclear ribonucleoprotein-associated proteins B and B' isoform B' [Homo sapiens] (24.6 kD)

MTVGKSSKMLQHIDYRMRCILQDGRIFIGTFKAFDKHMNLILCDCDEFRKIKPKNSKQAEREEKRVLGLVLLRGENLVSMTVEGPPPKDTGIARVPLAGAAGGPGIGRAAGRGIPAGVPMPQAPAGLAGPVRGVGGPSQQVMTPQGRGTVAAAAAAATASIAGAPTQYPPGRGGPPPPMGRGAPPPGMMGPPPGMRPPMGPPMGIPPGRGTPMGMPPPGMRPPPPGMRGPPPPGMRPPRP

4) >gi|67782307|ref|NP_001019636.1| superoxide dismutase [Mn], mitochondrial isoform A precursor [Homo sapiens] (24.7 kD)

MLSRAVCGTSRQLAPVLGYLGSRQKHSLPDLPYDYGALEPHINAQIMQLHHSKHHAAYVNNLNVTEEKYQEALAKGDVTAQIALQPALKFNGGGHINHSIFWTNLSPNGGGEPKGELLEAIKRDFGSFDKFKEKLTAASVGVQGSGWGWLGFNKERGHLQIAACPNQDPLQGTTGLIPLLGIDVWEHAYYLQYKNVRPDYLKAIWNVINWENVTERYMACKK

5) >gi|378548190|ref|NP_001243731.1| 40S ribosomal protein S3 isoform 1 [Homo sapiens] (26.7 kD)

MAVQISKKRKFVADGIFKAELNEFLTRELAEDGYSGVEVRVTPTRTEIIILATRTQNVLGEKGRRIRELTAVVQKRFGFPEGSVELYAEKVATRGLCAIAQAESLRYKLLGGLAVRRACYGVLRFIMESGAKGCEVVVSGKLRGQRAKSMKFVDGLMIHSGDPVNYYVDTAVRHVLLRQGVLGIKVKIMLPWDPTGKIGPKKPLPDHVSIVEPKDEILPTTPISEQKGGKPEPPAMPQPVPTA

6) >gi|4502677|ref|NP_001770.1| lymphocyte function-associated antigen 3 isoform 1 [Homo sapiens] (28.1 kD)

MVAGSDAGRALGVLSVVCLLHCFGFISCFSQQIYGVVYGNVTFHVPSNVPLKEVLWKKQKDKVAELENSEFRAFSSFKNRVYLDTVSGSLTIYNLTSSDEDEYEMESPNITDTMKFFLYVLESLPSPTLTCALTNGSIEVQCMIPEHYNSHRGLIMYSWDCPMEQCKRNSTSIYFKMENDLPQKIQCTLSNPLFNTTSSIILTTCIPSSGHSRHRYALIPIPLAVITTCIVLYMNGILKCDRKPDRTNSN

7) >gi|5803185|ref|NP_006745.1| synaptophysin-like protein 1 isoform a [Homo sapiens] (28.5 kD)

MAPNIYLVRQRISRLGQRMSGFQINLNPLKEPLGFIKVLEWIASIFAFATCGGFKGQTEIQVNCPPAVTENKTVTATFGYPFRLNEASFQPPPGVNICDVNWKDYVLIGDYSSSAQFYVTFAVFVFLYCIAALLLYVGYTSLYLDSRKLPMIDFVVTLVATFLWLVSTSAWAKALTDIKIATGHNIIDELPPCKKKAVLCYFGSVTSMGSLNVSVIFGFLNMILWGGNAWFVYKETSLHSPSNTSAPHSQGGIPPPTGI

8) >gi|530404479|ref|XP_005268055.1| PREDICTED: serine/arginine-rich splicing factor 5 isoform X1 [Homo sapiens] (31.2 kD)

MSGCRVFIGRLNPAAREKDVERFFKGYGRIRDIDLKRGFGFVEFEDPRDADDAVYELDGKELCSERVTIEHARARSRGGRGRGRYSDRFSSRRPRNDRRNAPPVRTENRLIVENLSSRVSWQDLKDFMRQAGEVTFADAHRPKLNEGVVEFASYGDLKNAIEKLSGKEINGRKIKLIEGSKRHSRSRSRSRSRTRSSSRSRSRSRSRSRKSYSRSRSRSRSRSRSKSRSVSRSPVPEKSQKRGSSSRSKSPASVDRQRSRSRSRSRSVDSGN

9) >gi|153070260|ref|NP_002347.5| myristoylated alanine-rich C-kinase substrate [Homo sapiens] (31.5 kD)

MGAQFSKTAAKGEAAAERPGEAAVASSPSKANGQENGHVKVNGDASPAAAESGAKEELQANGSAPAADKEEPAAAGSGAASPSAAEKGEPAAAAAPEAGASPVEKEAPAEGEAAEPGSPTAAEGEAASAASSTSSPKAEDGATPSPSNETPKKKKKRFSFKKSFKLSGFSFKKNKKEAGEGGEAEAPAAEGGKDEAAGGAAAAAAEAGAASGEQAAAPGEEAAAGEEGAAGGDPQEAKPQEAAVAPEKPPASDETKAAEEPSKVEEKKAEEAGASAAACEAPSAAGPGAPPEQEAAPAEEPAAAAASSACAAPSQEAQPECSPEAPPAEAAE

10) >gi|61966711|ref|NP_001013653.1| heterogeneous nuclear ribonucleoprotein C-like 1 [Homo sapiens] (32.1 kD)

MASNVTNKMDPHSMNSRVFIGNLNTLVVKKSDVEAIFSKYGKIAGCSVHKGFAFVQYDKEKNARAAVAGEDGRMIASQVVDINLAAEPKVNRGNAGVKRSAAEMYGSSFDLDYGFQRDYYDGMYSFPARVPPPPPIALAVVPSKRQRLSGNTSRRGKSGFNSKSGKRGSSKSGKLKGDDLQAIKQELTQIKQKVDSLLENLEKIEKEQSKQEVEVKNAKSEEEQSSSSMKKDETHVKMESEGGAEDSAEEGDPLDDDVNEDQGDDQLELIKDDEKEAEEGEDDRDSTNGQDDS

11) >gi|10835063|ref|NP_002511.1| nucleophosmin isoform 1 [Homo sapiens] (32.6 kD)

MEDSMDMDMSPLRPQNYLFGCELKADKDYHFKVDNDENEHQLSLRTVSLGAGAKDELHIVEAEAMNYEGSPIKVTLATLKMSVQPTVSLGGFEITPPVVLRLKCGSGPVHISGQHLVAVEEDAESEDEEEEDVKLLSISGKRSAPGGGSKVPQKKVKLAADEDDDDDDEEDDDEDDDDDDFDDEEAEEKAPVKKSIRDTPAKNAQKSNQNGKDSKPSSTPRSKGQESFKKQEKTPKTPKGPSSVEDIKAKMQASIEKGGSLPKVEAKFINYVKNCFRMTDQEAIQDLWQWRKSL

12) >gi|156071459|ref|NP_001143.2| ADP/ATP translocase 2 [Homo sapiens] (32.9 kD)

MTDAAVSFAKDFLAGGVAAAISKTAVAPIERVKLLLQVQHASKQITADKQYKGIIDCVVRIPKEQGVLSFWRGNLANVIRYFPTQALNFAFKDKYKQIFLGGVDKRTQFWLYFAGNLASGGAAGATSLCFVYPLDFARTRLAADVGKAGAEREFRGLGDCLVKIYKSDGIKGLYQGFNVSVQGIIIYRAAYFGIYDTAKGMLPDPKNTHIVISWMIAQTVTAVAGLTSYPFDTVRRRMMMQSGRKGTDIMYTGTLDCWRKIARDEGGKAFFKGAWSNVLRGMGGAFVLVLYDEIKKYT

13) >gi|116812600|ref|NP_006636.2| PCTP-like protein [Homo sapiens] (33 kD)

MEKLAASTEPQGPRPVLGRESVQVPDDQDFRSFRSECEAEVGWNLTYSRAGVSVWVQAVEMDRTLHKIKCRMECCDVPAETLYDVLHDIEYRKKWDSNVIETFDIARLTVNADVGYYSWRCPKPLKNRDVITLRSWLPMGADYIIMNYSVKHPKYPPRKDLVRAVSIQTGYLIQSTGPKSCVITYLAQVDPKGSLPKWVVNKSSQFLAPKAMKKMYKACLKYPEWKQKHLPHFKPWLHPEQSPLPSLALSELSVQHADSLENIDESAVAESREERMGGAGGEGSDDDTSLT

14) >gi|55749577|ref|NP_001142.2| ADP/ATP translocase 1 [Homo sapiens] (33 kD)

MGDHAWSFLKDFLAGGVAAAVSKTAVAPIERVKLLLQVQHASKQISAEKQYKGIIDCVVRIPKEQGFLSFWRGNLANVIRYFPTQALNFAFKDKYKQLFLGGVDRHKQFWRYFAGNLASGGAAGATSLCFVYPLDFARTRLAADVGKGAAQREFHGLGDCIIKIFKSDGLRGLYQGFNVSVQGIIIYRAAYFGVYDTAKGMLPDPKNVHIFVSWMIAQSVTAVAGLVSYPFDTVRRRMMMQSGRKGADIMYTGTVDCWRKIAKDEGAKAFFKGAWSNVLRGMGGAFVLVLYDEIKKYV

15) >gi|221307584|ref|NP_001138303.1| prohibitin-2 isoform 1 [Homo sapiens] (33.3 kD)

MAQNLKDLAGRLPAGPRGMGTALKLLLGAGAVAYGVRESVFTVEGGHRAIFFNRIGGVQQDTILAEGLHFRIPWFQYPIIYDIRARPRKISSPTGSKDLQMVNISLRVLSRPNAQELPSMYQRLGLDYEERVLPSIVNEVLKSVVAKFNASQLITQRAQVSLLIRRELTERAKDFSLILDDVAITELSFSREYTAAVEAKQVAQQEAQRAQFLVEKAKQEQRQKIVQAEGEAEAAKMLGEALSKNPGYIKLRKIRAAQNISKTIATSQNRIYLTADNLVLNLQDESFTRGSDSLIKGKK

16) >gi|5031981|ref|NP_005796.1| 26S proteasome non-ATPase regulatory subunit 14 [Homo sapiens] (34.6 kD)

MDRLLRLGGGMPGLGQGPPTDAPAVDTAEQVYISSLALLKMLKHGRAGVPMEVMGLMLGEFVDDYTVRVIDVFAMPQSGTGVSVEAVDPVFQAKMLDMLKQTGRPEMVVGWYHSHPGFGCWLSGVDINTQQSFEALSERAVAVVVDPIQSVKGKVVIDAFRLINANMMVLGHEPRQTTSNLGHLNKPSIQALIHGLNRHYYSITINYRKNELEQKMLLNLHKKSWMEGLTLQDYSEHCKHNESVVKEMLELAKNYNKAVEEEDKMTPEQLAIKNVGKQDPKRHLEEHVDVLMTSNIVQCLAAMLDTVVFK

17) >gi|21735621|ref|NP_005909.2| malate dehydrogenase, mitochondrial isoform 1 precursor [Homo sapiens] (35.5 kD)

MLSALARPASAALRRSFSTSAQNNAKVAVLGASGGIGQPLSLLLKNSPLVSRLTLYDIAHTPGVAADLSHIETKAAVKGYLGPEQLPDCLKGCDVVVIPAGVPRKPGMTRDDLFNTNATIVATLTAACAQHCPEAMICVIANPVNSTIPITAEVFKKHGVYNPNKIFGVTTLDIVRANTFVAELKGLDPARVNVPVIGGHAGKTIIPLISQCTPKVDFPQDQLTALTGRIQEAGTEVVKAKAGAGSATLSMAYAGARFVFSLVDAMNGKEGVVECSFVKSQETECTYFSTPLLLGKKGIEKNLGIGKVSSFEEKMISDAIPELKASIKKGEDFVKTLK

18) >gi|7669492|ref|NP_002037.2| glyceraldehyde-3-phosphate dehydrogenase isoform 1 [Homo sapiens] (36 kD)

MGKVKVGVNGFGRIGRLVTRAAFNSGKVDIVAINDPFIDLNYMVYMFQYDSTHGKFHGTVKAENGKLVINGNPITIFQERDPSKIKWGDAGAEYVVESTGVFTTMEKAGAHLQGGAKRVIISAPSADAPMFVMGVNHEKYDNSLKIISNASCTTNCLAPLAKVIHDNFGIVEGLMTTVHAITATQKTVDGPSGKLWRDGRGALQNIIPASTGAAKAVGKVIPELNGKLTGMAFRVPTANVSVVDLTCRLEKPAKYDDIKKVVKQASEGPLKGILGYTEHQVVSSDFNSDTHSSTFDAGAGIALNDHFVKLISWYDNEFGYSNRVVDLMAHMASKE

19) >gi|4758256|ref|NP_004085.1| eukaryotic translation initiation factor 2 subunit 1 [Homo sapiens] (36.1 kD)

MPGLSCRFYQHKFPEVEDVVMVNVRSIAEMGAYVSLLEYNNIEGMILLSELSRRRIRSINKLIRIGRNECVVVIRVDKEKGYIDLSKRRVSPEEAIKCEDKFTKSKTVYSILRHVAEVLEYTKDEQLESLFQRTAWVFDDKYKRPGYGAYDAFKHAVSDPSILDSLDLNEDEREVLINNINRRLTPQAVKIRADIEVACYGYEGIDAVKEALRAGLNCSTENMPIKINLIAPPRYVMTTTTLERTEGLSVLSQAMAVIKEKIEEKRGVFNVQMEPKVVTDTDETELARQMERLERENAEVDGDDDAEEMEAKAED

20) >gi|4557032|ref|NP_002291.1| L-lactate dehydrogenase B chain [Homo sapiens] (36.6 kD)

MATLKEKLIAPVAEEEATVPNNKITVVGVGQVGMACAISILGKSLADELALVDVLEDKLKGEMMDLQHGSLFLQTPKIVADKDYSVTANSKIVVVTAGVRQQEGESRLNLVQRNVNVFKFIIPQIVKYSPDCIIIVVSNPVDILTYVTWKLSGLPKHRVIGSGCNLDSARFRYLMAEKLGIHPSSCHGWILGEHGDSSVAVWSGVNVAGVSLQELNPEMGTDNDSENWKEVHKMVVESAYEVIKLKGYTNWAIGLSVADLIESMLKNLSRIHPVSTMVKGMYGIENEVFLSLPCILNARGLTSVINQKLKDDEVAQLKKSADTLWDIQKDLKDL

**Figure legend**: These are 20 smallest proteins identified in the 55-kD stripe (MCF7). The red underlined sequences are the LC-MS/MS identified peptide fragments that are unique to the protein, while the green underlined sequences are the LC-MS/MS identified peptide fragments that are not unique to the protein but can also appear in other proteins.

**The 40 largest proteins in the 55-kD stripe (MCF7)**

1) >gi|41322916|ref|NP_958782.1| plectin isoform 1 [Homo sapiens] (531.5 kD)

MVAGMLMPRDQLRAIYEVLFREGVMVAKKDRRPRSLHPHVPGVTNLQVMRAMASLRARGLVRETFAWCHFYWYLTNEGIAHLRQYLHLPPEIVPASLQRVRRPVAMVMPARRTPHVQAVQGPLGSPPKRGPLPTEEQRVYRRKELEEVSPETPVVPATTQRTLARPGPEPAPATDERDRVQKKTFTKWVNKHLIKAQRHISDLYEDLRDGHNLISLLEVLSGDSLPREKGRMRFHKLQNVQIALDYLRHRQVKLVNIRNDDIADGNPKLTLGLIWTIILHFQISDIQVSGQSEDMTAKEKLLLWSQRMVEGYQGLRCDNFTSSWRDGRLFNAIIHRHKPLLIDMNKVYRQTNLENLDQAFSVAERDLGVTRLLDPEDVDVPQPDEKSIITYVSSLYDAMPRVPDVQDGVRANELQLRWQEYRELVLLLLQWMRHHTAAFEERRFPSSFEEIEILWSQFLKFKEMELPAKEADKNRSKGIYQSLEGAVQAGQLKVPPGYHPLDVEKEWGKLHVAILEREKQLRSEFERLECLQRIVTKLQMEAGLCEEQLNQADALLQSDVRLLAAGKVPQRAGEVERDLDKADSMIRLLFNDVQTLKDGRHPQGEQMYRRVYRLHERLVAIRTEYNLRLKAGVAAPATQVAQVTLQSVQRRPELEDSTLRYLQDLLAWVEENQHRVDGAEWGVDLPSVEAQLGSHRGLHQSIEEFRAKIERARSDEGQLSPATRGAYRDCLGRLDLQYAKLLNSSKARLRSLESLHSFVAAATKELMWLNEKEEEEVGFDWSDRNTNMTAKKESYSALMRELELKEKKIKELQNAGDRLLREDHPARPTVESFQAALQTQWSWMLQLCCCIEAHLKENAAYFQFFSDVREAEGQLQKLQEALRRKYSCDRSATVTRLEDLLQDAQDEKEQLNEYKGHLSGLAKRAKAVVQLKPRHPAHPMRGRLPLLAVCDYKQVEVTVHKGDECQLVGPAQPSHWKVLSSSGSEAAVPSVCFLVPPPNQEAQEAVTRLEAQHQALVTLWHQLHVDMKSLLAWQSLRRDVQLIRSWSLATFRTLKPEEQRQALHSLELHYQAFLRDSQDAGGFGPEDRLMAEREYGSCSHHYQQLLQSLEQGAQEESRCQRCISELKDIRLQLEACETRTVHRLRLPLDKEPARECAQRIAEQQKAQAEVEGLGKGVARLSAEAEKVLALPEPSPAAPTLRSELELTLGKLEQVRSLSAIYLEKLKTISLVIRGTQGAEEVLRAHEEQLKEAQAVPATLPELEATKASLKKLRAQAEAQQPTFDALRDELRGAQEVGERLQQRHGERDVEVERWRERVAQLLERWQAVLAQTDVRQRELEQLGRQLRYYRESADPLGAWLQDARRRQEQIQAMPLADSQAVREQLRQEQALLEEIERHGEKVEECQRFAKQYINAIKDYELQLVTYKAQLEPVASPAKKPKVQSGSESVIQEYVDLRTHYSELTTLTSQYIKFISETLRRMEEEERLAEQQRAEERERLAEVEAALEKQRQLAEAHAQAKAQAEREAKELQQRMQEEVVRREEAAVDAQQQKRSIQEELQQLRQSSEAEIQAKARQAEAAERSRLRIEEEIRVVRLQLEATERQRGGAEGELQALRARAEEAEAQKRQAQEEAERLRRQVQDESQRKRQAEVELASRVKAEAEAAREKQRALQALEELRLQAEEAERRLRQAEVERARQVQVALETAQRSAEAELQSKRASFAEKTAQLERSLQEEHVAVAQLREEAERRAQQQAEAERAREEAERELERWQLKANEALRLRLQAEEVAQQKSLAQAEAEKQKEEAEREARRRGKAEEQAVRQRELAEQELEKQRQLAEGTAQQRLAAEQELIRLRAETEQGEQQRQLLEEELARLQREAAAATQKRQELEAELAKVRAEMEVLLASKARAEEESRSTSEKSKQRLEAEAGRFRELAEEAARLRALAEEAKRQRQLAEEDAARQRAEAERVLAEKLAAIGEATRLKTEAEIALKEKEAENERLRRLAEDEAFQRRRLEEQAAQHKADIEERLAQLRKASDSELERQKGLVEDTLRQRRQVEEEILALKASFEKAAAGKAELELELGRIRSNAEDTLRSKEQAELEAARQRQLAAEEERRRREAEERVQKSLAAEEEAARQRKAALEEVERLKAKVEEARRLRERAEQESARQLQLAQEAAQKRLQAEEKAHAFAVQQKEQELQQTLQQEQSVLDQLRGEAEAARRAAEEAEEARVQAEREAAQSRRQVEEAERLKQSAEEQAQARAQAQAAAEKLRKEAEQEAARRAQAEQAALRQKQAADAEMEKHKKFAEQTLRQKAQVEQELTTLRLQLEETDHQKNLLDEELQRLKAEATEAARQRSQVEEELFSVRVQMEELSKLKARIEAENRALILRDKDNTQRFLQEEAEKMKQVAEEAARLSVAAQEAARLRQLAEEDLAQQRALAEKMLKEKMQAVQEATRLKAEAELLQQQKELAQEQARRLQEDKEQMAQQLAEETQGFQRTLEAERQRQLEMSAEAERLKLRVAEMSRAQARAEEDAQRFRKQAEEIGEKLHRTELATQEKVTLVQTLEIQRQQSDHDAERLREAIAELEREKEKLQQEAKLLQLKSEEMQTVQQEQLLQETQALQQSFLSEKDSLLQRERFIEQEKAKLEQLFQDEVAKAQQLREEQQRQQQQMEQERQRLVASMEEARRRQHEAEEGVRRKQEELQQLEQQRRQQEELLAEENQRLREQLQLLEEQHRAALAHSEEVTASQVAATKTLPNGRDALDGPAAEAEPEHSFDGLRRKVSAQRLQEAGILSAEELQRLAQGHTTVDELARREDVRHYLQGRSSIAGLLLKATNEKLSVYAALQRQLLSPGTALILLEAQAASGFLLDPVRNRRLTVNEAVKEGVVGPELHHKLLSAERAVTGYKDPYTGQQISLFQAMQKGLIVREHGIRLLEAQIATGGVIDPVHSHRVPVDVAYRRGYFDEEMNRVLADPSDDTKGFFDPNTHENLTYLQLLERCVEDPETGLCLLPLTDKAAKGGELVYTDSEARDVFEKATVSAPFGKFQGKTVTIWEIINSEYFTAEQRRDLLRQFRTGRITVEKIIKIIITVVEEQEQKGRLCFEGLRSLVPAAELLESRVIDRELYQQLQRGERSVRDVAEVDTVRRALRGANVIAGVWLEEAGQKLSIYNALKKDLLPSDMAVALLEAQAGTGHIIDPATSARLTVDEAVRAGLVGPEFHEKLLSAEKAVTGYRDPYTGQSVSLFQALKKGLIPREQGLRLLDAQLSTGGIVDPSKSHRVPLDVACARGCLDEETSRALSAPRADAKAYSDPSTGEPATYGELQQRCRPDQLTGLSLLPLSEKAARARQEELYSELQARETFEKTPVEVPVGGFKGRTVTVWELISSEYFTAEQRQELLRQFRTGKVTVEKVIKILITIVEEVETLRQERLSFSGLRAPVPASELLASGVLSRAQFEQLKDGKTTVKDLSELGSVRTLLQGSGCLAGIYLEDTKEKVSIYEAMRRGLLRATTAALLLEAQAATGFLVDPVRNQRLYVHEAVKAGVVGPELHEQLLSAEKAVTGYRDPYSGSTISLFQAMQKGLVLRQHGIRLLEAQIATGGIIDPVHSHRVPVDVAYQRGYFSEEMNRVLADPSDDTKGFFDPNTHENLTYRQLLERCVEDPETGLRLLPLKGAEKAEVVETTQVYTEEETRRAFEETQIDIPGGGSHGGSTMSLWEVMQSDLIPEEQRAQLMADFQAGRVTKERMIIIIIEIIEKTEIIRQQGLASYDYVRRRLTAEDLFEARIISLETYNLLREGTRSLREALEAESAWCYLYGTGSVAGVYLPGSRQTLSIYQALKKGLLSAEVARLLLEAQAATGFLLDPVKGERLTVDEAVRKGLVGPELHDRLLSAERAVTGYRDPYTEQTISLFQAMKKELIPTEEALRLLDAQLATGGIVDPRLGFHLPLEVAYQRGYLNKDTHDQLSEPSEVRSYVDPSTDERLSYTQLLRRCRRDDGTGQLLLPLSDARKLTFRGLRKQITMEELVRSQVMDEATALQLREGLTSIEEVTKNLQKFLEGTSCIAGVFVDATKERLSVYQAMKKGIIRPGTAFELLEAQAATGYVIDPIKGLKLTVEEAVRMGIVGPEFKDKLLSAERAVTGYKDPYSGKLISLFQAMKKGLILKDHGIRLLEAQIATGGIIDPEESHRLPVEVAYKRGLFDEEMNEILTDPSDDTKGFFDPNTEENLTYLQLMERCITDPQTGLCLLPLKEKKRERKTSSKSSVRKRRVVIVDPETGKEMSVYEAYRKGLIDHQTYLELSEQECEWEEITISSSDGVVKSMIIDRRSGRQYDIDDAIAKNLIDRSALDQYRAGTLSITEFADMLSGNAGGFRSRSSSVGSSSSYPISPAVSRTQLASWSDPTEETGPVAGILDTETLEKVSITEAMHRNLVDNITGQRLLEAQACTGGIIDPSTGERFPVTDAVNKGLVDKIMVDRINLAQKAFCGFEDPRTKTKMSAAQALKKGWLYYEAGQRFLEVQYLTGGLIEPDTPGRVPLDEALQRGTVDARTAQKLRDVGAYSKYLTCPKTKLKISYKDALDRSMVEEGTGLRLLEAAAQSTKGYYSPYSVSGSGSTAGSRTGSRTGSRAGSRRGSFDATGSGFSMTFSSSSYSSSGYGRRYASGSSASLGGPESAVA

2) >gi|13654237|ref|NP_008835.5| DNA-dependent protein kinase catalytic subunit isoform 1 [Homo sapiens] (468.8 kD)

MAGSGAGVRCSLLRLQETLSAADRCGAALAGHQLIRGLGQECVLSSSPAVLALQTSLVFSRDFGLLVFVRKSLNSIEFRECREEILKFLCIFLEKMGQKIAPYSVEIKNTCTSVYTKDRAAKCKIPALDLLIKLLQTFRSSRLMDEFKIGELFSKFYGELALKKKIPDTVLEKVYELLGLLGEVHPSEMINNAENLFRAFLGELKTQMTSAVREPKLPVLAGCLKGLSSLLCNFTKSMEEDPQTSREIFNFVLKAIRPQIDLKRYAVPSAGLRLFALHASQFSTCLLDNYVSLFEVLLKWCAHTNVELKKAALSALESFLKQVSNMVAKNAEMHKNKLQYFMEQFYGIIRNVDSNNKELSIAIRGYGLFAGPCKVINAKDVDFMYVELIQRCKQMFLTQTDTGDDRVYQMPSFLQSVASVLLYLDTVPEVYTPVLEHLVVMQIDSFPQYSPKMQLVCCRAIVKVFLALAAKGPVLRNCISTVVHQGLIRICSKPVVLPKGPESESEDHRASGEVRTGKWKVPTYKDYVDLFRHLLSSDQMMDSILADEAFFSVNSSSESLNHLLYDEFVKSVLKIVEKLDLTLEIQTVGEQENGDEAPGVWMIPTSDPAANLHPAKPKDFSAFINLVEFCREILPEKQAEFFEPWVYSFSYELILQSTRLPLISGFYKLLSITVRNAKKIKYFEGVSPKSLKHSPEDPEKYSCFALFVKFGKEVAVKMKQYKDELLASCLTFLLSLPHNIIELDVRAYVPALQMAFKLGLSYTPLAEVGLNALEEWSIYIDRHVMQPYYKDILPCLDGYLKTSALSDETKNNWEVSALSRAAQKGFNKVVLKHLKKTKNLSSNEAISLEEIRIRVVQMLGSLGGQINKNLLTVTSSDEMMKSYVAWDREKRLSFAVPFREMKPVIFLDVFLPRVTELALTASDRQTKVAACELLHSMVMFMLGKATQMPEGGQGAPPMYQLYKRTFPVLLRLACDVDQVTRQLYEPLVMQLIHWFTNNKKFESQDTVALLEAILDGIVDPVDSTLRDFCGRCIREFLKWSIKQITPQQQEKSPVNTKSLFKRLYSLALHPNAFKRLGASLAFNNIYREFREEESLVEQFVFEALVIYMESLALAHADEKSLGTIQQCCDAIDHLCRIIEKKHVSLNKAKKRRLPRGFPPSASLCLLDLVKWLLAHCGRPQTECRHKSIELFYKFVPLLPGNRSPNLWLKDVLKEEGVSFLINTFEGGGCGQPSGILAQPTLLYLRGPFSLQATLCWLDLLLAALECYNTFIGERTVGALQVLGTEAQSSLLKAVAFFLESIAMHDIIAAEKCFGTGAAGNRTSPQEGERYNYSKCTVVVRIMEFTTTLLNTSPEGWKLLKKDLCNTHLMRVLVQTLCEPASIGFNIGDVQVMAHLPDVCVNLMKALKMSPYKDILETHLREKITAQSIEELCAVNLYGPDAQVDRSRLAAVVSACKQLHRAGLLHNILPSQSTDLHHSVGTELLSLVYKGIAPGDERQCLPSLDLSCKQLASGLLELAFAFGGLCERLVSLLLNPAVLSTASLGSSQGSVIHFSHGEYFYSLFSETINTELLKNLDLAVLELMQSSVDNTKMVSAVLNGMLDQSFRERANQKHQGLKLATTILQHWKKCDSWWAKDSPLETKMAVLALLAKILQIDSSVSFNTSHGSFPEVFTTYISLLADTKLDLHLKGQAVTLLPFFTSLTGGSLEELRRVLEQLIVAHFPMQSREFPPGTPRFNNYVDCMKKFLDALELSQSPMLLELMTEVLCREQQHVMEELFQSSFRRIARRGSCVTQVGLLESVYEMFRKDDPRLSFTRQSFVDRSLLTLLWHCSLDALREFFSTIVVDAIDVLKSRFTKLNESTFDTQITKKMGYYKILDVMYSRLPKDDVHAKESKINQVFHGSCITEGNELTKTLIKLCYDAFTENMAGENQLLERRRLYHCAAYNCAISVICCVFNELKFYQGFLFSEKPEKNLLIFENLIDLKRRYNFPVEVEVPMERKKKYIEIRKEAREAANGDSDGPSYMSSLSYLADSTLSEEMSQFDFSTGVQSYSYSSQDPRPATGRFRRREQRDPTVHDDVLELEMDELNRHECMAPLTALVKHMHRSLGPPQGEEDSVPRDLPSWMKFLHGKLGNPIVPLNIRLFLAKLVINTEEVFRPYAKHWLSPLLQLAASENNGGEGIHYMVVEIVATILSWTGLATPTGVPKDEVLANRLLNFLMKHVFHPKRAVFRHNLEIIKTLVECWKDCLSIPYRLIFEKFSGKDPNSKDNSVGIQLLGIVMANDLPPYDPQCGIQSSEYFQALVNNMSFVRYKEVYAAAAEVLGLILRYVMERKNILEESLCELVAKQLKQHQNTMEDKFIVCLNKVTKSFPPLADRFMNAVFFLLPKFHGVLKTLCLEVVLCRVEGMTELYFQLKSKDFVQVMRHRDDERQKVCLDIIYKMMPKLKPVELRELLNPVVEFVSHPSTTCREQMYNILMWIHDNYRDPESETDNDSQEIFKLAKDVLIQGLIDENPGLQLIIRNFWSHETRLPSNTLDRLLALNSLYSPKIEVHFLSLATNFLLEMTSMSPDYPNPMFEHPLSECEFQEYTIDSDWRFRSTVLTPMFVETQASQGTLQTRTQEGSLSARWPVAGQIRATQQQHDFTLTQTADGRSSFDWLTGSSTDPLVDHTSPSSDSLLFAHKRSERLQRAPLKSVGPDFGKKRLGLPGDEVDNKVKGAAGRTDLLRLRRRFMRDQEKLSLMYARKGVAEQKREKEIKSELKMKQDAQVVLYRSYRHGDLPDIQIKHSSLITPLQAVAQRDPIIAKQLFSSLFSGILKEMDKFKTLSEKNNITQKLLQDFNRFLNTTFSFFPPFVSCIQDISCQHAALLSLDPAAVSAGCLASLQQPVGIRLLEEALLRLLPAELPAKRVRGKARLPPDVLRWVELAKLYRSIGEYDVLRGIFTSEIGTKQITQSALLAEARSDYSEAAKQYDEALNKQDWVDGEPTEAEKDFWELASLDCYNHLAEWKSLEYCSTASIDSENPPDLNKIWSEPFYQETYLPYMIRSKLKLLLQGEADQSLLTFIDKAMHGELQKAILELHYSQELSLLYLLQDDVDRAKYYIQNGIQSFMQNYSSIDVLLHQSRLTKLQSVQALTEIQEFISFISKQGNLSSQVPLKRLLNTWTNRYPDAKMDPMNIWDDIITNRCFFLSKIEEKLTPLPEDNSMNVDQDGDPSDRMEVQEQEEDISSLIRSCKFSMKMKMIDSARKQNNFSLAMKLLKELHKESKTRDDWLVSWVQSYCRLSHCRSRSQGCSEQVLTVLKTVSLLDENNVSSYLSKNILAFRDQNILLGTTYRIIANALSSEPACLAEIEEDKARRILELSGSSSEDSEKVIAGLYQRAFQHLSEAVQAAEEEAQPPSWSCGPAAGVIDAYMTLADFCDQQLRKEEENASVIDSAELQAYPALVVEKMLKALKLNSNEARLKFPRLLQIIERYPEETLSLMTKEISSVPCWQFISWISHMVALLDKDQAVAVQHSVEEITDNYPQAIVYPFIISSESYSFKDTSTGHKNKEFVARIKSKLDQGGVIQDFINALDQLSNPELLFKDWSNDVRAELAKTPVNKKNIEKMYERMYAALGDPKAPGLGAFRRKFIQTFGKEFDKHFGKGGSKLLRMKLSDFNDITNMLLLKMNKDSKPPGNLKECSPWMSDFKVEFLRNELEIPGQYDGRGKPLPEYHVRIAGFDERVTVMASLRRPKRIIIRGHDEREHPFLVKGGEDLRQDQRVEQLFQVMNGILAQDSACSQRALQLRTYSVVPMTSRLGLIEWLENTVTLKDLLLNTMSQEEKAAYLSDPRAPPCEYKDWLTKMSGKHDVGAYMLMYKGANRTETVTSFRKRESKVPADLLKRAFVRMSTSPEAFLALRSHFASSHALICISHWILGIGDRHLNNFMVAMETGGVIGIDFGHAFGSATQFLPVPELMPFRLTRQFINLMLPMKETGLMYSIMVHALRAFRSDPGLLTNTMDVFVKEPSFDWKNFEQKMLKKGGSWIQEINVAEKNWYPRQKICYAKRKLAGANPAVITCDELLLGHEKAPAFRDYVAVARGSKDHNIRAQEPESGLSEETQVKCLMDQATDPNILGRTWEGWEPWM

3) >gi|66346674|ref|NP_150648.2| vacuolar protein sorting-associated protein 13A isoform A [Homo sapiens] (360 kD)

MVFESVVVDVLNRFLGDYVVDLDTSQLSLGIWKGAVALKNLQIKENALSQLDVPFKVKVGHIGNLKLIIPWKNLYTQPVEAVLEEIYLLIVPSSRIKYDPLKEEKQLMEAKQQELKRIEEAKQKVVDQEQHLPEKQDTFAEKLVTQIIKNLQVKISSIHIRYEDDITNRDKPLSFGISLQNLSMQTTDQYWVPCLHDETEKLVRKLIRLDNLFAYWNVKSQMFYLSDYDNSLDDLKNGIVNENIVPEGYDFVFRPISANAKLVMNRRSDFDFSAPKINLEIELHNIAIEFNKPQYFSIMELLESVDMMAQNLPYRKFKPDVPLHHHAREWWAYAIHGVLEVNVCPRLWMWSWKHIRKHRQKVKQYKELYKKKLTSKKPPGELLVSLEELEKTLDVFNITIARQTAEVEVKKAGYKIYKEGVKDPEDNKGWFSWLWSWSEQNTNEQQPDVQPETLEEMLTPEEKALLYEAIGYSETAVDPTLLKTFEALKFFVHLKSMSIVLRENHQKPELVDIVIEEFSTLIVQRPGAQAIKFETKIDSFHITGLPDNSEKPRLLSSLDDAMSLFQITFEINPLDETVSQRCIIEAEPLEIIYDARTVNSIVEFFRPPKEVHLAQLTAATLTKLEEFRSKTATGLLYIIETQKVLDLKINLKASYIIVPQDGIFSPTSNLLLLDLGHLKVTSKSRSELPDVKQGEANLKEIMDRAYDSFDIQLTSVQLLYSRVGDNWREARKLSVSTQHILVPMHFNLELSKAMVFMDVRMPKFKIYGKLPLISLRISDKKLQGIMELIESIPKPEPVTEVSAPVKSFQIQTSTSLGTSQISQKIIPLLELPSVSEDDSEEEFFDAPCSPLEEPLQFPTGVKSIRTRKLQKQDCSVNMTTFKIRFEVPKVLIEFYHLVGDCELSVVEILVLGLGAEIEIRTYDLKANAFLKEFCLKCPEYLDENKKPVYLVTTLDNTMEDLLTLEYVKAEKNVPDLKSTYNNVLQLIKVNFSSLDIHLHTEALLNTINYLHNILPQSEEKSAPVSTTETEDKGDVIKKLALKLSTNEDIITLQILAELSCLQIFIQDQKCNISEIKIEGLDSEMIMRPSETEINAKLRNIIVLDSDITAIYKKAVYITGKEVFSFKMVSYMDATAGSAYTDMNVVDIQVNLIVGCIEVVFVTKFLYSILAFIDNFQAAKQALAEATVQAAGMAATGVKELAQRSSRMALDINIKAPVVVIPQSPVSENVFVADFGLITMTNTFHMITESQSSPPPVIDLITIKLSEMRLYRSRFINDAYQEVLDLLLPLNLEVVVERNLCWEWYQEVPCFNVNAQLKPMEFILSQEDITTIFKTLHGNIWYEKDGSASPAVTKDQYSATSGVTTNASHHSGGATVVTAAVVEVHSRALLVKTTLNISFKTDDLTMVLYSPGPKQASFTDVRDPSLKLAEFKLENIISTLKMYTDGSTFSSFSLKNCILDDKRPHVKKATPRMIGLTVGFDKKDMMDIKYRKVRDGCVTDAVFQEMYICASVEFLQTVANVFLEAYTTGTAVETSVQTWTAKEEVPTQESVKWEINVIIKNPEIVFVADMTKNDAPALVITTQCEICYKGNLENSTMTAAIKDLQVRACPFLPVKRKGKITTVLQPCDLFYQTTQKGTDPQVIDMSVKSLTLKVSPVIINTMITITSALYTTKETIPEETASSTAHLWEKKDTKTLKMWFLEESNETEKIAPTTELVPKGEMIKMNIDSIFIVLEAGIGHRTVPMLLAKSRFSGEGKNWSSLINLHCQLELEVHYYNEMFGVWEPLLEPLEIDQTEDFRPWNLGIKMKKKAKMAIVESDPEEENYKVPEYKTVISFHSKDQLNITLSKCGLVMLNNLVKAFTEAATGSSADFVKDLAPFMILNSLGLTISVSPSDSFSVLNIPMAKSYVLKNGESLSMDYIRTKDNDHFNAMTSLSSKLFFILLTPVNHSTADKIPLTKVGRRLYTVRHRESGVERSIVCQIDTVEGSKKVTIRSPVQIRNHFSVPLSVYEGDTLLGTASPENEFNIPLGSYRSFIFLKPEDENYQMCEGIDFEEIIKNDGALLKKKCRSKNPSKESFLINIVPEKDNLTSLSVYSEDGWDLPYIMHLWPPILLRNLLPYKIAYYIEGIENSVFTLSEGHSAQICTAQLGKARLHLKLLDYLNHDWKSEYHIKPNQQDISFVSFTCVTEMEKTDLDIAVHMTYNTGQTVVAFHSPYWMVNKTGRMLQYKADGIHRKHPPNYKKPVLFSFQPNHFFNNNKVQLMVTDSELSNQFSIDTVGSHGAVKCKGLKMDYQVGVTIDLSSFNITRIVTFTPFYMIKNKSKYHISVAEEGNDKWLSLDLEQCIPFWPEYASSKLLIQVERSEDPPKRIYFNKQENCILLRLDNELGGIIAEVNLAEHSTVITFLDYHDGAATFLLINHTKNELVQYNQSSLSEIEDSLPPGKAVFYTWADPVGSRRLKWRCRKSHGEVTQKDDMMMPIDLGEKTIYLVSFFEGLQRIILFTEDPRVFKVTYESEKAELAEQEIAVALQDVGISLVNNYTKQEVAYIGITSSDVVWETKPKKKARWKPMSVKHTEKLEREFKEYTESSPSEDKVIQLDTNVPVRLTPTGHNMKILQPHVIALRRNYLPALKVEYNTSAHQSSFRIQIYRIQIQNQIHGAVFPFVFYPVKPPKSVTMDSAPKPFTDVSIVMRSAGHSQISRIKYFKVLIQEMDLRLDLGFIYALTDLMTEAEVTENTEVELFHKDIEAFKEEYKTASLVDQSQVSLYEYFHISPIKLHLSVSLSSGREEAKDSKQNGGLIPVHSLNLLLKSIGATLTDVQDVVFKLAFFELNYQFHTTSDLQSEVIRHYSKQAIKQMYVLILGLDVLGNPFGLIREFSEGVEAFFYEPYQGAIQGPEEFVEGMALGLKALVGGAVGGLAGAASKITGAMAKGVAAMTMDEDYQQKRREAMNKQPAGFREGITRGGKGLVSGFVSGITGIVTKPIKGAQKGGAAGFFKGVGKGLVGAVARPTGGIIDMASSTFQGIKRATETSEVESLRPPRFFNEDGVIRPYRLRDGTGNQMLQVMENGRFAKYKYFTHVMINKTDMLMITRRGVLFVTKGTFGQLTCEWQYSFDEFTKEPFIVHGRRLRIEAKERVKSVFHAREFGKIINFKTPEDARWILTKLQEAREPSPSL

4) >gi|58530840|ref|NP_004406.2| desmoplakin isoform I [Homo sapiens] (331.6 kD)

MSCNGGSHPRINTLGRMIRAESGPDLRYEVTSGGGGTSRMYYSRRGVITDQNSDGYCQTGTMSRHQNQNTIQELLQNCSDCLMRAELIVQPELKYGDGIQLTRSRELDECFAQANDQMEILDSLIREMRQMGQPCDAYQKRLLQLQEQMRALYKAISVPRVRRASSKGGGGYTCQSGSGWDEFTKHVTSECLGWMRQQRAEMDMVAWGVDLASVEQHINSHRGIHNSIGDYRWQLDKIKADLREKSAIYQLEEEYENLLKASFERMDHLRQLQNIIQATSREIMWINDCEEEELLYDWSDKNTNIAQKQEAFSIRMSQLEVKEKELNKLKQESDQLVLNQHPASDKIEAYMDTLQTQWSWILQITKCIDVHLKENAAYFQFFEEAQSTEAYLKGLQDSIRKKYPCDKNMPLQHLLEQIKELEKEREKILEYKRQVQNLVNKSKKIVQLKPRNPDYRSNKPIILRALCDYKQDQKIVHKGDECILKDNNERSKWYVTGPGGVDMLVPSVGLIIPPPNPLAVDLSCKIEQYYEAILALWNQLYINMKSLVSWHYCMIDIEKIRAMTIAKLKTMRQEDYMKTIADLELHYQEFIRNSQGSEMFGDDDKRKIQSQFTDAQKHYQTLVIQLPGYPQHQTVTTTEITHHGTCQDVNHNKVIETNRENDKQETWMLMELQKIRRQIEHCEGRMTLKNLPLADQGSSHHITVKINELKSVQNDSQAIAEVLNQLKDMLANFRGSEKYCYLQNEVFGLFQKLENINGVTDGYLNSLCTVRALLQAILQTEDMLKVYEARLTEEETVCLDLDKVEAYRCGLKKIKNDLNLKKSLLATMKTELQKAQQIHSQTSQQYPLYDLDLGKFGEKVTQLTDRWQRIDKQIDFRLWDLEKQIKQLRNYRDNYQAFCKWLYDAKRRQDSLESMKFGDSNTVMRFLNEQKNLHSEISGKRDKSEEVQKIAELCANSIKDYELQLASYTSGLETLLNIPIKRTMIQSPSGVILQEAADVHARYIELLTRSGDYYRFLSEMLKSLEDLKLKNTKIEVLEEELRLARDANSENCNKNKFLDQNLQKYQAECSQFKAKLASLEELKRQAELDGKSAKQNLDKCYGQIKELNEKITRLTYEIEDEKRRRKSVEDRFDQQKNDYDQLQKARQCEKENLGWQKLESEKAIKEKEYEIERLRVLLQEEGTRKREYENELAKVRNHYNEEMSNLRNKYETEINITKTTIKEISMQKEDDSKNLRNQLDRLSRENRDLKDEIVRLNDSILQATEQRRRAEENALQQKACGSEIMQKKQHLEIELKQVMQQRSEDNARHKQSLEEAAKTIQDKNKEIERLKAEFQEEAKRRWEYENELSKVRNNYDEEIISLKNQFETEINITKTTIHQLTMQKEEDTSGYRAQIDNLTRENRSLSEEIKRLKNTLTQTTENLRRVEEDIQQQKATGSEVSQRKQQLEVELRQVTQMRTEESVRYKQSLDDAAKTIQDKNKEIERLKQLIDKETNDRKCLEDENARLQRVQYDLQKANSSATETINKLKVQEQELTRLRIDYERVSQERTVKDQDITRFQNSLKELQLQKQKVEEELNRLKRTASEDSCKRKKLEEELEGMRRSLKEQAIKITNLTQQLEQASIVKKRSEDDLRQQRDVLDGHLREKQRTQEELRRLSSEVEALRRQLLQEQESVKQAHLRNEHFQKAIEDKSRSLNESKIEIERLQSLTENLTKEHLMLEEELRNLRLEYDDLRRGRSEADSDKNATILELRSQLQISNNRTLELQGLINDLQRERENLRQEIEKFQKQALEASNRIQESKNQCTQVVQERESLLVKIKVLEQDKARLQRLEDELNRAKSTLEAETRVKQRLECEKQQIQNDLNQWKTQYSRKEEAIRKIESEREKSEREKNSLRSEIERLQAEIKRIEERCRRKLEDSTRETQSQLETERSRYQREIDKLRQRPYGSHRETQTECEWTVDTSKLVFDGLRKKVTAMQLYECQLIDKTTLDKLLKGKKSVEEVASEIQPFLRGAGSIAGASASPKEKYSLVEAKRKKLISPESTVMLLEAQAATGGIIDPHRNEKLTVDSAIARDLIDFDDRQQIYAAEKAITGFDDPFSGKTVSVSEAIKKNLIDRETGMRLLEAQIASGGVVDPVNSVFLPKDVALARGLIDRDLYRSLNDPRDSQKNFVDPVTKKKVSYVQLKERCRIEPHTGLLLLSVQKRSMSFQGIRQPVTVTELVDSGILRPSTVNELESGQISYDEVGERIKDFLQGSSCIAGIYNETTKQKLGIYEAMKIGLVRPGTALELLEAQAATGFIVDPVSNLRLPVEEAYKRGLVGIEFKEKLLSAERAVTGYNDPETGNIISLFQAMNKELIEKGHGIRLLEAQIATGGIIDPKESHRLPVDIAYKRGYFNEELSEILSDPSDDTKGFFDPNTEENLTYLQLKERCIKDEETGLCLLPLKEKKKQVQTSQKNTLRKRRVVIVDPETNKEMSVQEAYKKGLIDYETFKELCEQECEWEEITITGSDGSTRVVLVDRKTGSQYDIQDAIDKGLVDRKFFDQYRSGSLSLTQFADMISLKNGVGTSSSMGSGVSDDVFSSSRHESVSKISTISSVRNLTIRSSSFSDTLEESSPIAAIFDTENLEKISITEGIERGIVDSITGQRLLEAQACTGGIIHPTTGQKLSLQDAVSQGVIDQDMATRLKPAQKAFIGFEGVKGKKKMSAAEAVKEKWLPYEAGQRFLEFQYLTGGLVDPEVHGRISTEEAIRKGFIDGRAAQRLQDTSSYAKILTCPKTKLKISYKDAINRSMVEDITGLRLLEAASVSSKGLPSPYNMSSAPGSRSGSRSGSRSGSRSGSRSGSRRGSFDATGNSSYSYSYSFSSSSIGH

5) >gi|54607053|ref|NP_006827.1| translational activator GCN1 [Homo sapiens] (292.6 kD)

MAADTQVSETLKRFAGKVTTASVKERREILSELGKCVAGKDLPEGAVKGLCKLFCLTLHRYRDAASRRALQAAIQQLAEAQPEATAKNLLHSLQSSGIGSKAGVPSKSSGSAALLALTWTCLLVRIVFPSRAKRQGDIWNKLVEVQCLLLLEVLGGSHKHAVDGAVKKLTKLWKENPGLVEQYLSAILSLEPNQNYAGMLGLLVQFCTSHKEMDVVSQHKSALLDFYMKNILMSKVKPPKYLLDSCAPLLRYLSHSEFKDLILPTIQKSLLRSPENVIETISSLLASVTLDLSQYAMDIVKGLAGHLKSNSPRLMDEAVLALRNLARQCSDSSAMESLTKHLFAILGGSEGKLTVVAQKMSVLSGIGSVSHHVVSGPSSQVLNGIVAELFIPFLQQEVHEGTLVHAVSVLALWCNRFTMEVPKKLTEWFKKAFSLKTSTSAVRHAYLQCMLASYRGDTLLQALDLLPLLIQTVEKAASQSTQVPTITEGVAAALLLLKLSVADSQAEAKLSSFWQLIVDEKKQVFTSEKFLVMASEDALCTVLHLTERLFLDHPHRLTGNKVQQYHRALVAVLLSRTWHVRRQAQQTVRKLLSSLGGFKLAHGLLEELKTVLSSHKVLPLEALVTDAGEVTEAGKAYVPPRVLQEALCVISGVPGLKGDVTDTEQLAQEMLIISHHPSLVAVQSGLWPALLARMKIDPEAFITRHLDQIIPRMTTQSPLNQSSMNAMGSLSVLSPDRVLPQLISTITASVQNPALRLVTREEFAIMQTPAGELYDKSIIQSAQQDSIKKANMKRENKAYSFKEQIIELELKEEIKKKKGIKEEVQLTSKQKEMLQAQLDREAQVRRRLQELDGELEAALGLLDIILAKNPSGLTQYIPVLVDSFLPLLKSPLAAPRIKNPFLSLAACVMPSRLKALGTLVSHVTLRLLKPECVLDKSWCQEELSVAVKRAVMLLHTHTITSRVGKGEPGAAPLSAPAFSLVFPFLKMVLTEMPHHSEEEEEWMAQILQILTVQAQLRASPNTPPGRVDENGPELLPRVAMLRLLTWVIGTGSPRLQVLASDTLTTLCASSSGDDGCAFAEQEEVDVLLCALQSPCASVRETVLRGLMELHMVLPAPDTDEKNGLNLLRRLWVVKFDKEEEIRKLAERLWSMMGLDLQPDLCSLLIDDVIYHEAAVRQAGAEALSQAVARYQRQAAEVMGRLMEIYQEKLYRPPPVLDALGRVISESPPDQWEARCGLALALNKLSQYLDSSQVKPLFQFFVPDALNDRHPDVRKCMLDAALATLNTHGKENVNSLLPVFEEFLKNAPNDASYDAVRQSVVVLMGSLAKHLDKSDPKVKPIVAKLIAALSTPSQQVQESVASCLPPLVPAIKEDAGGMIQRLMQQLLESDKYAERKGAAYGLAGLVKGLGILSLKQQEMMAALTDAIQDKKNFRRREGALFAFEMLCTMLGKLFEPYVVHVLPHLLLCFGDGNQYVREAADDCAKAVMSNLSAHGVKLVLPSLLAALEEESWRTKAGSVELLGAMAYCAPKQLSSCLPNIVPKLTEVLTDSHVKVQKAGQQALRQIGSVIRNPEILAIAPVLLDALTDPSRKTQKCLQTLLDTKFVHFIDAPSLALIMPIVQRAFQDRSTDTRKMAAQIIGNMYSLTDQKDLAPYLPSVTPGLKASLLDPVPEVRTVSAKALGAMVKGMGESCFEDLLPWLMETLTYEQSSVDRSGAAQGLAEVMAGLGVEKLEKLMPEIVATASKVDIAPHVRDGYIMMFNYLPITFGDKFTPYVGPIIPCILKALADENEFVRDTALRAGQRVISMYAETAIALLLPQLEQGLFDDLWRIRFSSVQLLGDLLFHISGVTGKMTTETASEDDNFGTAQSNKAIITALGVERRNRVLAGLYMGRSDTQLVVRQASLHVWKIVVSNTPRTLREILPTLFGLLLGFLASTCADKRTIAARTLGDLVRKLGEKILPEIIPILEEGLRSQKSDERQGVCIGLSEIMKSTSRDAVLYFSESLVPTARKALCDPLEEVREAAAKTFEQLHSTIGHQALEDILPFLLKQLDDEEVSEFALDGLKQVMAIKSRVVLPYLVPKLTTPPVNTRVLAFLSSVAGDALTRHLGVILPAVMLALKEKLGTPDEQLEMANCQAVILSVEDDTGHRIIIEDLLEATRSPEVGMRQAAAIILNIYCSRSKADYTSHLRSLVSGLIRLFNDSSPVVLEESWDALNAITKKLDAGNQLALIEELHKEIRLIGNESKGEHVPGFCLPKKGVTSILPVLREGVLTGSPEQKEEAAKALGLVIRLTSADALRPSVVSITGPLIRILGDRFSWNVKAALLETLSLLLAKVGIALKPFLPQLQTTFTKALQDSNRGVRLKAADALGKLISIHIKVDPLFTELLNGIRAMEDPGVRDTMLQALRFVIQGAGAKVDAVIRKNIVSLLLSMLGHDEDNTRISSAGCLGELCAFLTEEELSAVLQQCLLADVSGIDWMVRHGRSLALSVAVNVAPGRLCAGRYSSDVQEMILSSATADRIPIAVSGVRGMGFLMRHHIETGGGQLPAKLSSLFVKCLQNPSSDIRLVAEKMIWWANKDPLPPLDPQAIKPILKALLDNTKDKNTVVRAYSDQAIVNLLKMRQGEEVFQSLSKILDVASLEVLNEVNRRSLKKLASQADSTEQVDDTILT

6) >gi|57864582|ref|NP_001009931.1| hornerin [Homo sapiens] (282.2 kD)

MPKLLQGVITVIDVFYQYATQHGEYDTLNKAELKELLENEFHQILKNPNDPDTVDIILQSLDRDHNKKVDFTEYLLMIFKLVQARNKIIGKDYCQVSGSKLRDDTHQHQEEQEETEKEENKRQESSFSHSSWSAGENDSYSRNVRGSLKPGTESISRRLSFQRDFSGQHNSYSGQSSSYGEQNSDSHQSSGRGQCGSGSGQSPNYGQHGSGSGQSSSNDTHGSGSGQSSGFSQHKSSSGQSSGYSQHGSGSGHSSGYGQHGSRSGQSSRGERHRSSSGSSSSYGQHGSGSRQSLGHGRQGSGSRQSPSHVRHGSGSGHSSSHGQHGSGSSYSYSRGHYESGSGQTSGFGQHESGSGQSSGYSKHGSGSGHSSSQGQHGSTSGQASSSGQHGSSSRQSSSYGQHESASRHSSGRGQHSSGSGQSPGHGQRGSGSGQSPSSGQHGTGFGRSSSSGPYVSGSGYSSGFGHHESSSEHSSGYTQHGSGSGHSSGHGQHGSRSGQSSRGERQGSSAGSSSSYGQHGSGSRQSLGHSRHGSGSGQSPSPSRGRHESGSRQSSSYGPHGYGSGRSSSRGPYESGSGHSSGLGHQESRSGQSSGYGQHGSSSGHSSTHGQHGSTSGQSSSCGQHGATSGQSSSHGQHGSGSSQSSRYGQQGSGSGQSPSRGRHGSDFGHSSSYGQHGSGSGWSSSNGPHGSVSGQSSGFGHKSGSGQSSGYSQHGSGSSHSSGYRKHGSRSGQSSRSEQHGSSSGLSSSYGQHGSGSHQSSGHGRQGSGSGHSPSRVRHGSSSGHSSSHGQHGSGTSCSSSCGHYESGSGQASGFGQHESGSGQGYSQHGSASGHFSSQGRHGSTSGQSSSSGQHDSSSGQSSSYGQHESASHHASGRGRHGSGSGQSPGHGQRGSGSGQSPSYGRHGSGSGRSSSSGRHGSGSGQSSGFGHKSSSGQSSGYTQHGSGSGHSSSYEQHGSRSGQSSRSEQHGSSSGSSSSYGQHGSGSRQSLGHGQHGSGSGQSPSPSRGRHGSGSGQSSSYGPYRSGSGWSSSRGPYESGSGHSSGLGHRESRSGQSSGYGQHGSSSGHSSTHGQHGSTSGQSSSCGQHGASSGQSSSHGQHGSGSSQSSGYGRQGSGSGQSPGHGQRGSGSRQSPSYGRHGSGSGRSSSSGQHGSGLGESSGFGHHESSSGQSSSYSQHGSGSGHSSGYGQHGSRSGQSSRGERHGSSSGSSSHYGQHGSGSRQSSGHGRQGSGSGHSPSRGRHGSGLGHSSSHGQHGSGSGRSSSRGPYESRSGHSSVFGQHESGSGHSSAYSQHGSGSGHFCSQGQHGSTSGQSSTFDQEGSSTGQSSSYGHRGSGSSQSSGYGRHGAGSGQSPSRGRHGSGSGHSSSYGQHGSGSGWSSSSGRHGSGSGQSSGFGHHESSSWQSSGCTQHGSGSGHSSSYEQHGSRSGQSSRGERHGSSSGSSSSYGQHGSGSRQSLGHGQHGSGSGQSPSPSRGRHGSGSGQSSSYSPYGSGSGWSSSRGPYESGSSHSSGLGHRESRSGQSSGYGQHGSSSGHSSTHGQHGSTSGQSSSCGQHGASSGQSSSHGQHGSGSSQSSGYGRQGSGSGQSPGHGQRGSGSRQSPSYGRHGSGSGRSSSSGQHGSGLGESSGFGHHESSSGQSSSYSQHGSGSGHSSGYGQHGSRSGQSSRGERHGSSSRSSSRYGQHGSGSRQSSGHGRQGSGSGQSPSRGRHGSGLGHSSSHGQHGSGSGRSSSRGPYESRSGHSSVFGQHESGSGHSSAYSQHGSGSGHFCSQGQHGSTSGQSSTFDQEGSSTGQSSSHGQHGSGSSQSSSYGQQGSGSGQSPSRGRHGSGSGHSSSYGQHGSGSGWSSSSGRHGSGSGQSSGFGHHESSSWQSSGYTQHGSGSGHSSSYEQHGSRSGQSSRGEQHGSSSGSSSSYGQHGSGSRQSLGHGQHGSGSGQSPSPSRGRHGSGSGQSSSYGPYGSGSGWSSSRGPYESGSGHSSGLGHRESRSGQSSGYGQHGSSSGHSSTHGQHGSASGQSSSCGQHGASSGQSSSHGQHGSGSSQSSGYGRQGSGSGQSPGHGQRGSGSRQSPSYGRHGSGSGRSSSSGQHGPGLGESSGFGHHESSSGQSSSYSQHGSGSGHSSGYGQHGSRSGQSSRGERHGSSSGSSSRYGQHGSGSRQSSGHGRQGSGSGHSPSRGRHGSGSGHSSSHGQHGSGSGRSSSRGPYESRSGHSSVFGQHESGSGHSSAYSQHGSGSGHFCSQGQHGSTSGQSSTFDQEGSSTGQSSSHGQHGSGSSQSSSYGQQGSGSGQSPSRGRHGSGSGHSSSYGQHGSGSGWSSSSGRHGSGSGQSSGFGHHESSSWQSSGYTQHGSGSGHSSSYEQHGSRSGQSSRGERHGSSSGSSSSYGQHGSGSRQSLGHGQHGSGSGQSPSPSRGRHGSGSGQSSSYSPYGSGSGWSSSRGPYESGSGHSSGLGHRESRSGQSSGYGQHGSSSGHSSTHGQHGSTSGQSSSCGQHGASSGQSSSHGQHGSGSSQSSGYGRQGSGSGQSPGHGQRGSGSRQSPSYGRHGSGSGRSSSSGQHGSGLGESSGFGHHESSSGQSSSYSQHGSGSGHSSGYGQHGSRSGQSSRGERHGSSSGSSSHYGQHGSGSRQSSGHGRQGSGSGQSPSRGRHGSGLGHSSSHGQHGSGSGRSSSRGPYESRLGHSSVFGQHESGSGHSSAYSQHGSGSGHFCSQGQHGSTSGQSSTFDQEGSSTGQSSSYGHRGSGSSQSSGYGRHGAGSGQSLSHGRHGSGSGQSSSYGQHGSGSGQSSGYSQHGSGSGQDGYSYCKGGSNHDGGSSGSYFLSFPSSTSPYEYVQEQRCYFYQ

7) >gi|160420317|ref|NP_001104026.1| filamin-A isoform 2 [Homo sapiens] (280.6 kD)

MSSSHSRAGQSAAGAAPGGGVDTRDAEMPATEKDLAEDAPWKKIQQNTFTRWCNEHLKCVSKRIANLQTDLSDGLRLIALLEVLSQKKMHRKHNQRPTFRQMQLENVSVALEFLDRESIKLVSIDSKAIVDGNLKLILGLIWTLILHYSISMPMWDEEEDEEAKKQTPKQRLLGWIQNKLPQLPITNFSRDWQSGRALGALVDSCAPGLCPDWDSWDASKPVTNAREAMQQADDWLGIPQVITPEEIVDPNVDEHSVMTYLSQFPKAKLKPGAPLRPKLNPKKARAYGPGIEPTGNMVKKRAEFTVETRSAGQGEVLVYVEDPAGHQEEAKVTANNDKNRTFSVWYVPEVTGTHKVTVLFAGQHIAKSPFEVYVDKSQGDASKVTAQGPGLEPSGNIANKTTYFEIFTAGAGTGEVEVVIQDPMGQKGTVEPQLEARGDSTYRCSYQPTMEGVHTVHVTFAGVPIPRSPYTVTVGQACNPSACRAVGRGLQPKGVRVKETADFKVYTKGAGSGELKVTVKGPKGEERVKQKDLGDGVYGFEYYPMVPGTYIVTITWGGQNIGRSPFEVKVGTECGNQKVRAWGPGLEGGVVGKSADFVVEAIGDDVGTLGFSVEGPSQAKIECDDKGDGSCDVRYWPQEAGEYAVHVLCNSEDIRLSPFMADIRDAPQDFHPDRVKARGPGLEKTGVAVNKPAEFTVDAKHGGKAPLRVQVQDNEGCPVEALVKDNGNGTYSCSYVPRKPVKHTAMVSWGGVSIPNSPFRVNVGAGSHPNKVKVYGPGVAKTGLKAHEPTYFTVDCAEAGQGDVSIGIKCAPGVVGPAEADIDFDIIRNDNDTFTVKYTPRGAGSYTIMVLFADQATPTSPIRVKVEPSHDASKVKAEGPGLSRTGVELGKPTHFTVNAKAAGKGKLDVQFSGLTKGDAVRDVDIIDHHDNTYTVKYTPVQQGPVGVNVTYGGDPIPKSPFSVAVSPSLDLSKIKVSGLGEKVDVGKDQEFTVKSKGAGGQGKVASKIVGPSGAAVPCKVEPGLGADNSVVRFLPREEGPYEVEVTYDGVPVPGSPFPLEAVAPTKPSKVKAFGPGLQGGSAGSPARFTIDTKGAGTGGLGLTVEGPCEAQLECLDNGDGTCSVSYVPTEPGDYNINILFADTHIPGSPFKAHVVPCFDASKVKCSGPGLERATAGEVGQFQVDCSSAGSAELTIEICSEAGLPAEVYIQDHGDGTHTITYIPLCPGAYTVTIKYGGQPVPNFPSKLQVEPAVDTSGVQCYGPGIEGQGVFREATTEFSVDARALTQTGGPHVKARVANPSGNLTETYVQDRGDGMYKVEYTPYEEGLHSVDVTYDGSPVPSSPFQVPVTEGCDPSRVRVHGPGIQSGTTNKPNKFTVETRGAGTGGLGLAVEGPSEAKMSCMDNKDGSCSVEYIPYEAGTYSLNVTYGGHQVPGSPFKVPVHDVTDASKVKCSGPGLSPGMVRANLPQSFQVDTSKAGVAPLQVKVQGPKGLVEPVDVVDNADGTQTVNYVPSREGPYSISVLYGDEEVPRSPFKVKVLPTHDASKVKASGPGLNTTGVPASLPVEFTIDAKDAGEGLLAVQITDPEGKPKKTHIQDNHDGTYTVAYVPDVTGRYTILIKYGGDEIPFSPYRVRAVPTGDASKCTVTVSIGGHGLGAGIGPTIQIGEETVITVDTKAAGKGKVTCTVCTPDGSEVDVDVVENEDGTFDIFYTAPQPGKYVICVRFGGEHVPNSPFQVTALAGDQPSVQPPLRSQQLAPQYTYAQGGQQTWAPERPLVGVNGLDVTSLRPFDLVIPFTIKKGEITGEVRMPSGKVAQPTITDNKDGTVTVRYAPSEAGLHEMDIRYDNMHIPGSPLQFYVDYVNCGHVTAYGPGLTHGVVNKPATFTVNTKDAGEGGLSLAIEGPSKAEISCTDNQDGTCSVSYLPVLPGDYSILVKYNEQHVPGSPFTARVTGDDSMRMSHLKVGSAADIPINISETDLSLLTATVVPPSGREEPCLLKRLRNGHVGISFVPKETGEHLVHVKKNGQHVASSPIPVVISQSEIGDASRVRVSGQGLHEGHTFEPAEFIIDTRDAGYGGLSLSIEGPSKVDINTEDLEDGTCRVTYCPTEPGNYIINIKFADQHVPGSPFSVKVTGEGRVKESITRRRRAPSVANVGSHCDLSLKIPEISIQDMTAQVTSPSGKTHEAEIVEGENHTYCIRFVPAEMGTHTVSVKYKGQHVPGSPFQFTVGPLGEGGAHKVRAGGPGLERAEAGVPAEFSIWTREAGAGGLAIAVEGPSKAEISFEDRKDGSCGVAYVVQEPGDYEVSVKFNEEHIPDSPFVVPVASPSGDARRLTVSSLQESGLKVNQPASFAVSLNGAKGAIDAKVHSPSGALEECYVTEIDQDKYAVRFIPRENGVYLIDVKFNGTHIPGSPFKIRVGEPGHGGDPGLVSAYGAGLEGGVTGNPAEFVVNTSNAGAGALSVTIDGPSKVKMDCQECPEGYRVTYTPMAPGSYLISIKYGGPYHIGGSPFKAKVTGPRLVSNHSLHETSSVFVDSLTKATCAPQHGAPGPGPADASKVVAKGLGLSKAYVGQKSSFTVDCSKAGNNMLLVGVHGPRTPCEEILVKHVGSRLYSVSYLLKDKGEYTLVVKWGDEHIPGSPYRVVVP

8) >gi|190194412|ref|NP_004230.2| thyroid receptor-interacting protein 11 [Homo sapiens] (227.5 kD)

MSSWLGGLGSGLGQSLGQVGGSLASLTGQISNFTKDMLMEGTEEVEAELPDSRTKEIEAIHAILRSENERLKKLCTDLEEKHEASEIQIKQQSTSYRNQLQQKEVEISHLKARQIALQDQLLKLQSAAQSVPSGAGVPATTASSSFAYGISHHPSAFHDDDMDFGDIISSQQEINRLSNEVSRLESEVGHWRHIAQTSKAQGTDNSDQSEICKLQNIIKELKQNRSQEIDDHQHEMSVLQNAHQQKLTEISRRHREELSDYEERIEELENLLQQGGSGVIETDLSKIYEMQKTIQVLQIEKVESTKKMEQLEDKIKDINKKLSSAENDRDILRREQEQLNVEKRQIMEECENLKLECSKLQPSAVKQSDTMTEKERILAQSASVEEVFRLQQALSDAENEIMRLSSLNQDNSLAEDNLKLKMRIEVLEKEKSLLSQEKEELQMSLLKLNNEYEVIKSTATRDISLDSELHDLRLNLEAKEQELNQSISEKETLIAEIEELDRQNQEATKHMILIKDQLSKQQNEGDSIISKLKQDLNDEKKRVHQLEDDKMDITKELDVQKEKLIQSEVALNDLHLTKQKLEDKVENLVDQLNKSQESNVSIQKENLELKEHIRQNEEELSRIRNELMQSLNQDSNSNFKDTLLKEREAEVRNLKQNLSELEQLNENLKKVAFDVKMENEKLVLACEDVRHQLEECLAGNNQLSLEKNTIVETLKMEKGEIEAELCWAKKRLLEEANKYEKTIEELSNARNLNTSALQLEHEHLIKLNQKKDMEIAELKKNIEQMDTDHKETKDVLSSSLEEQKQLTQLINKKEIFIEKLKERSSKLQEELDKYSQALRKNEILRQTIEEKDRSLGSMKEENNHLQEELERLREEQSRTAPVADPKTLDSVTELASEVSQLNTIKEHLEEEIKHHQKIIEDQNQSKMQLLQSLQEQKKEMDEFRYQHEQMNATHTQLFLEKDEEIKSLQKTIEQIKTQLHEERQDIQTDNSDIFQETKVQSLNIENGSEKHDLSKAETERLVKGIKERELEIKLLNEKNISLTKQIDQLSKDEVGKLTQIIQQKDLEIQALHARISSTSHTQDVVYLQQQLQAYAMEREKVFAVLNEKTRENSHLKTEYHKMMDIVAAKEAALIKLQDENKKLSTRFESSGQDMFRETIQNLSRIIREKDIEIDALSQKCQTLLAVLQTSSTGNEAGGVNSNQFEELLQERDKLKQQVKKMEEWKQQVMTTVQNMQHESAQLQEELHQLQAQVLVDSDNNSKLQVDYTGLIQSYEQNETKLKNFGQELAQVQHSIGQLCNTKDLLLGKLDIISPQLSSASLLTPQSAECLRASKSEVLSESSELLQQELEELRKSLQEKDATIRTLQENNHRLSDSIAATSELERKEHEQTDSEIKQLKEKQDVLQKLLKEKDLLIKAKSDQLLSSNENFTNKVNENELLRQAVTNLKERILILEMDIGKLKGENEKIVETYRGKETEYQALQETNMKFSMMLREKEFECHSMKEKALAFEQLLKEKEQGKTGELNQLLNAVKSMQEKTVVFQQERDQVMLALKQKQMENTALQNEVQRLRDKEFRSNQELERLRNHLLESEDSYTREALAAEDREAKLRKKVTVLEEKLVSSSNAMENASHQASVQVESLQEQLNVVSKQRDETALQLSVSQEQVKQYALSLANLQMVLEHFQQEEKAMYSAELEKQKQLIAEWKKNAENLEGKVISLQECLDEANAALDSASRLTEQLDVKEEQIEELKRQNELRQEMLDDVQKKLMSLANSSEGKVDKVLMRNLFIGHFHTPKNQRHEVLRLMGSILGVRREEMEQLFHDDQGGVTRWMTGWLGGGSKSVPNTPLRPNQQSVVNSSFSELFVKFLETESHPSIPPPKLSVHDMKPLDSPGRRKRDTNAPESFKDTAESRSGRRTDVNPFLAPRSAAVPLINPAGLGPGGPGHLLLKPISDVLPTFTPLPALPDNSAGVVLKDLLKQ

9) >gi|302699237|ref|NP_886553.3| eukaryotic translation initiation factor 4 gamma 1 isoform 1 [Homo sapiens] (175.4 kD)

MNKAPQSTGPPPAPSPGLPQPAFPPGQTAPVVFSTPQATQMNTPSQPRQHFYPSRAQPPSSAASRVQSAAPARPGPAAHVYPAGSQVMMIPSQISYPASQGAYYIPGQGRSTYVVPTQQYPVQPGAPGFYPGASPTEFGTYAGAYYPAQGVQQFPTGVAPAPVLMNQPPQIAPKRERKTIRIRDPNQGGKDITEEIMSGARTASTPTPPQTGGGLEPQANGETPQVAVIVRPDDRSQGAIIADRPGLPGPEHSPSESQPSSPSPTPSPSPVLEPGSEPNLAVLSIPGDTMTTIQMSVEESTPISRETGEPYRLSPEPTPLAEPILEVEVTLSKPVPESEFSSSPLQAPTPLASHTVEIHEPNGMVPSEDLEPEVESSPELAPPPACPSESPVPIAPTAQPEELLNGAPSPPAVDLSPVSEPEEQAKEVTASMAPPTIPSATPATAPSATSPAQEEEMEEEEEEEEGEAGEAGEAESEKGGEELLPPESTPIPANLSQNLEAAAATQVAVSVPKRRRKIKELNKKEAVGDLLDAFKEANPAVPEVENQPPAGSNPGPESEGSGVPPRPEEADETWDSKEDKIHNAENIQPGEQKYEYKSDQWKPLNLEEKKRYDREFLLGFQFIFASMQKPEGLPHISDVVLDKANKTPLRPLDPTRLQGINCGPDFTPSFANLGRTTLSTRGPPRGGPGGELPRGPQAGLGPRRSQQGPRKEPRKIIATVLMTEDIKLNKAEKAWKPSSKRTAADKDRGEEDADGSKTQDLFRRVRSILNKLTPQMFQQLMKQVTQLAIDTEERLKGVIDLIFEKAISEPNFSVAYANMCRCLMALKVPTTEKPTVTVNFRKLLLNRCQKEFEKDKDDDEVFEKKQKEMDEAATAEERGRLKEELEEARDIARRRSLGNIKFIGELFKLKMLTEAIMHDCVVKLLKNHDEESLECLCRLLTTIGKDLDFEKAKPRMDQYFNQMEKIIKEKKTSSRIRFMLQDVLDLRGSNWVPRRGDQGPKTIDQIHKEAEMEEHREHIKVQQLMAKGSDKRRGGPPGPPISRGLPLVDDGGWNTVPISKGSRPIDTSRLTKITKPGSIDSNNQLFAPGGRLSWGKGSSGGSGAKPSDAASEAARPATSTLNRFSALQQAVPTESTDNRRVVQRSSLSRERGEKAGDRGDRLERSERGGDRGDRLDRARTPATKRSFSKEVEERSRERPSQPEGLRKAASLTEDRDRGRDAVKREAALPPVSPLKAALSEEELEKKSKAIIEEYLHLNDMKEAVQCVQELASPSLLFIFVRHGVESTLERSAIAREHMGQLLHQLLCAGHLSTAQYYQGLYEILELAEDMEIDIPHVWLYLAELVTPILQEGGVPMGELFREITKPLRPLGKAASLLLEILGLLCKSMGPKKVGTLWREAGLSWKEFLPEGQDIGAFVAEQKVEYTLGEESEAPGQRALPSEELNRQLEKLLKEGSSNQRVFDWIEANLSEQQIVSNTLVRALMTAVCYSAIIFETPLRVDVAVLKARAKLLQKYLCDEQKELQALYALQALVVTLEQPPNLLRMFFDALYDEDVVKEDAFYSWESSKDPAEQQGKGVALKSVTAFFKWLREAEEESDHN

10) >gi|28559039|ref|NP_004765.2| mediator of RNA polymerase II transcription subunit 1 [Homo sapiens] (168.4 kD)

MKAQGETEESEKLSKMSSLLERLHAKFNQNRPWSETIKLVRQVMEKRVVMSSGGHQHLVSCLETLQKALKVTSLPAMTDRLESIARQNGLGSHLSASGTECYITSDMFYVEVQLDPAGQLCDVKVAHHGENPVSCPELVQQLREKNFDEFSKHLKGLVNLYNLPGDNKLKTKMYLALQSLEQDLSKMAIMYWKATNAGPLDKILHGSVGYLTPRSGGHLMNLKYYVSPSDLLDDKTASPIILHENNVSRSLGMNASVTIEGTSAVYKLPIAPLIMGSHPVDNKWTPSFSSITSANSVDLPACFFLKFPQPIPVSRAFVQKLQNCTGIPLFETQPTYAPLYELITQFELSKDPDPIPLNHNMRFYAALPGQQHCYFLNKDAPLPDGRSLQGTLVSKITFQHPGRVPLILNLIRHQVAYNTLIGSCVKRTILKEDSPGLLQFEVCPLSESRFSVSFQHPVNDSLVCVVMDVQDSTHVSCKLYKGLSDALICTDDFIAKVVQRCMSIPVTMRAIRRKAETIQADTPALSLIAETVEDMVKKNLPPASSPGYGMTTGNNPMSGTTTPTNTFPGGPITTLFNMSMSIKDRHESVGHGEDFSKVSQNPILTSLLQITGNGGSTIGSSPTPPHHTPPPVSSMAGNTKNHPMLMNLLKDNPAQDFSTLYGSSPLERQNSSSGSPRMEICSGSNKTKKKKSSRLPPEKPKHQTEDDFQRELFSMDVDSQNPIFDVNMTADTLDTPHITPAPSQCSTPPTTYPQPVPHPQPSIQRMVRLSSSDSIGPDVTDILSDIAEEASKLPSTSDDCPAIGTPLRDSSSSGHSQSTLFDSDVFQTNNNENPYTDPADLIADAAGSPSSDSPTNHFFHDGVDFNPDLLNSQSQSGFGEEYFDESSQSGDNDDFKGFASQALNTLGVPMLGGDNGETKFKGNNQADTVDFSIISVAGKALAPADLMEHHSGSQGPLLTTGDLGKEKTQKRVKEGNGTSNSTLSGPGLDSKPGKRSRTPSNDGKSKDKPPKRKKADTEGKSPSHSSSNRPFTPPTSTGGSKSPGSAGRSQTPPGVATPPIPKITIQIPKGTVMVGKPSSHSQYTSSGSVSSSGSKSHHSHSSSSSSSASTSGKMKSSKSEGSSSSKLSSSMYSSQGSSGSSQSKNSSQSGGKPGSSPITKHGLSSGSSSTKMKPQGKPSSLMNPSLSKPNISPSHSRPPGGSDKLASPMKPVPGTPPSSKAKSPISSGSGGSHMSGTSSSSGMKSSSGLGSSGSLSQKTPPSSNSCTASSSSFSSSGSSMSSSQNQHGSSKGKSPSRNKKPSLTAVIDKLKHGVVTSGPGGEDPLDGQMGVSTNSSSHPMSSKHNMSGGEFQGKREKSDKDKSKVSTSGSSVDSSKKTSESKNVGSTGVAKIIISKHDGGSPSIKAKVTLQKPGESSGEGLRPQMASSKNYGSPLISGSTPKHERGSPSHSKSPAYTPQNLDSESESGSSIAEKSYQNSPSSDDGIRPLPEYSTEKHKKHKKEKKKVKDKDRDRDRDKDRDKKKSHSIKPESWSKSPISSDQSLSMTSNTILSADRPSRLSPDFMIGEEDDDLMDVALIGN

11) >gi|31621305|ref|NP_573566.2| leucine-rich PPR motif-containing protein, mitochondrial precursor [Homo sapiens] (157.8 kD)

MAALLRSARWLLRAGAAPRLPLSLRLLPGGPGRLHAASYLPAARAGPVAGGLLSPARLYAIAAKEKDIQEESTFSSRKISNQFDWALMRLDLSVRRTGRIPKKLLQKVFNDTCRSGGLGGSHALLLLRSCGSLLPELKLEERTEFAHRIWDTLQKLGAVYDVSHYNALLKVYLQNEYKFSPTDFLAKMEEANIQPNRVTYQRLIASYCNVGDIEGASKILGFMKTKDLPVTEAVFSALVTGHARAGDMENAENILTVMRDAGIEPGPDTYLALLNAYAEKGDIDHVKQTLEKVEKSELHLMDRDLLQIIFSFSKAGYPQYVSEILEKVTCERRYIPDAMNLILLLVTEKLEDVALQILLACPVSKEDGPSVFGSFFLQHCVTMNTPVEKLTDYCKKLKEVQMHSFPLQFTLHCALLANKTDLAKALMKAVKEEGFPIRPHYFWPLLVGRRKEKNVQGIIEILKGMQELGVHPDQETYTDYVIPCFDSVNSARAILQENGCLSDSDMFSQAGLRSEAANGNLDFVLSFLKSNTLPISLQSIRSSLLLGFRRSMNINLWSEITELLYKDGRYCQEPRGPTEAVGYFLYNLIDSMSDSEVQAKEEHLRQYFHQLEKMNVKIPENIYRGIRNLLESYHVPELIKDAHLLVESKNLDFQKTVQLTSSELESTLETLKAENQPIRDVLKQLILVLCSEENMQKALELKAKYESDMVTGGYAALINLCCRHDKVEDALNLKEEFDRLDSSAVLDTGKYVGLVRVLAKHGKLQDAINILKEMKEKDVLIKDTTALSFFHMLNGAALRGEIETVKQLHEAIVTLGLAEPSTNISFPLVTVHLEKGDLSTALEVAIDCYEKYKVLPRIHDVLCKLVEKGETDLIQKAMDFVSQEQGEMVMLYDLFFAFLQTGNYKEAKKIIETPGIRARSARLQWFCDRCVANNQVETLEKLVELTQKLFECDRDQMYYNLLKLYKINGDWQRADAVWNKIQEENVIPREKTLRLLAEILREGNQEVPFDVPELWYEDEKHSLNSSSASTTEPDFQKDILIACRLNQKKGAYDIFLNAKEQNIVFNAETYSNLIKLLMSEDYFTQAMEVKAFAETHIKGFTLNDAANSRLIITQVRRDYLKEAVTTLKTVLDQQQTPSRLAVTRVIQALAMKGDVENIEVVQKMLNGLEDSIGLSKMVFINNIALAQIKNNNIDAAIENIENMLTSENKVIEPQYFGLAYLFRKVIEEQLEPAVEKISIMAERLANQFAIYKPVTDFFLQLVDAGKVDDARALLQRCGAIAEQTPILLLFLLRNSRKQGKASTVKSVLELIPELNEKEEAYNSLMKSYVSEKDVTSAKALYEHLTAKNTKLDDLFLKRYASLLKYAGEPVPFIEPPESFEFYAQQLRKLRENSS

12) >gi|24430149|ref|NP_705618.1| nuclear pore complex protein Nup155 isoform 1 [Homo sapiens] (155.1 kD)

MPSSLLGAAMPASTSAAALQEALENAGRLIDRQLQEDRMYPDLSELLMVSAPNNPTVSGMSDMDYPLQGPGLLSVPNLPEISSIRRVPLPPELVEQFGHMQCNCMMGVFPPISRAWLTIDSDIFMWNYEDGGDLAYFDGLSETILAVGLVKPKAGIFQPHVRHLLVLATPVDIVILGLSYANLQTGSGVLNDSLSGGMQLLPDPLYSLPTDNTYLLTITSTDNGRIFLAGKDGCLYEVAYQAEAGWFSQRCRKINHSKSSLSFLVPSLLQFTFSEDDPILQIAIDNSRNILYTRSEKGVIQVYDLGQDGQGMSRVASVSQNAIVSAAGNIARTIDRSVFKPIVQIAVIENSESLDCQLLAVTHAGVRLYFSTCPFRQPLARPNTLTLVHVRLPPGFSASSTVEKPSKVHRALYSKGILLMAASENEDNDILWCVNHDTFPFQKPMMETQMTAGVDGHSWALSAIDELKVDKIITPLNKDHIPITDSPVVVQQHMLPPKKFVLLSAQGSLMFHKLRPVDQLRHLLVSNVGGDGEEIERFFKLHQEDQACATCLILACSTAACDREVSAWATRAFFRYGGEAQMRFPTTLPPPSNVGPILGSPVYSSSPVPSGSPYPNPSFLGTPSHGIQPPAMSTPVCALGNPATQATNMSCVTGPEIVYSGKHNGICIYFSRIMGNIWDASLVVERIFKSGNREITAIESSVPCQLLESVLQELKGLQEFLDRNSQFAGGPLGNPNTTAKVQQRLIGFMRPENGNPQQMQQELQRKFHEAQLSEKISLQAIQQLVRKSYQALALWKLLCEHQFTIIVAELQKELQEQLKITTFKDLVIRDKELTGALIASLINCYIRDNAAVDGISLHLQDICPLLYSTDDAICSKANELLQRSRQVQNKTEKERMLRESLKEYQKISNQVDLSNVCAQYRQVRFYEGVVELSLTAAEKKDPQGLGLHFYKHGEPEEDIVGLQAFQERLNSYKCITDTLQELVNQSKAAPQSPSVPKKPGPPVLSSDPNMLSNEEAGHHFEQMLKLSQRSKDELFSIALYNWLIQVDLADKLLQVASPFLEPHLVRMAKVDQNRVRYMDLLWRYYEKNRSFSNAARVLSRLADMHSTEISLQQRLEYIARAILSAKSSTAISSIAADGEFLHELEEKMEVARIQLQIQETLQRQYSHHSSVQDAVSQLDSELMDITKLYGEFADPFKLAECKLAIIHCAGYSDPILVQTLWQDIIEKELSDSVTLSSSDRMHALSLKIVLLGKIYAGTPRFFPLDFIVQFLEQQVCTLNWDVGFVIQTMNEIGVPLPRLLEVYDQLFKSRDPFWNRMKKPLHLLDCIHVLLIRYVENPSQVLNCERRRFTNLCLDAVCGYLVELQSMSSSVAVQAITGNFKSLQAKLERLH

13) >gi|45827771|ref|NP_055144.3| enhancer of mRNA-decapping protein 4 [Homo sapiens] (151.6 kD)

MASCASIDIEDATQHLRDILKLDRPAGGPSAESPRPSSAYNGDLNGLLVPDPLCSGDSTSANKTGLRTMPPINLQEKQVICLSGDDSSTCIGILAKEVEIVASSDSSISSKARGSNKVKIQPVAKYDWEQKYYYGNLIAVSNSFLAYAIRAANNGSAMVRVISVSTSERTLLKGFTGSVADLAFAHLNSPQLACLDEAGNLFVWRLALVNGKIQEEILVHIRQPEGTPLNHFRRIIWCPFIPEESEDCCEESSPTVALLHEDRAEVWDLDMLRSSHSTWPVDVSQIKQGFIVVKGHSTCLSEGALSPDGTVLATASHDGYVKFWQIYIEGQDEPRCLHEWKPHDGRPLSCLLFCDNHKKQDPDVPFWRFLITGADQNRELKMWCTVSWTCLQTIRFSPDIFSSVSVPPSLKVCLDLSAEYLILSDVQRKVLYVMELLQNQEEGHACFSSISEFLLTHPVLSFGIQVVSRCRLRHTEVLPAEEENDSLGADGTHGAGAMESAAGVLIKLFCVHTKALQDVQIRFQPQLNPDVVAPLPTHTAHEDFTFGESRPELGSEGLGSAAHGSQPDLRRIVELPAPADFLSLSSETKPKLMTPDAFMTPSASLQQITASPSSSSSGSSSSSSSSSSSLTAVSAMSSTSAVDPSLTRPPEELTLSPKLQLDGSLTMSSSGSLQASPRGLLPGLLPAPADKLTPKGPGQVPTATSALSLELQEVEPLGLPQASPSRTRSPDVISSASTALSQDIPEIASEALSRGFGSSAPEGLEPDSMASAASALHLLSPRPRPGPELGPQLGLDGGPGDGDRHNTPSLLEAALTQEASTPDSQVWPTAPDITRETCSTLAESPRNGLQEKHKSLAFHRPPYHLLQQRDSQDASAEQSDHDDEVASLASASGGFGTKVPAPRLPAKDWKTKGSPRTSPKLKRKSKKDDGDAAMGSRLTEHQVAEPPEDWPALIWQQQRELAELRHSQEELLQRLCTQLEGLQSTVTGHVERALETRHEQEQRRLERALAEGQQRGGQLQEQLTQQLSQALSSAVAGRLERSIRDEIKKTVPPCVSRSLEPMAGQLSNSVATKLTAVEGSMKENISKLLKSKNLTDAIARAAADTLQGPMQAAYREAFQSVVLPAFEKSCQAMFQQINDSFRLGTQEYLQQLESHMKSRKAREQEAREPVLAQLRGLVSTLQSATEQMAATVAGSVRAEVQHQLHVAVGSLQESILAQVQRIVKGEVSVALKEQQAAVTSSIMQAMRSAAGTPVPSAHLDCQAQQAHILQLLQQGHLNQAFQQALTAADLNLVLYVCETVDPAQVFGQPPCPLSQPVLLSLIQQLASDLGTRTDLKLSYLEEAVMHLDHSDPITRDHMGSVMAQVRQKLFQFLQAEPHNSLGKAARRLSLMLHGLVTPSLP

14) >gi|21361794|ref|NP_060918.2| cullin-associated NEDD8-dissociated protein 1 [Homo sapiens] (136.3 kD)

MASASYHISNLLEKMTSSDKDFRFMATNDLMTELQKDSIKLDDDSERKVVKMILKLLEDKNGEVQNLAVKCLGPLVSKVKEYQVETIVDTLCTNMLSDKEQLRDISSIGLKTVIGELPPASSGSALAANVCKKITGRLTSAIAKQEDVSVQLEALDIMADMLSRQGGLLVNFHPSILTCLLPQLTSPRLAVRKRTIIALGHLVMSCGNIVFVDLIEHLLSELSKNDSMSTTRTYIQCIAAISRQAGHRIGEYLEKIIPLVVKFCNVDDDELREYCIQAFESFVRRCPKEVYPHVSTIINICLKYLTYDPNYNYDDEDEDENAMDADGGDDDDQGSDDEYSDDDDMSWKVRRAAAKCLDAVVSTRHEMLPEFYKTVSPALISRFKEREENVKADVFHAYLSLLKQTRPVQSWLCDPDAMEQGETPLTMLQSQVPNIVKALHKQMKEKSVKTRQCCFNMLTELVNVLPGALTQHIPVLVPGIIFSLNDKSSSSNLKIDALSCLYVILCNHSPQVFHPHVQALVPPVVACVGDPFYKITSEALLVTQQLVKVIRPLDQPSSFDATPYIKDLFTCTIKRLKAADIDQEVKERAISCMGQIICNLGDNLGSDLPNTLQIFLERLKNEITRLTTVKALTLIAGSPLKIDLRPVLGEGVPILASFLRKNQRALKLGTLSALDILIKNYSDSLTAAMIDAVLDELPPLISESDMHVSQMAISFLTTLAKVYPSSLSKISGSILNELIGLVRSPLLQGGALSAMLDFFQALVVTGTNNLGYMDLLRMLTGPVYSQSTALTHKQSYYSIAKCVAALTRACPKEGPAVVGQFIQDVKNSRSTDSIRLLALLSLGEVGHHIDLSGQLELKSVILEAFSSPSEEVKSAASYALGSISVGNLPEYLPFVLQEITSQPKRQYLLLHSLKEIISSASVVGLKPYVENIWALLLKHCECAEEGTRNVVAECLGKLTLIDPETLLPRLKGYLISGSSYARSSVVTAVKFTISDHPQPIDPLLKNCIGDFLKTLEDPDLNVRRVALVTFNSAAHNKPSLIRDLLDTVLPHLYNETKVRKELIREVEMGPFKHTVDDGLDIRKAAFECMYTLLDSCLDRLDIFEFLNHVEDGLKDHYDIKMLTFLMLVRLSTLCPSAVLQRLDRLVEPLRATCTTKVKANSVKQEFEKQDELKRSAMRAVAALLTIPEAEKSPLMSEFQSQISSNPELAAIFESIQKDSSSTNLESMDTS

15) >gi|22748937|ref|NP_065801.1| exportin-5 [Homo sapiens] (136.2 kD)

MAMDQVNALCEQLVKAVTVMMDPNSTQRYRLEALKFCEEFKEKCPICVPCGLRLAEKTQVAIVRHFGLQILEHVVKFRWNGMSRLEKVYLKNSVMELIANGTLNILEEENHIKDALSRIVVEMIKREWPQHWPDMLIELDTLSKQGETQTELVMFILLRLAEDVVTFQTLPPQRRRDIQQTLTQNMERIFSFLLNTLQENVNKYQQVKTDTSQESKAQANCRVGVAALNTLAGYIDWVSMSHITAENCKLLEILCLLLNEQELQLGAAECLLIAVSRKGKLEDRKPLMVLFGDVAMHYILSAAQTADGGGLVEKHYVFLKRLCQVLCALGNQLCALLGADSDVETPSNFGKYLESFLAFTTHPSQFLRSSTQMTWGALFRHEILSRDPLLLAIIPKYLRASMTNLVKMGFPSKTDSPSCEYSRFDFDSDEDFNAFFNSSRAQQGEVMRLACRLDPKTSFQMAGEWLKYQLSTFLDAGSVNSCSAVGTGEGSLCSVFSPSFVQWEAMTLFLESVITQMFRTLNREEIPVNDGIELLQMVLNFDTKDPLILSCVLTNVSALFPFVTYRPEFLPQVFSKLFSSVTFETVEESKAPRTRAVRNVRRHACSSIIKMCRDYPQLVLPNFDMLYNHVKQLLSNELLLTQMEKCALMEALVLISNQFKNYERQKVFLEELMAPVASIWLSQDMHRVLSDVDAFIAYVGTDQKSCDPGLEDPCGLNRARMSFCVYSILGVVKRTCWPTDLEEAKAGGFVVGYTSSGNPIFRNPCTEQILKLLDNLLALIRTHNTLYAPEMLAKMAEPFTKALDMLDAEKSAILGLPQPLLELNDSPVFKTVLERMQRFFSTLYENCFHILGKAGPSMQQDFYTVEDLATQLLSSAFVNLNNIPDYRLRPMLRVFVKPLVLFCPPEHYEALVSPILGPLFTYLHMRLSQKWQVINQRSLLCGEDEAADENPESQEMLEEQLVRMLTREVMDLITVCCVSKKGADHSSAPPADGDDEEMMATEVTPSAMAELTDLGKCLMKHEDVCTALLITAFNSLAWKDTLSCQRTTSQLCWPLLKQVLSGTLLADAVTWLFTSVLKGLQMHGQHDGCMASLVHLAFQIYEALRPRYLEIRAVMEQIPEIQKDSLDQFDCKLLNPSLQKVADKRRKDQFKRLIAGCIGKPLGEQFRKEVHIKNLPSLFKKTKPMLETEVLDNDGGGLATIFEP

16) >gi|21327715|ref|NP_006697.2| transcription elongation regulator 1 isoform 1 [Homo sapiens] (123.8 kD)

MAERGGDGGESERFNPGELRMAQQQALRFRGPAPPPNAVMRGPPPLMRPPPPFGMMRGPPPPPRPPFGRPPFDPNMPPMPPPGGIPPPMGPPHLQRPPFMPPPMSSMPPPPGMMFPPGMPPVTAPGTPALPPTEEIWVENKTPDGKVYYYNARTRESAWTKPDGVKVIQQSELTPMLAAQAQVQAQAQAQAQAQAQAQAQAQAQAQAQAQAQAQAQAQAQAQAQAQAQAQAQAQAQAQAQAQVQAQVQAQVQAQAVGASTPTTSSPAPAVSTSTSSSTPSSTTSTTTTATSVAQTVSTPTTQDQTPSSAVSVATPTVSVSTPAPTATPVQTVPQPHPQTLPPAVPHSVPQ

PTTAIPAFPPVMVPPFRVPLPGMPIPLPGVAMMQIVSCPYVKTVATTKTGVLPGMAPPIVPMIHPQVAIAASPATLAGATAVSEWTEYKTADGKTYYYNNRTLESTWEKPQELKEKEKLEEKIKEPIKEPSEEPLPMETEEEDPKEEPIKEIKEEPKEEEMTEEEKAAQKAKPVATAPIPGTPWCVVWTGDERVFFYNPTTRLSMWDRPDDLIGRADVDKIIQEPPHKKGMEELKKLRHPTPTMLSIQKWQFSMSAIKEEQELMEEINEDEPVKAKKRKRDDNKDIDSEKEAAMEAEIKAARERAIVPLEARMKQFKDMLLERGVSAFSTWEKELHKIVFDPRYLLLNPKERKQVFDQYVKTRAEEERREKKNKIMQAKEDFKKMMEEAKFNPRATFSEFAAKHAKDSRFKAIEKMKDREALFNEFVAAARKKEKEDSKTRGEKIKSDFFELLSNHHLDSQSRWSKVKDKVESDPRYKAVDSSSMREDLFKQYIEKIAKNLDSEKEKELERQARIEASLREREREVQKARSEQTKEIDREREQHKREEAIQNFKALLSDMVRSSDVSWSDTRRTLRKDHRWESGSLLEREEKEKLFNEHIEALTKKKREHFRQLLDETSAITLTSTWKEVKKIIKEDPRCIKFSSSDRKKQREFEEYIRDKYITAKADFRTLLKETKFITYRSKKLIQESDQHLKDVEKILQNDKRYLVLDCVPEERRKLIVAYVDDLDRRGPPPPPTASEPTRRSTK

17) >gi|14149680|ref|NP_056107.1| extended synaptotagmin-1 isoform 2 [Homo sapiens] (122.8 kD)

MERSPGEGPSPSPMDQPSAPSDPTDQPPAAHAKPDPGSGGQPAGPGAAGEALAVLTSFGRRLLVLIPVYLAGAVGLSVGFVLFGLALYLGWRRVRDEKERSLRAARQLLDDEEQLTAKTLYMSHRELPAWVSFPDVEKAEWLNKIVAQVWPFLGQYMEKLLAETVAPAVRGSNPHLQTFTFTRVELGEKPLRIIGVKVHPGQRKEQILLDLNISYVGDVQIDVEVKKYFCKAGVKGMQLHGVLRVILEPLIGDLPFVGAVSMFFIRRPTLDINWTGMTNLLDIPGLSSLSDTMIMDSIAAFLVLPNRLLVPLVPDLQDVAQLRSPLPRGIIRIHLLAARGLSSKDKYVKGLIEGKSDPYALVRLGTQTFCSRVIDEELNPQWGETYEVMVHEVPGQEIEVEVFDKDPDKDDFLGRMKLDVGKVLQASVLDDWFPLQGGQGQVHLRLEWLSLLSDAEKLEQVLQWNWGVSSRPDPPSAAILVVYLDRAQDLPLKKGNKEPNPMVQLSIQDVTQESKAVYSTNCPVWEEAFRFFLQDPQSQELDVQVKDDSRALTLGALTLPLARLLTAPELILDQWFQLSSSGPNSRLYMKLVMRILYLDSSEICFPTVPGCPGAWDVDSENPQRGSSVDAPPRPCHTTPDSQFGTEHVLRIHVLEAQDLIAKDRFLGGLVKGKSDPYVKLKLAGRSFRSHVVREDLNPRWNEVFEVIVTSVPGQELEVEVFDKDLDKDDFLGRCKVRLTTVLNSGFLDEWLTLEDVPSGRLHLRLERLTPRPTAAELEEVLQVNSLIQTQKSAELAAALLSIYMERAEDLPLRKGTKHLSPYATLTVGDSSHKTKTISQTSAPVWDESASFLIRKPHTESLELQVRGEGTGVLGSLSLPLSELLVADQLCLDRWFTLSSGQGQVLLRAQLGILVSQHSGVEAHSHSYSHSSSSLSEEPELSGGPPHITSSAPELRQRLTHVDSPLEAPAGPLGQVKLTLWYYSEERKLVSIVHGCRSLRQNGRDPPDPYVSLLLLPDKNRGTKRRTSQKKRTLSPEFNERFEWELPLDEAQRRKLDVSVKSNSSFMSRERELLGKVQLDLAETDLSQGVARWYDLMDNKDKGSS

18) >gi|74027249|ref|NP_056990.3| E3 ubiquitin-protein ligase TRIM33 isoform alpha [Homo sapiens] (122.4 kD)

MAENKGGGEAESGGGGSGSAPVTAGAAGPAAQEAEPPLTAVLVEEEEEEGGRAGAEGGAAGPDDGGVAAASSGSAQAASSPAASVGTGVAGGAVSTPAPAPASAPAPGPSAGPPPGPPASLLDTCAVCQQSLQSRREAEPKLLPCLHSFCLRCLPEPERQLSVPIPGGSNGDIQQVGVIRCPVCRQECRQIDLVDNYFVKDTSEAPSSSDEKSEQVCTSCEDNASAVGFCVECGEWLCKTCIEAHQRVKFTKDHLIRKKEDVSESVGASGQRPVFCPVHKQEQLKLFCETCDRLTCRDCQLLEHKEHRYQFLEEAFQNQKGAIENLLAKLLEKKNYVHFAATQVQNRIKEVNETNKRVEQEIKVAIFTLINEINKKGKSLLQQLENVTKERQMKLLQQQNDITGLSRQVKHVMNFTNWAIASGSSTALLYSKRLITFQLRHILKARCDPVPAANGAIRFHCDPTFWAKNVVNLGNLVIESKPAPGYTPNVVVGQVPPGTNHISKTPGQINLAQLRLQHMQQQVYAQKHQQLQQMRMQQPPAPVPTTTTTTQQHPRQAAPQMLQQQPPRLISVQTMQRGNMNCGAFQAHQMRLAQNAARIPGIPRHSGPQYSMMQPHLQRQHSNPGHAGPFPVVSVHNTTINPTSPTTATMANANRGPTSPSVTAIELIPSVTNPENLPSLPDIPPIQLEDAGSSSLDNLLSRYISGSHLPPQPTSTMNPSPGPSALSPGSSGLSNSHTPVRPPSTSSTGSRGSCGSSGRTAEKTSLSFKSDQVKVKQEPGTEDEICSFSGGVKQEKTEDGRRSACMLSSPESSLTPPLSTNLHLESELDALASLENHVKIEPADMNESCKQSGLSSLVNGKSPIRSLMHRSARIGGDGNNKDDDPNEDWCAVCQNGGDLLCCEKCPKVFHLTCHVPTLLSFPSGDWICTFCRDIGKPEVEYDCDNLQHSKKGKTAQGLSPVDQRKCERLLLYLYCHELSIEFQEPVPASIPNYYKIIKKPMDLSTVKKKLQKKHSQHYQIPDDFVADVRLIFKNCERFNEMMKVVQVYADTQEINLKADSEVAQAGKAVALYFEDKLTEIYSDRTFAPLPEFEQEEDDGEVTEDSDEDFIQPRRKRLKSDERPVHIK

19) >gi|32484979|ref|NP_003655.3| AP-3 complex subunit beta-1 isoform 1 [Homo sapiens] (121.2 kD)

MSSNSFPYNEQSGGGEATELGQEATSTISPSGAFGLFSSDLKKNEDLKQMLESNKDSAKLDAMKRIVGMIAKGKNASELFPAVVKNVASKNIEIKKLVYVYLVRYAEEQQDLALLSISTFQRALKDPNQLIRASALRVLSSIRVPIIVPIMMLAIKEASADLSPYVRKNAAHAIQKLYSLDPEQKEMLIEVIEKLLKDKSTLVAGSVVMAFEEVCPDRIDLIHKNYRKLCNLLVDVEEWGQVVIIHMLTRYARTQFVSPWKEGDELEDNGKNFYESDDDQKEKTDKKKKPYTMDPDHRLLIRNTKPLLQSRNAAVVMAVAQLYWHISPKSEAGIISKSLVRLLRSNREVQYIVLQNIATMSIQRKGMFEPYLKSFYVRSTDPTMIKTLKLEILTNLANEANISTLLREFQTYVKSQDKQFAAATIQTIGRCATNILEVTDTCLNGLVCLLSNRDEIVVAESVVVIKKLLQMQPAQHGEIIKHMAKLLDSITVPVARASILWLIGENCERVPKIAPDVLRKMAKSFTSEDDLVKLQILNLGAKLYLTNSKQTKLLTQYILNLGKYDQNYDIRDRTRFIRQLIVPNVKSGALSKYAKKIFLAQKPAPLLESPFKDRDHFQLGTLSHTLNIKATGYLELSNWPEVAPDPSVRNVEVIELAKEWTPAGKAKQENSAKKFYSESEEEEDSSDSSSDSESESGSESGEQGESGEEGDSNEDSSEDSSSEQDSESGRESGLENKRTAKRNSKAKGKSDSEDGEKENEKSKTSDSSNDESSSIEDSSSDSESESEPESESESRRVTKEKEKKTKQDRTPLTKDVSLLDLDDFNPVSTPVALPTPALSPSLMADLEGLHLSTSSSVISVSTPAFVPTKTHVLLHRMSGKGLAAHYFFPRQPCIFGDKMVSIQITLNNTTDRKIENIHIGEKKLPIGMKMHVFNPIDSLEPEGSITVSMGIDFCDSTQTASFQLCTKDDCFNVNIQPPVGELLLPVAMSEKDFKKEQGVLTGMNETSAVIIAAPQNFTPSVIFQKVVNVANVGAVPSGQDNIHRFAAKTVHSGSLMLVTVELKEGSTAQLIINTEKTVIGSVLLRELKPVLSQG

20) >gi|149158692|ref|NP_004630.3| large proline-rich protein BAG6 isoform a [Homo sapiens] (119.3 kD)

MEPNDSTSTAVEEPDSLEVLVKTLDSQTRTFIVGAQMNVKEFKEHIAASVSIPSEKQRLIYQGRVLQDDKKLQEYNVGGKVIHLVERAPPQTHLPSGASSGTGSASATHGGGSPPGTRGPGASVHDRNANSYVMVGTFNLPSDGSAVDVHINMEQAPIQSEPRVRLVMAQHMIRDIQTLLSRMETLPYLQCRGGPQPQHSQPPPQPPAVTPEPVALSSQTSEPVESEAPPREPMEAEEVEERAPAQNPELTPGPAPAGPTPAPETNAPNHPSPAEYVEVLQELQRLESRLQPFLQRYYEVLGAAATTDYNNNHEGREEDQRLINLVGESLRLLGNTFVALSDLRCNLACTPPRHLHVVRPMSHYTTPMVLQQAAIPIQINVGTTVTMTGNGTRPPPTPNAEAPPPGPGQASSVAPSSTNVESSAEGAPPPGPAPPPATSHPRVIRISHQSVEPVVMMHMNIQDSGTQPGGVPSAPTGPLGPPGHGQTLGQQVPGFPTAPTRVVIARPTPPQARPSHPGGPPVSGTLQGAGLGTNASLAQMVSGLVGQLLMQPVLVAQGTPGMAPPPAPATASASAGTTNTATTAGPAPGGPAQPPPTPQPSMADLQFSQLLGNLLGPAGPGAGGSGVASPTITVAMPGVPAFLQGMTDFLQATQTAPPPPPPPPPPPPAPEQQTMPPPGSPSGGAGSPGGLGLESLSPEFFTSVVQGVLSSLLGSLGARAGSSESIAAFIQRLSGSSNIFEPGADGALGFFGALLSLLCQNFSMVDVVMLLHGHFQPLQRLQPQLRSFFHQHYLGGQEPTPSNIRMATHTLITGLEEYVRESFSLVQVQPGVDIIRTNLEFLQEQFNSIAAHVLHCTDSGFGARLLELCNQGLFECLALNLHCLGGQQMELAAVINGRIRRMSRGVNPSLVSWLTTMMGLRLQVVLEHMPVGPDAILRYVRRVGDPPQPLPEEPMEVQGAERASPEPQRENASPAPGTTAEEAMSRGPPPAPEGGSRDEQDGASAETEPWAAAVPPEWVPIIQQDIQSQRKVKPQPPLSDAYLSGMPAKRRKTMQGEGPQLLLSEAVSRAAKAAGARPLTSPESLSRDLEAPEVQESYRQQLRSDIQKRLQEDPNYSPQRFPNAQRAFADDP

21) >gi|62460637|ref|NP_078934.3| importin-4 [Homo sapiens] (118.6 kD)

MESAGLEQLLRELLLPDTERIRRATEQLQIVLRAPAALPALCDLLASAADPQIRQFAAVLTRRRLNTRWRRLAAEQRESLKSLILTALQRETEHCVSLSLAQLSATIFRKEGLEAWPQLLQLLQHSTHSPHSPEREMGLLLLSVVVTSRPEAFQPHHRELLRLLNETLGEVGSPGLLFYSLRTLTTMAPYLSTEDVPLARMLVPKLIMAMQTLIPIDEAKACEALEALDELLESEVPVITPYLSEVLTFCLEVARNVALGNAIRIRILCCLTFLVKVKSKALLKNRLLPPLLHTLFPIVAAEPPPGQLDPEDQDSEEEELEIELMGETPKHFAVQVVDMLALHLPPEKLCPQLMPMLEEALRSESPYQRKAGLLVLAVLSDGAGDHIRQRLLPPLLQIVCKGLEDPSQVVRNAALFALGQFSENLQPHISSYSREVMPLLLAYLKSVPLGHTHHLAKACYALENFVENLGPKVQPYLPELMECMLQLLRNPSSPRAKELAVSALGAIATAAQASLLPYFPAIMEHLREFLLTGREDLQPVQIQSLETLGVLARAVGEPMRPLAEECCQLGLGLCDQVDDPDLRRCTYSLFAALSGLMGEGLAPHLEQITTLMLLSLRSTEGIVPQYDGSSSFLLFDDESDGEEEEELMDEDVEEEDDSEISGYSVENAFFDEKEDTCAAVGEISVNTSVAFLPYMESVFEEVFKLLECPHLNVRKAAHEALGQFCCALHKACQSCPSEPNTAALQAALARVVPSYMQAVNRERERQVVMAVLEALTGVLRSCGTLTLKPPGRLAELCGVLKAVLQRKTACQDTDEEEEEEDDDQAEYDAMLLEHAGEAIPALAAAAGGDSFAPFFAGFLPLLVCKTKQGCTVAEKSFAVGTLAETIQGLGAASAQFVSRLLPVLLSTAQEADPEVRSNAIFGMGVLAEHGGHPAQEHFPKLLGLLFPLLARERHDRVRDNICGALARLLMASPTRKPEPQVLAALLHALPLKEDLEEWVTIGRLFSFLYQSSPDQVIDVAPELLRICSLILADNKIPPDTKAALLLLLTFLAKQHTDSFQAALGSLPVDKAQELQAVLGLS

22) >gi|41327773|ref|NP_055644.2| probable ATP-dependent RNA helicase DDX46 [Homo sapiens] (117.3 kD)

MGRESRHYRKRSASRGRSGSRSRSRSPSDKRSKRGDDRRSRSRDRDRRRERSRSRDKRRSRSRDRKRLRRSRSRERDRSRERRRSRSRDRRRSRSRSRGRRSRSSSPGNKSKKTENRSRSKEKTDGGESSKEKKKDKDDKEDEKEKDAGNFDQNKLEEEMRKRKERVEKWREEQRKKAMENIGELKKEIEEMKQGKKWSLEDDDDDEDDPAEAEKEGNEMEGEELDPLDAYMEEVKEEVKKFNMRSVKGGGGNEKKSGPTVTKVVTVVTTKKAVVDSDKKKGELMENDQDAMEYSSEEEEVDLQTALTGYQTKQRKLLEPVDHGKIEYEPFRKNFYVEVPELAKMSQEEVNVFRLEMEGITVKGKGCPKPIKSWVQCGISMKILNSLKKHGYEKPTPIQTQAIPAIMSGRDLIGIAKTGSGKTIAFLLPMFRHIMDQRSLEEGEGPIAVIMTPTRELALQITKECKKFSKTLGLRVVCVYGGTGISEQIAELKRGAEIIVCTPGRMIDMLAANSGRVTNLRRVTYVVLDEADRMFDMGFEPQVMRIVDNVRPDRQTVMFSATFPRAMEALARRILSKPIEVQVGGRSVVCSDVEQQVIVIEEEKKFLKLLELLGHYQESGSVIIFVDKQEHADGLLKDLMRASYPCMSLHGGIDQYDRDSIINDFKNGTCKLLVATSVAARGLDVKHLILVVNYSCPNHYEDYVHRAGRTGRAGNKGYAYTFITEDQARYAGDIIKALELSGTAVPPDLEKLWSDFKDQQKAEGKIIKKSSGFSGKGFKFDETEQALANERKKLQKAALGLQDSDDEDAAVDIDEQIESMFNSKKRVKDMAAPGTSSVPAPTAGNAEKLEIAKRLALRINAQKNLGIESQDVMQQATNAILRGGTILAPTVSAKTIAEQLAEKINAKLNYVPLEKQEEERQDGGQNESFKRYEEELEINDFPQTARWKVTSKEALQRISEYSEAAITIRGTYFPPGKEPKEGERKIYLAIESANELAVQKAKAEITRLIKEELIRLQNSYQPTNKGRYKVL

23) >gi|34101286|ref|NP_057191.2| zinc finger RNA-binding protein [Homo sapiens] (116.9 kD)

MIPICPVVSFTYVPSRLGEDAKMATGNYFGFTHSGAAAAAAAAQYSQQPASGVAYSHPTTVASYTVHQAPVAAHTVTAAYAPAAATVAVARPAPVAVAAAATAAAYGGYPTAHTATDYGYTQRQQEAPPPPPPATTQNYQDSYSYVRSTAPAVAYDSKQYYQQPTATAAAVAAAAQPQPSVAETYYQTAPKAGYSQGATQYTQAQQTRQVTAIKPATPSPATTTFSIYPVSSTVQPVAAAATVVPSYTQSATYSTTAVTYSGTSYSGYEAAVYSAASSYYQQQQQQQKQAAAAAAAAAATAAWTGTTFTKKAPFQNKQLKPKQPPKPPQIHYCDVCKISCAGPQTYKEHLEGQKHKKKEAALKASQNTSSSNSSTRGTQNQLRCELCDVSCTGADAYAAHIRGAKHQKVVKLHTKLGKPIPSTEPNVVSQATSSTAVSASKPTASPSSIAANNCTVNTSSVATSSMKGLTTTGNSSLNSTSNTKVSAVPTNMAAKKTSTPKINFVGGNKLQSTGNKAEDIKGTECVKSTPVTSAVQIPEVKQDTVSEPVTPASLAALQSDVQPVGHDYVEEVRNDEGKVIRFHCKLCECSFNDPNAKEMHLKGRRHRLQYKKKVNPDLQVEVKPSIRARKIQEEKMRKQMQKEEYWRRREEEERWRMEMRRYEEDMYWRRMEEEQHHWDDRRRMPDGGYPHGPPGPLGLLGVRPGMPPQPQGPAPLRRPDSSDDRYVMTKHATIYPTEEELQAVQKIVSITERALKLVSDSLSEHEKNKNKEGDDKKEGGKDRALKGVLRVGVLAKGLLLRGDRNVNLVLLCSEKPSKTLLSRIAENLPKQLAVISPEKYDIKCAVSEAAIILNSCVEPKMQVTITLTSPIIREENMREGDVTSGMVKDPPDVLDRQKCLDALAALRHAKWFQARANGLQSCVIIIRILRDLCQRVPTWSDFPSWAMELLVEKAISSASSPQSPGDALRRVFECISSGIILKGSPGLLDPCEKDPFDTLATMTDQQREDITSSAQFALRLLAFRQIHKVLGMDPLPQMSQRFNIHNNRKRRRDSDGVDGFEAEGKKDKKDYDNF

24) >gi|21071054|ref|NP_620636.1| helicase-like transcription factor [Homo sapiens] (113.9 kD)

MSWMFKRDPVWKYLQTVQYGVHGNFPRLSYPTFFPRFEFQDVIPPDDFLTSDEEVDSVLFGSLRGHVVGLRYYTGVVNNNEMVALQRDPNNPYDKNAIKVNNVNGNQVGHLKKELAGALAYIMDNKLAQIEGVVPFGANNAFTMPLHMTFWGKEENRKAVSDQLKKHGFKLGPAPKTLGFNLESGWGSGRAGPSYSMPVHAAVQMTTEQLKTEFDKLFEDLKEDDKTHEMEPAEAIETPLLPHQKQALAWMVSRENSKELPPFWEQRNDLYYNTITNFSEKDRPENVHGGILADDMGLGKTLTAIAVILTNFHDGRPLPIERVKKNLLKKEYNVNDDSMKLGGNNTSEKADGLSKDASRCSEQPSISDIKEKSKFRMSELSSSRPKRRKTAVQYIESSDSEEIETSELPQKMKGKLKNVQSETKGRAKAGSSKVIEDVAFACALTSSVPTTKKKMLKKGACAVEGSKKTDVEERPRTTLIICPLSVLSNWIDQFGQHIKSDVHLNFYVYYGPDRIREPALLSKQDIVLTTYNILTHDYGTKGDSPLHSIRWLRVILDEGHAIRNPNAQQTKAVLDLESERRWVLTGTPIQNSLKDLWSLLSFLKLKPFIDREWWHRTIQRPVTMGDEGGLRRLQSLIKNITLRRTKTSKIKGKPVLELPERKVFIQHITLSDEERKIYQSVKNEGRATIGRYFNEGTVLAHYADVLGLLLRLRQICCHTYLLTNAVSSNGPSGNDTPEELRKKLIRKMKLILSSGSDEECAICLDSLTVPVITHCAHVFCKPCICQVIQNEQPHAKCPLCRNDIHEDNLLECPPEELARDSEKKSDMEWTSSSKINALMHALTDLRKKNPNIKSLVVSQFTTFLSLIEIPLKASGFVFTRLDGSMAQKKRVESIQCFQNTEAGSPTIMLLSLKAGGVGLNLSAASRVFLMDPAWNPAAEDQCFDRCHRLGQKQEVIITKFIVKDSVEENMLKIQNKKRELAAGAFGTKKPNADEMKQAKINEIRTLIDL

25) >gi|195976805|ref|NP_001124463.1| hypoxia up-regulated protein 1 precursor [Homo sapiens] (111.3 kD)

MADKVRRQRPRRRVCWALVAVLLADLLALSDTLAVMSVDLGSESMKVAIVKPGVPMEIVLNKESRRKTPVIVTLKENERFFGDSAASMAIKNPKATLRYFQHLLGKQADNPHVALYQARFPEHELTFDPQRQTVHFQISSQLQFSPEEVLGMVLNYSRSLAEDFAEQPIKDAVITVPVFFNQAERRAVLQAARMAGLKVLQLINDNTATALSYGVFRRKDINTTAQNIMFYDMGSGSTVCTIVTYQMVKTKEAGMQPQLQIRGVGFDRTLGGLEMELRLRERLAGLFNEQRKGQRAKDVRENPRAMAKLLREANRLKTVLSANADHMAQIEGLMDDVDFKAKVTRVEFEELCADLFERVPGPVQQALQSAEMSLDEIEQVILVGGATRVPRVQEVLLKAVGKEELGKNINADEAAAMGAVYQAAALSKAFKVKPFVVRDAVVYPILVEFTREVEEEPGIHSLKHNKRVLFSRMGPYPQRKVITFNRYSHDFNFHINYGDLGFLGPEDLRVFGSQNLTTVKLKGVGDSFKKYPDYESKGIKAHFNLDESGVLSLDRVESVFETLVEDSAEEESTLTKLGNTISSLFGGGTTPDAKENGTDTVQEEEESPAEGSKDEPGEQVELKEEAEAPVEDGSQPPPPEPKGDATPEGEKATEKENGDKSEAQKPSEKAEAGPEGVAPAPEGEKKQKPARKRRMVEEIGVELVVLDLPDLPEDKLAQSVQKLQDLTLRDLEKQEREKAANSLEAFIFETQDKLYQPEYQEVSTEEQREEISGKLSAASTWLEDEGVGATTVMLKEKLAELRKLCQGLFFRVEERKKWPERLSALDNLLNHSSMFLKGARLIPEMDQIFTEVEMTTLEKVINETWAWKNATLAEQAKLPATEKPVLLSKDIEAKMMALDREVQYLLNKAKFTKPRPRPKDKNGTRAEPPLNASASDQGEKVIPPAGQTEDAEPISEPEKVETGSEPGDTEPLELGGPGAEPEQKEQSTGQKRPLKNDEL

26) >gi|29029559|ref|NP_001307.2| exportin-2 isoform 1 [Homo sapiens] (110.3 kD)

MELSDANLQTLTEYLKKTLDPDPAIRRPAEKFLESVEGNQNYPLLLLTLLEKSQDNVIKVCASVTFKNYIKRNWRIVEDEPNKICEADRVAIKANIVHLMLSSPEQIQKQLSDAISIIGREDFPQKWPDLLTEMVNRFQSGDFHVINGVLRTAHSLFKRYRHEFKSNELWTEIKLVLDAFALPLTNLFKATIELCSTHANDASALRILFSSLILISKLFYSLNFQDLPEFFEDNMETWMNNFHTLLTLDNKLLQTDDEEEAGLLELLKSQICDNAALYAQKYDEEFQRYLPRFVTAIWNLLVTTGQEVKYDLLVSNAIQFLASVCERPHYKNLFEDQNTLTSICEKVIVPNMEFRAADEEAFEDNSEEYIRRDLEGSDIDTRRRAACDLVRGLCKFFEGPVTGIFSGYVNSMLQEYAKNPSVNWKHKDAAIYLVTSLASKAQTQKHGITQANELVNLTEFFVNHILPDLKSANVNEFPVLKADGIKYIMIFRNQVPKEHLLVSIPLLINHLQAESIVVHTYAAHALERLFTMRGPNNATLFTAAEIAPFVEILLTNLFKALTLPGSSENEYIMKAIMRSFSLLQEAIIPYIPTLITQLTQKLLAVSKNPSKPHFNHYMFEAICLSIRITCKANPAAVVNFEEALFLVFTEILQNDVQEFIPYVFQVMSLLLETHKNDIPSSYMALFPHLLQPVLWERTGNIPALVRLLQAFLERGSNTIASAAADKIPGLLGVFQKLIASKANDHQGFYLLNSIIEHMPPESVDQYRKQIFILLFQRLQNSKTTKFIKSFLVFINLYCIKYGALALQEIFDGIQPKMFGMVLEKIIIPEIQKVSGNVEKKICAVGITKLLTECPPMMDTEYTKLWTPLLQSLIGLFELPEDDTIPDEEHFIDIEDTPGYQTAFSQLAFAGKKEHDPVGQMVNNPKIHLAQSLHKLSTACPGRVPSMVSTSLNAEALQYLQGYLQAASVTLL

27) >gi|146231940|ref|NP_001078927.1| catenin delta-1 isoform 1ABC [Homo sapiens] (108.1 kD)

MDDSEVESTASILASVKEQEAQFEKLTRALEEERRHVSAQLERVRVSPQDANPLMANGTLTRRHQNGRFVGDADLERQKFSDLKLNGPQDHSHLLYSTIPRMQEPGQIVETYTEEDPEGAMSVVSVETSDDGTTRRTETTVKKVVKTVTTRTVQPVAMGPDGLPVDASSVSNNYIQTLGRDFRKNGNGGPGPYVGQAGTATLPRNFHYPPDGYSRHYEDGYPGGSDNYGSLSRVTRIEERYRPSMEGYRAPSRQDVYGPQPQVRVGGSSVDLHRFHPEPYGLEDDQRSMGYDDLDYGMMSDYGTARRTGTPSDPRRRLRSYEDMIGEEVPSDQYYWAPLAQHERGSLASLDSLRKGGPPPPNWRQPELPEVIAMLGFRLDAVKSNAAAYLQHLCYRNDKVKTDVRKLKGIPVLVGLLDHPKKEVHLGACGALKNISFGRDQDNKIAIKNCDGVPALVRLLRKARDMDLTEVITGTLWNLSSHDSIKMEIVDHALHALTDEVIIPHSGWEREPNEDCKPRHIEWESVLTNTAGCLRNVSSERSEARRKLRECDGLVDALIFIVQAEIGQKDSDSKLVENCVCLLRNLSYQVHREIPQAERYQEAAPNVANNTGPHAASCFGAKKGKDEWFSRGKKPIEDPANDTVDFPKRTSPARGYELLFQPEVVRIYISLLKESKTPAILEASAGAIQNLCAGRWTYGRYIRSALRQEKALSAIADLLTNEHERVVKAASGALRNLAVDARNKELIGKHAIPNLVKNLPGGQQNSSWNFSEDTVISILNTINEVIAENLEAAKKLRETQGIEKLVLINKSGNRSEKEVRAAALVLQTIWGYKELRKPLEKEGWKKSDFQVNLNNASRSQSSHSYDDSTLPLIDRNQKSDKKPDREEIQMSNMGSNTKSLDNNYSTPNERGDHNRTLDRSGDLGDMEPLKGTTPLMQDEGQESLEEELDVLVLDDEGGQVSYPSMQKI

28) >gi|530418825|ref|XP_005260998.1| PREDICTED: trifunctional purine biosynthetic protein adenosine-3 isoform X1 [Homo sapiens] (107.7 kD)

MAARVLIIGSGGREHTLAWKLAQSHHVKQVLVAPGNAGTACSEKISNTAISISDHTALAQFCKEKKIEFVVVGPEAPLAAGIVGNLRSAGVQCFGPTAEAAQLESSKRFAKEFMDRHGIPTAQWKAFTKPEEACSFILSADFPALVVKASGLAAGKGVIVAKSKEEACKAVQEIMQEKAFGAAGETIVIEELLDGEEVSCLCFTDGKTVAPMPPAQDHKRLLEGDGGPNTGGMGAYCPAPQVSNDLLLKIKDTVLQRTVDGMQQEGTPYTGILYAGIMLTKNGPKVLEFNCRFGDPECQVILPLLKSDLYEVIQSTLDGLLCTSLPVWLENHTALTVVMASKGYPGDYTKGVEITGFPEAQALGLEVFHAGTALKNGKVVTHGGRVLAVTAIRENLISALEEAKKGLAAIKFEGAIYRKDVGFRAIAFLQQPRSLTYKESGVDIAAGNMLVKKIQPLAKATSRSGCKVDLGGFAGLFDLKAAGFKDPLLASGTDGVGTKLKIAQLCNKHDTIGQDLVAMCVNDILAQGAEPLFFLDYFSCGKLDLSVTEAVVAGIAKACGKAGCALLGGETAEMPDMYPPGEYDLAGFAVGAMERDQKLPHLERITEGDVVVGIASSGLHSNGFSLVRKIVAKSSLQYSSPAPDGCGDQTLGDLLLTPTRIYSHSLLPVLRSGHVKAFAHITGGGLLENIPRVLPEKLGVDLDAQTWRIPRVFSWLQQEGHLSEEEMARTFNCGVGAVLVVSKEQTEQILRDIQQHKEEAWVIGSVVARAEGSPRVKVKNLIESMQINGSVLKNGSLTNHFSFEKKKARVAVLISGTGSNLQALIDSTREPNSSAQIDIVISNKAAVAGLDKAERAGIPTRVINHKLYKNRVEFDSAIDLVLEEFSIDIVCLAGFMRILSGPFVQKWNGKMLNIHPSLLPSFKGSNAHEQALETGVTVTGCTVHFVAEDVDAGQIILQEAVPVKRGDTVATLSERVKLAEHKIFPAALQLVASGTVQLGENGKICWVKEE

29) >gi|221316630|ref|NP_001137533.1| coatomer subunit beta [Homo sapiens] (107.1 kD)

MTAAENVCYTLINVPMDSEPPSEISLKNDLEKGDVKSKTEALKKVIIMILNGEKLPGLLMTIIRFVLPLQDHTIKKLLLVFWEIVPKTTPDGRLLHEMILVCDAYRKDLQHPNEFIRGSTLRFLCKLKEAELLEPLMPAIRACLEHRHSYVRRNAVLAIYTIYRNFEHLIPDAPELIHDFLVNEKDASCKRNAFMMLIHADQDRALDYLSTCIDQVQTFGDILQLVIVELIYKVCHANPSERARFIRCIYNLLQSSSPAVKYEAAGTLVTLSSAPTAIKAAAQCYIDLIIKESDNNVKLIVLDRLIELKEHPAHERVLQDLVMDILRVLSTPDLEVRKKTLQLALDLVSSRNVEELVIVLKKEVIKTNNVSEHEDTDKYRQLLVRTLHSCSVRFPDMAANVIPVLMEFLSDNNEAAAADVLEFVREAIQRFDNLRMLIVEKMLEVFHAIKSVKIYRGALWILGEYCSTKEDIQSVMTEIRRSLGEIPIVESEIKKEAGELKPEEEITVGPVQKLVTEMGTYATQSALSSSRPTKKEEDRPPLRGFLLDGDFFVAASLATTLTKIALRYVALVQEKKKQNSFVAEAMLLMATILHLGKSSLPKKPITDDDVDRISLCLKVLSECSPLMNDIFNKECRQSLSHMLSAKLEEEKLSQKKESEKRNVTVQPDDPISFMQLTAKNEMNCKEDQFQLSLLAAMGNTQRKEAADPLASKLNKVTQLTGFSDPVYAEAYVHVNQYDIVLDVLVVNQTSDTLQNCTLELATLGDLKLVEKPSPLTLAPHDFANIKANVKVASTENGIIFGNIVYDVSGAASDRNCVVLSDIHIDIMDYIQPATCTDAEFRQMWAEFEWENKVTVNTNMVDLNDYLQHILKSTNMKCLTPEKALSGYCGFMAANLYARSIFGEDALANVSIEKPIHQGPDAAVTGHIRIRAKSQGMALSLGDKINLSQKKTSI

30) >gi|21396489|ref|NP_004784.2| lon protease homolog, mitochondrial isoform 1 precursor [Homo sapiens] (106.4 kD)

MAASTGYVRLWGAARCWVLRRPMLAAAGGRVPTAAGAWLLRGQRTCDASPPWALWGRGPAIGGQWRGFWEASSRGGGAFSGGEDASEGGAEEGAGGAGGSAGAGEGPVITALTPMTIPDVFPHLPLIAITRNPVFPRFIKIIEVKNKKLVELLRRKVRLAQPYVGVFLKRDDSNESDVVESLDEIYHTGTFAQIHEMQDLGDKLRMIVMGHRRVHISRQLEVEPEEPEAENKHKPRRKSKRGKKEAEDELSARHPAELAMEPTPELPAEVLMVEVENVVHEDFQVTEEVKALTAEIVKTIRDIIALNPLYRESVLQMMQAGQRVVDNPIYLSDMGAALTGAESHELQDVLEETNIPKRLYKALSLLKKEFELSKLQQRLGREVEEKIKQTHRKYLLQEQLKIIKKELGLEKDDKDAIEEKFRERLKELVVPKHVMDVVDEELSKLGLLDNHSSEFNVTRNYLDWLTSIPWGKYSNENLDLARAQAVLEEDHYGMEDVKKRILEFIAVSQLRGSTQGKILCFYGPPGVGKTSIARSIARALNREYFRFSVGGMTDVAEIKGHRRTYVGAMPGKIIQCLKKTKTENPLILIDEVDKIGRGYQGDPSSALLELLDPEQNANFLDHYLDVPVDLSKVLFICTANVTDTIPEPLRDRMEMINVSGYVAQEKLAIAERYLVPQARALCGLDESKAKLSSDVLTLLIKQYCRESGVRNLQKQVEKVLRKSAYKIVSGEAESVEVTPENLQDFVGKPVFTVERMYDVTPPGVVMGLAWTAMGGSTLFVETSLRRPQDKDAKGDKDGSLEVTGQLGEVMKESARIAYTFARAFLMQHAPANDYLVTSHIHLHVPEGATPKDGPSAGCTIVTALLSLAMGRPVRQNLAMTGEVSLTGKILPVGGIKEKTIAAKRAGVTCIVLPAENKKDFYDLAAFITEGLEVHFVEHYREIFDIAFPDEQAEALAVER

31) >gi|22035565|ref|NP_057715.2| PAX3- and PAX7-binding protein 1 isoform 1 [Homo sapiens] (104.7 kD)

MFRKARRVNVRKRNDSEEEERERDEEQEPPPLLPPPGTGEEAGPGGGDRAPGGESLLGPGPSPPSALTPGLGAEAGGGFPGGAEPGNGLKPRKRPRENKEVPRASLLSFQDEEEENEEVFKVKKSSYSKKIVKLLKKEYKEDLEKSKIKTELNSSAESEQPLDKTGHVKDTNQEDGVIISEHGEDEMDMESEKEEEKPKTGGAFSNALSSLNVLRPGEIPDAAFIHAARKKRQMARELGDFTPHDNEPGKGRLVREDENDASDDEDDDEKRRIVFSVKEKSQRQKIAEEIGIEGSDDDALVTGEQDEELSRWEQEQIRKGINIPQVQASQPAEVNMYYQNTYQTMPYGSSYGIPYSYTAYGSSDAKSQKTDNTVPFKTPSNEMTPVTIDLVKKQLKDRLDSMKELHKTNRQQHEKHLQSRVDSTRAIERLEGSSGGIGERYKFLQEMRGYVQDLLECFSEKVPLINELESAIHQLYKQRASRLVQRRQDDIKDESSEFSSHSNKALMAPNLDSFGRDRALYQEHAKRRIAEREARRTRRRQAREQTGKMADHLEGLSSDDEETSTDITNFNLEKDRISKESGKVFEDVLESFYSIDCIKSQFEAWRSKYYTSYKDAYIGLCLPKLFNPLIRLQLLTWTPLEAKCRDFENMLWFESLLFYGCEEREQEKDDVDVALLPTIVEKVILPKLTVIAENMWDPFSTTQTSRMVGITLKLINGYPSVVNAENKNTQVYLKALLLRMRRTLDDDVFMPLYPKNVLENKNSGPYLFFQRQFWSSVKLLGNFLQWYGIFSNKTLQELSIDGLLNRYILMAFQNSEYGDDSIKKAQNVINCFPKQWFMNLKGERTISQLENFCRYLVHLADTIYRNSIGCSDVEKRNARENIKQIVKLLASVRALDHAMSVASDHNVKEFKSLIEGK

32) >gi|260436862|ref|NP_001118.3| AP-1 complex subunit beta-1 isoform a [Homo sapiens] (104.6 kD)

MTDSKYFTTTKKGEIFELKAELNSDKKEKKKEAVKKVIASMTVGKDVSALFPDVVNCMQTDNLELKKLVYLYLMNYAKSQPDMAIMAVNTFVKDCEDPNPLIRALAVRTMGCIRVDKITEYLCEPLRKCLKDEDPYVRKTAAVCVAKLHDINAQLVEDQGFLDTLKDLISDSNPMVVANAVAALSEIAESHPSSNLLDLNPQSINKLLTALNECTEWGQIFILDCLANYMPKDDREAQSICERVTPRLSHANSAVVLSAVKVLMKFMEMLSKDLDYYGTLLKKLAPPLVTLLSAEPELQYVALRNINLIVQKRPEILKHEMKVFFVKYNDPIYVKLEKLDIMIRLASQANIAQVLAELKEYATEVDVDFVRKAVRAIGRCAIKVEQSAERCVSTLLDLIQTKVNYVVQEAIVVIKDIFRKYPNKYESVIATLCENLDSLDEPEARAAMIWIVGEYAERIDNADELLESFLEGFHDESTQVQLQLLTAIVKLFLKKPTETQELVQQVLSLATQDSDNPDLRDRGYIYWRLLSTDPVAAKEVVLAEKPLISEETDLIEPTLLDELICYIGTLASVYHKPPSAFVEGGRGVVHKSLPPRTASSESAESPETAPTGAPPGEQPDVIPAQGDLLGDLLNLDLGPPVSGPPLATSSVQMGAVDLLGGGLDSLMGDEPEGIGGTNFVAPPTAAVPANLGAPIGSGLSDLFDLTSGVGTLSGSYVAPKAVWLPAMKAKGLEISGTFTRQVGSISMDLQLTNKALQVMTDFAIQFNRNSFGLAPAAPLQVHAPLSPNQTVEISLPLSTVGSVMKMEPLNNLQVAVKNNIDVFYFSTLYPLHILFVEDGKMDRQMFLATWKDIPNENEAQFQIRDCPLNAEAASSKLQSSNIFTVAKRNVEGQDMLYQSLKLTNGIWVLAELRIQPGNPSCTDLELSLKCRAPEVSQHVYQAYETILKN

33) >gi|4557469|ref|NP_001273.1| AP-2 complex subunit beta isoform b [Homo sapiens] (104.5 kD)

MTDSKYFTTNKKGEIFELKAELNNEKKEKRKEAVKKVIAAMTVGKDVSSLFPDVVNCMQTDNLELKKLVYLYLMNYAKSQPDMAIMAVNSFVKDCEDPNPLIRALAVRTMGCIRVDKITEYLCEPLRKCLKDEDPYVRKTAAVCVAKLHDINAQMVEDQGFLDSLRDLIADSNPMVVANAVAALSEISESHPNSNLLDLNPQNINKLLTALNECTEWGQIFILDCLSNYNPKDDREAQSICERVTPRLSHANSAVVLSAVKVLMKFLELLPKDSDYYNMLLKKLAPPLVTLLSGEPEVQYVALRNINLIVQKRPEILKQEIKVFFVKYNDPIYVKLEKLDIMIRLASQANIAQVLAELKEYATEVDVDFVRKAVRAIGRCAIKVEQSAERCVSTLLDLIQTKVNYVVQEAIVVIRDIFRKYPNKYESIIATLCENLDSLDEPDARAAMIWIVGEYAERIDNADELLESFLEGFHDESTQVQLTLLTAIVKLFLKKPSETQELVQQVLSLATQDSDNPDLRDRGYIYWRLLSTDPVTAKEVVLSEKPLISEETDLIEPTLLDELICHIGSLASVYHKPPNAFVEGSHGIHRKHLPIHHGSTDAGDSPVGTTTATNLEQPQVIPSQGDLLGDLLNLDLGPPVNVPQVSSMQMGAVDLLGGGLDSLVGQSFIPSSVPATFAPSPTPAVVSSGLNDLFELSTGIGMAPGGYVAPKAVWLPAVKAKGLEISGTFTHRQGHIYMEMNFTNKALQHMTDFAIQFNKNSFGVIPSTPLAIHTPLMPNQSIDVSLPLNTLGPVMKMEPLNNLQVAVKNNIDVFYFSCLIPLNVLFVEDGKMERQVFLATWKDIPNENELQFQIKECHLNADTVSSKLQNNNVYTIAKRNVEGQDMLYQSLKLTNGIWILAELRIQPGNPNYTLSLKCRAPEVSQYIYQVYDSILKN

34) >gi|27477041|ref|NP_036437.1| AP-2 complex subunit alpha-2 isoform 2 [Homo sapiens] (103.9 kD)

MPAVSKGDGMRGLAVFISDIRNCKSKEAEIKRINKELANIRSKFKGDKALDGYSKKKYVCKLLFIFLLGHDIDFGHMEAVNLLSSNRYTEKQIGYLFISVLVNSNSELIRLINNAIKNDLASRNPTFMGLALHCIASVGSREMAEAFAGEIPKVLVAGDTMDSVKQSAALCLLRLYRTSPDLVPMGDWTSRVVHLLNDQHLGVVTAATSLITTLAQKNPEEFKTSVSLAVSRLSRIVTSASTDLQDYTYYFVPAPWLSVKLLRLLQCYPPPDPAVRGRLTECLETILNKAQEPPKSKKVQHSNAKNAVLFEAISLIIHHDSEPNLLVRACNQLGQFLQHRETNLRYLALESMCTLASSEFSHEAVKTHIETVINALKTERDVSVRQRAVDLLYAMCDRSNAPQIVAEMLSYLETADYSIREEIVLKVAILAEKYAVDYTWYVDTILNLIRIAGDYVSEEVWYRVIQIVINRDDVQGYAAKTVFEALQAPACHENLVKVGGYILGEFGNLIAGDPRSSPLIQFHLLHSKFHLCSVPTRALLLSTYIKFVNLFPEVKPTIQDVLRSDSQLRNADVELQQRAVEYLRLSTVASTDILATVLEEMPPFPERESSILAKLKKKKGPSTVTDLEDTKRDRSVDVNGGPEPAPASTSAVSTPSPSADLLGLGAAPPAPAGPPPSSGGSGLLVDVFSDSASVVAPLAPGSEDNFARFVCKNNGVLFENQLLQIGLKSEFRQNLGRMFIFYGNKTSTQFLNFTPTLICSDDLQPNLNLQTKPVDPTVEGGAQVQQVVNIECVSDFTEAPVLNIQFRYGGTFQNVSVQLPITLNKFFQPTEMASQDFFQRWKQLSNPQQEVQNIFKAKHPMDTEVTKAKIIGFGSALLEEVDPNPANFVGAGIIHTKTTQIGCLLRLEPNLQAQMYRLTLRTSKEAVSQRLCELLSAQF

35) >gi|4501891|ref|NP_001093.1| alpha-actinin-1 isoform b [Homo sapiens] (103 kD)

MDHYDSQQTNDYMQPEEDWDRDLLLDPAWEKQQRKTFTAWCNSHLRKAGTQIENIEEDFRDGLKLMLLLEVISGERLAKPERGKMRVHKISNVNKALDFIASKGVKLVSIGAEEIVDGNVKMTLGMIWTIILRFAIQDISVEETSAKEGLLLWCQRKTAPYKNVNIQNFHISWKDGLGFCALIHRHRPELIDYGKLRKDDPLTNLNTAFDVAEKYLDIPKMLDAEDIVGTARPDEKAIMTYVSSFYHAFSGAQKAETAANRICKVLAVNQENEQLMEDYEKLASDLLEWIRRTIPWLENRVPENTMHAMQQKLEDFRDYRRLHKPPKVQEKCQLEINFNTLQTKLRLSNRPAFMPSEGRMVSDINNAWGCLEQVEKGYEEWLLNEIRRLERLDHLAEKFRQKASIHEAWTDGKEAMLRQKDYETATLSEIKALLKKHEAFESDLAAHQDRVEQIAAIAQELNELDYYDSPSVNARCQKICDQWDNLGALTQKRREALERTEKLLETIDQLYLEYAKRAAPFNNWMEGAMEDLQDTFIVHTIEEIQGLTTAHEQFKATLPDADKERLAILGIHNEVSKIVQTYHVNMAGTNPYTTITPQEINGKWDHVRQLVPRRDQALTEEHARQQHNERLRKQFGAQANVIGPWIQTKMEEIGRISIEMHGTLEDQLSHLRQYEKSIVNYKPKIDQLEGDHQLIQEALIFDNKHTNYTMEHIRVGWEQLLTTIARTINEVENQILTRDAKGISQEQMNEFRASFNHFDRDHSGTLGPEEFKACLISLGYDIGNDPQGEAEFARIMSIVDPNRLGVVTFQAFIDFMSRETADTDTADQVMASFKILAGDKNYITMDELRRELPPDQAEYCIARMAPYTGPDSVPGALDYMSFSTALYGESDL

36) >gi|222136639|ref|NP_005947.3| C-1-tetrahydrofolate synthase, cytoplasmic [Homo sapiens] (101.5 kD)

MAPAEILNGKEISAQIRARLKNQVTQLKEQVPGFTPRLAILQVGNRDDSNLYINVKLKAAEEIGIKATHIKLPRTTTESEVMKYITSLNEDSTVHGFLVQLPLDSENSINTEEVINAIAPEKDVDGLTSINAGKLARGDLNDCFIPCTPKGCLELIKETGVPIAGRHAVVVGRSKIVGAPMHDLLLWNNATVTTCHSKTAHLDEEVNKGDILVVATGQPEMVKGEWIKPGAIVIDCGINYVPDDKKPNGRKVVGDVAYDEAKERASFITPVPGGVGPMTVAMLMQSTVESAKRFLEKFKPGKWMIQYNNLNLKTPVPSDIDISRSCKPKPIGKLAREIGLLSEEVELYGETKAKVLLSALERLKHRPDGKYVVVTGITPTPLGEGKSTTTIGLVQALGAHLYQNVFACVRQPSQGPTFGIKGGAAGGGYSQVIPMEEFNLHLTGDIHAITAANNLVAAAIDARIFHELTQTDKALFNRLVPSVNGVRRFSDIQIRRLKRLGIEKTDPTTLTDEEINRFARLDIDPETITWQRVLDTNDRFLRKITIGQAPTEKGHTRTAQFDISVASEIMAVLALTTSLEDMRERLGKMVVASSKKGEPVSAEDLGVSGALTVLMKDAIKPNLMQTLEGTPVFVHAGPFANIAHGNSSIIADRIALKLVGPEGFVVTEAGFGADIGMEKFFNIKCRYSGLCPHVVVLVATVRALKMHGGGPTVTAGLPLPKAYIQENLELVEKGFSNLKKQIENARMFGIPVVVAVNAFKTDTESELDLISRLSREHGAFDAVKCTHWAEGGKGALALAQAVQRAAQAPSSFQLLYDLKLPVEDKIRIIAQKIYGADDIELLPEAQHKAEVYTKQGFGNLPICMAKTHLSLSHNPEQKGVPTGFILPIRDIRASVGAGFLYPLVGTMSTMPGLPTRPCFYDIDLDPETEQVNGLF

37) >gi|18079218|ref|NP_065892.1| oxysterol-binding protein-related protein 8 isoform a [Homo sapiens] (101.1 kD)

MEGGLADGEPDRTSLLGDSKDVLGPSTVVANSDESQLLTPGKMSQRQGKEAYPTPTKDLHQPSLSPASPHSQGFERGKEDISQNKDESSLSMSKSKSESKLYNGSEKDSSTSSKLTKKESLKVQKKNYREEKKRATKELLSTITDPSVIVMADWLKIRGTLKSWTKLWCVLKPGVLLIYKTQKNGQWVGTVLLNACEIIERPSKKDGFCFKLFHPLEQSIWAVKGPKGEAVGSITQPLPSSYLIIRATSESDGRCWMDALELALKCSSLLKRTMIREGKEHDLSVSSDSTHVTFYGLLRANNLHSGDNFQLNDSEIERQHFKDQDMYSDKSDKENDQEHDESDNEVMGKSEESDTDTSERQDDSYIEPEPVEPLKETTYTEQSHEELGEAGEASQTETVSEENKSLIWTLLKQVRPGMDLSKVVLPTFILEPRSFLDKLSDYYYHADFLSEAALEENPYFRLKKVVKWYLSGFYKKPKGLKKPYNPILGETFRCLWIHPRTNSKTFYIAEQVSHHPPISAFYVSNRKDGFCLSGSILAKSKFYGNSLSAILEGEARLTFLNRGEDYVMTMPYAHCKGILYGTMTLELGGTVNITCQKTGYSAILEFKLKPFLGSSDCVNQISGKLKLGKEVLATLEGHWDSEVFITDKKTDNSEVFWNPTPDIKQWRLIRHTVKFEEQGDFESEKLWQRVTRAINAKDQTEATQEKYVLEEAQRQAARDRKTKNEEWSCKLFELDPLTGEWHYKFADTRPWDPLNDMIQFEKDGVIQTKVKHRTPMVSVPKMKHKPTRQQKKVAKGYSSPEPDIQDSSGSEAQSVKPSTRRKKGIELGDIQSSIESIKQTQEEIKRNIMALRNHLVSSTPATDYFLQQKDYFIIFLLILLQVIINFMFK

38) >gi|51094101|ref|NP_076977.3| ATP-dependent RNA helicase DDX54 isoform 2 [Homo sapiens] (98.5 kD)

MAADKGPAAGPRSRAAMAQWRKKKGLRKRRGAASQARGSDSEDGEFEIQAEDDARARKLGPGRPLPTFPTSECTSDVEPDTREMVRAQNKKKKKSGGFQSMGLSYPVFKGIMKKGYKVPTPIQRKTIPVILDGKDVVAMARTGSGKTACFLLPMFERLKTHSAQTGARALILSPTRELALQTLKFTKELGKFTGLKTALILGGDRMEDQFAALHENPDIIIATPGRLVHVAVEMSLKLQSVEYVVFDEADRLFEMGFAEQLQEIIARLPGGHQTVLFSATLPKLLVEFARAGLTEPVLIRLDVDTKLNEQLKTSFFLVREDTKAAVLLHLLHNVVRPQDQTVVFVATKHHAEYLTELLTTQRVSCAHIYSALDPTARKINLAKFTLGKCSTLIVTDLAARGLDIPLLDNVINYSFPAKGKLFLHRVGRVARAGRSGTAYSLVAPDEIPYLLDLHLFLGRSLTLARPLKEPSGVAGVDGMLGRVPQSVVDEEDSGLQSTLEASLELRGLARVADNAQQQYVRSRPAPSPESIKRAKEMDLVGLGLHPLFSSRFEEEELQRLRLVDSIKNYRSRATIFEINASSRDLCSQVMRAKRQKDRKAIARFQQGQQGRQEQQEGPVGPAPSRPALQEKQPEKEEEEEAGESVEDIFSEVVGRKRQRSGPNRGAKRRREEARQRDQEFYIPYRPKDFDSERGLSISGEGGAFEQQAAGAVLDLMGDEAQNLTRGRQQLKWDRKKKRFVGQSGQEDKKKIKTESGRYISSSYKRDLYQKWKQKQKIDDRDSDEEGASDRRGPERRGGKRDRGQGASRPHAPGTPAGRVRPELKTKQQILKQRRRAQKLHFLQRGGLKQLSARNRRRVQELQQGAFGRGARSKKGKMRKRM

39) >gi|255918194|ref|NP_001157633.1| serine/threonine-protein phosphatase 6 regulatory subunit 3 isoform 6 [Homo sapiens] (97.6 kD)

MFWKFDLHSSSHIDTLLEREDVTLKELMDEEDVLQECKAQNRKLIEFLLKAECLEDLVSFIIEEPPQDMDEKIRYKYPNISCELLTSDVSQMNDRLGEDESLLMKLYSFLLNDSPLNPLLASFFSKVLSILISRKPEQIVDFLKKKHDFVDLIIKHIGTSAIMDLLLRLLTCIEPPQPRQDVLNWLNEEKIIQRLVEIVHPSQEEDRHSNASQSLCEIVRLSRDQMLQIQNSTEPDPLLATLEKQEIIEQLLSNIFHKEKNESAIVSAIQILLTLLETRRPTFEGHIEICPPGMSHSACSVNKSVLEAIRGRLGSFHELLLEPPKKSVMKTTWGVLDPPVGNTRLNVIRLISSLLQTNTSSINGDLMELNSIGVILNMFFKYTWNNFLHTQVEICIALILASPFENTENATITDQDSTGDNLLLKHLFQKCQLIERILEAWEMNEKKQAEGGRRHGYMGHLTRIANCIVHSTDKGPNSALVQQLIKDLPDEVRERWETFCTSSLGETNKRNTVDLVTTCHIHSSSDDEIDFKETGFSQDSSLQQAFSDYQMQQMTSNFIDQFGFNDEKFADQDDIGNVSFDRVSDINFTLNTNESGNIALFEACCKERIQQFDDGGSDEEDIWEEKHIAFTPESQRRSSSGSTDSEESTDSEEEDGAKQDLFEPSSANTEDKMEVDLSEPPNWSANFDVPMETTHGAPLDSVGSDVWSTEEPMPTKETGWASFSEFTSSLSTKDSLRSNSPVEMETSTEPMDPLTPSAAALAVQPEAAGSVAMEASSDGEEDAESTDKVTETVMNGGMKETLSLTVDAKTETAVFKSEEGKLSTSQDAACKDAEECPETAEAKCAAPRPPSSSPEQRTGQPSAPGDTSVNGPV

40) >gi|42544159|ref|NP_006635.2| heat shock protein 105 kDa isoform 1 [Homo sapiens] (96.8 kD)

MSVVGLDVGSQSCYIAVARAGGIETIANEFSDRCTPSVISFGSKNRTIGVAAKNQQITHANNTVSNFKRFHGRAFNDPFIQKEKENLSYDLVPLKNGGVGIKVMYMGEEHLFSVEQITAMLLTKLKETAENSLKKPVTDCVISVPSFFTDAERRSVLDAAQIVGLNCLRLMNDMTAVALNYGIYKQDLPSLDEKPRIVVFVDMGHSAFQVSACAFNKGKLKVLGTAFDPFLGGKNFDEKLVEHFCAEFKTKYKLDAKSKIRALLRLYQECEKLKKLMSSNSTDLPLNIECFMNDKDVSGKMNRSQFEELCAELLQKIEVPLYSLLEQTHLKVEDVSAVEIVGGATRIPAVKERIAKFFGKDISTTLNADEAVARGCALQCAILSPAFKVREFSVTDAVPFPISLIWNHDSEDTEGVHEVFSRNHAAPFSKVLTFLRRGPFELEAFYSDPQGVPYPEAKIGRFVVQNVSAQKDGEKSRVKVKVRVNTHGIFTISTASMVEKVPTEENEMSSEADMECLNQRPPENPDTDKNVQQDNSEAGTQPQVQTDAQQTSQSPPSPELTSEENKIPDADKANEKKVDQPPEAKKPKIKVVNVELPIEANLVWQLGKDLLNMYIETEGKMIMQDKLEKERNDAKNAVEEYVYEFRDKLCGPYEKFICEQDHQNFLRLLTETEDWLYEEGEDQAKQAYVDKLEELMKIGTPVKVRFQEAEERPKMFEELGQRLQHYAKIAADFRNKDEKYNHIDESEMKKVEKSVNEVMEWMNNVMNAQAKKSLDQDPVVRAQEIKTKIKELNNTCEPVVTQPKPKIESPKLERTPNGPNIDKKEEDLEDKNNFGAEPPHQNGECYPNEKNSVNMDLD

**Figure legend**: These are 40 largest proteins identified from the 55-kD stripe (MCF7). The red underlined sequences are the LC-MS/MS identified peptide fragments that are unique to the protein, while the green underlined sequences are the LC-MS/MS identified peptide fragments that are not unique to the protein but can also appear in other proteins.

**The 20 smallest proteins identified in the 72-kD stripe (MCF7)**

1) >gi|16751921|ref|NP_444513.1| dermcidin preproprotein [Homo sapiens] (11.3 kD)

MRFMTLLFLTALAGALVCAYDPEAASAPGSGNPCHEASAAQKENAGEDPGLARQAPKPRKQRSSLLEKGLDGAKKAVGGLGKLGKDAVEDLESVGKGAVHDVKDVLDSVL

2) >gi|4504301|ref|NP_003529.1| histone H4 [Homo sapiens] (11.4 kD)

MSGRGKGGKGLGKGGAKRHRKVLRDNIQGITKPAIRRLARRGGVKRISGLIYEETRGVLKVFLENVIRDAVTYTEHAKRKTVTAMDVVYALKRQGRTLYGFGG

3) >gi|4502205|ref|NP_001651.1| ADP-ribosylation factor 4 [Homo sapiens] (20.5 kD)

MGLTISSLFSRLFGKKQMRILMVGLDAAGKTTILYKLKLGEIVTTIPTIGFNVETVEYKNICFTVWDVGGQDRIRPLWKHYFQNTQGLIFVVDSNDRERIQEVADELQKMLLVDELRDAVLLLFANKQDLPNAMAISEMTDKLGLQSLRNRTWYVQATCATQGTGLYEGLDWLSNELSKR

4) >gi|320461711|ref|NP_001189360.1| peroxiredoxin-1 [Homo sapiens] (22.1 kD)

MSSGNAKIGHPAPNFKATAVMPDGQFKDISLSDYKGKYVVFFFYPLDFTFVCPTEIIAFSDRAEEFKKLNCQVIGASVDSHFCHLAWVNTPKKQGGLGPMNIPLVSDPKRTIAQDYGVLKADEGISFRGLFIIDDKGILRQITVNDLPVGRSVDETLRLVQAFQFTDKHGEVCPAGWKPGSDTIKPDVQKSKEYFSKQK

5) >gi|5453555|ref|NP_006316.1| GTP-binding nuclear protein Ran [Homo sapiens] (24.4 kD)

MAAQGEPQVQFKLVLVGDGGTGKTTFVKRHLTGEFEKKYVATLGVEVHPLVFHTNRGPIKFNVWDTAGQEKFGGLRDGYYIQAQCAIIMFDVTSRVTYKNVPNWHRDLVRVCENIPIVLCGNKVDIKDRKVKAKSIVFHRKKNLQYYDISAKSNYNFEKPFLWLARKLIGDPNLEFVAMPALAPPEVVMDPALAAQYEHDLEVAQTTALPDEDDDL

6) >gi|67782307|ref|NP_001019636.1| superoxide dismutase [Mn], mitochondrial isoform A precursor [Homo sapiens] (24.7 kD)

MLSRAVCGTSRQLAPVLGYLGSRQKHSLPDLPYDYGALEPHINAQIMQLHHSKHHAAYVNNLNVTEEKYQEALAKGDVTAQIALQPALKFNGGGHINHSIFWTNLSPNGGGEPKGELLEAIKRDFGSFDKFKEKLTAASVGVQGSGWGWLGFNKERGHLQIAACPNQDPLQGTTGLIPLLGIDVWEHAYYLQYKNVRPDYLKAIWNVINWENVTERYMACKK

7) >gi|383872447|ref|NP_001244318.1| CD63 antigen isoform A [Homo sapiens] (25.6 kD)

MAVEGGMKCVKFLLYVLLLAFCACAVGLIAVGVGAQLVLSQTIIQGATPGSLLPVVIIAVGVFLFLVAFVGCCGACKENYCLMITFAIFLSLIMLVEVAAAIAGYVFRDKVMSEFNNNFRQQMENYPKNNHTASILDRMQADFKCCGAANYTDWEKIPSMSKNRVPDSCCINVTVGCGINFNEKAIHKEGCVEKIGGWLRKNVLVVAAAALGIAFVEVLGIVFACCLVKSIRSGYEVM

8) >gi|378548190|ref|NP_001243731.1| 40S ribosomal protein S3 isoform 1 [Homo sapiens] (26.7 kD)

MAVQISKKRKFVADGIFKAELNEFLTRELAEDGYSGVEVRVTPTRTEIIILATRTQNVLGEKGRRIRELTAVVQKRFGFPEGSVELYAEKVATRGLCAIAQAESLRYKLLGGLAVRRACYGVLRFIMESGAKGCEVVVSGKLRGQRAKSMKFVDGLMIHSGDPVNYYVDTAVRHVLLRQGVLGIKVKIMLPWDPTGKIGPKKPLPDHVSIVEPKDEILPTTPISEQKGGKPEPPAMPQPVPTA

9) >gi|4507953|ref|NP_003397.1| 14-3-3 protein zeta/delta [Homo sapiens] (27.7 kD)

MDKNELVQKAKLAEQAERYDDMAACMKSVTEQGAELSNEERNLLSVAYKNVVGARRSSWRVVSSIEQKTEGAEKKQQMAREYREKIETELRDICNDVLSLLEKFLIPNASQAESKVFYLKMKGDYYRYLAEVAAGDDKKGIVDQSQQAYQEAFEISKKEMQPTHPIRLGLALNFSVFYYEILNSPEKACSLAKTAFDEAIAELDTLSEESYKDSTLIMQLLRDNLTLWTSDTQGDEAEAGEGGEN

10) >gi|4502677|ref|NP_001770.1| lymphocyte function-associated antigen 3 isoform 1 [Homo sapiens] (28.1 kD)

MVAGSDAGRALGVLSVVCLLHCFGFISCFSQQIYGVVYGNVTFHVPSNVPLKEVLWKKQKDKVAELENSEFRAFSSFKNRVYLDTVSGSLTIYNLTSSDEDEYEMESPNITDTMKFFLYVLESLPSPTLTCALTNGSIEVQCMIPEHYNSHRGLIMYSWDCPMEQCKRNSTSIYFKMENDLPQKIQCTLSNPLFNTTSSIILTTCIPSSGHSRHRYALIPIPLAVITTCIVLYMNGILKCDRKPDRTNSN

11) >gi|5803225|ref|NP_006752.1| 14-3-3 protein epsilon [Homo sapiens] (29.2 kD)

MDDREDLVYQAKLAEQAERYDEMVESMKKVAGMDVELTVEERNLLSVAYKNVIGARRASWRIISSIEQKEENKGGEDKLKMIREYRQMVETELKLICCDILDVLDKHLIPAANTGESKVFYYKMKGDYHRYLAEFATGNDRKEAAENSLVAYKAASDIAMTELPPTHPIRLGLALNFSVFYYEILNSPDRACRLAKAAFDDAIAELDTLSEESYKDSTLIMQLLRDNLTLWTSDMQGDGEEQNKEALQDVEDENQ

12) >gi|4507879|ref|NP_003365.1| voltage-dependent anion-selective channel protein 1 [Homo sapiens] (30.8 kD)

MAVPPTYADLGKSARDVFTKGYGFGLIKLDLKTKSENGLEFTSSGSANTETTKVTGSLETKYRWTEYGLTFTEKWNTDNTLGTEITVEDQLARGLKLTFDSSFSPNTGKKNAKIKTGYKREHINLGCDMDFDIAGPSIRGALVLGYEGWLAGYQMNFETAKSRVTQSNFAVGYKTDEFQLHTNVNDGTEFGGSIYQKVNKKLETAVNLAWTAGNSNTRFGIAAKYQIDPDACFSAKVNNSSLIGLGYTQTLKPGIKLTLSALLDGKNVNAGGHKLGLGLEFQA

13) >gi|530404479|ref|XP_005268055.1| PREDICTED: serine/arginine-rich splicing factor 5 isoform X1 [Homo sapiens] (31.2 kD)

MSGCRVFIGRLNPAAREKDVERFFKGYGRIRDIDLKRGFGFVEFEDPRDADDAVYELDGKELCSERVTIEHARARSRGGRGRGRYSDRFSSRRPRNDRRNAPPVRTENRLIVENLSSRVSWQDLKDFMRQAGEVTFADAHRPKLNEGVVEFASYGDLKNAIEKLSGKEINGRKIKLIEGSKRHSRSRSRSRSRTRSSSRSRSRSRSRSRKSYSRSRSRSRSRSRSKSRSVSRSPVPEKSQKRGSSSRSKSPASVDRQRSRSRSRSRSVDSGN

14) >gi|9257240|ref|NP_059429.1| neuroplastin isoform a precursor [Homo sapiens] (31.3 kD)

MSGSSLPSALALSLLLVSGSLLPGPGAAQNEPRIVTSEEVIIRDSPVLPVTLQCNLTSSSHTLTYSYWTKNGVELSATRKNASNMEYRINKPRAEDSGEYHCVYHFVSAPKANATIEVKAAPDITGHKRSENKNEGQDATMYCKSVGYPHPDWIWRKKENGMPMDIVNTSGRFFIINKENYTELNIVNLQITEDPGEYECNATNAIGSASVVTVLRVRSHLAPLWPFLGILAEIIILVVIIVVYEKRKRPDEVPDDDEPAGPMKTNSTNNHKDKNLRQRNTN

15) >gi|153070260|ref|NP_002347.5| myristoylated alanine-rich C-kinase substrate [Homo sapiens] (31.5 kD)

MGAQFSKTAAKGEAAAERPGEAAVASSPSKANGQENGHVKVNGDASPAAAESGAKEELQANGSAPAADKEEPAAAGSGAASPSAAEKGEPAAAAAPEAGASPVEKEAPAEGEAAEPGSPTAAEGEAASAASSTSSPKAEDGATPSPSNETPKKKKKRFSFKKSFKLSGFSFKKNKKEAGEGGEAEAPAAEGGKDEAAGGAAAAAAEAGAASGEQAAAPGEEAAAGEEGAAGGDPQEAKPQEAAVAPEKPPASDETKAAEEPSKVEEKKAEEAGASAAACEAPSAAGPGAPPEQEAAPAEEPAAAAASSACAAPSQEAQPECSPEAPPAEAAE

16) >gi|61966711|ref|NP_001013653.1| heterogeneous nuclear ribonucleoprotein C-like 1 [Homo sapiens] (32.1 kD)

MASNVTNKMDPHSMNSRVFIGNLNTLVVKKSDVEAIFSKYGKIAGCSVHKGFAFVQYDKEKNARAAVAGEDGRMIASQVVDINLAAEPKVNRGNAGVKRSAAEMYGSSFDLDYGFQRDYYDGMYSFPARVPPPPPIALAVVPSKRQRLSGNTSRRGKSGFNSKSGKRGSSKSGKLKGDDLQAIKQELTQIKQKVDSLLENLEKIEKEQSKQEVEVKNAKSEEEQSSSSMKKDETHVKMESEGGAEDSAEEGDPLDDDVNEDQGDDQLELIKDDEKEAEEGEDDRDSTNGQDDS

17) >gi|10835063|ref|NP_002511.1| nucleophosmin isoform 1 [Homo sapiens] (32.6 kD)

MEDSMDMDMSPLRPQNYLFGCELKADKDYHFKVDNDENEHQLSLRTVSLGAGAKDELHIVEAEAMNYEGSPIKVTLATLKMSVQPTVSLGGFEITPPVVLRLKCGSGPVHISGQHLVAVEEDAESEDEEEEDVKLLSISGKRSAPGGGSKVPQKKVKLAADEDDDDDDEEDDDEDDDDDDFDDEEAEEKAPVKKSIRDTPAKNAQKSNQNGKDSKPSSTPRSKGQESFKKQEKTPKTPKGPSSVEDIKAKMQASIEKGGSLPKVEAKFINYVKNCFRMTDQEAIQDLWQWRKSL

18) >gi|16753227|ref|NP_000961.2| 60S ribosomal protein L6 [Homo sapiens] (32.7 kD)

MAGEKVEKPDTKEKKPEAKKVDAGGKVKKGNLKAKKPKKGKPHCSRNPVLVRGIGRYSRSAMYSRKAMYKRKYSAAKSKVEKKKKEKVLATVTKPVGGDKNGGTRVVKLRKMPRYYPTEDVPRKLLSHGKKPFSQHVRKLRASITPGTILIILTGRHRGKRVVFLKQLASGLLLVTGPLVLNRVPLRRTHQKFVIATSTKIDISNVKIPKHLTDAYFKKKKLRKPRHQEGEIFDTEKEKYEITEQRKIDQKAVDSQILPKIKAIPQLQGYLRSVFALTNGIYPHKLVF

19) >gi|156071462|ref|NP_001627.2| ADP/ATP translocase 3 [Homo sapiens] (32.8 kD)

MTEQAISFAKDFLAGGIAAAISKTAVAPIERVKLLLQVQHASKQIAADKQYKGIVDCIVRIPKEQGVLSFWRGNLANVIRYFPTQALNFAFKDKYKQIFLGGVDKHTQFWRYFAGNLASGGAAGATSLCFVYPLDFARTRLAADVGKSGTEREFRGLGDCLVKITKSDGIRGLYQGFSVSVQGIIIYRAAYFGVYDTAKGMLPDPKNTHIVVSWMIAQTVTAVAGVVSYPFDTVRRRMMMQSGRKGADIMYTGTVDCWRKIFRDEGGKAFFKGAWSNVLRGMGGAFVLVLYDELKKVI

20) >gi|221307584|ref|NP_001138303.1| prohibitin-2 isoform 1 [Homo sapiens] (33.3 kD)

MAQNLKDLAGRLPAGPRGMGTALKLLLGAGAVAYGVRESVFTVEGGHRAIFFNRIGGVQQDTILAEGLHFRIPWFQYPIIYDIRARPRKISSPTGSKDLQMVNISLRVLSRPNAQELPSMYQRLGLDYEERVLPSIVNEVLKSVVAKFNASQLITQRAQVSLLIRRELTERAKDFSLILDDVAITELSFSREYTAAVEAKQVAQQEAQRAQFLVEKAKQEQRQKIVQAEGEAEAAKMLGEALSKNPGYIKLRKIRAAQNISKTIATSQNRIYLTADNLVLNLQDESFTRGSDSLIKGKK

**Figure legend**: These are 20 smallest proteins identified in the 72-kD stripe (MCF7). The red underlined sequences are the LC-MS/MS identified peptide fragments that are unique to the protein, while the green underlined sequences are the LC-MS/MS identified peptide fragments that are not unique to the protein but can also appear in other proteins.

**The 40 largest proteins in the 72-kD stripe (MCF7)**

1) >gi|61743954|ref|NP_001611.1| neuroblast differentiation-associated protein AHNAK isoform 1 [Homo sapiens] (628.7 kD)

MEKEETTRELLLPNWQGSGSHGLTIAQRDDGVFVQEVTQNSPAARTGVVKEGDQIVGATIYFDNLQSGEVTQLLNTMGHHTVGLKLHRKGDRSPEPGQTWTREVFSSCSSEVVLSGDDEEYQRIYTTKIKPRLKSEDGVEGDLGETQSRTITVTRRVTAYTVDVTGREGAKDIDISSPEFKIKIPRHELTEISNVDVETQSGKTVIRLPSGSGAASPTGSAVDIRAGAISASGPELQGAGHSKLQVTMPGIKVGGSGVNVNAKGLDLGGRGGVQVPAVDISSSLGGRAVEVQGPSLESGDHGKIKFPTMKVPKFGVSTGREGQTPKAGLRVSAPEVSVGHKGGKPGLTIQAPQLEVSVPSANIEGLEGKLKGPQITGPSLEGDLGLKGAKPQGHIGVDASAPQIGGSITGPSVEVQAPDIDVQGPGSKLNVPKMKVPKFSVSGAKGEETGIDVTLPTGEVTVPGVSGDVSLPEIATGGLEGKMKGTKVKTPEMIIQKPKISMQDVDLSLGSPKLKGDIKVSAPGVQGDVKGPQVALKGSRVDIETPNLEGTLTGPRLGSPSGKTGTCRISMSEVDLNVAAPKVKGGVDVTLPRVEGKVKVPEVDVRGPKVDVSAPDVEAHGPEWNLKMPKMKMPTFSTPGAKGEGPDVHMTLPKGDISISGPKVNVEAPDVNLEGLGGKLKGPDVKLPDMSVKTPKISMPDVDLHVKGTKVKGEYDVTVPKLEGELKGPKVDIDAPDVDVHGPDWHLKMPKMKMPKFSVPGFKAEGPEVDVNLPKADVDISGPKIDVTAPDVSIEEPEGKLKGPKFKMPEMNIKVPKISMPDVDLHLKGPNVKGEYDVTMPKVESEIKVPDVELKSAKMDIDVPDVEVQGPDWHLKMPKMKMPKFSMPGFKAEGPEVDVNLPKADVDISGPKVGVEVPDVNIEGPEGKLKGPKFKMPEMNIKAPKISMPDVDLHMKGPKVKGEYDMTVPKLEGDLKGPKVDVSAPDVEMQGPDWNLKMPKIKMPKFSMPSLKGEGPEFDVNLSKANVDISAPKVDTNAPDLSLEGPEGKLKGPKFKMPEMHFRAPKMSLPDVDLDLKGPKMKGNVDISAPKIEGEMQVPDVDIRGPKVDIKAPDVEGQGLDWSLKIPKMKMPKFSMPSLKGEGPEVDVNLPKADVVVSGPKVDIEAPDVSLEGPEGKLKGPKFKMPEMHFKTPKISMPDVDLHLKGPKVKGDVDVSVPKVEGEMKVPDVEIKGPKMDIDAPDVEVQGPDWHLKMPKMKMPKFSMPGFKGEGREVDVNLPKADIDVSGPKVDVEVPDVSLEGPEGKLKGPKFKMPEMHFKAPKISMPDVDLNLKGPKLKGDVDVSLPEVEGEMKVPDVDIKGPKVDISAPDVDVHGPDWHLKMPKVKMPKFSMPGFKGEGPEVDVKLPKADVDVSGPKMDAEVPDVNIEGPDAKLKGPKFKMPEMSIKPQKISIPDVGLHLKGPKMKGDYDVTVPKVEGEIKAPDVDIKGPKVDINAPDVEVHGPDWHLKMPKVKMPKFSMPGFKGEGPEVDMNLPKADLGVSGPKVDIDVPDVNLEAPEGKLKGPKFKMPSMNIQTHKISMPDVGLNLKAPKLKTDVDVSLPKVEGDLKGPEIDVKAPKMDVNVGDIDIEGPEGKLKGPKFKMPEMHFKAPKISMPDVDLHLKGPKVKGDMDVSVPKVEGEMKVPDVDIKGPKVDIDAPDVEVHDPDWHLKMPKMKMPKFSMPGFKAEGPEVDVNLPKADIDVSGPSVDTDAPDLDIEGPEGKLKGSKFKMPKLNIKAPKVSMPDVDLNLKGPKLKGEIDASVPELEGDLRGPQVDVKGPFVEAEVPDVDLECPDAKLKGPKFKMPEMHFKAPKISMPDVDLHLKGPKVKGDADVSVPKLEGDLTGPSVGVEVPDVELECPDAKLKGPKFKMPDMHFKAPKISMPDVDLHLKGPKVKGDVDVSVPKLEGDLTGPSVGVEVPDVELECPDAKLKGPKFKMPEMHFKTPKISMPDVDLHLKGPKVKGDMDVSVPKVEGEMKVPDVDIKGPKMDIDAPDVDVHGPDWHLKMPKMKMPKFSMPGFKAEGPEVDVNLPKADVVVSGPKVDVEVPDVSLEGPEGKLKGPKLKMPEMHFKAPKISMPDVDLHLKGPKVKGDVDVSLPKLEGDLTGPSVDVEVPDVELECPDAKLKGPKFKMPEMHFKTPKISMPDVNLNLKGPKVKGDMDVSVPKVEGEMKVPDVDIRGPKVDIDAPDVDVHGPDWHLKMPKMKMPKFSMPGFKGEGPEVDVNLPKADVDVSGPKVDVEVPDVSLEGPEGKLKGPKFKMPEMHFKTPKISMPDVDFNLKGPKIKGDVDVSAPKLEGELKGPELDVKGPKLDADMPEVAVEGPNGKWKTPKFKMPDMHFKAPKISMPDLDLHLKSPKAKGEVDVDVPKLEGDLKGPHVDVSGPDIDIEGPEGKLKGPKFKMPDMHFKAPNISMPDVDLNLKGPKIKGDVDVSVPEVEGKLEVPDMNIRGPKVDVNAPDVQAPDWHLKMPKMKMPKFSMPGFKAEGPEVDVNLPKADVDISGPKVDIEGPDVNIEGPEGKLKGPKLKMPEMNIKAPKISMPDFDLHLKGPKVKGDVDVSLPKVEGDLKGPEVDIKGPKVDINAPDVGVQGPDWHLKMPKVKMPKFSMPGFKGEGPDGDVKLPKADIDVSGPKVDIEGPDVNIEGPEGKLKGPKFKMPEMNIKAPKISMPDIDLNLKGPKVKGDVDVSLPKVEGDLKGPEVDIKGPKVDIDAPDVDVHGPDWHLKMPKIKMPKISMPGFKGEGPDVDVNLPKADIDVSGPKVDVECPDVNIEGPEGKWKSPKFKMPEMHFKTPKISMPDIDLNLTGPKIKGDVDVTGPKVEGDLKGPEVDLKGPKVDIDVPDVNVQGPDWHLKMPKMKMPKFSMPGFKAEGPEVDVNLPKADVDVSGPKVDVEGPDVNIEGPEGKLKGPKFKMPEMNIKAPKIPMPDFDLHLKGPKVKGDVDISLPKVEGDLKGPEVDIRGPQVDIDVPDVGVQGPDWHLKMPKVKMPKFSMPGFKGEGPDVDVNLPKADLDVSGPKVDIDVPDVNIEGPEGKLKGPKFKMPEMNIKAPKISMPDIDLNLKGPKVKGDMDVSLPKVEGDMKVPDVDIKGPKVDINAPDVDVQGPDWHLKMPKIKMPKISMPGFKGEGPEVDVNLPKADLDVSGPKVDVDVPDVNIEGPDAKLKGPKFKMPEMNIKAPKISMPDLDLNLKGPKMKGEVDVSLANVEGDLKGPALDIKGPKIDVDAPDIDIHGPDAKLKGPKLKMPDMHVNMPKISMPEIDLNLKGSKLKGDVDVSGPKLEGDIKAPSLDIKGPEVDVSGPKLNIEGKSKKSRFKLPKFNFSGSKVQTPEVDVKGKKPDIDITGPKVDINAPDVEVQGKVKGSKFKMPFLSISSPKVSMPDVELNLKSPKVKGDLDIAGPNLEGDFKGPKVDIKAPEVNLNAPDVDVHGPDWNLKMPKMKMPKFSVSGLKAEGPDVAVDLPKGDINIEGPSMNIEGPDLNVEGPEGGLKGPKFKMPDMNIKAPKISMPDIDLNLKGPKVKGDVDISLPKLEGDLKGPEVDIKGPKVDINAPDVDVHGPDWHLKMPKVKMPKFSMPGFKGEGPEVDVTLPKADIDISGPNVDVDVPDVNIEGPDAKLKGPKFKMPEMNIKAPKISMPDFDLNLKGPKMKGDVVVSLPKVEGDLKGPEVDIKGPKVDIDTPDINIEGSEGKFKGPKFKIPEMHLKAPKISMPDIDLNLKGPKVKGDVDVSLPKMEGDLKGPEVDIKGPKVDINAPDVDVQGPDWHLKMPKVKMPKFSMPGFKGEGPDVDVNLPKADLDVSGPKVDIDVPDVNIEGPEGKLKGPKFKMPEMNIKAPKISMPDIDLNLKGPKVKGDMDVSLPKVEGDMQVPDLDIKGPKVDINAPDVDVRGPDWHLKMPKIKMPKISMPGFKGEGPEVDVNLPKADLDVSGPKVDVDVPDVNIEGPDAKLKGPKFKMPEMNIKAPKISMPDFDLHLKGPKVKGDVDVSLPKMEGDLKAPEVDIKGPKVDIDAPDVDVHGPDWHLKMPKVKMPKFSMPGFKGEGPEVDVNLPKADIDVSGPKVDIDTPDIDIHGPEGKLKGPKFKMPDLHLKAPKISMPEVDLNLKGPKMKGDVDVSLPKVEGDLKGPEVDIKGPKVDIDVPDVDVQGPDWHLKMPKVKMPKFSMPGFKGEGPDVDVNLPKADLDVSGPKVDIDVPDVNIEGPDAKLKGPKFKMPEMNIKAPKISMPDFDLHLKGPKVKGDVDVSLPKVEGDLKGPEVDIKGPKVDIDAPDVDVHGPDWHLKMPKVKMPKFSMPGFKGEGPDVDVTLPKADIEISGPKVDIDAPDVSIEGPDAKLKGPKFKMPEMNIKAPKISMPDIDFNLKGPKVKGDVDVSLPKVEGDLKGPEIDIKGPSLDIDTPDVNIEGPEGKLKGPKFKMPEMNIKAPKISMPDFDLHLKGPKVKGDVDVSLPKVESDLKGPEVDIEGPEGKLKGPKFKMPDVHFKSPQISMSDIDLNLKGPKIKGDMDISVPKLEGDLKGPKVDVKGPKVGIDTPDIDIHGPEGKLKGPKFKMPDLHLKAPKISMPEVDLNLKGPKVKGDMDISLPKVEGDLKGPEVDIRDPKVDIDVPDVDVQGPDWHLKMPKVKMPKFSMPGFKGEGPDVDVNLPKADIDVSGPKVDVDVPDVNIEGPDAKLKGPKFKMPEMSIKAPKISMPDIDLNLKGPKVKGDVDVTLPKVEGDLKGPEADIKGPKVDINTPDVDVHGPDWHLKMPKVKMPKFSMPGFKGEGPDVDVSLPKADIDVSGPKVDVDIPDVNIEGPDAKLKGPKFKMPEINIKAPKISIPDVDLDLKGPKVKGDFDVSVPKVEGTLKGPEVDLKGPRLDFEGPDAKLSGPSLKMPSLEISAPKVTAPDVDLHLKAPKIGFSGPKLEGGEVDLKGPKVEAPSLDVHMDSPDINIEGPDVKIPKFKKPKFGFGAKSPKADIKSPSLDVTVPEAELNLETPEISVGGKGKKSKFKMPKIHMSGPKIKAKKQGFDLNVPGGEIDASLKAPDVDVNIAGPDAALKVDVKSPKTKKTMFGKMYFPDVEFDIKSPKFKAEAPLPSPKLEGELQAPDLELSLPAIHVEGLDIKAKAPKVKMPDVDISVPKIEGDLKGPKVQANLGAPDINIEGLDAKVKTPSFGISAPQVSIPDVNVNLKGPKIKGDVPSVGLEGPDVDLQGPEAKIKFPKFSMPKIGIPGVKMEGGGAEVHAQLPSLEGDLRGPDVKLEGPDVSLKGPGVDLPSVNLSMPKVSGPDLDLNLKGPSLKGDLDASVPSMKVHAPGLNLSGVGGKMQVGGDGVKVPGIDATTKLNVGAPDVTLRGPSLQGDLAVSGDIKCPKVSVGAPDLSLEASEGSIKLPKMKLPQFGISTPGSDLHVNAKGPQVSGELKGPGVDVNLKGPRISAPNVDFNLEGPKVKGSLGATGEIKGPTVGGGLPGIGVQGLEGNLQMPGIKSSGCDVNLPGVNVKLPTGQISGPEIKGGLKGSEVGFHGAAPDISVKGPAFNMASPESDFGINLKGPKIKGGADVSGGVSAPDISLGEGHLSVKGSGGEWKGPQVSSALNLDTSKFAGGLHFSGPKVEGGVKGGQIGLQAPGLSVSGPQGHLESGSGKVTFPKMKIPKFTFSGRELVGREMGVDVHFPKAEASIQAGAGDGEWEESEVKLKKSKIKMPKFNFSKPKGKGGVTGSPEASISGSKGDLKSSKASLGSLEGEAEAEASSPKGKFSLFKSKKPRHRSNSFSDEREFSGPSTPTGTLEFEGGEVSLEGGKVKGKHGKLKFGTFGGLGSKSKGHYEVTGSDDETGKLQGSGVSLASKKSRLSSSSSNDSGNKVGIQLPEVELSVSTKKE

2) >gi|41322916|ref|NP_958782.1| plectin isoform 1 [Homo sapiens] (531.5 kD)

MVAGMLMPRDQLRAIYEVLFREGVMVAKKDRRPRSLHPHVPGVTNLQVMRAMASLRARGLVRETFAWCHFYWYLTNEGIAHLRQYLHLPPEIVPASLQRVRRPVAMVMPARRTPHVQAVQGPLGSPPKRGPLPTEEQRVYRRKELEEVSPETPVVPATTQRTLARPGPEPAPATDERDRVQKKTFTKWVNKHLIKAQRHISDLYEDLRDGHNLISLLEVLSGDSLPREKGRMRFHKLQNVQIALDYLRHRQVKLVNIRNDDIADGNPKLTLGLIWTIILHFQISDIQVSGQSEDMTAKEKLLLWSQRMVEGYQGLRCDNFTSSWRDGRLFNAIIHRHKPLLIDMNKVYRQTNLENLDQAFSVAERDLGVTRLLDPEDVDVPQPDEKSIITYVSSLYDAMPRVPDVQDGVRANELQLRWQEYRELVLLLLQWMRHHTAAFEERRFPSSFEEIEILWSQFLKFKEMELPAKEADKNRSKGIYQSLEGAVQAGQLKVPPGYHPLDVEKEWGKLHVAILEREKQLRSEFERLECLQRIVTKLQMEAGLCEEQLNQADALLQSDVRLLAAGKVPQRAGEVERDLDKADSMIRLLFNDVQTLKDGRHPQGEQMYRRVYRLHERLVAIRTEYNLRLKAGVAAPATQVAQVTLQSVQRRPELEDSTLRYLQDLLAWVEENQHRVDGAEWGVDLPSVEAQLGSHRGLHQSIEEFRAKIERARSDEGQLSPATRGAYRDCLGRLDLQYAKLLNSSKARLRSLESLHSFVAAATKELMWLNEKEEEEVGFDWSDRNTNMTAKKESYSALMRELELKEKKIKELQNAGDRLLREDHPARPTVESFQAALQTQWSWMLQLCCCIEAHLKENAAYFQFFSDVREAEGQLQKLQEALRRKYSCDRSATVTRLEDLLQDAQDEKEQLNEYKGHLSGLAKRAKAVVQLKPRHPAHPMRGRLPLLAVCDYKQVEVTVHKGDECQLVGPAQPSHWKVLSSSGSEAAVPSVCFLVPPPNQEAQEAVTRLEAQHQALVTLWHQLHVDMKSLLAWQSLRRDVQLIRSWSLATFRTLKPEEQRQALHSLELHYQAFLRDSQDAGGFGPEDRLMAEREYGSCSHHYQQLLQSLEQGAQEESRCQRCISELKDIRLQLEACETRTVHRLRLPLDKEPARECAQRIAEQQKAQAEVEGLGKGVARLSAEAEKVLALPEPSPAAPTLRSELELTLGKLEQVRSLSAIYLEKLKTISLVIRGTQGAEEVLRAHEEQLKEAQAVPATLPELEATKASLKKLRAQAEAQQPTFDALRDELRGAQEVGERLQQRHGERDVEVERWRERVAQLLERWQAVLAQTDVRQRELEQLGRQLRYYRESADPLGAWLQDARRRQEQIQAMPLADSQAVREQLRQEQALLEEIERHGEKVEECQRFAKQYINAIKDYELQLVTYKAQLEPVASPAKKPKVQSGSESVIQEYVDLRTHYSELTTLTSQYIKFISETLRRMEEEERLAEQQRAEERERLAEVEAALEKQRQLAEAHAQAKAQAEREAKELQQRMQEEVVRREEAAVDAQQQKRSIQEELQQLRQSSEAEIQAKARQAEAAERSRLRIEEEIRVVRLQLEATERQRGGAEGELQALRARAEEAEAQKRQAQEEAERLRRQVQDESQRKRQAEVELASRVKAEAEAAREKQRALQALEELRLQAEEAERRLRQAEVERARQVQVALETAQRSAEAELQSKRASFAEKTAQLERSLQEEHVAVAQLREEAERRAQQQAEAERAREEAERELERWQLKANEALRLRLQAEEVAQQKSLAQAEAEKQKEEAEREARRRGKAEEQAVRQRELAEQELEKQRQLAEGTAQQRLAAEQELIRLRAETEQGEQQRQLLEEELARLQREAAAATQKRQELEAELAKVRAEMEVLLASKARAEEESRSTSEKSKQRLEAEAGRFRELAEEAARLRALAEEAKRQRQLAEEDAARQRAEAERVLAEKLAAIGEATRLKTEAEIALKEKEAENERLRRLAEDEAFQRRRLEEQAAQHKADIEERLAQLRKASDSELERQKGLVEDTLRQRRQVEEEILALKASFEKAAAGKAELELELGRIRSNAEDTLRSKEQAELEAARQRQLAAEEERRRREAEERVQKSLAAEEEAARQRKAALEEVERLKAKVEEARRLRERAEQESARQLQLAQEAAQKRLQAEEKAHAFAVQQKEQELQQTLQQEQSVLDQLRGEAEAARRAAEEAEEARVQAEREAAQSRRQVEEAERLKQSAEEQAQARAQAQAAAEKLRKEAEQEAARRAQAEQAALRQKQAADAEMEKHKKFAEQTLRQKAQVEQELTTLRLQLEETDHQKNLLDEELQRLKAEATEAARQRSQVEEELFSVRVQMEELSKLKARIEAENRALILRDKDNTQRFLQEEAEKMKQVAEEAARLSVAAQEAARLRQLAEEDLAQQRALAEKMLKEKMQAVQEATRLKAEAELLQQQKELAQEQARRLQEDKEQMAQQLAEETQGFQRTLEAERQRQLEMSAEAERLKLRVAEMSRAQARAEEDAQRFRKQAEEIGEKLHRTELATQEKVTLVQTLEIQRQQSDHDAERLREAIAELEREKEKLQQEAKLLQLKSEEMQTVQQEQLLQETQALQQSFLSEKDSLLQRERFIEQEKAKLEQLFQDEVAKAQQLREEQQRQQQQMEQERQRLVASMEEARRRQHEAEEGVRRKQEELQQLEQQRRQQEELLAEENQRLREQLQLLEEQHRAALAHSEEVTASQVAATKTLPNGRDALDGPAAEAEPEHSFDGLRRKVSAQRLQEAGILSAEELQRLAQGHTTVDELARREDVRHYLQGRSSIAGLLLKATNEKLSVYAALQRQLLSPGTALILLEAQAASGFLLDPVRNRRLTVNEAVKEGVVGPELHHKLLSAERAVTGYKDPYTGQQISLFQAMQKGLIVREHGIRLLEAQIATGGVIDPVHSHRVPVDVAYRRGYFDEEMNRVLADPSDDTKGFFDPNTHENLTYLQLLERCVEDPETGLCLLPLTDKAAKGGELVYTDSEARDVFEKATVSAPFGKFQGKTVTIWEIINSEYFTAEQRRDLLRQFRTGRITVEKIIKIIITVVEEQEQKGRLCFEGLRSLVPAAELLESRVIDRELYQQLQRGERSVRDVAEVDTVRRALRGANVIAGVWLEEAGQKLSIYNALKKDLLPSDMAVALLEAQAGTGHIIDPATSARLTVDEAVRAGLVGPEFHEKLLSAEKAVTGYRDPYTGQSVSLFQALKKGLIPREQGLRLLDAQLSTGGIVDPSKSHRVPLDVACARGCLDEETSRALSAPRADAKAYSDPSTGEPATYGELQQRCRPDQLTGLSLLPLSEKAARARQEELYSELQARETFEKTPVEVPVGGFKGRTVTVWELISSEYFTAEQRQELLRQFRTGKVTVEKVIKILITIVEEVETLRQERLSFSGLRAPVPASELLASGVLSRAQFEQLKDGKTTVKDLSELGSVRTLLQGSGCLAGIYLEDTKEKVSIYEAMRRGLLRATTAALLLEAQAATGFLVDPVRNQRLYVHEAVKAGVVGPELHEQLLSAEKAVTGYRDPYSGSTISLFQAMQKGLVLRQHGIRLLEAQIATGGIIDPVHSHRVPVDVAYQRGYFSEEMNRVLADPSDDTKGFFDPNTHENLTYRQLLERCVEDPETGLRLLPLKGAEKAEVVETTQVYTEEETRRAFEETQIDIPGGGSHGGSTMSLWEVMQSDLIPEEQRAQLMADFQAGRVTKERMIIIIIEIIEKTEIIRQQGLASYDYVRRRLTAEDLFEARIISLETYNLLREGTRSLREALEAESAWCYLYGTGSVAGVYLPGSRQTLSIYQALKKGLLSAEVARLLLEAQAATGFLLDPVKGERLTVDEAVRKGLVGPELHDRLLSAERAVTGYRDPYTEQTISLFQAMKKELIPTEEALRLLDAQLATGGIVDPRLGFHLPLEVAYQRGYLNKDTHDQLSEPSEVRSYVDPSTDERLSYTQLLRRCRRDDGTGQLLLPLSDARKLTFRGLRKQITMEELVRSQVMDEATALQLREGLTSIEEVTKNLQKFLEGTSCIAGVFVDATKERLSVYQAMKKGIIRPGTAFELLEAQAATGYVIDPIKGLKLTVEEAVRMGIVGPEFKDKLLSAERAVTGYKDPYSGKLISLFQAMKKGLILKDHGIRLLEAQIATGGIIDPEESHRLPVEVAYKRGLFDEEMNEILTDPSDDTKGFFDPNTEENLTYLQLMERCITDPQTGLCLLPLKEKKRERKTSSKSSVRKRRVVIVDPETGKEMSVYEAYRKGLIDHQTYLELSEQECEWEEITISSSDGVVKSMIIDRRSGRQYDIDDAIAKNLIDRSALDQYRAGTLSITEFADMLSGNAGGFRSRSSSVGSSSSYPISPAVSRTQLASWSDPTEETGPVAGILDTETLEKVSITEAMHRNLVDNITGQRLLEAQACTGGIIDPSTGERFPVTDAVNKGLVDKIMVDRINLAQKAFCGFEDPRTKTKMSAAQALKKGWLYYEAGQRFLEVQYLTGGLIEPDTPGRVPLDEALQRGTVDARTAQKLRDVGAYSKYLTCPKTKLKISYKDALDRSMVEEGTGLRLLEAAAQSTKGYYSPYSVSGSGSTAGSRTGSRTGSRAGSRRGSFDATGSGFSMTFSSSSYSSSGYGRRYASGSSASLGGPESAVA

3) >gi|61676188|ref|NP_113584.3| E3 ubiquitin-protein ligase HUWE1 [Homo sapiens] (481.6 kD)

MKVDRTKLKKTPTEAPADCRALIDKLKVCNDEQLLLELQQIKTWNIGKCELYHWVDLLDRFDGILADAGQTVENMSWMLVCDRPEREQLKMLLLAVLNFTALLIEYSFSRHLYSSIEHLTTLLASSDMQVVLAVLNLLYVFSKRSNYITRLGSDKRTPLLTRLQHLAESWGGKENGFGLAECCRDLHMMKYPPSATTLHFEFYADPGAEVKIEKRTTSNTLHYIHIEQLDKISESPSEIMESLTKMYSIPKDKQMLLFTHIRLAHGFSNHRKRLQAVQARLHAISILVYSNALQESANSILYNGLIEELVDVLQITDKQLMEIKAASLRTLTSIVHLERTPKLSSIIDCTGTASYHGFLPVLVRNCIQAMIDPSMDPYPHQFATALFSFLYHLASYDAGGEALVSCGMMEALLKVIKFLGDEQDQITFVTRAVRVVDLITNLDMAAFQSHSGLSIFIYRLEHEVDLCRKECPFVIKPKIQRPNTTQEGEEMETDMDGVQCIPQRAALLKSMLNFLKKAIQDPAFSDGIRHVMDGSLPTSLKHIISNAEYYGPSLFLLATEVVTVFVFQEPSLLSSLQDNGLTDVMLHALLIKDVPATREVLGSLPNVFSALCLNARGLQSFVQCQPFERLFKVLLSPDYLPAMRRRRSSDPLGDTASNLGSAVDELMRHQPTLKTDATTAIIKLLEEICNLGRDPKYICQKPSIQKADGTATAPPPRSNHAAEEASSEDEEEEEVQAMQSFNSTQQNETEPNQQVVGTEERIPIPLMDYILNVMKFVESILSNNTTDDHCQEFVNQKGLLPLVTILGLPNLPIDFPTSAACQAVAGVCKSILTLSHEPKVLQEGLLQLDSILSSLEPLHRPIESPGGSVLLRELACAGNVADATLSAQATPLLHALTAAHAYIMMFVHTCRVGQSEIRSISVNQWGSQLGLSVLSKLSQLYCSLVWESTVLLSLCTPNSLPSGCEFGQADMQKLVPKDEKAGTTQGGKRSDGEQDGAAGSMDASTQGLLEGIGLDGDTLAPMETDEPTASDSKGKSKITPAMAARIKQIKPLLSASSRLGRALAELFGLLVKLCVGSPVRQRRSHHAASTTTAPTPAARSTASALTKLLTKGLSWQPPPYTPTPRFRLTFFICSVGFTSPMLFDERKYPYHLMLQKFLCSGGHNALFETFNWALSMGGKVPVSEGLEHSDLPDGTGEFLDAWLMLVEKMVNPTTVLESPHSLPAKLPGGVQNFPQFSALRFLVVTQKAAFTCIKNLWNRKPLKVYGGRMAESMLAILCHILRGEPVIRERLSKEKEGSRGEEDTGQEEGGSRREPQVNQQQLQQLMDMGFTREHAMEALLNTSTMEQATEYLLTHPPPIMGGVVRDLSMSEEDQMMRAIAMSLGQDIPMDQRAESPEEVACRKEEEERKAREKQEEEEAKCLEKFQDADPLEQDELHTFTDTMLPGCFHLLDELPDTVYRVCDLIMTAIKRNGADYRDMILKQVVNQVWEAADVLIKAALPLTTSDTKTVSEWISQMATLPQASNLATRILLLTLLFEELKLPCAWVVESSGILNVLIKLLEVVQPCLQAAKEQKEVQTPKWITPVLLLIDFYEKTAISSKRRAQMTKYLQSNSNNWRWFDDRSGRWCSYSASNNSTIDSAWKSGETSVRFTAGRRRYTVQFTTMVQVNEETGNRRPVMLTLLRVPRLNKNSKNSNGQELEKTLEESKEMDIKRKENKGNDTPLALESTNTEKETSLEETKIGEILIQGLTEDMVTVLIRACVSMLGVPVDPDTLHATLRLCLRLTRDHKYAMMFAELKSTRMILNLTQSSGFNGFTPLVTLLLRHIIEDPCTLRHTMEKVVRSAATSGAGSTTSGVVSGSLGSREINYILRVLGPAACRNPDIFTEVANCCIRIALPAPRGSGTASDDEFENLRIKGPNAVQLVKTTPLKPSPLPVIPDTIKEVIYDMLNALAAYHAPEEADKSDPKPGVMTQEVGQLLQDMGDDVYQQYRSLTRQSSDFDTQSGFSINSQVFAADGASTETSASGTSQGEASTPEESRDGKKDKEGDRASEEGKQKGKGSKPLMPTSTILRLLAELVRSYVGIATLIANYSYTVGQSELIKEDCSVLAFVLDHLLPHTQNAEDKDTPALARLFLASLAAAGSGTDAQVALVNEVKAALGRALAMAESTEKHARLQAVMCIISTIMESCPSTSSFYSSATAKTQHNGMNNIIRLFLKKGLVNDLARVPHSLDLSSPNMANTVNAALKPLETLSRIVNQPSSLFGSKSASSKNKSEQDAQGASQDSSSNQQDPGEPGEAEVQEEDHDVTQTEVADGDIMDGEAETDSVVIAGQPEVLSSQEMQVENELEDLIDELLERDGGSGNSTIIVSRSGEDESQEDVLMDEAPSNLSQASTLQANREDSMNILDPEDEEEHTQEEDSSGSNEDEDDSQDEEEEEEEDEEDDQEDDEGEEGDEDDDDDGSEMELDEDYPDMNASPLVRFERFDREDDLIIEFDNMFSSATDIPPSPGNIPTTHPLMVRHADHSSLTLGSGSSTTRLTQGIGRSQRTLRQLTANTGHTIHVHYPGNRQPNPPLILQRLLGPSAAADILQLSSSLPLQSRGRARLLVGNDDVHIIARSDDELLDDFFHDQSTATSQAGTLSSIPTALTRWTEECKVLDAESMHDCVSVVKVSIVNHLEFLRDEELEERREKRRKQLAEEETKITDKGKEDKENRDQSAQCTASKSNDSTEQNLSDGTPMPDSYPTTPSSTDAATSESKETLGTLQSSQQQPTLPTPPALGEVPQELQSPAGEGGSSTQLLMPVEPEELGPTRPSGEAETTQMELSPAPTITSLSPERAEDSDALTAVSSQLEGSPMDTSSLASCTLEEAVGDTSAAGSSEQPRAGSSTPGDAPPAVAEVQGRSDGSGESAQPPEDSSPPASSESSSTRDSAVAISGADSRGILEEPLPSTSSEEEDPLAGISLPEGVDPSFLAALPDDIRREVLQNQLGIRPPTRTAPSTNSSAPAVVGNPGVTEVSPEFLAALPPAIQEEVLAQQRAEQQRRELAQNASSDTPMDPVTFIQTLPSDLRRSVLEDMEDSVLAVMPPDIAAEAQALRREQEARQRQLMHERLFGHSSTSALSAILRSPAFTSRLSGNRGVQYTRLAVQRGGTFQMGGSSSHNRPSGSNVDTLLRLRGRLLLDHEALSCLLVLLFVDEPKLNTSRLHRVLRNLCYHAQTRHWVIRSLLSILQRSSESELCIETPKLTTSEEKGKKSSKSCGSSSHENRPLDLLHKMESKSSNQLSWLSVSMDAALGCRTNIFQIQRSGGRKHTEKHASGGSTVHIHPQAAPVVCRHVLDTLIQLAKVFPSHFTQQRTKETNCESDRERGNKACSPCSSQSSSSGICTDFWDLLVKLDNMNVSRKGKNSVKSVPVSAGGEGETSPYSLEASPLGQLMNMLSHPVIRRSSLLTEKLLRLLSLISIALPENKVSEAQANSGSGASSTTTATSTTSTTTTTAASTTPTPPTAPTPVTSAPALVAATAISTIVVAASTTVTTPTTATTTVSISPTTKGSKSPAKVSDGGSSSTDFKMVSSGLTENQLQLSVEVLTSHSCSEEGLEDAANVLLQLSRGDSGTRDTVLKLLLNGARHLGYTLCKQIGTLLAELREYNLEQQRRAQCETLSPDGLPEEQPQTTKLKGKMQSRFDMAENVVIVASQKRPLGGRELQLPSMSMLTSKTSTQKFFLRVLQVIIQLRDDTRRANKKAKQTGRLGSSGLGSASSIQAAVRQLEAEADAIIQMVREGQRARRQQQAATSESSQSEASVRREESPMDVDQPSPSAQDTQSIASDGTPQGEKEKEERPPELPLLSEQLSLDELWDMLGECLKELEESHDQHAVLVLQPAVEAFFLVHATERESKPPVRDTRESQLAHIKDEPPPLSPAPLTPATPSSLDPFFSREPSSMHISSSLPPDTQKFLRFAETHRTVLNQILRQSTTHLADGPFAVLVDYIRVLDFDVKRKYFRQELERLDEGLRKEDMAVHVRRDHVFEDSYRELHRKSPEEMKNRLYIVFEGEEGQDAGGLLREWYMIISREMFNPMYALFRTSPGDRVTYTINPSSHCNPNHLSYFKFVGRIVAKAVYDNRLLECYFTRSFYKHILGKSVRYTDMESEDYHFYQGLVYLLENDVSTLGYDLTFSTEVQEFGVCEVRDLKPNGANILVTEENKKEYVHLVCQMRMTGAIRKQLAAFLEGFYEIIPKRLISIFTEQELELLISGLPTIDIDDLKSNTEYHKYQSNSIQIQWFWRALRSFDQADRAKFLQFVTGTSKVPLQGFAALEGMNGIQKFQIHRDDRSTDRLPSAHTCFNQLDLPAYESFEKLRHMLLLAIQECSEGFGLA

4) >gi|13654237|ref|NP_008835.5| DNA-dependent protein kinase catalytic subunit isoform 1 [Homo sapiens] (468.8 kD)

MAGSGAGVRCSLLRLQETLSAADRCGAALAGHQLIRGLGQECVLSSSPAVLALQTSLVFSRDFGLLVFVRKSLNSIEFRECREEILKFLCIFLEKMGQKIAPYSVEIKNTCTSVYTKDRAAKCKIPALDLLIKLLQTFRSSRLMDEFKIGELFSKFYGELALKKKIPDTVLEKVYELLGLLGEVHPSEMINNAENLFRAFLGELKTQMTSAVREPKLPVLAGCLKGLSSLLCNFTKSMEEDPQTSREIFNFVLKAIRPQIDLKRYAVPSAGLRLFALHASQFSTCLLDNYVSLFEVLLKWCAHTNVELKKAALSALESFLKQVSNMVAKNAEMHKNKLQYFMEQFYGIIRNVDSNNKELSIAIRGYGLFAGPCKVINAKDVDFMYVELIQRCKQMFLTQTDTGDDRVYQMPSFLQSVASVLLYLDTVPEVYTPVLEHLVVMQIDSFPQYSPKMQLVCCRAIVKVFLALAAKGPVLRNCISTVVHQGLIRICSKPVVLPKGPESESEDHRASGEVRTGKWKVPTYKDYVDLFRHLLSSDQMMDSILADEAFFSVNSSSESLNHLLYDEFVKSVLKIVEKLDLTLEIQTVGEQENGDEAPGVWMIPTSDPAANLHPAKPKDFSAFINLVEFCREILPEKQAEFFEPWVYSFSYELILQSTRLPLISGFYKLLSITVRNAKKIKYFEGVSPKSLKHSPEDPEKYSCFALFVKFGKEVAVKMKQYKDELLASCLTFLLSLPHNIIELDVRAYVPALQMAFKLGLSYTPLAEVGLNALEEWSIYIDRHVMQPYYKDILPCLDGYLKTSALSDETKNNWEVSALSRAAQKGFNKVVLKHLKKTKNLSSNEAISLEEIRIRVVQMLGSLGGQINKNLLTVTSSDEMMKSYVAWDREKRLSFAVPFREMKPVIFLDVFLPRVTELALTASDRQTKVAACELLHSMVMFMLGKATQMPEGGQGAPPMYQLYKRTFPVLLRLACDVDQVTRQLYEPLVMQLIHWFTNNKKFESQDTVALLEAILDGIVDPVDSTLRDFCGRCIREFLKWSIKQITPQQQEKSPVNTKSLFKRLYSLALHPNAFKRLGASLAFNNIYREFREEESLVEQFVFEALVIYMESLALAHADEKSLGTIQQCCDAIDHLCRIIEKKHVSLNKAKKRRLPRGFPPSASLCLLDLVKWLLAHCGRPQTECRHKSIELFYKFVPLLPGNRSPNLWLKDVLKEEGVSFLINTFEGGGCGQPSGILAQPTLLYLRGPFSLQATLCWLDLLLAALECYNTFIGERTVGALQVLGTEAQSSLLKAVAFFLESIAMHDIIAAEKCFGTGAAGNRTSPQEGERYNYSKCTVVVRIMEFTTTLLNTSPEGWKLLKKDLCNTHLMRVLVQTLCEPASIGFNIGDVQVMAHLPDVCVNLMKALKMSPYKDILETHLREKITAQSIEELCAVNLYGPDAQVDRSRLAAVVSACKQLHRAGLLHNILPSQSTDLHHSVGTELLSLVYKGIAPGDERQCLPSLDLSCKQLASGLLELAFAFGGLCERLVSLLLNPAVLSTASLGSSQGSVIHFSHGEYFYSLFSETINTELLKNLDLAVLELMQSSVDNTKMVSAVLNGMLDQSFRERANQKHQGLKLATTILQHWKKCDSWWAKDSPLETKMAVLALLAKILQIDSSVSFNTSHGSFPEVFTTYISLLADTKLDLHLKGQAVTLLPFFTSLTGGSLEELRRVLEQLIVAHFPMQSREFPPGTPRFNNYVDCMKKFLDALELSQSPMLLELMTEVLCREQQHVMEELFQSSFRRIARRGSCVTQVGLLESVYEMFRKDDPRLSFTRQSFVDRSLLTLLWHCSLDALREFFSTIVVDAIDVLKSRFTKLNESTFDTQITKKMGYYKILDVMYSRLPKDDVHAKESKINQVFHGSCITEGNELTKTLIKLCYDAFTENMAGENQLLERRRLYHCAAYNCAISVICCVFNELKFYQGFLFSEKPEKNLLIFENLIDLKRRYNFPVEVEVPMERKKKYIEIRKEAREAANGDSDGPSYMSSLSYLADSTLSEEMSQFDFSTGVQSYSYSSQDPRPATGRFRRREQRDPTVHDDVLELEMDELNRHECMAPLTALVKHMHRSLGPPQGEEDSVPRDLPSWMKFLHGKLGNPIVPLNIRLFLAKLVINTEEVFRPYAKHWLSPLLQLAASENNGGEGIHYMVVEIVATILSWTGLATPTGVPKDEVLANRLLNFLMKHVFHPKRAVFRHNLEIIKTLVECWKDCLSIPYRLIFEKFSGKDPNSKDNSVGIQLLGIVMANDLPPYDPQCGIQSSEYFQALVNNMSFVRYKEVYAAAAEVLGLILRYVMERKNILEESLCELVAKQLKQHQNTMEDKFIVCLNKVTKSFPPLADRFMNAVFFLLPKFHGVLKTLCLEVVLCRVEGMTELYFQLKSKDFVQVMRHRDDERQKVCLDIIYKMMPKLKPVELRELLNPVVEFVSHPSTTCREQMYNILMWIHDNYRDPESETDNDSQEIFKLAKDVLIQGLIDENPGLQLIIRNFWSHETRLPSNTLDRLLALNSLYSPKIEVHFLSLATNFLLEMTSMSPDYPNPMFEHPLSECEFQEYTIDSDWRFRSTVLTPMFVETQASQGTLQTRTQEGSLSARWPVAGQIRATQQQHDFTLTQTADGRSSFDWLTGSSTDPLVDHTSPSSDSLLFAHKRSERLQRAPLKSVGPDFGKKRLGLPGDEVDNKVKGAAGRTDLLRLRRRFMRDQEKLSLMYARKGVAEQKREKEIKSELKMKQDAQVVLYRSYRHGDLPDIQIKHSSLITPLQAVAQRDPIIAKQLFSSLFSGILKEMDKFKTLSEKNNITQKLLQDFNRFLNTTFSFFPPFVSCIQDISCQHAALLSLDPAAVSAGCLASLQQPVGIRLLEEALLRLLPAELPAKRVRGKARLPPDVLRWVELAKLYRSIGEYDVLRGIFTSEIGTKQITQSALLAEARSDYSEAAKQYDEALNKQDWVDGEPTEAEKDFWELASLDCYNHLAEWKSLEYCSTASIDSENPPDLNKIWSEPFYQETYLPYMIRSKLKLLLQGEADQSLLTFIDKAMHGELQKAILELHYSQELSLLYLLQDDVDRAKYYIQNGIQSFMQNYSSIDVLLHQSRLTKLQSVQALTEIQEFISFISKQGNLSSQVPLKRLLNTWTNRYPDAKMDPMNIWDDIITNRCFFLSKIEEKLTPLPEDNSMNVDQDGDPSDRMEVQEQEEDISSLIRSCKFSMKMKMIDSARKQNNFSLAMKLLKELHKESKTRDDWLVSWVQSYCRLSHCRSRSQGCSEQVLTVLKTVSLLDENNVSSYLSKNILAFRDQNILLGTTYRIIANALSSEPACLAEIEEDKARRILELSGSSSEDSEKVIAGLYQRAFQHLSEAVQAAEEEAQPPSWSCGPAAGVIDAYMTLADFCDQQLRKEEENASVIDSAELQAYPALVVEKMLKALKLNSNEARLKFPRLLQIIERYPEETLSLMTKEISSVPCWQFISWISHMVALLDKDQAVAVQHSVEEITDNYPQAIVYPFIISSESYSFKDTSTGHKNKEFVARIKSKLDQGGVIQDFINALDQLSNPELLFKDWSNDVRAELAKTPVNKKNIEKMYERMYAALGDPKAPGLGAFRRKFIQTFGKEFDKHFGKGGSKLLRMKLSDFNDITNMLLLKMNKDSKPPGNLKECSPWMSDFKVEFLRNELEIPGQYDGRGKPLPEYHVRIAGFDERVTVMASLRRPKRIIIRGHDEREHPFLVKGGEDLRQDQRVEQLFQVMNGILAQDSACSQRALQLRTYSVVPMTSRLGLIEWLENTVTLKDLLLNTMSQEEKAAYLSDPRAPPCEYKDWLTKMSGKHDVGAYMLMYKGANRTETVTSFRKRESKVPADLLKRAFVRMSTSPEAFLALRSHFASSHALICISHWILGIGDRHLNNFMVAMETGGVIGIDFGHAFGSATQFLPVPELMPFRLTRQFINLMLPMKETGLMYSIMVHALRAFRSDPGLLTNTMDVFVKEPSFDWKNFEQKMLKKGGSWIQEINVAEKNWYPRQKICYAKRKLAGANPAVITCDELLLGHEKAPAFRDYVAVARGSKDHNIRAQEPESGLSEETQVKCLMDQATDPNILGRTWEGWEPWM

5) >gi|58530840|ref|NP_004406.2| desmoplakin isoform I [Homo sapiens] (331.6 kD)

MSCNGGSHPRINTLGRMIRAESGPDLRYEVTSGGGGTSRMYYSRRGVITDQNSDGYCQTGTMSRHQNQNTIQELLQNCSDCLMRAELIVQPELKYGDGIQLTRSRELDECFAQANDQMEILDSLIREMRQMGQPCDAYQKRLLQLQEQMRALYKAISVPRVRRASSKGGGGYTCQSGSGWDEFTKHVTSECLGWMRQQRAEMDMVAWGVDLASVEQHINSHRGIHNSIGDYRWQLDKIKADLREKSAIYQLEEEYENLLKASFERMDHLRQLQNIIQATSREIMWINDCEEEELLYDWSDKNTNIAQKQEAFSIRMSQLEVKEKELNKLKQESDQLVLNQHPASDKIEAYMDTLQTQWSWILQITKCIDVHLKENAAYFQFFEEAQSTEAYLKGLQDSIRKKYPCDKNMPLQHLLEQIKELEKEREKILEYKRQVQNLVNKSKKIVQLKPRNPDYRSNKPIILRALCDYKQDQKIVHKGDECILKDNNERSKWYVTGPGGVDMLVPSVGLIIPPPNPLAVDLSCKIEQYYEAILALWNQLYINMKSLVSWHYCMIDIEKIRAMTIAKLKTMRQEDYMKTIADLELHYQEFIRNSQGSEMFGDDDKRKIQSQFTDAQKHYQTLVIQLPGYPQHQTVTTTEITHHGTCQDVNHNKVIETNRENDKQETWMLMELQKIRRQIEHCEGRMTLKNLPLADQGSSHHITVKINELKSVQNDSQAIAEVLNQLKDMLANFRGSEKYCYLQNEVFGLFQKLENINGVTDGYLNSLCTVRALLQAILQTEDMLKVYEARLTEEETVCLDLDKVEAYRCGLKKIKNDLNLKKSLLATMKTELQKAQQIHSQTSQQYPLYDLDLGKFGEKVTQLTDRWQRIDKQIDFRLWDLEKQIKQLRNYRDNYQAFCKWLYDAKRRQDSLESMKFGDSNTVMRFLNEQKNLHSEISGKRDKSEEVQKIAELCANSIKDYELQLASYTSGLETLLNIPIKRTMIQSPSGVILQEAADVHARYIELLTRSGDYYRFLSEMLKSLEDLKLKNTKIEVLEEELRLARDANSENCNKNKFLDQNLQKYQAECSQFKAKLASLEELKRQAELDGKSAKQNLDKCYGQIKELNEKITRLTYEIEDEKRRRKSVEDRFDQQKNDYDQLQKARQCEKENLGWQKLESEKAIKEKEYEIERLRVLLQEEGTRKREYENELAKVRNHYNEEMSNLRNKYETEINITKTTIKEISMQKEDDSKNLRNQLDRLSRENRDLKDEIVRLNDSILQATEQRRRAEENALQQKACGSEIMQKKQHLEIELKQVMQQRSEDNARHKQSLEEAAKTIQDKNKEIERLKAEFQEEAKRRWEYENELSKVRNNYDEEIISLKNQFETEINITKTTIHQLTMQKEEDTSGYRAQIDNLTRENRSLSEEIKRLKNTLTQTTENLRRVEEDIQQQKATGSEVSQRKQQLEVELRQVTQMRTEESVRYKQSLDDAAKTIQDKNKEIERLKQLIDKETNDRKCLEDENARLQRVQYDLQKANSSATETINKLKVQEQELTRLRIDYERVSQERTVKDQDITRFQNSLKELQLQKQKVEEELNRLKRTASEDSCKRKKLEEELEGMRRSLKEQAIKITNLTQQLEQASIVKKRSEDDLRQQRDVLDGHLREKQRTQEELRRLSSEVEALRRQLLQEQESVKQAHLRNEHFQKAIEDKSRSLNESKIEIERLQSLTENLTKEHLMLEEELRNLRLEYDDLRRGRSEADSDKNATILELRSQLQISNNRTLELQGLINDLQRERENLRQEIEKFQKQALEASNRIQESKNQCTQVVQERESLLVKIKVLEQDKARLQRLEDELNRAKSTLEAETRVKQRLECEKQQIQNDLNQWKTQYSRKEEAIRKIESEREKSEREKNSLRSEIERLQAEIKRIEERCRRKLEDSTRETQSQLETERSRYQREIDKLRQRPYGSHRETQTECEWTVDTSKLVFDGLRKKVTAMQLYECQLIDKTTLDKLLKGKKSVEEVASEIQPFLRGAGSIAGASASPKEKYSLVEAKRKKLISPESTVMLLEAQAATGGIIDPHRNEKLTVDSAIARDLIDFDDRQQIYAAEKAITGFDDPFSGKTVSVSEAIKKNLIDRETGMRLLEAQIASGGVVDPVNSVFLPKDVALARGLIDRDLYRSLNDPRDSQKNFVDPVTKKKVSYVQLKERCRIEPHTGLLLLSVQKRSMSFQGIRQPVTVTELVDSGILRPSTVNELESGQISYDEVGERIKDFLQGSSCIAGIYNETTKQKLGIYEAMKIGLVRPGTALELLEAQAATGFIVDPVSNLRLPVEEAYKRGLVGIEFKEKLLSAERAVTGYNDPETGNIISLFQAMNKELIEKGHGIRLLEAQIATGGIIDPKESHRLPVDIAYKRGYFNEELSEILSDPSDDTKGFFDPNTEENLTYLQLKERCIKDEETGLCLLPLKEKKKQVQTSQKNTLRKRRVVIVDPETNKEMSVQEAYKKGLIDYETFKELCEQECEWEEITITGSDGSTRVVLVDRKTGSQYDIQDAIDKGLVDRKFFDQYRSGSLSLTQFADMISLKNGVGTSSSMGSGVSDDVFSSSRHESVSKISTISSVRNLTIRSSSFSDTLEESSPIAAIFDTENLEKISITEGIERGIVDSITGQRLLEAQACTGGIIHPTTGQKLSLQDAVSQGVIDQDMATRLKPAQKAFIGFEGVKGKKKMSAAEAVKEKWLPYEAGQRFLEFQYLTGGLVDPEVHGRISTEEAIRKGFIDGRAAQRLQDTSSYAKILTCPKTKLKISYKDAINRSMVEDITGLRLLEAASVSSKGLPSPYNMSSAPGSRSGSRSGSRSGSRSGSRSGSRRGSFDATGNSSYSYSYSFSSSSIGH

6) >gi|15147337|ref|NP_056986.2| E3 ubiquitin-protein ligase UBR5 isoform 1 [Homo sapiens] (309.2 kD)

MTSIHFVVHPLPGTEDQLNDRLREVSEKLNKYNLNSHPPLNVLEQATIKQCVVGPNHAAFLLEDGRVCRIGFSVQPDRLELGKPDNNDGSKLNSNSGAGRTSRPGRTSDSPWFLSGSETLGRLAGNTLGSRWSSGVGGSGGGSSGRSSAGARDSRRQTRVIRTGRDRGSGLLGSQPQPVIPASVIPEELISQAQVVLQGKSRSVIIRELQRTNLDVNLAVNNLLSRDDEDGDDGDDTASESYLPGEDLMSLLDADIHSAHPSVIIDADAMFSEDISYFGYPSFRRSSLSRLGSSRVLLLPLERDSELLRERESVLRLRERRWLDGASFDNERGSTSKEGEPNLDKKNTPVQSPVSLGEDLQWWPDKDGTKFICIGALYSELLAVSSKGELYQWKWSESEPYRNAQNPSLHHPRATFLGLTNEKIVLLSANSIRATVATENNKVATWVDETLSSVASKLEHTAQTYSELQGERIVSLHCCALYTCAQLENSLYWWGVVPFSQRKKMLEKARAKNKKPKSSAGISSMPNITVGTQVCLRNNPLYHAGAVAFSISAGIPKVGVLMESVWNMNDSCRFQLRSPESLKNMEKASKTTEAKPESKQEPVKTEMGPPPSPASTCSDASSIASSASMPYKRRRSTPAPKEEEKVNEEQWSLREVVFVEDVKNVPVGKVLKVDGAYVAVKFPGTSSNTNCQNSSGPDADPSSLLQDCRLLRIDELQVVKTGGTPKVPDCFQRTPKKLCIPEKTEILAVNVDSKGVHAVLKTGNWVRYCIFDLATGKAEQENNFPTSSIAFLGQNERNVAIFTAGQESPIILRDGNGTIYPMAKDCMGGIRDPDWLDLPPISSLGMGVHSLINLPANSTIKKKAAVIIMAVEKQTLMQHILRCDYEACRQYLMNLEQAVVLEQNLQMLQTFISHRCDGNRNILHACVSVCFPTSNKETKEEEEAERSERNTFAERLSAVEAIANAISVVSSNGPGNRAGSSSSRSLRLREMMRRSLRAAGLGRHEAGASSSDHQDPVSPPIAPPSWVPDPPAMDPDGDIDFILAPAVGSLTTAATGTGQGPSTSTIPGPSTEPSVVESKDRKANAHFILKLLCDSVVLQPYLRELLSAKDARGMTPFMSAVSGRAYPAAITILETAQKIAKAEISSSEKEEDVFMGMVCPSGTNPDDSPLYVLCCNDTCSFTWTGAEHINQDIFECRTCGLLESLCCCTECARVCHKGHDCKLKRTSPTAYCDCWEKCKCKTLIAGQKSARLDLLYRLLTATNLVTLPNSRGEHLLLFLVQTVARQTVEHCQYRPPRIREDRNRKTASPEDSDMPDHDLEPPRFAQLALERVLQDWNALKSMIMFGSQENKDPLSASSRIGHLLPEEQVYLNQQSGTIRLDCFTHCLIVKCTADILLLDTLLGTLVKELQNKYTPGRREEAIAVTMRFLRSVARVFVILSVEMASSKKKNNFIPQPIGKCKRVFQALLPYAVEELCNVAESLIVPVRMGIARPTAPFTLASTSIDAMQGSEELFSVEPLPPRPSSDQSSSSSQSQSSYIIRNPQQRRISQSQPVRGRDEEQDDIVSADVEEVEVVEGVAGEEDHHDEQEEHGEENAEAEGQHDEHDEDGSDMELDLLAAAETESDSESNHSNQDNASGRRSVVTAATAGSEAGASSVPAFFSEDDSQSNDSSDSDSSSSQSDDIEQETFMLDEPLERTTNSSHANGAAQAPRSMQWAVRNTQHQRAASTAPSSTSTPAASSAGLIYIDPSNLRRSGTISTSAAAAAAALEASNASSYLTSASSLARAYSIVIRQISDLMGLIPKYNHLVYSQIPAAVKLTYQDAVNLQNYVEEKLIPTWNWMVSIMDSTEAQLRYGSALASAGDPGHPNHPLHASQNSARRERMTAREEASLRTLEGRRRATLLSARQGMMSARGDFLNYALSLMRSHNDEHSDVLPVLDVCSLKHVAYVFQALIYWIKAMNQQTTLDTPQLERKRTRELLELGIDNEDSEHENDDDTNQSATLNDKDDDSLPAETGQNHPFFRRSDSMTFLGCIPPNPFEVPLAEAIPLADQPHLLQPNARKEDLFGRPSQGLYSSSASSGKCLMEVTVDRNCLEVLPTKMSYAANLKNVMNMQNRQKKEGEEQPVLPEETESSKPGPSAHDLAAQLKSSLLAEIGLTESEGPPLTSFRPQCSFMGMVISHDMLLGRWRLSLELFGRVFMEDVGAEPGSILTELGGFEVKESKFRREMEKLRNQQSRDLSLEVDRDRDLLIQQTMRQLNNHFGRRCATTPMAVHRVKVTFKDEPGEGSGVARSFYTAIAQAFLSNEKLPNLECIQNANKGTHTSLMQRLRNRGERDREREREREMRRSSGLRAGSRRDRDRDFRRQLSIDTRPFRPASEGNPSDDPEPLPAHRQALGERLYPRVQAMQPAFASKITGMLLELSPAQLLLLLASEDSLRARVDEAMELIIAHGRENGADSILDLGLVDSSEKVQQENRKRHGSSRSVVDMDLDDTDDGDDNAPLFYQPGKRGFYTPRPGKNTEARLNCFRNIGRILGLCLLQNELCPITLNRHVIKVLLGRKVNWHDFAFFDPVMYESLRQLILASQSSDADAVFSAMDLAFAIDLCKEEGGGQVELIPNGVNIPVTPQNVYEYVRKYAEHRMLVVAEQPLHAMRKGLLDVLPKNSLEDLTAEDFRLLVNGCGEVNVQMLISFTSFNDESGENAEKLLQFKRWFWSIVEKMSMTERQDLVYFWTSSPSLPASEEGFQPMPSITIRPPDDQHLPTANTCISRLYVPLYSSKQILKQKLLLAIKTKNFGFV

7) >gi|54607053|ref|NP_006827.1| translational activator GCN1 [Homo sapiens] (292.6 kD)

MAADTQVSETLKRFAGKVTTASVKERREILSELGKCVAGKDLPEGAVKGLCKLFCLTLHRYRDAASRRALQAAIQQLAEAQPEATAKNLLHSLQSSGIGSKAGVPSKSSGSAALLALTWTCLLVRIVFPSRAKRQGDIWNKLVEVQCLLLLEVLGGSHKHAVDGAVKKLTKLWKENPGLVEQYLSAILSLEPNQNYAGMLGLLVQFCTSHKEMDVVSQHKSALLDFYMKNILMSKVKPPKYLLDSCAPLLRYLSHSEFKDLILPTIQKSLLRSPENVIETISSLLASVTLDLSQYAMDIVKGLAGHLKSNSPRLMDEAVLALRNLARQCSDSSAMESLTKHLFAILGGSEGKLTVVAQKMSVLSGIGSVSHHVVSGPSSQVLNGIVAELFIPFLQQEVHEGTLVHAVSVLALWCNRFTMEVPKKLTEWFKKAFSLKTSTSAVRHAYLQCMLASYRGDTLLQALDLLPLLIQTVEKAASQSTQVPTITEGVAAALLLLKLSVADSQAEAKLSSFWQLIVDEKKQVFTSEKFLVMASEDALCTVLHLTERLFLDHPHRLTGNKVQQYHRALVAVLLSRTWHVRRQAQQTVRKLLSSLGGFKLAHGLLEELKTVLSSHKVLPLEALVTDAGEVTEAGKAYVPPRVLQEALCVISGVPGLKGDVTDTEQLAQEMLIISHHPSLVAVQSGLWPALLARMKIDPEAFITRHLDQIIPRMTTQSPLNQSSMNAMGSLSVLSPDRVLPQLISTITASVQNPALRLVTREEFAIMQTPAGELYDKSIIQSAQQDSIKKANMKRENKAYSFKEQIIELELKEEIKKKKGIKEEVQLTSKQKEMLQAQLDREAQVRRRLQELDGELEAALGLLDIILAKNPSGLTQYIPVLVDSFLPLLKSPLAAPRIKNPFLSLAACVMPSRLKALGTLVSHVTLRLLKPECVLDKSWCQEELSVAVKRAVMLLHTHTITSRVGKGEPGAAPLSAPAFSLVFPFLKMVLTEMPHHSEEEEEWMAQILQILTVQAQLRASPNTPPGRVDENGPELLPRVAMLRLLTWVIGTGSPRLQVLASDTLTTLCASSSGDDGCAFAEQEEVDVLLCALQSPCASVRETVLRGLMELHMVLPAPDTDEKNGLNLLRRLWVVKFDKEEEIRKLAERLWSMMGLDLQPDLCSLLIDDVIYHEAAVRQAGAEALSQAVARYQRQAAEVMGRLMEIYQEKLYRPPPVLDALGRVISESPPDQWEARCGLALALNKLSQYLDSSQVKPLFQFFVPDALNDRHPDVRKCMLDAALATLNTHGKENVNSLLPVFEEFLKNAPNDASYDAVRQSVVVLMGSLAKHLDKSDPKVKPIVAKLIAALSTPSQQVQESVASCLPPLVPAIKEDAGGMIQRLMQQLLESDKYAERKGAAYGLAGLVKGLGILSLKQQEMMAALTDAIQDKKNFRRREGALFAFEMLCTMLGKLFEPYVVHVLPHLLLCFGDGNQYVREAADDCAKAVMSNLSAHGVKLVLPSLLAALEEESWRTKAGSVELLGAMAYCAPKQLSSCLPNIVPKLTEVLTDSHVKVQKAGQQALRQIGSVIRNPEILAIAPVLLDALTDPSRKTQKCLQTLLDTKFVHFIDAPSLALIMPIVQRAFQDRSTDTRKMAAQIIGNMYSLTDQKDLAPYLPSVTPGLKASLLDPVPEVRTVSAKALGAMVKGMGESCFEDLLPWLMETLTYEQSSVDRSGAAQGLAEVMAGLGVEKLEKLMPEIVATASKVDIAPHVRDGYIMMFNYLPITFGDKFTPYVGPIIPCILKALADENEFVRDTALRAGQRVISMYAETAIALLLPQLEQGLFDDLWRIRFSSVQLLGDLLFHISGVTGKMTTETASEDDNFGTAQSNKAIITALGVERRNRVLAGLYMGRSDTQLVVRQASLHVWKIVVSNTPRTLREILPTLFGLLLGFLASTCADKRTIAARTLGDLVRKLGEKILPEIIPILEEGLRSQKSDERQGVCIGLSEIMKSTSRDAVLYFSESLVPTARKALCDPLEEVREAAAKTFEQLHSTIGHQALEDILPFLLKQLDDEEVSEFALDGLKQVMAIKSRVVLPYLVPKLTTPPVNTRVLAFLSSVAGDALTRHLGVILPAVMLALKEKLGTPDEQLEMANCQAVILSVEDDTGHRIIIEDLLEATRSPEVGMRQAAAIILNIYCSRSKADYTSHLRSLVSGLIRLFNDSSPVVLEESWDALNAITKKLDAGNQLALIEELHKEIRLIGNESKGEHVPGFCLPKKGVTSILPVLREGVLTGSPEQKEEAAKALGLVIRLTSADALRPSVVSITGPLIRILGDRFSWNVKAALLETLSLLLAKVGIALKPFLPQLQTTFTKALQDSNRGVRLKAADALGKLISIHIKVDPLFTELLNGIRAMEDPGVRDTMLQALRFVIQGAGAKVDAVIRKNIVSLLLSMLGHDEDNTRISSAGCLGELCAFLTEEELSAVLQQCLLADVSGIDWMVRHGRSLALSVAVNVAPGRLCAGRYSSDVQEMILSSATADRIPIAVSGVRGMGFLMRHHIETGGGQLPAKLSSLFVKCLQNPSSDIRLVAEKMIWWANKDPLPPLDPQAIKPILKALLDNTKDKNTVVRAYSDQAIVNLLKMRQGEEVFQSLSKILDVASLEVLNEVNRRSLKKLASQADSTEQVDDTILT

8) >gi|57864582|ref|NP_001009931.1| hornerin [Homo sapiens] (282.2 kD)

MPKLLQGVITVIDVFYQYATQHGEYDTLNKAELKELLENEFHQILKNPNDPDTVDIILQSLDRDHNKKVDFTEYLLMIFKLVQARNKIIGKDYCQVSGSKLRDDTHQHQEEQEETEKEENKRQESSFSHSSWSAGENDSYSRNVRGSLKPGTESISRRLSFQRDFSGQHNSYSGQSSSYGEQNSDSHQSSGRGQCGSGSGQSPNYGQHGSGSGQSSSNDTHGSGSGQSSGFSQHKSSSGQSSGYSQHGSGSGHSSGYGQHGSRSGQSSRGERHRSSSGSSSSYGQHGSGSRQSLGHGRQGSGSRQSPSHVRHGSGSGHSSSHGQHGSGSSYSYSRGHYESGSGQTSGFGQHESGSGQSSGYSKHGSGSGHSSSQGQHGSTSGQASSSGQHGSSSRQSSSYGQHESASRHSSGRGQHSSGSGQSPGHGQRGSGSGQSPSSGQHGTGFGRSSSSGPYVSGSGYSSGFGHHESSSEHSSGYTQHGSGSGHSSGHGQHGSRSGQSSRGERQGSSAGSSSSYGQHGSGSRQSLGHSRHGSGSGQSPSPSRGRHESGSRQSSSYGPHGYGSGRSSSRGPYESGSGHSSGLGHQESRSGQSSGYGQHGSSSGHSSTHGQHGSTSGQSSSCGQHGATSGQSSSHGQHGSGSSQSSRYGQQGSGSGQSPSRGRHGSDFGHSSSYGQHGSGSGWSSSNGPHGSVSGQSSGFGHKSGSGQSSGYSQHGSGSSHSSGYRKHGSRSGQSSRSEQHGSSSGLSSSYGQHGSGSHQSSGHGRQGSGSGHSPSRVRHGSSSGHSSSHGQHGSGTSCSSSCGHYESGSGQASGFGQHESGSGQGYSQHGSASGHFSSQGRHGSTSGQSSSSGQHDSSSGQSSSYGQHESASHHASGRGRHGSGSGQSPGHGQRGSGSGQSPSYGRHGSGSGRSSSSGRHGSGSGQSSGFGHKSSSGQSSGYTQHGSGSGHSSSYEQHGSRSGQSSRSEQHGSSSGSSSSYGQHGSGSRQSLGHGQHGSGSGQSPSPSRGRHGSGSGQSSSYGPYRSGSGWSSSRGPYESGSGHSSGLGHRESRSGQSSGYGQHGSSSGHSSTHGQHGSTSGQSSSCGQHGASSGQSSSHGQHGSGSSQSSGYGRQGSGSGQSPGHGQRGSGSRQSPSYGRHGSGSGRSSSSGQHGSGLGESSGFGHHESSSGQSSSYSQHGSGSGHSSGYGQHGSRSGQSSRGERHGSSSGSSSHYGQHGSGSRQSSGHGRQGSGSGHSPSRGRHGSGLGHSSSHGQHGSGSGRSSSRGPYESRSGHSSVFGQHESGSGHSSAYSQHGSGSGHFCSQGQHGSTSGQSSTFDQEGSSTGQSSSYGHRGSGSSQSSGYGRHGAGSGQSPSRGRHGSGSGHSSSYGQHGSGSGWSSSSGRHGSGSGQSSGFGHHESSSWQSSGCTQHGSGSGHSSSYEQHGSRSGQSSRGERHGSSSGSSSSYGQHGSGSRQSLGHGQHGSGSGQSPSPSRGRHGSGSGQSSSYSPYGSGSGWSSSRGPYESGSSHSSGLGHRESRSGQSSGYGQHGSSSGHSSTHGQHGSTSGQSSSCGQHGASSGQSSSHGQHGSGSSQSSGYGRQGSGSGQSPGHGQRGSGSRQSPSYGRHGSGSGRSSSSGQHGSGLGESSGFGHHESSSGQSSSYSQHGSGSGHSSGYGQHGSRSGQSSRGERHGSSSRSSSRYGQHGSGSRQSSGHGRQGSGSGQSPSRGRHGSGLGHSSSHGQHGSGSGRSSSRGPYESRSGHSSVFGQHESGSGHSSAYSQHGSGSGHFCSQGQHGSTSGQSSTFDQEGSSTGQSSSHGQHGSGSSQSSSYGQQGSGSGQSPSRGRHGSGSGHSSSYGQHGSGSGWSSSSGRHGSGSGQSSGFGHHESSSWQSSGYTQHGSGSGHSSSYEQHGSRSGQSSRGEQHGSSSGSSSSYGQHGSGSRQSLGHGQHGSGSGQSPSPSRGRHGSGSGQSSSYGPYGSGSGWSSSRGPYESGSGHSSGLGHRESRSGQSSGYGQHGSSSGHSSTHGQHGSASGQSSSCGQHGASSGQSSSHGQHGSGSSQSSGYGRQGSGSGQSPGHGQRGSGSRQSPSYGRHGSGSGRSSSSGQHGPGLGESSGFGHHESSSGQSSSYSQHGSGSGHSSGYGQHGSRSGQSSRGERHGSSSGSSSRYGQHGSGSRQSSGHGRQGSGSGHSPSRGRHGSGSGHSSSHGQHGSGSGRSSSRGPYESRSGHSSVFGQHESGSGHSSAYSQHGSGSGHFCSQGQHGSTSGQSSTFDQEGSSTGQSSSHGQHGSGSSQSSSYGQQGSGSGQSPSRGRHGSGSGHSSSYGQHGSGSGWSSSSGRHGSGSGQSSGFGHHESSSWQSSGYTQHGSGSGHSSSYEQHGSRSGQSSRGERHGSSSGSSSSYGQHGSGSRQSLGHGQHGSGSGQSPSPSRGRHGSGSGQSSSYSPYGSGSGWSSSRGPYESGSGHSSGLGHRESRSGQSSGYGQHGSSSGHSSTHGQHGSTSGQSSSCGQHGASSGQSSSHGQHGSGSSQSSGYGRQGSGSGQSPGHGQRGSGSRQSPSYGRHGSGSGRSSSSGQHGSGLGESSGFGHHESSSGQSSSYSQHGSGSGHSSGYGQHGSRSGQSSRGERHGSSSGSSSHYGQHGSGSRQSSGHGRQGSGSGQSPSRGRHGSGLGHSSSHGQHGSGSGRSSSRGPYESRLGHSSVFGQHESGSGHSSAYSQHGSGSGHFCSQGQHGSTSGQSSTFDQEGSSTGQSSSYGHRGSGSSQSSGYGRHGAGSGQSLSHGRHGSGSGQSSSYGQHGSGSGQSSGYSQHGSGSGQDGYSYCKGGSNHDGGSSGSYFLSFPSSTSPYEYVQEQRCYFYQ

9) >gi|105990514|ref|NP_001448.2| filamin-B isoform 2 [Homo sapiens] (278 kD)

MPVTEKDLAEDAPWKKIQQNTFTRWCNEHLKCVNKRIGNLQTDLSDGLRLIALLEVLSQKRMYRKYHQRPTFRQMQLENVSVALEFLDRESIKLVSIDSKAIVDGNLKLILGLVWTLILHYSISMPVWEDEGDDDAKKQTPKQRLLGWIQNKIPYLPITNFNQNWQDGKALGALVDSCAPGLCPDWESWDPQKPVDNAREAMQQADDWLGVPQVITPEEIIHPDVDEHSVMTYLSQFPKAKLKPGAPLKPKLNPKKARAYGRGIEPTGNMVKQPAKFTVDTISAGQGDVMVFVEDPEGNKEEAQVTPDSDKNKTYSVEYLPKVTGLHKVTVLFAGQHISKSPFEVSVDKAQGDASKVTAKGPGLEAVGNIANKPTYFDIYTAGAGVGDIGVEVEDPQGKNTVELLVEDKGNQVYRCVYKPMQPGPHVVKIFFAGDTIPKSPFVVQVGEACNPNACRASGRGLQPKGVRIRETTDFKVDTKAAGSGELGVTMKGPKGLEELVKQKDFLDGVYAFEYYPSTPGRYSIAITWGGHHIPKSPFEVQVGPEAGMQKVRAWGPGLHGGIVGRSADFVVESIGSEVGSLGFAIEGPSQAKIEYNDQNDGSCDVKYWPKEPGEYAVHIMCDDEDIKDSPYMAFIHPATGGYNPDLVRAYGPGLEKSGCIVNNLAEFTVDPKDAGKAPLKIFAQDGEGQRIDIQMKNRMDGTYACSYTPVKAIKHTIAVVWGGVNIPHSPYRVNIGQGSHPQKVKVFGPGVERSGLKANEPTHFTVDCTEAGEGDVSVGIKCDARVLSEDEEDVDFDIIHNANDTFTVKYVPPAAGRYTIKVLFASQEIPASPFRVKVDPSHDASKVKAEGPGLSKAGVENGKPTHFTVYTKGAGKAPLNVQFNSPLPGDAVKDLDIIDNYDYSHTVKYTPTQQGNMQVLVTYGGDPIPKSPFTVGVAAPLDLSKIKLNGLENRVEVGKDQEFTVDTRGAGGQGKLDVTILSPSRKVVPCLVTPVTGRENSTAKFIPREEGLYAVDVTYDGHPVPGSPYTVEASLPPDPSKVKAHGPGLEGGLVGKPAEFTIDTKGAGTGGLGLTVEGPCEAKIECSDNGDGTCSVSYLPTKPGEYFVNILFEEVHIPGSPFKADIEMPFDPSKVVASGPGLEHGKVGEAGLLSVDCSEAGPGALGLEAVSDSGTKAEVSIQNNKDGTYAVTYVPLTAGMYTLTMKYGGELVPHFPARVKVEPAVDTSRIKVFGPGIEGKDVFREATTDFTVDSRPLTQVGGDHIKAHIANPSGASTECFVTDNADGTYQVEYTPFEKGLHVVEVTYDDVPIPNSPFKVAVTEGCQPSRVQAQGPGLKEAFTNKPNVFTVVTRGAGIGGLGITVEGPSESKINCRDNKDGSCSAEYIPFAPGDYDVNITYGGAHIPGSPFRVPVKDVVDPSKVKIAGPGLGSGVRARVLQSFTVDSSKAGLAPLEVRVLGPRGLVEPVNVVDNGDGTHTVTYTPSQEGPYMVSVKYADEEIPRSPFKVKVLPTYDASKVTASGPGLSSYGVPASLPVDFAIDARDAGEGLLAVQITDQEGKPKRAIVHDNKDGTYAVTYIPDKTGRYMIGVTYGGDDIPLSPYRIRATQTGDASKCLATGPGIASTVKTGEEVGFVVDAKTAGKGKVTCTVLTPDGTEAEADVIENEDGTYDIFYTAAKPGTYVIYVRFGGVDIPNSPFTVMATDGEVTAVEEAPVNACPPGFRPWVTEEAYVPVSDMNGLGFKPFDLVIPFAVRKGEITGEVHMPSGKTATPEIVDNKDGTVTVRYAPTEVGLHEMHIKYMGSHIPESPLQFYVNYPNSGSVSAYGPGLVYGVANKTATFTIVTEDAGEGGLDLAIEGPSKAEISCIDNKDGTCTVTYLPTLPGDYSILVKYNDKHIPGSPFTAKITDDSRRCSQVKLGSAADFLLDISETDLSSLTASIKAPSGRDEPCLLKRLPNNHIGISFIPREVGEHLVSIKKNGNHVANSPVSIMVVQSEIGDARRAKVYGRGLSEGRTFEMSDFIVDTRDAGYGGISLAVEGPSKVDIQTEDLEDGTCKVSYFPTVPGVYIVSTKFADEHVPGSPFTVKISGEGRVKESITRTSRAPSVATVGSICDLNLKIPEINSSDMSAHVTSPSGRVTEAEIVPMGKNSHCVRFVPQEMGVHTVSVKYRGQHVTGSPFQFTVGPLGEGGAHKVRAGGPGLERGEAGVPAEFSIWTREAGAGGLSIAVEGPSKAEITFDDHKNGSCGVSYIAQEPGNYEVSIKFNDEHIPESPYLVPVIAPSDDARRLTVMSLQESGLKVNQPASFAIRLNGAKGKIDAKVHSPSGAVEECHVSELEPDKYAVRFIPHENGVHTIDVKFNGSHVVGSPFKVRVGEPGQAGNPALVSAYGTGLEGGTTGIQSEFFINTTRAGPGTLSVTIEGPSKVKMDCQETPEGYKVMYTPMAPGNYLISVKYGGPNHIVGSPFKAKVTGQRLVSPGSANETSSILVESVTRSSTETCYSAIPKASSDASKVTSKGAGLSKAFVGQKSSFLVDCSKAGSNMLLIGVHGPTTPCEEVSMKHVGNQQYNVTYVVKERGDYVLAVKWGEEHIPGSPFHVTVP

10) >gi|114155142|ref|NP_003283.2| nucleoprotein TPR [Homo sapiens] (267.1 kD)

MAAVLQQVLERTELNKLPKSVQNKLEKFLADQQSEIDGLKGRHEKFKVESEQQYFEIEKRLSHSQERLVNETRECQSLRLELEKLNNQLKALTEKNKELEIAQDRNIAIQSQFTRTKEELEAEKRDLIRTNERLSQELEYLTEDVKRLNEKLKESNTTKGELQLKLDELQASDVSVKYREKRLEQEKELLHSQNTWLNTELKTKTDELLALGREKGNEILELKCNLENKKEEVSRLEEQMNGLKTSNEHLQKHVEDLLTKLKEAKEQQASMEEKFHNELNAHIKLSNLYKSAADDSEAKSNELTRAVEELHKLLKEAGEANKAIQDHLLEVEQSKDQMEKEMLEKIGRLEKELENANDLLSATKRKGAILSEEELAAMSPTAAAVAKIVKPGMKLTELYNAYVETQDQLLLEKLENKRINKYLDEIVKEVEAKAPILKRQREEYERAQKAVASLSVKLEQAMKEIQRLQEDTDKANKQSSVLERDNRRMEIQVKDLSQQIRVLLMELEEARGNHVIRDEEVSSADISSSSEVISQHLVSYRNIEELQQQNQRLLVALRELGETREREEQETTSSKITELQLKLESALTELEQLRKSRQHQMQLVDSIVRQRDMYRILLSQTTGVAIPLHASSLDDVSLASTPKRPSTSQTVSTPAPVPVIESTEAIEAKAALKQLQEIFENYKKEKAENEKIQNEQLEKLQEQVTDLRSQNTKISTQLDFASKRYEMLQDNVEGYRREITSLHERNQKLTATTQKQEQIINTMTQDLRGANEKLAVAEVRAENLKKEKEMLKLSEVRLSQQRESLLAEQRGQNLLLTNLQTIQGILERSETETKQRLSSQIEKLEHEISHLKKKLENEVEQRHTLTRNLDVQLLDTKRQLDTETNLHLNTKELLKNAQKEIATLKQHLSNMEVQVASQSSQRTGKGQPSNKEDVDDLVSQLRQTEEQVNDLKERLKTSTSNVEQYQAMVTSLEESLNKEKQVTEEVRKNIEVRLKESAEFQTQLEKKLMEVEKEKQELQDDKRRAIESMEQQLSELKKTLSSVQNEVQEALQRASTALSNEQQARRDCQEQAKIAVEAQNKYERELMLHAADVEALQAAKEQVSKMASVRQHLEETTQKAESQLLECKASWEERERMLKDEVSKCVCRCEDLEKQNRLLHDQIEKLSDKVVASVKEGVQGPLNVSLSEEGKSQEQILEILRFIRREKEIAETRFEVAQVESLRYRQRVELLERELQELQDSLNAEREKVQVTAKTMAQHEELMKKTETMNVVMETNKMLREEKERLEQDLQQMQAKVRKLELDILPLQEANAELSEKSGMLQAEKKLLEEDVKRWKARNQHLVSQQKDPDTEEYRKLLSEKEVHTKRIQQLTEEIGRLKAEIARSNASLTNNQNLIQSLKEDLNKVRTEKETIQKDLDAKIIDIQEKVKTITQVKKIGRRYKTQYEELKAQQDKVMETSAQSSGDHQEQHVSVQEMQELKETLNQAETKSKSLESQVENLQKTLSEKETEARNLQEQTVQLQSELSRLRQDLQDRTTQEEQLRQQITEKEEKTRKAIVAAKSKIAHLAGVKDQLTKENEELKQRNGALDQQKDELDVRITALKSQYEGRISRLERELREHQERHLEQRDEPQEPSNKVPEQQRQITLKTTPASGERGIASTSDPPTANIKPTPVVSTPSKVTAAAMAGNKSTPRASIRPMVTPATVTNPTTTPTATVMPTTQVESQEAMQSEGPVEHVPVFGSTSGSVRSTSPNVQPSISQPILTVQQQTQATAFVQPTQQSHPQIEPANQELSSNIVEVVQSSPVERPSTSTAVFGTVSATPSSSLPKRTREEEEDSTIEASDQVSDDTVEMPLPKKLKSVTPVGTEEEVMAEESTDGEVETQVYNQDSQDSIGEGVTQGDYTPMEDSEETSQSLQIDLGPLQSDQQTTTSSQDGQGKGDDVIVIDSDDEEEDDDENDGEHEDYEEDEEDDDDDEDDTGMGDEGEDSNEGTGSADGNDGYEADDAEGGDGTDPGTETEESMGGGEGNHRAADSQNSGEGNTGAAESSFSQEVSREQQPSSASERQAPRAPQSPRRPPHPLPPRLTIHAPPQELGPPVQRIQMTRRQSVGRGLQLTPGIGGMQQHFFDDEDRTVPSTPTLVVPHRTDGFAEAIHSPQVAGVPRFRFGPPEDMPQTSSSHSDLGQLASQGGLGMYETPLFLAHEEESGGRSVPTTPLQVAAPVTVFTESTTSDASEHASQSVPMVTTSTGTLSTTNETATGDDGDEVFVEAESEGISSEAGLEIDSQQEEEPVQASDESDLPSTSQDPPSSSSVDTSSSQPKPFRRVRLQTTLRQGVRGRQFNRQRGVSHAMGGRGGINRGNIN

11) >gi|42716275|ref|NP_057368.3| CCR4-NOT transcription complex subunit 1 isoform a [Homo sapiens] (266.8 kD)

MNLDSLSLALSQISYLVDNLTKKNYRASQQEIQHIVNRHGPEADRHLLRCLFSHVDFSGDGKSSGKDFHQTQFLIQECALLITKPNFISTLSYAIDNPLHYQKSLKPAPHLFAQLSKVLKLSKVQEVIFGLALLNSSSSDLRGFAAQFIKQKLPDLLRSYIDADVSGNQEGGFQDIAIEVLHLLLSHLLFGQKGAFGVGQEQIDAFLKTLRRDFPQERCPVVLAPLLYPEKRDILMDRILPDSGGVAKTMMESSLADFMQEVGYGFCASIEECRNIIVQFGVREVTAAQVARVLGMMARTHSGLTDGIPLQSISAPGSGIWSDGKDKSDGAQAHTWNVEVLIDVLKELNPSLNFKEVTYELDHPGFQIRDSKGLHNVVYGIQRGLGMEVFPVDLIYRPWKHAEGQLSFIQHSLINPEIFCFADYPCHTVATDILKAPPEDDNREIATWKSLDLIESLLRLAEVGQYEQVKQLFSFPIKHCPDMLVLALLQINTSWHTLRHELISTLMPIFLGNHPNSAIILHYAWHGQGQSPSIRQLIMHAMAEWYMRGEQYDQAKLSRILDVAQDLKALSMLLNGTPFAFVIDLAALASRREYLKLDKWLTDKIREHGEPFIQACMTFLKRRCPSILGGLAPEKDQPKSAQLPPETLATMLACLQACAGSVSQELSETILTMVANCSNVMNKARQPPPGVMPKGRPPSASSLDAISPVQIDPLAGMTSLSIGGSAAPHTQSMQGFPPNLGSAFSTPQSPAKAFPPLSTPNQTTAFSGIGGLSSQLPVGGLGTGSLTGIGTGALGLPAVNNDPFVQRKLGTSGLNQPTFQQSKMKPSDLSQVWPEANQHFSKEIDDEANSYFQRIYNHPPHPTMSVDEVLEMLQRFKDSTIKREREVFNCMLRNLFEEYRFFPQYPDKELHITACLFGGIIEKGLVTYMALGLALRYVLEALRKPFGSKMYYFGIAALDRFKNRLKDYPQYCQHLASISHFMQFPHHLQEYIEYGQQSRDPPVKMQGSITTPGSIALAQAQAQAQVPAKAPLAGQVSTMVTTSTTTTVAKTVTVTRPTGVSFKKDVPPSINTTNIDTLLVATDQTERIVEPPENIQEKIAFIFNNLSQSNMTQKVEELKETVKEEFMPWVSQYLVMKRVSIEPNFHSLYSNFLDTLKNPEFNKMVLNETYRNIKVLLTSDKAAANFSDRSLLKNLGHWLGMITLAKNKPILHTDLDVKSLLLEAYVKGQQELLYVVPFVAKVLESSIRSVVFRPPNPWTMAIMNVLAELHQEHDLKLNLKFEIEVLCKNLALDINELKPGNLLKDKDRLKNLDEQLSAPKKDVKQPEELPPITTTTTSTTPATNTTCTATVPPQPQYSYHDINVYSLAGLAPHITLNPTIPLFQAHPQLKQCVRQAIERAVQELVHPVVDRSIKIAMTTCEQIVRKDFALDSEESRMRIAAHHMMRNLTAGMAMITCREPLLMSISTNLKNSFASALRTASPQQREMMDQAAAQLAQDNCELACCFIQKTAVEKAGPEMDKRLATEFELRKHARQEGRRYCDPVVLTYQAERMPEQIRLKVGGVDPKQLAVYEEFARNVPGFLPTNDLSQPTGFLAQPMKQAWATDDVAQIYDKCITELEQHLHAIPPTLAMNPQAQALRSLLEVVVLSRNSRDAIAALGLLQKAVEGLLDATSGADADLLLRYRECHLLVLKALQDGRAYGSPWCNKQITRCLIECRDEYKYNVEAVELLIRNHLVNMQQYDLHLAQSMENGLNYMAVAFAMQLVKILLVDERSVAHVTEADLFHTIETLMRINAHSRGNAPEGLPQLMEVVRSNYEAMIDRAHGGPNFMMHSGISQASEYDDPPGLREKAEYLLREWVNLYHSAAAGRDSTKAFSAFVGQMHQQGILKTDDLITRFFRLCTEMCVEISYRAQAEQQHNPAANPTMIRAKCYHNLDAFVRLIALLVKHSGEATNTVTKINLLNKVLGIVVGVLLQDHDVRQSEFQQLPYHRIFIMLLLELNAPEHVLETINFQTLTAFCNTFHILRPTKAPGFVYAWLELISHRIFIARMLAHTPQQKGWPMYAQLLIDLFKYLAPFLRNVELTKPMQILYKGTLRVLLVLLHDFPEFLCDYHYGFCDVIPPNCIQLRNLILSAFPRNMRLPDPFTPNLKVDMLSEINIAPRILTNFTGVMPPQFKKDLDSYLKTRSPVTFLSDLRSNLQVSNEPGNRYNLQLINALVLYVGTQAIAHIHNKGSTPSMSTITHSAHMDIFQNLAVDLDTEGRYLFLNAIANQLRYPNSHTHYFSCTMLYLFAEANTEAIQEQITRVLLERLIVNRPHPWGLLITFIELIKNPAFKFWNHEFVHCAPEIEKLFQSVAQCCMGQKQAQQVMEGTGAS

12) >gi|7305053|ref|NP_038479.1| myoferlin isoform a [Homo sapiens] (234.6 kD)

MLRVIVESASNIPKTKFGKPDPIVSVIFKDEKKKTKKVDNELNPVWNEILEFDLRGIPLDFSSSLGIIVKDFETIGQNKLIGTATVALKDLTGDQSRSLPYKLISLLNEKGQDTGATIDLVIGYDPPSAPHPNDLSGPSVPGMGGDGEEDEGDEDRLDNAVRGPGPKGPVGTVSEAQLARRLTKVKNSRRMLSNKPQDFQIRVRVIEGRQLSGNNIRPVVKVHVCGQTHRTRIKRGNNPFFDELFFYNVNMTPSELMDEIISIRVYNSHSLRADCLMGEFKIDVGFVYDEPGHAVMRKWLLLNDPEDTSSGSKGYMKVSMFVLGTGDEPPPERRDRDNDSDDVESNLLLPAGIALRWVTFLLKIYRAEDIPQMDDAFSQTVKEIFGGNADKKNLVDPFVEVSFAGKKVCTNIIEKNANPEWNQVVNLQIKFPSVCEKIKLTIYDWDRLTKNDVVGTTYLHLSKIAASGGEVEDFSSSGTGAASYTVNTGETEVGFVPTFGPCYLNLYGSPREYTGFPDPYDELNTGKGEGVAYRGRILVELATFLEKTPPDKKLEPISNDDLLVVEKYQRRRKYSLSAVFHSATMLQDVGEAIQFEVSIGNYGNKFDTTCKPLASTTQYSRAVFDGNYYYYLPWAHTKPVVTLTSYWEDISHRLDAVNTLLAMAERLQTNIEALKSGIQGKIPANQLAELWLKLIDEVIEDTRYTLPLTEGKANVTVLDTQIRKLRSRSLSQIHEAAVRMRSEATDVKSTLAEIEDWLDKLMQLTEEPQNSMPDIIIWMIRGEKRLAYARIPAHQVLYSTSGENASGKYCGKTQTIFLKYPQEKNNGPKVPVELRVNIWLGLSAVEKKFNSFAEGTFTVFAEMYENQALMFGKWGTSGLVGRHKFSDVTGKIKLKREFFLPPKGWEWEGEWIVDPERSLLTEADAGHTEFTDEVYQNESRYPGGDWKPAEDTYTDANGDKAASPSELTCPPGWEWEDDAWSYDINRAVDEKGWEYGITIPPDHKPKSWVAAEKMYHTHRRRRLVRKRKKDLTQTASSTARAMEELQDQEGWEYASLIGWKFHWKQRSSDTFRRRRWRRKMAPSETHGAAAIFKLEGALGADTTEDGDEKSLEKQKHSATTVFGANTPIVSCNFDRVYIYHLRCYVYQARNLLALDKDSFSDPYAHICFLHRSKTTEIIHSTLNPTWDQTIIFDEVEIYGEPQTVLQNPPKVIMELFDNDQVGKDEFLGRSIFSPVVKLNSEMDITPKLLWHPVMNGDKACGDVLVTAELILRGKDGSNLPILPPQRAPNLYMVPQGIRPVVQLTAIEILAWGLRNMKNFQMASITSPSLVVECGGERVESVVIKNLKKTPNFPSSVLFMKVFLPKEELYMPPLVIKVIDHRQFGRKPVVGQCTIERLDRFRCDPYAGKEDIVPQLKASLLSAPPCRDIVIEMEDTKPLLASKLTEKEEEIVDWWSKFYASSGEHEKCGQYIQKGYSKLKIYNCELENVAEFEGLTDFSDTFKLYRGKSDENEDPSVVGEFKGSFRIYPLPDDPSVPAPPRQFRELPDSVPQECTVRIYIVRGLELQPQDNNGLCDPYIKITLGKKVIEDRDHYIPNTLNPVFGRMYELSCYLPQEKDLKISVYDYDTFTRDEKVGETIIDLENRFLSRFGSHCGIPEEYCVSGVNTWRDQLRPTQLLQNVARFKGFPQPILSEDGSRIRYGGRDYSLDEFEANKILHQHLGAPEERLALHILRTQGLVPEHVETRTLHSTFQPNISQGKLQMWVDVFPKSLGPPGPPFNITPRKAKKYYLRVIIWNTKDVILDEKSITGEEMSDIYVKGWIPGNEENKQKTDVHYRSLDGEGNFNWRFVFPFDYLPAEQLCIVAKKEHFWSIDQTEFRIPPRLIIQIWDNDKFSLDDYLGFLELDLRHTIIPAKSPEKCRLDMIPDLKAMNPLKAKTASLFEQKSMKGWWPCYAEKDGARVMAGKVEMTLEILNEKEADERPAGKGRDEPNMNPKLDLPNRPETSFLWFTNPCKTMKFIVWRRFKWVIIGLLFLLILLLFVAVLLYSLPNYLSMKIVKPNV

13) >gi|530383731|ref|XP_005267053.1| PREDICTED: afadin isoform X4 [Homo sapiens] (206.7 kD)

MSAGGRDEERRKLADIIHHWNANRLDLFEISQPTEDLEFHGVMRFYFQDKAAGNFATKCIRVSSTATTQDVIETLAEKFRPDMRMLSSPKYSLYEVHVSGERRLDIDEKPLVVQLNWNKDDREGRFVLKNENDAIPPKKAQSNGPEKQEKEGVIQNFKRTLSKKEKKEKKKREKEALRQASDKDDRPFQGEDVENSRLAAEVYKDMPETSFTRTISNPEVVMKRRRQQKLEKRMQEFRSSDGRPDSGGTLRIYADSLKPNIPYKTILLSTTDPADFAVAEALEKYGLEKENPKDYCIARVMLPPGAQHSDEKGAKEIILDDDECPLQIFREWPSDKGILVFQLKRRPPDHIPKKTKKHLEGKTPKGKERADGSGYGSTLPPEKLPYLVELSPGRRNHFAYYNYHTYEDGSDSRDKPKLYRLQLSVTEVGTEKLDDNSIQLFGPGIQPHHCDLTNMDGVVTVTPRSMDAETYVEGQRISETTMLQSGMKVQFGASHVFKFVDPSQDHALAKRSVDGGLMVKGPRHKPGIVQETTFDLGGDIHSGTALPTSKSTTRLDSDRVSSASSTAERGMVKPMIRVEQQPDYRRQESRTQDASGPELILPASIEFRESSEDSFLSAIINYTNSSTVHFKLSPTYVLYMACRYVLSNQYRPDISPTERTHKVIAVVNKMVSMMEGVIQKQKNIAGALAFWMANASELLNFIKQDRDLSRITLDAQDVLAHLVQMAFKYLVHCLQSELNNYMPAFLDDPEENSLQRPKIDDVLHTLTGAMSLLRRCRVNAALTIQLFSQLFHFINMWLFNRLVTDPDSGLCSHYWGAIIRQQLGHIEAWAEKQGLELAADCHLSRIVQATTLLTMDKYAPDDIPNINSTCFKLNSLQLQALLQNYHCAPDEPFIPTDLIENVVTVAENTADELARSDGREVQLEEDPDLQLPFLLPEDGYSCDVVRNIPNGLQEFLDPLCQRGFCRLIPHTRSPGTWTIYFEGADYESHLLRENTELAQPLRKEPEIITVTLKKQNGMGLSIVAAKGAGQDKLGIYVKSVVKGGAADVDGRLAAGDQLLSVDGRSLVGLSQERAAELMTRTSSVVTLEVAKQGAIYHGLATLLNQPSPMMQRISDRRGSGKPRPKSEGFELYNNSTQNGSPESPQLPWAEYSEPKKLPGDDRLMKNRADHRSSPNVANQPPSPGGKSAYASGTTAKITSVSTGNLCTEEQTPPPRPEAYPIPTQTYTREYFTFPASKSQDRMAPPQNQWPNYEEKPHMHTDSNHSSIAIQRVTRSQEELREDKAYQLERHRIEAAMDRKSDSDMWINQSSSLDSSTSSQEHLNHSSKSVTPASTLTKSGPGRWKTPAAIPATPVAVSQPIRTDLPPPPPPPPVHYAGDFDGMSMDLPLPPPPSANQIGLPSAQVAAAERRKREEHQRWYEKEKARLEEERERKRREQERKLGQMRTQSLNPAPFSPLTAQQMKPEKPSTLQRPQETVIRELQPQQQPRTIERRDLQYITVSKEELSSGDSLSPDPWKRDAKEKLEKQQQMHIVDMLSKEIQELQSKPDRSAEESDRLRKLMLEWQFQKRLQESKQKDEDDEEEEDDDVDTMLIMQRLEAERRARLQDEERRRQQQLEEMRKREAEDRARQEEERRRQEEERTKRDAEEKRRQEEGYYSRLEAERRRQHDEAARRLLEPEAPGLCRPPLPRDYEPPSPSPAPGAPPPPPQRNASYLKTQVLSPDSLFTAKFVAYNEEEEEEDCSLAGPNSYPGSTGAAVGAHDACRDAKEKRSKSQDADSPGSSGAPENLTFKERQRLFSQGQDVSNKVKASRKLTELENELNTK
[truncated: 36,275 more chars]
